# Use of community engagement interventions to improve child immunisation in low‐ and middle‐income countries: A systematic review and meta‐analysis

**DOI:** 10.1002/cl2.1253

**Published:** 2022-07-27

**Authors:** Monica Jain, Shannon Shisler, Charlotte Lane, Avantika Bagai, Elizabeth Brown, Mark Engelbert, Yoav Vardy, John Eyers, Daniela Anda Leon, Shradha S. Parsekar

**Affiliations:** ^1^ International Initiative for Impact Evaluation (3ie) New Delhi India; ^2^ International Initiative for Impact Evaluation (3ie) Washington USA; ^3^ Center for Effective Global Action University of California Berkeley Berkeley USA; ^4^ Department of Sociomedical Sciences Columbia University New York USA; ^5^ Independent Consultant (3ie) New Delhi India

## Abstract

Immunisation is one of the most cost‐effective interventions to prevent and control life‐threatening infectious diseases. Nonetheless, rates of routine vaccination of children in low‐ and middle‐income countries (LMICs) are strikingly low or stagnant. In 2019, an estimated 19.7 million infants did not receive routine immunisations. Community engagement interventions are increasingly being emphasised in international and national policy frameworks as a means to improve immunisation coverage and reach marginalised communities. This systematic review examines the effectiveness and cost‐effectiveness of community engagement interventions on outcomes related to childhood immunisation in LMICs and identifies contextual, design and implementation features that may be associated with effectiveness. We identified 61 quantitative and mixed methods impact evaluations and 47 associated qualitative studies related to community engagement interventions for inclusion in the reteview. For cost‐effectiveness analysis 14 of the 61 studies had the needed combination of cost and effectiveness data. The 61 included impact evaluations were concentrated in South Asia and Sub‐Saharan Africa and spread across 19 LMICs. The review found that community engagement interventions had a small but significant, positive effect on all primary immunisation outcomes related to coverage and their timeliness. The findings are robust to exclusion of studies assessed as high risk of bias. Qualitative evidence indicates appropriate intervention design, including building in community engagement features; addressing common contextual barriers of immunisation and leveraging facilitators; and accounting for existing implementation constraints and practicalities on the ground are consistently cited as reasons for intervention success. Among the studies for which we were able to calculate cost‐effectiveness, we find that the median non‐vaccine cost per dose of intervention to increase immunisation coverage by 1% was US $3.68. Given the broad scope of the review in terms of interventions and outcomes, there is significant variation in findings. Among the various types of community engagement interventions, those that involve creation of community buy‐in or development of new cadres of community‐based structures were found to have consistent positive effect on more primary vaccination coverage outcomes than if the engagement is limited to the design or delivery of an intervention or is a combination of the various types. The evidence base for sub‐group analysis for female children was sparse (only two studies) and the effect on coverage of both full immunisation and third dose of diphtheria pertussis tetanus for this group was insignificant.

## 1 PLAIN LANGUAGE SUMMARY

### 1.1 Community engagement interventions can improve immunisation outcomes

Community engagement interventions in low‐ and middle‐income countries (LMICs) appear to be effective in improving routine child immunisation coverage and its timeliness.

### 1.2 What is this review about?

Immunisation is one of the most cost‐effective interventions to prevent and control life‐threatening infectious diseases. Nonetheless, rates of routine vaccination of children in LMICs are strikingly low. This systematic review examines the effectiveness and cost‐effectiveness of community engagement interventions on outcomes related to childhood immunisation in LMICs.

In this review, community engagement refers to engagement in the design and/or implementation of the intervention, and as the intervention itself (engagement is embedded).

The review also identifies contextual, design and implementation features that may be associated with effectiveness.

**What is the aim of this review?**

This systematic review examines the effectiveness and cost‐effectiveness of community engagement interventions on outcomes related to childhood immunisation in low‐ and middle‐income countries. The studies included in this review are spread across 19 LMICs.

### 1.3 What studies are included?

The review synthesises evidence from 61 impact evaluations (IE) and 47 associated qualitative studies, as well as 69 project documents, across 19 countries. The cost‐effectiveness synthesis is based on 14 of the 61 IE which have the required combination of cost and effectiveness data.

### 1.4 What are the main findings of this review?

Community engagement interventions have a small but significant positive effect on all primary immunisation outcomes related to coverage and their timeliness. Sensitivity analyses excluding high risk of bias studies showed that the effect was slightly larger and still statistically significant for almost all the primary outcomes. The effects were also uniform across geographies and baseline immunisation rates.

Among the different types of community engagement interventions, the review finds that engagement as the intervention (embedded community engagement), which involves creation of community buy‐in or development of new community‐based structures, had consistent positive effects on more primary vaccination coverage outcomes than the others.

Qualitative evidence indicates that appropriate intervention design—including building in community engagement features, addressing common contextual barriers of immunisation and leveraging facilitators, and accounting for existing implementation constraints and practicalities—are associated with intervention success.

The median intervention cost per treated child per vaccine dose (excluding the cost of vaccines) to increase absolute immunisation coverage by 1% was US $.

### 1.5 What do the findings of the review mean?

Positive effects of these interventions can be expected across a variety of settings. Some engagement approaches appear to be more effective than others. The review provides evidence that features such as holding community dialogues or involving community leaders, and non‐community engagement features such as local supportive supervision and incentives to healthcare workers or caregivers are effective strategies across a wide range of settings and should therefore be integrated into these interventions.

Wherever possible, contextual barriers to immunisation, such as social norms and weak health systems, should be accounted for in the design of interventions. Existing contextual facilitators for immunisation, such as good existing health systems or high maternal education, could be leveraged for increasing intervention impacts. Important implementation pre‐conditions, such as regular internet service or sufficient staffing, should be assessed and established before implementation or addressed through the design itself. Close monitoring of intervention implementation along with a good understanding of context is important to help make modifications in case of unexpected challenges, such as political instability.

For improved understanding of causal mechanisms and resultant lessons, researchers should prioritise better reporting of interventions, more rounded analyses through mixed‐methods evaluations of why the interventions worked, and greater focus on intermediate outcomes. Also, researchers should collect high‐quality, comparable data on the cost of the intervention.

### 1.6 How up‐to‐date is this review?

The review authors searched for studies up to May 2020.

## 2 EXECUTIVE SUMMARY

### 2.1 Background

Immunisation remains one of the most cost‐effective interventions to prevent and control life‐threatening infectious diseases. Yet rates of routine vaccination of children in low‐ and middle‐income countries (LMICs) are strikingly low or stagnant, leading to a high disease burden and high infant and child mortality. In 2019, an estimated 19.7 million infants worldwide were not reached with routine immunisation services. Around 60% of these children live in 10 LMICs, including Ethiopia, India, Nigeria and Pakistan. Furthermore, national averages on immunisation coverage often obscure the underlying disparities within countries, and as a result, inequalities in immunisation often go unobserved or are underreported. Therefore, there is an urgent need for interventions that improve immunisation of children in these countries. A number of innovative approaches to this problem have arisen in recent years. These approaches include strategies and technologies that enhance communities' engagement in the planning, delivery, monitoring and uptake of routine vaccinations of children. These strategies have received considerable attention from funders, researchers, and practitioners. However, there is at present a dearth of rigorous and systematic evidence about the effectiveness and cost‐effectiveness of these interventions.

### 2.2 Objectives

This systematic review includes community engagement interventions that aim to improve low or stagnating immunisation rates for children in LMICs. The review applied a mixed methods approach that sought to understand the mechanisms and processes through which change happens, and to systematically identify the key factors that influence whether an intervention may be effective in a given context.

The review aimed to answer the following four questions:

- What evidence exists regarding the effectiveness of community engagement interventions in improving routine immunisation coverage of children in LMICs?
- Is there evidence for heterogeneous effects of community engagement strategies (i.e., does effectiveness vary by region, population, gender or programme implementation)?
- What factors relating to programme design, implementation, context, and mechanism are associated with better or worse outcomes along the causal chain? Do these vary by the kind l of community engagement?
- What is the cost‐effectiveness of different community engagement interventions in improving children routine immunisation outcomes?

### 2.3 Search methods

We implemented a systematic and comprehensive search strategy, developed in consultation with an information specialist, following the Campbell Collaborations' guidelines to systematic searching. We searched a range of databases and websites, including general sources of social science literature as well as sources specific to immunisation, vaccination and impact evaluation. We complemented this with citation tracking, checking reference list of included studies and existing reviews, and contacting experts. No language or publication restrictions were applied to the searches. The initial searches were conducted in May 2019 and an updated search was done in May 2020. At both the title and abstract and full‐text screening stages, all papers were double screened by two authors.

### 2.4 Selection criteria

To address questions of intervention effects we included quantitative IE using experimental designs or quasi‐experimental designs with nonrandom assignment that attempt to address confounding and selection bias in the analysis. To address questions related to intervention design, process and implementation we also included qualitative studies, project documents and process evaluations corresponding to the included evaluations. To address questions related to cost‐effectiveness we considered all economic information available in the included evaluations and the supporting cost documentation. Studies had to evaluate a community engagement intervention in countries classified by the World Bank as lower income, lower‐middle income, or upper‐middle income (LMICs), targeted at communities as a whole or selected community groups or members.

### 2.5 Data collection and analysis

To conduct this review, we developed a conceptual framework for various types of community engagement to assess the studies for inclusion. We consider three points within an intervention during which engagement can occur: engagement can occur in the design of the intervention, engagement can occur in the implementation of the intervention, or the intervention may be engagement (engagement is embedded).

To answer Review Questions 1 and 2, we conducted a detailed critical appraisal (risk of bias) and external validity assessment of the included studies, to assess the credibility of the findings. Effect size data measuring the change in outcomes in consistent units from each included impact evaluation was also extracted. We used statistical meta‐analysis to synthesise the findings. To structure the meta‐analysis, we conceptualised a community engagement framework that describes different types of engagement (engagement in intervention design, engagement in implementation autonomy of interventions, engagement as the intervention and multiple engagement types) and how might these impact the intermediate outcomes (like caregiver knowledge of and attitudes towards immunisation), final outcomes (like full immunisation coverage) and long term impacts (morbidity and mortality) along the causal chain.

To answer Review Question 3, a thematic analysis and synthesis of all included studies plus supplemental qualitative and programmatic documents was conducted, to systematically identify the key barriers, facilitators, uptake and fidelity challenges and the reasons for intervention success or failure that could explain why an intervention was more likely to achieve its expected results in a given context. Similar to the quantitative synthesis, all included qualitative papers were critically appraised for quality. The analysis also segregated the included papers and additional documentation based on the type of engagement. Finally, evidence on costs from the included IE and supplemental documentation was collected and analysed to answer Review Question 4.

### 2.6 Results

#### 2.6.1 Characteristics of included studies

The search returned over 46,000 papers, out of which 61 impact evaluation met the criteria for being included in the review, along with an additional five on‐going studies. For the included IE, authors undertook a targeted search and identified 47 qualitative papers and 69 project reports that were used to strengthen understanding of the design, context and implementation of the programmes.

Though mostly concentrated in South Asia (32) and Sub‐Saharan Africa (25), the studies included in this review are spread across 19 LMICs. Within these two regions, the majority of the studies were from India (21) and Nigeria (8). The majority of the studies largely fell into two engagement classifications, engagement as the intervention (engagement is embedded) (27) and engagement in design (16). There were 15 studies that fell into more than one engagement classification and were classified as having multiple engagement types. The remaining five studies were classified as engagement in implementation autonomy of interventions. Full immunisation coverage or FIC was the most commonly reported outcome (33) followed by DPT3 (27), measles (21) and Bacille Calmette–Guerin (BCG) (16). About 52 per cent of the studies included in the review were randomised controlled trials (RCTs). Of the remaining studies, the majority either employed a difference‐in‐difference estimation strategy (33%) or controlled‐before after study design (8%).

Regarding the characteristics of cost evidence, 22 of the 61 evaluations selected for inclusion in the systematic review reported some type of cost analysis. Of these, 18 used an experimental design and the remaining four used quasi‐experimental methods to identify the impact of treatment. Of the 22 studies: studies reported a total cost of the intervention; 12 included a cost‐effectiveness analysis; three included cost‐efficiency analysis; two studies included a cost–benefit analysis and two analysed cost per quality‐adjusted life year (QALY) or cost per disability‐adjusted life year (DALY).

#### 2.6.2 Quality of evidence base

The authors appraised the quality of both quantitative and qualitative as well as cost evidence base. The quantitative evidence was mostly low quality, though the randomised studies were generally of higher quality than quasi‐experimental studies. The randomised studies for the most part followed recommended allocation sequence methods ensuring comparability of intervention and control groups and that they have the same prognosis before the start of intervention. A majority of the quasi‐experimental studies were controlled before after studies and most of them either did not provide the required information or provided incomplete information on baseline balance between control and treatment arms or there was a high degree of imbalance at baseline leading to high risk or some concerns of selection bias. For most of the randomised and quasi‐experimental studies, we identified concerns related to the way vaccination coverage outcomes were measured. This was mainly due to the use of caregiver reported (self‐reported) measures of vaccinations received by a child in the absence and/or incompleteness of immunisation cards, which is common in LMICs and is often influenced by the intervention itself and subject to recall or social desirability biases.

Risk of bias assessments were conducted on the 47 included qualitative papers and their quality was generally high. Papers were scored as absent, weak, or present on 12 key elements and received corresponding scores of zero, one, or two for these ratings. Most (27) papers were missing at least some key elements, resulting in them receiving a zero value for these elements in their quality assessment scores. However, 18 of the 46 qualitative papers received quality assessment scores over 20, indicating that they received a value of two, or a ‘strong’ rating, for most key elements. The most common key elements to be missing were descriptions of sample characteristics and the analytic methods, with 17 studies failing to report on each of these.

The quality of the cost evidence of the 22 studies that included any kind of cost evidence was mixed. About half of the included evaluations appear to have carried out a planned, organised, cost analysis which included stating the form of economic evaluation, indicating the perspective from which the costing is carried out and describing the method of cost data collection. Indications of the quality of underlying cost data also are mixed—just over half (12) of the 22 studies reviewed in the cost analysis used expenditures (rather than budgets) to produce total cost estimates, whereas other studies simply did not report the source of cost data. About half of the included cost studies had high quality, detailed description of costs and cost analyses—which is essential for determining the comparability of total and average cost estimates.

#### 2.6.3 Review questions 1 and 2

The evidence indicates that community engagement interventions had a small but significant positive effect on all the primary immunisation outcomes related to coverage and their timeliness. The findings are robust to exclusion of studies assessed as high risk of bias. The average pooled effect on full immunisation coverage in the random effects (RE) model was an increase of 0.14 standard deviations units (95% confidence interval [CI]: 0.06, 0.23) across all kinds of community engagement interventions. The community engagement interventions also had positive and mostly significant effect on the timeliness of vaccinations and led to an average pooled increase of 0.15 standard deviation units (95% CI: 0.07, 0. 24) in the timeliness of full immunisation.

We found that among the four types of community engagement interventions, it was the engagement as the intervention (engagement is embedded), which involves creation of community buy‐in or development of new cadres of community‐based structures, that had consistent positive effects on more primary vaccination coverage outcomes than the other engagement types. For example, it had a small but positive and significant increase in full immunisation coverage by 0.08 standard deviations (95% CI: 0.03, 0.13). The evidence base for sub‐group analysis for female children was sparse (only 2 studies) and the effect on both full immunisation and DPT3 coverage for this group was insignificant.

#### 2.6.4 Review question 3

The qualitative synthesis highlighted the importance of accounting for contextual factors which could act as barriers or facilitators to immunisation. Several studies note that limited availability of services, especially insufficient staff and vaccine supply, were dominant barriers to immunisation, affecting outcomes in the early portion of the causal chain. There was more variation in barriers to related to social norms, fear, and an understanding of the importance of immunisations by type of engagement. Poor quality of services, including uninviting attitudes of health workers, posed a barrier to immunisation in communities that received engagement as the intervention or were engaged in the design of the intervention. Interventions that even acknowledge or address these barriers are likely to be more effective in improving outcomes. Studies also note that these very constraints could become facilitators or enablers of favourable outcomes provided a study has adequately situated itself to leverage them. Across all engagement types, most studies associated favourable social norms, caregivers' awareness and perception of the benefits of vaccination and high maternal education rates to improved immunisation outcomes.

In terms of programme design, intervention features or characteristics, across all engagement types, were often associated with intervention success or failure of an intervention. When it came to success, certain aspects of community engagement itself such as conducting stakeholder consultations, holding community dialogues or involving community leaders were associated with better immunisation outcomes. Studies also attributed intervention success to nonengagement intervention features including incentives given to caregivers, leadership and supportive supervision, which improved overall health service delivery and health worker performance. Among the studies which attributed intervention failure to intervention characteristics, inadequate duration frequency or exposure to the intervention were the most notable reasons.

Implementation failures, such as low fidelity, were a common reason for intervention failure. Across all engagement types, most studies did not properly account for realities on the ground and were forced to change their implementation plans. These issues may have cropped due to exogenous or uncontrollable factors or may have been related to invalid theory of change assumptions. These issues, in turn, may affect the early portion of the causal chain such intervention uptake.

This synthesis also tried to tease out factors associated with mechanisms of change. Studies reported that certain uptake and fidelity challenges may have resulted in inefficient mechanisms for change. For example, administrative challenges, particularly related to technical issues and communication, were common. There may be other mechanisms of change underlying the causal chain of an intervention. However, an extensive analysis was not possible due to limited reporting by studies on mechanisms of change and the factors influencing them.

#### 2.6.5 Review question 4

Among the studies for which we calculated cost‐effectiveness, we find that the non‐vaccine cost per dose of intervention to increase immunisation coverage by 1% averaged US $44.10 (all costs are reported in 2019 US dollars). This estimate of cost‐effectiveness excludes an outlier observation from Carnell (2014) for which the cost estimate is unreliable. There are two other outlier observations that drive up average cost‐effectiveness. The average cost per vaccine dose to increase immunisation coverage by 1% without the three outliers was US $3.97. The range of cost‐effectiveness estimates varied from a minimum of $0.89 to a maximum of $29.98 (the excluded estimate for Carnell was $641.08).

The cost‐efficiency of the interventions was measured by calculating the non‐vaccine intervention cost per vaccine dose. Cost‐efficiency ranged from a minimum of US $3.87 per vaccine dose to a maximum of US $5449 per vaccine dose. The average cost‐efficiency was US $42.34 per vaccine dose (when the maximum value is excluded). The observed variation in cost‐efficiency is at least partly explained by the inclusion of the most costly intervention which included health‐system building activities (i.e., building and staffing a health post) and is several orders of magnitude larger than the nearest estimate of the non‐vaccine intervention cost per vaccine dose.

### 2.7 Authors' conclusions

#### 2.7.1 Implications for policy makers and practitioners

Generally, community engagement interventions were successful in improving immunisation outcomes. The effects were robust to exclusion of high risk of bias studies. The effects were also uniform across geographies and baseline immunisation rates. However, some engagement approaches, like those with embedded community engagement, appear to be more effective than the others.

The results suggest it is important to pay particular attention when designing and implementing interventions in the following areas:

##### 2.7.1.1 Appropriate intervention design, including building in community engagement, can lead to intervention success

It was reassuring that the dominant reasons for project success were positive intervention features and not external factors. This implies that stakeholders can influence the success of their project and do not have to rely on random chance. Positive intervention features included local, supportive supervision; incentives for health care workers or caregivers; and health system integration and organisation. Many studies cited the effectiveness of the engagement strategy as a reason for the project success: dialogues developed into action plans (Andersson et al., 2009) and needs assessments resulted in practical adjustments (Modi et al., 2019). Policy makers and implementers can attempt to integrate these positive intervention features into their projects.

Methods for achieving sufficient intervention exposure should be integrated into the design of interventions. Some interventions did not report significant positive findings which the study authors attributed to inadequate exposure to the intervention. However, in the quantitative analysis, we did not find a significant impact of intervention exposure on the size of the effects. These intricacies may not be captured in simple quantitative measures of exposure to intervention in months. Nonetheless, some interventions may require a longer implementation period to build community trust and buy‐in which may be essential for its effectiveness to be visible.

##### 2.7.1.2 Addressing common contextual barriers of immunisation and leveraging facilitators may be useful in designing new interventions

Projects tended to fail because policy makers and implementers did not account for existing contextual constraints or uncontrollable trends. This finding is somewhat less positive because these external factors, such as those with political instability, may be impossible to overcome in some settings. Nevertheless, ongoing monitoring or understanding of context can help in risk mitigation by making necessary modifications to the intervention design or implementation mid‐way.

Other constraints, like social norms or weak health systems, may be addressed in intervention design. For instance, the sensitisation of heads of households may address constraints imposed by social norms about decision making in the utilisation of health care services (Oche et al., 2011). Also, Shukla (2018) shows that an intensive participatory strategy can be used to strengthen health system. Many community engagement interventions target awareness raising, although it is was not a commonly identified barrier to immunisation uptake.

Building on existing facilitators to immunisation may increase intervention impacts. Interventions which leverage facilitators may be more successful as a result of the presence of these facilitators, but are seriously threatened if the facilitators prove to be absent. For example, ‘good’ existing health care services can be leveraged in the implementation of community engagement activities; however, if the health care infrastructure turns out to be ‘not as good’ as originally assumed, then the intervention activities will fail to achieve their objectives. High maternal education may be leveraged in communication activities through the distribution of written materials. But, the distribution of written literature in areas where maternal education is low will be unsuccessful (‘Barriers to immunisatiopropriate intervention’ section).

##### 2.7.1.3 Implementation challenges could be avoided through appropriate intervention design

Policy makers and implementers should conduct scoping work to ensure that the interventions are practical to implement. Many interventions failed to account for existing implementation constraints and practicalities on the ground, such as limited cellphone service and insufficient staffing levels. This resulted in serious interruptions to implementation and low implementation fidelity (‘Uptake and fidelity challenges’ section) leading to inefficient use of limited resources. Wherever possible, existing implementation constraints should be accounted for in the design of interventions and should be addressed before implementation, rather than used to justify why interventions failed to reach their goals. Administrative challenges, such as delayed approvals from local authorities, can be overcome or avoided through designs that include close collaboration with local partners.

#### 2.7.2 Implications for research

While we found significant heterogeneity across a multitude of immunisation‐related outcomes, there were inconsistent effects related to moderators that might explain the variability among the studies. The risk of bias analysis has shown that, for the randomised studies, while the confounding bias is relatively low, researchers should rely less on self‐reported outcome measures of vaccination coverage, which are more susceptible to biases. A majority of non‐randomised studies were controlled before after designs and had a high risk of selection bias and confounding, due to incomplete or omission in reporting of baseline balance or having high baseline imbalance. There are therefore opportunities to conduct these studies with more rigorous evaluation designs. There are concerns related to reporting, in particular for quasi‐experimental studies there is a lack of transparency with regard to how authors addressed potential contamination or how authors responded to implementation problems (e.g., attrition).

Researchers should consider the following when undertaking IE in this area:

*Better reporting of interventions*: In many cases, we had difficulties in identifying precisely what the impact evaluation was evaluating due to limited reporting of the intervention components and characteristics. In addition, most studies lacked or had inadequate description and discussion of the intervention theory of change. This limits the amount of learning that can take place from the studies. Authors should consider drawing on tools such as The Template for Intervention Description and Replication (TIDieR) reporting guidelines for health.

*Consideration of equity*: There is a lack of research on how community engagement interventions affect immunisation outcomes by gender, income or for hard to reach populations. Sub‐group analysis needs to be incorporated right at the beginning of the evaluation and not as an afterthought. More disaggregated data by characteristics of interest, such as sex, socio‐economics status, religion, etc. is needed to draw valid conclusions about potential differences in the impacts of community engagement interventions on immunisations by them.

*Prioritisation of mixed‐methods IE and greater focus on intermediate outcomes*: Few studies incorporated qualitative research that would allow them to uncover the mechanisms that lead to the success or failure of the intervention. Drawing from qualitative work in mixed methods studies, evaluators should be sure to report on *why* they think their interventions worked, not just *if* the interventions worked. Moreover, greater focus on effect of interventions on intermediate outcomes, in addition to final immunisation outcomes, will improve the learnings on mechanisms of change. These crucial considerations will ensure the best use of their research in informing future policy and practice.

*Improved and standardised reporting of cost data and analysis*: Detailed cost data tables, with the clear descriptive information about the data, methods, unit costs of resources used, and cost adjustments that may have been performed on the cost data is not currently standard practice, but should be. Reporting of total costs, average costs, and marginal costs per impact, and the cost per vaccine dose per child all should be reported for evaluated immunisation programs. There is a wide range of available guidance to assist researchers in this reporting.

*Donors play a key role in driving the demand and allocating needed budget for sub‐group and cost analysis in IE*: Research teams are more likely to plan and report on sub‐group and cost analysis when the when the donor requires it —starting with a request for the relevant information in the Request For Proposal (RFP) and when donors enforce its reporting at program's end.

## 3 BACKGROUND

### 3.1 The problem, condition, or issue

Immunisation is one of the most cost‐effective interventions to prevent and control life‐threatening infectious diseases. From 2001 to 2020, projects that introduced or increased coverage of vaccines are estimated to have averted over 14 million deaths, 350 million cases of illness, 8 million cases of long‐term disability and 700 million disability‐adjusted life‐years (Ozawa et al., 2017).

Nonetheless, rates of routine vaccination of children in low‐ and middle‐income countries (LMICs) are strikingly low or stagnant. In 2020, an estimated 23 million infants did not receive routine immunisations. Around 60% of these children live in ten LMICs, including Ethiopia, India, Nigeria, and Pakistan as of 2020 (WHO, 2020a). Even though immunisation coverage in LMICs has increased in recent years, many WHO‐member countries still struggle to reach the target of 90% coverage for diphtheria, pertussis tetanus (DPT) and provide equitable access to life‐saving vaccines (WHO, 2019). Strengthening routine immunisation programmes is not sufficient to reach marginalised and vulnerable communities, which may also be geographically or socially secluded and are susceptible to being left out.

Therefore, there is an urgent need for interventions that improve the immunisation of children in LMICs. Common barriers to achieving universal immunisation coverage in LMICs include: (1) low rates of institutional births, which results in missed opportunities for vaccination; (2) poor infrastructure, which create logistical challenges for maintaining vaccination stocks and cold chains; (3) health professionals with low literacy and skill, which presents challenges for the delivery of quality immunisation services. However, there remains a dearth of evidence regarding how to address these barriers and increase immunisation rates.

### 3.2 The intervention

The focus of our review is community engagement interventions, which are increasingly being emphasised in international and national policy frameworks as a means to improve immunisation coverage and reach marginalised communities (UNICEF, 2018; WHO, 2015).

The most common approach for categorising community engagement interventions is probably the International Association for Public Participation (IAP2) framework (iap2. org), which identifies five levels of engagement ranging from inform to empower corresponding to increasing community influence over the decisions. This framework corresponds to the ‘social justice perspective’ in Brunton et al. (2017) in which the community engagement is rooted in concerns about social justice, which requires that the health needs are identified by communities themselves and they mobilise themselves into action to make changes within the community. However, after pilot testing of the IAP2 framework on a range of community engagement interventions, we determined that most interventions are based on a more ‘utilitarian perspective’. These two perspectives of community engagement are very well captured and articulated in Brunton et al. (2017), who in their systematic review of community engagement narratives in public health point out that:

Historically, interventions to promote health were driven by professionals, with little or no input from the targeted populations; more recently, community engagement has become central to national strategy and guidance for promoting public health, because, from a ‘utilitarian’ point of view, it is thought that more acceptable and appropriate interventions will result, which may result in improved service use and outcomes. Interventions that are based on a utilitarian perspective seek to involve communities to improve the effectiveness of the intervention. The intervention itself may be decided upon before the community is invited for its views; or, while the intervention itself is not designed by community members they may be involved in other ways, such as priority setting, or in its delivery. In utilitarian perspectives, health (and other) services reach out to engage particular communities that they have identified require assistance and the intervention is devised within existing policy, practice, and resource frameworks.

Due to the difference in how the IAP2 framework approaches community engagement and how interventions are actually implemented, it was difficult to systematically categorise interventions using the IAP2 framework with a high degree of consistency among coders. In addition, even for the interventions rooted in ‘social justice’ perspective, their description in the studies was too limited to identify them and map their intensity of community engagement without introducing a lot of subjectivity and non‐systematicness.

To avoid this misclassification as far as possible, we experimented with different frameworks and ultimately settled on the one which focuses on process of engagement rather than its intensity. We found that focusing on when and how the community is engaged is a more practical framework for the kind of community engagement interventions that have been evaluated in real world settings. Our approach corresponds to some degree to the ‘extent of engagement’ part of the conceptual framework developed in Brunton et al. (2017) and we also kept the spirit of IAP2 framework by including interventions in which engagement goes beyond one‐way communication, that is, beyond the inform level of IAP2, to include some consultation or dialogue with the community or some decision making by the community members. We found this approach to be relatively easier to apply, less subjective, less prone to classification error and potentially useful to practitioners. The development of this process‐oriented framework took a period of around 1 month and involved three of our core team members.

We consider three points within an intervention during which engagement can occur: engagement can occur in the design of the intervention, engagement can occur in the implementation of the intervention, or the intervention may be engagement. We break these categories into further sub‐groups representing specific ways through which engagement can occur; for example, the development of new cadres of health workers, pilot studies, and the involvement of the community in governance and decision making.

This framework adds a new dimension to the discussion of types of engagement. As practitioners consider the design of their programs, they can use the evidence provided through this framework to determine when and how best to engage with the community.

#### 3.2.1 Engagement as the intervention (engagement is embedded)

In these interventions a serious attempt was made to gain community buy‐in for activities or new cadres of community‐based structures were established, such as village health committees or community health volunteers. While, in a few cases, the development of new cadres can be solely for the purpose of didactic teaching and one‐way communication, it generally results in dynamic discussion and two‐way communication. As such, we chose to include these interventions. Interventions which are themselves community engagement can motivate communities to take ownership of service delivery and address local problems with local solutions.

#### 3.2.2 Engagement in the design of interventions

In these interventions community input or feedback was sought before the implementation of an intervention. Such feedback can take the form of a pilot, needs assessment, formative evaluation, or other outreach effort. This form of engagement must occur before the implementer undertakes an action. These interventions allow the community to influence the form of the ultimate action taken. Depending on the weight that is given to the feedback, these engagement activities can align to any of the IAP2 categories other than inform, which was not considered.

#### 3.2.3 Engagement in implementation autonomy of interventions

In many interventions the community is not asked for input in their design, but is utilised in their implementation as health care workers, facilitators, or problem solvers. In the spirit of IAP2 framework to go beyond inform level interventions, we only included those interventions in this category where the community members involved in the implementation of the intervention had some opportunity to affect or influence its implementation. This broadly aligns with the involve, collaborate and empower levels of engagement. Due to the inclusion criteria of some autonomy in implementation, the interventions under this category generally involved an existing community led governance structure which weighed in on implementation decisions or the community providing resources without which the intervention could not be implemented. We excluded interventions in which community members, like community health workers or frontline health workers, were involved in implementation, but no new cadres of health workers were created and they could not influence its implementation. For example, community health workers supplying hygiene kits or doing home visits. In addition, interventions which built capacity of existing cadres of community members or provided supportive supervision were excluded. For example, m‐health apps for community workers and training of peer facilitators or community health workers or frontline health workers that only allowed for a one‐way transfer of knowledge.

**Examples of engagement types**

ENGAGEMENT AS THE INTERVENTION (ENGAGEMENT IS EMBEDDED)

**Effect of health intervention integration within women's self‐help groups on collectivisation and healthy practices around reproductive, maternal, neonatal and child health in rural India**

Health‐focused self‐help groups were created for women of reproductive age in marginalised communities.

**Effect of peer education on knowledge, attitude and completeness of childhood routine immunisation in a rural community of Plateau State**

A new cadre of peer educators was created. Women were trained to provide women with information about routine childhood immunisation. This does not qualify as engagement in implementation because the intervention was the training of peer educators and not the subsequent actions of the peer educators.

**Impacts of engaging communities through traditional and religious leaders on vaccination coverage in Cross River State, Nigeria**

Traditional and religious leaders were trained to utilise their leadership role to support immunisation. After the training, these leaders then presented data at ward development committee meetings and engaged with the community to encourage immunsation.

ENGAGEMENT IN THE DESIGN OF AN INTERVENTION

**Cognitive behaviour therapy‐based intervention by community health workers for mothers with depression and their infants in rural Pakistan: a cluster‐randomised controlled trial**

Many pilots directly seek the feedback of community members. In this study, health workers and depressed mothers were asked about the relevance and usefulness of the intervention before scaling.

**Mobile Phone Incentives for Childhood Immunisations in Rural India**

Caregivers were given mobile phone credit incentives for completing immunisations. The amount of the incentive was decided upon through conversations that involved community members.

ENGAGEMENT IN THE IMPLEMENTATION OF AN INTERVENTION

**The impact of an immunisation programme administered through the Growth Monitoring Programme Plus as an alternative way of implementing Integrated Management of Childhood Illnesses in urban‐slum areas of Lusaka, Zambia**

The GMP+ sessions were conducted by medical personnel from Public Health Centers. During these session, community volunteers provided some operational and managerial support to ensure the effective implementation of the sessions.

**Effects of payment for performance on accountability mechanisms: Evidence from Pwani, Tanzania**

The intervention is a performance‐based financing mechanism. The community health committee was involved in decisions about how to spend the funds gained through the pay for performance mechanism.

Interventions are defined based on the actions of the external actors, that is, implementers, not the community itself. If an intervention spurred the community to take action, the categorisation of the intervention is based on the intervention which spurred the action and not the actions that the community took as a result of the intervention. This is because another community might take different actions as a result of the same intervention. The distinction is especially important for interventions to empower communities to improve their own systems. In these cases, the intervention is the activity that empowered the community; this intervention is engagement. For example, interventions which develop village committees to identify local challenges and solutions are themselves engagement interventions. This is because the intervention is the development of the committee and not the actions of the committee. A similar committee in a different area may choose to take different actions, even if the implementing agency does the same development process. Although the community may have engaged in the design of the committee, they likely were not engaged in the design of development process which brought about the committee. For interventions to be qualified as engagement in the design or implementation, the intervention must be an action by an implementer which the community influenced. For example, community members could be consulted in the design of materials used in outreach activities (Murthy, 2019; Nagar et al., 2020). In the example of the development of a new committee, the implementer influenced the action of the community, the community did not influence the action of the implementer.

We defined ‘communities’ in reference to the lowest level of the health service delivery system (or whatever level provides routine immunisation services in the local context). A community is a group of people who are served by a particular primary health facility. Thus, communities encompass a wide range of stakeholders, including caregivers, health service providers, and influential community members such as religious or other traditional leaders. Therefore, our review included any intervention that was directed towards any of these types of community members. Interventions that targeted higher levels of the health system, such as state‐level officials, were excluded.

### 3.3 How the interventions might work

A 2015 3ie scoping paper (Sabarwal et al., 2015) systematically mapped the literature on immunisation interventions involving community engagement. Several programme managers and policy experts provided insight regarding why community engagement could be the key to improving immunisation outcomes for children in areas where the coverage has stagnated or declined or that are hard to reach. The findings from the scoping study indicate that working with or engaging communities could help develop a better understanding of the context, target population, problems and barriers, and lead to identification of contextually relevant solutions and desired outcomes, and mobilising community support for them. Because individuals usually function under the influence of social norms, efforts to change these norms can be effective in changing behaviour (Bicchieri & Xiao, 2009; Reynolds et al., 2015). People respond to their peers and community and, while activities such as information and education campaigns might have some influence, individuals might feel bound by collective decisions, preventing sustained change (Riedy, 2012). The role of peers and of social norms in shaping attitudes towards vaccination is particularly important given that vaccine hesitancy has been documented in countries of all income levels (although it takes different forms in different countries; Dubé et al., 2014). Community engagement may be effective at overcoming these barriers to immunisation. In both high‐income countries (HICs; O'Mara‐Eves et al., 2013) and LMICs (De Buck et al., 2017), community engagement has been an effective model of modifying health behaviours in particular. Hence, community engagement could be an important determinant of success or failure of an intervention aimed at improving immunisation coverage.

Due to the variability in contexts, activities to address the barriers to immunisation through community engagement also vary. Therefore, no single theory of change can capture all the different ways that community engagement will affect immunisation outcomes. Furthermore, there is no strict correspondence between types of engagement and intervention activities. For the most part, each activity can be structured such that community engagement is the intervention (engagement is embedded) or the community is engaged in the design or implementation of the intervention. For example, consider an intervention involving village health committees in which community members are adequately represented. These committees can be created by a sponsoring agency with little or no input from the actual community. In this case, the intervention is engagement. On the other hand, community members may develop a village health committee during the design phase of major clinic infrastructure projects to ensure the efficient management of the intervention. Hence, these committees could arise from community involvement in the design of the intervention. In addition, an existing village health committee could be leveraged for the implementation of community meetings at which committee members lead a dialogue on immunisation.

**Figure 1:**
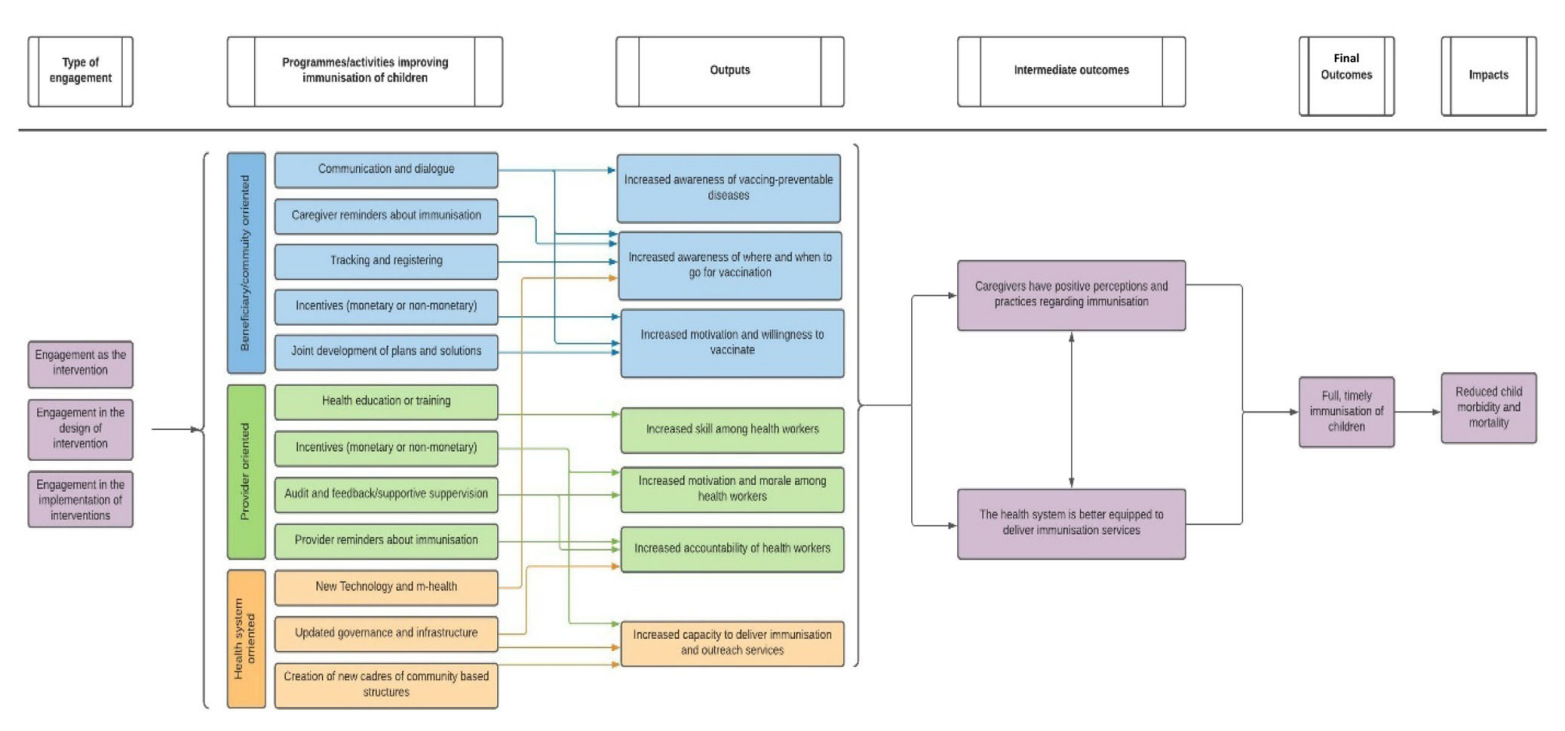
Intervention theory of change

Broadly, we expect that community engagement activities will increase awareness of vaccine‐preventable diseases, knowledge of where and when to get vaccinations, and motivation to get vaccinations among caregivers (Figure 1). Community engagement may also increase skill, motivation, and accountability of health workers. These ought to lead to improved demand for and delivery of services, which will increase the number of vaccinated children and could potentially reduce child morbidity and mortality. It is important to note that child mortality and morbidity are affected by critical factors beyond immunisation, such as access to safe food and adequate nutrition, safe water and quality care by a trained health provider when needed. Without these critical enabling factors immunisation alone may not be effective in reduction of child mortality and morbidity.

### 3.4 Why is it important to do the review

Community engagement approaches feature prominently in the global immunisation strategies (WHO, 2020c). Its role has become even more important in preparing and responding to COVID‐19 through both pharmaceutical and non‐pharmaceutical public health measures (UNICEF, 2020; WHO, 2020d). However, a systematic review of the impacts of community engagement interventions on immunisations outcomes in LMICs is lacking. This lack of evidence leaves policy makers, implementers, and other stakeholders without the information that they need to make decisions regarding the effective use of limited resources. They do not know what community engagement interventions work to increase childhood immunisations, for whom, or at what cost. Because LMICs face similar challenges in achieving universal immunisation coverage, a single review across contexts will successfully bring together the information needed by policymakers, implementers, funders, researchers, and other key stakeholders in the field. There are a number of existing systematic reviews (SR) that address the effectiveness of interventions to increase immunisation coverage. However, each has limitations that prevent it from being useful to stakeholders interested in understanding whether and how to pursue community engagement interventions to increase immunisation coverage in LMICs. Below we list 10 reviews and briefly document why they do not already accomplish the objectives of our review.

- Batt and colleagues (2004): This review covers only grey literature and is nearly 15 years old.
- Glenton and colleagues (2011): This review covers the use of lay health workers for increasing immunisation rates. While this overlaps with our focus, our review encompasses other types of interventions that encourage community participation beyond those involving lay health workers (e.g., interventions aimed at community leaders and caregivers). In addition, only about half of the studies included in this review were conducted in LMICs.
- Kaufman and colleagues (2018): This review covers interventions that provide face‐to‐face information or education to caregivers about vaccination allowing for real time dialogue. Our review includes interventions with any form of community engagement beyond just informing caregivers or other community members. It also includes those where community members may be involved in the design and implementation of the intervention. In addition, 7 of 10 studies included in this review were conducted in HICs.
- Lassi and colleagues (2015): Similar to our review, the scope of Lassi et al.'s review is related to both community engagement as an intervention strategy and child health as an outcome. However, Lassi et al.'s review focuses on neonatal health and thus excludes many immunisation‐related interventions.
- Mureed and colleagues (2015): The reporting in this review is limited, so it is not clear what interventions were included in the scope of the review. In addition, the review did not include grey or non‐English literature and lacks key policy‐relevant components such as causal path analysis and cost‐effectiveness information.
- Oyo‐Ita and colleagues (2016): This review is comprehensive and recent, covering a broad range of interventions targeting DPT immunisation in LMICs. However, this review covers only interventions that target either caregivers or health service providers, whereas our review will also include interventions aimed primarily at other community members (such as religious or other community leaders). Thus, an intervention that aimed to improve immunisation coverage by enlisting influential community members (who may not have young children themselves) to spread information about the importance of immunisation would not meet the intervention criteria reported in Oyo‐Ita et al.'s review but meets our scopes. Moreover, we look at outcomes beyond DPT3 in our review and use an analysis framework focusing on different kinds of community engagement that is different from the one used in this review.
- Pegurri and colleagues (2005): This review did not include grey literature and is over 10 years old.
- Ryman and colleagues (2008): This review is comprehensive and targets many of the same interventions as our proposed review (though without the specific focus on community‐participation strategies). However, it is now 10 years old.
- Saeterdal colleagues (2014): This review is relatively recent (and we understand an update will likely be published soon) and focuses specifically on interventions targeting communities. However, the interventions covered in this review address only one of the barriers to vaccination delivery: lack of knowledge or information. In contrast, our review includes interventions aimed at barriers all along the causal chain leading to vaccine delivery. For example, interventions that aim to motivate (caregivers to vaccinate or health workers to strengthen outreach efforts) would not fall under Saeterdal and colleague's scope but would be included in ours. In addition, our review will include multifaceted interventions, whereas the Saeterdal review will include such interventions only if the effects of the information/education component can be isolated. Thus, while Saeterdal and colleague's review did not include studies such as Bolam et al. (1998) or Olken (2014), these studies meet the intervention criteria for our review.
- Shea and colleagues (2009): This review is nearly 10 years old and focuses exclusively on demand‐side interventions, whereas our review covers all interventions targeting the caregivers, other community members or the health system as long as community engagement approaches have been used.

## 4 OBJECTIVES

Through this review, we aimed to add to the literature by collecting and synthesising all existing relevant evidence on the use of community engagement strategies to improve immunisation outcomes in LMICs. We also wanted to identify any evidence of adverse effects of community participation interventions. In particular, our objective was to answer following five questions:

- What evidence exists regarding the effectiveness of community engagement interventions in improving routine immunisation coverage of children in LMICs?
- Is there evidence for heterogeneous effects of community engagement strategies (i.e., does effectiveness vary by region, population, gender or programme implementation)?
- What factors relating to programme design, implementation, context, and mechanism are associated with better or worse outcomes along the causal chain? Do these vary by the kind of community engagement?
- What is the cost‐effectiveness of different community engagement interventions in improving children routine immunisation outcomes?

## 5 METHODS

The review adapted the Cochrane guidelines for RCTs and non‐randomised procedures to develop a standardised set of categories of bias. It also drew on the concepts of theory‐based impact evaluation (White, 2009) and theory‐based SR (Snilstveit, 2012; Waddington et al., 2012) to provide a mixed‐methods systematic review and analysis along the causal chain and to address questions related to intervention design, implementation and context. To respond to questions 1, 2 and 4, we relied on quantitative data and adopted quantitative methodological approaches. To respond to question 3, we used qualitative data and adopted qualitative methodological approaches. Therefore, our methods are presented separately for these research questions. This approach is similar to recent reviews published by the Campbell Collaboration (Snilsveit et al., 2019). Jain and colleagues (2020) described the protocol for this review (Table 1).

**Table 1:** PICOS inclusion and exclusion criteria

Characteristics	Inclusion criteria
Population	Rural, peri‐urban and urban populations living in low and middle‐income countries.
Interventions	Interventions involving community participation.
Comparisons	A comparison group or counterfactual that does not receive the intervention or business as usual.
Outcomes	Full, partial, timely immunisation of children and other outcomes such as morbidity, mortality, etc.
Study design	To answer questions 1, 2 and 4, experimental and quasi‐experimental studies.
	To answer question 3, qualitative studies, descriptive quantitative studies, process evaluations, project documents, formative research studies, protocols, baseline and midline and endline/final impact evaluation reports, policy briefs and website content.
Other	No inclusion restrictions by publication status or language.

### 5.1 Criteria for considering studies for this review

#### 5.1.1 Types of study designs

##### 5.1.1.1 Study designs to respond to quantitative research questions 1 and 2

To respond to research questions 1 and 2, we included experimental and quasi‐experimental studies that estimated the causal impact of an intervention by establishing a counterfactual. Quasi‐experimental designs were included because randomisation is often impractical or unethical in the context of development interventions. The following established methods for drawing causal inferences about intervention effects were included (Shadish et al., 2002):

1. Studies where participants were randomly assigned to treatment and comparison group (experimental study designs).
2. Studies where assignment to treatment and comparison group was based on other known allocation rules, including a threshold on a continuous variable (regression discontinuity designs) and where the assignment variable is not truly random allocation (e.g., using date of birth or patient registration number).
3. Studies with nonrandom assignment to treatment and comparison groups that include pre‐ and posttest measures of the outcome variables of interest to ensure comparability between groups on the baseline measure, and that use appropriate methods to control for selection bias and confounding. Such methods include statistical matching (e.g., propensity score matching, or covariate matching), regression adjustment (e.g., difference‐in‐differences, fixed effects regression, single difference regression analysis, and instrumental variables).
  3.1. Studies that did not perform a difference‐in‐differences estimation, but that presented data such that this estimate could be derived—that is, the studies presented measurements of the outcome both before and after the introduction of the intervention, for both an intervention and a control group.
4. Studies with nonrandom assignment to treatment and comparison groups that include posttest measures of the outcome variables of interest only and attempt to use methods to control for selection bias and confounding, as above. This includes pipeline and cohort studies.

Observational studies that did not report a pre–post comparison between treatment and control groups were excluded. Modelling based studies, commentaries and literature reviews were excluded.

In addition to using appropriate methods, studies had to be designed to measure the *effectiveness* of an intervention, rather than *efficacy*. Because there is a continuum from efficacy to effectiveness (Singal et al., 2014; Thorpe et al., 2009), we adapted a set of questions to guide the determination of effectiveness or efficacy. Strong, positive responses to these questions indicated that the study tested effectiveness; whereas, ambiguous or negative responses indicated that the study tested efficacy.^1^ Reviewers flagged any unclear cases for further review and discussion.

1. **Research Objective**: Does the study aim to determine how an intervention (e.g., technology, treatment, procedure or service) functions under ‘real‐world’ conditions, as opposed to how the intervention functions under ideal conditions (i.e., approximating the conditions that would inhere in a large‐scale rollout of the intervention)?
2. **Population**: Are the participants likely representative of the general population? Strict inclusion and exclusion criteria used to enrol a relatively homogenous population are characteristic of efficacy trials.
3. **Providers**: Is the intervention primarily delivered by those who might be expected to deliver the intervention under large‐scale rollout conditions (e.g., health workers, community leaders, or NGOs), rather than researchers?

##### 5.1.1.2 Study designs to respond to qualitative research question 3

To respond to our qualitative research question, we included additional papers that provided detail on programme design, implementation and context of the included IE. To be included, these additional papers had to be related to the programmes in the included IE and also be one or more of the following types of studies.

- A qualitative study collecting primary data using qualitative methods of data collection and analysis and reporting some information on the following: the research question, procedures for collecting data, sampling and recruitment, and sample characteristics.
- A descriptive quantitative study collecting primary data using quantitative methods of data collection and descriptive quantitative analysis and report some information on the following: the research question, procedures for collecting data, sampling and recruitment, and sample characteristics.
- Formative research work reporting on the feasibility, acceptability and appropriateness of a programme before it is fully implemented.
- Process evaluations assessing whether a programme was implemented as intended, which components were successful, and why certain components were successful (MRC framework) (MRC, 2015). Process evaluations may include the collection of qualitative and quantitative data from stakeholders regarding perceptions of intervention success or how an intervention was operationalised. They might also present organisational information.
- Project documents providing information about planned, ongoing or completed programmes. Project documents may describe the background and design of an intervention or the resources available for a project. These documents do not typically include much analysis of primary evidence, but they provide context for the included studies.
- Study protocols; baseline, midline, and endline reports; and IE.
- Policy briefs and website content providing project‐related insights or lessons learned from the programme implementation.

##### 5.1.1.3 Study designs to respond to quantitative research question 4

To address question 4 on cost‐effectiveness, we selected from among the set of studies that were included in response to quantitative research questions 1 and 2 that additionally had:

- Quantitative analysis of program costs, specifically, those evaluations that reported estimates of both costs and effectiveness.
- We included any studies that reported cost‐effectiveness ratios, any quantitative discussions of cost (and by type), any cost claims, any cost data tables and their contents.
- We additionally contacted the authors to obtain any additional cost data files or publications of cost‐evidence that were produced separately from the impact evaluation report.

#### 5.1.2 Types of participants

Rural, peri‐urban and urban populations in LMICs were considered. Country's income status was determined by its World Bank classification at the time an intervention was carried out. Our review focused on interventions targeting community members, but interventions need not target the community as a whole. Studies that targeted subgroups were included.

#### 5.1.3 Types of interventions

Interventions falling into one of the three types of community engagement outlined in Section 4.2 were included.

#### 5.1.4 Types of outcomes

The primary outcomes considered in this review are coverage rates for (a)full immunisation, (b) third dose of DPT or pentavalent, (c) measles, or (d) the timeliness of any of these doses. Additional antigen‐specific immunisation coverage outcomes and secondary outcomes reflecting upstream conditions (e.g., attitudes about vaccination and access to immunisation services) and downstream effects (e.g., morbidity and mortality) of the primary outcomes were also included. Some outcome measures differed in degree (full, partial or no routine immunisation) or were a subset of each other (dropout after DPT1/Penta 1 represents partial immunisation). These were included to account for differences in reporting. If heterogeneity in results was explored by subgroups, subgroup specific outcomes were considered.

Official health records and parent recall were considered acceptable measures of immunisation coverage. If a study reported both types of measures, we extracted data on both. However, we used the information from official health records (immunisation card) as the ‘true’ measure of the outcome.

Although there are standardised tools for measuring constructs like parental attitudes towards vaccination (e.g., Oladejo et al., 2016; Shapiro et al., 2018), these have not yet been universally adopted or validated. Therefore, we deferred to authors' definitions of these constructs and their instruments for measuring them. The list of outcomes along with their definitions is provided in Supporting Information: Appendix B.

##### 5.1.4.1 Primary outcomes

1. Full immunisation coverage (FIC)
2. Antigen specific immunisation coverage (e.g., DPT3 or penta 3 coverage, measles coverage)
3. Timely uptake of vaccines

##### 5.1.4.2 Secondary outcomes

1. Partial or no routine immunisation of children
2. Dropout rates for multi‐dose vaccines
3. Thinking and feeling (attitudes, confidence)
  a. Knowledge about immunisation
  b. Attitudes about immunisation
  c. Attitudes about health providers
4. Social processes
  a. Community norms
  b. Household norms and decision‐making
5. Readiness to vaccinate
  a. Readiness to vaccinate
  b. Reasons for not vaccinating
6. Practical factors
  a. Awareness of place, time, schedule for vaccination
  b. Actual cost of vaccinating
  c. Experience and satisfaction with health workers
  d. Vaccination health card availability/retention
  e. Perception of vaccination side effects
7. Health workforce
  a. Community health workers
    i. Community HW motivation, capacity and performance
    ii. Supply of CHWs
  b. Vaccinators
    i. Formal HW supply
    ii. Availability of HWs at vaccination point of service
    iii. Formal HW motivation, capacity and performance
  c. Administrators
    i. Admin staffing
    ii. Capacity of health admin. responsible for vaccination
    iii. Immunisation data collection (quality, completeness)
    iv. Defaulter tracing
    v. Supply chain management
    vi. Immunisation data availability/transparency
8. Vaccine availability
  a. Stockouts
  b. Quality of cold chain infrastructure
9. Resources
  a. National or subnational vaccine financing
10. Mortality
11. Morbidity
12. Cost‐effectiveness of interventions
13. Cost per additional child fully immunised
  a. Cost per additional child not dropping out from penta 1 to penta 3
  b. Cost per additional DALY averted
  c. Cost per additional death averted

#### 5.1.5 Duration of follow up

Because the timeline for administering most childhood vaccinations is short (most should be administered in the first 14 weeks after birth), there were no restrictions on duration of follow‐up.

#### 5.1.6 Types of settings

Studies must have been conducted in a LMIC. There were no additional restrictions on types of settings.

### 5.2 Search methods for identification of studies

#### 5.2.1 Search strategy to respond to quantitative research questions 1, 2, and 4

The search strategy for this systematic review was combined with that of an evidence gap map on the state of evidence in the immunisation sector. The EGM had a broad scope and included IE and SR, both completed and ongoing, that report findings on effectiveness of *any* intervention on immunisation outcomes of children in LMICs. It was developed in consultation with an information specialist.

##### 5.2.1.1 Electronic searches

We searched the academic databases and websites listed below on 17 May, 2019 and updated the search on 5 May, 2020. A full record of the applied search terms is provided in Supporting Information: Appendix C.

1. MEDLINE
2. CAB Global Health
3. EMBASE
4. Cochrane Controlled Trials Register (CENTRAL)
5. CINAHL
6. PsycINFO
7. Popline
8. Africa‐wide information
9. Academic search complete
10. Scopus
11. Campbell Library
12. Google Scholar
13. EconLit
14. IDEAS/RePEc
15. WHO Global Index Medicus
16. Pascal‐Francis
17. Open‐Grey
18. Grey Literature Report
19. Social Science Research Network (SSRN)
20. Eldis
21. GAVI
22. Epistemonikos
23. Innovations for Poverty Action (IPA)
24. Abdul Latif Jameel Poverty Action Lab (J‐PAL)
25. 3ie Impact Evaluation Repository
26. 3ie Systematic Review Repository
27. Registry of International Development Impact Evaluations (RIDIE)
28. Global Development Network
29. World Bank Development Impact Evaluation (DIME) and Impact Evaluation Policy Papers
30. Inter‐American Development Bank
31. Center for Global Development
32. Center for Effective Global Action (CEGA)
33. DFID Research for Development (R4D)
34. USAID

Website searches for capturing grey literature were carried out from January to May 2020.

##### 5.2.1.2 Searching other sources

We screened the bibliography of existing SR and literature reviews. We also performed backward‐ and forward‐citation tracking on all included studies, using Google Scholar for the latter. Finally, we contacted experts in the field to identify additional studies.

#### 5.2.2 Search strategy to respond to qualitative research question 3

After identifying included IE, we undertook a targeted search for supporting documentation related to the interventions evaluated in the included studies (Supporting Information: Appendix D). We searched clinical trial registries and databases using the names of programmes from included studies. We conducted targeted searches of websites of implementing agencies and funder websites.

#### 5.2.3 Search strategy to respond to cost analysis question 4

The evaluations included in the cost and cost‐effectiveness evidence were drawn from the 61 evaluations that were identified for the systematic review. Since cost reporting and analysis is often not required by donors or included in IE of global development interventions, we undertook structured outreach to the authors of the 61 included studies to request any additional cost evidence. In case the lead or corresponding author email addresses were nonfunctional, we contacted one of the co‐authors.

We successfully contacted 58 out of 61 study authors that reported intervention effectiveness only or intervention effectiveness and partial cost information and requested estimates of the intervention costs as well as any additional raw data files, summary tables, sensitivity analyses, cost evidence, published reports or notes, and non‐published documentation, methods or analyses relating to cost‐effectiveness. In the first two weeks of this request, our team obtained responses from a total of 25 study authors, of which 12 produced or pointed us in the direction of economic evidence for their respective interventions and 7 authors confirmed that any such costing analysis was not undertaken as part of their study. We excluded any studies that did not report estimates of both costs and effectiveness and identified 22 evaluations with both cost and effectiveness estimates.

### 5.3 Data collection and analysis

#### 5.3.1 Description of methods used in primary research

Quantitative methods were expected to rely heavily on cluster randomisation at the clinic level. Where randomisation was not possible, difference‐in‐difference and fixed effect estimation were likely to be used. Other methods, such as interrupted time series and synthetic control, were possible but expected to be less likely.

#### 5.3.2 Criteria for determination of independent findings

Statistically dependent effect sizes occurred when several publications used the same underlying data, multiple treatment arms were compared to a single control, similar analyses were conducted at different times, or the different outcome measures were used to reflect a single underlying construct (Borenstein et al., 2009).

To avoid double‐counting of evidence from different papers that focus on the same study, we linked these papers before analysis. We extracted data from the most recent publication when several publications reported on the same effect. After extracting the effect sizes from the main paper, we only extracted data on outcome measures and samples or subgroups from the linked papers that did not appear in the main paper.

Where studies reported data for the same outcome for an intervention over multiple time periods, we extracted data for each of the reported time periods. Selected time points that were most similar across papers for inclusion in the meta‐analysis. Where authors reported the same outcome using more than one analytical model, we extracted the data from the authors' preferred model specification. Where the authors did not specify a preference, we extracted data from the model with the most controls.

Where studies reported an index of different outcomes and the effects on the individual factors that comprised the index, we extracted data for the overall index measure. Where studies reported outcomes or evidence according to subgroups of participants, we extracted data on both the full sample (where possible) and on the individual subgroups, to answer Review Question 3 regarding differential effects by population type.

Where studies reported outcomes related to multiple treatment arms and only one comparison group, we extracted the data and estimated an effect size for each of the treatment arms. Further discussion of selection of independent effects can be found in Section 5.3.2.

#### 5.3.3 Selection of studies

Quantitative search results were screened on title/abstract by a team of trained reviewers. Two reviewers independently screened each abstract. After screening a ‘training set’ of 1000 random records, we activated EPPI‐Reviewer's machine learning algorithm (O'Mara‐Eves et al., 2015; Thomas et al., 2011), which ranked the remaining records according to their likelihood of inclusion, based on data from the training set. We then screened the prioritised list of records in order, with the machine learning function continually re‐prioritising the list based on the additional screening data. We intended to cease screening after reaching a point where 500 consecutive records had been excluded. However, this never occurred and all abstracts were screened.

During title and abstract screening, weekly reconciliation meetings were held to discuss and resolve disagreements. Studies included at the title/abstract level were retrieved for full review. Full texts were also independently screened by two reviewers, with disagreements resolved through discussion.

Qualitative papers were screened as they were identified in the search and reviewed again before they were fully coded.

### 5.4 Data extraction and management

#### 5.4.1 Quantitative data extraction

We used Microsoft Excel to extract descriptive information and effect sizes from included studies. This information was double‐coded using procedures set out in a codebook developed as part of the review protocol. Coders reconciled their answers. If an agreement could not be reached, a core team member was consulted. A third reviewer undertook spot checks for quality assurance (Supporting Information: Appendix E1). Cost data was single coded and checked by a core team member. The following broad categories of data were extracted (Supporting Information: Appendix E2):

- Descriptive data including authors, publication date and status, as well as other information to characterise the study including country, type of intervention and outcome, population, and cost study context including implementing partners, analytical perspective, intervention activities, intervention ingredients, intervention exposure, and cost data sources.
- Methodological information on study design, type of efficiency analysis, and type of comparison (if relevant).
- Information needed to convert costs into common values, including the base year of the costing, currency of expenditure, exchange rate, exchange rate year, inflation rate, and discount rate if reported.
- Key cost estimates, including total intervention cost (excluding the cost of vaccines), average costs and their denominators, cost per vaccine delivered, and average and marginal cost per immunised child.
- Quantitative data for outcome measures including outcome descriptive information, immunisation outcomes, including the types of vaccines, number of vaccine doses reported, baseline and final immunisation coverage, and the estimate program impacts on the immunisation status of participants in the treatment and control groups at baseline and endline.

We extracted effects reported across different outcomes or subgroups within a study, and where information was collected on the same programme for different outcomes at the same or different periods of time, we extracted information on the full range of outcomes over time. Where studies reported effects from multiple model specifications, we used the author's preferred model specification. If this was not stated or was unclear, we used the specification with the most controls. Where studies reported multiple outcomes or evidence according to subgroups of participants, we recorded data on relevant subgroups separately.

#### 5.4.2 Qualitative data extraction

All impact evaluation and additional documentation identified in the search were coded in Nvivo. CL developed an initial set of themes related to this study question with support from AB and MJ. Themes included barriers and facilitators, reasons for project success or failure, and uptake and fidelity challenges (Table 2). Other themes related to the data sources, research design and inclusion criteria were also included for potential future analyses (Supporting Information: Appendix E3). Four consultants were trained on the application of these thematic codes in Nvivo (various versions). Consultants selected all text related to the provided list of themes within the assigned documents. They were encouraged to add themes as they were identified, that is, deductive coding was applied. However, inductive coding was applied to the *Reasons for project success or failure* code because we did not have any a priori expectations and it contained more information than anticipated.

**Table 2:** Definitions and coding approaches used for the four sets of codes which are reported on in detail in this analysis

Code	Definition	Coding approach
Barriers to immunisation	Text related to reasons children were not vaccinated *before* the intervention.	An initial set of sub‐themes was developed a priori, but additional codes were added as new barriers were encountered.
Facilitators of immunisation	Text related to reasons children were vaccinated *before* the intervention.	Only the main theme was initially created with sub‐themes added as they were identified.
Reasons for intervention success or failure	Text reflecting the author's views as to why the intervention was successful or not.	CL applied inductive coding on a 75% sample of the papers. AB was informed of the codes developed and then conducted inductive coding on the full sample.
Uptake and fidelity challenges	Text related to challenges in intervention or research implementation.	An initial set of codes was determined a priori. We allowed for the opportunity to add codes; however, ultimately no additional codes were added.

For the majority of the coding period, AB and CL reviewed the work of consultants on a weekly basis and provided written and/or verbal feedback. After coding was completed, CL and AB reviewed the coding for each theme that would be analysed to ensure consistency across consultants and over time. Reviews were conducted by reading the Nvivo files; therefore, they largely resulted in the un‐coding or recoding of text that had been selected by consultants rather than the addition of new text. During the final review, redundant information related to the same impact evaluation, whether it was identified in the same paper or a different paper, was uncoded. Different pieces of information related to the same theme for the same evaluation were not uncoded. In some cases, uncoded text was added to the annotations of the coded text so that it could be retrieved later.

### 5.5 Assessment of risk of bias in included studies

#### 5.5.1 Assessment of risk of bias in IE

The risk of bias assessments for each included study were conducted by two independent reviewers, with disagreements resolved through discussion (and the core team member making final decisions on any contested cases). We used the Cochrane Non‐Randomised Studies Group and procedures recommended by Waddington et al. (2017) to develop a standardised set of categories of bias (Supporting Information: Appendix F1). We coded papers as ‘Yes,’ ‘Probably Yes’, ‘Probably No’, ‘No’ and ‘No information’, reflecting if each category was free from the bias. Overall risk of bias was scored as low when all domains were assessed as yes or probably yes, high when any domain was no or probably no, and some concerns when one or more domains had no information, but the remaining domains were yes or probably yes. We included all studies, regardless of risk of bias rating, in the synthesis, but we conducted a sensitivity analysis wherever possible (e.g., where there were at least two studies not assessed as high risk of bias) leaving out studies assessed as high risk of bias. We report both results for all instances where sensitivity analyses were completed.

- Bias arising from the randomisation process (for RCT's) or confounding bias (for quasi‐experimental designs)
- Bias due to missing outcome data (e.g., attrition)
- Bias due to deviations from intended interventions (e.g., performance bias, survey effects, or Hawthorne effects)
- Outcome measurement bias (e.g., social desirability or recall bias)
- Reporting bias

#### 5.5.2 Critical appraisal of qualitative studies

We critically appraised qualitative and mixed‐methods studies using an adaptation of the nine‐item framework developed by the Critical Appraisal Skills Programme (CASP 2018). The critical appraisal tool was piloted by C. L. and Y. V. to assess inter‐rater reliability of the tool. A. B., C. L., M. J., and Y. V. collectively refined the assessment tool pre and post pilot. Appraisals were done by Y. V. Detailed notes were kept while Y. V. conducted the appraisal and referred back to during two phases of self‐checking.

The tool consisted of 22 questions arranged according to the order in which a standard academic article is arranged: Introduction, Methodology, Results, Discussion (Supporting Information: Appendix F2). For each question there were three possible responses ‘strong’, ‘weak’, or ‘none’ with slight variations in phrasing to suit each question. Ethical approvals were coded as present/absent. Of the 22 questions, 12 represented key elements which we expected to be present in all qualitative papers. Scores were developed by assigning 2, 1, or 0 for each key element, corresponding to ‘strong’, ‘weak’, and ‘none’, and summing these values.

Documents that provided supporting descriptive or quantitative information on the design, delivery, or context of interventions did not undergo critical appraisal because these documents were not expected to fulfil the criteria of many of the questions in the appraisal tool. The purpose of including them in our review was to ensure we had all possible information about the context and interventions included in our review. These documents helped triangulate the information extracted from the quantitative and qualitative documents.

#### 5.5.3 Critical appraisal of cost evidence

For appraisal of cost evidence we assessed the quality of underlying cost data, reporting, and analysis using information that was included in the immunisation studies. We assessed risk of bias along six primary dimensions which were adapted from a combination of tools (Supporting Information: Appendix F3), including: Doocy and Tappis (2017); Campbell Collaboration Economic Methods Policy Brief (Shemilt et al., 2008); and Methods for the Economic Evaluation of Health Care Programmes (Drummond et al., 2015).

This risk of bias tool specifically assesses the bias that arises from the collection and reporting of cost analysis in conjunction with impact evaluation studies of global development interventions. These studies often do not incorporate cost analyses. Indeed recent estimates suggest just 15%–18% of impact analyses include any kind of cost analysis (Brown & Tanner, 2019). This under‐reporting of cost in conjunction with IE leads to very small samples from which to draw inferences about the cost, and cost‐effectiveness of development interventions. Moreover, often when estimates are included with IE, the quality of the underlying data is low. Cost may have been added as an ‘after‐thought’, rather than planned for in advance or treated as a research endeavour. Data sources for cost information may not be well‐documented or may have been estimated using ‘back‐of‐the‐envelope’ techniques (this phrase literally is used in write‐ups of cost methods). There is often insufficient detail in reporting of cost to assess the quality of estimates and data and basic robustness checks of the analysis are very infrequently performed.

Finally, this tool specifically considers the elements of cost and effectiveness that were extracted for the analysis of incremental cost effectiveness. Our particular measure estimates the non‐vaccine cost per dose of interventions to increase absolute immunisation coverage by 1%, using available data extracted from the evaluations wherever possible, or by adapt the steps and calculations as outlined in Ozawa et al. (2018). The specific inputs to this analysis include estimates of: total intervention cost (net of vaccine cost); the number of vaccine doses provided to each child; the endline proportion of children that received immunisations in the treatment and control groups; and the cost per child immunised.

##### 5.5.3.1 Dimensions of potential bias in costs reported and cost‐effectiveness estimates

5.5.3.1.1

**Planned, organised, cost analysis**. A common challenge of cost analysis is a lack of planning which often can lead to poor underlying data quality. For example, cost estimates are subject to recall bias when assessed long after the program and evaluation are completed. Therefore, we examine three indicators of an organised or planned cost analysis. Specifically, we look for a clear description of the form of economic evaluation, and a description of the method used. We also look for a clear statement of analytical perspective—which is the choice of which actor has standing in the costing—this may be the donor, the implementing partner, or perspective of the costing may be from a societal point of view. The analytical perspective is important because it determines whose costs and benefits will be counted in the costing. It may, for example, directly impact how the constituent components of total cost are counted. Although we considered a standard ROB question which asks: ‘Is a well‐defined research question posed in answerable form?’, we find that impact evaluation studies do not pose research questions in the expected form. The three questions, given equal weight in the risk of bias assessment, were:

a. Is the form of economic evaluation clearly stated?
b. Is the perspective of the costing stated?
c. Was a method of costing described?
**Quality data sources**. The highest quality data for assessing the cost of interventions in low‐and middle‐income settings often are drawn from the expenditure reports or accounting statements of the program implementers. Expenditures are better quality because they represent actual, rather than planned expenses and they often are subject to audit, making them more reliable as compared with cost data taken from program budgets (Levin et al., 2018).
d. What is the quality of the primary data sources used for the cost estimates?
**Descriptive, detailed cost information**. The quality of descriptive detail on costs allows judgement into cost components and whether they align with intervention activities. Since the three criteria tend to be positively correlated, we give equal weight to the three indicators of detailed cost information. Specifically, we assessed the descriptive detail of reported costs:
e. Whether costs are reported by ingredients (or input or resource).
f. Whether unit costs are reported.
g. Whether the information is presented in an organised cost table.
  We consider the **quality of cost estimates** that were key inputs to the cost synthesis, specifically
h. Total cost: What is the quality of the specific data components of total cost? Since total cost is a key input to the analysis we examine how it was reported. For example, some studies say simply, the total cost of the program was USD $3 Million. In the absence of descriptive information, that is, the elements that comprise total cost, it is difficult to assess the quality or reliability of the total cost estimate.
i. Vaccine cost: Can we tell if vaccines were excluded? Since we need to exclude vaccine cost, it must be excluded or reported separately from total cost to be valid in this study.
j. Cost per child immunised: If cost per child immunised was not directly estimated and reported, we take total cost divided by the number of children in the treatment group multiplied by the proportion of children that were immunised. If the quality of underlying data are poor, this estimate may be biased – with unclear magnitude and direction.
k. Number of vaccines. Was the number of vaccines reported from observation of study participants, or was it reported per protocol? When we estimate cost per vaccine dose, the cost estimate will be biased downwards (less costly per dose) if the study does not report the actual number of doses administered. This is because some children may already have received a part of the vaccine protocol before enroling in the study.
  Are **key cost details** reported which allow us to adjust for time and currency differences? In this assessment of bias risk, the responses are given equal weight since we need both elements to accurately adjust for time and currency differences.
l. Are details of inflation and currency conversion clearly stated?
m. Is the time horizon for costs clearly stated?
  We consider the **quality of cost analysis**, specifically:
n. Is sensitivity analysis conducted?

### 5.6 Measurement of treatment effect

For continuous outcomes comparing group means in a treatment and control group, we calculated the standardised mean difference (SMDs), or Cohen's *d*, its variance and standard error using formulae provided in Borenstein and colleagues (2009). A SMD is a difference in means between the treatment and control groups divided by the pooled standard deviation of the outcome measure. Cohen's *d* can be biased in cases where sample sizes are small. Therefore, in all cases we simply adjusted *d* using Hedges' method, adjusting Cohen's *d* to Hedges' *g* using the following formula (Ellis, 2010):

$$g≅d\left(1-\frac{3}{4\left(n_{1}+n_{2}\right)-9}\right).$$

In all instances, we chose the appropriate formulae for effect size calculations in reference to, and dependent upon, the data provided in included studies. For example, for studies reporting means (*x*) and pooled standard deviation (*SD*) for treatment (*T*) and control or comparison (*C*) at follow up only:

$$d=\frac{x_{\mathrm{Tp}+1}-x_{\mathrm{Cp}+1}}{\mathrm{SD}}.$$

If the study did not report the pooled standard deviation, we calculate it using the following formula:

$${\mathrm{SD}}_{p+1}=\sqrt{\frac{(n_{\mathrm{Tp}+1}-1){\mathrm{SD}}_{\mathrm{Tp}+1}^{2}+(n_{\mathrm{Cp}+1}-1){\mathrm{SD}}_{\mathrm{Cp}+1}^{2}}{n_{\mathrm{Tp}+1}+n_{\mathrm{Cp}+1}-2}},$$

where the intervention was expected to change the standard deviation of the outcome variable, we used the standard deviation of the control group only.

For studies reporting means ($\underline{X})$ and standard deviations (SD) for treatment and control or comparison groups at baseline (*p*) and follow up (*p* + 1):

$$d=\frac{{∆\underline{X}}_{p+1}-{∆\underline{X}}_{p}}{{\mathrm{SD}}_{p+1}}.$$

For studies reporting mean differences ($∆\underline{X})$ between treatment and control and standard deviation (SD) at follow up (*p* + 1):

$$d=\frac{{∆\underline{X}}_{p+1}}{{\mathrm{SD}}_{p+1}}=\frac{{\underline{X}}_{\mathrm{Tp}+1}-{\underline{X}}_{\mathrm{Cp}+1}}{{\mathrm{SD}}_{p+1}}.$$

For studies reporting mean differences between treatment and control, standard error (SE) and sample size (*n*):

$$d=\frac{{∆\underline{X}}_{p+1}}{\mathrm{SE}\sqrt{n}}.$$

As primary studies have become increasingly complex, it has become commonplace for authors to extract partial effect sizes (e.g., a regression coefficient adjusted for covariates) in the context of meta‐analysis. For studies reporting regression results, we followed the approach suggested by Keef and Roberts (2004) using the regression coefficient and the pooled standard deviation of the outcome. Where the pooled standard deviation of the outcome is unavailable, we used regression coefficients and standard errors or *t*‐statistics to do the following, where sample size information was available in each group:

$$d=t\sqrt{\frac{1}{n_{T}}+\frac{1}{n_{C}}},$$

where *n* denotes the sample size of treatment group and control. We used the following where only the total sample size information (*N*) is available, as suggested in Polanin and colleagues (2016):

$$d=\frac{2t}{\sqrt{N}}{\mathrm{Var}}_{d}=\frac{4}{N}+\frac{d^{2}}{4N}.$$

We calculated the *t*‐statistic (*t*) by dividing the coefficient by the standard error. If the authors only reported confidence intervals and no standard error, we calculated the standard error from the confidence intervals. If the study did not report the standard error, but report *t*, we extracted and used this as reported by the authors. In cases in which significance levels were reported rather than *t* or SE (b), then *t* was imputed as follows:

Prob > 0.1: *t* = 0.5

0.1 ≥ Prob > 0.05: *t* = 1.8

0.05 ≥ Prob > 0.01: *t* = 2.4

0.01 ≥ Prob: *t* = 2.8

Where outcomes were reported in proportions of individuals, we calculated the Cox‐transformed log odds ratio effect size (Sánchez‐Meca et al., 2003):

$$d=\frac{\ln(\mathrm{OR})}{1.65},$$

where *OR* is the odds ratio calculated from the two‐by‐two frequency table.

Where outcomes are reported based on proportions of events or days, we used the standardised proportion difference effect size:

$$d=\frac{p_{T}-p_{C}}{\mathrm{SD}(p)},$$

where *p*_*t*_ is the proportion in the treatment group and *p*_*c*_ the proportion in the comparison group, and the denominator is given by:

$$\mathrm{SD}(p)=\sqrt{p(1-p)},$$

where *p* is the weighted average of *p*_*c*_ and *p*_*t*_:

$$p=\frac{n_{T}p_{T}+n_{C}p_{C}}{n_{T}+n_{C}}.$$

An independent reviewer evaluated a random selection of 10 per cent of effect sizes to ensure that the correct formulae were employed in effect size calculations.

### 5.7 Unit of analysis issues

Unit of analysis errors can arise when the unit of allocation of a treatment is different to the unit of analysis of effect size estimate, and this is not accounted for in the analysis (e.g., by clustering standard errors at the level of allocation). We assessed studies for unit of analysis errors (The Campbell Collaboration 2019), and where they existed, we corrected for them by adjusting the standard errors according to the following formula (Hedges, 2009a, 2009b; Waddington et al., 2012):

$${\left(d\right)}^{\prime }=(d)\times \sqrt{\;1+(m-1)c},$$

where *m* is the average number of observations per cluster and *c* is the intra‐cluster correlation coefficient. Where included studies used robust Huber–White standard errors to correct for clustering, we calculated the standard error of *d* by dividing *d* by the *t*‐statistic on the coefficient of interest.

### 5.8 Dealing with missing data

In cases of relevant missing or incomplete data in studies identified for inclusion, we made every effort to contact study authors to obtain the required information. We contacted seven authors and received responses from six. For studies where we are unable to obtain the necessary data (*n* = 5), we reported the characteristics of the study but do not include these studies in the meta‐analysis or reporting of effect sizes due to missing data.

### 5.9 Assessment of heterogeneity

To provide an estimate of the amount of variability in the distribution of the true effect sizes, the amount of heterogeneity (i.e., $\tau^{2}$), was estimated using the DerSimonian–Laird estimator (DerSimonian & Laird, 1986). In addition to the estimate of $\tau^{2}$, the Q‐test for heterogeneity (Cochran, 1954) and the $I^{2}$ statistic (Higgins & Simon, 2002) are reported. We complemented this with an assessment of heterogeneity of effect sizes graphically using forest plots.

Whenever feasible, we conducted moderator analyses to investigate sources of heterogeneity. Following the PROGRESS‐PLUS approach (Oliver et al., 2017), we assessed moderators falling into three broad categories of extrinsic, methodological and substantive characteristics to address inequity aspects within the study context. The specific moderators we coded for analysis were:

- Total risk of bias score
- Exposure to intervention (in months)
- Evaluation period (number of months between end of intervention and data collection)
- Study design (experimental [0] vs. quasi‐experimental [1])
- Publication year
- Region (‘World Bank Country And Lending Groups—World Bank Data Help Desk’, 2021)
- Data source (comparing studies that used immunisation cards to studies using any other method, e.g., caregiver recall)
- Post‐intervention (0) versus change from baseline (1)
- Government implementation (whether the government was involved as an implementer, either alone or in conjunction with another agency, vs. any other implementer) (0 = no government involvement, 1 = government involvement)
- Whether the intervention created new cadres of health workers (0 = no, 1 = yes)
- Vaccine hesitancy (0 = absent as a barrier, 1 = present as a barrier)
- Baseline levels of vaccination coverage (only applicable for full immunisation and DPT3 outcomes)

We used RE meta‐regression to investigate the association between moderator variables and heterogeneity of treatment effects (Borenstein et al., 2009).

Our framework reflects a theoretical understanding of the process for community engagement. As such, the determination of which category each intervention fell into was largely a qualitative process. Quantitative analysis of the intervention types was used to determine the average impact of each intervention type on outcomes of interest.

### 5.10 Assessment of reporting biases

To reduce the possibility of publication bias, we searched for and included unpublished studies in the review. The rank correlation test (Begg & Mazumdar, 1994) and the regression test (Sterne & Egger, 2005), using the standard error of the observed outcomes as predictor, are used to check for funnel plot asymmetry (an indicator of publication bias) whenever there are at least 10 studies contributing to the analysis.

### 5.11 Data synthesis

#### 5.11.1 Review questions 1 and 2: Statistical meta‐analysis

Once all effect sizes were calculated and converted to a SMD, we examined the data for outliers. We defined outliers as any effect sizes ±3.29 standard deviations from the mean (Tabachnick & Fidell, 2001). Outliers were confirmed to be sure they were not errors or spurious.

We conducted meta‐analyses of all sets of two or more studies with sufficiently similar intervention, outcome, and comparison groupings. This approach was taken by Wilson and colleagues (2011). While we recognise the potential statistical bias in combining only two studies (namely that we cannot be certain about the dispersion of the effects), the conclusion we can draw from a statistical summary (albeit with limitations) is preferable to the unknown bias of an ad hoc summary that is less transparent and less valid (Borenstein et al., 2009; Ryan, 2016; Valentine et al., 2010). We used the metafor package (Viechtbauer, 2010) and/or the robumeta package (Fisher & Tipton, 2015) in R software to conduct the meta‐analyses (R Core Team, 2020). At the conclusion of the results section, we also include a table of the summary effects that has been converted to the estimated percent increase in the intervention group compared to the control group. This conversion is akin to the What Works Clearinghouse ‘improvement index’, whose computation is described in their Procedures Handbook, Version 4.0 (What Works Clearinghouse, 2017).

We dealt with dependent effect sizes by using robust variance estimation (RVE: Fisher & Tipton, 2015; Hedges et al., 2010) or data processing and selection techniques. The RVE approach allows us to use all available data in our effect size estimates, even data that is statistically dependent. However, these analyses must have >4 degrees of freedom to make valid inferences. In all cases, we first report the average estimated effects from the RE models using independent effects for the outcome category, and then separately for subgroup analyses by intervention type (and other subgroups where data are available) as this is our preferred model specification. The dependencies in our data arise most often due to multiple intervention arms compared to a single control, or due to several different data sources being used for estimation (e.g., immunisation card and caregiver recall). We believe we have chosen criteria for selection that increases the reliability of the data in our independent RE models (i.e., preferring immunisation card data over recall data) as well as selections that are most salient for practitioners (i.e., choosing the effect from the intervention i.e. being scaled up or that has been determined to be the most cost effective). While we will present the estimates for both, we will only present plots and figures from the models using traditional meta‐analysis with independent effects and will draw largely on the traditional meta‐analysis models for our discussion and conclusions. Following the presentation of the RE models we present the robust variance estimation for each outcome category (if sufficiently powered), as a robustness check.

The following data processing approaches were used to ensure the independence of data:

- **Several publications reported on the same study**: We used effect sizes from the most recent publication or used the one which contains more detailed reporting of results.
- **Outcome measures at different time points within the same study**: We followed De La Rue and colleagues (2014) and synthesised outcomes measured immediately after the intervention (defined as 1–6 months) and at follow‐up (longer than 6 months) separately. If multiple time points exist within these time periods, we used the most recent measure.

1. **Ongoing programmes**: In these cases, follow‐up reflections duration in a program rather than time since intervention. When such studies reported outcome measures at different time points, we used the follow up periods that were most similar across studies.
2. **Multiple outcome measures assessing related outcome constructs**: We followed Macdonald and colleagues (2012) and selected the outcome that appeared to most accurately reflect the construct of interest without reference to the results.
3. **Multiple treatment arms with only one control group**: We chose the treatment arm with the most components. When there were equal numbers of components, we used several additional criteria. First, if known, we used the treatment arm that had been selected for scale up (e.g., for Banerjee [2020] we utilised the ‘gossip’ arm because this is the arm that has been selected for scale‐up [J‐PAL 2020]). If the arm to be scaled up was not known, we chose the arm with the most benefit to the participant (e.g., in the case of a cash transfer program, the arm that offered the highest benefit). When choosing between a conditional and unconditional cash transfer program, we chose the unconditional program as it is more cost‐effective for the implementing agency.

#### 5.11.2 Review question 3: Qualitative synthesis

Qualitative analysis applied a logic model approach to comprehend underlying processes by which quantitative outcomes could have been achieved (Harden, 2017). This allowed for the qualitative research to directly relate to the quantitative work. Project context, design, and implementation can all affect outcomes along the causal chain. These factors are generally interrelated: project design should account for local context and implementation is influenced by context and design. To understand the interrelationship between these, we identified themes related to barriers to immunisation, facilitators of immunisation, reasons for project success, reasons for project failure, and uptake and fidelity challenges. In the discussion section, we then arranged these themes based on their relevance to context, design, and implementation.

Hierarchy charts related to barriers, facilitators, reasons for project success or failure, and uptake and fidelity challenges were created in Nvivo 12. They reflect the frequency at which a theme was reported by an impact evaluation or its supporting documents. The most frequently cited sub‐themes were selected for reporting. Quotes illustrating these themes were selected based on (1) their representativeness of the other coded text, (2) their ability to stand alone, without context, and (3) brevity. A mixture of direct quotes from study participants and author experiences were selected. Although information from literature reviews was coded, this was generally not included in the selected quotes because it represents secondary data.

#### 5.11.3 Review question 4: Cost analysis

We conducted an initial review and inventory of the cost and effectiveness evidence for each of the 22 evaluations using a CEA Data Inventory Tool. A single analyst conducted the inventory by reviewing the full‐text of the published impact evaluation and any additional evidence of cost or cost‐effectiveness that was collected. For each study, the analyst documented the evaluation outcomes, number of treated units, treatment arms; and created binary indicators (and documented the tables and page numbers) for any reported: cost‐effectiveness ratios, quantitative discussions of cost (and by type), any cost claims, any cost data tables and their contents. The analyst additionally documented and inventoried any additional cost data files and publications of cost‐evidence that were produced separately from the impact evaluation report.

The core team member reviewed all 22 of the evaluations selected for the cost and cost‐effectiveness evidence synthesis and the data inventory assembled by the team. This initial review confirmed wide variation in the quality and type of cost evidence reported in the evaluation studies. The core team member developed a data extraction tool which was piloted for initial data extraction on several studies by two independent analysts. The team collectively reviewed the extracted information and resolved any differences in the interpretation of the evidence through discussion.

Two analysts split the sample of 22 evaluations and independently extracted the data. The core team member independently reviewed the extracted data from each evaluation. For each of the included evaluations, we extracted the author, publication year, and country in which the evaluation took place. We extracted information on the context of the costing study, including the targeted population, implementing partners, analytical perspective, intervention activities, intervention ingredients, intervention exposure, cost data source, methods, and type of efficiency analysis. We extracted information necessary to convert costs into common values, including the base year of the costing, currency of expenditure, exchange rate, exchange rate year, inflation rate, and discount rate if reported. We extracted total intervention cost (excluding the cost of vaccines), average costs and their denominators, cost per vaccine delivered, and average and marginal cost per immunised child. We extracted immunisation outcomes, including the types of vaccines, number of vaccine doses reported, baseline and final immunisation coverage, and the estimate program impacts on the immunisation status of participants in the treatment and control groups at baseline and endline.

To ensure the comparability of the extracted cost elements, we converted expenditures in foreign currency to $US dollars using the official exchange rate (LCU per US$, period average) from the International Financial Statistics of the International Monetary Fund. Historical exchange rates were retrieved from the World Bank's World Development Indicators database.^2^ All costs were then to $US 2019 using annual averages of the seasonally‐adjusted Consumer Price Index for All Urban Consumers (CPI‐U) reported by the Bureau of Labor Statistics.^3^

The goal of the cost and cost‐effectiveness review was to compare the cost‐effectiveness of the included studies. However, a chronic and persistent challenge with assembling comparable cost‐effectiveness estimates is the inconsistency of reporting—both in terms of the elements of cost selected for reporting in each evaluation (e.g., do we report total costs, average cost or marginal costs) but also in terms of how those costs are analysed in tandem with impact estimates. Available guidance for the systematic review of economic evaluation is still under development. Chapter 20 of the Cochrane Handbook for Systematic Reviews of Interventions version 6.1 (updated September 2020; Aluko et al., 2020) has yet to provide guidance for conducting an integrated full systematic review of economic evidence.^4^

A few recent SR have begun to address the challenge of synthesising economic evidence. For example, Doocy and Tappis (2017) systematically review cash‐based approaches in humanitarian emergencies reporting total cost, cost‐effectiveness and cost‐efficiency where those values are computed by the study authors. The challenge of course is that only a modicum of evaluations actually report the key information. This limits the ability to synthesise the cost‐effectiveness evidence.

Several recent SR of immunisation evidence (Munk et al., 2019; Ozawa et al., 2012, 2018; Portnoy et al., 2021) begin to assemble cost evidence in ways that enable comparisons between evaluations. The analysis here adapts the approach applied in (Ozawa et al., 2018) to synthesise the cost‐effectiveness of the 22 evaluations that included elements needed to estimate the cost‐effectiveness of community participation interventions to improve child immunisation in LMICs.

We estimate the non‐vaccine cost per dose of interventions to increase absolute immunisation coverage by 1% using available data extracted from the evaluations wherever possible, or by adapting  the steps and calculations as outlined in Ozawa et al. (2018).

**Step 1: Extract total cost or estimate total intervention cost, if needed**.

A majority of the included evaluations do report an estimate of total program costs, excluding the cost of vaccines. In cases where vaccine costs were included we refer to detailed tables to subtract vaccine cost from the total. In the case where the total intervention cost is not reported we estimate total cost by multiplying the average cost per beneficiary by the number of individuals in the treatment group. If cost data only are reported for a single intervention arm, we estimate the total cost of treatment in that arm and report in separate lines for more multiple treatment arms.

**Step 2: Extract vaccine change in coverage or calculate change in vaccination coverage if needed**

A majority of the included evaluations report the difference in immunisation coverage between treatment and control groups. In this case, we estimate the change in vaccination coverage as the difference in the proportion of children *immunised* in the treatment group—the proportion of children *immunised* in the control group at endline. If these differences also are reported by treatment arm, we break out the reporting of coverage change by treatment arm. Several studies do not have sufficient information to calculate this change, in which case they are dropped from the analysis. In the case where studies report a null or negative effect, we do not calculate vaccine coverage change. Using the method described in Ozawa et al. (2018), we take the average of the difference in vaccination coverage in cases where vaccine coverage changes are reported for each vaccine type (i.e., separately for measles and DPT3, two common childhood vaccines). However in cases where the corresponding cost information also is available (and we can calculate the cost per vaccine dose), we calculate the cost per dose for the individual vaccines, rather than take the average. In with more than one intervention and for which authors report intervention costs and coverage information separately, we reported the multiple interventions separately.

**Step 3: Extract the number of vaccine doses delivered in each study and the cost per dose when reported, or calculate if needed**.

We extract the number of vaccine doses delivered when it is reported, and the cost per dose. However, in many cases the number of vaccine doses is not reported. Using the method described in Ozawa et al. (2018), we estimate the number of doses that a child in the requisite age group should receive per the appropriate country‐specific vaccination protocol that was in use at the time the study was conducted. We estimate number of vaccine doses per protocol (as described in the study itself or using standard protocols for time/country.

Using this information together with the coverage information, we estimate cost per dose (a measure of cost‐efficiency) by using the following approach:

$$\text{Cost}\;\text{per}\;\text{vaccine}\;\text{dose}=(C/n_{t})*\left(\frac{n_{t}^{v}}{n_{t}}\right)/v_{d},$$

where *C* is the total cost; *n*_*t*_ is the number treated; $n_{t}^{v}$ is the number pf treated beneficiaries who were vaccinated; $\left(\frac{n_{t}^{v}}{n_{t}}\right)$ is the proportion vaccinated; *v*_*d*_ is the number of vaccine doses for full treatment.

**Step 4: Calculate the non‐vaccine cost per dose of interventions to increase absolute immunisation coverage by 1%**.

#### 5.11.4 Subgroup analysis and investigation of heterogeneity

Heterogeneity in findings by type of engagement was examined. In the meta‐analysis, this was considered through subgroup analysis. In the qualitative analysis, this was done by developing hierarchy charts for each type of engagement and considering themes separately by type of engagement. For the meta‐analysis, four types of community engagement were considered: engagement as the interventions, engagement in the design of the intervention, engagement in the implementation of the intervention, and multiple levels of engagement. In the qualitative analysis, studies that used multiple types of engagement were considered to fall into each of the categories of engagement they used. This was because there was insufficient data to consider this group separately. In addition, contextual information found through the qualitative work is likely to apply equally to each type of engagement that a study used. Where qualitative findings were similar across levels of engagement, these are reported together; where qualitative findings are meaningfully different by level of engagement, results are presented in separate sections.

Wherever there were at least four studies contributing effects (for RE models with independent effects) and there was evidence of heterogeneity, that heterogeneity was examined through moderator analysis. In RVE analyses, moderator analyses were only conducted when there were at least 4 degrees of freedom for the predictor (when degrees of freedom are less than 4, we cannot make valid inferences). Where there were too few studies, or included studies were too heterogeneous in terms of interventions or outcomes, we present a discussion of individual effect sizes along the causal chain. As heterogeneity exists in theory due to the variety of interventions and contexts included, we used inverse‐variance weighted, RE meta‐analytic models (Borenstein, Hedges, Higgins & Rothstein, 2009).

#### 5.11.5 Sensitivity analysis of quantitative studies

We conducted sensitivity analysis to assess whether the results of the meta‐analysis were sensitive to the removal of any single study. We did this by removing studies from the meta‐analysis one‐by‐one and assessing changes in results. In the context of analyses using independent effects, studentized residuals and Cook's distances are used to examine whether studies may be outliers and/or influential in the context of the model (Viechtbauer & Cheung, 2010). Studies with a studentized residual larger than the 100×(1−0.05/(2×k))th percentile of a standard normal distribution are considered potential outliers (i.e., using a Bonferroni correction with two‐sided α=0.05 for k studies included in the meta‐analysis). Studies with a Cook's distance larger than the median plus six times the interquartile range of the Cook's distances are considered to be influential.

## 6 RESULTS

### 6.1 Description of studies

#### 6.1.1 Results of the search

**Figure 2:**
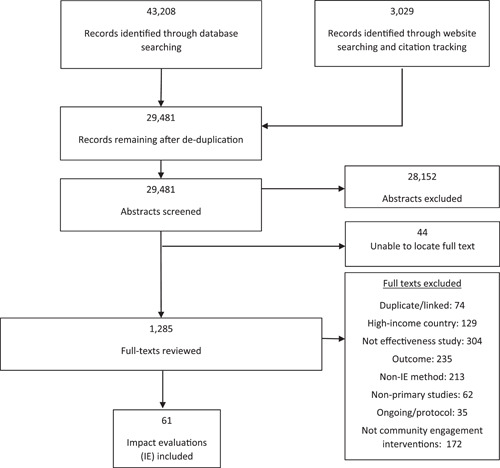
PRISMA diagram of search and screening process. PRISMA, preferred reporting items for systematic reviews and meta‐analyses.

Due to the broad initial search strategy of the EGM, we retrieved 43,208 results through our database and website searches and citation tracking. After de‐duplication, we identified 29,481 unique abstracts for screening. Of these, 1285 articles were screened at full‐text. After eliminating the studies that did not meet our scope, we arrived at 61 IEs that met our inclusion criteria for this review. Figure 2 summarises how the included studies were selected from the search results.

We identified 116 additional papers through the qualitative search process. The distribution of these papers by document type is provided in Table 3.

**Table 3:** Distribution of additional papers by document type

Document type	Number of studies
Cross sectional studies	14
Formative evaluation	6
Process evaluation	3
Qualitative studies	47
Theses, protocols and other associated IEs	17
Briefs/short communications/blogs	16
Reports	10
Cost‐effectiveness studies	3
Total	116

#### 6.1.2 Included studies

This section gives an overview of the characteristics of included studies, including geographic coverage, breakdown by community engagement type, outcomes and study design characteristics. A comprehensive overview of included studies is presented in Supporting Information: Appendix G: Table 1.

##### 6.1.2.1 Geographic distribution of the impact evaluation evidence base

**Figure 3:**
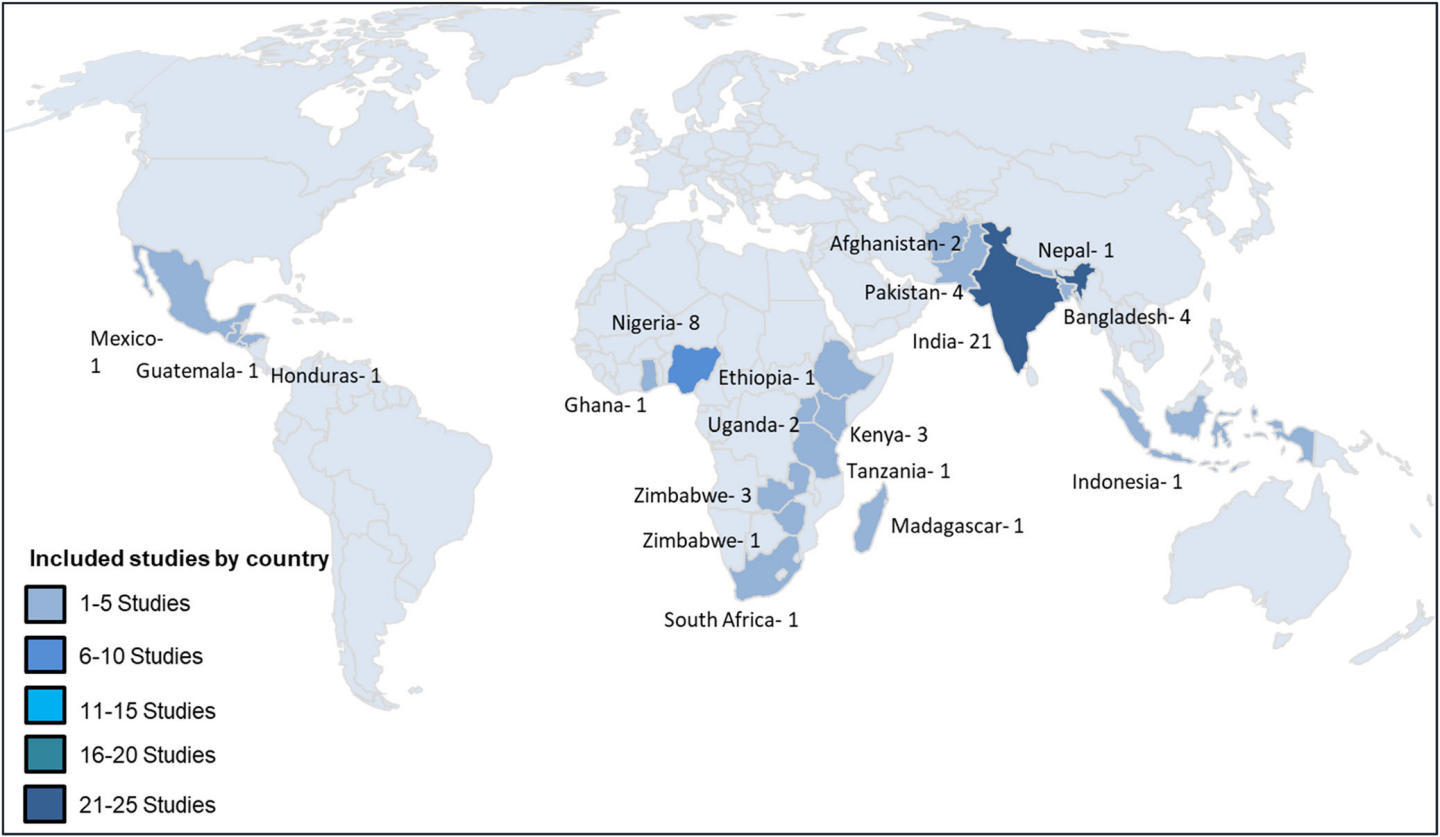
Geographic distribution of the studies and number of studies by country

Though mostly concentrated in South Asia (32) and Sub‐Saharan Africa (25), the studies included in this review are spread across 19 LMICs. Within these two regions, the majority of the studies were from India (21) and Nigeria (8). Figure 3 provides the geographic coverage of the included studies and number.

##### 6.1.2.2 Type of engagement

The majority of the studies largely fell into two engagement classifications, *engagement as the intervention* (25) and *engagement in design* (15). There were 16 studies that fell into more than one engagement classification and were classified as having *multiple engagement* types. The remaining five studies were classified as *engagement in implementation autonomy of interventions*. Table 4 provides a detailed breakdown of the studies by the type of engagement.

**Table 4:** Breakdown of the included studies by type of community engagement

Type of engagement	Number of studies
*Engagement as the intervention (engagement is embedded)*	1^a^
Developing community buy‐in	10
New cadres of health workers or formation of health committees^b^	13
Both	3
**Total**	**27**
*Engagement in design*	
Community decision making	0
Community feedback	
Formative evaluation or stakeholder consultation	11
Pilots	4
Needs assessment	0
Multiple^c^	1
**Total**	**16**
*Engagement in implementation autonomy of interventions*	
Governance and decisions	5
Provision of resources	0
**Total**	**5**
*Multiple engagement types*	
Engagement in design + engagement as the intervention	5
Engagement in design + engagement in implementation autonomy of interventions	1
Engagement as the intervention + engagement in implementation autonomy of interventions	9
**Total**	**15**

^a^ A study by Banerjee et al. (2020) evaluates the impact of three distinct interventions independently. Two of the interventions are included in our SR and have been assigned different engagement categories. Therefore, we have categorised this study in two intervention categories—engagement as intervention and engagement in design: community feedback. Similarly, another study Banerjee et al. (2010) evaluated the impact of two distinct treatments independently and both these treatment arms have been coded under separate community engagement categories. Because of this double counting, the total number of studies in this table is 63 and not 61.

^b^ These could also include identification or formation of cadres of health champions or volunteers from the community.

^c^ A study that employed more than two kinds of community feedback mechanisms.

##### 6.1.2.3 Outcomes

Table 5 presents the breakdown of studies by the primary outcomes of interest for all studies, including those that were not included in the meta‐analyses. Full immunisation coverage or FIC is the most commonly reported outcome (33) followed by DPT3 (27), measles (21) and BCG (16). A small but significant number of studies have also reported vaccination timeliness (12) and caregiver knowledge about immunisation (7). The complete list of outcomes reported by each study is presented in Supporting Information: Appendix G: Table 1.

**Table 5:** Breakdown of the included studies by type of outcomes^a^

Outcomes	Number of studies
*Primary outcomes*	
Full immunisation coverage (FIC)	35
DPT3	28
Measles	23
Vaccination timeliness	13
*Secondary outcomes*	
BCG	18
OPV0	8
OPV1	6
OPV2	6
OPV3	13
DPT1	14
DPT2	10
Morbidity	12
Mortality	6
Drop‐out rate for multi‐dose vaccines	6
No or partial routine immunisation	11
Knowledge about immunisation	9
Attitudes about immunisation	9
Attitudes about health providers	1
Health card availability	7
Formal HW motivation, capacity and performance	1
Stockouts	1
Community norms	4
Readiness to vaccinate	1
Household norms and decision‐making	1
CHW Capacity	1
Awareness of place, time, schedule for vaccination	1
Experience and satisfaction with health services	2
Supply of CHWs	2

^a^ A breakdown of the outcomes reported by all 61 included papers, regardless of whether or not they have been included in the meta‐analysis is provided. Thus, the number of studies per outcome here may be greater than the number of studies included in the meta‐analysis in some instances.

##### 6.1.2.4 Study design

About 52% of the studies included in the review were RCTs. Of the remaining studies, the majority either employed a difference‐in‐difference estimation strategy (33%) or controlled‐before after study design (8%). The breakdown of all studies by type of study design is given in Table 6.

**Table 6:** Breakdown of the included studies by type of study designs

Study design	Number	% of total
Randomised controlled trial (RCT)	31	50.82
Difference‐in‐difference (DID)	20	32.78
Controlled before‐after (CBA)	5	8.20
Instrumental variable (IV)	2	3.28
Propensity score matching (PSM)	1	1.64
Interrupted time series (ITS)	2	3.28
Total	61	100.00

Among the qualitative papers, 12 were mixed methods impact evaluation studies and 34 were qualitative papers associated with 17 IE. Seven IE had more than one qualitative paper and 44 IE had no associated qualitative papers.

##### 6.1.2.5 Research ethics

Of the 31 included RCTs, 25 specifically reported within their IE that they had obtained ethical clearance for their study. Quasi‐experimental studies were less likely to report obtaining ethical approval, with only 17 out of 30 authors confirming their studies had received ethical approval. There were two quasi‐experimental studies that discussed ethical approval, but concluded it was not necessary because either they were completing a secondary data analysis (Shukla et al., 2018) or a consultation with an ethics review committee determined approval was not required (Igarashi et al., 2010).

#### 6.1.3 Excluded studies

The main reason for exclusion at the title stage was irrelevance or not considering outcomes related to routine immunisation in LMICs (26,611). The most common reasons for exclusion at full text were not meeting the country (120), outcome (229), study design (338) or community engagement criteria (165).^5^ A complete breakdown of the reasons for excluding studies at various stages of screening is provided in Figure 2: the PRISMA flow.

### 6.2 Risk of bias in included studies

#### 6.2.1 Quantitative risk of bias

##### 6.2.1.1 Risk of bias in RCTs

**Figure 4:**
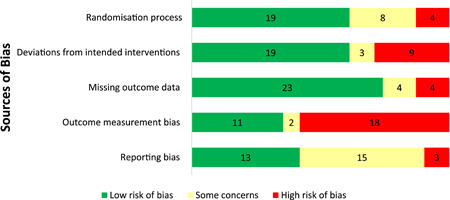
Frequencies of Bias in RCTs. RCT, randomised controlled trial.

Of the included studies with experimental designs (*k* = 31) the majority (*k* = 23) have been identified as being at high risk of bias, and only two were rated as having a low risk of bias. Six studies were assessed as having some concerns. Of the six potential causes of bias that were analysed, outcome measurement bias and deviations from intended interventions were the most commonly documented issues. The most common pitfall related to outcome measurement bias was the use of caregiver reported (self‐reported) measures of vaccinations received by a child in the absence and/or incompleteness of immunisation cards, which is common in LMICs and is often influenced by the intervention itself. One such study stated that ‘The calculation of FIC coverage was based on data from the child's immunisation card and/or the mother's recall. Mother's recall was taken in situations in which the card or specific data points on the card were missing’ (Gurley, 2020). Relatedly, Demilew (2021) reported that their outcome measures ‘…could be subject to recall bias or social desirability bias differentially by treatment group….’ Deviations from intended interventions had to do with issues such as potential spillover related to geography (e.g., in Siddiqi et al., 2020; centres were contiguously located) or transferable vouchers (e.g., Morris et al., 2004). Other sources of bias in included RCT studies are reporting bias, and bias in the randomisation process. Figure 4 illustrates the frequencies with which each cause of bias was identified across the 31 included RCTs.

##### 6.2.1.2 Bias in Quasi‐Experimental designs

**Figure 5:**
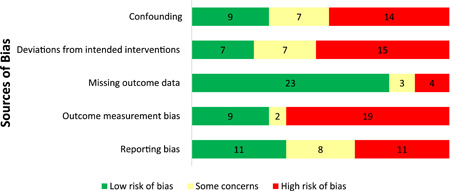
Frequencies of Bias in QEDs. QED, quasi experimental design.

Of the 30 included quasi‐experimental studies, the majority (*k* = 27) present a high risk for bias, two were assessed as low risk of bias and one study was assessed as having some concerns. Like the experimental studies the most common identified causes of bias amongst quasi‐experimental designs were issues related to outcome measurement bias and deviations from intended interventions. This is no surprise as self‐reporting was again a common concern (e.g., Admassie et al., 2009; Findley et al., 2013). Figure 5 illustrates the frequencies of other biases in our included quasi experimental designs (QEDs).

##### 6.2.1.3 Qualitative risk of bias

**Figure 6:**
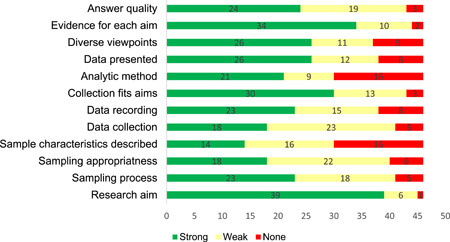
Distribution of papers by 12 key elements

**Figure 7:**
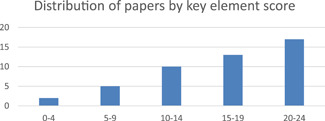
Distribution of qualitative assessment scores. Papers received scores of 0, 1 or 2 reflecting the strength of reporting on 12 key elements in the risk of bias assessment tool (Supporting Information: Appendix F2).

Risk of bias assessments were conducted on 47 qualitative papers. Papers were scored as absent, weak, or present on 12 key elements and received corresponding scores of zero, one, or two for these ratings. Most (27) papers were missing at least some key elements, resulting in them receiving a zero value for these elements in their quality assessment scores (Table 7; Figure 6). However, 17 received quality assessment scores over 20, indicating that they received a value of two, or a ‘strong’ rating, for most key elements (Figure 7). The most common key elements to be missing were descriptions of sample characteristics and the analytic methods, with 17 studies failing to report on each of these (Figure 6). The research aim was the most common key element to be stated strongly, with 39 papers clearly stating their aims.

**Table 7:** Distribution of papers by strength of key elements

	Number
One or more key elements absent	27
One or more key elements weak	15
All key elements strong	5

#### 6.2.2 Risk of bias in cost effectiveness estimates

The risk of bias analysis of the cost and cost‐effectiveness analysis assessed the quality of underlying cost data, reporting, and analysis using information that was reported in the immunisation studies. We assessed risk of bias along six primary dimensions which were adapted from a combination of tools, including: Doocy and Tappis (2017); Campbell Collaboration Economic Methods Policy Brief (Shemilt et al., 2008); and Methods for the Economic Evaluation of Health Care Programmes (Drummond et al., 2015), that were adapted for cost analyses carried out in conjunction with impact evaluation studies of global development interventions.

##### 6.2.2.1 Results

**Figure 8:**
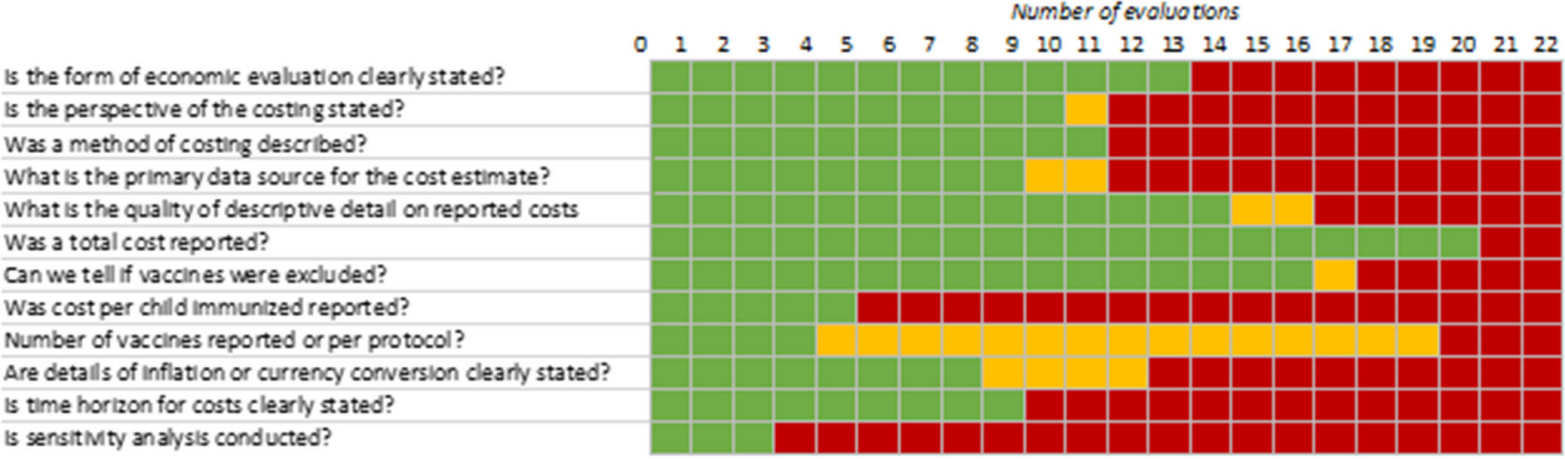
Risk of bias assessment of cost and cost‐effectiveness estimates used in the analysis. Green cells indicate that clear information was presented in the report to assess the risk of bias; yellow cells indicate incomplete information; and red cell indicate we found very little or no information to assess bias risk.

The risk of bias protocol was applied to the 22 immunisation evaluations that reported cost information. A summary of the six dimensions used to assess risk of bias is summarised below and results of this analysis are shown in Figure 8.

- Planned, organised, cost analysis. A common challenge of cost analysis is a lack of planning which often can lead to poor underlying data quality.
- Quality of data sources. The highest quality data for assessing the cost of interventions in low‐and middle‐income settings often are drawn from the expenditure reports or accounting statements of the program implementers, rather than from budgets or using back‐of the envelope estimates.
- Descriptive, detailed cost information. The quality of descriptive detail on the costs of intervention inputs allows judgement into cost components and whether they align with intervention activities.
- We consider the quality of other data elements that were key inputs to the cost synthesis, for example we specifically review the quality of total cost and its components; vaccine costs; the cost per child immunised; and the number of vaccines.
- The reporting of key cost details needed to adjust for time and currency differences.
- The analytical assessment of cost parameter and cost model uncertainty.

- *Just over half of the included evaluations appear to have carried out a planned, organised, cost analysis*. Thirteen of 22 evaluations clearly state the form of economic evaluation (i.e., cost‐effectiveness analysis); 10 studies report the perspective of the costing, which is key for judging the correct inclusion and exclusion criteria for the components of a total cost estimate; and 11 of 22 evaluations describe the method of costing that was used to collect cost data (i.e., the ingredients method).
- *Indications of the quality of underlying cost data are mixed*. Nine of the 22 evaluations used expenditure reports to generate cost estimates, two used budgets and the remaining 11 evaluations provided no information on the provenience of the underlying cost data that was used in the analysis.
- *The quality of the descriptive detail on reported costs was mixed. Just over half of all evaluations (14 of 22)* provided thorough, descriptive information on costs; two evaluations provided some descriptive information, for example, a breakdown of key unit costs; and six evaluations gave very minimal or no descriptive information on costs.
- *In 17 of 22 evaluations, we have high confidence that total cost excludes vaccines costs*. A majority of evaluations reported an estimate of total cost (20 of 22 evaluations). In 16 of the 20 total cost estimates, there were clear indications that vaccine costs had been excluded from the estimate, and in one case, vaccine costs were included but reported separately so that we could subtract vaccine cost from total cost to derive the comparable total cost. It was not possible to tell if vaccines were excluded from the total cost estimates of five evaluations.
- *The cost per immunised child* was only reported by the authors in 5 of 22 evaluations and for the remaining 16 was estimated by the authors to compile Table 15.
- *The number of vaccine doses received per treated child was reported in only four evaluations*; in 15 studies, vaccine doses per child were estimated based on information ‘per protocol’ which does not account for children's partial vaccination status at the point of enrolment in the study.
- *Fewer than half of the evaluations included the key information needed to adjust for time, currency, inflation or base year differences in the timing of expenditures*. This lack of reporting makes it very difficult to have complete confidence in the comparability of the cost estimates we generated.
- *Only three evaluations reported any kind of sensitivity analysis*, an indication of analytical robustness.

### 6.3 Synthesis of results

#### 6.3.1 Quantitative synthesis of results

Below we report the results related to our first review question, which aims to examine the evidence that exists regarding the effectiveness of community engagement interventions in improving routine immunisation coverage of children in LMICs. A total of 61 studies were determined to be includable based on full‐text screening, and were assigned to research assistants for double coding and data extraction. Out of the 61 studies assigned for coding, there were five that did not have sufficient quantitative data for us to be able to calculate an effect size. For example, the reports by Tandon and Sahai (1988) and Roy et al. (2008) did not provide the standard deviations necessary to compute a SMD, and Goel et al. (2012) did not report any sample sizes. Memon and colleagues (2015) reported regression coefficients, but did not report the associated standard errors, nor was there a p‐value reported from which we could approximate a *t*‐value. This left us with 56 studies includable in the quantitative analysis. Many studies reported on multiple immunisation‐related outcomes, thus the 56 studies contributed a total of 738 effect sizes. Throughout, where a study reports outcomes for which a negative movement actually indicates something positive (e.g., a reduction in diarrhea), the sign of the effect has been reversed. Thus, in all cases an increase indicates a positive impact of community engagement interventions.

We began with an overall test examining the impact of any type of community engagement intervention on any immunisation‐related outcome, using robust variance estimation to account for the dependency in the data (i.e., a single sample contributing multiple outcomes to the analysis). This omnibus analysis indicated that the estimated average outcome based on the random‐effects model was $\hat{\mu }=0.08$ (95% CI: 0.04 to 0.12), indicating a small but significant positive impact of community engagement interventions on child immunisation outcomes (p<0.001). Not surprisingly, there was a considerable amount of heterogeneity among the effects (*I* ^2^ = 85.41). Thus, we next examined a set of moderators that might explain the variability among the effects. For this overall analysis, the only significant moderator was publication year, such that studies published more recently showed smaller effects than older studies, with a reduction of about 0.02 standard deviations for each additional year ($\hat{\mu }=-0.02$ [95% CI: −0.03 to −0.004],p=0.02). Sensitivity analysis indicated the average effect was robust to all values of *ρ*.

Though the significant and positive movement on immunisation‐related outcomes is encouraging, it does not give us sufficient insights into what intervention types might work best for which outcomes. Thus, below we examine more closely the impact of community engagement interventions on specific outcomes, with subgroup analyses for specific intervention types. All moderators (listed in Section 5.9) were tested for each of the analyses below whenever the data allows (e.g., there is sufficient power to test, or enough studies per group to make a valid inference). The subsequent sections explore the following outcomes in turn: full routine immunisation (*k* = 28), partial immunisation (*k* = 9), measles vaccination (*k* = 20), BCG vaccination (*k* = 12), DPT 1 vaccination (*k* = 8), DPT 2 vaccination (*k* = 5), DPT 3 vaccination (*k* = 22), OPV0 vaccination (*k* = 5), OPV1 vaccination (*k* = 5), OPV2 vaccination (*k* = 5), OPV3 vaccination (*k* = 9), vaccination timeliness for all vaccinations (*k* = 11), timeliness of DPT3 (*k* = 7), timeliness of measles vaccination (*k* = 2), timeliness of full immunisation (*k* = 5), dropout rate (*k* = 5), childhood morbidity (*k* = 10), childhood mortality (*k* = 6), knowledge about immunisation (*k* = 9), attitudes about immunisation (*k* = 6), and vaccination health card availability/retention (*k* = 4). Within each outcome category, we examined the specific impacts of four broad categories of community engagement interventions using subgroup analyses: engagement as the intervention, engagement in the design of the intervention, engagement in implementation autonomy, and interventions with multiple engagement types.

There were several outcomes mapped in our initial framework that were either not reported in any of the included studies, or were reported in only one study and thus a synthesis was not possible. There were no included studies that reported on attitudes about health providers, readiness to vaccinate, reasons for not vaccinating, actual cost of vaccination, perceived convenience of vaccination, perception of vaccination side effects, formal health worker supply, availability of health workers at vaccination point of service, administrative staffing, capacity of health administrators responsible for vaccination, quality and completeness of immunisation data collection, defaulter tracing, supply chain management, availability and transparency of immunisation data, quality of cold chain infrastructure, national or subnational vaccine financing and IPV vaccination. There was only one study reporting on community norms around immunisation, household norms and decision making, awareness of place, time and schedule for vaccination, community health worker capacity, supply chain of community health workers and vaccine stockouts.

A summary of the results for all of the main analyses can be found in Table 8. A summary of all moderator analyses can be found in Supporting Information: Appendix I Table 3. Detailed results for the analyses are reported below.

**Table 8:** Summary of quantitative result

	Total sample		Engagement as the intervention	Engagement in the design	Engagement in implementation autonomy	Multiple engagement types
	RVE (dependent effects)	RE (independent effects)	RE (independent effects)	RE (independent effects	RE (independent effects)	RE (independent effects)
	*g*, [95% CI], *I* ^2^, (*k*; # of effects)	*g*, [95% CI], *I* ^2^, (*k*)	*g*, [95% CI], *I* ^2^, (*k*)	*g*, [95% CI], *I* ^2^, (*k*)	*g*, [95% CI], *I* ^2^, (*k*)	*g*, [95% CI], *I* ^2^, (*k*)
Full immunisation	0.11**, [0.04, 0.18], 87.27, (28; 53)	0.14**, [0.06, 0.23], 94.46, (28)	0.08**, [0.03, 0.13], 70.00, (12)	0.10*, [0.02, 0.19], 23.83, (5)	0.23, [−0.001, 0.47], 73.07, (2)	0.22, [−0.12, 0.56], 97.94, (9)
Partial immunisation	0.21*, [0.03, 0.38], 95.90, (9; 13)	0.23**, [0.09, 0.37], 96.35, (9)	0.31, [−0.25, 0.87], 98.96, (2)	0.14*, [0.01, 0.27], 0.00, (2)	N/A	0.28, [−0.01, 0.56], 97.05, (4)
Measles	0.06*, [0.01, 0.11], 72.46, (20; 34)	0.07**, [0.03, 0.11], 73.64, (20)	0.10***, [0.05, 0.15], 60.29, (10)	0.11*, [0.02, 0.21], 0.00, (2)	0.03, [−0.09, 0.15],54.84, (2)	0.03, [−0.10, 0.16], 86.76, (6)
BCG	0.04, [−0.02, 0.10], 79.17, (12; 16)	0.06*, [0.01, 0.11], 86.94, (12)	0.02***, [0.01, 0.03], 00.00, (4)	0.02, [−0.09, 0.13], 0.00, (2)	0.03, [−0.05, 0.11], 0.00, (2)	0.22, [−0.07, 0.52], 96.02, (4)
DPT1	0.01, [−0.06, 0.09], 62.55, (8; 21)	0.04, [−0.04, 0.11], 76.81, (8)	0.10, [−0.03, 0.22] 90.21, (3)	0.03, [−0.08.0.14], 0.00, (2)	N/A	−0.17**, [−0.29, −**0.05]**, 0.00, (2)
DPT2	Not powered	0.07*, [0.01, 0.12], 0.00, (5)	N/A	0.05, [−0.06, 0.16], 0.00, (2)	N/A	N/A
DPT3	0.10**, [0.05, 0.15], 76.42, (22; 36)	0.10***, [0.06, 0.14], 76.78, (22)	0.09**, [0.03, 0.15], 73.17, (6)	0.04, [−0.01, 0.08], 0.00, (6)	0.11, [−0.05, 0.28], 80.12, (3)	0.20**, [0.06, 0.34], 87.93, (7)
OPV0	Not powered	0.10, [−0.06, 0.26], 91.26, (5)	N/A	0.01, [−0.13, 0.14], 0.00, (2)	N/A	N/A
OPV1	Not powered	0.08*, [0.004, 0.15], 16.17, (5)	N/A	0.08, [−0.03, 0.19], 0.00, (2)	N/A	0.22, [−0.15, 0.59], 72.32, (2)
OPV2	Not powered	0.24**, [0.07, 0.40], 82.08, (5)	N/A	0.23, [−0.09, 0.55], 87.44, (2)	N/A	0.34***, [0.19, 0.50], 15.51, (2)
OPV3	0.24, [−0.02, 0.51], 96.42, (9; 10)	0.24**, [0.09, 0.40], 96.41, (9)	0.16, [−0.02, 0.34], 83.83, (3)	N/A	0.03, [−0.12, 0.18], 70.38, (2)	0.48, [−0.24, 1.20], 98.92, (3)
Timeliness (all)	0.11**, [0.06, 0.16], 39.92, (11; 54)	‐‐‐‐	‐‐‐‐	‐‐‐‐	‐‐‐‐	‐‐‐‐
Timeliness (DTP3)	No dependent effects	0.09**, [0.03, 0.14], 0.00, (7)	N/A	0.12**, [0.03, 0.21], 0.00, (4)	0.04, [−0.06, 0.13], 0.00, (2)	N/A
Timeliness (measles)	No dependent effects	0.23***, [0.14, 0.32], 0.00, (2)	N/A	N/A	N/A	N/A
Timeliness (complete immunisations)	No dependent effects	0.15***, [0.07, 0.24], 9.66 (5)	N/A	0.15*, [0.004, 0.29], 0.00, (2)	0.38, [−0.28, 1.03], 72.02, (2)	N/A
Dropouts	Not powered	0.03, [−0.11, 0.16], 91.08, (5)	0.02, [−0.03, 0.06], 0.00, (2)	N/A	N/A	0.18**, [0.06, 0.30], 57.38, (2)
Morbidity	0.01, [−0.09, 0.10], 83.32, (10; 26)	0.01, [−0.06, 0.08], 82.82, (10)	−0.004, [−0.11, 0.10], 88.86, (5)	0.05, [−0.06, 0.15], 45.71, (2)	N/A	−0.10, [−0.60, 0.40], 80.88, (2)
Mortality	−0.04, [−0.09, 0.01], 69.91, (6; 25)	−0.04, [−0.09, 0.01], 74.07, (6)	−0.04, [−0.11, 0.04], 80.68, (3)	N/A	N/A	−0.04, [−0.10, 0.03], 62.75, (3)
Immunisation knowledge	0.17, [−0.02, 0.37], 87.30, (9; 13)	0.19*, [0.07, 0.31], 87.28, (9)	0.20, [−0.08, 0.49], 93.80, (4)	0.31*, [0.04, 0.58], 72.34, (3)	N/A	0.09***, [0.04, 0.13], 0.00, (2)
Immunisation attitudes	No dependent effects	0.14, [−0.03, 0.31], 90.09, (6)	0.47, [−0.19, 1.13], 96.36, (2)	−0.11, [−0.53, 0.30], 75.61, (2)	N/A	0.06*, [0.01, 0.11], 0.00, (2)
Vaccination card availability/Retention	No dependent effects	−0.01, [−0.05, 0.02], 00.00, (4)	N/A	N/A	N/A	−0.01, [−0.05, 0.02], 00.00, (2)
Experience and satisfaction with health services	Not powered	0.04, [−0.015,.023], 48.22. (2)	N/A	N/A	N/A	N/A
Formal health worker capacity and performance	Not powered	0.11, [−0.07, 0.29], 0.00, (2)	N/A	N/A	N/A	N/A

*p < .05; **p < .01; ***p < .001.

##### 6.3.1.1 Full routine childhood immunisation

**Figure 9:**
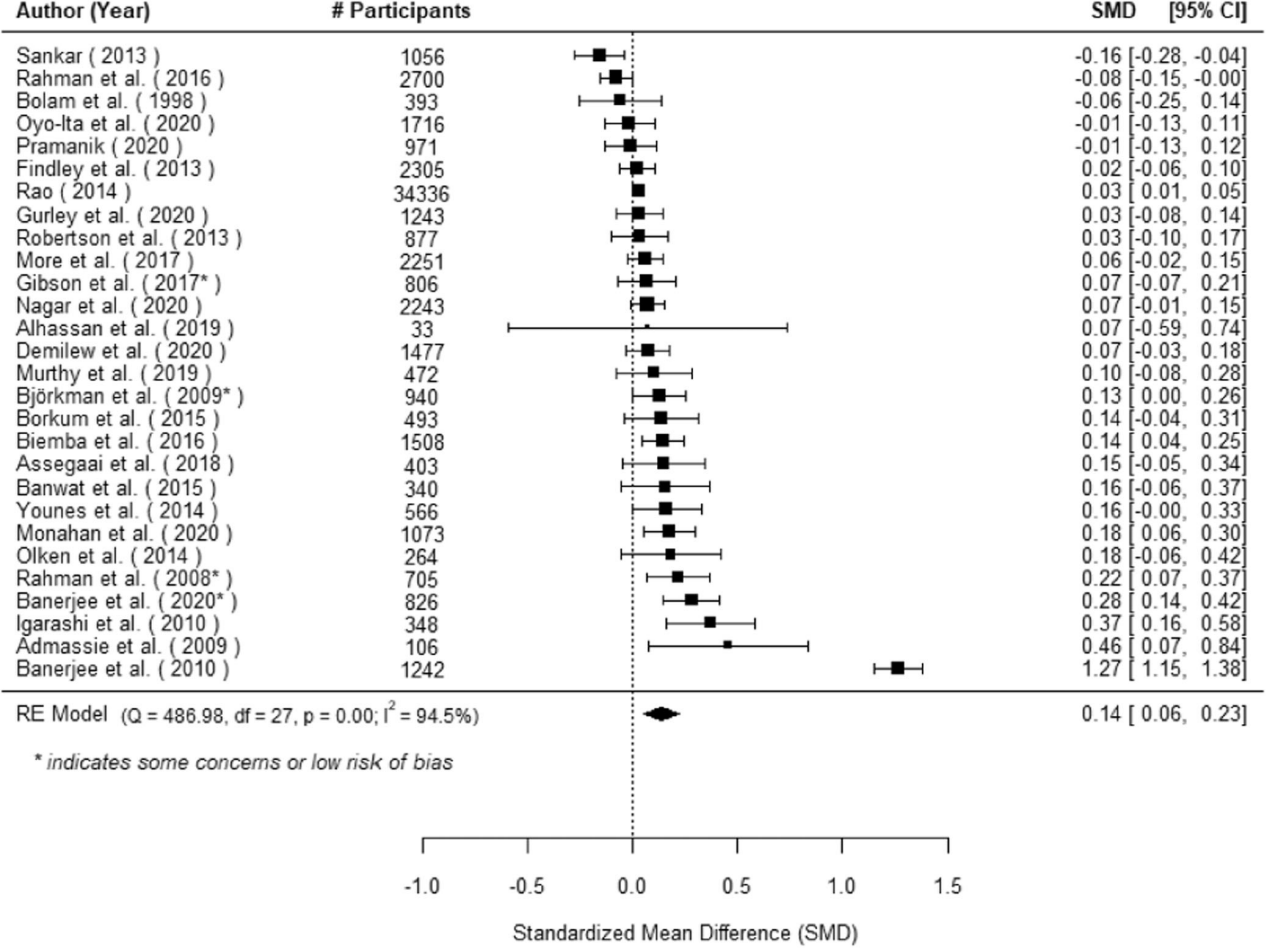
Forest plot showing the observed outcomes and the estimate of the random‐effects model for the impact of community engagement interventions on full childhood immunisation.

**Figure 10:**
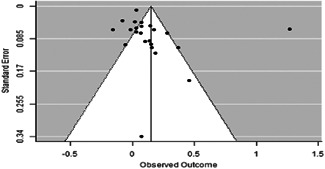
Funnel plot for studies examining the effect of community engagement interventions on full immunisation.

For full routine childhood immunisations, we examined all outcomes where the study reported on the full sample (i.e., we did not include effects for any subgroup analyses). A random‐effects model was fitted to the data, using only independent effects chosen as outlined in the methods section. A total of k=28 studies examined the relationship between community engagement interventions and full childhood immunisation. The estimated average outcome was $\hat{\mu }=0.14$ ([95% CI: 0.06 to 0.23], z=3.28, p=0.001), indicating a small but significant benefit for the treated group of.14 standard deviation units (see Figure 9). The rank correlation test indicated funnel plot asymmetry (p=0.03) but not the regression test (p=0.57; Figure 10). The true outcomes appear to be heterogeneous (Q(27)=486.98, p<0.0001, ${\hat{\tau }}^{2}=0.06$, $I^{2}=94.5$%). Outlier analyses revealed that one study (Banerjee, 2010) may be a potential outlier in the context of this model, and sensitivity analyses leaving each study out indicated that removing Banerjee (2010) would reduce the overall average effect (μ = 0.08 [95% CI: 0.04 to 0.12]), but the effect would still be positive and significant (z = 4.12, p < 0.001).

We conducted sensitivity analyses to examine the robustness of the results to the exclusion of low‐quality studies. When studies assessed as high risk of bias were removed (leaving four medium/high quality studies), the resulting effect was slightly larger and still statistically significant ($\hat{\mu }=0.18$ [95% CI: 0.08 to 0.27]), z=3.67, p<0.001). We examined several potential sources of heterogeneity, including exposure to the intervention, evaluation period, study design, year, region, data source, whether the intervention was implemented by a government agency (either alone or in combination with another agency), whether new cadres of health workers were established, presence of vaccine hesitancy, and baseline vaccine coverage rates. There were no significant moderators in the context of this model (see Supporting Information: Appendix I Table 3).

As a robustness check, we used robust variance analysis and included all dependent effects in the analysis, totalling 53 effects from the same 28 studies (*df* = 25.5). The overall average effect was slightly smaller but still significant ($\hat{\mu }=0.11$ [95% CI: 0.04 to 0.18], *p* = 0.002). Sensitivity analyses show the effect to be sensitive to all values of *ρ*. When using robust variance estimation, one can only make valid inferences from the results if there are at least 4 degrees of freedom for the moderator test. For this analysis, all moderators except for evaluation period and publication year met this criteria and thus were tested as potential sources of heterogeneity, but none were significant predictors of the variation among effects (see Supporting Information: Appendix I Table 3).

Next, we examined full immunisation outcomes in the context of the four types of community engagement interventions. All subgroup analyses were conducted using the RE models where all effects are independent. We did not do robustness checks using RVE for subgroup analyses.

###### 6.3.1.1.1 Effects of engagement as the intervention on full childhood immunisation

**Figure 11:**
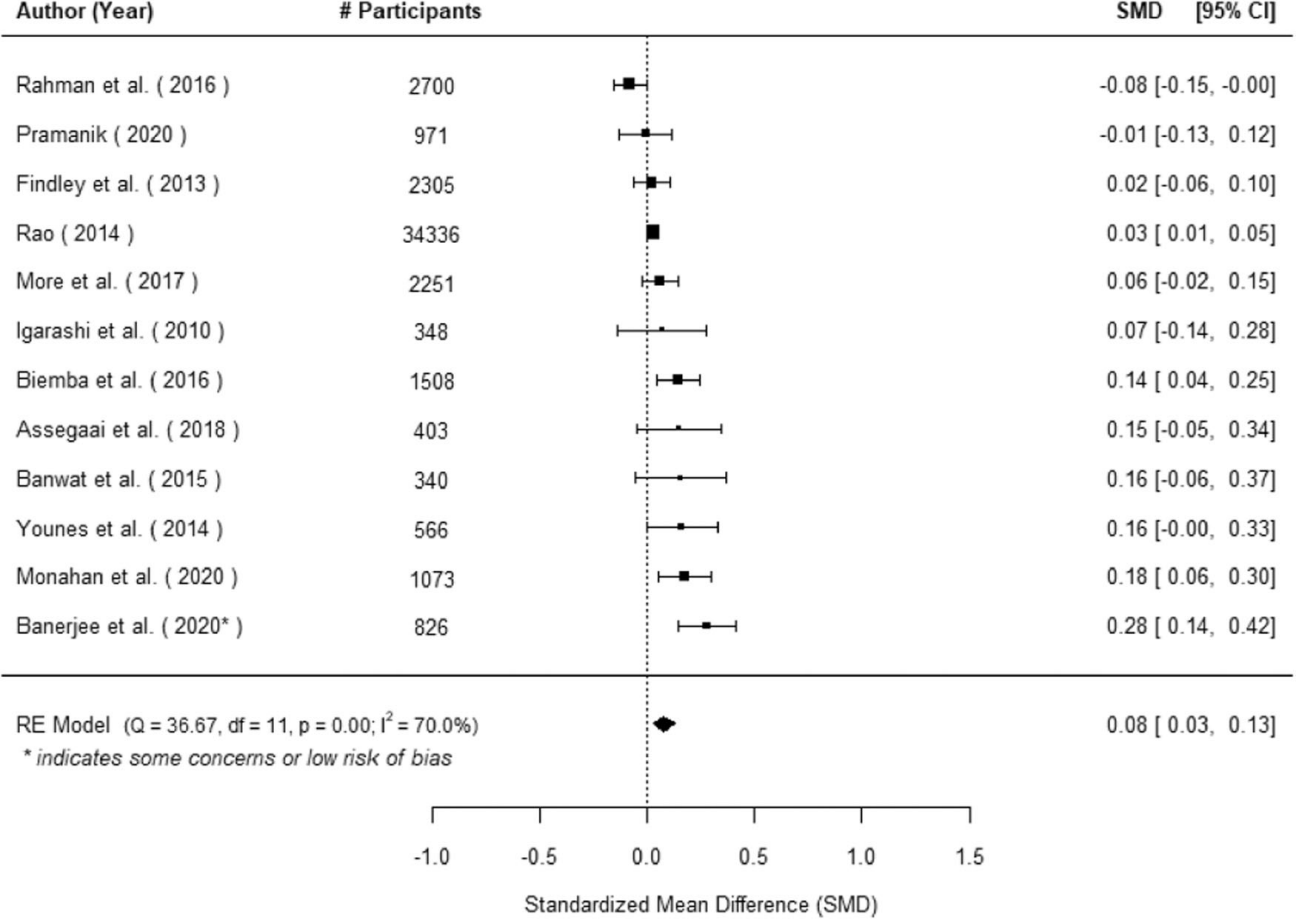
Forest plot showing the observed outcomes and the estimate of the random‐effects model for interventions with community engagement as the intervention on full immunisation.

A total of k=12 examined the effects of interventions with engagement as the intervention on full childhood immunisation. Studies that used engagement as the intervention had a significant effect on full childhood immunisation ($\hat{\mu }=0.08$ [95% CI: 0.03 to 0.13]). Again, the average outcome differed significantly from zero (z=3.02, p=0.003). A forest plot showing the observed outcomes and the estimate based on the random‐effects model is shown in Figure 11.

According to the Q‐test, the true outcomes appear to be heterogeneous (Q(11)=36.67, p<0.01, ${\hat{\tau }}^{2}=0.00$, $I^{2}=70.00$%). An examination of the studentized residuals revealed that none of the studies had a value larger than ±2.87 and hence there was no indication of outliers in the context of this model. Likewise, according to the Cook's distances, none of the studies could be considered to be overly influential.

**Figure 12:**
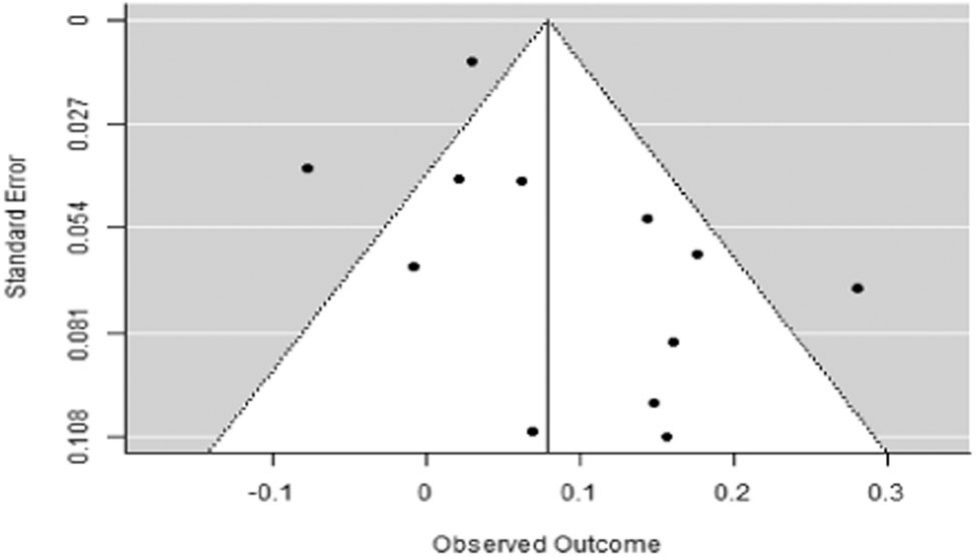
Funnel plot for interventions with community engagement as the intervention on full immunisation.

With only one high or moderate quality study, sensitivity analysis by study quality could not be completed for this body of evidence. A funnel plot of the estimates is shown in Figure 12. Neither the rank correlation nor the regression test indicated any funnel plot asymmetry (p=0.20 and p=0.07, respectively). Exposure to the intervention (in months) was the only significant source of heterogeneity such that for each additional month of intervention exposure, effects decreased by 0.003 standard deviation units ($\hat{B}=-0.003,p=0.049$ [95% CI: −0.01 to −0.00001]).

###### 6.3.1.1.2 Effects of engagement in the intervention design on full childhood immunisation

**Figure 13:**
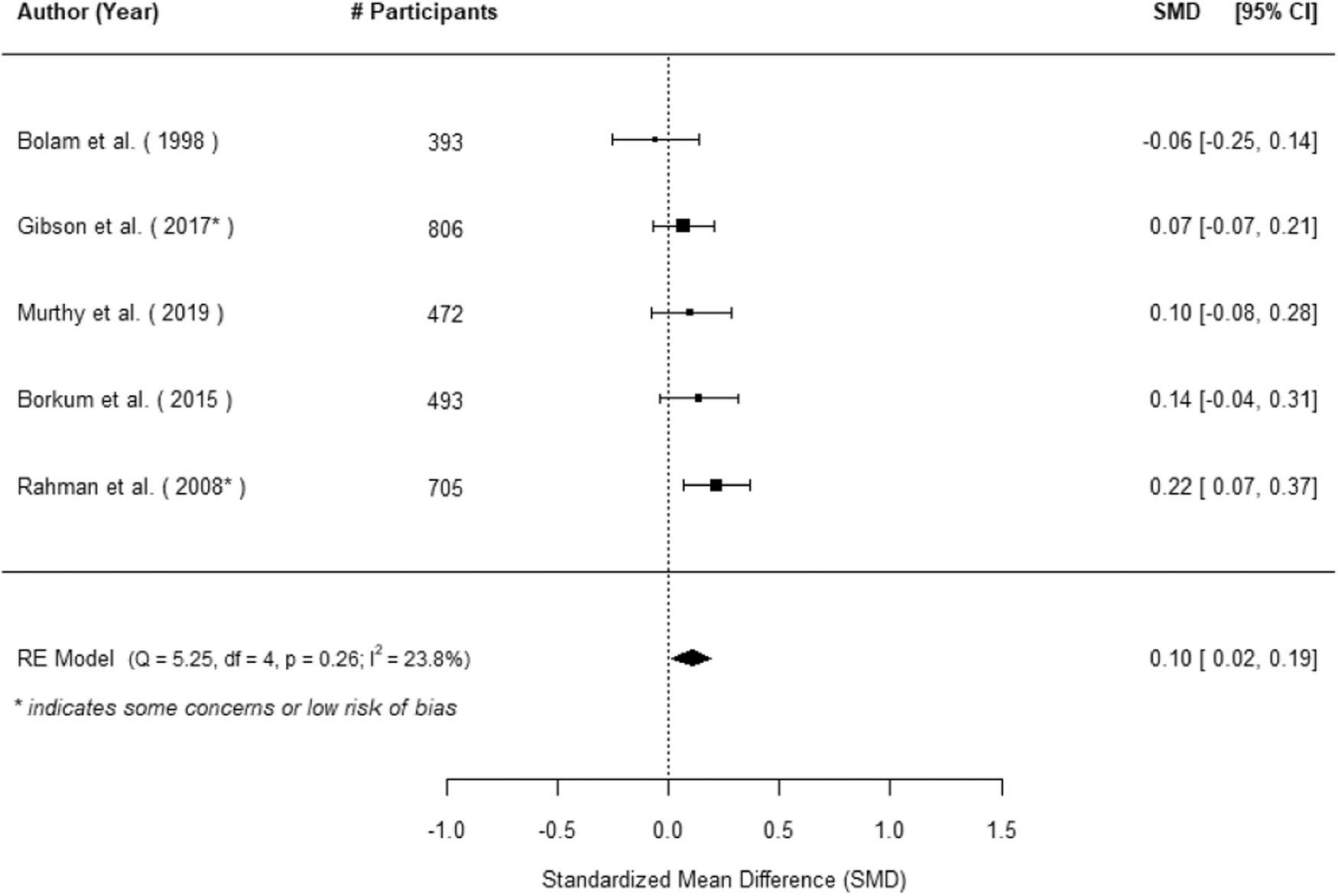
Forest plot showing the observed outcomes and the estimate of the random‐effects model for interventions with community engagement in the design on full immunisation.

A total of k=5 studies were included in the analysis. The observed outcomes ranged from −0.06 to 0.22. The estimated average outcome based on the random‐effects model was $\hat{\mu }=0.10$ [95% CI: 0.02 to 0.19]. Therefore, the average outcome differed significantly from zero (z=2.40, p=0.02). A forest plot showing the observed outcomes and the estimate based on the random‐effects model is shown in Figure 13.

According to the Q‐test, there was no significant amount of heterogeneity in the true outcomes (Q(4)=5.25, p=0.26, ${\hat{\tau }}^{2}=0.00$, $I^{2}=23.83$%). An examination of the studentized residuals revealed that none of the studies had a value larger than ±2.58 and hence there was no indication of outliers in the context of this model. According to the Cook's distances, none of the studies could be considered to be overly influential.

With no heterogeneity among effects, moderator analyses were not appropriate. When low quality studies were removed, two studies remained, and the summary effect increased ($\hat{\mu }=0.14$ [95% CI: −0.01 to 0.29, but the effect was no longer significant (z=1.89, p=0.06).

###### 6.3.1.1.3 Effects of engagement in implementation autonomy on full childhood immunisation

**Figure 14:**
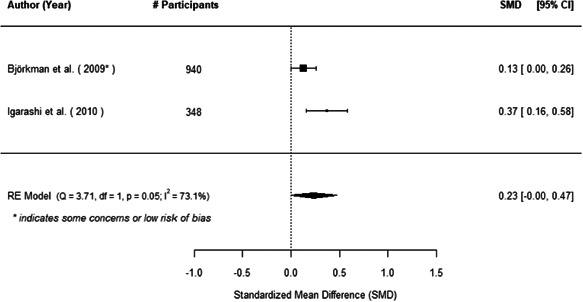
Forest plot showing the observed outcomes and the estimate of the random‐effects model for interventions with community engagement in implementation autonomy on full childhood immunisation.

Only k = 2 studies using engagement in implementation autonomy examined the effect on full childhood immunisation. The estimated average outcome based on the random‐effects model was $\hat{\mu }=0.23$ ([95% CI: −0.001 to 0.47],z=1.95, p=0.051), indicating no effect of these programmes on full immunisation (see Figure 14). According to the Q‐test, the true outcomes appear to be homogeneous (Q(1)=3.71, p=0.054, ${\hat{\tau }}^{2}=0.02$, $I^{2}=73.07$%). There was no indication of outliers in the context of this model, and with only two studies, we could not test for moderation or publication bias.

###### 6.3.1.1.4 Effects of interventions with multiple engagement types on full childhood immunisation

**Figure 15:**
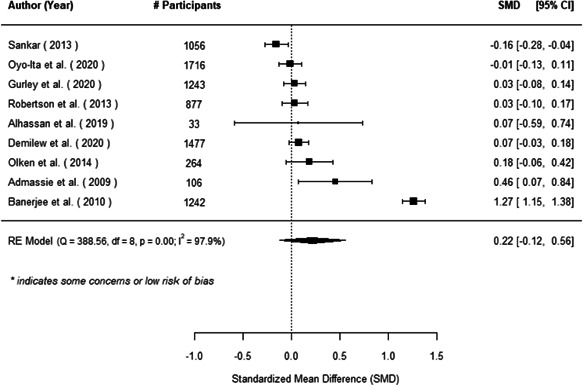
Forest plot showing the observed outcomes and the estimate of the random‐effects model for multiple engagement types on full childhood immunisation.

We included a total of k=10 studies in the analysis. The observed outcomes ranged from −0.12 to 1.27. The estimated average outcome based on the random‐effects model was $\hat{\mu }=0.22\;$(95% CI: −0.12 to 0.56, and the average outcome did not differ significantly from zero (z=1.27, p=0.20). A forest plot showing the observed outcomes and the estimate based on the random‐effects model is shown in Figure 15.

According to the Q‐test, the true outcomes appear to be heterogeneous (Q(8)=388.56, p<0.01, ${\hat{\tau }}^{2}=0.25$, $I^{2}=97.94$%). There was no indication of outliers in the context of this model, and no high or moderate quality studies, so we were unable to perform a sensitivity analysis. None of the moderators were significant sources of heterogeneity in the context of this model, including whether the specific combination of engagement types led to different effects (see Supporting Information: Appendix I Table 3), but for full childhood immunisation we found no differences by engagement packages ($\hat{\beta }=0.36,p=0.30$ [95% CI: −0.31 to 1.02]).

###### 6.3.1.1.5 Full childhood immunisation for girls

**Figure 16:**
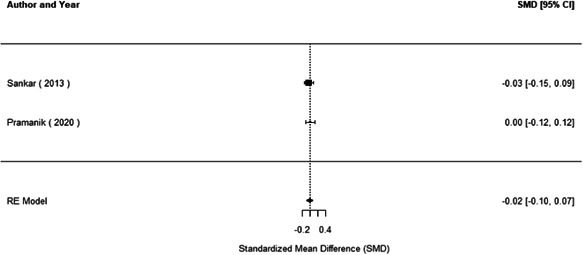
Forest plot showing the observed outcomes and the estimate of the random‐effects model for full childhood immunisation for girls.

Wherever possible, we sought to examine subgroups in cases where results of primary studies were disaggregated by group (e.g., child sex, SES, etc.). For full immunisation, two studies reported disaggregated data on the effect of community engagement interventions on full immunisation for female children, thus we included *k* = 2 studies in the analysis. The estimated average outcome based on the random‐effects model was $\hat{\mu }=-0.02$ (95% CI: −0.10to0.07). Therefore, the average outcome did not differ significantly from zero (z=−0.36, p=0.72). A forest plot showing the observed outcomes and the estimate based on the random‐effects model is shown in Figure 16. Given the small number of studies, this result should be interpreted with caution. With only two studies, moderator analyses were not possible and tests of publication bias are not valid.

##### 6.3.1.2 Partial immunisation

**Figure 17:**
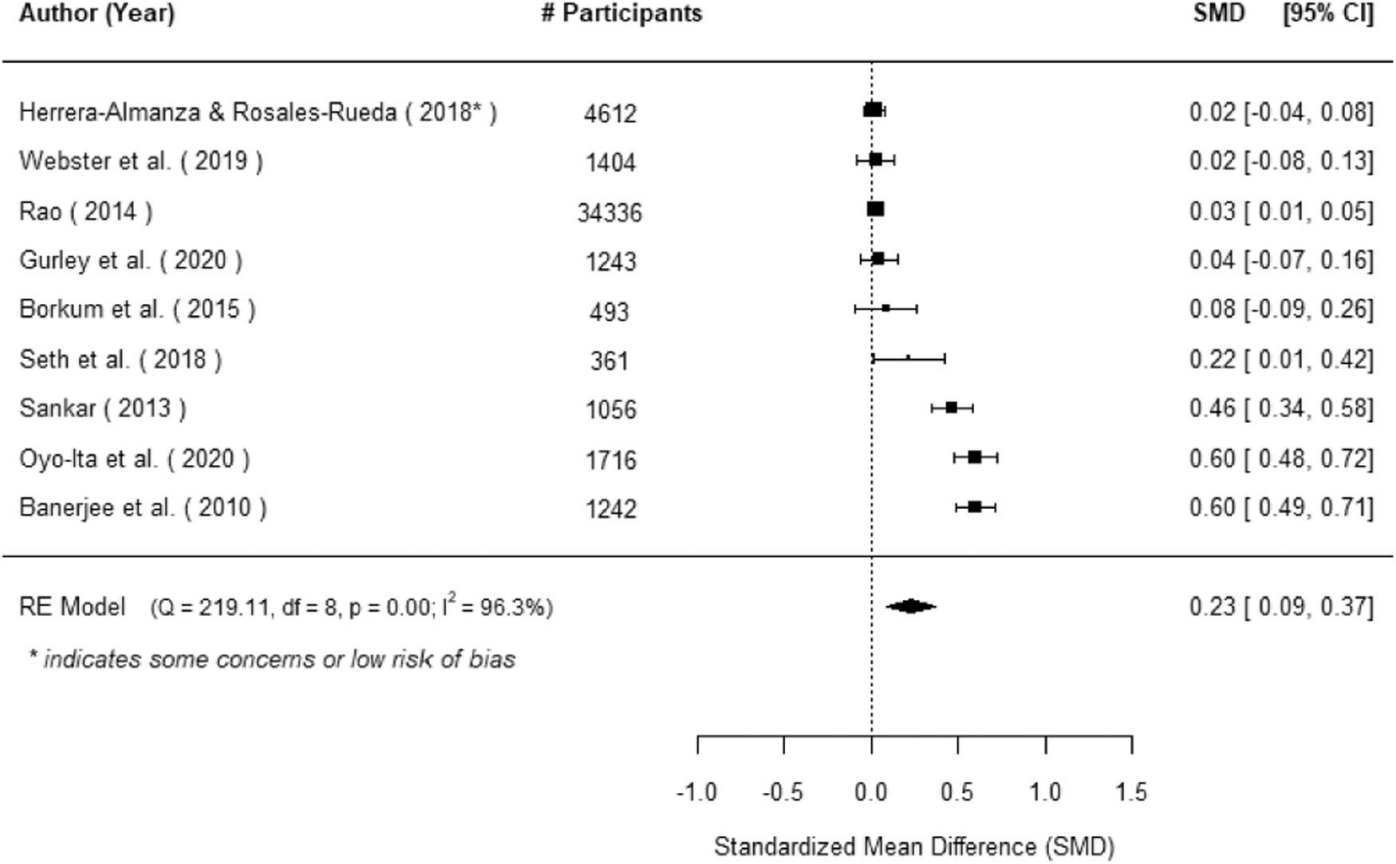
Forest plot showing the observed outcomes and the estimate of the random‐effects model for the impact of community engagement interventions on partial childhood immunisation.

We included a total of k=9 studies the analysis. The estimated average outcome based on the random‐effects model was $\hat{\mu }=0.23$ ([95% CI: 0.09 to 0.37], z=3.15, p=0.002), indicating a benefit for the intervention group compared to the control group (see Figure 17).

According to the Q‐test, the true outcomes appear to be heterogeneous (Q(8)=219.11, p<0.001, ${\hat{\tau }}^{2}=0.04$, $I^{2}=96.351$%). An examination of the studentized residuals revealed that none of the studies had a value larger than ±2.77 and hence there was no indication of outliers in the context of this model. Likewise, according to the Cook's distances, none of the studies could be considered to be overly influential. With eight out of the nine studies being assessed as high risk of bias, we were unable to conduct sensitivity analysis by study quality.

As a robustness check, we used robust variance analysis and included all dependent effects in the analysis, totalling 13 effects from the same 9 studies (*df* = 7.96). The overall average effect was slightly smaller but still significant ($\hat{\mu }=0.21$ [95% CI: 0.03 to 0.38], *p* = 0.03). Sensitivity analyses show the effect to be sensitive to all values of *ρ*.

###### 6.3.1.2.1 Effects of interventions with engagement as the intervention on partial childhood immunisation

**Figure 18:**
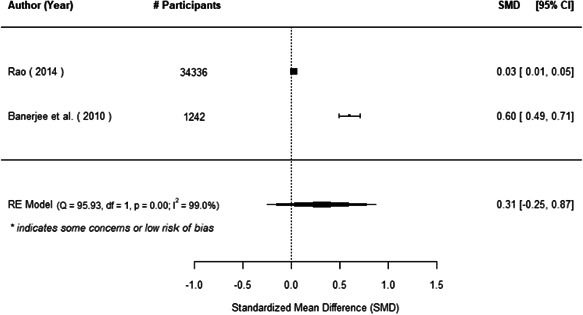
Forest plot showing the observed outcomes and the estimate of the random‐effects model for interventions with community engagement as the intervention on partial immunisation.

Only two studies reporting on partial immunisation used interventions with community engagement as the intervention. The estimated average outcome based on the random‐effects model was $\hat{\mu }=0.31$ ([95% CI: −0.25 to 0.87],z=1.10, p=0.27), indicating no significant difference between the intervention group and the control group on partial immunisation (see Figure 18). Given the small number of studies, this result should be interpreted with caution. According to the Q‐test, the true outcomes appear to be heterogeneous (Q(1)=95.93, p<0.001, ${\hat{\tau }}^{2}=0.16$, $I^{2}=98.96$%). With only two studies, moderator analyses were not appropriate and tests of publication bias are not valid.

###### 6.3.1.2.2 Effects of interventions with engagement in the intervention design on partial childhood immunisation

**Figure 19:**
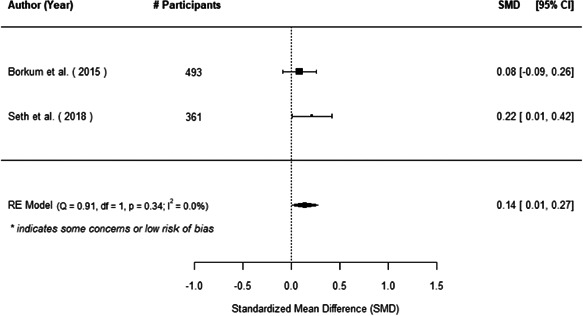
Forest plot showing the observed outcomes and the estimate of the random‐effects model for interventions with community engagement in the design of the intervention on partial childhood immunisation.

Only two studies reporting on partial immunisation used interventions with community engagement in the intervention design. The estimated average outcome based on the random‐effects model was $\hat{\mu }=0.14$ ([95%CI:0.01to0.27],z=2.05, p=0.04), indicating a small but significant benefit to the intervention group compared to the control group (see Figure 19). Given the small number of studies, this result should be interpreted with caution. According to the Q‐test, the true outcomes appear to be homogeneous (Q(1)=0.91, p=0.34, ${\hat{\tau }}^{2}=0.00$, $I^{2}=0.00$%). With only two studies and no heterogeneity, moderator analyses were not appropriate and tests of publication bias are not valid.

###### 6.3.1.2.3 Effects of interventions with engagement in implementation autonomy on partial childhood immunisation

Only one study reporting on partial immunisation utilised an intervention which used engagement in the implementation (Webster, 2019), so a quantitative synthesis was not possible. This cluster RCT implemented in Uganda found a null effect of their programme on partial childhood immunisation (*g* = 0.02 [95% CI: −0.08 to 0.13]), but like most studies, it was assessed as having a high risk of bias.

###### 6.3.1.2.4 Effects of interventions with multiple engagement types on partial childhood immunisation

**Figure 20:**
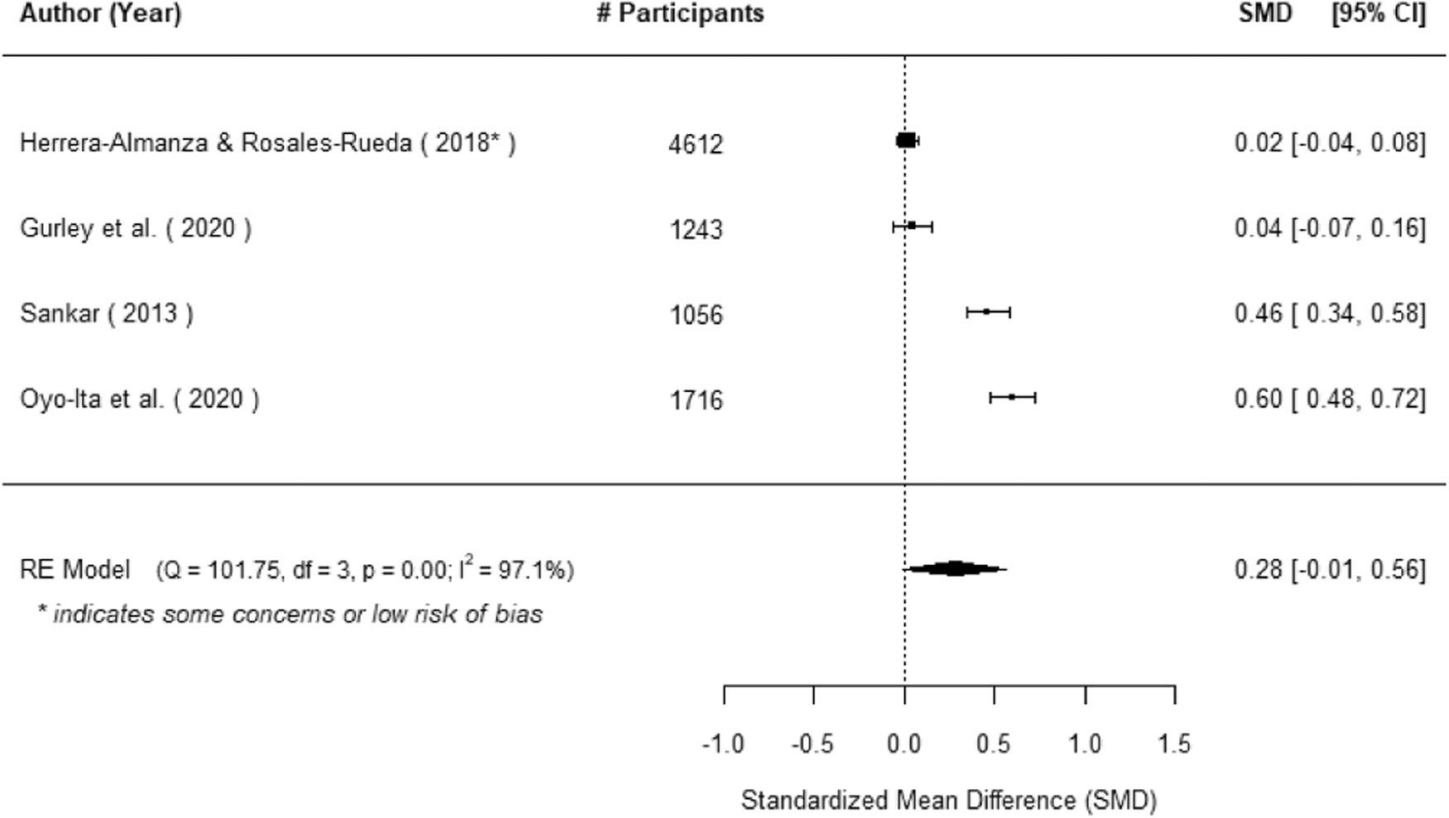
Forest plot showing the observed outcomes and the estimate of the random‐effects model for interventions with multiple engagement types on partial immunisation.

We included a total of k=4 studies in the analysis. The estimated average outcome based on the random‐effects model was $\hat{\mu }=0.28$ (95% CI: −0.01 to 0.56). Therefore, the average outcome did not differ significantly from zero (z=1.91, p=0.06), indicating no difference between the intervention and control groups (see Figure 20). According to the Q‐test, the true outcomes appear to be heterogeneous (Q(3)=101.75, p<0.01, ${\hat{\tau }}^{2}=0.08$, $I^{2}=97.05$%).

An examination of the studentized residuals revealed that none of the studies had a value larger than ±2.50 and hence there was no indication of outliers in the context of this model. According to the Cook's distances, none of the studies could be considered to be overly influential. Of the moderators we were able to test, none were significant sources of heterogeneity (see Supporting Information: Appendix I Table 3). We were unable to test for differences among engagement packages because only one study used a combination of engagement in the design and engagement as the intervention (Gurley et al., 2020) while the remaining studies used a combination of engagement in implementation autonomy and engagement as the intervention.

##### 6.3.1.3 Measles vaccination

**Figure 21:**
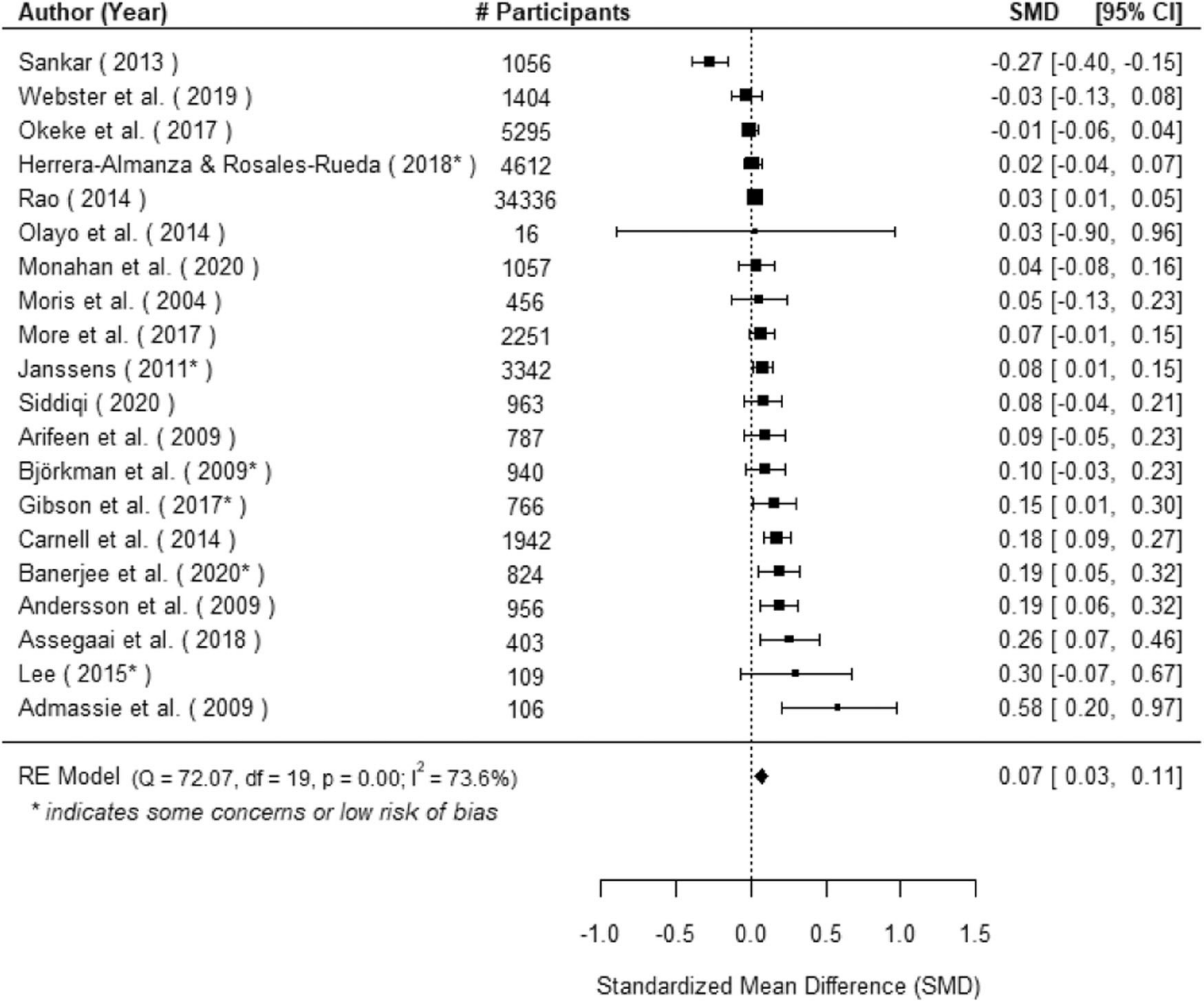
Forest plot showing the observed outcomes and the estimate of the random‐effects model for the impact of community engagement interventions on measles vaccination.

**Figure 22:**
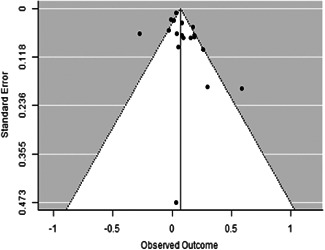
Funnel plot for studies examining the effect of community engagement interventions on measles vaccination.

A total of k=20 studies examined the relationship between community engagement interventions and measles vaccination. The estimated average outcome was $\hat{\mu }=0.07$ ([95% CI: 0.03 to 0.11], z=3.21, p<0.01) indicating a very small but significant benefit for the treated group compared to the untreated group (see Figure 21). The true outcomes appear to be heterogeneous (Q(19)=72.07, p<0.01, ${\hat{\tau }}^{2}=0.01$, $I^{2}=73.64$%). An examination of the studentized residuals revealed that one study (Sankar, 2013) had a value larger than ±3.02 and may be a potential outlier in the context of this model. Indeed, sensitivity analysis leaving out Sankar (2013) would result in an increase in the average effect ($\hat{\mu }=0.08$ [95% CI: 0.05 to 0.12]), and it was still statistically significant (z=4.44, p<0.001). According to the Cook's distances, none of the studies could be considered to be overly influential. A funnel plot of the estimates is shown in Figure 22. Both the rank correlation and the regression test indicated potential funnel plot asymmetry (p=0.05 and p=0.02, respectively).

**Figure 23:**
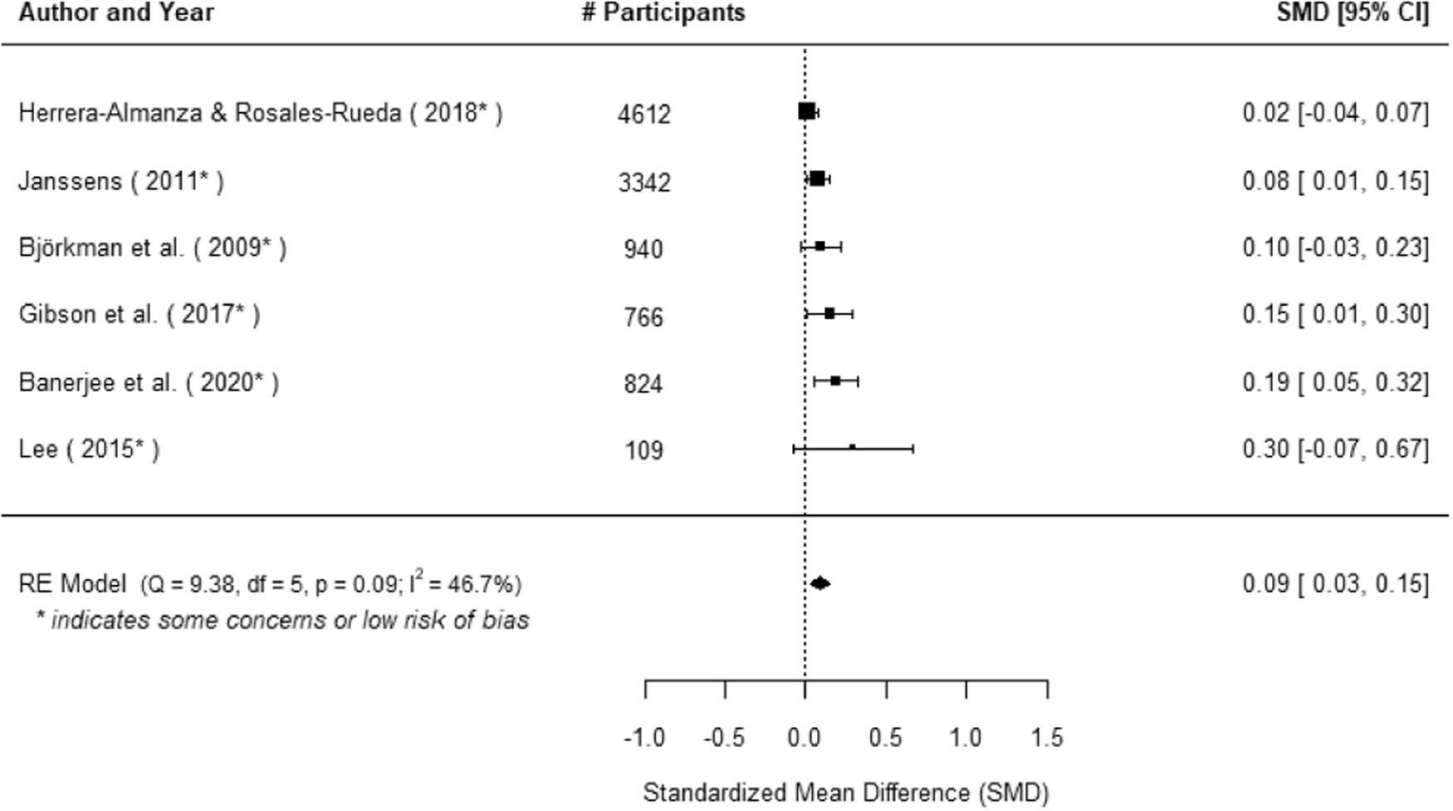
Forest plot showing the observed outcomes and the estimate of the random‐effects model for the impact of community engagement interventions on measles vaccination when low quality studies are removed.

When low quality studies are removed, the average effect increases ($\hat{\mu }=0.09$, k = 6, [95% CI: 0.03 to 0.15]) and is still statistically significant (z=2.98, p=0.003; Figure 23).

As a robustness check, we used robust variance analysis and included all dependent effects in the analysis, totalling 32 effects from the same 20 studies (*df* = 15.5). The overall average effect was slightly smaller but still significant ($\hat{\mu }=0.06$ [95% CI: 0.01 to 0.11], *p* = 0.03). Sensitivity analyses show the effect to be sensitive to all values of *ρ*.

###### 6.3.1.3.1 Effects of engagement as the intervention on measles vaccination

**Figure 24:**
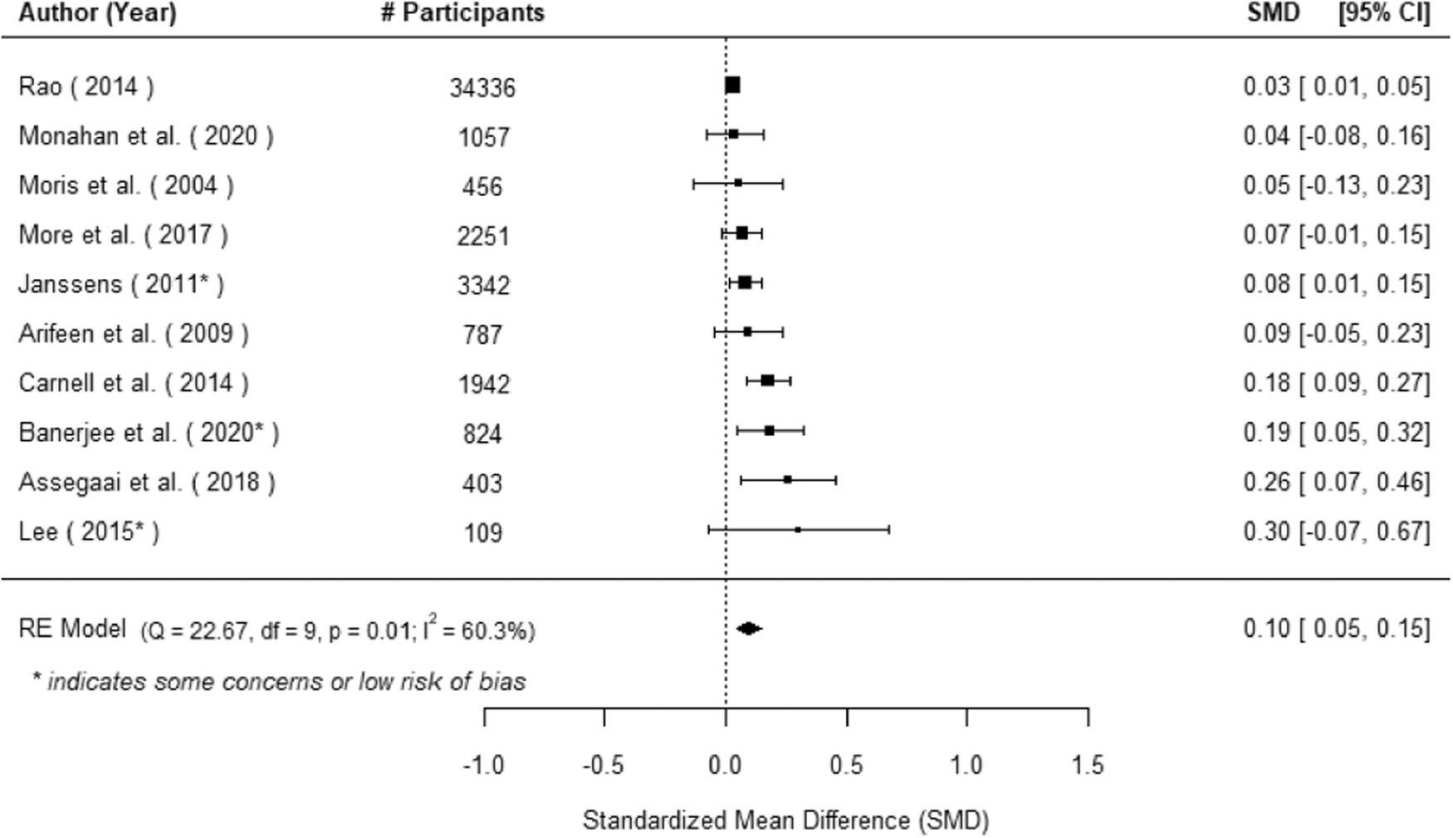
Forest plot showing the observed outcomes and the estimate of the random‐effects model of community engagement as the intervention on measles.

We included a total of k=10 studies in the analysis. The observed outcomes ranged from 0.03 to 0.30. The estimated average outcome based on the random‐effects model was $\hat{\mu }=0.10$ (95% CI: 0.05 to 0.15). Therefore, the average outcome differed significantly from zero (z=3.89, p<0.001). A forest plot showing the observed outcomes and the estimate based on the random‐effects model is shown in Figure 24.

According to the Q‐test, the true outcomes appear to be heterogeneous (Q(9)=22.67, p=0.007, ${\hat{\tau }}^{2}=0.003$, $I^{2}=60.29$%). An examination of the studentized residuals revealed that none of the studies had a value larger than ±2.81 and hence there was no indication of outliers in the context of this model. According to the Cook's distances, none of the studies could be considered to be overly influential.

**Figure 25:**
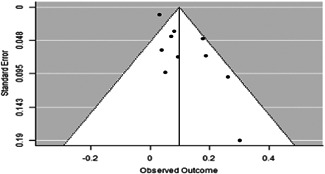
Funnel plot showing studies of community engagement as the intervention on measles.

A funnel plot of the estimates is shown in Figure 25. The regression test indicated funnel plot asymmetry (p<0.01) but not the rank correlation test (p=0.38). None of the moderators we tested were significant sources of heterogeneity (see Table 3, Supporting Information: Appendix I).

###### 6.3.1.3.2 Effects of engagement in the intervention design on measles vaccination

**Figure 26:**
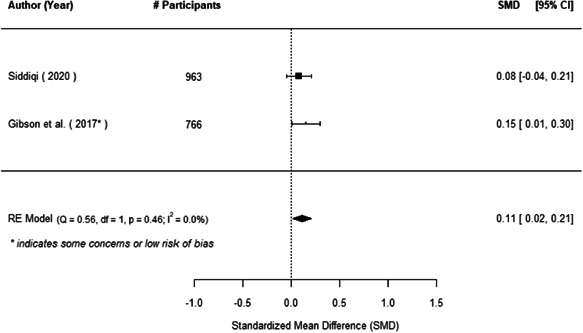
Forest plot showing the observed outcomes and the estimate of the random‐effects model for interventions with community engagement in the design on measles vaccination.

Only two studies on measles vaccination used interventions with engagement in the design. The estimated average outcome based on the random‐effects model was $\hat{\mu }=0.11$ (95% CI: 0.02 to 0.21), indicating a small but significant benefit to the intervention participants compared to the control group (z=2.36, p=0.02). A forest plot showing the observed outcomes and the estimate based on the random‐effects model is shown in Figure 26. Given the small number of studies, this result should be interpreted with caution. According to the Q‐test, there was no significant amount of heterogeneity in the true outcomes (Q(1)=0.56, p=0.46, ${\hat{\tau }}^{2}=0.00$, $I^{2}=0.00$%). With only two studies and no heterogeneity among effects, moderator analyses and tests of publication bias were not appropriate.

###### 6.3.1.3.3 Effects intervention with engagement in implementation autonomy on measles vaccination

**Figure 27:**
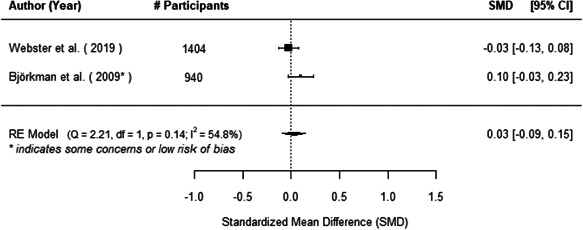
Forest plot showing the observed outcomes and the estimate of the random‐effects model for interventions with community engagement in implementation autonomy on measles vaccination.

Only two studies on measles vaccination used interventions with engagement in implementation autonomy. The estimated average outcome based on the random‐effects model was $\hat{\mu }=0.03$ (95% CI: −0.09 to 0.15). Therefore, the average outcome did not differ significantly from zero (z=0.47, p=0.64). A forest plot showing the observed outcomes and the estimate based on the random‐effects model is shown in Figure 27. Given the small number of studies, this result should be interpreted with caution. According to the Q‐test, the true outcomes appear to be homogeneous (Q(1)=2.21, p=0.14, ${\hat{\tau }}^{2}=0.004$, $I^{2}=54.84$%; see Figure 27). With only two studies we were unable to test for publication bias or sources of heterogeneity.

###### 6.3.1.3.4 Effects of interventions with multiple engagement types on measles vaccination

**Figure 28:**
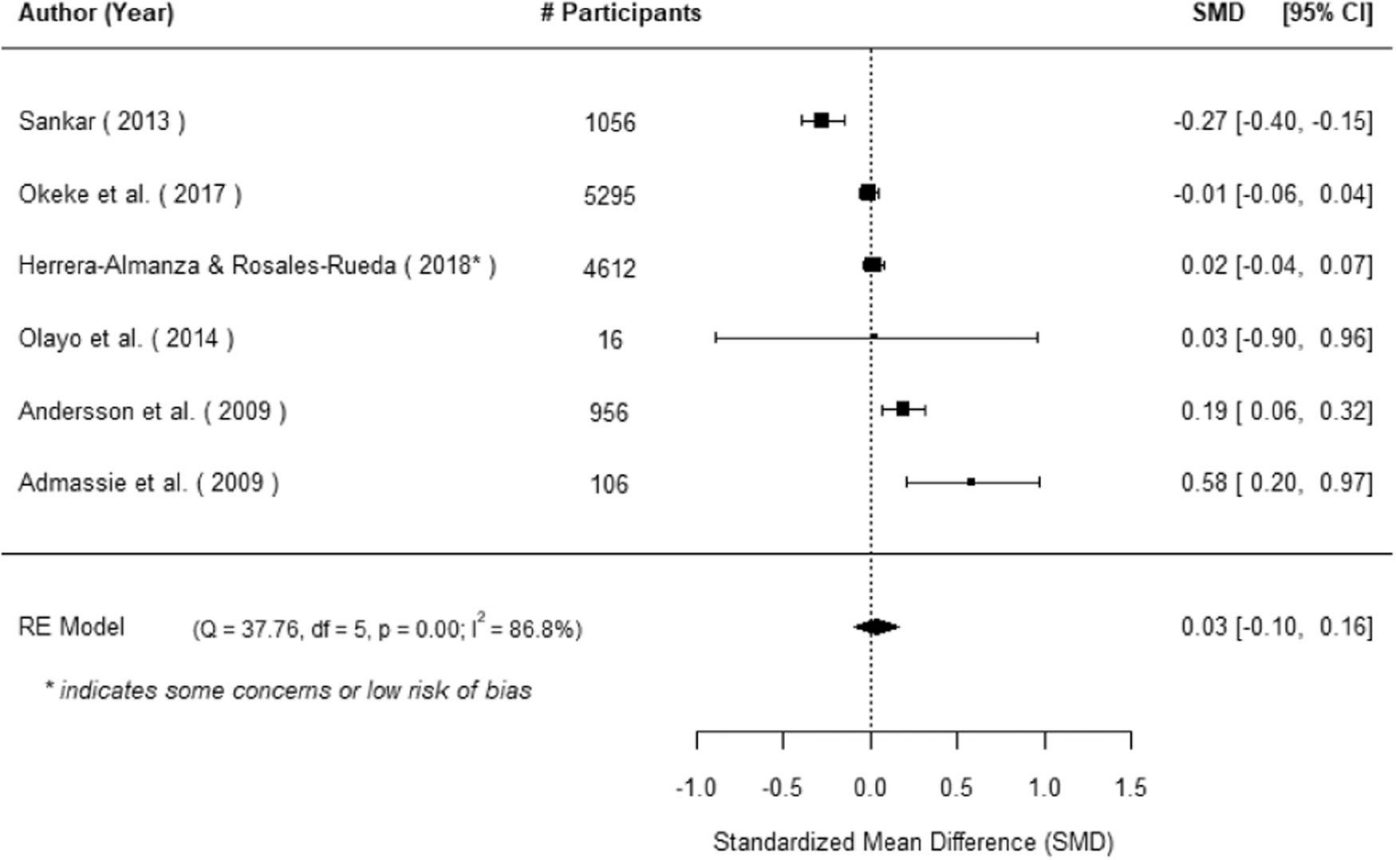
Forest plot showing the observed outcomes and the estimate of the random‐effects model.

A total of k=6 studies were included in the analysis. The observed outcomes ranged from −0.27 to 0.58. The estimated average outcome based on the random‐effects model was $\hat{\mu }=0.03$ (95% CI: −0.10 to 0.16). Therefore, the average outcome did not differ significantly from zero (z=0.44, p=0.20). A forest plot showing the observed outcomes and the estimate based on the random‐effects model is shown in Figure 28.

According to the Q‐test, the true outcomes appear to be heterogeneous (Q(5)=37.76, p<0.01, ${\hat{\tau }}^{2}=0.02$, $I^{2}=86.76$%; see Figure 28). An examination of the studentized residuals revealed that one study (Sankar, 2013) had a value larger than ±2.64 and may be a potential outlier in the context of this model. According to the Cook's distances, none of the studies could be considered to be overly influential. Indeed, sensitivity analyses leaving each study out indicated that removing Sankar (2013) would increase the overall average effect ($\hat{\mu }=0.09$ [95% CI: −0.02 to 0.19]), but the effect would still be nonsignificant (z=1.58, p=0.11). With only one high or medium quality study, we were unable to conduct sensitivity analysis by study quality. None of the moderators that could be tested were significant in the context of this model (see Supporting Information: Appendix I Table 3). For measles vaccinations, we were unable to test whether different combinations of community engagement produced different results because all but one study used a combination of engagement in the implementation and engagement as the intervention.

##### 6.3.1.4 BCG vaccination

**Figure 29:**
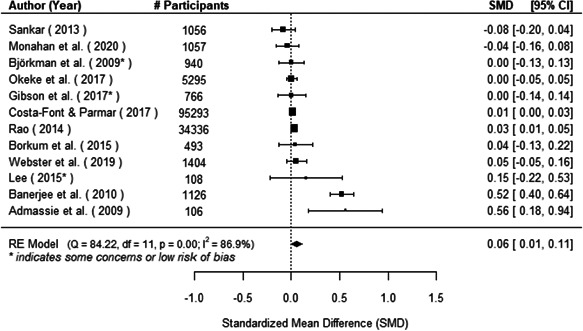
Forest plot showing the observed outcomes and the estimate of the random‐effects model for the impact of community engagement interventions on BCG vaccination.

We included a total of k=12 studies in the analysis. The estimated average outcome based on the random‐effects model was $\hat{\mu }=0.06$ ([95% CI: 0.01 to 0.11], z=2.28, p=0.02), indicating a very small but significant benefit to the intervention participants compared to the control participants (see Figure 29). According to the Q‐test, the true outcomes appear to be heterogeneous (Q(11)=84.22, p<0.01, ${\hat{\tau }}^{2}=0.00$, $I^{2}=86.94$%). An examination of the studentized residuals revealed that one study (Banerjee, 2010) had a value larger than ±2.87 and may be a potential outlier in the context of this model. According to the Cook's distances, Banerjee (2010) could also be considered to be overly influential. Sensitivity analyses leaving each study out indicated that removing Banerjee (2010) would reduce the overall average effect ($\hat{\mu }=0.02$ (95% CI: −0.005 to 0.04), and the resulting effect would be nonsignificant (z=1.51, p=0.13). Moderator analysis revealed that publication year was a significant predictor such that more recent studies find smaller effects that older studies, with each additional year reducing the size of the effect by 0.03 standard deviation units (β=−0.03 [95% CI: −0.05 to −0.001], p = 0.04). No other moderators were significant (see Supporting Information: Appendix I Table 3). Only three studies were high or moderate quality. When those three studies were synthesised, the resulting effect was reduced $(\hat{\mu }=0.01$ [95% CI: −0.08 to 0.10]) and no longer significant (z=0.22, p=0.82).

**Figure 30:**
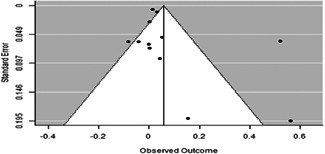
Funnel plot for the impact of community engagement interventions on BCG vaccination.

A funnel plot of the estimates is shown in Figure 30. The regression test indicated funnel plot asymmetry (p=0.04) but not the rank correlation test (p=0.20).

As a robustness check, we used robust variance analysis and included all dependent effects in the analysis, totalling 16 effects from the same 12 studies (*df* = 7.4). The overall average effect was slightly smaller and nonsignificant ($\hat{\mu }=0.04$ [95% CI: −0.02 to 0.10], *p* = .20). Sensitivity analyses show the effect to be sensitive to all values of *ρ*.

###### 6.3.1.4.1 Effects of engagement as the intervention on BCG vaccination

**Figure 31:**
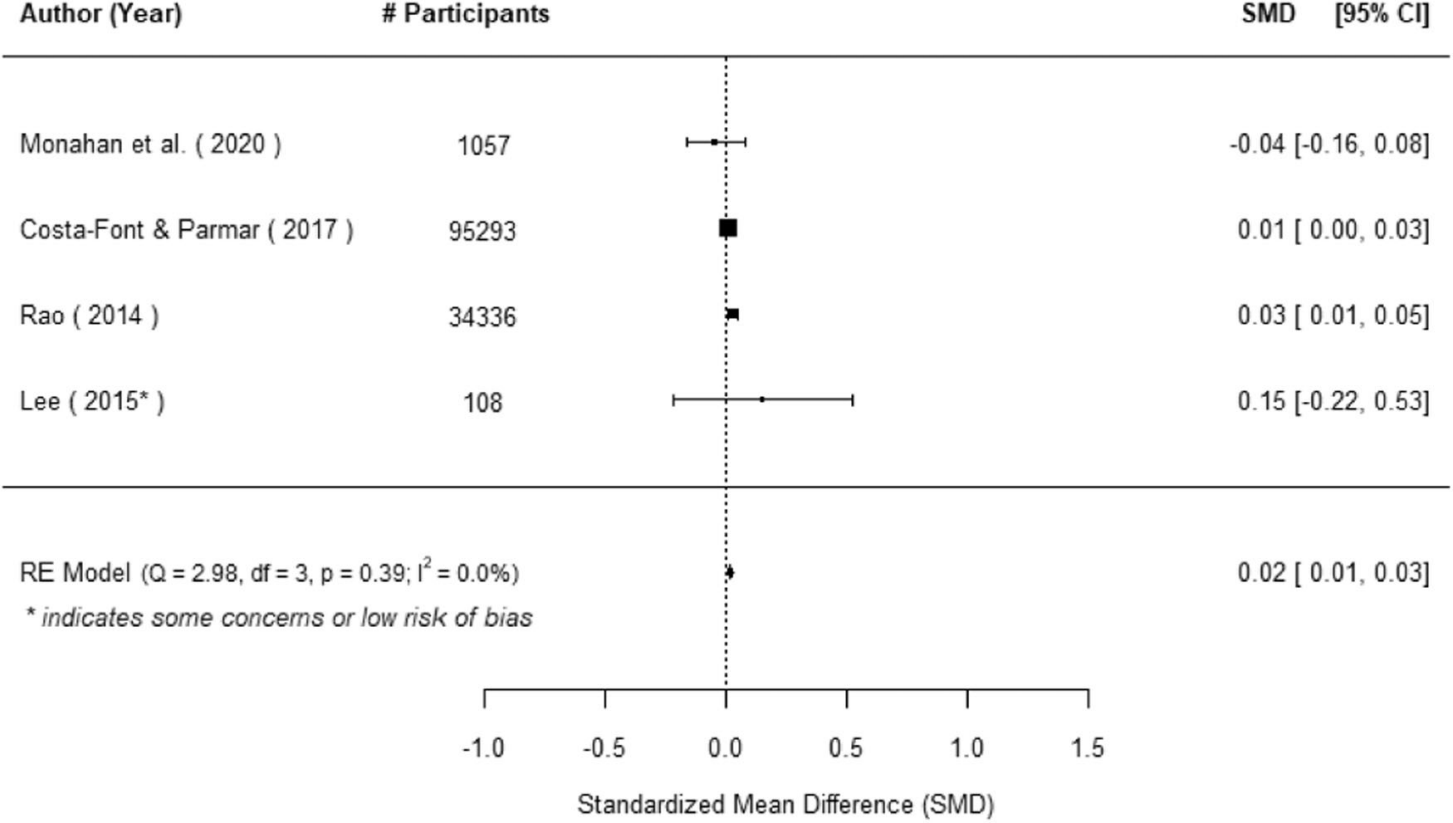
Forest plot showing the observed outcomes and the estimate of the random‐effects model for interventions with community engagement as the intervention on BCG vaccination.

We included a total of k=4 studies the analysis. The estimated average outcome based on the random‐effects model was $\hat{\mu }=0.02$ (95% CI: 0.01 to 0.03). Therefore, the average outcome differed significantly from zero (z=3.32, p<0.01), indicating a very small but significant benefit to the treated group compared to the control group (see Figure 31). According to the Q‐test, there was no significant amount of heterogeneity in the true outcomes (Q(3)=2.98, p=0.39, ${\hat{\tau }}^{2}=0.00$, $I^{2}=0.00$%), thus we did not examine potential sources of heterogeneity for this model. With three of the four studies assessed as high risk of bias, we were unable to conduct a sensitivity analysis by study quality. An examination of the studentized residuals revealed that none of the studies had a value larger than ±2.50 and hence there was no indication of outliers in the context of this model. According to the Cook's distances, none of the studies could be considered to be overly influential.

###### 6.3.1.4.2 Effects of engagement in the intervention design on BCG vaccination

**Figure 32:**
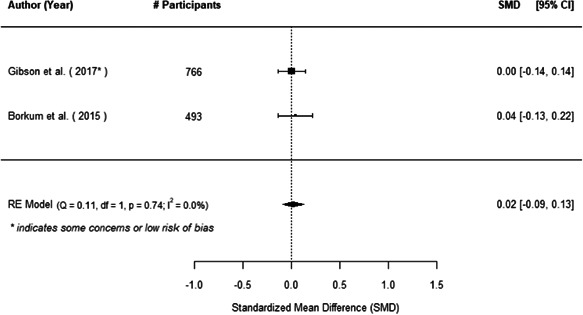
Forest plot showing the observed outcomes and the estimate of the random‐effects model for interventions with community engagement in the design of the intervention on BCG vaccination

Only two studies reporting on BCG vaccination used interventions with community engagement in the intervention design. The estimated average outcome based on the random‐effects model was $\hat{\mu }=0.02$ ([95% CI: −0.09 to 0.13],z=0.32, p=0.75), indicating no difference between the intervention group and the control group (see Figure 32). Given the small number of studies, this result should be interpreted with caution. According to the Q‐test, the true outcomes appear to be homogeneous (Q(1)=0.11, p=0.75, ${\hat{\tau }}^{2}=0.00$, $I^{2}=0.00$%). With only two studies and no heterogeneity, moderator analyses were not appropriate and tests of publication bias are not valid.

###### 6.3.1.4.3 Effects of intervention with engagement in implementation autonomy on BCG vaccination

**Figure 33:**
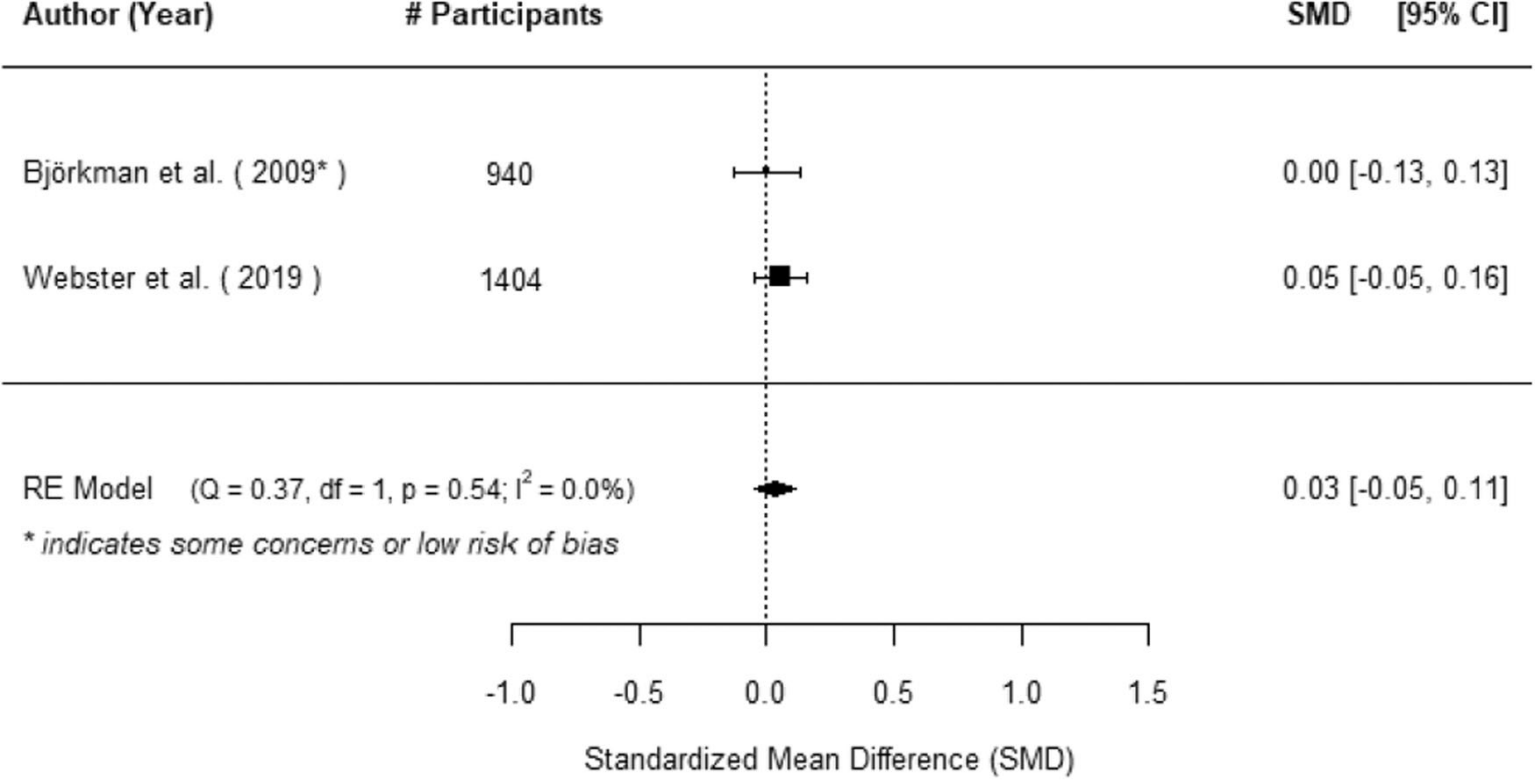
Forest plot showing the observed outcomes and the estimate of the random‐effects model for interventions with community engagement in implementation autonomy on BCG vaccination. BCG, Bacillus Calmette‐Guerin.

Only two studies reporting on BCG vaccination used interventions with community engagement in implementation autonomy. The estimated average outcome was $\hat{\mu }=0.03$ ([95% CI: −0.05 to 0.11], z=0.75, p=0.46) indicating no difference between the treated group and the untreated group (Figure 33). According to the Q‐test, the true outcomes appear to be homogeneous (Q(1)=0.37, p=0.54, ${\hat{\tau }}^{2}=0.00$, $I^{2}=00.00$%). With only two studies and no heterogeneity, moderator analyses were not appropriate and tests of publication bias are not valid.

###### 6.3.1.4.4 Effects of interventions with multiple engagement types on BCG vaccination

**Figure 34:**
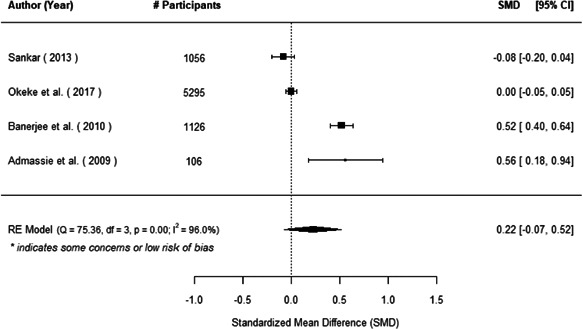
Forest plot showing the observed outcomes and the estimate of the random‐effects model for interventions with multiple engagement types on BCG vaccination. BCG, Bacillus Calmette‐Guerin.

A total of k=4 studies were included in the analysis. The observed outcomes ranged from −0.08 to 0.56. The estimated average outcome based on the random‐effects model was $\hat{\mu }=0.22$ (95% CI: −0.07 to 0.52). Therefore, the average outcome did not differ significantly from zero (z=1.48, p=0.14). A forest plot showing the observed outcomes and the estimate based on the random‐effects model is shown in Figure 34.

According to the Q‐test, the true outcomes appear to be heterogeneous (Q(3)=75.36, p<0.001, ${\hat{\tau }}^{2}=0.08$, $I^{2}=96.01$%). An examination of the studentized residuals revealed that one study (Banerjee, 2010) had a value larger than ±2.50 and may be a potential outlier in the context of this model. According to the Cook's distances, none of the studies could be considered to be overly influential. Indeed, sensitivity analyses leaving each study out indicated that removing Banerjee (2010) would reduce the overall average effect ($\hat{\mu }=0.05$ (95% CI: −0.13 to 0.23), but the effect is still positive and nonsignificant (z=0.56, p=0.58).

We tested all moderators and only post‐intervention versus change from baseline was a significant predictor of BCG vaccination, such that studies examining change from baseline had higher effects than studies examining post‐intervention changes by 0.55 standard deviation units ($\hat{\mu }=-0.55$ (95% CI: −0.69 to −0.40,p<0.001).

##### 6.3.1.5 DPT 1 vaccination

**Figure 35:**
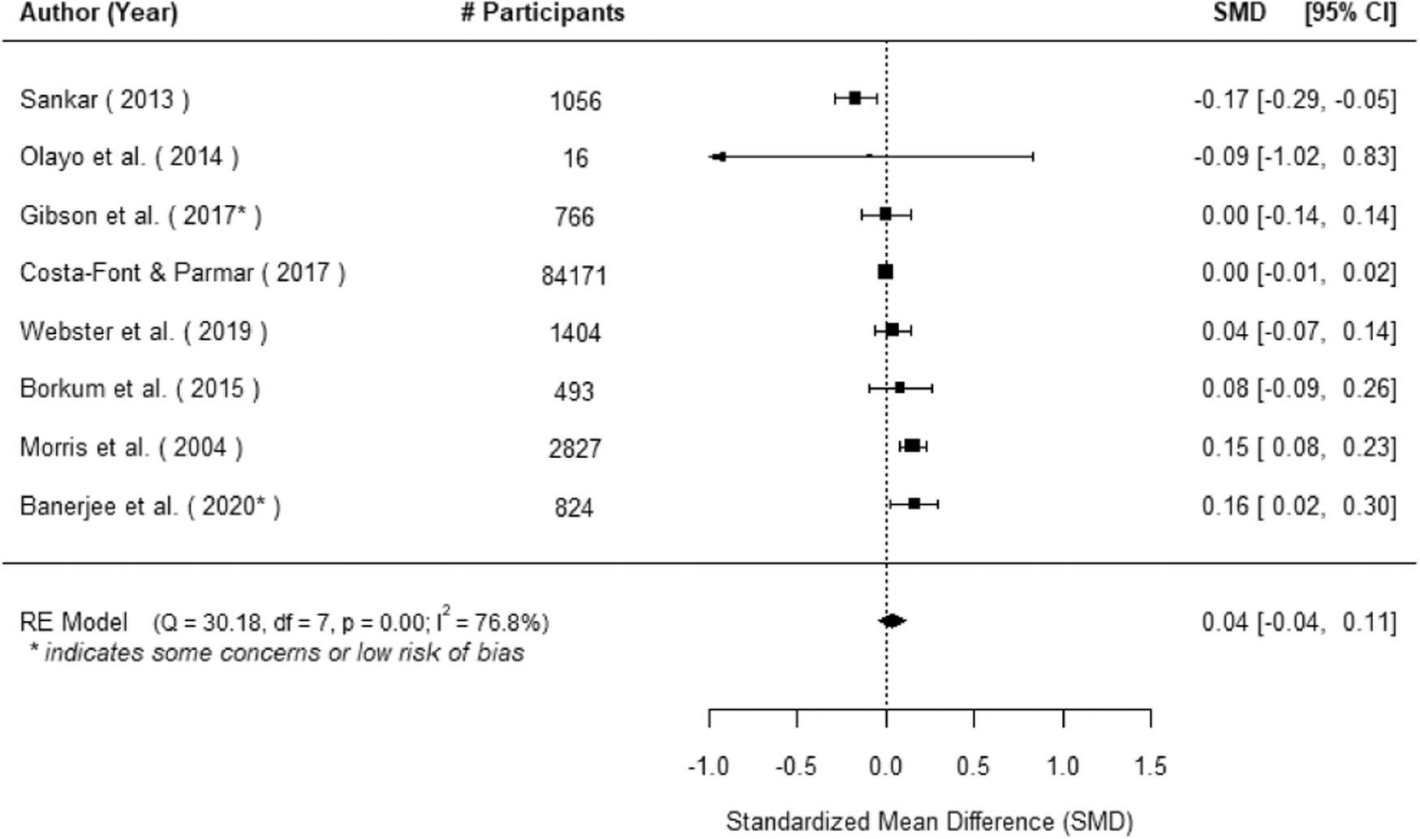
Forest plot showing the observed outcomes and the estimate of the random‐effects model for the impact of community engagement interventions on DPT1 vaccination. DPT, diphtheria, pertussis tetanus.

We included a total of k=8 studies in the analysis. The estimated average outcome based on the random‐effects model was $\hat{\mu }=0.04$ ([95% CI: −0.04 to 0.11],z=0.99, p=0.32), indicating no difference between the intervention group and the control group (see Figure 35). According to the Q‐test, the true outcomes appear to be heterogeneous (Q(7)=30.18, p<0.01, ${\hat{\tau }}^{2}=0.01$, $I^{2}=76.81$%). An examination of the studentized residuals revealed that none of the studies had a value larger than ±2.73 and hence there was no indication of outliers in the context of this model. According to the Cook's distances, none of the studies could be considered to be overly influential. When we removed studies assessed as high risk of bias, two studies remained, and the average effect increased slightly ($\hat{\mu }=0.04$ ([95% CI: −0.04 to 0.11]), but was still nonsignificant (z=0.99, p=0.32). Again, with only two studies contributing effects, this must be interpreted with caution.

We tested for potential sources of heterogeneity and found that study design was a significant predictor of the effect such that studies using quasi‐experimental designs had smaller effects than RCTs by 0.15 standard deviation units (β=−0.15 [95% CI: −0.29 to −0.02], p=0 0.03). We also found that there was a significant difference in the size of effects between programmes implemented by government agencies and those that were not, such that programmes implemented by government agencies (either alone or in tandem with another agency) had larger effects than programmes not implemented by a government agency by 0.17 standard deviation units (β=0.17 [95% CI: 0.01 to 0.34], p = 0.04). No other moderators were significant (see Supporting Information: Appendix I Table 3).

As a robustness check, we used robust variance analysis and included all dependent effects in the analysis, totalling 21 effects from the same 8 studies (*df* = 5.24). The overall average effect was smaller and still nonsignificant ($\hat{\mu }=0.01$ [95% CI: −0.06 to 0.09], *p* = 0.66). Sensitivity analyses show the effect to be sensitive to all values of *ρ*.

###### 6.3.1.5.1 Effects of engagement as the intervention on DPT 1 vaccination

**Figure 36:**
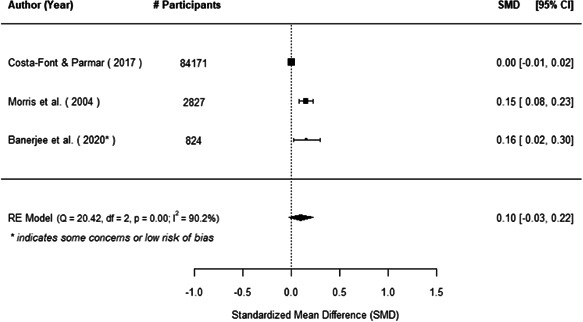
Forest plot showing the observed outcomes and the estimate of the random‐effects model for interventions with community engagement as the intervention on DPT1 vaccination. DPT, diphtheria, pertussis tetanus.

Only k = 3 studies on DPT1 vaccination used interventions with engagement as the intervention. The estimated average outcome based on the random‐effects model was $\hat{\mu }=0.10$ ([95% CI: −0.03 to 0.22],z=1.53, p=0.13), indicating no difference between the treatment and control groups (see Figure 36). According to the Q‐test, the true outcomes appear to be heterogeneous (Q(2)=20.42, p<0.01, ${\hat{\tau }}^{2}=0.01$, $I^{2}=90.21$%). An examination of the studentized residuals revealed that one study (Costa‐Font & Parmar, 2017) had a value larger than ±2.39 and may be a potential outlier in the context of this model. Indeed, sensitivity analyses leaving each study out indicated that removing Costa‐Font and Parmar (2017) would increase the overall average effect ($\hat{\mu }$ = 0.16 (95% CI: 0.09 to 0.22), and the resulting effect would be positive and significant (z = 4.72, p < 0.001). According to the Cook's distances, none of the studies could be considered to be overly influential. With only three studies, we were unable to conduct moderator analyses or test for publication bias. Only one study (Banerjee et al., 2020) was not assessed as high risk of bias.

###### 6.3.1.5.2 Effects of engagement in the intervention design on DPT 1 vaccination

**Figure 37:**
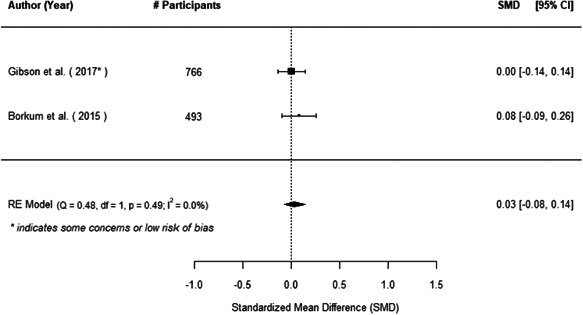
Forest plot showing the observed outcomes and the estimate of the random‐effects model for interventions with community engagement in the design on the intervention on DPT1 vaccination. DPT, diphtheria, pertussis tetanus.

Only two studies on DPT1 vaccination used interventions with engagement in the design. The estimated average outcome based on the random‐effects model was $\hat{\mu }=0.03$ (95% CI: −0.08 to 0.14). Therefore, the average outcome did not differ significantly from zero (z=0.60, p=0.55, see Figure 37), indicating no difference between the treatment group and the control group on DPT1 vaccination. Given the small number of studies, this result should be interpreted with caution. According to the Q‐test, the true outcomes appear to be homogeneous (Q(1)=0.48, p=0.49, ${\hat{\tau }}^{2}=0.00$, $I^{2}=0.00$%). With only two studies, moderator analyses were not possible and test of publication bias are not valid.

###### 6.3.1.5.3 Effects of intervention with engagement in implementation autonomy on DPT 1 vaccination

Only one study reporting on DPT1 vaccination used interventions with engagement in the implementation (Webster, 2019), thus we were unable to perform a statistical synthesis. This cluster RCT from Uganda found a null effect of their programme on DPT1 vaccination (*g* = 0.04 [95% CI: −0.07 to 0.14]), but like most studies, it was assessed as having a high risk of bias.

###### 6.3.1.5.4 Effects of interventions with multiple engagement types on DPT 1 vaccination

**Figure 38:**
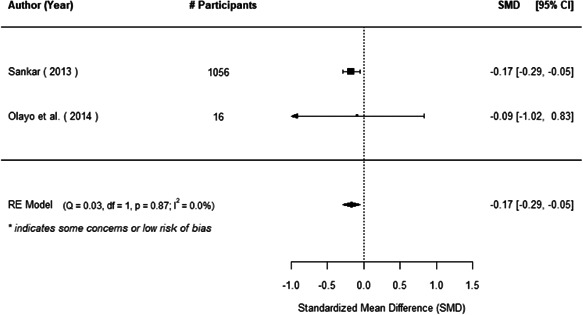
Forest plot showing the observed outcomes and the estimate of the random‐effects model for interventions with multiple engagement types on DPT1 vaccination. DPT, diphtheria, pertussis tetanus.

Only k = 2 studies reporting on DPT1 vaccination used interventions with multiple engagement types. The estimated average outcome based on the random‐effects model was $\hat{\mu }=-0.17$ ([95% CI: −0.29 to −0.05],z=−2.81, p=0.005), thus there was a significant negative impact of the programmes on DPT1 vaccination (see Figure 38). Given the small number of studies, this result should be interpreted with caution. According to the Q‐test, the true outcomes appear to be homogeneous (Q(1)=0.03, p=0.87, ${\hat{\tau }}^{2}=0.00$, $I^{2}=0.00$%). With only two studies, moderator analyses were not appropriate and tests of publication bias are not valid.

##### 6.3.1.6 DPT 2 vaccination

**Figure 39:**
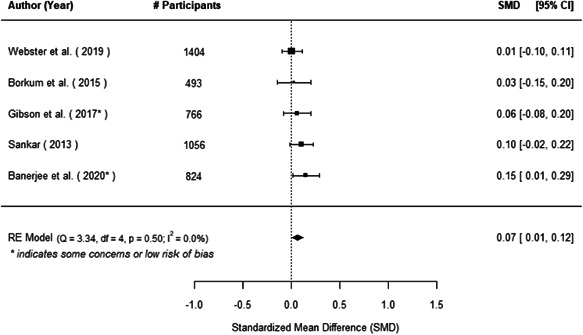
Forest plot showing the observed outcomes and the estimate of the random‐effects model for the impact of community engagement interventions on DPT2 vaccination. DPT, diphtheria, pertussis tetanus. This group of studies was not sufficiently powered for a robustness check using robust variance analysis (*df* = 3.51).

We included a total of k=5 studies in the analysis. The estimated average outcome based on the random‐effects model was $\hat{\mu }=0.07$ (95% CI: 0.01 to 0.12). Therefore, the average outcome differed significantly from zero (z=2.23, p=0.03), indicating very small but significant benefit to the treated group compared to the control group (see Figure 39). According to the Q‐test, there was no significant amount of heterogeneity in the true outcomes (Q(4)=3.34, p=0.50, ${\hat{\tau }}^{2}=0.00$, $I^{2}=0.00$%). An examination of the studentized residuals revealed that none of the studies had a value larger than ±2.58 and hence there was no indication of outliers in the context of this model. According to the Cook's distances, none of the studies could be considered to be overly influential. With no heterogeneity present, we did not examine potential sources of variation. When high risk of bias studies were removed, the resulting average effect increased slightly ($\hat{\mu }=0.11$ [95% CI: 0.01 to 0.20]) and was still significantly different from zero (z=2.12, p=0.03).

###### 6.3.1.6.1 Effects of engagement as the intervention on DPT 2 vaccination

Only one study (Banerjee et al., 2020) fell into this intervention/outcome category, thus we were unable to perform a statistical synthesis. This cluster RCT from India found a small but significant positive effect of their programme on DPT2 vaccination (*g* = 0.15 [95% CI: 0.01 to 0.29]), but like most studies, it was assessed as having a high risk of bias.

###### 6.3.1.6.2 Effects of engagement in the intervention design on DPT 2 vaccination

**Figure 40:**
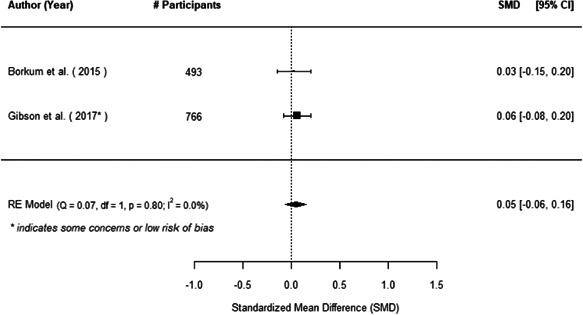
Forest plot showing the observed outcomes and the estimate of the random‐effects model for interventions with community engagement in the design on the intervention on DPT2 vaccination. DPT, diphtheria, pertussis tetanus.

Only two studies on DPT2 vaccination used interventions with engagement in the design. The estimated average outcome based on the random‐effects model was $\hat{\mu }=0.05$ (95% CI: −0.06 to 0.16). Therefore, the average outcome did not differ significantly from zero (z=0.83, p=0.41, see Figure 40), indicating no difference between the treatment group and the control group on DPT2 vaccination. Given the small number of studies, this result should be interpreted with caution. According to the Q‐test, the true outcomes appear to be homogeneous (Q(1)=0.07, p=0.41, ${\hat{\tau }}^{2}=0.00$, $I^{2}=0.00$%). With only two studies and no heterogeneity, moderator analyses were not appropriate and tests of publication bias are not valid.

###### 6.3.1.6.3 Effects of intervention with engagement in implementation autonomy on DPT 2 vaccination

Only one study (Webster et al., 2019) fell into this intervention/outcome category, thus we were unable to perform a statistical synthesis. This cluster RCT from Uganda found a null effect of their programme on DPT1 vaccination (*g* = 0.01 [95% CI: −0.10 to 0.11]), but like most studies, it was assessed as having a high risk of bias.

###### 6.3.1.6.4 Effects of interventions with multiple engagement types on DPT 2 vaccination

Only one study (Sankar, 2013) fell into this intervention/outcome category, thus we were unable to perform a statistical synthesis. This quasi‐experimental study from India found a null effect of their programme on OPV0 vaccination (*g* = 0.10 [95% CI: −0.02 to 0.22]), but like most studies, it was assessed as having a high risk of bias. Their programme included a combination of engagement in the implementation and engagement as the intervention.

##### 6.3.1.7 DPT 3 vaccination

**Figure 41:**
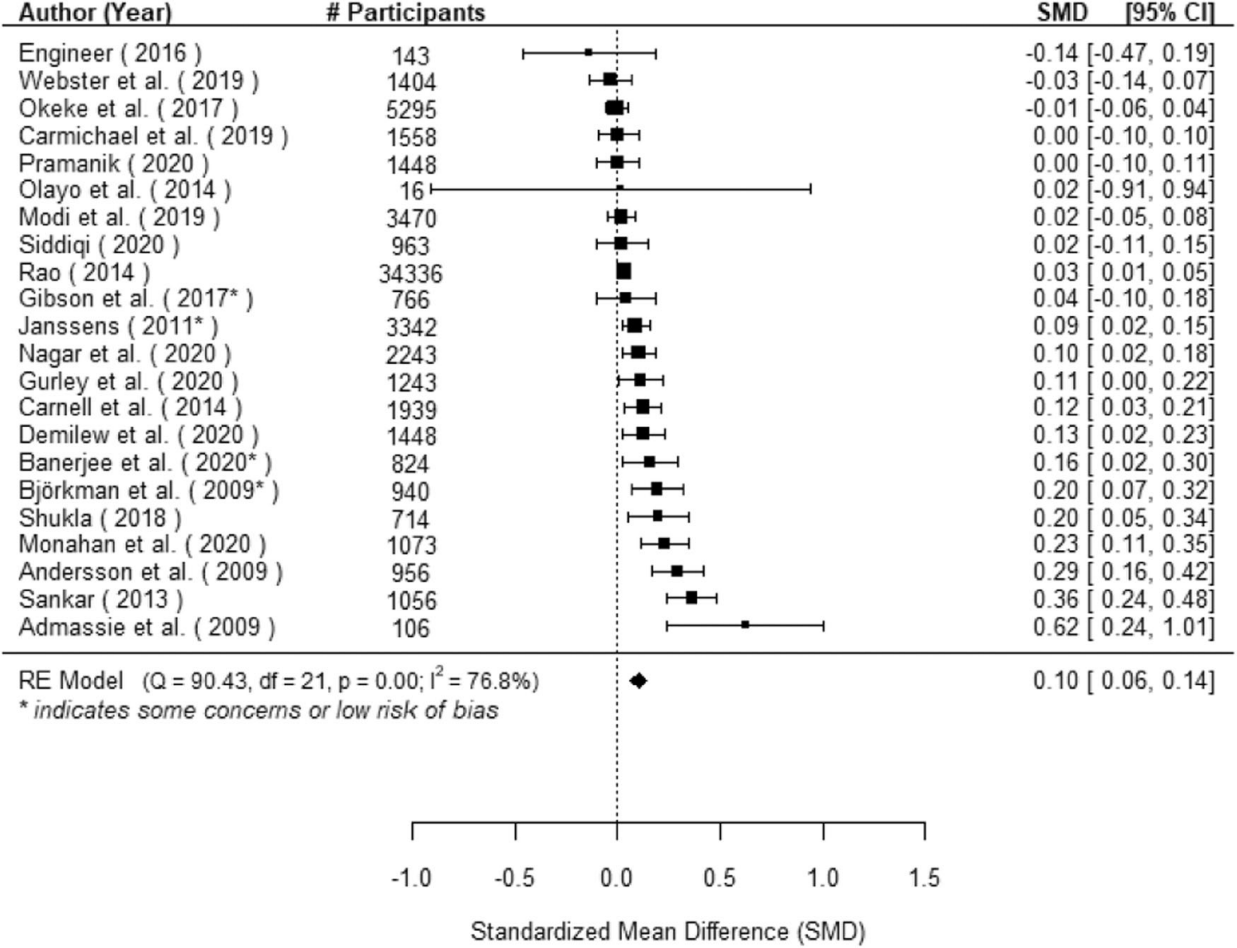
Forest plot showing the observed outcomes and the estimate of the random‐effects model for the impact of community engagement interventions on DPT3 vaccination. DPT, diphtheria, pertussis tetanus.

**Figure 42:**
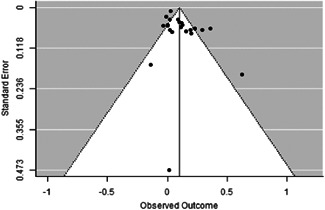
Funnel plot for studies examining the studies effect of community engagement interventions on DPT3 vaccination. DPT, diphtheria, pertussis tetanus.

**Figure 43:**
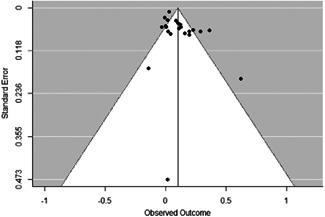
Trim and fill plot for examining the effect of community engagement interventions on DPT3 vaccination. DPT, diphtheria, pertussis tetanus.

**Figure 44:**
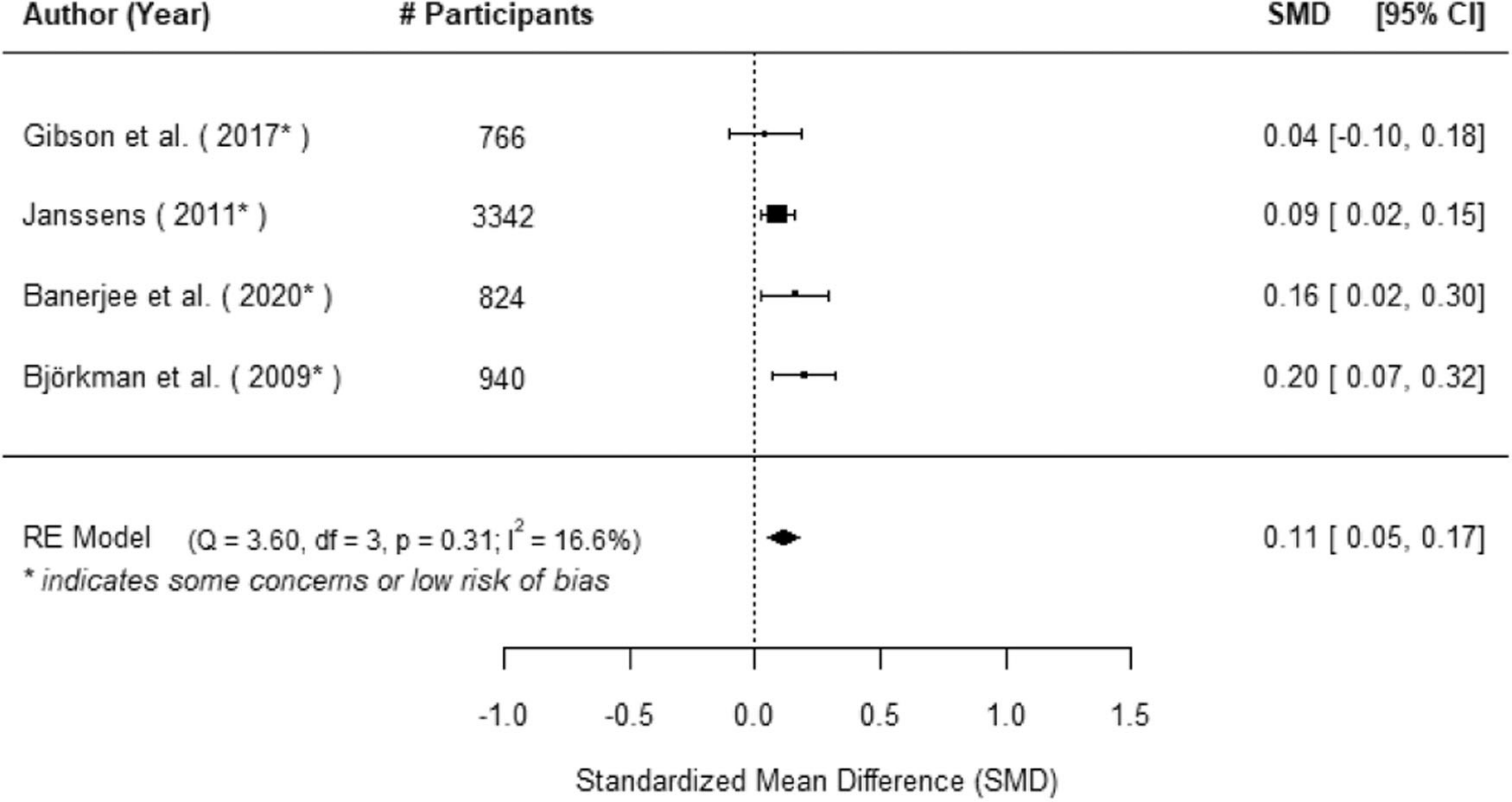
Forest plot showing the observed outcomes and the estimate of the random‐effects model for the impact of community engagement interventions on DPT3 vaccination when low quality studies are removed. DPT, diphtheria, pertussis tetanus.

A total of k=22 studies examined the relationship between community engagement interventions and DPT3 vaccinations. The estimated average outcome was $\hat{\mu }=0.10$ ([95% CI: 0.06 to 0.14], z=4.75, p<0.001) indicating a small but significant benefit to the treated group compared to the untreated group (Figure 41). The rank correlation test indicated funnel plot asymmetry (p=0.04) but not the regression test (p=0.06), but trim and fill analyses indicated an identical effect size (see Figures 42 and 43). When low quality studies were removed, the average effect increased slightly ($\hat{\mu }=0.11$ [95% CI: 0.05 to 0.17]), and was still statistically significant (z=3.70, p<0.001; see Figure 44). Only four studies were of high or moderate quality, so this analysis is based on a small sample of studies (Figure 44).

According to the Q‐test, the true outcomes appear to be heterogeneous (Q(21)=90.43, p<0.01, ${\hat{\tau }}^{2}=0.01$, $I^{2}=76.78$%). An examination of the studentized residuals revealed that none of the studies had a value larger than ±3.05 and hence there was no indication of outliers in the context of this model. According to the Cook's distances, none of the studies could be considered to be overly influential.

Publication year was a significant moderator such that each additional year reduced the size of the effect by 0.014 standard deviation units ($\hat{B}=-0.014,p=0.019$ [95% CI: −0.03 to −0.002]). In other words, new studies have found smaller effects. There were no other significant moderators in the context of this model (see Supporting Information: Appendix I Table 3).

As a robustness check, we used robust variance analysis and included all dependent effects in the analysis, totalling 36 effects from the same 22 studies (*df* = 18.2). The overall average effect was slightly smaller but still significant ($\hat{\mu }=0.10$ [95% CI: 0.05 to 0.15], *p* < .001). Sensitivity analyses show the effect to be sensitive to all values of *ρ*.

###### 6.3.1.7.1 Effects of engagement as the intervention on DPT 3 vaccination

**Figure 45:**
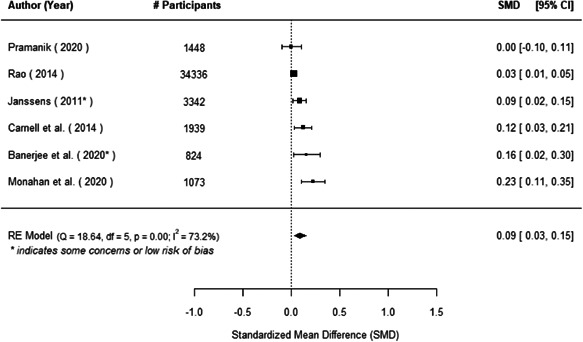
Forest plot showing the observed outcomes and the estimate of the random‐effects model for interventions with community engagement as the intervention on DPT3. DPT, diphtheria, pertussis tetanus.

We included a total of k=6 studies in the analysis. The observed outcomes ranged from 0.00 to 0.23. The estimated average outcome based on the random‐effects model was $\hat{\mu }=0.09$ (95% CI: 0.03 to 0.15). Therefore, the average outcome differed significantly from zero (z=3.01, p<0.01), indicating a benefit to the intervention participants compared to the control group. A forest plot showing the observed outcomes and the estimate based on the random‐effects model is shown in Figure 45.

According to the Q‐test, the true outcomes appear to be heterogeneous (Q(5)=18.64, p<0.01, ${\hat{\tau }}^{2}=0.00$, $I^{2}=73.17$%). An examination of the studentized residuals revealed that none of the studies had a value larger than ±2.64 and hence there was no indication of outliers in the context of this model. According to the Cook's distances, none of the studies could be considered to be overly influential. None of the moderators we tested were significant sources of heterogeneity (see Supporting Information: Appendix I Table 3).

###### 6.3.1.7.2 Effects of engagement in the intervention design on DPT 3 vaccination

**Figure 46:**
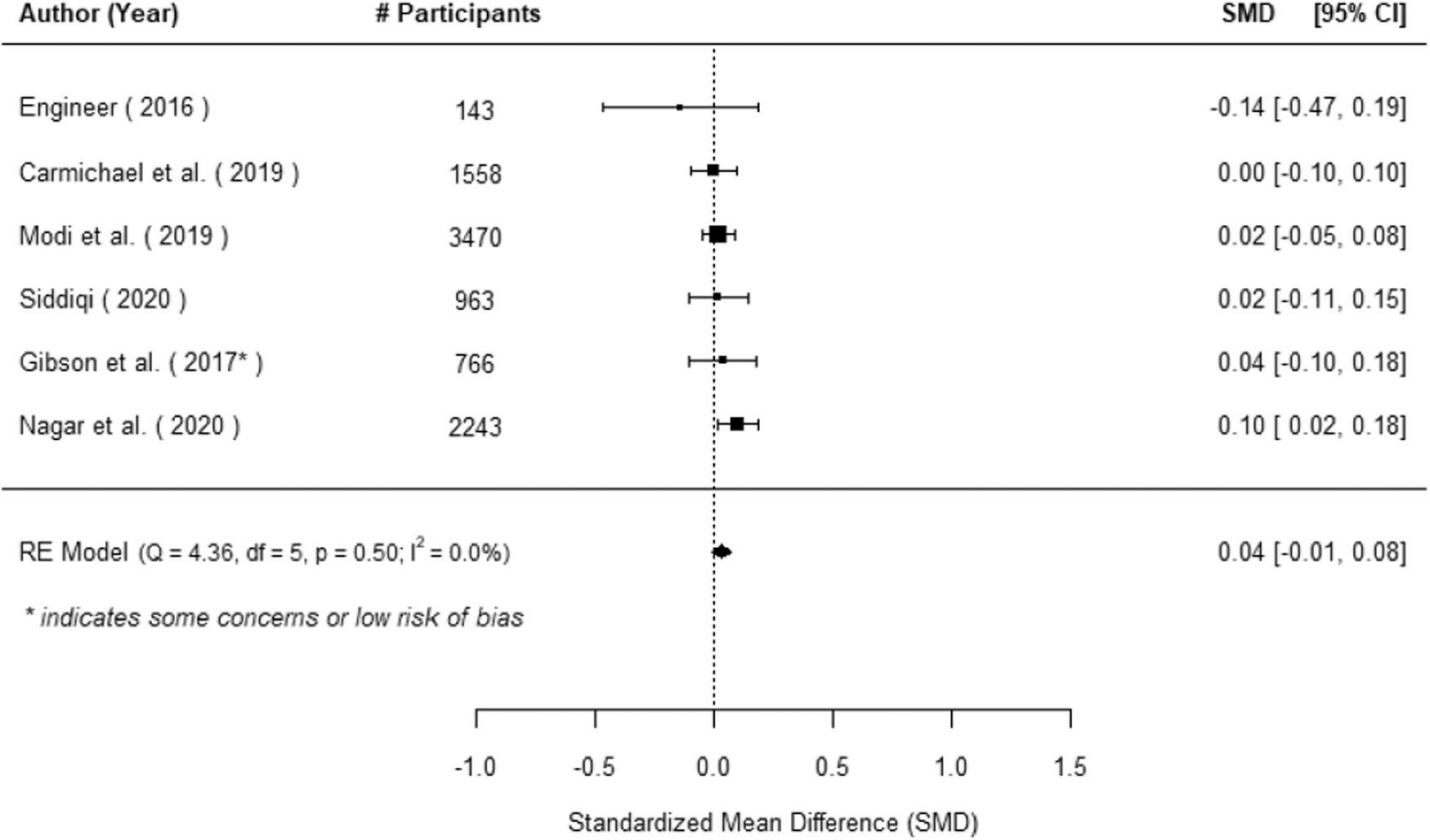
Forest plot showing the observed outcomes and the estimate of the random‐effects model for interventions with community engagement in the design on DPT3 vaccination. DPT, diphtheria, pertussis tetanus.

A total of k=6 studies were included in the analysis. The observed outcomes ranged from −0.14 to 0.10. The estimated average outcome based on the random‐effects model was 0.04 [95% CI: −0.01 to 0.08]. Therefore, the average outcome did not differ significantly from zero (z=1.69,p=0.09), indicating no benefit to intervention participants compared to control participants. A forest plot showing the observed outcomes and the estimate based on the random‐effects model is shown in Figure 46.

According to the Q‐test, there was no significant amount of heterogeneity in the true outcomes (Q(5)=4.36, p=0.50, ${\hat{\tau }}^{2}=0.00$, $I^{2}=0.00$%). An examination of the studentized residuals revealed that none of the studies had a value larger than ±2.64 and hence there was no indication of outliers in the context of this model. According to the Cook's distances, none of the studies could be considered to be overly influential. With no heterogeneity, we did not perform moderator analyses. With only one study of low or medium quality, we were also unable to complete sensitivity analyses for this body of evidence.

###### 6.3.1.7.3 Effects of engagement in implementation autonomy on DPT 3 vaccination

**Figure 47:**
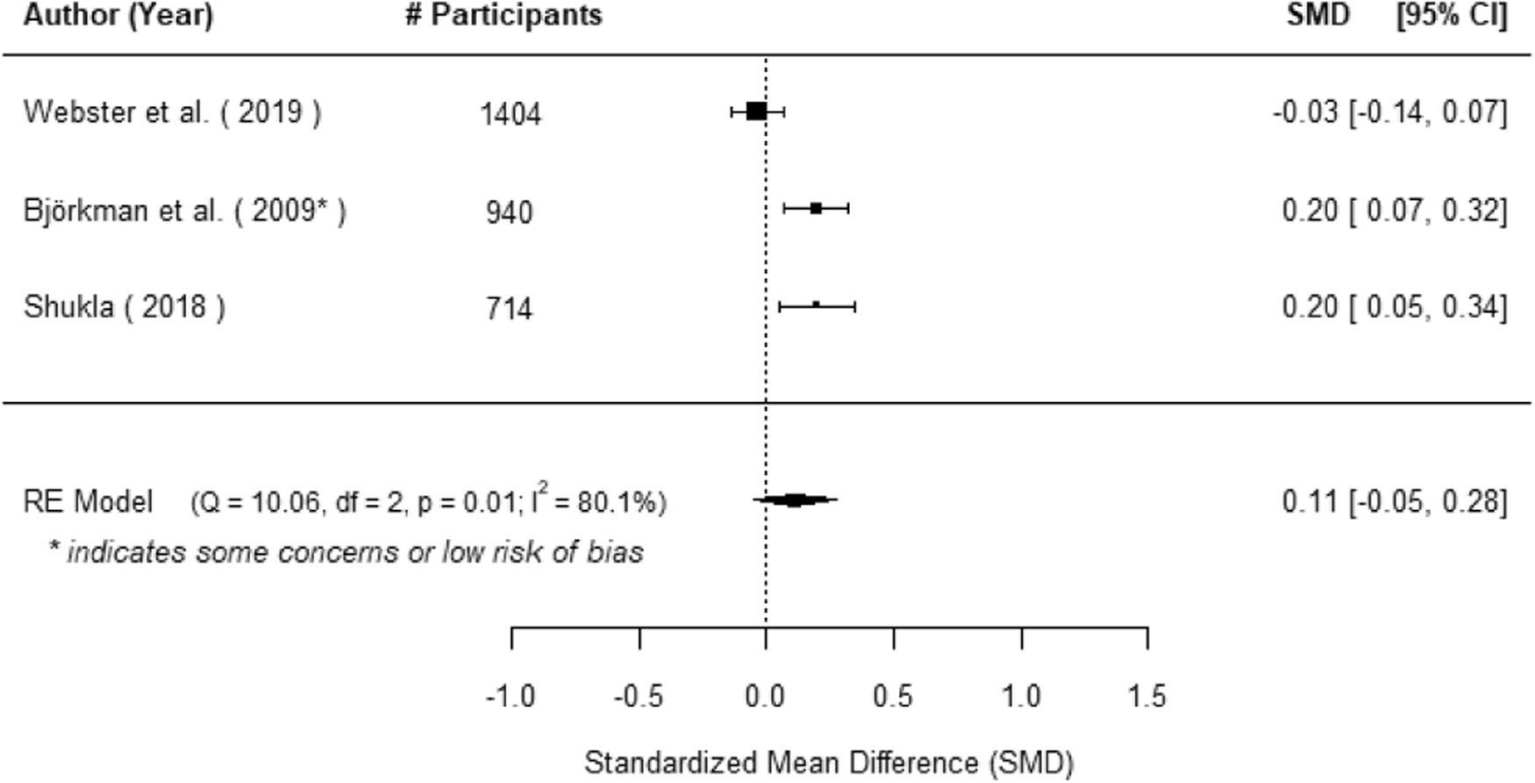
Forest plot showing the observed outcomes and the estimate of the random‐effects model for interventions with community engagement in implementation autonomy on DPT3 vaccination. DPT, diphtheria, pertussis tetanus.

Only three studies on DPT3 vaccination used interventions with engagement in implementation. The outcomes ranged from −0.03 to 0.20. The estimated average outcome based on the random‐effects model was $\hat{\mu }=0.11$ (95% CI: −005 to 0.28). Therefore, the average outcome did not differ significantly from zero (z=1.38, p=0.17) indicating no benefit to the intervention participants compared to the control participants. A forest plot showing the observed outcomes and the estimate based on the random‐effects model is shown in Figure 47. Given the small number of studies, this result should be interpreted with caution.

According to the Q‐test, the true outcomes appear to be heterogeneous (Q(2)=10.06, p<0.01, ${\hat{\tau }}^{2}=0.02$, $I^{2}=80.12$%). An examination of the studentized residuals revealed that one study (Webster, 2019) had a value larger than ±2.39 and may be a potential outlier in the context of this model. Indeed, sensitivity analyses leaving each study out indicated that removing Webster (2019) would increase the overall average effect ($\hat{\mu }$= 0.20 [95% CI: 0.10 to 0.29]), with the effect still positive and significant (*z* = 3.99, *p* < .001). According to the Cook's distances, none of the studies could be considered to be overly influential.

With only three studies, moderator analyses and tests of publication bias were not appropriate. With two of the three studies assessed as high risk of bias, we were also unable to conduct a sensitivity analysis by study quality.

###### 6.3.1.7.4 Effects of interventions with multiple engagement types on DPT 3 vaccination

**Figure 48:**
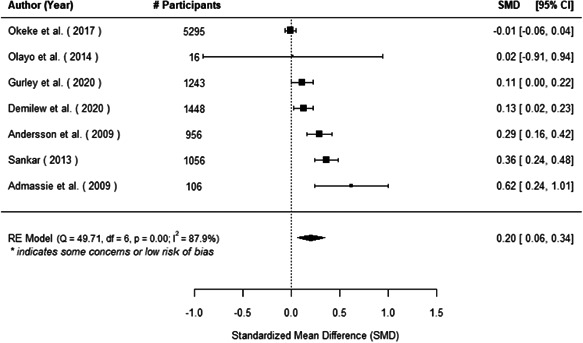
Forest plot showing the observed outcomes and the estimate of the random‐effects model for interventions with multiple engagement types on DPT3 vaccination. DPT, diphtheria, pertussis tetanus.

A total of k=8 studies were included in the analysis. The observed outcomes ranged from −0.01 to 0.62. The estimated average outcome based on the random‐effects model was $\hat{\mu }=0.20$ [95% CI: 0.06 to 0.34]). Therefore, the average outcome differed significantly from zero (z=2.85, p<0.01), indicating a benefit to the intervention participants compared to the control group. A forest plot showing the observed outcomes and the estimate based on the random‐effects model is shown in Figure 48.

According to the Q‐test, the true outcomes appear to be heterogeneous (Q(6)=49.71, p<0.01, ${\hat{\tau }}^{2}=0.03$, $I^{2}=87.93$%). An examination of the studentized residuals revealed that none of the studies had a value larger than ±2.69 and hence there was no indication of outliers in the context of this model. According to the Cook's distances, none of the studies could be considered to be overly influential. With no high or moderate quality studies, we were unable to perform a sensitivity analysis. There were several moderators that were significant sources of heterogeneity. Evaluation period was significant such that each additional month between the end of the intervention and the collection of outcome data reduced the size of the effect by 0.01 standard deviation units ($\hat{\beta }=-0.01,p=0.003$ [95% CI: −0.02 to −0.004]), suggesting smaller long term effect of the interventions. Publication year was also significant, such that each additional year reduced the size of the effect by 0.03 standard deviation units ($\hat{\beta }=-0.03,p=0.03$ [95% CI: −0.05 to −0.003]). In other words, more recent studies have found smaller effects. Finally, baseline DPT3 coverage rates were significant such that a one unit increase in baseline DPT3 coverage was associated with a decrease of.56 in the effect of the programme ($\hat{\beta }=-0.56,p=0.002$ [95% CI: −0.90 to −0.21]). In other words, the programmes were significantly more effective in areas with lower baseline coverage rates. We also tested whether the specific combination of engagement types led to different effects, but for DPT3 vaccination we found no differences by engagement packages ($\hat{\beta }=-0.07,p=0.66$ [95% CI: −0.40 to −0.25]).

###### 6.3.1.7.5 DPT3 vaccinations for girls

**Figure 49:**
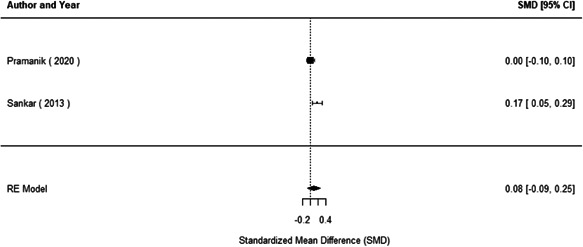
Forest plot showing the observed outcomes and the estimate of the random‐effects model for effects of community engagement on DPT3 immunisation for girls. DPT, diphtheria, pertussis tetanus.

Wherever possible, we sought to examine subgroups in cases where results of primary studies were disaggregated by group (e.g., child sex, SES, etc.). For DPT3 vaccinations, two studies reported disaggregated data for full immunisation for female children, thus *k* = 2 studies were included in the analysis. The estimated average outcome based on the random‐effects model was $\hat{\mu }=0.08$ (95% CI: −0.09to0.25). Therefore, the average outcome did not differ significantly from zero (z=0.96, p=0.33). A forest plot showing the observed outcomes and the estimate based on the random‐effects model is shown in Figure 49. Given the small number of studies, this result should be interpreted with caution. With only two studies, moderator analyses were not possible and tests of publication bias are not valid.

###### 6.3.1.7.6 DPT3 vaccinations for boys

**Figure 50:**
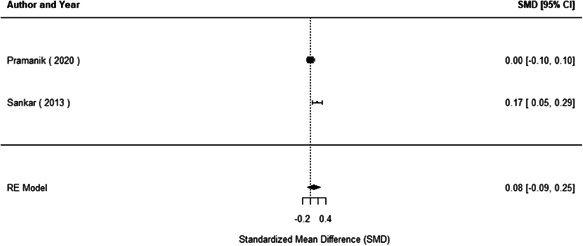
Forest plot showing the observed outcomes and the estimate of the random‐effects model for effects of community engagement on DPT3 immunisation for boys. DPT, diphtheria, pertussis tetanus.

Wherever possible, we sought to examine subgroups in cases where results of primary studies were disaggregated by group (e.g., child sex, SES, etc.). For full immunisation, two studies reported disaggregated data for full immunisation for female children, thus *k* = 2 studies were included in the analysis. The estimated average outcome based on the random‐effects model was $\hat{\mu }=0.08$ (95% CI: −0.09to0.25). Therefore, the average outcome did not differ significantly from zero (z=0.97, p=0.33). A forest plot showing the observed outcomes and the estimate based on the random‐effects model is shown in Figure 50. Given the small number of studies, this result should be interpreted with caution. With only two studies, moderator analyses were not possible and tests of publication bias are not valid.

##### 6.3.1.8 OPV0 vaccination

**Figure 51:**
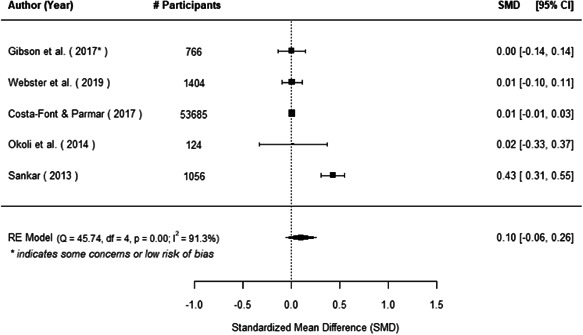
Forest plot showing the observed outcomes and the estimate of the random‐effects model for the impact of community engagement interventions on OPV0 vaccination. OPV, oral polio vaccine.

We included a total of k=5 studies in the analysis. The estimated average outcome based on the random‐effects model was $\hat{\mu }=0.10$ (95% CI: −0.06 to 0.26). Therefore, the average outcome did not differ significantly from zero (z=1.24, p=0.22), indicating no difference between the intervention and control groups (see Figure 51). According to the Q‐test, the true outcomes appear to be heterogeneous (Q(4)=45.74, p<0.01, ${\hat{\tau }}^{2}=0.03$, $I^{2}=91.26$%). An examination of the studentized residuals revealed that one study (Sankar, 2013) had a value larger than ±2.58 and may be a potential outlier in the context of this model. According to the Cook's distances, Sankar (2013) could also be considered to be overly influential. Indeed, sensitivity analyses leaving each study out indicated that removing Sankar (2013) would reduce the overall average effect ($\hat{\mu }$ = −0.01 [95% CI: −0.01 to 0.03]) which would become nonsignificant (z = 1.02, p = 0.31).

Exposure to the intervention (in months) was a significant predictor of variation on OPV0 vaccinations such that each additional month of exposure increased the size of the effect by 0.02 standard deviation units (*β* = .02, [95% CI: 0.01 to 0.03]; p < 0.001). Publication year was also a significant predictor of variation on OPV0 vaccinations such that each additional year decreased the size of the effect by 0.07 standard deviation units (*β* = −0.07, [95% CI: −0.11 to −0.02]; p = 0.002), meaning more recent studies found smaller effects than older studies. With only one study that was not assessed as high risk of bias, we could not conduct a sensitivity analysis by study quality.

This group of studies was not sufficiently powered for a robustness check using robust variance analysis (*df* = 3.76).

###### 6.3.1.8.1 Effects of engagement as the intervention on OPV0 vaccination

Only one study reporting on OPV0 vaccination used interventions with engagement in the design (Costa‐Font & Parmar, 2017), thus we were unable to perform a statistical synthesis. This quasi‐experimental study from India found a null effect of their programme on OPV0 vaccination (*g* = 0.01 [95% CI: −0.01 to 0.03]), but like most studies, it was assessed as having a high risk of bias.

###### 6.3.1.8.2 Effects of engagement in the intervention design on OPV0 vaccination

**Figure 52:**
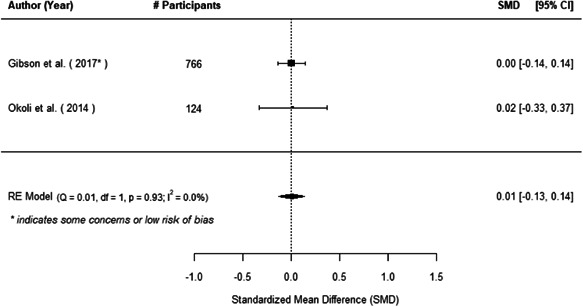
Forest plot showing the observed outcomes and the estimate of the random‐effects model for interventions with community engagement in the design on the intervention on OPV0 vaccination. OPV, oral polio vaccine.

Only two studies on OPV0 vaccination used interventions with engagement in the design. The estimated average outcome based on the random‐effects model was $\hat{\mu }=0.01$ (95% CI: −0.13 to 0.14). Therefore, the average outcome did not differ significantly from zero (z=0.08, p=0.94, see Figure 52). Given the small number of studies, this result should be interpreted with caution. According to the Q‐test, the true outcomes appear to be heterogeneous (Q(1)=0.01, p=0.93, ${\hat{\tau }}^{2}=0.00$, $I^{2}=0.00$%). With only two studies and no heterogeneity, moderator analyses were not appropriate and tests of publication bias are not valid.

###### 6.3.1.8.3 Effects of interventions with engagement in implementation autonomy on OPV0 vaccination

Only one study reporting on OPV0 vaccination used interventions with engagement in the implementation (Webster et al., 2019), thus we were unable to perform a statistical synthesis. This cluster RCT from Uganda found a null effect of their programme on OPV0 vaccination (*g* = 0.01 [95% CI: −0.10 to 0.11]), but like most studies, it was assessed as having a high risk of bias.

###### 6.3.1.8.4 Effects of interventions with multiple engagement types on OPV0 vaccination

Only one study reporting on OPV0 vaccination used interventions with multiple engagement types (Sankar, 2013), thus we were unable to perform a statistical synthesis. This quasi‐experimental study from India found a significant positive effect of their programme on OPV0 vaccination (*g* = 0.43 [95% CI: 0.31 to 0.55]), but like most studies, it was assessed as having a high risk of bias. Their programme included a combination of engagement in implementation autonomy and engagement as the intervention.

##### 6.3.1.9 OPV1 vaccination

**Figure 53:**
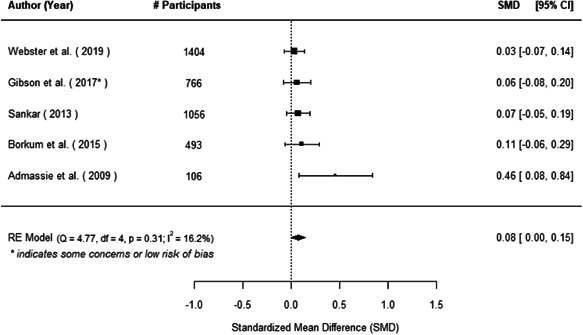
Forest plot showing the observed outcomes and the estimate of the random‐effects model for the impact of community engagement interventions on OPV1 vaccination. OPV, oral polio vaccine.

We included a total of k=5 studies in the analysis. The estimated average outcome based on the random‐effects model was $\hat{\mu }=0.08$ ([95% CI: 0.004 to 0.15],z=2.06, p=0.04), indicating a very small but significant benefit to the treated group compared to the control group (see Figure 53). According to the Q‐test, there was no significant amount of heterogeneity in the true outcomes (Q(4)=4.77, p=0.31, ${\hat{\tau }}^{2}=0.00$, $I^{2}=16.17$%), thus we did not test for sources of heterogeneity. An examination of the studentized residuals revealed that none of the studies had a value larger than ±2.58 and hence there was no indication of outliers in the context of this model. According to the Cook's distances, none of the studies could be considered to be overly influential.

This group of studies was not sufficiently powered for a robustness check using robust variance analysis (*df* = 3.02).

###### 6.3.1.9.1 Effects of engagement as the intervention on OPV1 vaccination

There were no studies reporting on OPV1 vaccination that used interventions with community engagement as the intervention.

###### 6.3.1.9.2 Effects of engagement in the intervention design on OPV1 vaccination

**Figure 54:**
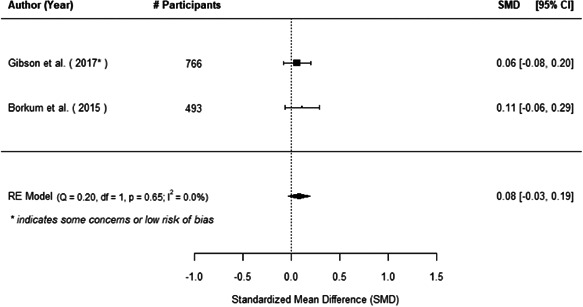
Forest plot showing the observed outcomes and the estimate of the random‐effects model for interventions with community engagement in the design on the intervention on OPV1 vaccination. OPV, oral polio vaccine.

Only two studies reporting on OPV1 vaccination used interventions with community engagement in the intervention design. The estimated average outcome based on the random‐effects model was $\hat{\mu }=0.08$ (95% CI: −0.03 to 0.19). Therefore, the average outcome did not differ significantly from zero (z=1.44, p=0.15, see Figure 54). Given the small number of studies, this result should be interpreted with caution. According to the Q‐test, the true outcomes appear to be homogeneous (Q(1)=0.20, p=0.65, ${\hat{\tau }}^{2}=0.00$, $I^{2}=0.00$%). With only two studies, moderator analyses were not appropriate and tests of publication bias are not valid.

###### 6.3.1.9.3 Effects of interventions with engagement in implementation autonomy on OPV1 vaccination

Only one study reporting on OPV1 vaccination used interventions with engagement in the implementation (Webster et al., 2019), thus we were unable to perform a statistical synthesis. This cluster RCT from Uganda found a null effect of their programme on OPV1 vaccination (*g* = 0.03 [95% CI: −0.07 to 0.14]), but like most studies, it was assessed as having a high risk of bias.

###### 6.3.1.9.4 Effects of interventions with multiple engagement types on OPV1 vaccination

**Figure 55:**
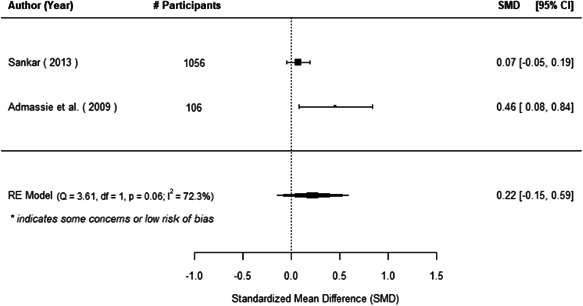
Forest plot showing the observed outcomes and the estimate of the random‐effects model for interventions with multiple engagement types on OPV1 vaccination. OPV, oral polio vaccine.

Only two studies on OPV1 vaccination used interventions with multiple engagement types. The estimated average outcome based on the random‐effects model was $\hat{\mu }=0.22$ (95% CI: −0.15 to 0.59). Therefore, the average outcome did not differ significantly from zero (z=1.17, p=0.24, see Figure 55). Given the small number of studies, this result should be interpreted with caution. According to the Q‐test, the true outcomes appear to be homogeneous (Q(1)=3.61, p=0.06, ${\hat{\tau }}^{2}=0.05$, $I^{2}=72.32$%). With only two studies, moderator analyses were not possible and tests of publication bias are not valid.

##### 6.3.1.10 OPV2 vaccination

**Figure 56:**
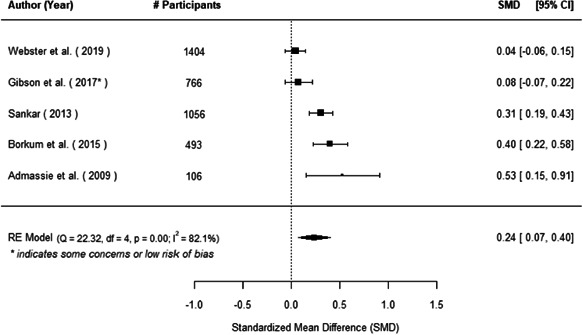
Forest plot showing the observed outcomes and the estimate of the random‐effects model for the impact of community engagement interventions on OPV2 vaccination. OPV, oral polio vaccine.

We included a total of k=5 studies in the analysis. The estimated average outcome based on the random‐effects model was $\hat{\mu }=0.24$ ([95% CI: 0.07 to 0.40],z=2.84, p<0.01), indicating a moderate but significant benefit to the treated group compared to the control group (see Figure 56). According to the Q‐test, the true outcomes appear to be heterogeneous (Q(4)=22.32, p<0.01, ${\hat{\tau }}^{2}=0.03$, $I^{2}=82.08$%). An examination of the studentized residuals revealed that none of the studies had a value larger than ±2.58 and hence there was no indication of outliers in the context of this model. According to the Cook's distances, none of the studies could be considered to be overly influential. With only one study that was not assessed as high risk of bias, we could not conduct a sensitivity analysis by study quality.

We tested for potential sources of heterogeneity and found several significant moderators in the context of this model (see Supporting Information: Appendix I Table 3). Exposure to intervention was significant such that each additional month of exposure increased the average effect by 0.02 standard deviation units (β=0.02 [95% CI: 0.01 to 0.02], p < 0.0001). In other words, longer interventions produced larger effects. Publication year was also significant such that older studies reported larger effects than more recent studies. Specifically, each additional year decreased the average effect by 0.05 standard deviation units (β=−0.05 [95% CI: −0.08 to −0.02], p < 0.001). Finally, region was a significant predictor such that Sub‐Saharan Africa had smaller effects that South Asia by 0.26 standard deviation units (*β* =−0.26 [95% CI: −0.39 to −0.13], p < 0.001).

This group of studies was not sufficiently powered for a robustness check using robust variance analysis (*df* = 3.75).

###### 6.3.1.10.1 Effects of engagement as the intervention on OPV2 vaccination

There were no studies reporting on OPV2 vaccination that used interventions with community engagement as the intervention.

###### 6.3.1.10.2 Effects of engagement in the intervention design on OPV2 vaccination

**Figure 57:**
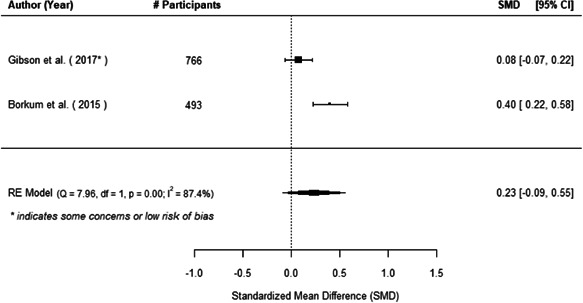
Forest plot showing the observed outcomes and the estimate of the random‐effects model for interventions with community engagement in the design on the intervention on OPV2 vaccination. OPV, oral polio vaccine.

Only *k* = 2 studies reporting on OPV2 vaccination used interventions with community engagement in the intervention design. The estimated average outcome based on the random‐effects model was $\hat{\mu }=0.23$ (95% CI: −0.09 to 0.55). Therefore, the average outcome did not differ significantly from zero (z=1.43, p=0.15, see Figure 57), indicating no significant difference between the intervention group and the control group on OPV2 vaccinations. Given the small number of studies, this result should be interpreted with caution. According to the Q‐test, the true outcomes appear to be heterogeneous (Q(1)=7.96, p=0.004, ${\hat{\tau }}^{2}=0.05$, $I^{2}=87.44$%). With only two studies, moderator analyses were not appropriate and tests of publication bias are not valid.

###### 6.3.1.10.3 Effects of intervention with engagement in implementation autonomy on OPV2 vaccination

Effects of intervention with engagement in implementation autonomy on OPV2 vaccination

Only one study reporting on OPV2 vaccination used interventions with engagement in the implementation (Webster et al., 2019), thus we were unable to perform a statistical synthesis. This cluster RCT from Uganda found a null effect of their programme on OPV2 vaccination (*g* = 0.04 [95% CI: −0.06 to 0.15]), but again, it was assessed as having a high risk of bias.

###### 6.3.1.10.4 Effects of interventions with multiple engagement types on OPV2 vaccination

**Figure 58:**
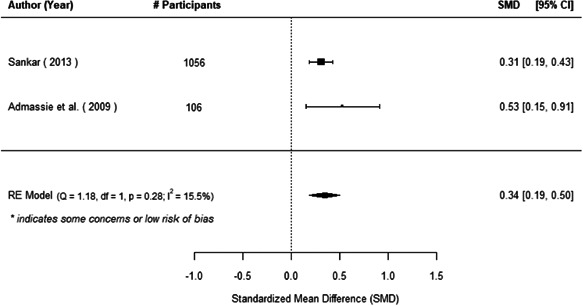
Forest plot showing the observed outcomes and the estimate of the random‐effects model for interventions with multiple engagement types on OPV2 vaccination. OPV, oral polio vaccine.

Only two studies on OPV2 vaccination used interventions with multiple engagement types. The estimated average outcome based on the random‐effects model was $\hat{\mu }=0.34$ (95% CI: 0.19 to 0.50,z=4.28, p<0.001), indicating a moderate and significant benefit to the treated group compared to the control participants (see Figure 58). Given the small number of studies, this result should be interpreted with caution. According to the Q‐test, the true outcomes appear to be homogeneous (Q(1)=1.18, p=0.28, ${\hat{\tau }}^{2}=0.004$, $I^{2}=15.51$%). With only two studies, moderator analyses were not appropriate and tests of publication bias are not valid. Both studies used a combination of engagement in implementation autonomy and engagement as the intervention.

##### 6.3.1.11 OPV3 vaccination

**Figure 59:**
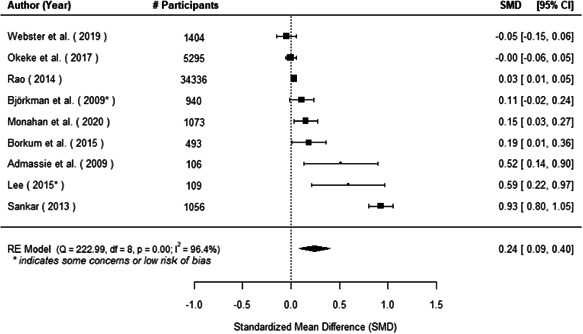
Forest plot showing the observed outcomes and the estimate of the random‐effects model for the impact of community engagement interventions on OPV3 vaccination. OPV, oral polio vaccine.

We included total of k=9 studies in the analysis. The estimated average outcome based on the random‐effects model was $\hat{\mu }=0.24$ ([95% CI: 0.09 to 0.40],z=3.06, p=0.002), indicating a moderate and significant benefit to the intervention group compared to the control group (see Figure 59). According to the Q‐test, the true outcomes appear to be heterogeneous (Q(8)=222.99, p<0.001, ${\hat{\tau }}^{2}=0.05$, $I^{2}=96.41$%). An examination of the studentized residuals revealed that one study (Sankar, 2013) had a value larger than ±2.77 and may be a potential outlier in the context of this model. According to the Cook's distances, one study (Sankar, 2013) could be considered to be overly influential. Sensitivity analyses leaving each study out indicated that removing Sankar (2013) would substantially reduce the overall average effect ($\hat{\mu }$ = 0.08 [95% CI: 0.01 to 0.15]), but the effect would still be positive and significant (z = 2.39, p = 0.02). When low quality studies were removed, the resulting effect increased, but was no longer significant ($\hat{\hat{\mu }}=0.32$ [95% CI: −0.15 to 0.79],z=1.32, p=0.19). With only two studies of high or moderate quality, this result should be interpreted with caution. There were no significant sources of heterogeneity (see Supporting Information: Appendix I Table 3).

As a robustness check, we used robust variance analysis and included all dependent effects in the analysis, totalling 10 effects from the same 9 studies (*df* = 6.74). The overall average effect was identical, but no longer significant ($\hat{\mu }=0.24$ [95% CI: −0.02 to 0.51], *p* = .06). Sensitivity analyses show the effect to be sensitive to all values of *ρ*.

###### 6.3.1.11.1 Effects of engagement as the intervention on OPV3 vaccination

**Figure 60:**
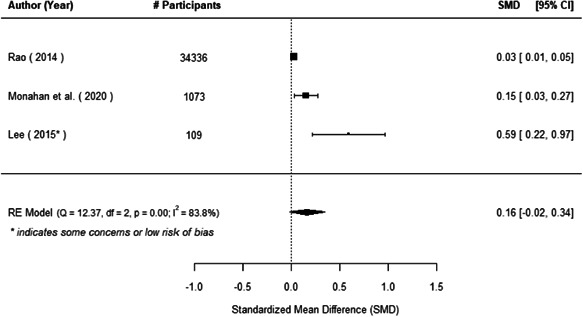
Forest plot showing the observed outcomes and the estimate of the random‐effects model for interventions with community engagement as the intervention on OPV3 vaccination. OPV, oral polio vaccine.

We included a total of k=3 studies in the analysis. The observed outcomes ranged from 0.03 to 0.59. The estimated average outcome based on the random‐effects model was $\hat{\mu }=0.16$ ([95% CI: −0.02 to 0.34],z=1.78, p=0.07), indicating no significant difference between the intervention group and the control group on OPV3 vaccination (see Figure 60). According to the Q‐test, the true outcomes appear to be heterogeneous (Q(2)=12.37, p=0.002, ${\hat{\tau }}^{2}=0.02$, $I^{2}=83.83$%). An examination of the studentized residuals revealed that one study (Lee, 2015) had a value larger than ±2.39 and may be a potential outlier in the context of this model. Indeed, sensitivity analyses leaving each study out indicated that removing Lee (2015) would reduce the overall average effect ($\hat{\mu }=0.08[$95% CI: −0.04 to 0.19]), though in either case the effect is not significantly different from zero (z=1.29, p=0.20). According to the Cook's distances, none of the studies could be considered to be overly influential. With only three studies contributing effects, moderator analyses were not appropriate. Two of the three studies were assessed as high risk of bias, so we were unable to conduct a sensitivity analysis by study quality.

###### 6.3.1.11.2 Effects of engagement in the intervention design on OPV3 vaccination

Only one study reporting on OPV3 vaccination used an intervention with engagement in the design (Borkum et al., 2014), thus we were unable to perform a statistical synthesis. This cluster RCT from India found a small but significant positive effect of their programme on OPV3 vaccination (*g* = 0.19 [95% CI: 0.01 to 0.36]), but again, it was assessed as having a high risk of bias.

###### 6.3.1.11.3 Effects of intervention with engagement in implementation autonomy on OPV3 vaccination

**Figure 61:**
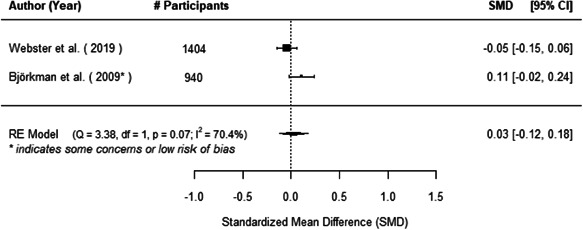
Forest plot showing the observed outcomes and the estimate of the random‐effects model for interventions with community engagement in implementation autonomy on OPV3 vaccinations. OPV, oral polio vaccine.

Only two studies examined the impact of interventions with community engagement in implementation autonomy on OPV3 vaccination. The estimated average outcome was $\hat{\mu }=0.03$ [95% CI: −0.12 to 0.18], which was not significant (z=0.35, p=0.73), indicating there was no difference between the intervention group and the control group. According to the Q‐test, the true outcomes appear to be homogeneous (Q(1)=3.38, p=0.07, ${\hat{\tau }}^{2}=0.01$, $I^{2}=70.38$%; see Figure 61). With only two studies we were unable to test for publication bias or sources of heterogeneity.

###### 6.3.1.11.4 Effects of interventions with multiple engagement types on OPV3 vaccination

**Figure 62:**
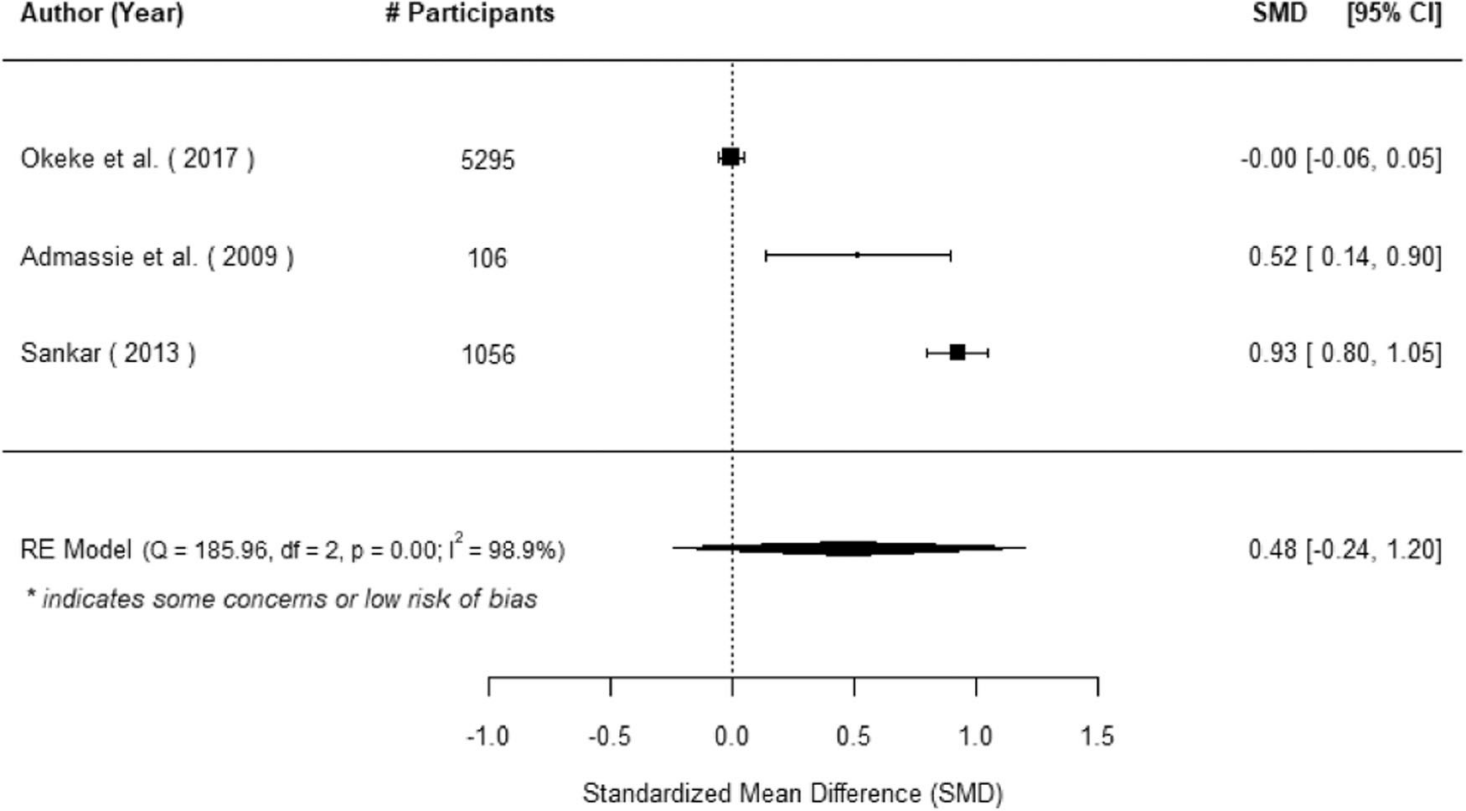
Forest plot showing the observed outcomes and the estimate of the random‐effects model for interventions with multiple engagement types on OPV3 vaccination. OPV, oral polio vaccine.

We included a total of k=3 studies in the analysis. The estimated average outcome based on the random‐effects model was $\hat{\mu }=0.48$ ([95% CI: −0.24 to 1.20],z=1.30, p=0.19), indicating no difference between the intervention group and the control group (see Figure 62). According to the Q‐test, the true outcomes appear to be heterogeneous (Q(2)=185.96, p<0.001, ${\hat{\tau }}^{2}=0.39$, $I^{2}=98.92$%). An examination of the studentized residuals revealed that none of the studies had a value larger than ±2.39 and hence there was no indication of outliers in the context of this model. According to the Cook's distances, none of the studies could be considered to be overly influential. With only three studies contributing effects, we were unable to examine sources of heterogeneity.

##### 6.3.1.12 Vaccination timeliness

We examined all outcomes where the study reported effects on vaccination timeliness. A total of 11 studies contributed 54 effects, and the average effect was computed using robust variance estimation. The estimated average outcome based on the random‐effects model was $\hat{\mu }=0.11$ ([95% CI: 0.06 to 0.16], p = 0.001), indicating a small but significant benefit to the intervention group compared to the control group. Sensitivity analyses showed that the effect was robust to all values of *ρ*.

There was a moderate amount of heterogeneity among the effects (*I* ^2^ = 39.92), which we examined using the same potential moderators discussed previously. However, the moderators were not powered for this analysis (see Supporting Information: Appendix I Table 3). Timeliness was a bit different than the other categories, in that the selection of independent effects could not be completed using our stated selection rules. In this case, studies would report on timeliness for specific vaccines. Thus, here we selected independent effects which reported on three outcomes: (1) timeliness of the third Pentavalent vaccination; (2) timeliness of the measles vaccination; and (3) timeliness of completing the full immunisation battery. These three separate analyses are discussed in turn below.

###### 6.3.1.12.1 Timeliness of Pentavalent 3 vaccination

**Figure 63:**
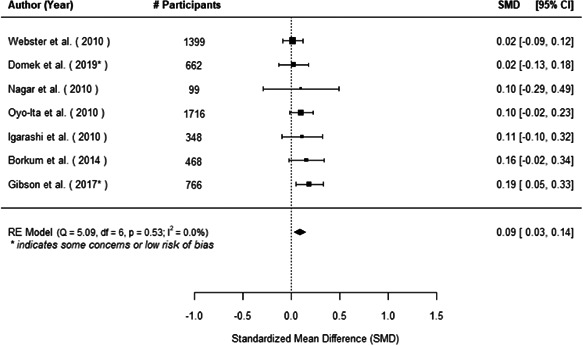
Forest plot showing the observed outcomes and the estimate of the random‐effects model for the impact of community engagement interventions on the timeliness of DPT3 vaccination. DPT, diphtheria, pertussis tetanus.

A total of k=7 studies examined the relationship between community engagement interventions and timeliness of DPT3 vaccination. The estimated average outcome was $\hat{\mu }=0.09$ ([95% CI: 0.03 to 0.14],z=3.00, p<0.01), indicating a very small but significant benefit to the intervention group compared to the control group (Figure 63). When low quality studies are removed, only two studies remain, and the estimated average outcome increases to $\hat{\mu }=0.11$ [95% CI: −0.05 to 0.27], but becomes nonsignificant z=1.32, p=0.19). However, with only two studies this should be interpreted with caution.

According to the Q‐test, there was no significant amount of heterogeneity in the true outcomes (Q(6)=5.09, p=0.53, ${\hat{\tau }}^{2}=0.00$, $I^{2}=0.00$%). An examination of the studentized residuals revealed that none of the studies had a value larger than ±2.69 and hence there was no indication of outliers in the context of this model. According to the Cook's distances, none of the studies could be considered to be overly influential. With no heterogeneity, moderator analyses were not appropriate. There were no dependent effects, so we could not use RVE as a robustness check in this case.

###### 6.3.1.12.2 Effects of engagement as the intervention on timeliness of DPT3 vaccination

No studies examining timeliness of DPT3 vaccinations used engagement as the intervention.

###### 6.3.1.12.3 Effects of engagement in the intervention design on timeliness of DPT3 vaccination

**Figure 64:**
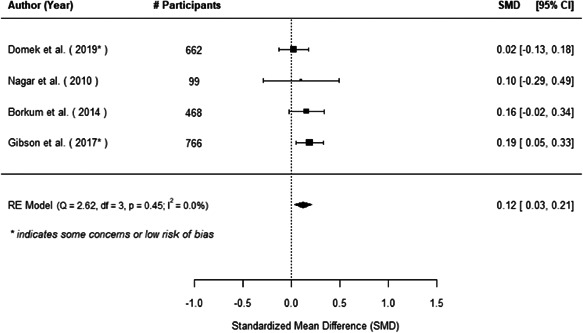
Forest plot showing the observed outcomes and the estimate of the random‐effects model for interventions with community engagement in the design of the intervention on the timeliness of DPT3 vaccinations. DPT, diphtheria, pertussis tetanus.

We included a total of k=4 studies in the analysis. The observed outcomes ranged from 0.02 to 0.19. The estimated average outcome based on the random‐effects model was $\hat{\mu }=0.12$ (95% CI: 0.03 to 0.21). Therefore, the average outcome differed significantly from zero (z=2.73, p<0.01). A forest plot showing the observed outcomes and the estimate based on the random‐effects model is shown in Figure 64.

According to the Q‐test, there was no significant amount of heterogeneity in the true outcomes (Q(3)=2.62, p=0.45, ${\hat{\tau }}^{2}=0.00$, $I^{2}=0.00$%). An examination of the studentized residuals revealed that none of the studies had a value larger than ±2.50 and hence there was no indication of outliers in the context of this model. According to the Cook's distances, none of the studies could be considered to be overly influential. With no heterogeneity indicated, we did not test for moderation.

###### 6.3.1.12.4 Effects of interventions with engagement in implementation autonomy on timeliness of DPT3 vaccination

**Figure 65:**
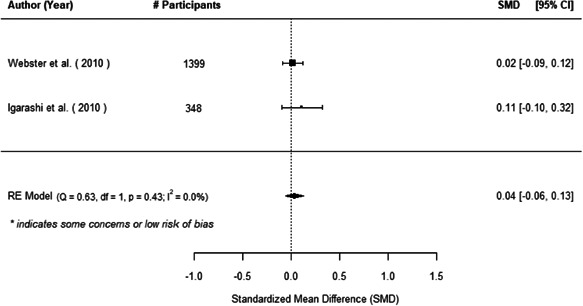
Forest plot showing the observed outcomes and the estimate of the random‐effects model for interventions with community engagement in implementation autonomy on DPT3 vaccination timeliness. DPT, diphtheria, pertussis tetanus.

We included a total of k=2 studies in the analysis. The observed outcomes ranged from 0.02 to 0.19. The estimated average outcome based on the random‐effects model was $\hat{\mu }=0.04$ [95% CI: −0.06 to 0.13],z=0.75, p=0.45), indicating no difference between the intervention and control groups. A forest plot showing the observed outcomes and the estimate based on the random‐effects model is shown in Figure 65.

According to the Q‐test, the true outcomes appear to be homogeneous (Q(1)=0.63, p=0.45, ${\hat{\tau }}^{2}=0.00$, $I^{2}=0.00$%). There was no indication of outliers in the context of this model, and with only two studies, we could not test for moderation. Both studies were assessed as having a high risk of bias.

###### 6.3.1.12.5 Effects of interventions with multiple engagement types on timeliness of DPT3 vaccination

Only one study reporting on timeliness of DPT3 vaccination used an intervention with multiple engagement types (Oyo‐Ita et al., 2020). This RCT conducted in Nigeria found a small but not‐significant positive effect (*g* = 0.10 [95% CI: −0.2 to 0.23). This study was assessed as having a high risk of bias.

###### 6.3.1.12.6 Timeliness of Measles vaccination

**Figure 66:**
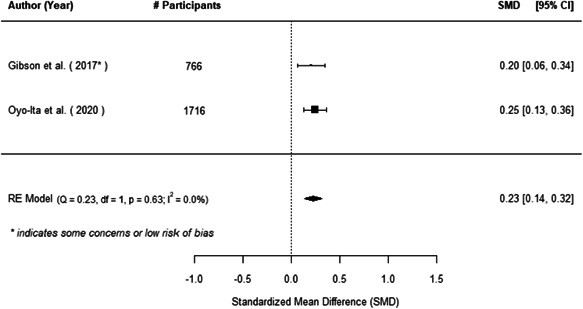
Forest plot showing the observed outcomes and the estimate of the random‐effects model for the impact of community engagement interventions on the timeliness of measles vaccination.

Only k=2 studies using community engagement interventions reported on the timeliness of measles vaccinations. The estimated average outcome was $\hat{\mu }=0.23$ ([95% CI: 0.14 to 0.32],z=5.06, p<0.001; see Figure 66), indicating a moderate but significant benefit to the treated group compared to the control group. With only two studies we were unable to conduct sensitivity analyses by study quality.

**Figure 67:**
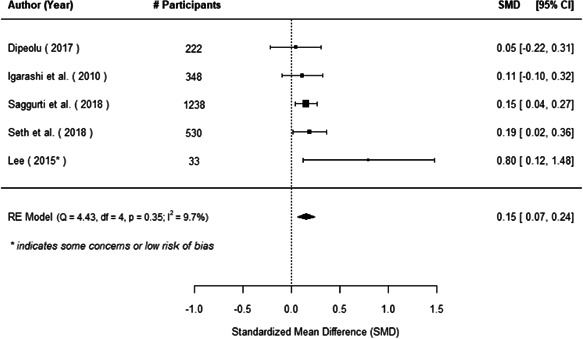
Forest plot showing the observed outcomes and the estimate of the random‐effects model for the impact of community engagement interventions on full childhood immunisation.

According to the Q‐test, there was no significant amount of heterogeneity in the true outcomes (Q(1)=0.23., p=0.63, ${\hat{\tau }}^{2}=0.00$, $I^{2}=0.00$%). An examination of the studentized residuals revealed that none of the studies had a value larger than ±2.69 and hence there was no indication of outliers in the context of this model. According to the Cook's distances, none of the studies could be considered to be overly influential. With no heterogeneity and only two studies contributing effects, moderator analyses were not appropriate. There were no dependent effects, so we could not use RVE as a robustness check in this case (Figures 66 and 67).

###### 6.3.1.12.7 Effects of engagement as the intervention on timeliness of Measles vaccination

No studies examining timeliness of measles vaccinations used engagement as the intervention.

###### 6.3.1.12.8 Effects of engagement in the intervention design on timeliness of Measles vaccination

Only one study reporting on timeliness of measles vaccination used an intervention with engagement in the design (Gibson et al., 2017). This RCT conducted in Kenya found a small but significant positive effect (*g* = 0.20 [95% CI: 0.06 to 0.34]). This study was assessed as having some concerns of bias.

###### 6.3.1.12.9 Effects of interventions with engagement in implementation autonomy on timeliness of Measles vaccination

No studies reporting on the timeliness of measles vaccinations used community engagement in implementation autonomy.

###### 6.3.1.12.10 Effects of interventions with multiple engagement types on timeliness of Measles vaccination

Finally, only one study reporting on timeliness of measles vaccination used an intervention with multiple engagement types (Oyo‐Ita et al., 2020). This RCT conducted in Nigeria found a moderate and significant positive effect (*g* = 0.25 [95% CI: 0.13 to 0.36]). They used a combination of engagement in implementation autonomy and engagement as the intervention. This study was also assessed as having a high risk of bias.

###### 6.3.1.12.11 Timeliness of full immunisation completion

We included a total of k=5 studies in the analysis. The estimated average outcome was $\hat{\mu }=0.15$ ([95% CI: 0.07 to 0.24],z=3.41, p<0.001; see Figure 67), indicating a small but significant benefit to the treated group compared to the control group. With only one study not assessed as high risk of bias, we were unable to conduct sensitivity analyses by study quality.

According to the Q‐test, there was no significant amount of heterogeneity in the true outcomes (Q(4)=4.43, p=0.35, ${\hat{\tau }}^{2}=0.00$, $I^{2}=9.66$%). An examination of the studentized residuals revealed that none of the studies had a value larger than ±2.58 and hence there was no indication of outliers in the context of this model. According to the Cook's distances, none of the studies could be considered to be overly influential. With no significant heterogeneity, we did not test for moderations. There were no dependent effects, so we could not use RVE as a robustness check in this case.

###### 6.3.1.12.12 Effects of engagement as the intervention on timeliness of full childhood immunisation

Only one study (Saggurti et al., 2018) fell into this intervention/outcome category, thus we were unable to perform a statistical synthesis. This quasi‐experimental study from India found a small but significant impact of their programme on full childhood immunisation (*g* = 0.15 [95% CI: 0.04 to 0.27]), but like most studies, it was assessed as having a high risk of bias.

###### 6.3.1.12.13 Effects of engagement in the intervention design on timeliness of full childhood immunisation

**Figure 68:**
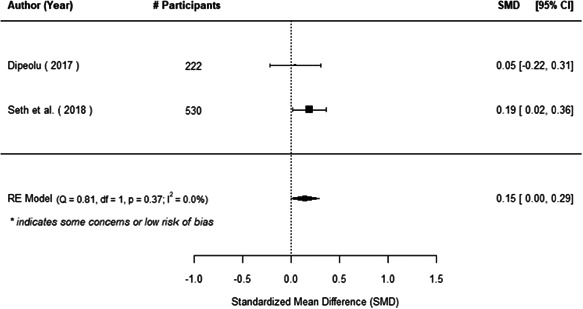
Forest plot showing the observed outcomes and the estimate of the random‐effects model for interventions with community engagement in the design on the timeliness of full childhood immunisation.

Two studies using engagement in the intervention design reported on the timeliness of full childhood immunisation. There was a significant effect on timeliness of full childhood immunisation ($\hat{\mu }=0.15$ [95% CI: 0.004 to 0.29],z=2.01, p=0.04), but this result should be interpreted with caution as it is based on only two studies (Figure 68). According to the Q‐test, there was no significant amount of heterogeneity in the true outcomes (Q(1)=0.81, p=0.37, ${\hat{\tau }}^{2}=0.00$, $I^{2}=0.00$%). Both studies were assessed as high risk of bias. With only two studies and no heterogeneity, moderator analyses were not appropriate.

###### 6.3.1.12.14 Effects of interventions with engagement in implementation autonomy on timeliness of full childhood immunisation

**Figure 69:**
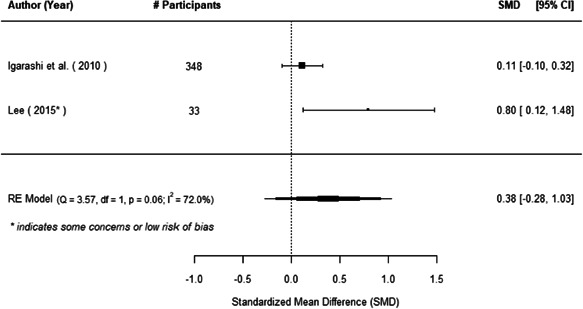
Forest plot showing the observed outcomes and the estimate of the random‐effects model for interventions with community engagement in implementation autonomy on DPT3 vaccination. DPT, diphtheria, pertussis tetanus.

Two studies using engagement in implementation autonomy reported on the timeliness of full childhood immunisation. There was no significant effect on timeliness of full childhood immunisation ($\hat{\mu }=0.38$ ([95% CI: −0.28 to 1.03],z=2.01, p=0.04), but this result should be interpreted with caution as it is based on only two studies (Figure 69).

According to the Q‐test, the true outcomes appear to be homogeneous (Q(1)=3.57, p=0.06, ${\hat{\tau }}^{2}=0.17$, $I^{2}=72.02$%). There was no indication of outliers in the context of this model, and with only two studies, we could not test for moderation. One study was assessed as having a high risk of bias (Igarashi et al., 2010) while the other was assessed as some concerns related to risk of bias (Lee, 2015).

###### 6.3.1.12.15 Effects of engagement as the intervention on timeliness of full childhood immunisation

Only one study (Saggurti et al., 2018) fell into this intervention/outcome category, thus we were unable to perform a statistical synthesis. This quasi‐experimental study from India found a small but significant impact of their programme on full childhood immunisation (*g* = 0.15 [95% CI: 0.04 to 0.27]), but like most studies, it was assessed as having a high risk of bias.

###### 6.3.1.12.16 Effects of engagement in the intervention design on timeliness of full childhood immunisation

**Figure 70:**
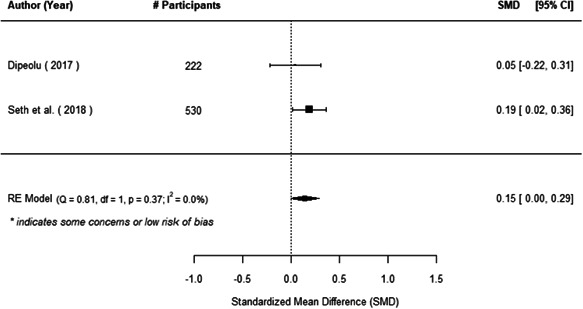
Forest plot showing the observed outcomes and the estimate of the random‐effects model for interventions with community engagement in the design on the timeliness of full childhood immunisation.

Two studies using engagement in the intervention design reported on the timeliness of full childhood immunisation. There was a significant effect on timeliness of full childhood immunisation ($\hat{\mu }=0.15$ ([95% CI: 0.004 to 0.29],z=2.01, p=0.04), but this result should be interpreted with caution as it is based on only two studies (Figure 70). According to the Q‐test, there was no significant amount of heterogeneity in the true outcomes (Q(1)=0.81, p=0.37, ${\hat{\tau }}^{2}=0.00$, $I^{2}=0.00$%). Both studies were assessed as high risk of bias. With only two studies and no heterogeneity, moderator analyses were not appropriate.

###### 6.3.1.12.17 Effects of interventions with engagement in implementation autonomy on timeliness of full childhood immunisation

**Figure 71:**
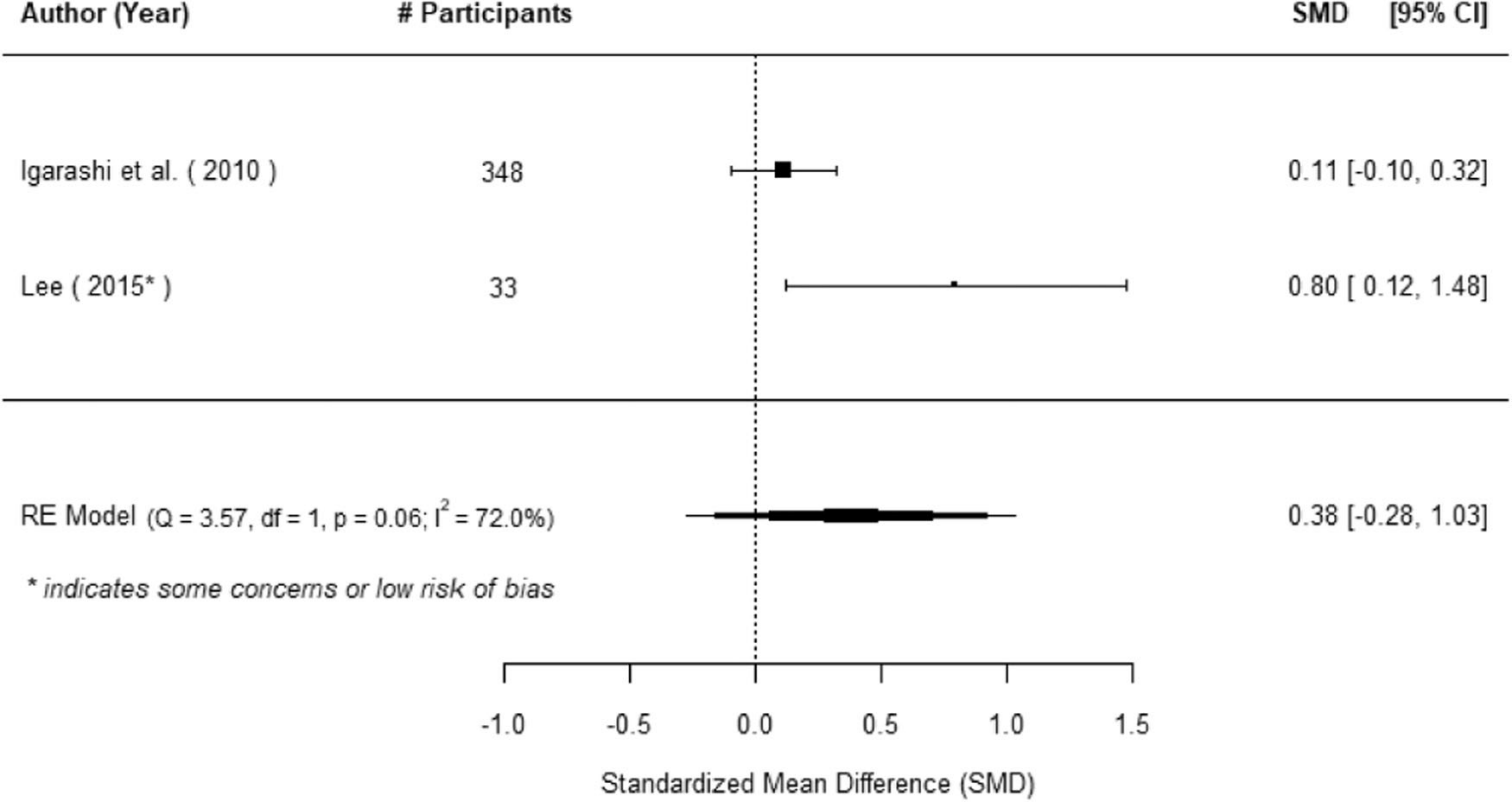
Forest plot showing the observed outcomes and the estimate of the random‐effects model for interventions with community engagement in implementation autonomy on DPT3 vaccination. DPT, diphtheria, pertussis tetanus.

Two studies using engagement in implementation autonomy reported on the timeliness of full childhood immunisation. There was no significant effect on timeliness of full childhood immunisation ($\hat{\mu }=0.38$ ([95% CI: −0.28 to 1.03],z=1.13, p=0.26), but this result should be interpreted with caution as it is based on only two studies (Figure 71).

According to the Q‐test, the true outcomes appear to be homogeneous (Q(1)=3.57, p=0.06, ${\hat{\tau }}^{2}=0.17$, $I^{2}=72.02$%). There was no indication of outliers in the context of this model, and with only two studies, we could not test for moderation. One study was assessed as having a high risk of bias (Igarashi et al., 2010) while the other was assessed as some concerns related to risk of bias (Lee, 2015).

###### 6.3.1.12.18 Effects of interventions with multiple engagement types on timeliness of full childhood immunisation

There were no studies using multiple engagement types that reported on the timeliness of full childhood immunisation.

##### 6.3.1.13 Childhood morbidity

**Figure 72:**
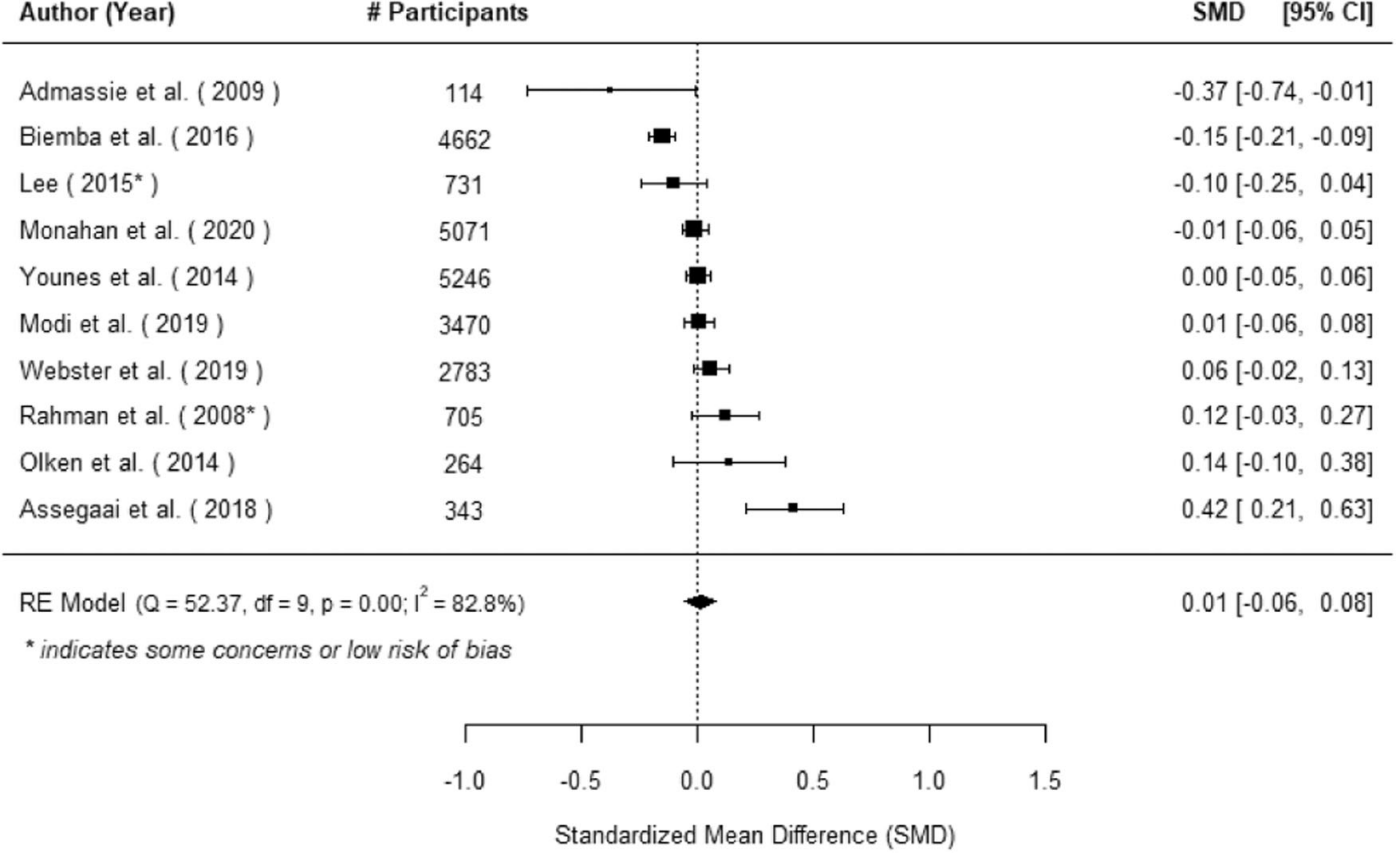
Forest plot showing the observed outcomes and the estimate of the random‐effects model for the impact of community engagement interventions on childhood morbidity.

We used reports of diarrhea (most typically in the past 2 weeks) as a proxy for childhood morbidity. In all cases, effects were reverse coded such that positive effects always indicate a benefit to the treated group. Thus, a positive effect here would be interpreted as a reduction in diarrhea among treated participants compared to control participants. A total of k=10 studies were included in the analysis. The estimated average outcome was $\hat{\mu }=0.01$ ([95% CI: −0.06 to 0.08],z=0.34, p=0.73), indicating no significant difference between intervention group and control group (Figure 72). According to the Q‐test, the true outcomes appear to be heterogeneous (Q(9)=52.37, p<0.01, ${\hat{\tau }}^{2}=0.01$, $I^{2}=82.82$%). When high risk of bias studies were removed, only two studies remained. The resulting estimated average outcome was identical ($\hat{\mu }=0.01$ [95% CI: −0.21 to 0.23]), and was still nonsignificant (z=0.09, p=0.93).

**Figure 73:**
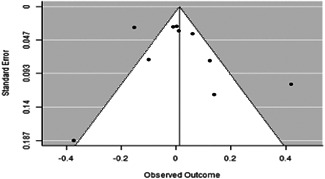
Funnel plot for studies examining the effect of community engagement interventions on childhood morbidity.

An examination of the studentized residuals revealed that one study (Assegaai et al., 2018) had a value larger than ±2.81 and may be a potential outlier in the context of this model. According to the Cook's distances, Assegaai and colleagues (2018) could also be considered to be overly influential. Indeed, sensitivity analyses leaving each study out indicated that removing Assegaai and colleagues (2018) would reduce the overall average effect ($\hat{\mu }$ = −0.02 (95% CI: −0.08 to 0.05), making the effect negative but still nonsignificant (z = −0.47, *p* = 0.64). A funnel plot of the estimates is shown in Figure 73. Neither the rank correlation nor the regression test indicated any funnel plot asymmetry (p=0.60 and p=0.49, respectively). We tested for sources of heterogeneity, but there were no significant moderators (see Supporting Information: Appendix I Table 3).

As a robustness check, we used robust variance analysis and included all dependent effects in the analysis, totalling 26 effects from the same 10 studies (*df* = 7.69). The overall average effect was identical and still nonsignificant ($\hat{\mu }=0.01$ [95% CI: −0.09 to 0.10], *p* = .86). Sensitivity analyses show the effect to be sensitive to all values of *ρ*.

###### 6.3.1.13.1 Effects of engagement as the intervention on childhood morbidity

**Figure 74:**
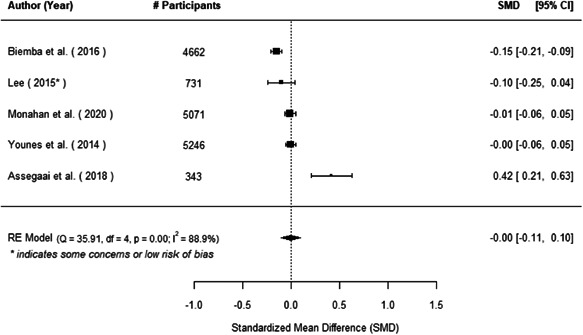
Forest plot showing the observed outcomes and the estimate of the random‐effects model for interventions with community engagement as the intervention on morbidity.

A total of k=5 studies were included in the analysis. The observed outcomes ranged from −0.15 to 0.42. The estimated average outcome based on the random‐effects model was $\hat{\mu }=-0.004$ (95% CI: −0.11 to 0.10). Therefore, the average outcome did not differ significantly from zero (z=−0.08, p=0.94), indicating no difference between the treatment and control groups on childhood morbidity (see Figure 74). According to the Q‐test, the true outcomes appear to be heterogeneous (Q(4)=35.91, p<0.01, ${\hat{\tau }}^{2}=0.01$, $I^{2}=88.86$%). An examination of the studentized residuals revealed that one study (Assegaai et al., 2018) had a value larger than ±2.58 and may be a potential outlier in the context of this model. Indeed, sensitivity analyses leaving each study out indicated that removing Assegaai and colleagues (2018) would reduce the overall average effect ($\hat{\mu }$ = −0.06 [95% CI: −0.14 to 0.02]). While the resulting effect would be negative, the effect would still be nonsignificant (*z* = −1.50, *p* = .13). According to the Cook's distances, none of the studies could be considered to be overly influential. There was only one study not assessed as high risk of bias, so we were unable to do a sensitivity analysis by study quality. Exposure to the intervention was a significant source of heterogeneity such that each additional month of exposure increased the positive impact on morbidity by 0.02 standard deviation units ($\hat{\mu }=0.02$ [95% CI: 0.01 to 0.03,p=0.002]). No other moderators were significant (see Supporting Information: Appendix I Table 3).

###### 6.3.1.13.2 Effects of engagement in the intervention design on childhood morbidity

**Figure 75:**
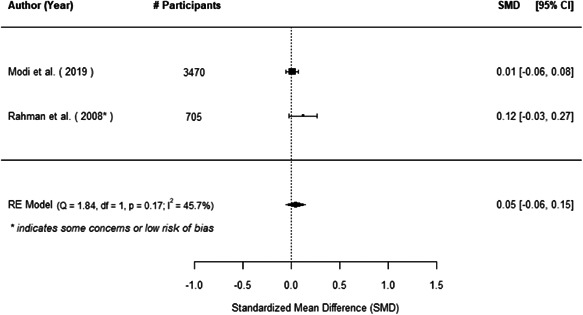
Forest plot showing the observed outcomes and the estimate of the random‐effects model for interventions with community engagement in the design on the intervention on childhood morbidity.

Only two studies reporting on childhood morbidity used interventions with community engagement in the intervention design. The estimated average outcome based on the random‐effects model was $\hat{\mu }=0.05$ (95% CI: −0.06 to 0.15). Therefore, the average outcome did not differ significantly from zero (z=0.88, p=0.38, see Figure 75). Given the small number of studies, this result should be interpreted with caution. According to the Q‐test, the true outcomes appear to be homogeneous (Q(1)=1.84, p=0.17, ${\hat{\tau }}^{2}=0.003$, $I^{2}=45.$71%). With only two studies, moderator analyses were not appropriate and tests of publication bias are not valid.

###### 6.3.1.13.3 Effects of interventions with implementation autonomy on childhood morbidity

Only one study (Webster et al., 2019) fell into this intervention/outcome category, thus we were unable to perform a statistical synthesis. This cluster RCT from Uganda found a null effect of their programme on morbidity (*g* = 0.06 [95% CI: −0.02 to 0.13]), but like most studies, it was assessed as having a high risk of bias.

###### 6.3.1.13.4 Effects of interventions with multiple engagement types on childhood morbidity

**Figure 76:**
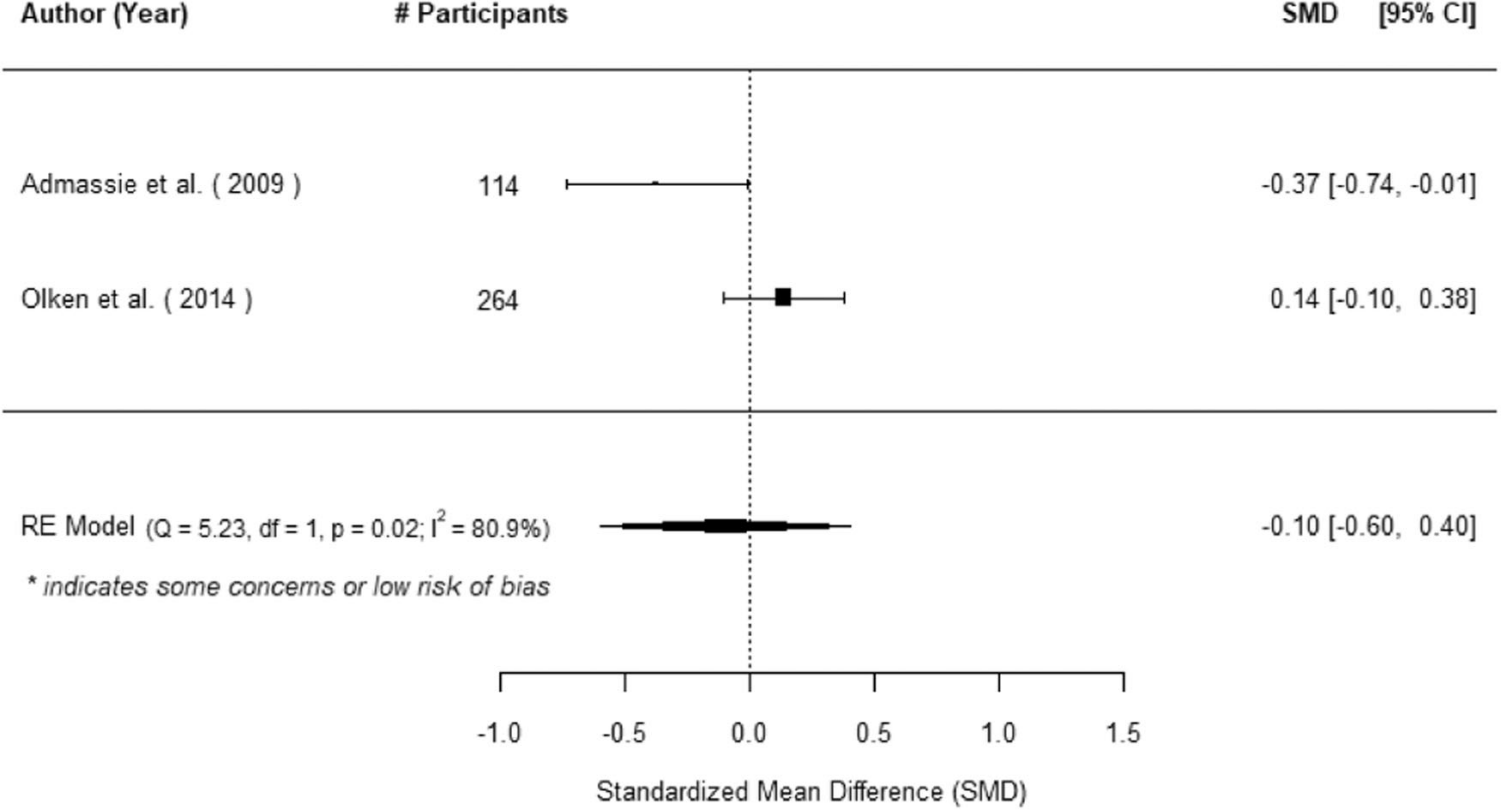
Forest plot showing the observed outcomes and the estimate of the random‐effects model for interventions with multiple engagement types on childhood morbidity.

Only two studies reporting on childhood morbidity used interventions with multiple engagement types. The estimated average outcome based on the random‐effects model was $\hat{\mu }=-0.10$ (95% CI: −0.60 to 0.40). Therefore, the average outcome did not differ significantly from zero, indicating no difference between the treated group compared to the control group (z=−0.38, p=0.70, see Figure 76). Given the small number of studies, this result should be interpreted with caution. According to the Q‐test, the true outcomes appear to be heterogeneous (Q(1)=5.23, p=0.02, ${\hat{\tau }}^{2}=0.11$, $I^{2}=80.88$%). With only two studies, moderator analyses were not appropriate and tests of publication bias are not valid.

##### 6.3.1.14 Childhood mortality

**Figure 77:**
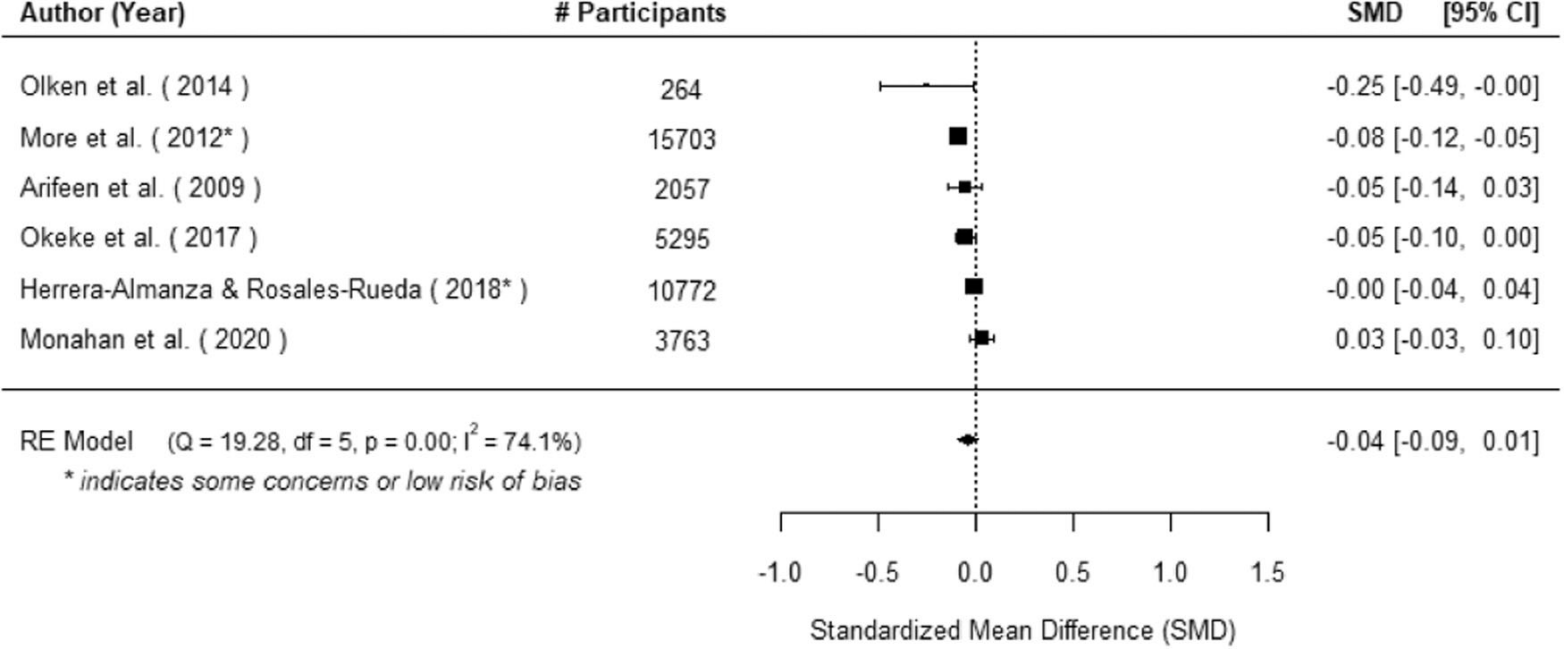
Forest plot showing the observed outcomes and the estimate of the random‐effects model for the impact of community engagement interventions on childhood morbidity.

We included total of k=6 studies in the analysis. As with morbidity, all outcomes are reverse coded so that positive numbers always reflect a benefit to the treated (in this case, a reduction in mortality for the intervention group). The estimated average outcome based on the random‐effects model was $\hat{\mu }=-0.04$ (95% CI: −0.09 to 0.01; see Figure 77). Therefore, the average outcome did not differ significantly from zero (z=−1.66, p=0.10), indicating no difference in mortality between the treatment and control groups. According to the Q‐test, the true outcomes appear to be heterogeneous (Q(5)=19.28, p<0.01, ${\hat{\tau }}^{2}=0.00$, $I^{2}=74.07$%). An examination of the studentized residuals revealed that none of the studies had a value larger than ±2.64 and hence there was no indication of outliers in the context of this model. According to the Cook's distances, none of the studies could be considered to be overly influential. When high risk of bias studies were removed, only two studies remained. The resulting estimated average outcome was identical ($\hat{\mu }=-0.04$ [95% CI: −0.12 to 0.04]), and was still nonsignificant (z=−1.07, p=0.29). We tested for sources of heterogeneity, and only publication year was significant such that each additional year increased the effect by 0.01 standard deviation units ($\hat{B}=0.01,p<0.001$ [95% CI: 0.01 to 0.02]). There were no other significant moderators (see Supporting Information: Appendix I Table 3) (Figure 77).

As a robustness check, we used robust variance analysis and included all dependent effects in the analysis, totalling 25 effects from the same 6 studies (*df* = 4.07). The overall average effect was identical and still nonsignificant ($\hat{\mu }=-0.04$ [95% CI: −0.09 to 0.01], *p* = .12). Sensitivity analyses show the effect to be sensitive to all values of *ρ*.

###### 6.3.1.14.1 Effects of engagement as the intervention on childhood mortality

**Figure 78:**
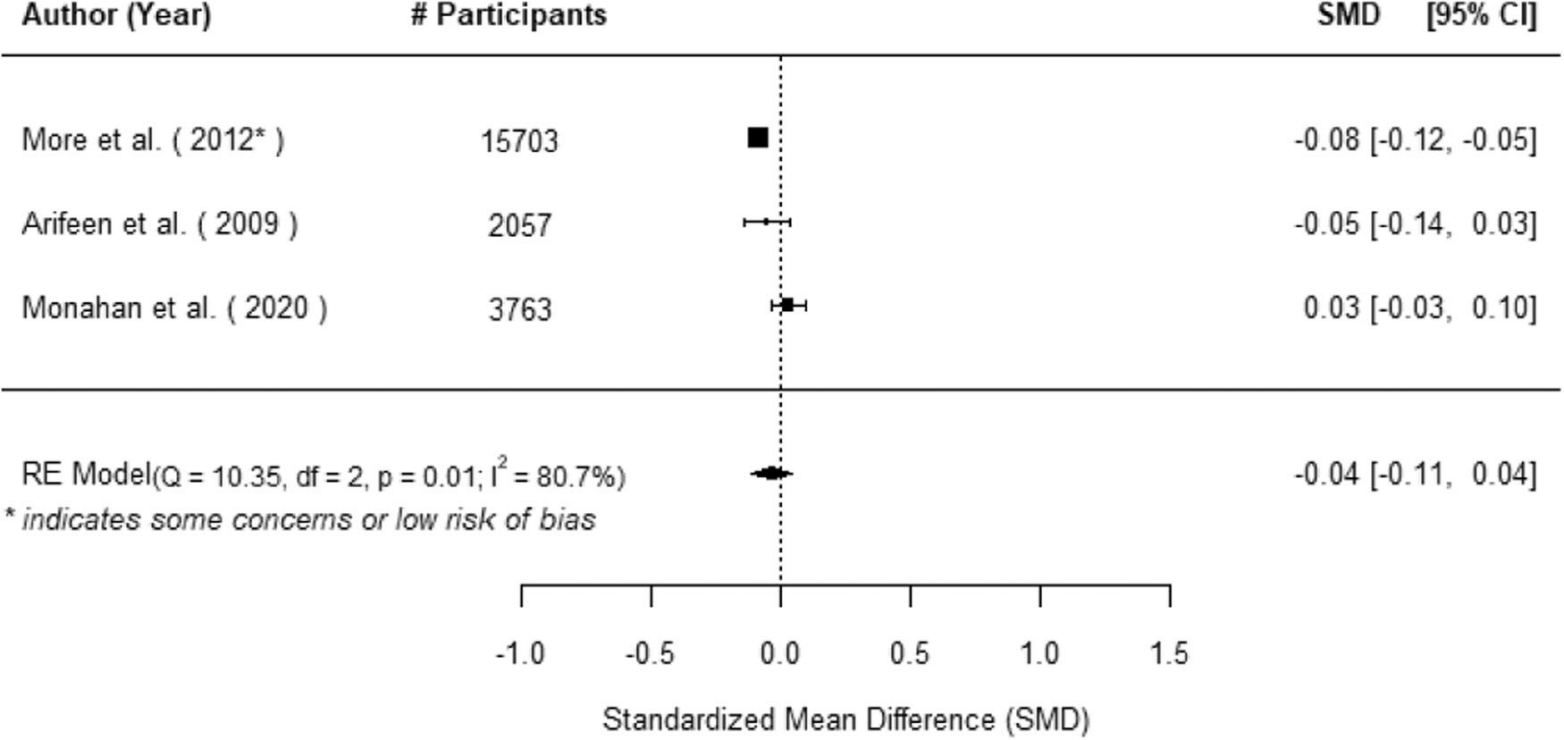
Forest plot showing the observed outcomes and the estimate of the random‐effects model for interventions with community engagement as the intervention on mortality.

We included a total of k=3 studies in the analysis. The estimated average outcome based on the random‐effects model was $\hat{\mu }=-0.04$ (95% CI: −0.11 to 0.04). Therefore, the average outcome did not differ significantly from zero (z=−0.94, p=0.34), indicating no difference between the treatment and control group (see Figure 78). According to the Q‐test, the true outcomes appear to be heterogeneous (Q(2)=10.35, p<0.01, ${\hat{\tau }}^{2}=0.00$, $I^{2}=80.68$%). An examination of the studentized residuals revealed that one study (Mohanan et al., 2020) had a value larger than ±2.39 and may be a potential outlier in the context of this model. Indeed, sensitivity analyses leaving each study out indicated that removing Mohanan and colleagues (2020) would reduce the overall average effect ($\hat{\mu }$ = 0.01 [95% CI: −0.14 to 0.16]), but the effect would still be positive and nonsignificant (z = 0.10, p = 0.92). According to the Cook's distances, none of the studies could be considered to be overly influential. With only three studies contributing effects, we were unable to test for potential sources of heterogeneity.

###### 6.3.1.14.2 Effects of engagement in the intervention design on childhood mortality

There were no studies using engagement in the intervention design that reported on child mortality.

###### 6.3.1.14.3 Effects of interventions with implementation autonomy on childhood mortality

There were no studies using community engagement in implementation autonomy that reported on child mortality.

###### 6.3.1.14.4 Effects of interventions with multiple engagement types on childhood mortality

**Figure 79:**
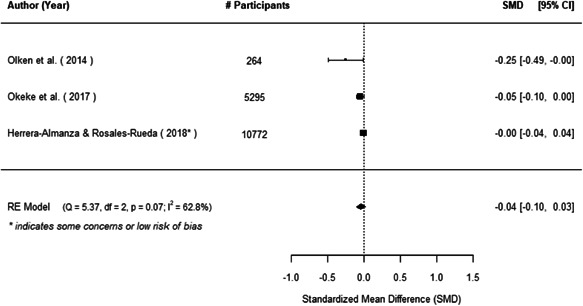
Forest plot showing the observed outcomes and the estimate of the random‐effects model for interventions with multiple engagement types on childhood morbidity.

We included a total of k=3 studies in the analysis. The observed outcomes ranged from −0.25 to −0.002. The estimated average outcome based on the random‐effects model was $\hat{\mu }=-0.04$ (95% CI: −0.10 to 0.03, see Figure 79). Therefore, the average outcome did not differ significantly from zero (z=−1.15, p=0.25), indicating no difference between the intervention participants and control participants on child mortality. The Q‐test for heterogeneity was not significant, but some heterogeneity may still be present in the true outcomes (Q(2)=5.37, p=0.07, ${\hat{\tau }}^{2}=0.0018$, $I^{2}=62.75$%). An examination of the studentized residuals revealed that none of the studies had a value larger than ±2.39 and hence there was no indication of outliers in the context of this model. According to the Cook's distances, none of the studies could be considered to be overly influential. With only three studies, moderator analyses were not appropriate and tests of publication bias are not valid (Figure 79).

##### 6.3.1.15 Vaccination dropouts

**Figure 80:**
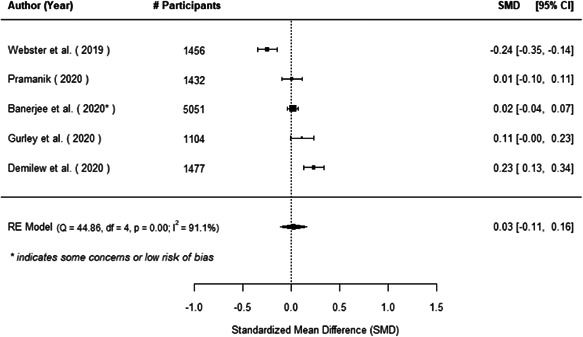
Forest plot showing the observed outcomes and the estimate of the random‐effects model for the impact of community engagement interventions on childhood morbidity.

We included a total of k=5 studies in the analysis. The estimated average outcome based on the random‐effects model was $\hat{\mu }=0.03$ ([95% CI: −0.11 to 0.16],z=0.36, p=0.72), indicating no significant difference between the intervention group and the control group (see Figure 80). According to the Q‐test, the true outcomes appear to be heterogeneous (Q(4)=44.86, p<0.001, ${\hat{\tau }}^{2}=0.02$, $I^{2}=91.08$%). An examination of the studentized residuals revealed that one study (Webster et al., 2019) had a value larger than ±2.58 and may be a potential outlier in the context of this model. Indeed, sensitivity analyses indicated that removing Webster and colleagues (2019) would increase the average effect to $\hat{\mu }=0.09$ (95% CI: −0.01 to 0.19), but the average outcome still did not differ significantly from zero (z=1.70p=0.09). According to the Cook's distances, none of the studies could be considered to be overly influential. With only one study assessed as having a low risk of bias (Banerjee et al., 2020) and the remaining studies at high risk of bias, we were unable to conduct sensitivity analysis by study quality. Only publication year was a significant predictor such that more recently published studies had higher average effects than older studies. Specifically, each additional year increased the average effect by 0.33 standard deviation units (β=0.33 [95% CI: 0.10 to 0.57], p=0.01).

This group of studies was not sufficiently powered for a robustness check using robust variance analysis (*df *= 3.98).

###### 6.3.1.15.1 Effects of engagement as the intervention on childhood vaccination dropouts

**Figure 81:**
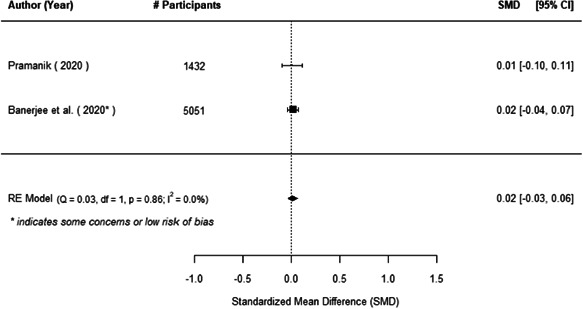
Forest plot showing the observed outcomes and the estimate of the random‐effects model for interventions with community engagement as the intervention on vaccination dropouts.

Only two studies reporting on vaccination dropouts used interventions with community engagement as the intervention. The estimated average outcome based on the random‐effects model was $\hat{\mu }=0.02$ ([95% CI: −0.03 to 0.06],z=0.63, p=0.53), indicating no significant difference between the intervention group and the control group on vaccination dropouts (see Figure 81). Given the small number of studies, this result should be interpreted with caution. According to the Q‐test, the true outcomes appear to be homogeneous (Q(1)=0.03, p=0.86, ${\hat{\tau }}^{2}=0.00$, $I^{2}=0.00$%). With only two studies and no heterogeneity, moderator analyses were not appropriate and tests of publication bias are not valid.

###### 6.3.1.15.2 Effects of engagement in the intervention design on childhood vaccination dropouts

There were no studies using engagement in the intervention design that reported on vaccination dropouts.

###### 6.3.1.15.3 Effects of engagement in implementation autonomy on childhood vaccination dropouts

Only one study reporting on childhood morbidity/mortality used an intervention with engagement in the implementation (Webster et al., 2019), thus we were unable to perform a statistical synthesis. This cluster RCT from Uganda found a small but significant negative impact of their programme on full childhood immunisation (*g* = −0.24 [95% CI: −0.35 to −0.14]), but like most studies, it was assessed as having a high risk of bias.

###### 6.3.1.15.4 Effects of interventions with multiple engagement types on childhood vaccination dropouts

*Dropouts*

**Figure 82:**
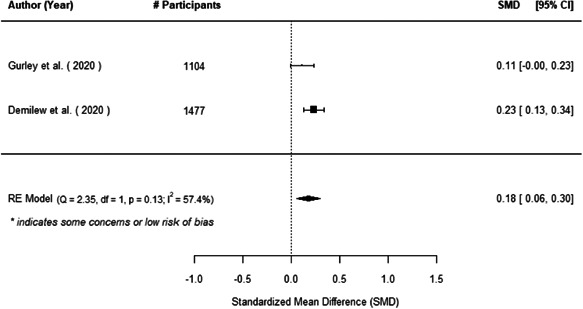
Forest plot showing the observed outcomes and the estimate of the random‐effects model for interventions with multiple engagement types on vaccination dropouts.

Only two studies reporting on immunisation dropouts used interventions with multiple engagement types. All effects were computed such that a positive effect reflects a benefit to the treated group (e.g., a reduction in dropouts). The estimated average outcome based on the random‐effects model was $\hat{\mu }=0.18$ (95% CI: 0.06 to 0.30). Therefore, the average outcome differed significantly from zero, indicating a small but significant benefit to the treated group compared to the control group (z=2.92, p=0.004, see Figure 82). Given the small number of studies, this result should be interpreted with caution. According to the Q‐test, the true outcomes appear to be homogeneous (Q(1)=2.35, p=0.13, ${\hat{\tau }}^{2}=0.004$, $I^{2}=57.38$%). With only two studies, moderator analyses were not appropriate and tests of publication bias are not valid.

##### 6.3.1.16 Immunisation knowledge

**Figure 83:**
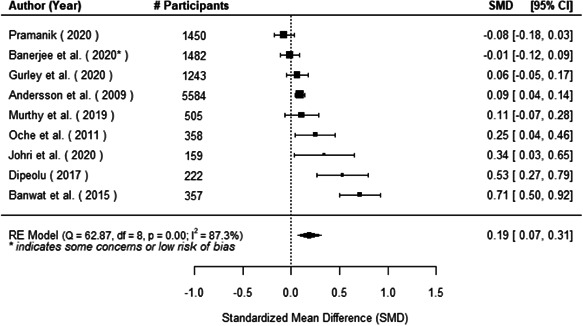
Forest plot showing the observed outcomes and the estimate of the random‐effects model for the impact of community engagement interventions on childhood morbidity.

We included a total of k=9 studies in the analysis. The estimated average outcome based on the random‐effects model was $\hat{\mu }=0.19$ (95% CI: 0.07 to 0.31). Therefore, the average outcome differed significantly from zero (z=3.02, p<0.01), indicating a significant benefit to the treatment group compared to the control group (Figure 83). According to the Q‐test, the true outcomes appear to be heterogeneous (Q(8)=62.87, p<0.01, ${\hat{\tau }}^{2}=0.03$, $I^{2}=87.28$%). An examination of the studentized residuals revealed that one study (Banwat, 2015) had a value larger than ±2.77 and may be a potential outlier in the context of this model. Indeed, sensitivity analyses leaving each study out indicated that removing Banwat and colleague (2015) would reduce the overall average effect ($\hat{\mu }\;$= 0.11 (95% CI: −0.02 to 0.20), but the effect would still be positive and significant (*z* = 2.33, *p* = .02). According to the Cook's distances, none of the studies could be considered to be overly influential.

We examined potential sources of heterogeneity, and study design was a significant predictor of immunisation knowledge such that studies using quasi‐experimental designs had larger average effects than RCT designs by 0.44 standard deviation units ($\hat{\beta }=0.44$ [95% CI: 0.23 to 0.64], p < 0.001). Region was also a significant predictor, but region was perfectly confounded with study design (e.g., all studies from South Asia were RCTs, while all studies from Sub‐Saharan Africa were all quasi‐experimental designs), so the effects were larger in Sub‐Saharan Africa than from South Asia by the same.44 standard deviation units (β=0.44 [95% CI: 0.23 to 0.64], p < 0.001). No other moderators were significant (see Supporting Information: Appendix I Table 33).

As a robustness check, we used robust variance analysis and included all dependent effects in the analysis, totalling 13 effects from the same 9 studies (*df* = 7.69). The overall average effect was slightly smaller and became nonsignificant ($\hat{\mu }=0.17$ [95% CI: −0.02 to 0.37], *p* = .07). Sensitivity analyses show the effect to be sensitive to all values of *ρ*.

###### 6.3.1.16.1 Effects of engagement as the intervention on immunisation knowledge

**Figure 84:**
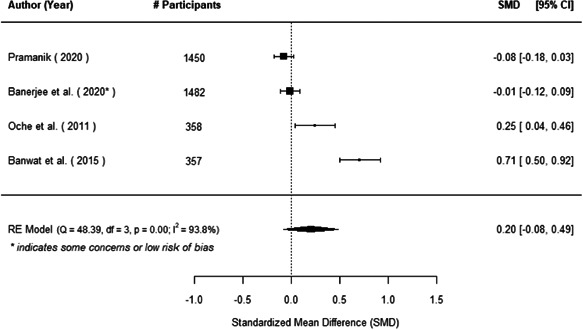
Forest plot showing the observed outcomes and the estimate of the random‐effects model for interventions with community engagement as the intervention on immunisation attitudes

We included a total of k=4 studies in the analysis. The estimated average outcome based on the random‐effects model was $\hat{\mu }=0.20$ (95% CI: −0.08 to 0.49). Therefore, the average outcome did not differ significantly from zero (z=1.41, p=0.16), indicating no difference between the treatment and control groups (Figure 84). According to the Q‐test, the true outcomes appear to be heterogeneous (Q(3)=48.39, p<0.01, ${\hat{\tau }}^{2}=0.08$, $I^{2}=93.80$%). An examination of the studentized residuals revealed that one study (Banwat, 2015) had a value larger than ±2.50 and may be a potential outlier in the context of this model. Indeed, sensitivity analyses leaving each study out indicated that removing Banwat and colleague (2015) would reduce the overall average effect ($\hat{\mu }$ = 0.02 (95% CI: −0.12 to 0.17), but the effect would still be positive and nonsignificant (*z* = 0.35, *p* = .73). According to the Cook's distances, none of the studies could be considered to be overly influential.

We examined potential sources of heterogeneity, and study design was a significant predictor of immunisation knowledge such that studies using quasi‐experimental designs had larger average effects than RCT designs by 0.52 standard deviation units ($\hat{\beta }=0.52$ [95% CI: 0.11 to 0.94], p = 0.01). Region was also a significant predictor, but region was perfectly confounded with study design (e.g., all studies from South Asia were RCTs, while all studies from Sub‐Saharan Africa were all quasi‐experimental designs), so the effects were larger in Sub‐Saharan Africa than from South Asia by the same.52 standard deviation units (β=0.52 [95% CI: 0.11 to 0.94], p = 0.01). No other moderators were significant (see Table 3 in Supporting Information: Appendix I).

###### 6.3.1.16.2 Effects of engagement in the intervention design on immunisation knowledge

**Figure 85:**
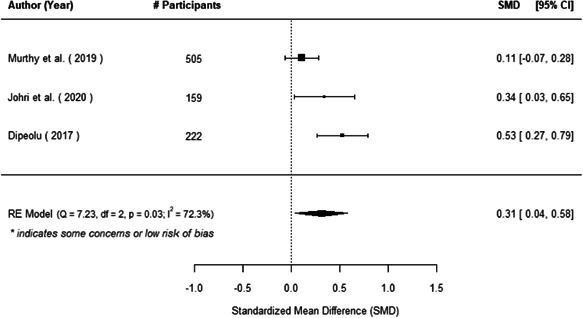
Forest plot showing the observed outcomes and the estimate of the random‐effects model for interventions with community engagement in the design on the intervention on childhood morbidity.

A total of k=3 studies were included in the analysis. The observed outcomes ranged from 0.11 to 0.53. The estimated average outcome based on the random‐effects model was $\hat{\mu }=0.31$ (95% CI: 0.04 to 0.58). Therefore, the average outcome differed significantly from zero (z=2.26, p=0.02), indicating a moderate significant benefit to the intervention group compared to the control group (see Figure 85). According to the Q‐test, the true outcomes appear to be heterogeneous (Q(2)=7.23, p=0.03, ${\hat{\tau }}^{2}=0.04$, $I^{2}=72.34$%). An examination of the studentized residuals revealed that one study (Murthy et al., 2019) had a value larger than ±2.39 and may be a potential outlier in the context of this model. Indeed, sensitivity analyses leaving each study out indicated that removing Murthy and colleagues (2019) would reduce the overall average effect ($\hat{\mu }$ = 0.19 (95% CI: −0.03 to 0.41), and the effect would still be positive but would be nonsignificant (z= 1.67, p= 0.09). According to the Cook's distances, none of the studies could be considered to be overly influential. With only three studies, we were unable to test for sources of heterogeneity.

###### 6.3.1.16.3 Effects of engagement in implementation autonomy on immunisation knowledge

There were no studies reporting on immunisation knowledge that used community engagement in implementation autonomy.

###### 6.3.1.16.4 Effects of interventions with multiple engagement types on immunisation knowledge

**Figure 86:**
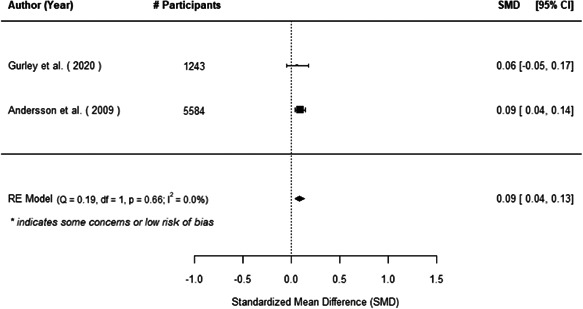
Forest plot showing the observed outcomes and the estimate of the random‐effects model for interventions with multiple engagement types on immunisation knowledge.

Only two studies reporting on immunisation knowledge used interventions with multiple engagement types. The estimated average outcome based on the random‐effects model was $\hat{\mu }=0.09$ (95% CI: 0.04 to 0.13). Therefore, the average outcome differed significantly from zero, indicating a very small but significant benefit to the treated group compared to the control group (z=3.55, p<0.001), see Figure 86. Given the small number of studies, this result should be interpreted with caution. According to the Q‐test, the true outcomes appear to be homogeneous (Q(1)=0.19, p=0.66, ${\hat{\tau }}^{2}=0.00$, $I^{2}=0.00$%). With only two studies, moderator analyses were not appropriate and tests of publication bias are not valid. Both studies were assessed as high risk of bias.

##### 6.3.1.17 Immunisation attitudes

**Figure 87:**
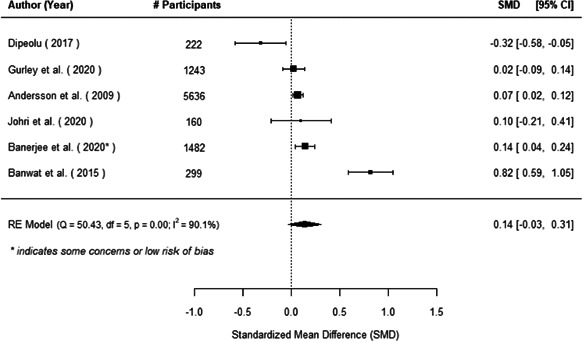
Funnel plot for studies examining the effect of community engagement interventions on immunisation attitudes.

We included a total of k=6 studies in the analysis. The estimated average outcome was $\hat{\mu }=0.14$ ([95% CI: −0.03 to 0.31],z=1.60, p=0.11), indicating no significant different between the intervention and control groups (see Figure 87). According to the Q‐test, the true outcomes appear to be heterogeneous (Q(5)=50.43, p<0.01, ${\hat{\tau }}^{2}=0.04$, $I^{2}=90.09$%). An examination of the studentized residuals revealed that one study (Banwat, 2015) had a value larger than ±2.64 and may be a potential outlier in the context of this model. Indeed, sensitivity analyses leaving each study out indicated that removing Banwat and colleague (2015) would reduce the overall average effect ($\hat{\mu }$ = 0.04 (95% CI: −0.05 to 0.14), but the effect would still be positive and nonsignificant (z = 0.92, *p* = .36). According to the Cook's distances, none of the studies could be considered to be overly influential. With only one study not assessed as high risk of bias, we were unable to conduct a sensitivity analysis by study quality. We tested for sources of heterogeneity, but of the moderators we were able to test, none were significant (see Supporting Information: Appendix I Table 3).

There were no dependent effects, so we did not complete a robustness check using RVE for this analysis.

###### 6.3.1.17.1 Effects of engagement as the intervention on immunisation attitudes

**Figure 88:**
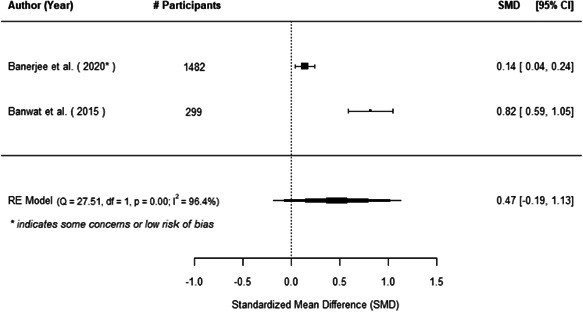
Forest plot showing the observed outcomes and the estimate of the random‐effects model for interventions with community engagement as the intervention on immunisation attitudes.

Only two studies reporting on immunisation attitudes used interventions with community engagement as the intervention. The estimated average outcome based on the random‐effects model was $\hat{\mu }=0.47$ ([95% CI: −0.19 to 1.13],z=1.40, p=0.16), indicating no significant difference between the intervention group and the control group (see Figure 88). Given the small number of studies, this result should be interpreted with caution. According to the Q‐test, the true outcomes appear to be heterogeneous (Q(1)=27.51, p<0.001, ${\hat{\tau }}^{2}=0.22$, $I^{2}=96.36$%; With only two studies and no heterogeneity, moderator analyses were not appropriate and tests of publication bias are not valid.

###### 6.3.1.17.2 Effects of engagement in the intervention design on immunisation attitudes

**Figure 89:**
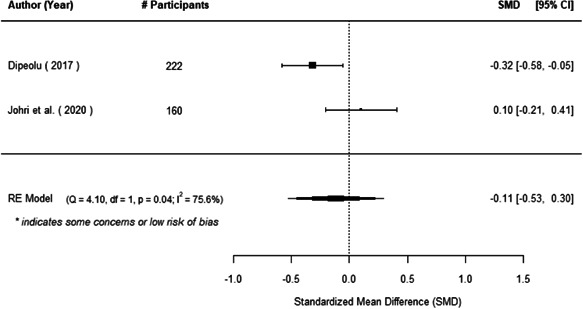
Forest plot showing the observed outcomes and the estimate of the random‐effects model for interventions with community engagement in the design on the intervention on immunisation attitudes.

Only two studies reporting on immunisation attitudes used interventions with community engagement in the intervention design. The estimated average outcome based on the random‐effects model was $\hat{\mu }=-0.11$ (95% CI: −0.53 to 0.30). Therefore, the average outcome did not differ significantly from zero (z=−0.55, p=0.58, see Figure 89). Given the small number of studies, this result should be interpreted with caution. According to the Q‐test, the true outcomes appear to be heterogeneous (Q(1)=4.10, p=0.04, ${\hat{\tau }}^{2}=0.07$, $I^{2}=75.61$%). With only two studies, moderator analyses were not appropriate and tests of publication bias are not valid.

###### 6.3.1.17.3 Effects of engagement in implementation autonomy on immunisation attitudes

There were no studies reporting on immunisation attitudes that used interventions with community engagement in implementation autonomy.

###### 6.3.1.17.4 Effects of interventions with multiple engagement types on immunisation attitudes

**Figure 90:**
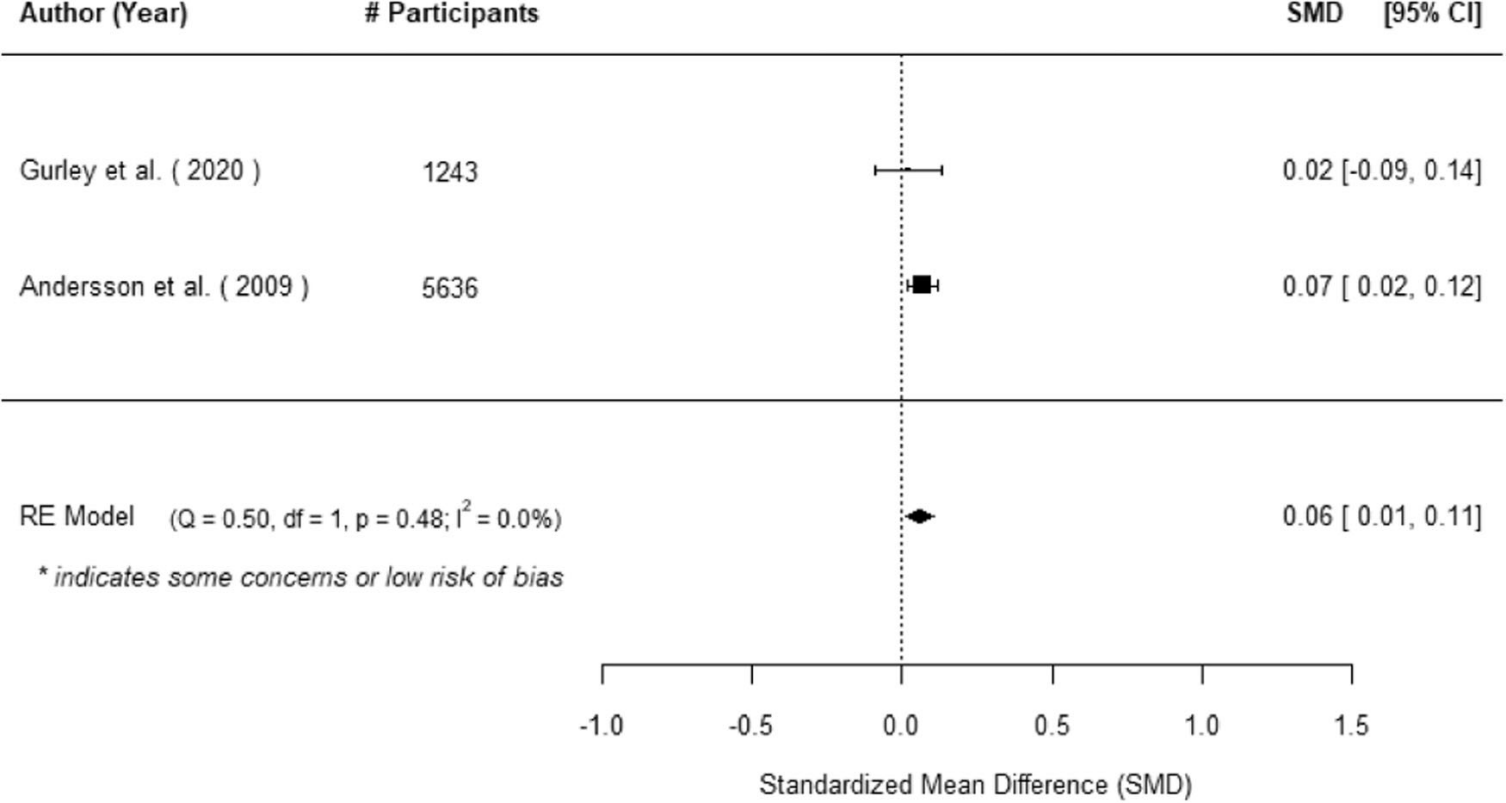
Forest plot showing the observed outcomes and the estimate of the random‐effects model for interventions with multiple engagement types on immunisation attitudes.

Only two studies reporting on immunisation attitudes used interventions with multiple engagement types. The estimated average outcome based on the random‐effects model was $\hat{\mu }=0.06$ (95% CI: 0.01 to 0.11). Therefore, the average outcome differed significantly from zero, indicating a very small but significant benefit to the treated group compared to the control group (z=2.50, p=0.01, see Figure 90). Given the small number of studies, this result should be interpreted with caution. According to the Q‐test, the true outcomes appear to be homogeneous (Q(1)=0.50, p=0.48, ${\hat{\tau }}^{2}=0.00$, $I^{2}=0.00$%). With only two studies, moderator analyses were not appropriate and tests of publication bias are not valid.

##### 6.3.1.18 Vaccination card availability/retention

**Figure 91:**
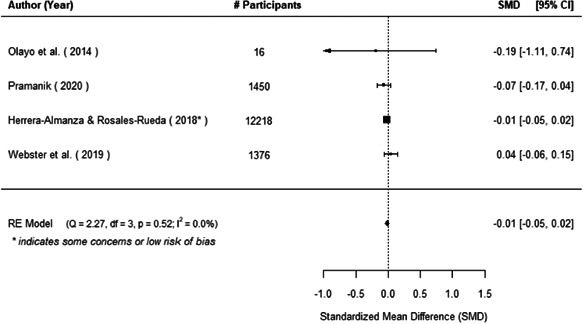
Forest plot showing the observed outcomes and the estimate of the random‐effects model for the impact of community engagement interventions on vaccination card availability.

We included total of k=4 studies in the analysis. The estimated average outcome was $\hat{\mu }=-0.01$ (95% CI: −0.05 to 0.02). The average outcome did not differ significantly from zero (z=−0.84, p=0.40), indicating no difference between the intervention group and the control group on vaccination card availability (see Figure 91). According to the Q‐test, there was no significant amount of heterogeneity in the true outcomes (Q(3)=2.27, p=0.52, ${\hat{\tau }}^{2}=0.00$, $I^{2}=0.00$%). One study (Herrera‐Almanza & Rosales‐Rueda, 2018) had a relatively large weight compared to the rest of the studies (i.e., weight≥3/k, so a weight at least three times as large as having equal weights across studies). However, this was also the only study assessed as having a low risk of bias. The three other studies in the analysis were assessed as high risk of bias. An examination of the studentized residuals revealed that none of the studies had a value larger than ±2.50 and hence there was no indication of outliers in the context of this model. According to the Cook's distances, none of the studies could be considered to be overly influential. With no heterogeneity present, we did not test for moderation.

There were no dependent effects, so we did not complete a robustness check using RVE for this analysis.

###### 6.3.1.18.1 Effects of engagement as the intervention on vaccination card availability/retention

Only one study (Pramanik et al., 2020) fell into this intervention/outcome category, thus we were unable to perform a statistical synthesis. This cluster RCT from India found a null effect of their programme on vaccination card retention/availability (*g* = −0.07 [95% CI: −0.17 to 0.04]), but like most studies, it was assessed as having a high risk of bias.

###### 6.3.1.18.2 Effects of engagement in the intervention design on vaccination card availability/retention

No studies reporting on vaccination card retention/availability used engagement in the intervention design.

###### 6.3.1.18.3 Effects of engagement in implementation autonomy on vaccination card availability/retention

Only one study reporting on vaccination card availability/retention used interventions with engagement in the implementation (Webster, 2019), thus we were unable to perform a statistical synthesis. This cluster RCT from Uganda found a very small positive but nonsignificant effect of their programme on full childhood immunisation (*g* = 0.04 [95% CI: −0.06 to −0.15]), but like most studies, it was assessed as having a high risk of bias.

###### 6.3.1.18.4 Effects of interventions with multiple engagement types on vaccination card availability/retention

**Figure 92:**
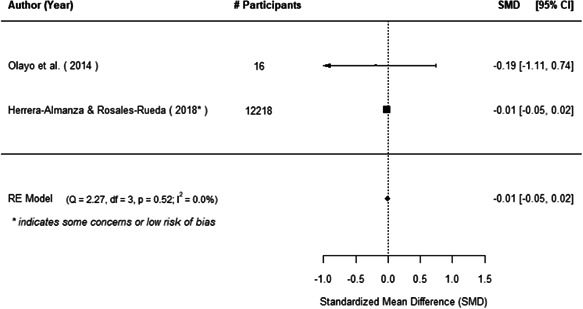
Forest plot showing the observed outcomes and the estimate of the random‐effects model for interventions with multiple engagement types on vaccination card availability

Only two studies reporting on vaccination card availability/retention used interventions with multiple engagement types. The estimated average outcome based on the random‐effects model was $\hat{\mu }=-0.01$ (95% CI: −0.05 to 0.02). Therefore, the average outcome did not differ significantly from zero (z=−0.78, p=0.43, see Figure 92). Given the small number of studies, this result should be interpreted with caution. According to the Q‐test, the true outcomes appear to be homogeneous (Q(5)=0.13, p=0.43, ${\hat{\tau }}^{2}=0.00$, $I^{2}=0.00$%; With only two studies and no heterogeneity, moderator analyses were not appropriate and tests of publication bias are not valid.

##### 6.3.1.19 Experience and satisfaction with health services

**Figure 93:**
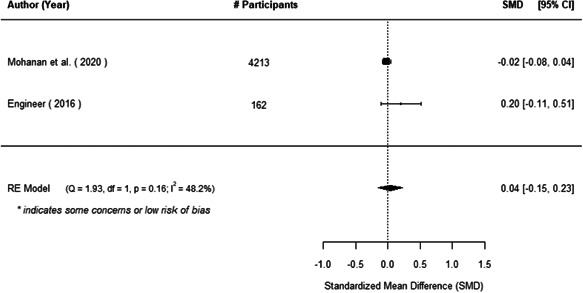
Forest plot showing the observed outcomes and the estimate of the random‐effects model for community engagement interventions on experience and satisfaction with health services.

Only two studies using community engagement interventions reported on experience and satisfaction with health services. The estimated average outcome based on the random‐effects model was $\hat{\mu }=0.04$ (95% CI: −0.15to 0.23). Therefore, the average outcome did not differ significantly from zero (z=0.38, p=0.70, see Figure 93). Given the small number of studies, this result should be interpreted with caution. According to the Q‐test, the true outcomes appear to be homogeneous (Q(1)=1.93, p=0.16, ${\hat{\tau }}^{2}=0.01$, $I^{2}=48.22$%; With only two studies and no heterogeneity, moderator analyses were not appropriate and tests of publication bias are not valid.

###### 6.3.1.19.1 Effects of engagement as the intervention on experience and satisfaction with health services

Only one study (Mohanan et al., 2020) fell into this intervention/outcome category, thus we were unable to perform a statistical synthesis. This cluster RCT from India did not find a significant effect of their programme on satisfaction with health services (*g* = −0.02 [95% CI: −0.08 to 0.04]), but like most studies, it was assessed as having a high risk of bias.

###### 6.3.1.19.2 Effects of engagement in the intervention design on experience and satisfaction with health services

Only one study (Engineer et al., 2016) fell into this intervention/outcome category, thus we were unable to perform a statistical synthesis. This cluster RCT from Afghanistan did not find a significant effect of their programme on satisfaction with health services (*g* = 0.20 [95% CI: −0.11 to 0.51]), but as with the other study reporting on this outcome, it was assessed as having a high risk of bias.

###### 6.3.1.19.3 Effects of engagement in implementation autonomy on experience and satisfaction with health services

No studies reporting on experience and satisfaction with health services used engagement in implementation autonomy.

###### 6.3.1.19.4 Effects of interventions with multiple engagement types on experience and satisfaction with health services

No studies reporting on experience and satisfaction with health services used multiple engagement types.

##### 6.3.1.20 Formal health worker's motivation, capacity and performance

**Figure 94:**
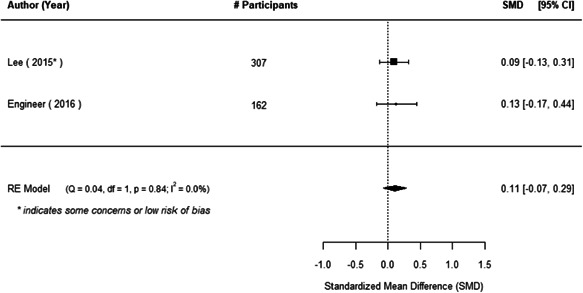
Forest plot showing the observed outcomes and the estimate of the random‐effects model for community engagement interventions on formal health worker's motivation, capacity and performance.

Only two studies using community engagement interventions reported on formal health worker's motivation, capacity and performance. The estimated average outcome based on the random‐effects model was $\hat{\mu }=0.11$ [95% CI: −0.07 to 0.29]. Therefore, the average outcome did not differ significantly from zero (z=0.38, p=0.70, see Figure 94). Given the small number of studies, this result should be interpreted with caution. According to the Q‐test, the true outcomes appear to be homogeneous (Q(1)=0.04, p=0.84, ${\hat{\tau }}^{2}=0.00$, $I^{2}=0.00$%; With only two studies and no heterogeneity, moderator analyses were not appropriate and tests of publication bias are not valid.

###### 6.3.1.20.1 Effects of engagement as the intervention on formal health worker's motivation, capacity and performance

Only one study (Lee, 2015) fell into this intervention/outcome category, thus we were unable to perform a statistical synthesis. This cluster RCT from Zambia did not find a significant effect of their programme on formal health worker's motivation, capacity, and performance (*g* = 0.09 [95% CI: −0.13 to 0.31]). This study was assessed as was assessed as having some concerns related to risk of bias.

###### 6.3.1.20.2 Effects of engagement in the intervention design on formal health worker's motivation, capacity and performance

Only one study (Engineer et al., 2016) fell into this intervention/outcome category, thus we were unable to perform a statistical synthesis. This cluster RCT from Afghanistan did not find a significant effect of their programme on satisfaction with health services (*g* = 0.13 [95% CI: −0.17 to 0.44]), but as with the other study reporting on this outcome, it was assessed as having a high risk of bias.

###### 6.3.1.20.3 Effects of engagement in implementation autonomy on formal health worker's motivation, capacity and performance

No studies reporting on formal health worker's motivation, capacity and performance used engagement in implementation autonomy.

###### 6.3.1.20.4 Effects of interventions with multiple engagement types on formal health worker's motivation, capacity and performance

No studies reporting on formal health worker's motivation, capacity and performance used multiple engagement types.

The results of all quantitative analyses, translated into percentile increase in the intervention group compared to the control group, is presented in Table 7 below (Table 9).

**Table 9:** Summary of quantitative effects translated into percentile increase in the intervention group compared to the control group

Outcome (intervention subgroup)	SMD	Estimated percentile change compared to control group
*Full immunisation*	0.14**	5.6%
Full immunisation (EAI)	0.08**	3.2%
Full immunisation (EID)	0.10*	4%
Full immunisation (EII)	0.23	9.1%
Full immunisation (MET)	0.22	8.7%
*Partial immunisation*	0.23**	9.1%
Partial immunisation (EAI)	0.31	12.2%
Partial immunisation (EID)	0.14*	5.6%
Partial immunisation (EII)		
Partial immunisation (MET)	0.28	11%
*Measles*	0.07**	2.8%
Measles (EAI)	0.1***	4%
Measles (EID)	0.11*	4.4%
Measles (EII)	0.03	1.2%
Measles (MET)	0.03	1.2%
*BCG*	0.06*	2.4%
BCG (EAI)	0.02***	0.8%
BCG (EID)	0.02	0.8%
BCG (EII)	0.02	0.8%
BCG (MET)	0.22	8.7%
*DPT1*	0.04	1.6%
DPT1 (EAI)	0.1	4%
DPT1 (EID)	0.03	1.2%
DPT1 (EII)		
DPT1 (MET)	−0.17**	−6.7%
*DPT2*	0.07*	2.8%
DPT2 (EAI)		
DPT2 (EID)	0.05	2%
DPT2 (EII)		
DPT2 (MET)		
*DPT3*	0.1***	4%
DPT3 (EAI)	0.09**	3.6%
DPT3 (EID)	0.04	1.6%
DPT3 (EII)	0.11	4.4%
DPT3 (MET)	0.2**	7.9%
*OPV0*	0.1	4%
OPV0 (EAI)		
OPV0 (EID)	0.01	0.4%
OPV0 (EII)		
OPV0 (MET)		
*OPV1*	0.08*	3.2%
OPV1 (EAI)		
OPV1 (EID)	0.08	3.2%
OPV1 (EII)		
OPV1 (MET)	0.22	8.7%
*OPV2*	0.24**	9.5%
OPV2 (EAI)		
OPV2 (EID)	0.23	9.1%
OPV2 (EII)		
OPV2 (MET)	0.34***	13.3%
*OPV3*	0.24**	9.5%
OPV3 (EAI)	0.16	6.4%
OPV3 (EID)		
OPV3 (EII)	0.03	1.2%
OPV3 (MET)	0.48	18.4%
*Timeliness of DPT3*	0.09**	3.6%
Timeliness of DPT3 (EAI)		
Timeliness of DPT3 (EID)	0.12**	4.8%
Timeliness of DPT3 (EII)	0.04	1.6%
Timeliness of DPT3 (MET)		
*Timeliness of measles*	0.23***	9.1%
Timeliness of measles (EAI)		
Timeliness of measles (EID)		
Timeliness of measles (EII)		
Timeliness of measles (MET)		
*Timeliness of full immunisation*	0.15***	6%
Timeliness of full immunisation (EAI)		
Timeliness of full immunisation (EID)	0.15*	6%
Timeliness of full immunisation (EII)	0.38	14.8%
Timeliness of full immunisation (MET)		
*Dropouts*	0.03	1.2%
Dropouts (EAI)	0.02	0.8%
Dropouts (EID)		
Dropouts (EII)		
Dropouts (MET)	0.18**	7.1%
*Morbidity*	0.01	0.4%
Morbidity (EAI)	−0.004	−0.04%
Morbidity (EID)	0.05	2%
Morbidity (EII)		
Morbidity (MET)	−0.1	−4%
*Mortality*	−0.04	−1.6%
Mortality (EAI)	−0.04	−1.6%
Mortality (EID)		
Mortality (EII)		
*Mortality (MET)*	−0.04	−1.6%
Immunisation knowledge	0.19*	7.5%
Immunisation knowledge (EAI)	0.2	7.9%
Immunisation knowledge (EID)	0.31*	12.2%
Immunisation knowledge (EII)		
Immunisation knowledge (MET)	0.09***	3.6%
*Immunisation attitudes*	0.14	5.6%
Immunisation attitudes (EAI)	0.47	18.1%
Immunisation attitudes (EID)	−0.11	−4.4%
Immunisation attitudes (EII)		
Immunisation attitudes (MET)	0.06*	2.4%
*Vaccination card availability/retention*	−0.01	−0.4%
Vaccination card availability/retention (EAI)		
Vaccination card availability/retention (EID)		
Vaccination card availability/retention (EII)		
Vaccination card availability/retention (MET)	−0.01	−0.4%
*Satisfaction with health services*	0.04	1.60%
Satisfaction with health services (EAI)		
Satisfaction with health services (EID)		
Satisfaction with health services (EII)		
Satisfaction with health services (MET)		
*Formal health worker capacity and performance*	0.11	4.40%
Formal health worker capacity and performance (EAI)		
Formal health worker capacity and performance (EID)		
Formal health worker capacity and performance (EII)		
Formal health worker capacity and performance (MET)		

*p < .05; **p < .01; ***p < .001.

#### 6.3.2 Qualitative synthesis of results

##### 6.3.2.1 Barriers to immunisation

**Figure 95:**
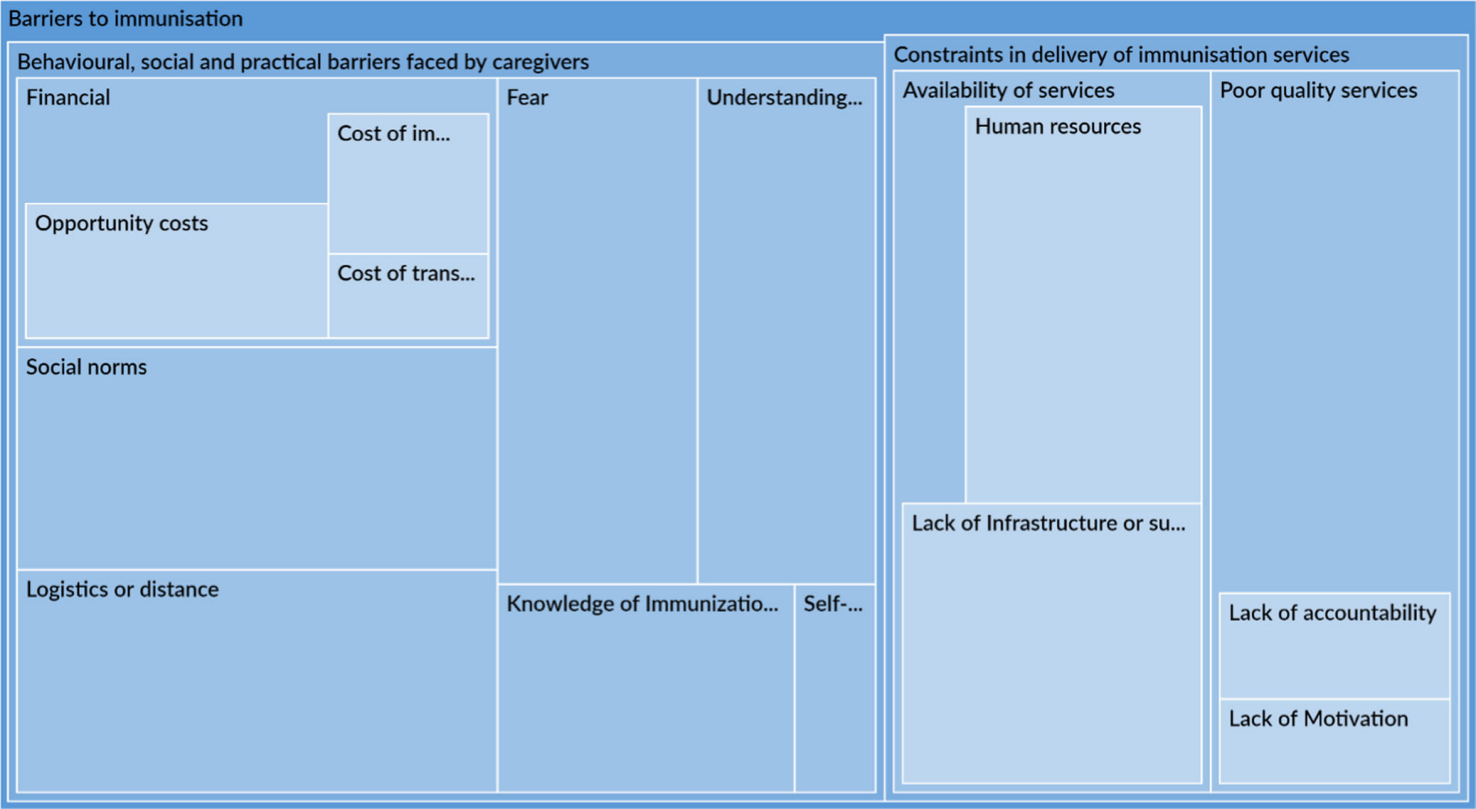
Summary figure: Behavioural, social and practical barriers faced by caregivers were more consistently experienced than constraints in delivery of immunisation services. The hierarchy charts showing barriers by type of community engagement are in Supporting Information: Appendix J: barriers to immunisation.

Most information on barriers came from studies which used engagement as the intervention. Often, multiple barriers were cited in the same sentence. The vast majority of the barriers were identified through individual interviews and focus group discussions in the primary papers from which they were drawn. In addition, these were often supported by the authors' reflections, implementers' experiences, and observations. Sensitivity analysis, in which only qualitative papers that received an assessment score greater than 20 were included, indicated similar results. Therefore, we view the strength of the evidence to be high. Behavioural, social and practical barriers faced by caregivers were more consistently cited than direct constraints within the health system (Figure 95, Table 10).

**Table 10:** Comparison of the barriers to immunisation in communities in which community engagement activities were undertaken

	Community engagement as the intervention	Community engagement in the design of the intervention	Community engagement in the implementation autonomy of the intervention
Availability of resources	Staff		
	Absenteeism	Staff	Staff
	Stockouts	Stockouts	Post abandonment
	Building infrastructure		Stockouts
Quality of services	Attitudes of health workers	Attitudes of health workers	Attitudes of health workers
		Quality affected attendance	Services not implemented as intended
Costs	Other costs	Other costs	Other costs
	Cost of the vaccine		Cost of the vaccine
Logistics or distance	Distance	Convenience	Convenience
		Distance and access	Transport
Social norms	Religious beliefs		Spousal consent
	Beliefs of others		Religious beliefs
Fear	Side effects (and overcoming this fear) Trust in the government or medicine	Side effects (from past experience)	
Understanding of importance	Cost and importance		
	Fear and importance		

The size of the rectangles reflects the number of times a unique theme was mentioned by an IE and its associated papers. Larger rectangles indicate that a theme was mentioned more times. The size of the rectangles corresponds to the relative frequencies presented in Table 7. Nested boxes reflect the proportion of times the main theme text also fell into the sub‐them.

###### 6.3.2.1.1 Behavioural, social and practical barriers faced by caregivers

The most consistent behavioural, social or practical barrier faced by caregivers was cost. However, the kind of cost which posed a barrier was inconsistent, and mostly not that of the vaccination itself. Associated costs include opportunity costs, transport costs, and the costs of treating side effects. The cost of the immunisation itself was mentioned as a barrier by community members that received engagement as the intervention or engagement in the implementation of the intervention.

*Logistics and distance* were also consistent barriers to immunisation in settings where all three types of interventions occurred. The nature of the logistical or distance challenge was variable. Distance alone was reported as an issue in the communities with engagement as the intervention. However, Olken (2014) mention other logistical barriers such as wait times and language barriers. In settings where the community was involved in the design or implementation of the intervention, convenience was an issue in addition to distance. Community members in these areas reported issues related to wait times and the timing of clinic visits. Adamu et al. (2019a) (Adamu et al., 2019b pre‐implementation and Adamu, 2019 Dissertation) mention demand being lower than it could be because women were unable to arrive at clinics at specified times due to competing demands.

*Fear* of side effects was common in communities in which engagement was the intervention or engagement was used in the design of the intervention. The concern was consistently related to known side effects and affected by past experience of side effect and trust in the government or medicine. The issue of vaccines causing sterilisation was also mentioned. In one case, the intervention reduced these fears (Gurley, 2020).

*Social norms* around immunisation posed consistent barriers; however, the source of the social restriction was variable. Spousal consent limited immunisations in areas where engagement was used in the implementation of interventions; women could not vaccinate their children without the explicit approval of their husband, who may refuse. Religious beliefs sometimes resulted in the preference for non‐Western medicine. In areas that received engagement as the intervention, religious beliefs and the influence of beliefs held by others sometimes inhibited immunisation. In particular, mother‐in‐laws and other family members who do not value immunisation may pose a barrier to immunisations.

Failure to *understand the importance of vaccination* was not consistently observed, but reported in settings that received engagement as the intervention. When this barrier was mentioned, it was always paired with another, such as cost or fear. Authors implied that these other barriers were experienced and could not be overcome because there was a lack of understanding of the importance of vaccinations.

###### 6.3.2.1.2 Constraints in delivery of immunisation services

Constraints in the delivery of immunisation services were widely reported, but did not seem to be as common of a barrier as the behavioural, social and practical barriers faced by caregivers. The *availability of services* was the most consistently reported delivery barrier across all three types of engagement. Limited staff and stockouts were dominant delivery challenges. Communities that had engagement as the intervention or were engaged in implementation consistently have issues with staff being assigned but unavailable. Poor infrastructure, such as dilapidated buildings, was a challenge in communities that were involved in the design of the intervention. In areas where engagement was the intervention or the community was involved in the design of the intervention, the *quality of services* was also a more consistently reported barrier than any behavioural, social, or practical barrier faced by caregivers. Often, these were related to health worker attitudes, either a lack of motivation or conflict with caregivers.

##### 6.3.2.2 Facilitators of immunisation

**Figure 96:**
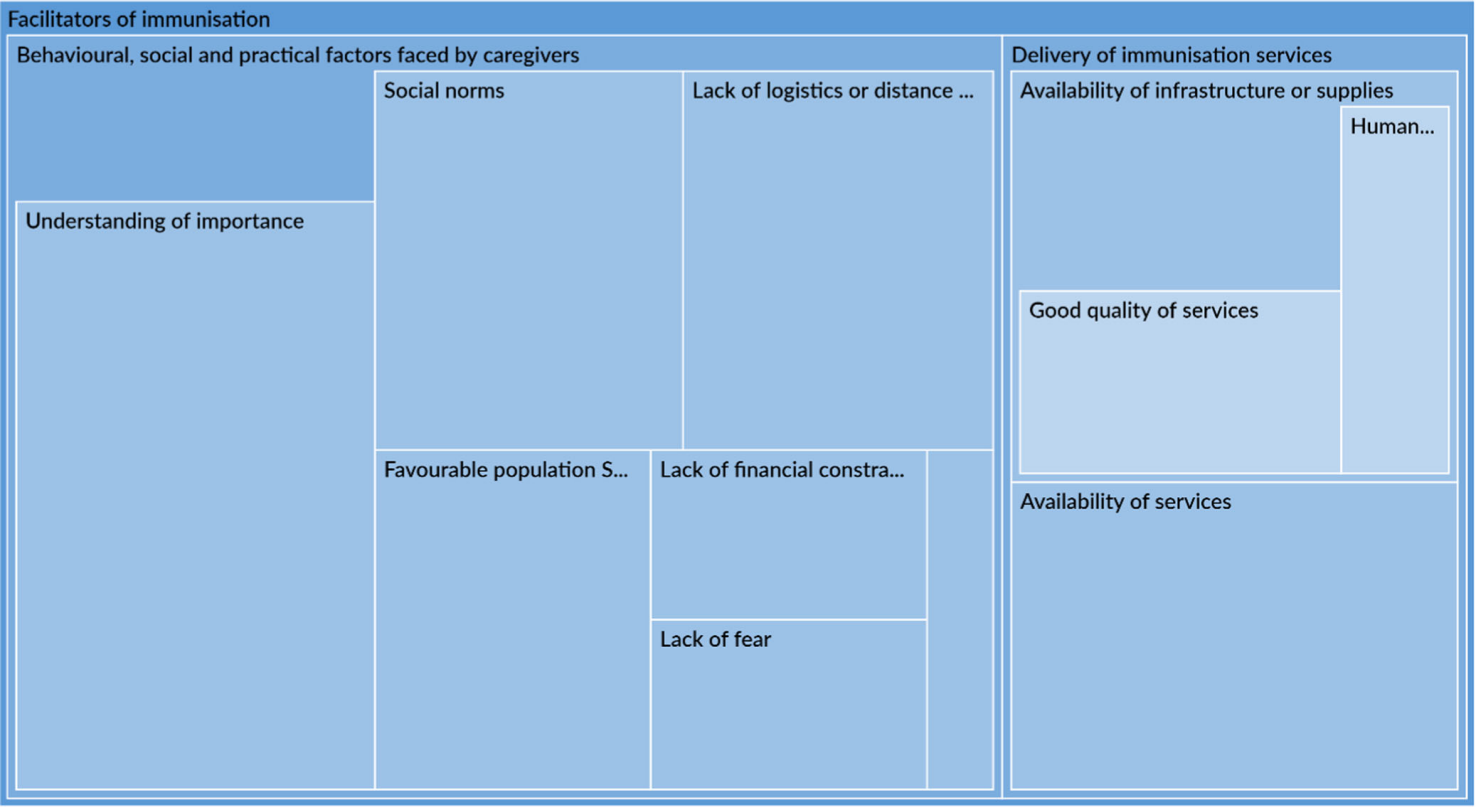
Summary figure: Facilitators related to behavioural, social and practical factors faced by caregivers were more common than those related to delivery of immunisation services. There was little variation by type of community engagement. The charts by type of engagement are presented in Supporting Information: Appendix J: Facilitators or immunisation.

Fewer studies discussed facilitators of immunisation as compared to barriers. Much of the information coded came from studies in which the intervention was engagement. To a substantial extent, the sources of this information were key individual interviews and focus group discussions. These were often supported by surveyor or enumerator's observations. In some instances, discussion on facilitators in the primary papers was informed by existing literature. A sensitivity analysis, comprising 19 qualitative papers with a risk of bias assessment score of 20 or higher, indicated similar results. Therefore, we view the evidence to be of high strength. Facilitators related to behavioural, social and practical factors faced by caregivers were more common than the facilitators related to the delivery of immunisation services (Figure 96). A segregated analysis of the facilitators by the engagement type was not possible due to the limited information coded on this theme.

###### 6.3.2.2.1 Behavioural, social and practical barriers faced by caregivers

The most consistent facilitator across all intervention types was the caregiver's *understanding of importance* of immunisation. In general, studies associated caregivers' awareness and perception of the benefits of vaccination to an enabling environment for immunisation uptake (Adamu et al, 2019a; Banerjee, 2020). In most instances caregivers were able to link benefits of vaccines to the diseases it prevented (Andersson et al., 2009; Cockcroft et al., 2009; Pramanik et al., 2020).

*Lack of logistics or distance related issues* and enabling *social norms* were also consistently sighted as facilitators of immunisation. When it came to logistics, easy access either in the form of proximity to a health care facility (Andersson et al., 2009; Cockcroft et al., 2009) or provision of transportation to an immunisation camp (Olken, 2014; Rahayu, 2008) were cited as enabling factors. *Social norms* were mentioned as a facilitator in instances where mothers had a significant influence of over decisions regarding her child's health. In addition, peer influence was also mentioned as a factor (Banerjee, 2020). Though less consistently cited than the other factors, *favourable Socioeconomic characteristics* such as maternal education (Admassie et al., 2009) were also cited as facilitators of immunisation.

###### 6.3.2.2.2 Constraints in delivery of immunisation services

Delivery of immunisation services was less consistently reported as a facilitator as compared to behavioural, social and practical factors. The most consistent delivery related facilitator was the *availability of infrastructure or supplies* which included provision of good quality of services, availability of adequate healthcare providers at the point of care and availability of vaccine supplies.

The size of the rectangles reflects the number of times a unique theme was mentioned by an IE and its associated papers. Larger rectangles indicate that a theme was mentioned more times. Nested boxes reflect the proportion of times the main theme text also fell into the sub‐them.

##### 6.3.2.3 Reasons for intervention success

Most studies with significant positive quantitative impacts provide some plausible explanation for why an intervention succeeded. These were often based on authors' notes, experiences and impressions of *why* their hypothesis was proven true. We expect the authors to be a good source of this information because of their knowledge of causal linkages and assumptions made in the intervention theory of change. Individual interviews and focus group discussions supported the authors' conclusions. The sensitivity analysis, which included qualitative papers with an assessment of score of 20 or higher, showed similar results. Given this, we consider the evidence to be of high strength.

**Figure 97:**
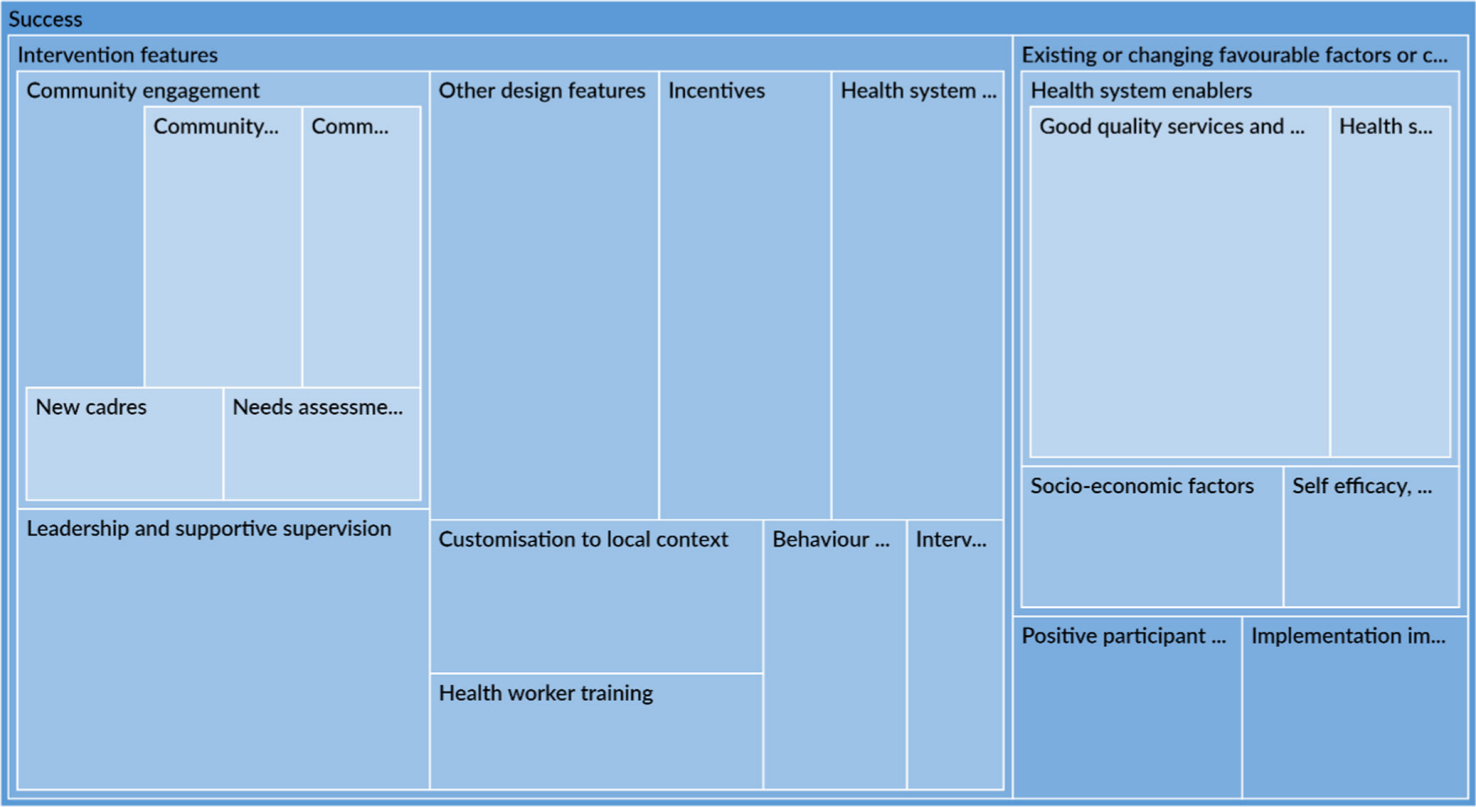
Reasons for success: a summary figure. Figures for reasons for success by type of engagement are presented in Supporting Information: Appendix J: reasons for success.

Overall, reasons for intervention success were consistently attributed to specific *intervention features* across all engagement types. These included community engagement, leadership and supportive supervision, customisation to local context, incentives, health system integration and other design features. Though not as common, success was also attributed to *existing or changing favourable characteristics* within a given context such as availability of and access to good quality health services as well as positive participant views with respect to the intervention (Figure 97; Table 11).

**Table 11:** Comparison of the reasons for intervention success by type of community engagement

	Community engagement as the intervention	Community engagement in design	Community engagement in implementation autonomy of interventions
Intervention features—community engagement	Community dialogues Creation of new cadres	Community dialogues	Community involvement in planning and implementation
		Needs assessments	
Intervention features—Leadership and supportive supervision	Encouragement from leaders	Supportive supervision	Involvement of leadership
			Health worker autonomy
Intervention features—Health system integration	Involvement of government in implementation	Separate workforce dedicated to the intervention	Intersectoral integration of health programmes
Intervention features—Health worker training	Training on monitoring health cards of children	Training of all health personnel involved in the intervention	Training of all health personnel involved in the intervention
Favourable contextual factors—Health system enablers	Good quality of services	Good quality of services	Good quality of services
	Health service access		
Favourable contextual factors—Socioeconomic factors	Female literacy	General literacy levels	Primary schooling of girls and women
		High maternal education	
Improvements in intervention Implementation over time	‐	Health worker performance improvement over time	Resolution of fidelity/adherence issues
Positive participant/beneficiary views	Caregiver perception	Health worker perception	Health worker perception

The size of the rectangles reflects the number of times a unique theme was mentioned by an IE and its associated papers. Larger rectangles indicate that a theme was mentioned more times. The size of the rectangles corresponds to the relative frequencies presented in Table 10. Nested boxes reflect the proportion of times the main theme text also fell into the subtheme.

###### 6.3.2.3.1 Community engagement as the intervention

Studies in which engagement was the intervention consistently attributed success to characteristics associated with intervention features and relatively less consistently to existing or changing favourable characteristics within a given context.

Notably, success was attributed to *intervention features* associated with community engagement such as community dialogues which were participatory in nature and improved relationship between the health system and communities (Assegaai, 2018; Findley et al., 2013; Padayachee, 2013). Involvement of community members such as traditional and religious leaders in planning and implementation of an intervention also came up as a reason for success (Oyo‐Ita, 2020). In a few instances, success was also attributed to acceptance of health worker cadres by the communities as long as these health workers belonged to the communities they served (Biemba et al., 2016).

Studies also attributed intervention success to nonengagement *intervention features* including incentives given to caregivers, leadership and supportive supervision which improved overall health service delivery and health worker performance. As noted above, some studies also cited *existing or changing favourable characteristics within a given context* as reasons for success. Among these, those related to the health system, particularly, good service quality (Rao, 2014) and, to a lesser extent, access to services (Banwat, 2015; Banwat et al., 2014) were consistently reported. Interestingly, none of the studies in this engagement category mentioned *implementation improvements* as a reason for intervention success.

###### 6.3.2.3.2 Community engagement in design

The most consistent reasons for success were related to *intervention features* with the most reported characteristics being community engagement and provision of incentives to caregivers. Within community engagement, participasttory community dialogues (Andersson et al., 2009), stakeholder consultations on intervention design (Modi, 2019) and community involvement in planning and implementation (Adamu et al, 2019a) were associated with intervention success. Receiving incentives was perceived to be motivating and empowering by the intended beneficiaries (Banerjee, 2020; Nagar et al., 2020).

Besides community engagement and incentives, o*ther intervention features* that were consistently reported included leadership and supportive supervision, health worker training and customisation to local context.

Very few studies discussed or attributed success to reasons other than intervention features. Nevertheless, the few studies that did, referred to *existing or changing favourable contextual factors* such as a high prevalence of maternal education (Johri et al., 2020) or *improved implementation* by resolution of issues related to adherence (Modi, 2019). A few studies also discussed *positive participant or beneficiary views* that could have potentially contributed to intervention success.

###### 6.3.2.3.3 Community engagement in implementation autonomy of intervention

Similar to the previous two engagement categories, reasons for success in interventions with engagement in implementation were also consistently attributed to certain *intervention features*. These characteristics were associated with certain aspects of *community engagement* itself such as conducting stakeholder consultations (Modi, 2019) or involvement of community members such as health workers in intervention planning and implementation (Adamu, 2019). Other aspects of intervention features such as *integration with the health system, customisation to the local context* and *leadership and supportive supervision* were also consistently cited.

Like the engagement in design category, crediting intervention success to *existing or changing favourable contextual factors* or *improved implementation* was less consistently reported among studies in which community engagement was involved in the implementation of the intervention. Nevertheless, *health system enablers* such as good quality of services and access to health services were mentioned as factors which could have contributed to intervention success.

##### 6.3.2.4 Reasons for intervention failure

Most studies that did not find a significant positive impact attributed the failure of the intervention to a specific cause. The most consistently reported reason for failure across all engagement types was *not accounting for contextual constraints*, though the exact nature of these reasons varied. Social norms such as misconception regarding immunisation and health worker attrition were reported as common challenges in interventions that were community engagement. Interventions that used engagement in design consistently reported poor access to health services and caregivers' skepticism regarding benefits of immunisation as the reasons for failure. Poor quality of health services was noted as an issue in interventions where engagement occurred in implementation.

**Figure 98:**
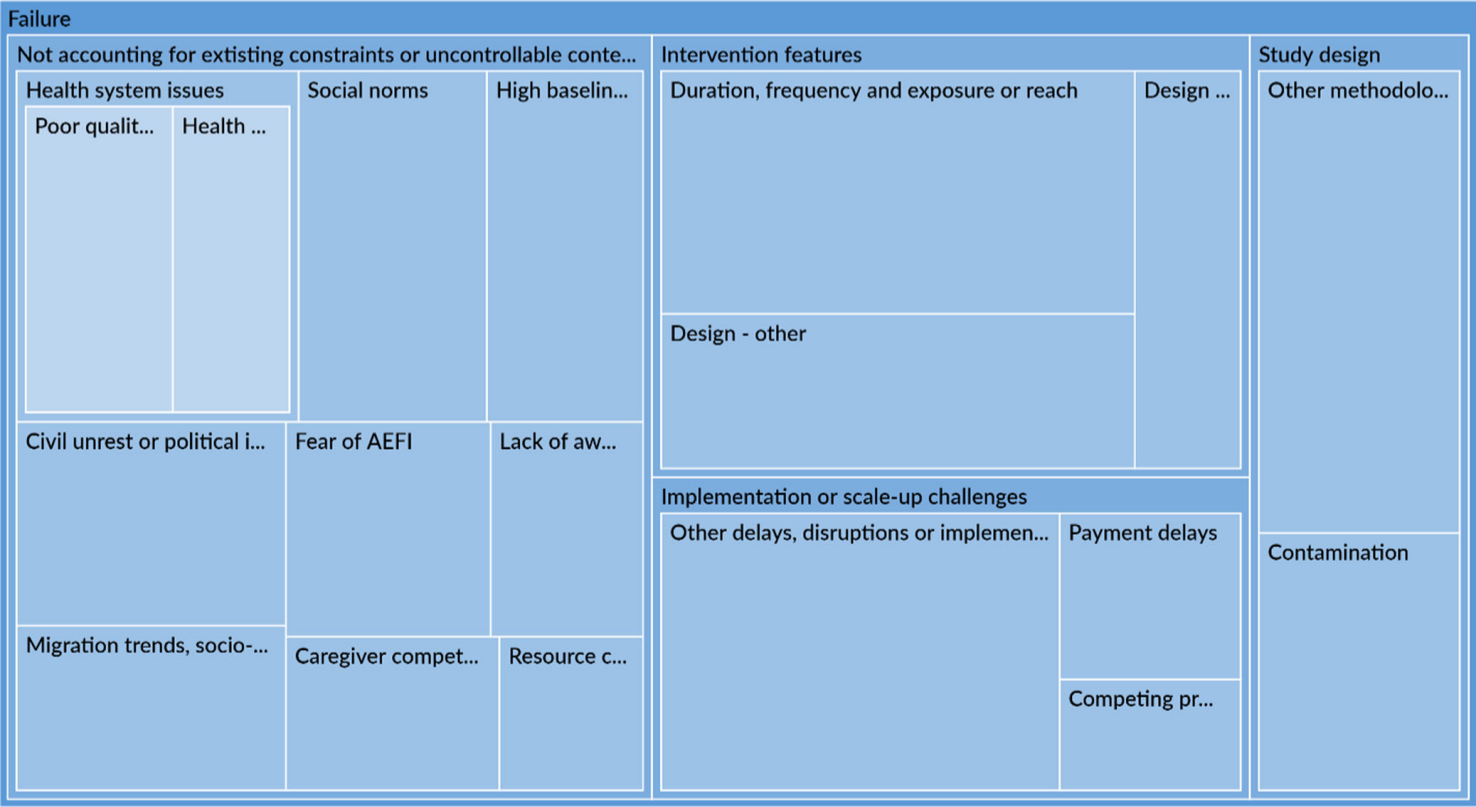
Summary figure: reasons for failure

The other consistently reported reasons for failure were attributed to inadequate *intervention features* and *implementation challenges*. Among the intervention features, inadequate duration, frequency or exposure to the intervention were the most notable reasons for failure across all engagement types. Though relatively less common, the nature of the community engagement itself was also attributed to failure in interventions that used engagement in implementation or as intervention. Among implementation challenges, disruption due to inadequate implementation instructions, difficulty in accessing the implementation sites due to geographic proximity and competing priorities of health workers were noted as some of the prevalent challenges (Figure 98; Table 12).

**Table 12:** Comparison of the reasons for intervention failure by type of community engagement

	Community engagement as the intervention	Community engagement in design	Community engagement in implementation autonomy of the intervention
Not accounting for contextual constraints—health system issues	Health worker attrition	Issues with accessing health services	Poor quality of services
Not accounting for contextual constraints—prevalent social norms	Fear of sterilisation	Skepticism towards perceived value of immunisation	‐
Not accounting for contextual constraints—baseline immunisation coverage	High baseline coverage	High baseline coverage	High BCG coverage
Not accounting for contextual constraints—unrest, insecurity or other	Civil unrest	Political instability	‐
	Natural calamity		
Intervention features—Duration, dose or frequency of intervention	Inadequate intervention duration or exposure	Inadequate intervention frequency	Inadequate intervention duration or exposure
Intervention feature—issue in design of engagement	Lack of collective action	‐	Elite capture
Implementation challenges—disruptions or delays	Resource constraint due to multiple programmes running at the same time	Fidelity issues	Staff turnover^a^
Study design—Contamination	Contamination due to similar programmes	Contamination due to similar programmes	Contamination between treatment and control

^a^ Most of the references coded under this theme were from Olken (2014) and associated papers.

Another less consistently reported reason for failure was study design issues such as contamination between treatment and control other methodological challenges such as biased sampling frame and issues with enroling study populations. The reasons for failure were largely based on authors' notes or experiences and individual interviews. We expect the authors to be a good source of this information because of their knowledge of causal linkages and assumptions made in the intervention theory of change. Focus group discussions and implementers' experiences supported these findings. The sensitivity analysis, which included qualitative papers with an assessment of score of 20 or higher, showed similar results. Except for one subtheme which was dominated by one or two studies, other sub‐themes were consistently distributed across the evidence base (indicated in Table 11). Therefore, we consider the evidence to be of high strength.

###### 6.3.2.4.1 Community engagement as the intervention

*Not accounting for contextual trends* were the most consistently reported reason for failure. Social norms, health system issues, migration, civil unrest and fear of adverse events following immunisation (AEFI) were reported consistently within existing constraints. Most of the challenges related to social norms mentioned misconception about immunisation (Banerjee, 2020) and limited decision‐making powers of mothers (Gurley, 2020) as the reason for low immunisation uptake. Civil unrest, political instability and natural calamities also hindered a few interventions from achieving intended impacts (Morris et al., 2004; Pramanik et al., 2020).

The other reasons for failure comprised issues related to *intervention features, implementation issues* and, to a lesser extent, *study design*. Issues with intervention feature ranged from inadequate duration or frequency of the intervention to certain aspects of community engagement failing to elicit a positive response among study participants (More, 2012; Pramanik et al., 2020). Implementation issues were consistently reported as reasons for failure. Some of the challenges mentioned were competing demands by different programmes running simultaneously (Carnell, 2014), high staff turnover (Gurley, 2020; Olken, 2014) and low implementation fidelity (Oyo‐Ita, 2020). These issues are also discussed in detail under the ‘uptake and fidelity challenges theme’.

###### 6.3.2.4.2 Community engagement in design

Most consistently reported reasons for failure of interventions in which communities were engaged in the design broadly fell under *not accounting for contextual constraints*. Among these, social norms were the most consistently reported reason with vaccine hesitancy being one of the driving forces behind immunisation refusal (Banerjee, 2020; Gurley, 2020). Other reasons for failure within this theme were high baseline immunisation coverage, health system issues related to quality of services, and civil unrest or political instability. Prevailing socioeconomic trends, lack of awareness, fear of AEFI and caregiver competing priorities were also attributed to intervention failure, though less consistently reported as compared to other factors.

Besides existing constraints, a few studies also attributed failure *intervention features* with the insufficient duration, frequency and exposure of intervention as a possible reason for failure. A much smaller subset of studies also referred to issues related to study design and implementation or scale‐up challenges.

###### 6.3.2.4.3 Community engagement in implementation autonomy of interventions

Fewer studies under this engagement classification reported reasons for failure as compared to success. Among the reasons cited for failure, those related to *not accounting for contextual constraints* were the most consistently reported followed by *implementation challenges*. Most of the references to implementation challenges or failures were made by Olken (2014) and its associated studies which discussed multiple implementation barriers to a block‐level grant intervention. Though less consistently reported, some studies also mentioned issues with intervention features or study design as the reasons for intervention failure. Highlighting the failure of community engagement, Olken (2014) (Grayman, 2014) discussed how elite capture wherein local community leaderships and elites leveraged the intervention to retain social standing became an issue for the programme.

##### 6.3.2.5 Reasons for heterogenous impacts

A small subset of studies is also coded under the theme *heterogeneous impacts*. Even though they reported overall significant positive quantitative findings, these studies discussed their heterogenous or nonuniform results with respect to not achieving the intended impact among certain subgroups, study sites or reported null results for some of the immunisation‐related outcomes. Each of these studies offer a slightly different perspective on their heterogeneous results. For instance, Adamu, et al. (2019a) and Carnell et al. (2014) postulate that the characteristics of individuals involved in the intervention implementation, contextual factors or implementation processes could have led to partial intervention success or measurable positive gain in only select study sites. Interestingly, both Banerjee et al. (2020) and Gibson et al. (2017) highlight very different challenges with respect to their interventions comprising incentives to caregivers. Banerjee et al. describe how incentives designed to motivate caregivers did not work on Muslim minorities due to high vaccine hesitancy which stemmed from the belief that vaccination causes sterilisation. On the other hand, the Gibson study reported that their SMS reminders did not work in the absence of incentives, implying that reminders alone were insufficient to encourage behaviour change among caregivers and result in immunisation uptake. This shows that the benefit of providing incentives may not be consistent across different contexts and uptake may be influenced by other socioeconomic factors. Both studies further elaborate the limits to which incentives can address deep rooted mistrust of vaccination among certain subgroups. Overall, the discussion on these studies provide important insights about how external factors, implementation variability or invalid theory of change assumptions may contribute to partial intervention success or failure. We also looked through the literature for *unintended impacts* but there was insufficient information to draw any broader conclusions.

##### 6.3.2.6 Uptake and fidelity challenges

Uptake and fidelity challenges were generally identified by authors through primary data or personal experience and knowledge of the intervention. Because this theme relates to how the project was implemented, we expect that authors are a good source of this information. Individual interviews and focus group discussions supported the findings. Themes were broadly consistent in the full analysis and in sensitivity analysis in which on the qualitative papers with a quality assessment score greater than 20 were included. However, there are several instances in which a single subtheme is dominated by one or two studies. These are indicated in Table 12 and discussed in the text. Given this, we believe that the evidence on uptake and fidelity challenges has only moderate strength.

**Figure 99:**
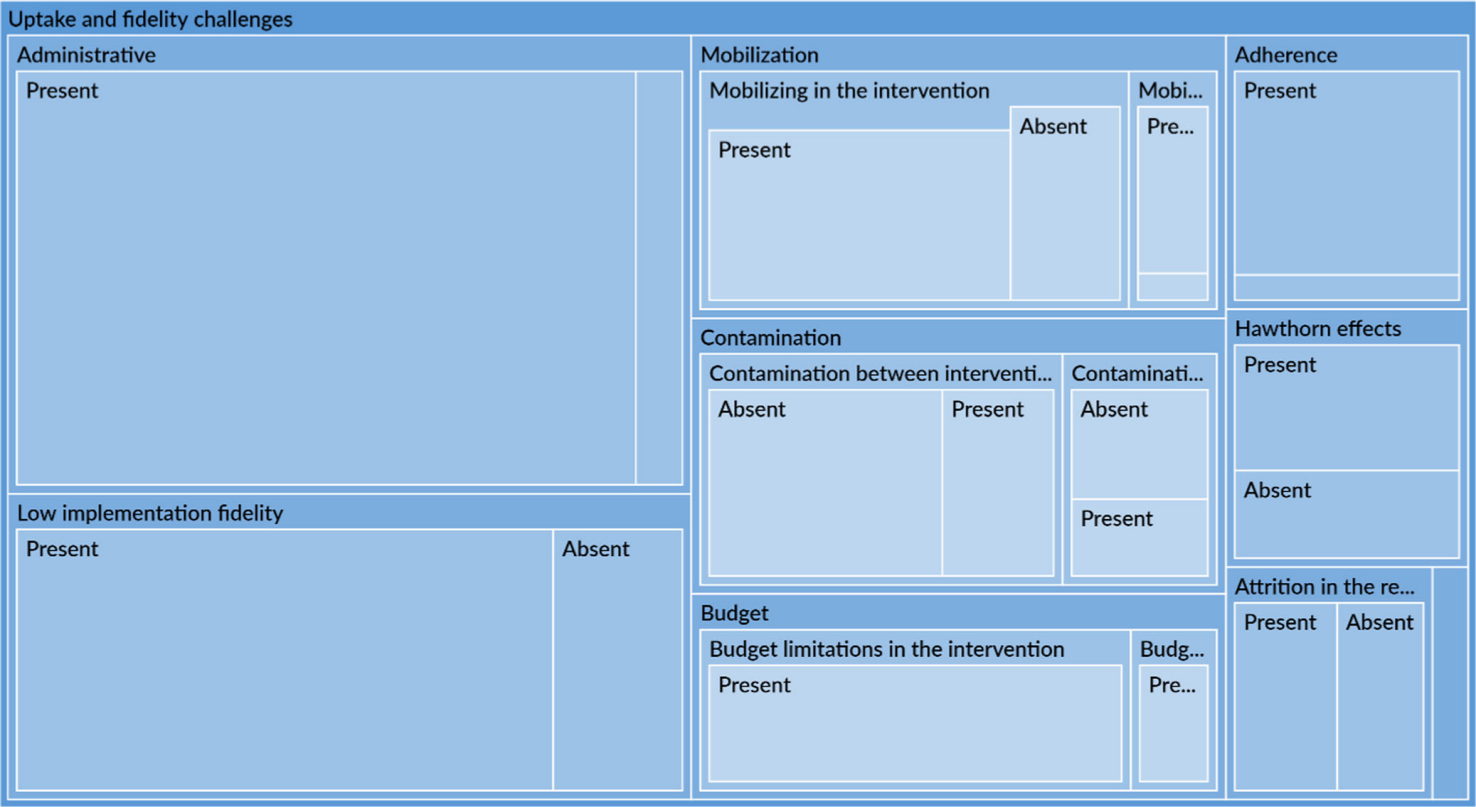
Summary figure: uptake and fidelity challenges. Coding is presented in a present/absent format because, in some cases, authors explicitly stated that a challenge was not encountered.

The most consistently reported challenges were related to administration, mobilisation, fidelity, and contamination (Figure 99; Table 13). Administrative challenges were cited consistently across all three types of interventions, but their nature varied. Although technical issues and politics plagued interventions that used community engagement in the design of interventions or as the intervention, these issues were not common in interventions that used engagement in their implementation. Staffing was a challenge in interventions that used engagement in implementation or as the intervention. Implementation fidelity was mentioned as a challenge by all three types of interventions. In all cases, the most consistent issue was that realities on the ground forced changes to the intervention. The issue of mobilising people into the intervention appears to be common; however, this category is dominated by two papers (Olken, 2014; Pérez et al., 2020) which cite a variety of mobilisation challenges. As such, the evidence on this theme is somewhat weak. Contamination was also consistently reported. For the most part, it was mentioned due to its absence. Interventions whose goal was engagement discussed this topic much more than interventions with engagement in their design or implementation.

**Table 13:** Comparison of the challenges faced by interventions using different types of community engagement. Numbers reflect relative frequency.

	Community engagement as the intervention	Community engagement in the design of the intervention	Community engagement in the implementation of the intervention
Administrative challenges	Technical issues	Technical issues	Funding disbursement
	Politics	Politics	Communication across stakeholders
	Coordination	Bureaucracy	Staffing
	Staffing	Security	
Mobilisation challenges	Gender based religious and cultural norms prevented participation***	Recruiting people into the intervention due to work, distance, and competing priorities*	Recruiting facilitators and participants into the intervention due to other opportunities, miscommunication of intervention expectations, declines in incentives, caregiving obligations, and more**
Fidelity challenges	Failure to account for on the ground realities resulted in changes or failures in intervention implementation.	Failure to account for on the ground realities resulted in changes or failures in intervention implementation	Failure to account for on the ground realities resulted in changes or failures in intervention implementation
	Improper or insufficient supervision was also common.		
Contamination	Authors were more likely to discuss the absence of this issue than to report upon its presence.	Authors were more likely to discuss the absence of this issue than to report upon its presence.	
Budget restrictions			Inadequate or delayed pay resulted in activities not being implemented.

###### 6.3.2.6.1 Community engagement as the intervention

Discussions of *administrative challenges* consistently related to coordination, technical issues, staffing, and politics. The single study that reported on the absence of certain administrative issues also reported on the presence of many others. This is likely because it was a uncommonly long report that included significant qualitative components.

Issues with the faithful *implementation* of these interventions were consistently the result of improper supervision and management or a failure to account for practicalities on the ground. We found one case where the administrative challenges directly led to low implementation fidelity. However, it was not uncommon for studies to reported statistics indicating high fidelity.

The studies that discussed contamination largely did so to demonstrate that contamination was not a concern. Authors consistently focused on the issue of contamination between intervention and control sites. This was often addressed with vague statements. Distance was largely used to avoid contamination.

Discussion of challenges in mobilisation interventions was dominated by a single source (Olken et al., 2014 also discussed the engagement in implementation section). However, Rahman et al. (2016) also discussed several mobilisation issues that resulted from religious and cultural norms, largely around gender, preventing participation.

###### 6.3.2.6.2 Community engagement in the design of the intervention

Among interventions that used community engagement in their design, the most consistently reported *administrative challenge* related to technical issues, largely surrounding phones and internet connections (Table 13). However, other administrative challenges were related to combinations of politics, bureaucracy, and security concerns. The single paper that indicated a lack of administrative challenges reported successful syncing to the server, but also reported on other technological issues.

*Mobilisation challenges* largely related to recruiting people into the intervention. Most of this information came from a 2020 (Pérez et al., 2020) paper which presented information on the implementation fidelity and acceptability of an intervention that combined in‐person and digital education to promote vaccination (Johri et al., 2020). Authors describe challenges with participants being unable to attend due to work, distance, and competing priorities. Limited access to phones made mobilisation difficult as well.

Acknowledgement of *low implementation fidelity* was consistently accompanied by discussions of why changes were made or the implications of these changes for results. Generally, changes in the intervention plan were forced due to practical constraints that had not been accounted for and were expected to result in an attenuation of impacts. Reports of high fidelity were succinct and responded to concerns a reader may be expected to have.

*Contamination* can occur between the intervention and control or due to ongoing activities of other programs. The former of these was reported more consistently than the latter. Authors were more likely to discuss the absence of this issue than to report upon its presence. Some offered explanations as to why contamination was not a concern or steps taken to avoid contamination. Others reported on results related to measures of contamination.

###### 6.3.2.6.3 Community engagement in the implementation autonomy of the intervention

*Administrative challenges* in interventions that used engagement in their implementation largely related to funding disbursement, communication across stakeholders, staffing issues (Table 10). One study (Björkman & Svensson, 2009) reported that they did not believe administrative differences affected results. This was coded as the absence of an administrative challenge, but does not mean that administrative issues were not encountered.

*Implementation fidelity* appears to have been more of a challenge for these interventions than for those that leveraged community engagement in their design. These issues were consistently related to the intervention not functioning as expected. In some cases, this was because the reality on the ground did not reflect expectations. However, two studies that reference challenges with implementation fidelity also report upon successes in implementing according to plan.

All reports of challenges *mobilising* participants into interventions that were implemented by the community came from a paper related to an intervention that used block grants to support maternal and child health and education (Olken, 2014). Challenges were encountered in the recruitment of both facilitators and participants. These related to other opportunities, miscommunication of intervention expectations, declines in incentives, caregiving obligations, and more. However, Herrara‐Almanza (2018) report that community health workers felt strong responsibility for the wellbeing of their communities and attrition was low.

Interventions that used engagement in their implementation also experienced significant *budgetary constraints*. Almost universally, these constraints were the result of inadequate or delayed pay. Often, this resulted in activities not being implemented.

#### 6.3.3 Synthesis of cost evidence

##### 6.3.3.1 Cost effectiveness analysis

This section reports on the results of the analysis of the inventory of included studies (see Table 14) and of the estimates of the non‐vaccine cost per dose of interventions to increase absolute immunisation coverage by 1% (see Table 15).

**Table 14:** Characteristics of studies including any type of cost analysis

Author (date)	Country	Evaluation design	Intervention exposure (months)	Type of cost analysis				
				Total cost	Cost‐efficiency analysis	Cost‐effectiveness analysis	Cost‐benefit analysis	Cost per QALY or DALY
Andersson (2009)	Pakistan	Experimental	8	✓				
Banerjee (2010)	India	Experimental	18	✓		✓		
Banerjee (2020)	India	Experimental	14	✓		✓		
Björkman and Svensson (2009)	Uganda	Experimental	0.17	✓				
Borkum (2014)	India	Experimental	12	✓	✓	✓		
Demilew et al. (2021)	Ethiopia	Experimental	17	✓				
Gurley (2020)	India	Experimental	11	✓		✓		
Johri (2020)	India	Experimental	3	✓				
Mohanan (2020)	India	Experimental	4	✓				
Modi (2019)	India	Experimental	12	✓	✓	✓	✓	✓
More (2017)	India	Experimental	24	✓				
Morris (2004)	Honduras	Experimental	24	✓		✓		
Nagar et al. (2020)	India	Experimental	20	✓		✓		
Olken (2014)	Indonesia	Experimental	24	✓		✓		
Oyo‐Ita (2020)	Nigeria	Experimental	18	✓		✓		
Pramanik (2020)	India	Experimental	13	✓				
Seth (2018)	India	Experimental	9.7			✓		
Webster (2019)	Uganda	Experimental	12	✓			✓	
Admassie (2009)	Ethiopia	Quasi‐experimental	48		✓			
Carnell (2014)	Ethiopia	Quasi‐experimental	48	✓				
Findley (2013)	Nigeria	Quasi‐experimental	24	✓		✓		
Saggurti (2018)	India	Quasi‐experimental	2	✓		✓		✓

Abbreviations: DALY, disability‐adjusted life year; QALY, quality‐adjusted life year.

**Table 15:** Detailed estimates of non‐vaccine intervention cost per dose and absolute coverage change

Author (date)	Country	Total cost USD $2019	Treatment	Outcome	Vaccines Reported (list)	Number of doses	Non‐vaccine intervention cost per vaccine dose ($2019 USD)	Absolute coverage change (Intervention—Control)	Non‐vaccine cost per dose of intervention to increase immunisation coverage by 1%	Impact notes	Cost notes	Immunisation notes
Oyo‐Ita (2020)	Nigeria	$15,158	Training of traditional and religious leaders; health workers; and community mobilisation to improve vaccination	Proportion of children with up‐to‐date vaccination	Pentavalent3, Measles	5 per protocol	$4.59	Null effect	negative/null result	Unadjusted proportion, endline (intervention—control)	Incremental to no intervention control group, excludes healthcare system‐building costs	46% is baseline up to date vaccination in intervention, 48% control
Demilew et al. (2021)	Ethiopia	$2,24,269	A system of tracking children's immunisation status using a poster and stamp system	Probability of receiving all 3 doses of DPT/PCV	DPT—Diphtheria, pertussis, and tetanus/PCV—Pneumococcal Conjugate Vaccine	6 per protocol	$23	Null effect	negative/null result	Unadjusted proportion, endline (intervention—control)	Incremental to no intervention control group, excludes healthcare system‐building costs	Baseline immunisation not reported
Seth et al. (2018)	India	$2178	Reminders + Incentives	Proportion of total number of immunisations received by a child divided by the total number of immunisations required	BCG, DPT, H influenzae type B, Polio, Hepatitis B, Rotavirus, Measles	3.21 per child, 574 doses	$4	3.1	$1.252	Unadjusted proportion, (end of study—baseline)	Incremental to no intervention control group, excludes healthcare system‐building costs	40% is baseline immunisation coverage in treatment and 39% in control
Banerjee (2010)	India	$24,599.27	Camps without incentives	Proportion of children fully immunised	Full immunisation is one dose of BCG vaccine, three doses of DPT (diphtheria‐pertussis, tetanus) vaccine, three doses of oral polio vaccine, and one dose of measles vaccine.	2.35 per child	$153.44	12.0	$12.79	Unadjusted proportion, endline (intervention—control)	Incremental to no intervention control group, excludes healthcare system‐building costs	2% is baseline full immunsation rate in the study area
Banerjee (2010)		$12,408.09	Camps with incentives	Proportion of children fully immunised		2.85 per child	$29.22	33.0	$0.89	Unadjusted proportion, endline (intervention—control)	Incremental to no intervention control group, excludes healthcare system‐building costs	2% is baseline full immunsation rate in the study area
Johri (2020)	India	‐	‐		‐	‐	‐	‐	‐	‐	‐	‐
Andersson (2009)	Pakistan	$70,981.46	Three structured community discussions of local vaccination rates; costs and benefits of childhood vaccination; and local action plans.	Uptake of full DPT‐3 and Measles vaccination in children 12–23 months	DPT‐3, Measles	5 per protocol	$50.16	24	$2.07	Unadjusted proportion, endline (intervention—control)	Incremental to no intervention control group, excludes healthcare system‐building costs	Baseline immunisation not reported
Findley (2013)	Nigeria	‐	‐		‐	‐	‐	‐	negative/null result	‐	‐	‐
Morris (2004)	Honduras	‐	‐		‐	‐	‐	‐	‐	‐	‐	
Pramanik (2020)	India	$1,01,636.61	Community‐based learning cycles where a community identifies a problem, makes an action plan, takes action and learns from the process	Full immunisation take‐up	1 BCG dose; 3 doses OPV; 3 doses DPT or Pentavalent vaccine, and one measles vaccine	8 per child 12–23 months	$17.20	FIC = −1%	negative/null result	Unadjusted proportion, endline (intervention—control)	Incremental to no intervention control group, excludes healthcare system‐building costs	75% is baseline full immunisation coverage in treatment and 76% in control
Saggurti et al. (2018)	India	$39,597.45	Participatory behaviour communication on maternal, neonatal, child health and promoting collectivisation processes through women's self‐help groups (SHGs)	Age‐appropriate immunisation among children under one year	9 doses—1 BCG, 1 OPV, 3 OPV, 1,2,3,; 3 DPT, 1 Measles	9 per child under 1 year	$9.37	9	$1.03	Quasi‐experimental. DID estimate. Intervention and control proportions were adjusted for baseline covariates	Incremental to no intervention control group, excludes healthcare system‐building costs	Baseline immunisation not reported
Admassie (2009)	Ethiopia	$10,08,991.46	Received Measles vaccine for children 9 to 11 months	Proportion of children 12–60 months old vaccinated against major diseases. tuberculosis, diphtheria, whooping cough (pertussis), tetanus, polio and measles	8 doses: tuberculosis, polio, diphtheria–pertussis–tetanus, and measles	8 per child 12–60 months	$56.19	9.9	$5.68	Quasi‐experimental. Intervention and control proportions were adjusted for baseline covariates	Incremental cost of running Health Services Extension Programme (HSEP) in the treatment areas, includes system‐building cost to construct and staff a health post	Baseline immunisation not reported
More (2017)	India	$2,13,420.20	Community resource centres	Proportion of children aged 12–23 months fully immunised	BCG, diphtheria, pertussis, and tetanus [three doses], polio, hepatitis B virus [three doses], and measles	8 per protocol	$37.02	5.8	$6.34	Unadjusted proportion endline, (intervention—control). Change was positive but not statistically significantly different from zero	Incremental to no intervention control group, excludes healthcare system‐building costs	65% is baseline full immunisation coverage in treatment and 63% in control
Olken (2014)	Indonesia	$1,22,64,782.40	Village‐level block grants for maternal and child health and education that incorporated relative performance incentives	Proportion of children under two years of age with BCG vaccine	1 dose, BCG	1.00	$36.97	1.60	$23.11	Adjusted average standardised effect (absolute coverage change) was positive but not statistically significantly different from zero.	Incremental cost of preference‐weighted incentive block grant program for vaccines relative to the cost of the program of community block grants without incentives	65.3% is baseline full immunisation coverage
Borkum (2014)	India	$39,571.93	A program that offers nonmonetary incentives for teamwork and goal‐setting activities to improve maternal and child health at health subcenters in India	Proportion of children ages 6–11 months who received DPT‐3 by the age of 6 monsths	DPT‐3	585	$22.54	5.4	$4.17	Adjusted proportion (intervention—control). Difference is positive but not significantly different from zero	Incremental to no intervention control group, excludes healthcare system‐building costs	45.5% is baseline mean for a child fully immunised except measles in the treatment group
Mohanan (2020)	India	$61,344.88	Information treatment	Full immunisation of children 12–23 months	8 doses: BCG, measles, and 3 doses each for Polio and DPT	8 per protocol	$14.10	8.8	$1.60	Unadjusted increase in proportion immunised in intervention relative to control. We report separately by treatment arm because there is corresponding cost info.	Incremental to no intervention control group, excludes healthcare system‐building costs	Baseline immunisation not reported
Mohanan (2020)	India	$61,344.88	Information + Facilitation treatment	Full immunisation of children 12–23 months	8 doses: BCG, measles, and 3 doses each for Polio and DPT	8 per protocol	$12.63	13.4	$0.94	Unadjusted increase in proportion immunised in intervention relative to control. We report separately by treatment arm because there is corresponding cost info.	Incremental to no intervention control group, excludes healthcare system‐building costs	Baseline immunisation not reported
Björkman and Svensson (2009)	Uganda	$1,82,003.09	Community‐based monitoring of public primary health care providers	Proportion of children ages 13–24 months who received required doses of DPT	3 doses: DPT	3	$76.83	6.0	$12.81	Adjusted percent increase in 1‐year old immunisation rate	Back of the envelope total cost, incremental intervention cost relative to control	Baseline immunisation not reported
Banerjee (2020)	India	$3,42,595.94	High slope incentives	Proportion of children under 12 months who received 1 dose of BCG, 4 doses of the oral polio vaccine, 3 doses of penta, 3 doses of rotavirus and 1 dose of measles	BCG, penta doses 1–3 and measles‐1	12 per protocol	$13.81	11	$1.21	Adjusted percent change in fully immunised	Incremental to no intervention control group, excludes healthcare system‐building costs	20.8%–50.05% is the range of baseline means, depending on the district for a child fully vaccinated
		$3,67,078.25	Low slope incentives			12 per protocol	$13.85	12	$1.14	Adjusted percent change in fully immunised	Incremental to no intervention control group, excludes healthcare system‐building costs	20.8%–50.05% is the range of baseline means, depending on the district for a child fully vaccinated
		$78,072.73	Gossip seeds			12 per protocol	NA	‐	‐	No coverage change reported for the Gossip seeds treatment.	Incremental to no intervention control group, excludes healthcare system‐building costs	20.8%–50.05% is the range of baseline means, depending on the district for a child fully vaccinated
Nagar et al. (2020)	India	$2,75,702.59	Tracking system: The Khushi Baby platform, a wearable digital health record (pendant) and with patient‐specific, dialect‐specific voice reminders	Full and timely Immunisation as verified by MAMTA card for children 12–23 months	8 doses: Polio 1–3, BCG, Pentavalent 1–3, Measles	6 per protocol	$41.17	11.2	$3.68	Unadjusted increase in proportion immunised in intervention relative to control	Incremental to no intervention control group, excludes healthcare system‐building costs	25.4% is intervention baseline, full immunisation without birth doses and 22.5% is control
Gurley (2020)	India	$1,75,603.81	PATH—community‐led videos	Proportion of children 6 to 17 months of age who had received all age‐appropriate vaccines in India's vaccine schedule	13 doses: Birth doses (OPV, BCG, HepB); DPT1‐3; HepB1‐3; MR; OPV1‐3	13 per protcol	$26.39	0.88	$29.98	Unadjusted proportion, endline (intervention—control). Difference is positive but not significantly different from zero	Incremental to no intervention control group, excludes healthcare system‐building costs	52.88% is baseline percentage of children in the State of Uttar Pradesh who received all necessary vaccines in 2012–2013
Carnell (2014)	Ethiopia	$2,73,16,577.57	Three pillars approach Eshe project: strengthening health systems, improving health workers' performance, and engaging the community	Percentage of children aged 12–23 months, who received DPT3 vaccination, or Measles	2 doses: DPT3, Measles	2 per protocol	$5,449.19	8.5	$641.08	Coverage change is the average for DPT3 and Measles. Cost data very low transparency, making cost estimate unreliable	System‐building' activities are evident in this intervention. However, cost data have very low transparency. To calculate total cost for treating vaccinated children we total cost/average # children in treatment at endline ages 12–23 (Table 5) for DPT3 3879 + measles 3885 = 3882 × proportion treated × doses)	Baseline immunisation not reported
Modi (2019)	India	$1,60,881.37	A mobile‐phone app to assist ASHAs and health providers to schedule and track home health visits and follow‐on care	Proportion of infants (6–8 months) who received all three doses of DPT or Pentavalent vaccine	3 doses: DPT or Pentavalent vaccine	3 per protocol		Null result	Negative/null result	Unadjusted proportion, endline (intervention—control)	Incremental to no intervention control group, excludes healthcare system‐building costs	Baseline immunisation not reported
Webster (2019)	Uganda	$2,24,224.18	mReach data platform and the ‘Fifth Child', a community engagement strategy to increase immunisation	Proportion of children ages 12–23 months with DPT3 and MCV combined immunisation coverage	2 doses: DPT3, Measles	2 per protocol	$204.63	Null result	Negative/null result	Unadjusted proportion, endline (intervention—control)	Incremental to no intervention control group, excludes healthcare system‐building costs	Baseline immunisation not reported

###### 6.3.3.1.1 Characteristics of included studies

Twenty‐two of the evaluations selected for inclusion in the systematic review reported some type of cost analysis. Of these, 18 used an experimental design and the remaining 4 used quasi‐experimental methods to identify the impact of treatment.

Of the 22 studies, 20 studies reported a total cost of the intervention; 12 included a cost‐effectiveness analysis; three included cost‐efficiency analysis; two studies included a cost–benefit analysis and two analysed cost per quality‐adjusted life year (QALY).

**Figure 100:**
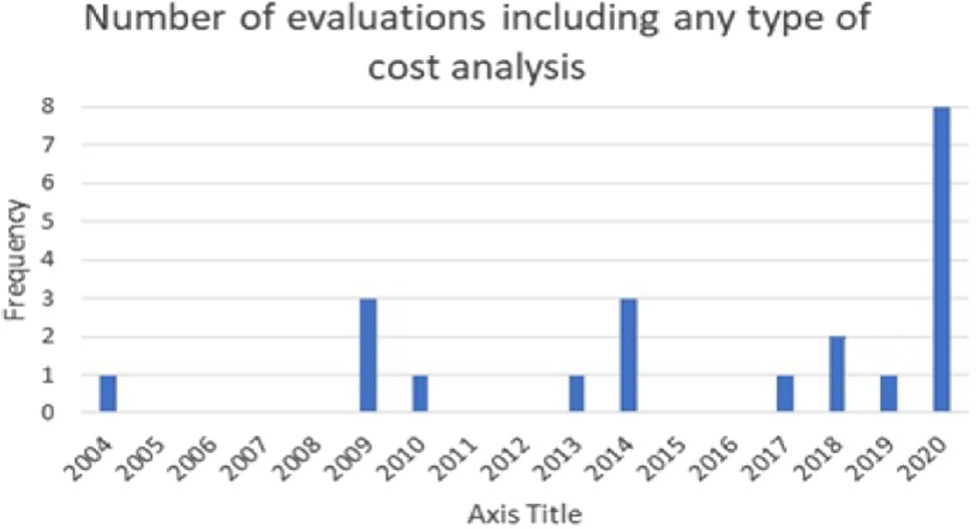
Number of evaluations with any type of cost analysis

By country. 14 of the community participation interventions to improve child immunisation studies were conducted in Asian countries: of these, 12 studies took place in India; and one each in Pakistan and Indonesia. One study was conducted in a Central American country, Honduras and seven studies were conducted in African countries: three in Ethiopia; and two each in Nigeria and Uganda (Figure 100).

The production of cost evidence in published evaluations of community participation interventions to improve child immunisation in LMICs was uneven over the years. The first recorded evaluation with any cost analysis was published in 2004. Between 2009 and 2019, no more than three of the identified evaluations included any type of cost analysis in any given year. In 2020, the largest number of evaluations, seven, were published in a single year. The eight evaluations published in 2020 were required to generate cost analysis in partial fulfilment of the grant requirements in 3ie's Innovations in Increasing Immunisation Evidence Programme. Funding for these studies was provided by the Bill and Melinda Gates Foundation.

###### 6.3.3.1.2 Non‐vaccine cost per dose of intervention to increase immunisation coverage by 1%

Of the 22 included studies reporting cost and impact information, we estimate the non‐vaccine cost per dose of intervention to increase immunisation coverage by 1% for 14. Because several studies report results by treatment arm, we report results for a total of 17 treatment arms. These results are summarised in detail in Table 12.

Three studies and one treatment arm were dropped from the analysis. In the case of Johri et al. (2020) this was because the proportion of children vaccinated was not reported as an outcome of this evaluation. Morris (2004) was dropped because it is unclear how to combine the PRAF II costs reported in a separate paper with intervention as described in Moris, 2020, in addition, the evaluation found a null result on the immunisation outcomes. Findley et al. (2013) was dropped because the reported cost information is insufficient for generating a total cost estimate or cost per vaccine dose, and ‘immunisation status’ is not clearly defined. In the case of the ‘Gossip Seeds’ treatment arm reported in Banerjee (2020), no coverage change information was separately reported for the treatment arm, which was dropped from the analysis as a result.

Six studies, Findley (2013); Pramanik (2020); Webster (2019); Oyo‐Ita (2020), Modi (2019); and Demilew et al. (2021) reported negative or null results and were excluded from the analysis of non‐vaccine cost per dose of intervention to increase immunisation coverage by 1%.

The median non‐vaccine cost per dose of intervention to increase immunisation coverage by 1% for the 14 estimates was US $3.68 (all costs are reported in 2019 US dollars). In comparison, the average cost‐effectiveness was US $44.10, which is driven by three observations, Olken (2014) (US $23.10); Carnell (2014) (US $641.08); and Gurley (2020) (US $29.98).

**Figure 101:**
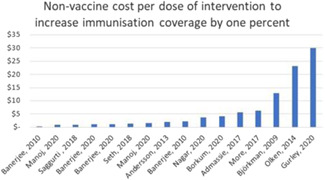
Non‐vaccine cost per dose of intervention to increase immunisation coverage by 1%.

Figure 101 illustrates that the range of estimates varied from a minimum of US $0.89 to a maximum of US $29.98. The cost‐effectiveness estimate for Carnell (2014) US $641 is excluded from the figure. The lowest cost per outcome was observed in Banerjee (2010) for the ‘camps with incentives’ intervention at US $0.89 per vaccibne dose to increase immunisation coverage by 1%. The highest cost per outcome was observed in Carnell (2014) at $641.08—where the 5‐year intervention included system‐building activities such as building and staffing a health post. In addition, the transparency of cost reporting in Carnell is very low, which further contributes to uncertainty in this cost estimate Indeed, Munk et al. (2019) dropped this study from their systematic review of costs because they judged the available cost information to be insufficient to calculate the cost per vaccine dose.

The cost‐efficiency of the interventions was measured by calculating the non‐vaccine intervention cost per vaccine dose. Cost‐efficiency of the interventions ranged from a minimum of US $3.87 per vaccine dose to a maximum of US $5449 per vaccine dose. The average cost‐efficiency was US $204.62 per vaccine dose (when the maximum value is excluded). The second‐highest estimate of the non‐vaccine intervention cost per vaccine dose was US $204.62. Thus the observed variation in cost‐efficiency is at least partly explained by including the most costly intervention, that is, from Carnell (2014), which included health‐system building activities (i.e., building and staffing a health post).

## 7 DISCUSSION

This systematic review synthesised the quantitative, qualitative, and cost evidence from 61 studies of different kinds of community engagement interventions in LMICs. The primary goal was to assess their impact on immunisation coverage and timeliness outcomes. We also considered the impact on intermediate outcomes along the causal chain, including caregiver knowledge and attitudes regarding immunisation, and how results vary by implementing agency and location (Review Question 2). In addition, we aimed to understand the mechanisms and processes through which change happens, by identifying factors relating to programme design, implementation, and context that were associated with better or worse outcomes along the causal chain (Review Question 3). Wherever cost data was sufficiently available, we also synthesised the evidence on cost effectiveness of community engagement interventions for immunisation outcomes (Review Question 4).

Based on the kind of community engagement interventions implemented and evaluated in relation to routine child immunisation outcomes, we identified three distinct types of interventions: (1) community engagement as the intervention (engagement is embedded), (2) community engagement in the design of the intervention, and (3) community engagement in the implementation autonomy of the intervention. In addition, there were a significant number of studies that fell into more than one engagement classification and were classified as having multiple engagement types.

We used quantitative meta‐analysis to combine the results of the IE, including sub‐group analysis to explore heterogeneity by intervention type, implementing agency, child sex, and other moderators. We conducted a detailed critical appraisal of the included IE, associated qualitative papers and cost evidence (wherever available) to assess the credibility of the results. From the included interventions, we identified 47 associated qualitative and 69 project reports that we used to address Review Question 3. We carried out deductive and inductive thematic analysis to synthesise the data. We found 22 studies with sufficient cost evidence data and we synthesised the evidence by estimating the non‐vaccine cost per dose of intervention to increase immunisation coverage by 1% as described in Ozawa et al. (2018).

### 7.1 Summary of main results

We reported quantitative results along the causal chain to address Review Questions 1 and 2, supported by the results from the qualitative thematic synthesis to address Review Question 3 and reported on the cost effectiveness of the interventions for immunisation outcomes to address Review Question 4. We started by presenting the results of the overall synthesis, followed by the individual results for the three kinds of community engagement interventions, wherever feasible.

#### 7.1.1 What evidence exists regarding the effectiveness of community engagement interventions in improving routine immunisation coverage of children in LMICs?

The evidence indicates that community engagement interventions had a small but significant positive effect on all the primary immunisation outcomes related to coverage and their timeliness. The findings are robust to exclusion of studies assessed as high risk of bias. The average pooled effect on full immunisation coverage in the RE model was an increase of 0.14 standard deviations units (95% CI: 0.06, 0.23) across all kinds of community engagement interventions. For antigen specific outcomes from BCG to DPT/pentavalent to measles the average pooled effects indicated an increase of 0.04–0.24 standard deviation in their coverage, which is equivalent to an estimated 1.6–5.6 percentile points increase in the coverage of intervention participants compared to the control group. The community engagement interventions also had positive and mostly significant effect on the timeliness of vaccinations and led to an average pooled increase of 0.15 standard deviation units (95% CI: 0.07, 0.24) in the timeliness of full immunisation. The community engagement interventions had a positive but nonsignificant pooled average effect of 0.03 (95% CI: −0.11, 0.16) on the DPT1–DPT3 dropout rate.

While the average effects were mostly positive and significant for the primary immunisation coverage and timeliness outcomes, the picture was mixed for intermediate secondary outcomes across the casual chain. While several intermediate outcomes were mapped on to our initial framework, they were not reported in any of our included studies or we did not have sufficient power to analyse them. These include community norms around immunisation, household norms and decision making, readiness to vaccinate, etc. Among the intermediate outcomes we were able to meta‐analyse only caregiver knowledge of and attitudes towards immunisation, vaccination card availability/retention, experience and satisfaction with health services and formal health worker supply, capacity and performance. Among the five, we found that community engagement interventions had a positive and significant increase of 0.19 standard deviation units (95% CI: 0.06, 0.46) in caregiver knowledge of immunisation. However, for the caregiver attitudes towards immunisation, though there was a positive increase of 0.14 standard deviation units, it was not statistically significant. There was no relationship between the interventions and vaccination card availability/retention, experience and satisfaction with health services or formal health worker supply, capacity and performance.

#### 7.1.2 Is there evidence for heterogeneous effects of community participation strategies (I.e., does effectiveness vary by region, population, gender or programme implementation)?

Though the significant and positive movement on immunisation‐related outcomes through community engagement is encouraging, it would be useful to get insights into what intervention types might work best for which outcomes of interest and where. Thus, below we examine more closely the impact of different kinds of community engagement interventions on the range of immunisation‐related outcomes, as well as some specific outcomes which are of general policy and programmatic interest.

We found that, among the four types of community engagement interventions, it was the engagement as the intervention (engagement is embedded) which had a more consistent positive and significant effect on the various primary immunisation outcomes. For example, participants in interventions with engagement as the intervention had an increase in full immunisation coverage of 0.08 standard deviations (95% CI: 0.03, 0.13) and in DPT3 coverage of 0.09 standard deviation (95% CI: 0.03, 0.15). We also found engaging the community in the design of the intervention had a positive effect on most primary outcomes related to coverage, except of DPT3.

Wherever possible, we sought to examine subgroups in cases where results of primary studies were disaggregated by group (e.g., child sex, socioeconomic status, etc.). For full immunisation (and DPT3), two studies reported disaggregated data for female children, which were included in the analysis. The estimated average effect of community engagement interventions on full immunisation for female children was −0.02 (95% CI: −0.10to0.07), meaning that there was a very small but nonsignificant reduction in vaccinations for girls. For DPT3 vaccinations, the estimated average outcomes for girls was 0.08 (95% CI: −0.09to0.25). While this was a very small increase, it was also not significantly different from zero. The effect was similar for boys DPT3 vaccination, with an estimated average outcome of 0.08 (95% CI: −0.09 to 0.25). Again, this was not statistically significant. All subgroup analysis results must be interpreted with extreme caution. Though none of the effects by child sex were statistically significant, in all cases the average effect is based on only two studies. More disaggregated data from primary studies is needed to explore true differences by child sex.

As discussed in Section 6.3.1 (full analysis presented in Supporting Information: Appendix I: Table 3) we also examined a set of moderators that might explain the variability among the effects. Analyses based on the RE model using independent effects revealed that publication year was the moderator that was most often significantly related to the size of the effect. However, this relationship was inconsistent among the individual outcomes. For example, in the case of mortality, newer studies actually reported higher effects, with an increase of 0.01 standard deviations per year. Even larger increases by year were seen for dropouts, with each additional year showing increases of 0.33 standard deviations in the average effect (recall that in our data set, all outcomes were recoded so that a positive number indicated a positive effect on the outcome, thus these increases in the effect are reflective of a decrease in vaccination dropout). But the negative relationship between publication year and the size of the average effect persisted for BCG vaccinations and DPT3 (showing reductions of 0.03 and 0.01 SDs per year respectively), and for OVP0 and OVP2 (showing reductions of 0.07 and 0.05 standard deviations respectively).

Results related to intervention duration were mixed. Increased exposure to interventions was related to increased effects for OPV0 and OPV2, such that each additional month of the intervention increased effects by 0.02 standard deviations. Child morbidity (proxied mostly by diarrhea) and mortality also showed a positive impact of intervention exposure, with each month of exposure increasing the effect by 0.02 standard deviation units (as with dropouts, a positive effect indicated a decrease in morbidity/mortality). Yet increased exposure to the intervention decreased the full immunisation effect by 0.003 standard deviation units for each additional month of the intervention. Meanwhile, increased evaluation period (time between end of intervention and data collection), while nonsignificant for most outcomes, was related to a decrease on average effects for DPT3, with a reduction of 0.01 standard deviation units for each month between the end of the intervention and the data collection. Thus, the positive effects on DPT3 tend to decrease over time in the post‐intervention period.

There were two outcomes for which study design was related to the size of the effect. For DPT3 vaccinations, quasi‐experimental studies reported smaller effects than RCTs by 0.15 standard deviations. However, for immunisation knowledge, effects of quasi‐experimental studies were larger than effects of RCTs by 0.44 standard deviation units. However, in this case, study design was perfectly confounded with region (all RCTs were in South Asia, and all quasi‐experimental studies were in Sub‐Saharan Africa), so we cannot parse out which is really contributing to this difference in effects.

#### 7.1.3 What factors relating to programme design, implementation, context, and mechanism are associated with better or worse outcomes along the causal chain? do these vary by the level of community engagement?

A recurring theme across the qualitative synthesis was the importance of accounting for *contextual factors* which could act as barriers or facilitators to immunisation. For instance, several studies note that limited availability of services, especially insufficient staff and vaccine supply, were dominant barriers to immunisation, affecting outcomes in the early portion of the causal chain. Other common barriers to immunisation included practical barriers faced by caregivers such as costs, largely indirect, and logistics (wait time and language barriers) or distance. There was more variation in barriers to related to social norms, fear, and an understanding of the importance of immunisations by type of engagement. Poor quality of services, including uninviting attitudes of health workers, posed a barrier to immunisation in communities that received engagement as the intervention or were engaged in the design of the intervention. These barriers may have resulted in poorer outcomes in the early portions of the causal chain. Interventions that even acknowledge or address these barriers are likely to be more effective in improving outcomes.

On the other hand, some studies also note that certain contextual factors could become facilitators of immunisation outcomes provided a study has adequately situated itself to leverage them. Across all engagement types, studies associated caregivers' awareness and perception of the benefits of vaccination with improved immunisation outcomes. Similarly, availability of health infrastructure and good quality of services were likely to have positive implications for intervention uptake and its intended impact.

In terms of *programme design*, intervention features or characteristics, across all engagement types, were often associated with cos intervention success or failure of an intervention. When it came to success, certain aspects of community engagement itself such as conducting stakeholder consultations, holding community dialogues or involving community leaders were associated with better immunisation outcomes. Studies also attributed intervention success to nonengagement intervention features including incentives given to caregivers, leadership and supportive supervision which improved overall health service delivery and health worker performance. Among the studies which attributed intervention failure to programme design, inadequate duration frequency or exposure to the intervention were the most notable reasons.

*Implementation* failures, such as low fidelity, were a common reason for intervention failure. Across all engagement types, most studies did not properly account for realities on the ground and were forced to change their implementation plans. These issues may have cropped due to exogenous or uncontrollable factors or may have been related to invalid theory of change assumptions. For instance, programme design may not have accounted for the unavailability of intended participants due to competing priorities. These issues, in turn, may affect the early portion of the causal chain such as beneficiary exposure to the intervention and its eventual uptake.

Beyond the contextual, programme design and implementation factors, the synthesis tried to tease out factors associated with *mechanisms of change*. Studies reported that certain uptake and fidelity challenges may have resulted in inefficient mechanisms for change. For example, administrative challenges, particularly related to technical issues and communication, were common. Issues with mobilising community members and recruiters were also common challenges. Thus, without adequate mobilisation, the mechanisms for change are not likely to function across the causal chain. There may be other mechanisms of change underlying the causal chain of an intervention. However, an extensive analysis was not possible due to limited reporting by studies on mechanisms of change and the factors influencing them.

#### 7.1.4 What is the cost‐effectiveness of different community participation interventions in improving children routine immunisation outcomes?

Among the studies for which we calculated cost‐effectiveness, we find that the median non‐vaccine cost per dose of intervention to increase immunisation coverage by 1% averaged US $3.68 (all costs are reported in 2019 US dollars) and the average cost‐effectiveness ratio was $44.10. There are three outlier observations that drive up average cost‐effectiveness: Olken (2014); Carnell (2014); Gurley (2020). The average cost per vaccine dose to increase immunisation coverage by 1% without the outliers was US $3.97. Outlier status can be a function of the intervention cost, or its impact or both its cost and impact since the incremental cost‐effectiveness ratio (i.e., the ‘non‐vaccine cost per dose of intervention to increase immunisation coverage by one percent’) includes both elements. Whereas Carnell (2014) is an outlier because the total project costs were extremely large relative to the other interventions included in the cost analysis, the cost‐effectiveness estimates for the Gurley (2020) and Olken (2014) studies were outliers because they reported among the smallest impacts (in terms of the absolute coverage change (intervention—control)). The impacts for both studies were positive but these differences were not statistically significant. In contrast to Carnell (2014), the estimated costs for interventions studied in Gurley (2020) and Olken (2014), that is, the non‐vaccine cost per vaccine dose for both studies is close to the median value in the range of cost estimates.

The range of cost‐effectiveness estimates varied from a minimum of $0.89 to a maximum of $641.08 (the second‐highest was $29.98) The cost‐efficiency of the interventions was measured by calculating the non‐vaccine intervention cost per vaccine dose. Cost‐efficiency ranged from a minimum of US $3.87 per vaccine dose for the reminders + incentives that Seth et al. (2018) studied to a maximum of US $5449 per vaccine dose for the intervention studied by Carnell (2014). The average cost‐efficiency was US $42.34 per vaccine dose (when the Carnell cost estimate is excluded). The observed variation in cost‐efficiency is at least partly explained by the inclusion of the Carnell (2014) intervention which included health‐system building activities (i.e., building and staffing a health post, which is costly when compared with interventions that offer a small incentive to families to bring their children to a health post for routine care, inclusive of vaccination, e.g. providing small in‐kind incentives in the setting of an immunisation camp.

### 7.2 Overall completeness and applicability of evidence

We identified 61 papers in LMICs. This is a growing evidence base, with almost 60 per cent of the included papers published within the last six years and about five ongoing studies identified. While this is a big evidence base in itself, the studies reported on a diverse range of immunisation outcomes. For outcomes like full immunisation, DPT3 and measles coverage, we could draw upon 28, 22 and 20 studies respectively for pooled effects. On the other hand, for outcomes on measles timeliness and DPT1 to DPT3 dropout we only had two and five studies respectively to assess the pooled effects. Thus, depending on the outcome of interest we had sufficient to limited evidence base from which to draw conclusions. Among the four kinds of community engagement interventions, for those with engagement as the intervention and multiple engagement types, we had a relatively larger evidence base, but for engagement in the implementation autonomy type it was quite limited.

Geographically, the evidence base is skewed towards India, representing one‐third of the evidence base. After India, Nigeria is the next biggest contributor to the evidence base with 8 studies. The evidence base is sparse or non‐existent for some countries where it is imperative to test effectiveness of community engagement interventions as they have low immunisation coverage rates indicated by DPT3 vaccination rates (WHO, 2020b). These include fragile countries like Democratic Republic of Congo and Central African Republic. In terms of regions, we identified no studies from North Africa or the Middle East and limited evidence from Latin America and the Caribbean and sparse evidence from East Asia and the Pacific region.

We assessed the external validity of studies primarily by drawing on the author's own discussion of factors such as study population, scale of the implementation, characteristics of the sample, and most importantly, the authors own discussions on generalisability. One argument that authors used to establish the external validity of their work is to highlight the heterogeneity of its treatment samples. This was particularly clear in Olayo et al. (2014); which reported that ‘…most of the components of the strategy were implemented and sustained in different socio‐demographic contexts’. We found that small sample or narrow sample sizes were the biggest challenge in establishing external validity, as in Biemba et al. (2016); which ‘…was limited to two districts and only six CHAs, making it difficult to fully generalise the results to the whole country’. Authors note that, while there were positive impacts with relatively small sample sizes, it may be hard to maintain delivery quality at scale (e.g., Borkum, 2014; Johri et al., 2020).

Several authors mentioned that these interventions can generalise to a broad range of settings as long as implementers consider the mechanisms of the theory of change, and how these may be adapted to different contexts (e.g., Gurley, 2020; Memon et al., 2015; Oyo‐Ita, 2020). That being said in some cases, strategies may generalise only to similar settings, especially in areas where demand‐side barriers may exist (Herrera‐Almanza & Rosales‐Rueda, 2018). For example, Pramanik et al. (2020) and Rahman et al. (2008) suggested their results would only apply in rural areas, while Morris et al. (2004) questioned whether their results would generalise to areas that were less severely disadvantaged. Studies where regions were purposively sampled for their low immunisation rates may not be generalisable to regions with higher immunisation rates (e.g., Demilew et al., 2021). Similarly, some interventions were implemented in areas with high mobile phone ownership and ubiquitous mobile‐money systems, and may not transfer to areas without similar resources (e.g., Gibson, 2017).

Most impact evaluation studies offered little to no information on factors relating to programme design, implementation, context, and mechanism that may be associated with better or worse outcomes along the causal chain. To supplement this knowledge gap, we identified additional documentation comprising qualitative studies, project reports, formative/process evaluations and observation studies for 39 of the 61 included IE. However, the 47 qualitative papers that we identified were associated with only 17 of the 61 IE. This has important implications for our synthesis. Non‐qualitative documentation such as observational studies and other project reports may reveal important information about the intervention activities, their implementation or the overall contextual trends. However, these reports are not typically set out to gain a deeper understanding of a variety of factors, including but not limited to contextual barriers or enablers, root causes of uptake or fidelity issues and the overall intervention mechanisms of change. Qualitative studies delve into these very aspects and help build an understanding of *why* an intervention may or may not have worked and for *whom*.

As for the cost‐effectiveness of different community participation interventions in improving children's routine immunisation, we find that it is infrequently estimated because the quality, reporting, and general availability of underlying cost data to estimate cost‐effectiveness is highly problematic.

Only 22 of 56 studies included in the systematic review reported any kind of cost information. Of those reporting cost information, only 14 evaluations were found to have the needed combination of cost and effectiveness data needed to estimate cost effectiveness: three studies did not report immunisation results in the format that was required and six studies had null or negative results, making the calculation of the cost‐effectiveness measure impossible. The resulting estimates are from a small, sub‐sample of studies that often use different cost inclusion criteria and different means to calculate total and incremental cost. The results should therefore be interpreted as estimates of the relative costs of alternative interventions—rather than absolute differences. Interpretation of these estimates should additionally take the scope of the intervention activities into consideration, as well as cost inclusion and exclusion criteria made in the reporting of costs.

### 7.3 Quality of evidence

The quantitative evidence was mostly low quality, though the randomised studies were generally of higher quality than quasi‐experimental studies. The randomised studies for the most part followed recommended allocation sequence methods ensuring comparability of intervention and control groups and that they have the same prognosis before the start of intervention. A majority of the quasi‐experimental studies were controlled before after studies and most of them either did not provide the required information or provided incomplete information on baseline balance between control and treatment arms or there was a high degree of imbalance at baseline leading to high risk or some concerns of selection bias. For most of the randomised and quasi‐experimental studies we identified concerns related to the way vaccination coverage outcomes were measured. This was mainly due to the use of caregiver reported (self‐reported) measures of vaccinations received by a child in the absence and/or incompleteness of immunisation cards, which is common in LMICs and is often influenced by the intervention itself. Risk of bias in measurement of self‐reported immunisation coverage outcomes could be ameliorated by priming the caregiver in both treatment and control groups such that recall is better. For example, checking about visits to the health centre or hospital at birth or in the first week of a child's life for vaccination, or corroborating self‐reports of BCG vaccination with the observation of a BCG scar on the child's arm. It would be useful if authors could discuss the steps that they took to minimise recall bias as data collection tools are not generally available to assess this. Transparency in reporting is more of an issue for quasi‐experimental studies as pre‐registrations of studies or analysis plans are less common than for randomised studies.

The quality of qualitative studies was relatively high. Although few (5) scored strong on all key elements, most studies received strong scores on the majority of key elements. The most common elements to be missing were sample characteristics and analytic methods. Better reporting on these could significantly improve the quality of the evidence base.

The quality of the cost evidence of the 22 studies that included any kind of cost evidence was mixed. Our risk of bias assessment indicated that about half of the included evaluations carried out a planned, organised, cost analysis which included stating the form of economic evaluation; indicating the perspective of the costing; and describing the method of cost data collection. Indications of the quality of underlying cost data also are mixed—just over half (12) of the 22 studies reviewed in the cost analysis used expenditures (rather than budgets) to produce total cost estimates, whereas other studies simply did not report the source of cost data. About half of the included cost studies had high quality, detailed description of costs and cost analyses—which is essential for determining the comparability of total and average cost estimates. We expect the quality of cost evidence to improve as donors and other research‐granting agencies demand high‐quality, transparent cost evidence when funding IE of development interventions.

### 7.4 Limitations and potential biases in the review process

There are several limitations of this review related to both the existing evidence base in this area and the synthesis approach.

#### 7.4.1 Limitations of the existing evidence base

1. Statistical power for the meta‐analyses and heterogeneity analysis: Our ability to make strong conclusions on the relative effectiveness of the different types of community engagement interventions was limited by the number of studies looking at each intervention type and outcome of interest. In addition, we were unable to undertake the full moderator analyses to explore heterogeneity quantitatively for all outcomes due to a limited number of included studies in each intervention type.
2. Lack of precision estimates in primary studies: The biggest challenge we faced in quantitative analysis was the lack of a precision estimate in a significant portion of studies. Authors often presented coefficients without their associated standard errors, and just an asterisk indicating significance rather than an exact *p*‐value. These reporting practices make it difficult to determine exact effect estimates. In many cases, we had to estimate *t*‐values based solely on the asterisk indicating significance, as described in our methods section. With a maximum assumed value of 2.8 (or −2.8, depending on the direction of the effect), we are likely underestimating the impact of these interventions given that most of the effects were positive and in reality, *t*‐values can certainly exceed 2.8.
3. Publication bias in primary studies: It is typical that positive results are more likely to be published than negative ones, which is why testing for publication bias is important in the context of any systematic review. We conducted analysis for publication bias for quantitative results, and generally had sufficient evidence to test when intervention types were combined. However, limited evidence was discerned for subgroup analysis by engagement type because few intervention types were sufficiently powered for this test. This limits our ability to interpret heterogeneity, as it is still unclear whether the variation among effects is being driven by publication bias. More rigorous IE are needed to have a sufficient evidence base to properly assess publication bias. However, no such analysis was conducted for the qualitative portion of this study because it is not feasible while working with nonquantifiable constructs.
4. Cost‐effectiveness analysis: We aimed to undertake a full‐scale analysis of the cost‐effectiveness of the included set of interventions (Review Question 4), however we were limited by the available cost data.
5. Most of the community engagement interventions are in combination with other intervention components. Therefore, its unique contribution to changes in outcomes may be difficult to establish.

#### 7.4.2 Limitations of the synthesis process

6. The screening of studies for inclusion into our systematic review was based on the description of the community engagement aspects of the intervention. There may be studies which might have fit our inclusion criteria, for example the community may have been consulted on the design or delivery of the intervention, but because of inadequate reporting might have got excluded. Thus, we may have excluded papers that should ideally have been included.
7. It is possible that our process of choosing the author's preferred model could potentially exacerbate reporting bias, if author's preferred models are biased towards finding an effect. However, we do examine outcome reporting bias in the risk of bias assessments, and test for publication bias when the number of studies allow, so we perceive this risk to be quite low.
8. The authors own biases could affect reporting, particularly of qualitative results. Qualitative coding requires the selection of portions of text that are thought to represent themes and the organisation of these themes. However, this process can be subjective as it requires the interpretation of the text and value judgements about the importance and representativeness of quotes.
9. A limitation of the cost‐effectiveness analysis is that it does not explicitly take into account the baseline level of immunisation in the targeted population. This is important because it is typically more costly to immunise children where the baseline levels already are very high: it is more expensive to reach ‘last‐mile’ children. The baseline levels of immunisation are important for interpreting and comparing cost‐effectiveness analyses of different development interventions.

### 7.5 Agreements and disagreements with other studies or reviews

This systematic review is the first that we are aware of to use a detailed framework of community engagement interventions for assessing their effectiveness for immunisation outcomes in LMICs. The findings from the review are broadly consistent with one review which is focused on community monitoring interventions to curb corruption and increase access and quality of service delivery in LMICs. This high‐quality review by Molina and colleagues (2016) found positive effects of community monitoring interventions on immunisation coverage. A medium quality narrative synthesis by Gilmore and McAuliffe (2013) examined the effectiveness of preventive interventions delivered by community health workers for maternal and child health in LMICs on essential new born care and found some evidence in its support, but found the evidence base to be insufficient to draw firm conclusions.

Like our review Molina and colleagues (2016) also found that effects tended to diminish further along the casual chain. They found that the community monitoring interventions reduced child mortality, but this effect was statistically insignificant. The review of community driven development programmes by White and colleagues (2018) also found that effects tended to diminish further along the causal chain, such as programmes were often ineffective in improving wellbeing outcomes.

## 8 AUTHORS' CONCLUSIONS

### 8.1 Implications for policy and practice

Generally, community engagement interventions were successful in improving immunisation outcomes. The effects were robust to exclusion of high risk of bias studies. The effects were also uniform across geographies and baseline immunisation rates. However, some engagement approaches, like those with embedded community engagement, appear to be more effective than the others. We also found that certain features of interventions may contribute to their success as discussed below.

#### 8.1.1 Appropriate intervention design, including embedded community engagement approaches, can lead to intervention success

It was reassuring that the dominant reasons for project success were positive intervention features and not external factors. This implies that stakeholders can influence the success of their project and do not have to rely on random chance. Positive intervention features included local, supportive supervision; incentives for health care workers or caregivers; and health system integration and organisation. Many studies cited the effectiveness of the engagement strategy as a reason for the project success: dialogues developed into action plans (Andersson et al., 2009) and needs assessments resulted in practical adjustments (Modi et al., 2019). Policy makers and implementers can attempt to integrate these positive intervention features into their projects.

Methods for achieving sufficient intervention exposure should be integrated into the design of interventions. Some mull impacts were attributed to inadequate exposure to the intervention. However, in the quantitative analysis, we did not find a significant impact of intervention exposure on the size of the effects. These intricacies may not be captured in simple quantitative measures of exposure to intervention in months. Nonetheless, some interventions may require a longer implementation period to build community trust and buy‐in which may be essential for its effectiveness to be visible.

#### 8.1.2 Accounting for local context, including addressing common barriers of immunisation and leveraging facilitators may be useful in designing new interventions

Projects tended to fail because policy makers and implementers did not account for existing contextual constraints or uncontrollable trends. This finding is somewhat less positive because these external factors, such as those with political instability, may be impossible to overcome in some settings. Nevertheless, ongoing monitoring or understanding of context can help in risk mitigation by making necessary modifications to the intervention design or implementation mid‐way.

Other constraints, like social norms or weak health systems, may be addressed in intervention design. For instance, the sensitisation of heads of households may address constraints imposed by social norms about decision making in the utilisation of health care services (Oche et al., 2011). Shukla (2018) shows that an intensive participatory strategy can be used to strengthen health system. Many community engagement interventions target awareness raising, although it is was not a commonly identified barrier to immunisation uptake.

Building on existing facilitators to immunisation may increase intervention impacts. Interventions which leverage facilitators may be more successful as a result of the presence of these facilitators but are seriously threatened if the facilitators prove to be absent. For example, ‘good’ existing health care services can be leveraged in the implementation of community engagement activities; however, if the health care infrastructure turns out to be ‘not as good’ as originally assumed, then the intervention activities will fail to achieve their objectives. High maternal education may be leveraged in communication activities through the distribution of written materials. But, the distribution of written literature in areas where maternal education is low will be unsuccessful (‘Barriers to immunisation’ section).

#### 8.1.3 Implementation challenges could be avoided through appropriate intervention design

Policy makers and implementers should conduct scoping work to ensure that the interventions are practical to implement. Many interventions failed to account for existing implementation constraints and practicalities on the ground, such as limited cellphone service and insufficient staffing levels. This resulted in serious interruptions to implementation and low implementation fidelity (‘Uptake and fidelity challenges’ section) leading to inefficient use of limited resources. Wherever possible, existing implementation constraints should be accounted for in the design of interventions and should be addressed before implementation, rather than used to justify why interventions failed to reach their goals. Administrative challenges, such as delayed approvals from local authorities, can be overcome or avoided through designs that include close collaboration with local partners.

### 8.2 Implications for research

While we found significant heterogeneity across a multitude of immunisation‐related outcomes, there were inconsistent effects related to moderators that might explain the variability among the studies. While we would typically expect risk of bias assessments to be significant predictors of differences in effect sizes, as RCTs tend to have smaller effects than non‐randomised studies across several different literatures, we did not find this pattern in our review. The immunisation literature may be unique in this case for several possible reasons. First, unlike some sectors, immunisation outcomes were measured in virtually the same way in both RCTs and quasi‐experimental studies, creating some level equivalence. Additionally, in the case of immunisation specifically, both RCTs and QEDs are more likely to have high risk of bias, primarily due to the reliance in both study designs on caregiver recall for the measurement of the outcome. The risk of bias analysis has shown that, for the randomised studies, while the confounding bias is relatively low, researchers should rely less on self‐reported outcome measures of vaccination coverage, which are more susceptible to biases, and/or validate them using administrative data, wherever feasible as was done in a recent study in India (Banerjee et al., 2020). A majority of non‐randomised studies were controlled before after designs and had a high risk of selection bias and confounding, due to incomplete or omission in reporting of baseline balance or having high baseline imbalance. There are therefore opportunities to conduct these studies with more rigorous evaluation designs. There are concerns related to reporting, in particular there is a lack of transparency with regard to how authors addressed potential contamination or how authors responded to implementation problems (e.g., attrition).

Researchers should consider the following when undertaking IE in this area:

**Better reporting of interventions**: In many cases, we had difficulties in identifying precisely what the impact evaluation was evaluating due to limited reporting of the intervention components and characteristics. This is more of an issue in quasi‐experimental studies than randomised studies. In addition, most studies lacked or had inadequate description and discussion of the intervention theory of change. This limits the amount of learning that can take place from the studies, for implementers who may wish to take the intervention to a new setting or for synthesis work. Authors should consider drawing on tools such as the TIDieR intervention reporting guidelines for health (Hoffman et al., 2014).

**Better reporting for quantitative risk of bias assessment**: In several areas reporting of information in the studies needs to improve, especially for quasi‐experimental studies: (a) the number of participants in various stages of evaluation and loss to follow‐up (attrition) needs to be more clearly reported and discussed; (b) potential contamination or spillovers needs to be reported better, especially description of geographic separation of treatment and control clusters (where applicable) or transfer of health administrators and health workers across intervention arm; (c) potential performance bias or its absence needs to be reported and discussed better.

**Consideration of equity**: There is a lack of research on how community engagement interventions affect immunisation outcomes by gender, income or for hard to reach populations. For example, few IE undertook sub‐group analysis for these groups or undertook parallel qualitative research to understand how women or marginalised or vulnerable populations are affected by these interventions or their perspectives. Sub‐group analysis needs to be incorporated right at the beginning of the evaluation and not as an afterthought. More sex‐disaggregated data (or any other disaggregated data by characteristic of interest, such as socio‐economics status, religion, etc.) is needed to draw valid conclusions about potential sex differences in the impacts of community engagement interventions on immunisations.

**Prioritization of mixed‐methods IE**: Few studies incorporated qualitative research that would allow them to uncover the mechanisms that lead to the success or failure of the intervention. Drawing from qualitative work in mixed methods studies, evaluators should be sure to report on *why* they think their interventions worked, not just *if* the interventions worked. This will ensure the best use of their research in informing future policy and practice.

**Greater focus on intermediate outcomes**: To understand the mechanisms behind effectiveness or non‐effectiveness of interventions it is important to know how the interventions are affecting not just the final immunisation outcomes, but also the intermediate outcomes which precede them in the theoretical causal chain. However, IE typically do not focus on intermediate outcomes and this limits the learnings on the mechanisms of change. This can be addressed relatively easily by collecting quantitative and qualitative information on the intermediate outcomes and analysing them along with the final outcomes.

**Improved and standardised reporting of cost data and analysis**: Detailed cost data tables, with the clear descriptive information about the data, methods, unit costs of resources used, and cost adjustments (e.g., currency, base year, inflation and discounting adjustments) that may have been performed on the cost data is not currently standard practice, but should be. Reporting of total costs, average costs, and marginal costs per impact all should be reported for any evaluated programs. There is a wide range of available guidance to assist researchers in this reporting. For example, the standards described in Chapter 3 of Drummond et al. (2015) would be a useful place to get detailed information on how to conduct and reporting a high‐quality cost analysis. The fundamental challenge is with the lack of disaggregation of costs, and the low transparency of underlying cost data—obtaining high quality data requires planning from the start of the evaluation—when the research questions are formed and the plans for data collection are being made. In addition, the collection of cost data is most effectively accomplished during implementation, as resources are expended, rather than after the program has ended—when the data are more subject to recall bias.

**Donors play a key role in driving the demand and allocating needed budget for sub‐group and cost analysis in IE**: Research teams are more likely to plan and report on sub‐group and cost analysis when the when the donor requires it—starting with a request for the relevant information in the RFP and when donors enforce its reporting at program's end.

**Bigger scope of future SR on effectiveness of community engagement interventions**: In this review we focused on the effectiveness of community engagement interventions on only immunisation related outcomes. Though we considered child health outcomes like morbidity, it was limited to those like diarrhea which could be affected by immunisations or could be a proxy for them. Typically, community engagement interventions in health sector aim to improve not only immunisation outcomes but a whole spectrum of maternal and child health outcomes. It would be useful for a future systematic review to assess its effectiveness on the full range of maternal and child health outcomes to provide a fuller picture of their effectiveness and policy relevance.

## CONTRIBUTION OF AUTHORS

The review team comprises Monica Jain (MJ), Shannon Shisler (SS), Charlotte Lane (CL), Avantika Bagai (AB), Elizabeth Brown (EB), Mark Engelbert (ME), Yoav Vardy (VY), John Eyres (JE), Daniela Anda Leon (DL) and Shradha Parsekar (SP). MJ, AB and ME have content knowledge in childhood immunisation programmes in low and middle income countries MJ and SS are quantitative methods experts, with over a decade of experience designing, managing and analysing quantitative research, including meta‐analyses. AB, DA and SP have prior experience with screening and coding data from studies. CL and AB have experience with qualitative research. YV is a qualitative researcher with considerable experience in carrying out appraisal of qualitative evidence. EB is a costing analysis expert. JE is an expert in information retrieval, with decades of experience in supporting information retrieval for systematic reviews.

**Content**: The content of the review has been developed by MJ, SS, CL, AB, EB and ME.

**Systematic review methods**: The review methods were drafted by MJ, SS, CL and AB.

**Statistical analysis**: The statistical analysis was overseen by SS with input and quality assurance from MJ. Effects data were extracted by DA and a team of independent consultants with quality assurance from MJ and SS, while outcome classification and grouping were undertaken by MJ, SS, DA and AB.

**Qualitative synthesis**: CL and AB oversaw the qualitative synthesis with input and quality assurance from MJ. The critical appraisal of qualitative evidence was undertaken by YV with input from MJ, CL and AB.

**Cost effectiveness synthesis**: EB carried out the cost effectiveness synthesis with input from MJ.

**Information retrieval**: The search strings were developed and executed by ME and JE. Grey literature review, snowballing and reference checks were developed by ME and carried out by AB, SP and a team of independent consultants.

## DECLARATIONS OF INTEREST

The International Initiative for Impact Evaluation (3ie) provided funding and technical assistance for seven impact evaluations of community engagement interventions for immunisation as a part of its immunisation evidence programme. This technical assistance included, but was not limited to: reviewing study designs, analysis plans, and data collection instruments; advising research teams on how to improve study components and address challenges that arise during the course of the evaluation; and supporting grantees in engaging with stakeholders to promote uptake and use of evidence generated by the evaluations.

As members of 3ie staff, authors Monica Jain, Avantika Bagai and Mark Engelbert, have all had varying levels of involvement in reviewing proposals for these evaluations and providing research teams with technical assistance. Several procedural safeguards and transparency measures were put in place to mitigate the risk this conflict of interest imposed. First, all candidate studies, including those funded by 3ie, underwent a rigorous multi‐step screening process, including review at the title, abstract, and full‐text levels. To qualify for inclusion in the SR, a study was judged to meet the inclusion criteria related to study design, outcomes and population by two independent screeners who have reviewed the full text of the study. The 3ie core team was responsible for assessing whether the study candidates met the inclusion criteria for community engagement because of the complexity of the framework. However, these authors have no financial interests in this area and have not published any prior reviews on the topic. The remaining study authors have no conflict of interests to declare.

## PLANS FOR UPDATING THIS REVIEW

The authors will undertake, or contribute to, updates once resources are identified and further rigorously evaluated studies become available.

## DIFFERENCES BETWEEN PROTOCOL AND REVIEW

1. In the report, we have replaced the word ‘participation’ for ‘engagement’, a change that is also reflected in our review title and objectives. Though the IAP2 framework uses the terms ‘public participation’ and ‘community engagement’ interchangeably, we realise that this may lead to confusion as there may be inter researcher differences in how these terms are perceived and used. Therefore, for maintaining consistency, we have used *community engagement* throughout the report and avoided any references to community participation. From a linguistic perspective, these words are used interchangeability in our review, as is evident from our protocol (please refer to pp. 1–3). Therefore, this change does not alter the scope of our review in any way.
2. In the protocol we had proposed to use the IAP2 spectrum of community engagement for determination of interventions to be included in the review. However, after pilot testing of the IAP2 framework on a range of community engagement interventions, we found that most interventions are based on a more ‘utilitarian perspective’ put forth in Brunton et al. (2017). Thus, for this review we ultimately settled on the community engagement framework which focuses on process of engagement rather than its intensity. Our approach corresponds to some degree to the ‘extent of engagement’ part of the conceptual framework developed in Brunton et al. (2017) and we also kept the spirit of IAP2 framework by including interventions in which engagement goes beyond one‐way communication, that is, beyond the inform level of IAP2, to include some consultation or dialogue with the community or some decision making by the community members. We found this approach to be relatively easier to apply, less subjective, less prone to classification error and potentially useful to practitioners. In the section on objectives, we have clubbed what were originally Review Questions 3 and 4 in the protocol (p. 5). These questions related to understanding how implementation features and contextual factors such as barriers and facilitators to immunisation, are associated with relative success or failure of community engagement interventions. When we set out to answer these questions through our qualitative synthesis, we realised that all these factors are interlinked and, in various combinations, may influence the outcomes along the causal chain. Therefore, we combined the two objectives and have presented our analysis and results accordingly. This modification to the review objectives does not alter our study's scope.
3. We had intended to include the relevant Chinese literature in the review. However, because of political disruptions in Hong Kong, it did not materialise.
4. We have added many more secondary outcomes as per our evidence gap map framework and also their categorisation has changed. But data was not available in primary studies for most of them, so they could not be analysed in this review.
5. In the protocol we stated that ‘When multiple papers report different results on an identical outcome, we will contact the authors to enquire about the differences and choose the results that more accurately reflect the impact of the intervention as relevant to our research questions. If contacting the author does not yield a clear decision, we will use results from the paper with the latest publication date.’ However, due to the large scope and limited resources of our review, we instead used results from the most recent paper.
6. In the protocol, we stated that ‘Where multiple outcomes are reported from different specifications, we will select the specification with the lowest risk of bias in attributing impact, for example, the most appropriately specified outcomes equation.’ However, given that risk of bias could not be assessed until after inclusion decisions were made, we instead used the authors preferred specification. If this was not explicitly stated, but the authors reported one specification as the ‘main results’ and other specifications as ‘robustness checks’ we used the ‘main results’ specification. If neither of these applied, we used the specification with the most controls.
7. In the protocol we stated that ‘When multiple treatment arms, we will divide the control group by the number of treatment arms.’ Instead, we chose independent effects, choosing either (a) the treatment arm with the most components, (b) the treatment arm that was identified by the authors as the one that would be scaled up, or (c) when number of components was equal, the arm that was the most cost effective (e.g., choosing unconditional cash transfers (UCTs) over conditional cash transfers as the administrative costs related to UCTs are significantly lower).

## SOURCES OF SUPPORT

This study was made possible by a grant from the Bill & Melinda Gates Foundation to the International Initiative for Impact Evaluation (3ie).

## Supporting information

Supporting information.

Supporting information.

## Footnotes

1 These criteria are adapted from Snilstveit and colleagues (2015, pp. 13–14), who in turn draw on Gartlehner and colleagues (2006) and Thorpe et al. (2009). See also Cameron and Mishra (2016).

2
https://data.worldbank.org/indicator/PA.NUS.FCRF

3 A custom table was extracted from https://data.bls.gov/cgi-bin/dsrv?cu

4 The methods for conducting an integrated full systematic review of economic evidence are under development. A description of the full SR of EE here; also see https://training.cochrane.org/cochrane-handbook-systematic-reviews-interventions.

5 Due to the large volume of studies excluded, presenting a table on characteristics of excluded studies was not feasible. The list of excluded impact evaluations, that is, those that did not meet the community engagement criteria, is included in the Supporting Information: Appendix.
