# Supplementary material for: Use of community engagement interventions to improve child immunisation in low‐ and middle‐income countries: A systematic review and meta‐analysis
Source: Campbell Syst Rev. 2022 Jul 27;18(3):e1253. doi: 10.1002/cl2.1253 (PMC9359116; doi:10.1002/cl2.1253)
Supplement: Supplementary file 1 — Supporting information. [file CL2-18-e1253-s001.docx]

**Appendixes**

Contents

[Appendix A: International Association for Public Participation (IAP2) framework 2](#_Toc108537408)

[Appendix B: Definitions of outcomes included in the review 2](#_Toc108537409)

[Appendix C: Applied search terms for the electronic searches 9](#_Toc108537410)

[Appendix D: Qualitative search protocol 14](#_Toc108537411)

[Appendix E1: Quantitative data extraction tool 17](#_Toc108537412)

[Appendix E2: Cost effectiveness data extraction tool 23](#_Toc108537413)

[Appendix E3: Qualitative coding tool 30](#_Toc108537414)

[Appendix F1: Quantitative risk of bias assessment tool 59](#_Toc108537415)

[Appendix F2: Qualitative risk of bias appraisal tool 78](#_Toc108537416)

[Appendix F3: Critical appraisal tool for cost evidence 86](#_Toc108537417)

[Appendix G: Table 1: Characteristics of the included studies 88](#_Toc108537418)

[References to included studies 92](#_Toc108537419)

[Included qualitative studies 98](#_Toc108537420)

[Included other studies 101](#_Toc108537421)

[References to ongoing studies 106](#_Toc108537422)

[References to excluded studies 106](#_Toc108537423)

[Appendix H: Table 2: Distribution of the quality of the 47 papers evaluated for risk of bias 121](#_Toc108537424)

[Appendix I: Table 3: Summary of all moderator analyses 124](#_Toc108537425)

[Appendix J: Qualitative synthesis – hierarchy charts and representative quotes 132](#_Toc108537426)

[Appendix K: Sensitivity analysis for qualitative evidence 172](#_Toc108537427)

##
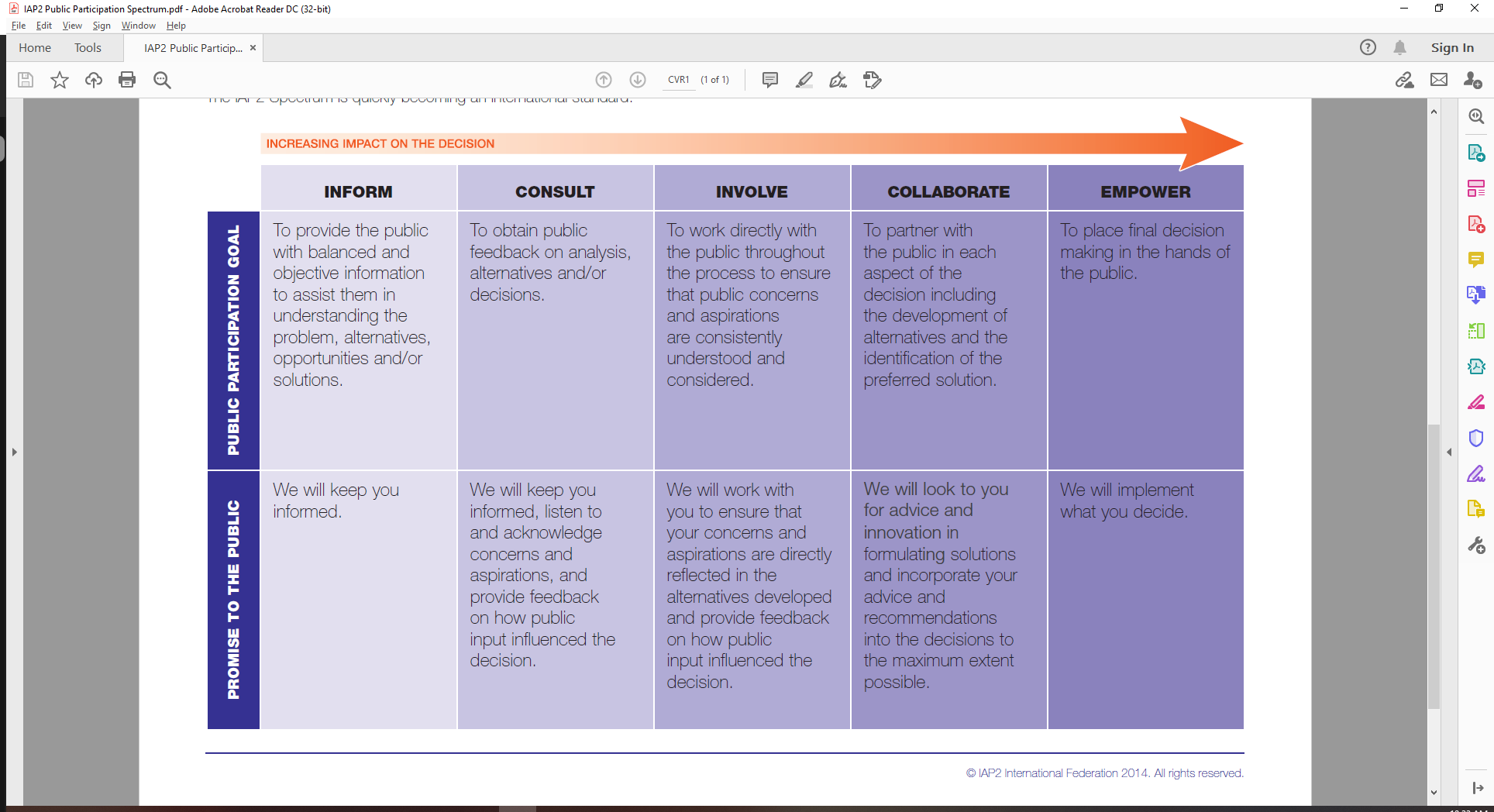
Appendix A: International Association for Public Participation (IAP2) framework

## Appendix B: Definitions of outcomes included in the review

| HAA. Thinking and feeling (attitudes, confidence) | | | HAA1. Knowledge about immunisation | | | Caregivers’ knowledge about immunisation in general (i.e., its purpose and role in preventing disease) | | |  |
| --- | --- | --- | --- | --- | --- | --- | --- | --- | --- |
|  |  |  | HAA2. Attitudes about immunisation | | | Caregivers’ attitudes towards immunisation in general (i.e., whether they view it favourably or unfavourably or have high or low confidence in its efficacy) | | |  |
|  |  |  | HAA3. Attitudes about health providers | | | Caregivers’ attitudes about health providers in general (i.e., whether they generally trust health providers to provide high-quality and appropriate care) | | |  |
| HAB. Social processes | | | HAB1. Community norms | | | Community-level attitudes and beliefs about immunisation, including whether there is social pressure to vaccinate or not vaccinate. This can be measured either objectively through aggregating community-level responses or subjectively by soliciting individual community members’ beliefs about the norms in their community. This includes attitudes and beliefs about immunisation of key influencers in the community like traditional or religious leaders. | | |  |
|  |  |  | HAB2. Household norms & decision-making | | | Norms and practices determining who in a household (e.g., mother, father, mother-in-law) provides input to decisions about whether to vaccinate, and how much decision-making power individual household members have. This also covers attitudes towards immunisation of household members other than the primary caregiver. | | |  |
| HAC. Readiness to vaccinate | | | HAC1. Readiness to vaccinate | | | Caregivers’ motivation, intention and plan to vaccinate their children. Note this is more specific than general attitudes towards immunisation covered under HAA2 | | |  |
|  |  |  | HAC2. Reasons for not vaccinating | | | Caregivers’ stated reasons for not vaccinating children. This may include factors such as convenience (which would also be coded under “perceived convenience of vaccination”), but only use this code if the factors mentioned are specifically framed as reasons for not vaccinating. Also, only use this code when the study measures *effects* of the intervention on this outcome. If the study gathers data on reasons for not vaccinating but does not provide an effect size for this as an outcome, use the cross-cutting theme but not this outcome code. | | |  |
| HAD. Practical factors | | | HAD1. Awareness of place, time, schedule for vacc. | | | Caregivers’ knowledge about when and where they should go for vaccinations. | | |  |
|  |  |  | HAD2. Actual cost of vaccinating | | | Actual cost of vaccinating the child, including vaccine cost, transportation cost, loss of wage/income due to missed work, and providing gifts/unofficial payments to the health providers | | |  |
|  |  |  | HAD3. Perceived convenience of vaccination | | | Subjective measures (i.e., caregivers’ beliefs) of the convenience of taking the child for vaccination, such as opportunity costs of vaccinating a child (e.g., not able to care for a younger child), long lines at health clinic, and inconvenient day/time of vaccination. | | |  |
|  |  |  | HAD4. Experience and satisfaction with health services | | | The actual experience of health services in the last visit such as duration of waiting time, availability of vaccine or vaccinator, and behaviour of the health staff (respect, rudeness). This also includes level of satisfaction with the health services, professionals and facilities. | | |  |
|  |  |  | HAD5. Vaccination health card availability/ retention | | | Measures of whether caregivers possess vaccination health cards provided by the health system, and/or whether caregivers can show the vaccination health card. | | |  |
|  |  |  | HAD6. Perception of vaccination side effects | | | Caregivers’ perceptions of the likelihood and severity of side effects from vaccination, and their knowledge of how to recognise and treat normal side effects. | | |  |
| IA. Health workforce | IAA. Comm. health workers | IAA1. Community HW motivation, capacity & performance | | | Any measure of CHWs’ capacity to deliver quality and timely vaccination services. | | | |  |
|  |  | IAA2. Supply of CHWs | | | The total availability of CHW services in the community, taking into account both the number of CHWs and the time they have available. | | | |  |
|  | IAB. Vaccinators | IAB1. Formal HW supply | | | The total availability of FHW services in the community, taking into account both the number of FHWs and the time they have available. | | | |  |
|  |  | IAB2. Availability of HWs at vacc. point of service | | | Whether vaccinators are present at vaccination point of service (e.g., health clinic) when vaccination services are supposed to be offered. This includes measures of health worker “absenteeism”. This is different from perception or experience of health workers from caregivers’ point of view. The source of information for this outcome can be administrative data or survey of health facilities and staff. | | | |  |
|  |  | IAB3. Formal HW motivation, capacity & performance | | | Any measure of FHWs’ capacity to deliver quality and timely vaccination services, or of their performance in doing so. | | | |  |
|  | IAC. Administrators | IAC1. Admin staffing | | | The number of staff in administrative posts in the health system (i.e., those not directly involved in health service provision). | | | |  |
|  |  | IAC2. Capacity of health admin. responsible for vaccination | | | The knowledge, skills, and motivation of staff in administrative posts in the health system, including leadership positions. | | | |  |
| IBA. Health information systems | | IBA1. Immunisation data collection (quality, completeness) | | | The health system’s capacity for and success in collecting data about vaccination coverage and service quality for regular monitoring and accountability. | | | |  |
|  |  | IBA2. Defaulter tracing | | | The health system’s capacity for and success in identifying vaccination “defaulters” (i.e., those whose children receive early vaccine doses but do not return for subsequent vaccinations). | | | |  |
|  |  | IBA3. Supply chain management | | | The health system’s capacity to monitor the supply of vaccines at points of service, ensure reliable supply chains, and avoid supply “bottlenecks”. | | | |  |
|  |  | IBA4. Immunisation data availability/ transparency | | | How easy it is for stakeholders within and beyond the health system to access data about vaccination service quality, coverage and timeliness. | | | |  |
| ICA. Vaccine availability | | ICA1. Stockouts | | | The frequency and duration of incidents when vaccines are out of stock at points of service, or when vaccines are in stock but cannot be administered to children who are brought in (e.g., because health workers are instructed not to open a new vial if there are not enough children to receive all doses in the vial, meaning that some of the vaccine would go to waste). Source of this information is administrative data and/or survey of health facilities and staff | | | |  |
|  |  | ICA2. Quality of cold chain infrastructure | | | The availability, quality and upkeep of physical equipment and place for cold chain storage of vaccines | | | |  |
| IDA. Resources | | IDA1. National or sub-national vaccine financing | | | Change in financial resources for national or sub-national vaccination programmes, policies or strategies. | | | |  |
|  |  | |  |  | | |  |  | |

| JAA. Vaccination coverage | JAA01. Full routine immunisation for children | Binary measure of whether or not children have received all routine vaccinations for the relevant country or region. |
| --- | --- | --- |
|  | JAA02. BCG | Binary measure of whether or not children have received the BCG vaccine. This may be measured by checking whether children have a BCG vaccination scar. |
|  | JAA03. DPT1 | Binary measure of whether children have received first dose of the DPT/penta vaccine. If study does not specify which doses were included in the outcome measures (i.e., the outcome is just “DPT/pentavalent vaccination” then apply this code unless it is clear they are talking about full DPT/penta vaccination, in which case code it as JAA05: DPT3. |
|  | JAA04. DPT2 | Binary measure of whether or not children have received the second dose of the DPT or pentavalent vaccine |
|  | JAA05. DPT3 | Binary measure of whether or not children have received the third dose of the DPT or pentavalent vaccine. If the study does not specifically say “DPT3” (or “pentavalent 3”), but refers to “complete DPT/penta vaccination”, then use this code. |
|  | JAA06. OPV0 | Binary measure of whether children have received 1^st^ dose of oral polio vaccine (recommended for administration at birth). |
|  | JAA07. OPV1 | Binary measure of whether children have received 2^nd^ dose of the oral polio vaccine (recommended for administration at 6 weeks). |
|  | JAA08. OPV2 | Binary measure of whether children have received the 3^rd^ dose of the oral polio vaccine (recommended for administration at 10 weeks). |
|  | JAA09. OPV3 | Binary measure of whether children have received the 4^th^ and final dose of the oral polio vaccine (recommended for administration at 14 weeks). |
|  | JAA10. IPV | Binary measure of whether children have received inactivated polio vaccine, given as injection. Countries differ in their guidelines/practices regarding IPV, so in the description cell of the codebook, please note the number of doses and age(s) when administered. |
|  | JAA11. Measles | Binary measure of whether or not children have received the measles vaccine |
|  | JAA12. No vs. partial routine immunisation | Proportion of children who receive at least one vaccination versus those who are completely unvaccinated. |
|  | JAA13. Vaccination timeliness | Proportion of vaccinations delivered on time according to the recommended schedule, vs those that are delivered late. |
|  | JAA14. Drop out rate for multi-dose vaccines | Proportion of children who fail to receive the complete course of a multi-dose vaccine (DPT/penta, OPV, or in some cases measles) after receiving the first dose. |
|  | JAA15. Vaccination coverage (unspecified) | Use this code if the evaluation or SR refers to impacts on routine vaccination coverage for children, but without specifying which vaccines. |
| KAA. Health outcomes | KAA1. Childhood morbidity | Incidence of vaccine-preventable diseases or symptoms associated with those diseases (e.g., diarrhoea) among children under 5. |
|  | KAA2. Neonatal/Infant/ Child Mortality | Incidence of mortality among children below five years from all causes. Neonatal mortality refers to death of a live-born baby within the first 28 days of life. *Infant mortality* is the death of young children under the age of 1. Child mortality, refers to the mortality of children under the age of five. This category includes all-cause mortality. |

## Appendix C: Applied search terms for the electronic searches

**Search terms**

*L&MICs*

2. (Afghanistan or Albania or Algeria or Angola or Argentina or Armenia or Armenian or Azerbaijan or Bangladesh or Benin or Byelarus or Byelorussian or Belarus or Belorussian or Belorussia or Belize or Bhutan or Bolivia or Bosnia or Herzegovina or Hercegovina or Botswana or Brazil or Bulgaria or Burkina Faso or Burkina Fasso or Upper Volta or Burundi or Urundi or Cambodia or Khmer Republic or Kampuchea or Cameroon or Cameroons or Cameron or Camerons or Cape Verde or Cabo Verde or Central African Republic or Chad or Tchad or China or Colombia or Comoros or Comoro Islands or Comores or Mayotte or Congo or Zaire or Costa Rica or Cote d'Ivoire or Ivory Coast or Cuba or Djibouti or French Somaliland or Dominica or Dominican Republic or East Timor or East Timur or Timor Leste or Ecuador or Egypt or United Arab Republic or El Salvador or Eritrea or Ethiopia or Fiji or Gabon or Gabonese Republic or Gambia or Gaza or Georgia Republic or Georgian Republic or Ghana or Grenada or Guatemala or Guinea or Guiana or Guyana or Haiti or Honduras or India or Maldives or Indonesia or Iran or Iraq or Jamaica or Jordan or Kazakhstan or Kazakh or Kenya or Kiribati or Korea or Kosovo or Kyrgyzstan or Kirghizia or Kyrgyz Republic or Kirghiz or Kirgizstan or Lao PDR or Laos or Lebanon or Lesotho or Basutoland or Liberia or Libya or Macedonia or Madagascar or Malagasy Republic or Malaysia or Malaya or Malay or Sabah or Sarawak or Malawi or Mali or Marshall Islands or Mauritania or Mauritius or Agalega Islands or Mexico or Micronesia or Middle East or Moldova or Moldovia or Moldovian or Mongolia or Montenegro or Morocco or Ifni or Mozambique or Myanmar or Myanma or Burma or Namibia or Nepal or Netherlands Antilles or Nicaragua or Niger or Nigeria or Muscat or Pakistan or Palau or Palestine or Panama or Paraguay or Peru or Philippines or Philipines or Phillipines or Phillippines or Papua New Guinea or Romania or Rumania or Roumania or Rwanda or Ruanda or Saint Lucia or St Lucia or Saint Vincent or St Vincent or Grenadines or Samoa or Samoan Islands or Navigator Island or Navigator Islands or Sao Tome or Senegal or Serbia or Montenegro or Seychelles or Sierra Leone or Sri Lanka or Solomon Islands or Somalia or Sudan or Suriname or Surinam or Swaziland or Eswatini or South Africa or Syria or Tajikistan or Tadzhikistan or Tadjikistan or Tadzhik or Tanzania or Thailand or Togo or Togolese Republic or Tonga or Tunisia or Turkey or Turkmenistan or Turkmen or Uganda or Ukraine or Uzbekistan or Uzbek or Vanuatu or New Hebrides or Venezuela or Vietnam or Viet Nam or West Bank or Yemen or Zambia or Zimbabwe or Rhodesia)

3. (Africa or Asia or Caribbean or West Indies or South America or Latin America or Central America)

4. ((developing or less* developed or least developed or under developed or underdeveloped or middle income or low* income or underserved or under served or deprived or poor* or resource limited or resource constrained) adj (countr* or nation? or population? or world or state* or emerging econom* or global south))

5. ((developing or less* developed or least developed or under developed or underdeveloped or middle income or low* income or resource limited or resource constrained) adj (economy or economies))

6. (low* adj (gdp or gnp or gross domestic or gross national))

7. (low adj3 middle adj3 countr*)

8. (lmic or lmics or third world or lami countr*)

9. transitional countr*

10. 1 OR 2 OR 3 OR 4 OR 5 OR 6 OR 7

*Immunisation*

1. (immuniz* or immunis* or vaccin* or inoculat* or innoculat* or immunotherap* or prophyla*)

*Young children and caregivers*

1. (child* or infant* or newborn* or neonat* or prenatal or pre natal or antenatal or ante natal or baby or babies or toddler* or preschool* or parent* or mother* or father* or maternal or paternal or caregiver* or grandparent* or grandmother* or grandfather* or family member*)

*Impact evaluation methods*

1. (random* or experiment* or (match* adj2 (propensity or coarsened or covariate)) or "propensity score" or "difference in difference*" or "difference-in-difference*" or "differences in difference*" or "differences-in-difference*" or "double difference*" or "quasi-experimental" or "quasi experimental" or "quasi-experiment" or "quasi experiment" or ((estimator or counterfactual) and evaluation*) or "instrumental variable*" or (IV adj2 (estimation or approach)) or regression discontinuity or time series or segment* regression)

- **Example full search strategy**

Below we present a draft of the full search strategy used to search MEDLINE. Note that in MEDLINE’s syntax, terms with strokes (e.g., Immunization/) denote Medical Subject Heading (MeSH) terms, while strings appended with “ti,ab,kw” are searched in the title, abstract, and keyword fields of records in the database.

**Ovid MEDLINE(R) and Epub Ahead of Print, In-Process & Other Non-Indexed Citations, Daily and Versions(R) <1946 to May 15, 2019> Searched 16^th^ May 2019**

1 (immuniz* or immunis* or vaccin* or inoculat* or innoculat* or immunotherap* or prophyla*).ti,ab,kw. (672732)

2 immunization/ or immunization, passive/ or immunization schedule/ or immunization, secondary/ or immunotherapy, active/ or vaccination/ or Immunization Programs/ or mass vaccination/ (158163)

3 Tuberculosis Vaccines/ or BCG Vaccine/ or Diphtheria-Tetanus Vaccine/ or Meningococcal Vaccines/ or Pertussis Vaccine/ or Diphtheria-Tetanus-acellular Pertussis Vaccines/ or Diphtheria-Tetanus-Pertussis Vaccine/ or Diphtheria-Tetanus Vaccine/ or Measles Vaccine/ or Mumps Vaccine/ or Rubella Vaccine/ or Measles-Mumps-Rubella Vaccine/ or Poliovirus Vaccines/ or Poliovirus Vaccine, Inactivated/ or Poliovirus Vaccine, Oral/ or Japanese Encephalitis Vaccines/ or Rotavirus Vaccine/ (49639)

4 or/1-3 (720583)

5 developing countries.sh,kf. (83271)

6 (Africa or Asia or Caribbean or West Indies or South America or Latin America or Central America).ti,ab,kw. (200981)

7 Africa/ or Asia/ or Caribbean/ or West Indies/ or South America/ or Latin America/ or Central America/ (73389)

8 (Africa or Central America or South America or Caribbean or Central Asia or Afghanistan or Albania or Algeria or Angola or Argentina or Armenia or Armenian or Azerbaijan or Bangladesh or Benin or Byelarus or Byelorussian or Belarus or Belorussian or Belorussia or Belize or Bhutan or Bolivia or Bosnia or Herzegovina or Hercegovina or Botswana or Brazil or Bulgaria or Burkina Faso or Burkina Fasso or Upper Volta or Burundi or Urundi or Cambodia or Khmer Republic or Kampuchea or Cameroon or Cameroons or Cameron or Camerons or Cape Verde or Cabo Verde or Central African Republic or Chad or Tchad or China or Colombia or Comoros or Comoro Islands or Comores or Mayotte or Congo or Zaire or Costa Rica or Cote d'Ivoire or Ivory Coast or Cuba or Djibouti or French Somaliland or Dominica or Dominican Republic or East Timor or East Timur or Timor Leste or Ecuador or Egypt or United Arab Republic or El Salvador or Eritrea or Ethiopia or Fiji or Gabon or Gabonese Republic or Gambia or Gaza or Georgia Republic or Georgian Republic or Ghana or Grenada or Guatemala or Guinea or Guiana or Guyana or Haiti or Honduras or India or Maldives or Indonesia or Iran or Iraq or Jamaica or Jordan or Kazakhstan or Kazakh or Kenya or Kiribati or Korea or Kosovo or Kyrgyzstan or Kirghizia or Kyrgyz Republic or Kirghiz or Kirgizstan or Lao PDR or Laos or Lebanon or Lesotho or Basutoland or Liberia or Libya or Macedonia or Madagascar or Malagasy Republic or Malaysia or Malaya or Malay or Sabah or Sarawak or Malawi or Mali or Marshall Islands or Mauritania or Mauritius or Agalega Islands or Mexico or Micronesia or Middle East or Moldova or Moldovia or Moldovian or Mongolia or Montenegro or Morocco or Ifni or Mozambique or Myanmar or Myanma or Burma or Namibia or Nepal or Netherlands Antilles or Nicaragua or Niger or Nigeria or Muscat or Pakistan or Palau or Palestine or Panama or Paraguay or Peru or Philippines or Philipines or Phillipines or Phillippines or Papua New Guinea or Romania or Rumania or Roumania or Rwanda or Ruanda or Saint Lucia or St Lucia or Saint Vincent or St Vincent or Grenadines or Samoa or Samoan Islands or Navigator Island or Navigator Islands or Sao Tome or Senegal or Serbia or Montenegro or Seychelles or Sierra Leone or Sri Lanka or Solomon Islands or Somalia or Sudan or Suriname or Surinam or Swaziland or Eswatini or South Africa or Syria or Tajikistan or Tadzhikistan or Tadjikistan or Tadzhik or Tanzania or Thailand or Togo or Togolese Republic or Tonga or Tunisia or Turkey or Turkmenistan or Turkmen or Uganda or Ukraine or Uzbekistan or Uzbek or Vanuatu or New Hebrides or Venezuela or Vietnam or Viet Nam or West Bank or Yemen or Zambia or Zimbabwe or Rhodesia).ti,ab,kw,sh. (1387951)

9 ((developing or less* developed or least developed or under developed or underdeveloped or middle income or low* income or underserved or under served or deprived or poor* or resource limited or resource constrained) adj (countr* or nation? or population? or world or state*)).ti,ab,kw. (91955)

10 ((developing or less* developed or least developed or under developed or underdeveloped or middle income or low* income or resource limited or resource constrained) adj (economy or economies)).ti,ab,kw. (504)

11 (low* adj (gdp or gnp or gross domestic or gross national)).ti,ab,kw. (234)

12 (low adj3 middle adj3 countr*).ti,ab,kw. (12819)

13 (lmic or lmics or third world or lami countr*).ti,ab,kw. (6486)

14 (transitional countr* or emerging econom* or global south).ti,ab,kw. (873)

15 or/5-14 (1487811)

16 4 and 15 (80257)

17 Parents/ or Fathers/ or Mothers/ or Grandparents/ or Caregivers/ or Single Parent/ or Pregnant Women/ or Child, Preschool/ or Infant/ or Infant, Newborn/ or Infant, Low Birth Weight/ or Infant, Small for Gestational Age/ or Infant, Very Low Birth Weight/ or Infant, Extremely Low Birth Weight/ or Infant, Postmature/ or Infant, Premature/ or Infant, Extremely Premature/ (1597959)

18 (child* or infant* or newborn* or neonat* or neo nat* or prenatal or pre natal or ante natal or antenatal or baby or babies or toddler* or preschool* or parent* or mother* or father* or maternal or paternal).ti,ab,kw. (2284298)

19 or/17-18 (2893768)

20 16 and 19 (28515)

21 (random* or experiment* or (match* adj2 (propensity or coarsened or covariate)) or "propensity score" or ("difference in difference*" or "difference-in-difference*" or "differences in difference*" or "differences-in-difference*" or "double difference*") or ("quasi-experimental" or "quasi experimental" or "quasi-experiment" or "quasi experiment") or ((estimator or counterfactual) and evaluation*) or "instrumental variable*" or (IV adj2 (estimation or approach)) or regression discontinuity or time series or segment* regression).ti,ab,kw. (2983909)

22 Randomized Controlled Trial/ or Random Allocation/ or Evaluation Studies/ or Propensity Score/ or Interrupted Time Series Analysis/ or Controlled Before-After Studies/ or Controlled Clinical Trial/ or Non-Randomized Controlled Trials as Topic/ (896249)

23 or/21-22 (3415351)

24 Cost Analysis/ or Cost-Benefit Analysis/ or Quality-Adjusted Life Years/ or Economics, Medical/ or Cost of Illness/ or Health Care Costs/ or Direct Service Costs/ or Budgets/ or Health Care Sector/ or Public Expenditures/ (193420)

25 (cost-effective* or cost-benefit).ti,ab,kw. (130711)

26 ("life year" or "life years" or qaly* or daly*).ti,ab,kw. (18749)

27 ((economic* or cost*) adj6 (mortality or death* or markov)).ti,ab,kw. (17246)

28 ("cost minimi*" or "cost-utilit*" or "economic evaluation*" or "economic review*" or "cost outcome" or "cost analys*" or "economic analys*" or "budget* impact analys*").ti,ab,kw. (27594)

29 or/24-28 (301485)

30 (review or meta-analysis).pt. (2557796)

31 meta-analysis/ or "systematic review"/ (163880)

32 cochrane database of systematic reviews.jn. (14162)

33 (systematic review or literature review).ti. (130870)

34 or/30-33 (2590211)

35 23 or 29 or 34 (5946083)

36 20 and 35 (8309)

37 exp Animals/ (22312530)

38 Humans/ (17732234)

39 37 not (37 and 38) (4580296)

40 36 not 39 (7984)

## Appendix D: Qualitative search protocol

**Purpose**

The purpose of this review is to inform policy recommendations related to community engagement interventions to improve immunisation outcomes in low- and middle-income countries. Specifically, we would like to provide insights related to (1) what to do / not do and (2) where to do it / not do it. We want to know not only what works, but why and how.

Our primary, qualitative research question is: What factors relating to programme design, implementation, context, and mechanism are associated with better or worse outcomes along the causal chain? Do these vary by the level of community engagement?

Sub questions are:

1. What pre-intervention activities and characteristics of local context facilitate or inhibit the effectiveness of an intervention?

a. What characteristics of local context facilitate or inhibit the effectiveness of an intervention? These include but are not limited to feasibility, clinic readiness, acceptability, need, barriers, and demographic composition.

b. What is the evidence regarding the likelihood that pre-intervention activities, including diagnostics, formative research, and community engagement, affect the effectiveness of an intervention?

2. What is the evidence supporting or refuting the existence of certain steps along the ToC?

a. Which steps in the causal chain tend to be barriers to or facilitators of impact? Where does the causal chain tend to be interrupted?

b. What time varying contextual factors affect the relationships seen in the ToC?

In order to respond to these questions, 3ie seeks to identify qualitative papers related to the quantitative papers included in the broader systematic review on effectiveness of community engagement interventions for improving immunisation outcomes. This information will contextualise the results of the quantitative papers. The qualitative papers will be instrumental in expanding our understanding of why and how certain interventions were successful (or not).

This document outlines the suggested approach for identifying these qualitative papers.

**Required steps**

1. Create a unique dropbox folder for each study using the first author’s last name and year as the folder title

2. For each step outlined below, save all potentially relevant documents to this file as they are identified.

a. Consider this stage to be equivalent to title and abstract screening. If you identify an article that can be quickly discarded as irrelevant (such as budgetary information), there is no need to include. However, if you think that there may be any information related to our research questions, please include at this stage.

b. If relevant websites are identified that cannot be easily converted to PDF format, create a word document. Write the title of the webpage and provide the link below it.

**If a trial registration number for clinicaltrials.gov is provided**

Use this approach if a trial registration number for clinicaltrials.gov is provided. This will be referenced in the abstract and / methods section and appear in a form similar to that found in Robertson et al 2013:

This trial is registered with ClinicalTrials.gov, number NCT00966849.

If no trial registration number is indicated, proceed to the following section (Section 2: If the project has a unique name).

1.1 Go to the website [clinialTrials.gov](https://clinicaltrials.gov/)

1.2 Under “find a study” in the field “other terms,” enter the trial registration number.

1.3 There should only be one hit. Click on the title under “study title”

1.4 Three tabs should be available under the basic study information. They read “study details,” “tabular view,” and “study results.” Select “study results”

1.5 Scroll to the bottom of the page. Under “more information” there are sections titled “Publication of results” and “other publications”

a. In most (but not all) cases, there should be a list of publications here. Save the relevant files to the folder

After this, proceed to section 2.

**If the project has an unique name**

Use this approach if the intervention is named. If a project name is not given in the paper, but the project is registered on clinicaltrials.gov, use the project title provided there.

2.1 Search the project name on google scholar.

a. Review each search result until you find 5 in a row that are irrelevant.

b. At this point, stop and proceed to step 2.2.

2.2 If the funder’s name is not provided, proceed directly to step 2.3. If the funder’s name is provided, search the project name on the funder’s website.

a. Review each search result until you find 5 in a row that are irrelevant.

b. At this point, stop and proceed to step 2.3.

2.3 If the implementer’s name is not provided, proceed directly to section 3. If the implementer’s name is provided, search the project name on the implementer's website.

a. Review each search result until you find 5 in a row that are irrelevant.

**Other options**

If the trial is not registered and does not have a formal name, the following approaches can be used as a last resort:

3.1 If funder and / or implementer names are provided, go to their websites. Search for the article on their website. It may link to a program page.

a. If no program page is identified, try to manually search through their website.

3.2 Search the full article name on google and google scholar. Review the top 10 hits on each to see if they provide more information about the program.

If during either of these steps, a program name and / or trial registration number are identified, return to the relevant steps above.

**Searching funder and implementer websites**

Some of these websites will have good, built-in search functions. If these are useful, use them. However, if the provided search functionality is not adequate, type site:HomepageOfFunderWebsite SearchTerm. For example, if I wanted to search the World Bank website for information about COVID, I would search “site:www.worldbank.org COVID” in google (Note: no space between “site:” and the url). This will provide me hits from the World Bank website related to COVID.

**If no linked articles are identified**

At the end of each day, email Avantika (abagai@3ieimpact.org) and Charlotte (clane@3ieimpact.org) the title of all papers for which you were unable to identify linked articles.

## Appendix E1: Quantitative data extraction tool

| 1. VARIABLE LABEL | **EXPLANATION** |
| --- | --- |
| Study ID | This is the study ID - it should match the study ID from the Outcome Mapping Sheet (e.g., SC-SR_1) |
| Estimate ID | The estimate ID will provide a specific number for each effect size extracted and should include the original study number, underscore, then the unique ID number (e.g., SC-SR1_1, SC-SR1_2 and so on) |
| Author | For 1 author: leading author last name (e.g., Gomez)  For 2 authors: both author last names with ampersand in between (e.g., Smith & Bahn)  For 3 or more authors: leading author last name followed by et al. (e.g., Gupta et al.) |
| Year | Year published |
| Design | 0=Experimental Design (e.g., RCT), 1=Quasi-Experimental Design |
| How Counterfactual is Chosen? | Free text (e.g., random control trial, propensity score matching, etc) - Multiple codes are ok |
| Analysis type for this effect size | Free text, what type of analysis was used (Regression, 2SLS, ANCOVA, etc.)- Multiple codes are ok |
| Country | Country of intervention |
| Region | Region/continent of intervention |
| Estimate Type | Type of data for this effect size: 1 = Continuous - means and SDs, 2 = Continuous - mean difference and SD, 3 = Dichotomous outcome - proportions, 4 = Regression data - dichotomous outcome (e.g., logistic regression)  5 = Regression data - continuous outcome (e.g., linear regression) |
| Comparison | 1=No intervention (service delivery as usual), 2=Other intervention, 3=Pipeline (wait-list) control (still service delivery as usual) |
| Describe Comparison Group | If answer above is (1) no intervention, type N/A, if (2) Other Intervention, list what intervention the control group is receiving, if (3) Pipeline control, report when the control group will receive the intervention in relation to the treatment group (e.g., one year later) |
| Subgroup | Is this analysis of a subgroup? 0=no, 1=yes |
| If yes to subgroup, describe | Free text, describe the subgoup if applicable (e.g., boys, girls). If no subgroup, type N/A |
| Source | Note the page number, table number, column, and row you used to extract the data |
| Treatment Effect | 1=Intention to Treat (ITT), 2=Average Treatment Effect on the Treated (ATET), 3=Average Treatment Effect (ATE) 4 = Local Average Treatment Effect (LATE) |
| Intervention | Free text, what is the intervention |
| Engagement as intervention | 1=yes, 0=No |
| Engagement as intervention: developing community buy-in | 1=yes, 0=No |
| Engagement as intervention: creation of new cadres or health committees | 1=yes, 0=No |
| Engagement as intervention: both | 1=yes, 0=No |
| Engagement in design | 1=yes, 0=No |
| Engagement in design: community decision makinh | 1=yes, 0=No |
| Engagement in design: community feedback | 1=yes, 0=No |
| Engagement in implementation | 1=yes, 0=No |
| Engagement in implementation: governance and decisions | 1=yes, 0=No |
| Engagement in implementation: provision of resources | 1=yes, 0=No |
| Multiple engagement types | 1=yes, 0=No |
| Engagement in design and engagement as intervention | 1=yes, 0=No |
| Engagement in design and implementation | 1=yes, 0=No |
| Engagement in implementation and engagement as intervention | 1=yes, 0=No |
| Exposure to intervention (in months) | How long is the intervention exposure itself? |
| Evaluation period (in months) | The total number of months elapsed between offering an intervention and the point at which an outcome measure is taken post intervention, or as a follow-up measurement. If less than one month, use decimals (e.g., one week would be .25) |
| Post-intervention or change from baseline? | 0 = Post-intervention, 1 = Change from baseline |
| Source of the Outcome data (only use for outcomes in category JAA) | 1=Immuniation Card, 2=Recall, 3=Combination of both immunisation card and recall, 4=Health Admin Data, 5=N/A |
| Author definition of outcome | Free text - How does the author define the outcome? |
| **OUTCOME CODES** | |
| **Thinking and Feeling** | Code 1 under any applicable columns. See OUTCOME GUIDANCE document for further explanations. |
| **Social Processes** | Code 1 under any applicable columns. See OUTCOME GUIDANCE document for further explanations. |
| **Readiness to Vaccinate** | Code 1 under any applicable columns. See OUTCOME GUIDANCE document for further explanations. |
| **Practical Factors** | Code 1 under any applicable columns. See OUTCOME GUIDANCE document for further explanations. |
| **Community Health Workers** | Code 1 under any applicable columns. See OUTCOME GUIDANCE document for further explanations. |
| **Vaccinators** | Code 1 under any applicable columns. See OUTCOME GUIDANCE document for further explanations. |
| **IAC Admins** | Code 1 under any applicable columns. See OUTCOME GUIDANCE document for further explanations. |
| **Health information systems** | Code 1 under any applicable columns. See OUTCOME GUIDANCE document for further explanations. |
| **Vaccine Availability** | Code 1 under any applicable columns. See OUTCOME GUIDANCE document for further explanations. |
| **Resources** | Code 1 under any applicable columns. See OUTCOME GUIDANCE document for further explanations. |
| **Vaccination coverage** | Code 1 under any applicable columns. See OUTCOME GUIDANCE document for further explanations. |
| **Health Outcomes** | Code 1 under any applicable columns. See OUTCOME GUIDANCE document for further explanations. |
| **EFFECT SIZE DATA EXTRACTION** | |
| Reverse Sign (i.e., decrease is good) | Record 0='no' if an increase is good, record 1='yes' if a decrease is good and the sign needs to be reversed. |
| Unit of analysis | What is the unit of analysis? UOA for this effect size: 1= Individual, 2= Household, 3= Group (e.g. community organisation), 4= Health Center, 5 = Village, 6 = Other, 7 = Not clear |
| mean_t | Outcome mean for the treatment group |
| sd_t | Outcome standard deviation for treatment group |
| mean_c | Outcome mean for the comparison group |
| sd_c | Outcome standard deviation for control group |
| mean_overall_diff | Overall mean difference (treatment - control) |
| diff se | Standard error of the overall mean difference |
| Diff _t | t-statistic of mean difference |
| Odds ratio | Odds ratio reported in the study |
| OR_se | Odds ratio standard error reported in the study |
| Risk ratio | Risk ratio reported in study |
| RR_se | Risk ratio standard error |
| reg_coeff | Report the regression coefficient of the treatment effect |
| reg_SE | Report the associated standard error of the regression coefficient. |
| reg_t | Report the associated t statistic of the effect size (coefficient/SE) |
| Exact p value | Exact p value if given, if not, record as written in the manuscript (e.g., p < .001, or p > .05) |
| clust_t | Number of clusters - treatment group |
| clust_c | Number of clusters - control group |
| clust_T | Number of clusters - total sample |
| n_t | Sample size - treatment group |
| n_c | Sample size - control group |
| n_T | Sample size - total sample |
| Does the sample size need to be adjusted (if so complete column CL)? | Code as ‘yes’ or no’ |
| periods (1 if cross sectional) | Record how many periods of evaluation there are (e.g., cross section is 1, panel data with 3 measurements is 3) |
| Treatment Variable | Record the treatment variable as written in the model (e.g., the variable name the author uses, such as ("Intervention x Time") |
| dataset | Record if data comes from an identified dataset |
| coder | Record your name |
| Notes | Record any notes important for the team |
| n_T_revised | THIS IS FOR SENIOR QUANT LEAD TO FILL OUT |
| sp | THIS IS FOR SENIOR QUANT LEAD TO FILL OUT |
| d | THIS IS FOR SENIOR QUANT LEAD TO FILL OUT |
| g | THIS IS FOR SENIOR QUANT LEAD TO FILL OUT |
| var(d) | THIS IS FOR SENIOR QUANT LEAD TO FILL OUT |
| se(d) | THIS IS FOR SENIOR QUANT LEAD TO FILL OUT |
| CI_l | THIS IS FOR SENIOR QUANT LEAD TO FILL OUT |
| CI_u | THIS IS FOR SENIOR QUANT LEAD TO FILL OUT |
| remove | THIS IS FOR PROJECT MANAGER TO FILL OUT |
| Formula Used | THIS IS FOR SENIOR QUANT LEAD TO FILL OUT |
| yi_1 | THIS IS FOR SENIOR QUANT LEAD TO FILL OUT |
| yi_rev | THIS IS FOR SENIOR QUANT LEAD TO FILL OUT |
| yi | THIS IS FOR SENIOR QUANT LEAD TO FILL OUT |
| vi | THIS IS FOR SENIOR QUANT LEAD TO FILL OUT |
| wi | THIS IS FOR SENIOR QUANT LEAD TO FILL OUT |
| ywi | THIS IS FOR SENIOR QUANT LEAD TO FILL OUT |
| 95ci_lower | THIS IS FOR SENIOR QUANT LEAD TO FILL OUT |
| 95ci_upper | THIS IS FOR SENIOR QUANT LEAD TO FILL OUT |
| cilow_3sf | THIS IS FOR SENIOR QUANT LEAD TO FILL OUT |
| cihigh_3sf | THIS IS FOR SENIOR QUANT LEAD TO FILL OUT |
| ci | THIS IS FOR SENIOR QUANT LEAD TO FILL OUT |
| wb_yi | THIS IS FOR SENIOR QUANT LEAD TO FILL OUT |
| Checked | THIS IS FOR EFFECT SIZE RELIABILITY CHECKER TO FILL OUT |
| ROB Category | THIS IS FOR SENIOR QUANT LEAD OR PM TO FILL OUT |

## Appendix E2: Cost effectiveness data extraction tool

| **Study Characteristics** | **Variable Label** | **Explanation** | **Source** |
| --- | --- | --- | --- |
| **Coder** |  |  |  |
| Study ID | Study ID | This is the study ID - it should match the study ID from the Outcome Mapping Sheet (e.g., SC-SR_1) | Data extraction tool_immunisation |
|  | Study ID Component |  |  |
| Year | Year | Year published | Data extraction tool_immunisation |
| Design | Design | 0=Experimental Design (e.g., RCT), 1=Quasi-Experimental Design | Data extraction tool_immunisation |
| Country | Country | Country of intervention | Data extraction tool_immunisation |
| Study Link |  |  |  |
| Which Vaccines were studied? |  |  |  |
| How many vaccine doses were delivered? |  |  |  |
| Source of info about vaccines |  |  |  |
| Outcomes | [Source from CEA Inventory] |  |  |
| Target population |  |  |  |
| Implementing partners |  | What organization(s) carried out the intervention? [Please list names of organization(s) that implemented the program, e.g. IRC, Government of Uganda. Etc)] | Studies |
| Analytical perspective |  | Was the perspective of the costing stated explicitly? [Yes, No]. Analytical perspective is the choice of who has standing in the costing and determines whose costs and benefits will be counted. Perspective should be stated explicitly in the costing, so here it is ok to do a kw search for "perspective" to check | Studies |
| Analytical perspective |  | What is the analytical perspective of the costing (i.e. donor, financial cost, economic or social perspective). | Studies |
| Treatment arms |  | How many treatment arms were included in the study [# of treatment arms] |  |
| Comparison | Comparison | 1=No intervention (service delivery as usual), 2=Other intervention, 3=Pipeline (wait-list) control (still service delivery as usual) | Data extraction tool_immunisation |
| Describe Comparison Group | Describe Comparison Group | If answer above is (1) no intervention, type N/A, if (2) Other Intervention, list what intervention the control group is receiving, if (3) Pipeline control, report when the control group will receive the intervention in relation to the treatment group (e.g., one year later) | Data extraction tool_immunisation |
| Subgroup | Subgroup | Is this analysis of a subgroup? 0=no, 1=yes | Data extraction tool_immunisation |
| If yes to subgroup, describe | If yes to subgroup, describe | Free text, describe the subgoup if applicable (e.g., boys, girls). If no subgroup, type N/A | Data extraction tool_immunisation |
| Source | Source | Note the page number, table number, column, and row you used to extract the data | Data extraction tool_immunisation |
| Treatment Effect | Treatment Effect | 1=Intention to Treat (ITT), 2=Average Treatment Effect on the Treated (ATET), 3=Average Treatment Effect (ATE) 4 = Local Average Treatment Effect (LATE) | Data extraction tool_immunisation |
| Summary of intervention | Summary of intervention | Free text, what is the intervention | Intervention_community engagement_dataset [col E] |
| Component # | Component # | List in numbers 1-6 | Intervention_community engagement_dataset [col H] |
| Description | Description | Description [Col I in Intervention_community engagement_dataset] | Intervention_community engagement_dataset [col I] |
| Notes | Notes | Description [Col S in Intervention_community engagement_dataset] | Intervention_community engagement_dataset [col S] |
| Exposure to intervention (in months) | Exposure to intervention (in months) | How long is the intervention exposure itself? | Data extraction tool_immunisation |
| Evaluation period (in months) | Evaluation period (in months) | The total number of months elapsed between offering an intervention and the point at which an outcome measure is taken post intervention, or as a follow-up measurement. If less than one month, use decimals (e.g., one week would be .25) | Data extraction tool_immunisation |
| Post-intervention or change from baseline? | Post-intervention or change from baseline? | 0 = Post-intervention, 1 = Change from baseline | Data extraction tool_immunisation |
| Source of the Outcome data (only use for outcomes in category JAA) | Source of the Outcome data (only use for outcomes in category JAA) | 1=Immunisation Card, 2=Recall, 3=Combination of both immunisation card and recall, 4=Health Admin Data, 5=N/A | Data extraction tool_immunisation |
| Author definition of outcome | Author definition of outcome | Free text - How does the author define the outcome? | Data extraction tool_immunisation |
| Activities |  | What are the main (non-evaluation) activities undertaken by the project? (e.g. program design, targeting, community outreach, training of trainers, training of beneficiaries, M&E, etc.). Describe | Studies |
| Activities Data Source |  | Was the source of descriptive information about program activities taken from a table or taken from text? [Text, Table, Other(describe)] | Studies |
| Timeline |  | Is an intervention timeline included with the report? [Yes, No, If yes, page #] | Studies |
| Ingredients (unit cost of ingredients) |  | Is a list of cost ingredients or inputs for CEA, CBA, or CUA provided? [Yes, No] Ingredients will be items like "personnel", "staff", "travel", "office support", "program materials", etc. Note that searches for the unit costs of activities should include text searches as well as searches of tables. | Studies |
| Are unit costs reported? |  | Look for a table with units or unit costs reported. Unit costs give the value of individual items that were used during the intervention, for example the unit cost of a staff person, e.g. a "health specialist" would be given in terms of monthly wages, or compensation or salary. Note that searches for the unit costs of activities should include text searches as well as searches of tables. [Yes, No, If Yes Page #] | Studies |
| Cost data sources |  | Was a source of cost data reported? (i.e. financial reports or accounting systems of implementing NGOs or donors (expenditures), NGO or donor (budgets), (market prices) Describe (or copy from text). Clarify - expenditure is what's actually spent, budget is what's planned for spending / not audited, often incorrect and not updated. | Studies |
| Costing methodology |  | Was a method of costing described? (i.e. key works: gross costing, micro-costing, ingredients method, activity-based costing method) [if yes, what was the method]. |  |
| Type of efficiency analysis (see glossary) |  | Indicate (CBA/CEA/CUA/cost-minimization, or innovative approaches: Social Return on Investment (SROI), Multi-Criteria Appraisal (MCA). Response options [CBA, CEA, CUA, SROI, MCA, or N/A if only "total cost", also ok to insert descriptive results that do not conform, e.g. “cost transfer ratio”] | Studies |
| Any cost-effectiveness analysis? | Source [from CEA Inventory] |  |  |
| Any quant discussion of costs? (Full program) | Source [from CEA Inventory] |  |  |
| If yes, what type? [Page number] | Source [from CEA Inventory] |  |  |
| Currency of cost reporting |  | What is the currency in which costs were reported, i.e. USD, Rwandan Francs, etc. | Studies |
| Exchange rate |  | What exchange rate was used for currency adjustments? [e.g. 1.2 USD to 1 Euro] |  |
| Exch rate year (estimated) |  |  |  |
| Exchange rate date |  | What was the date, month, year of the exchange rate used? [If the exchange rate was calculated as an average over the implementation period, copy verbatim from text the definition of average exchange rate used] |  |
| Was there discussion of a discount rate? |  | [Yes, No] | Studies |
| Discount rate |  | Which is the discount rate used in the analysis [this will be a % , please give the value] | Studies |
| If no discount rate is applied, was any justification provided? |  | Paste explanation provided in text. We consider any acknowledgement of the discount rate to be justification. For example, ok to report" "we did not discount costs or benefits" as justification | Studies |
| Inflation adjustment |  | Does the report mention if costs are given in "real" or "nominal" terms? Look also for "inflation" | Studies |
| Inflation adjustment of cost reported |  | If costs are reported in real terms, what inflation index was used to adjust for inflation? | Studies |
| Base year of the costing | base_year | Was a "base year" (or "start year") of the costing reported? [Yes, No] [If yes, What year?] | Studies |
| Average cost per number of participants |  | Was the average cost of the intervention reported per participant? [Yes, No] [If Yes, $ Amount per participant, and currency] | Studies |
| What is the denominator used in average intervention cost? | ac_ denominator | [Number, Description (please copy description verbatim from report)] | Studies |
| Average cost per DALY or QALY? |  | Is the average cost per DALY or QALY reported? [Yes, No, If Yes Page #], Also, add note if cost ‘per capita’ was given rather than average cost per number of participants. | Studies |
| Total intervention cost (excluding vaccine cost) | total_cost | Report total cost of the intervention, and page. | Studies |
| Did the total intervention cost reported above include the cost of vaccines? | vac_incl | A "No" indicates that cost ingredients were reported, but there is no reporting of vaccine costs. "Unclear" means we cannot tell from the information given if vaccines were included in total cost [Yes, No, Unclear] | Studies |
| Total cost description | total_cost_ desc | If there is a description of what is included in total cost, copy description from text here | Studies |
| Government contribution included in total cost? | tc_gov_ contb | [Yes, No] |  |
| Is more than one total cost given in the report? |  | [Yes, No, If Yes, please describe additional "total cost" that is reported |  |
| Number of immunisations |  |  |  |
| Cost per vaccine delivered, cost per additional immunisation administered or cost per dose (of vaccine). Type of vaccine is given in col I |  |  |  |
| Description and page number of cost per additional vaccine delivered. |  | Note if average costs per additional vaccine or marginal costs per additional vaccine is reported, where marginal costs should exclude fixed costs. |  |
| Cost per additional child immunised_ All | cpc_all | [Yes, No, If Yes, please report cost per additional child immunised_all] |  |
| Description and page number of cost per additional CHILD Immunised |  |  |  |
| Cost per additional child immunised_DPT3 | cpc_dpt3 | [Yes, No, If Yes, please report cost per additional child immunised with DPT3]. Please make a note if only DPT1 was reported. |  |
| Cost per additional child immunised_Measles | cpc_measles | [Yes, No, If Yes, please report cost per additional child immunised with measles] |  |
| Baseline immunisation coverage of the target population_All |  | What was the reported baseline rate of immunisation (all immunisations) in the target population? [Percentage, or range] | Studies |
| Was baseline immunisation coverage of All immunisations reported for the target population, general population, or sample? |  | Was baseline immunisation coverage of All immunisations reported for the target population, general population, or sample? [target population, general population, sample] | Studies |
| Baseline immunisation coverage of the target population_DPT3 |  | What was the reported baseline rate of immunisation (DPT3) in the target population? [Percentage, or range] | Studies |
| Was baseline immunisation coverage of DPT3 reported for the target population, general population, or sample? |  | Was baseline immunisation coverage of DPT3 reported for the target population, general population, or sample? [target population, general population, sample] | Studies |
| Baseline immunisation coverage of the target population_Measles |  | What was the reported baseline rate of immunisation (Measles) in the target population? [Percentage, or range] | Studies |
| Final immunisation coverage of the target population_All |  | What was the reported final rate of immunisation (all immunisations) in the target population? [Percentage, or range] | Studies |
| Final immunisation coverage of the target population_DPT3 |  | What was the reported final rate of immunisation (DPT3) in the target population? [Percentage, or range] | Studies |
| Final immunisation coverage of the target population_Measles |  | What was the reported final rate of immunisation (Measles) in the target population? [Percentage, or range] | Studies |
| Non-compliance |  | Was there any evidence of non-compliance with treatment assignment. Non-compliance is where individuals assigned to treatment do not take the treatment, or the case where individuals assigned to control DO take the treatment. Ok to keyword search on "non-compliance", "non compliance", "noncompliance" [Yes, No] [If Yes, copy related text on non-compliance] | Studies |
| Spillover |  | Was there any evidence of spillover where individuals assigned to control receive a benefit or incur a 'cost' that results from the treatment intervention? [Yes, No] [If Yes, copy related text on spillovers detected] | Studies |
| Attrition or drop out |  | Was there evidence that participants dropped out of the study before follow-up could be completed? [Yes, No] [If Yes, copy related text on drop outs] | Studies |
| Vaccination coverage_All | JAA01. Full routine immunisation for children | Binary measure of whether or not children have received all routine vaccinations for the relevant country or region. | Data extraction tool_immunisation |
| Vaccination coverage_DPT3 | JAA05. DPT3 | Binary measure of whether or not children have received the third dose of the DPT or pentavalent vaccine. If the study does not specifically say “DPT3” (or “pentavalent 3”), but refers to “complete DPT/penta vaccination” or something similar, then use this code. | Data extraction tool_immunisation |
| Vaccination coverage_Measles | JAA11. Measles | Binary measure of whether or not children have received the measles vaccine | Data extraction tool_immunisation |
| Costs for significant outcomes only? |  | Does the report mention that costs will be reported only where significant outcomes are observed? [Yes, No] |  |

## Appendix E3: Qualitative coding tool

| Name | Description |
| --- | --- |
| Causal mechanism | This set of codes describes the causal mechanisms observed or hypothesized |
| Barriers to immunisation | Select any state reasons that people were not immunised. Try to use previously added codes to reduce possible redundancies, but add codes if needed. |
| Demand side barriers | Barriers related to low demand for immunisations |
| Fear | Low demand for immunisation due to fear of side effects or other factors |
| Financial | Select the financial reason that people were not immunised Add sub-codes as needed. |
| Cost of immunisation | People were not immunised due to the cost of the immunisation Do not add sub-codes |
| Cost of transport | People were not immunised due to the cost of transport. |
| Opportunity costs | People were not immunised due to opportunity costs |
| Knowledge of Immunisation Schedule | Low demand for immunisation due to limited knowledge of its importance. This includes instances when the person simply does not feel that immunisations are important (regardless of "book knowledge") |
| Logistics or distance | Barriers related to logistics or distance. For example, it is too far away, I need to arrange for XXX to happen while I am gone... |
| Self-efficacy | Issues related to not feeling empowered to get vaccinated. This includes not knowing how to go about getting vaccinations. For example, not knowing when or where to get vaccinated |
| Social norms | Low demand for immunisation due to social pressures and social norms |
| Understanding of importance | Low demand for immunisation due to limited knowledge of its importance. This includes instances when the person simply does not feel that immunisations are important (regardless of "book knowledge") |
| Supply side barriers | Limitations to supply that cause problems for immunisation |
| Availability of services | Services may not be physically present. This includes clinics being closed. |
| Human resources | Limited human resources may reduce the availability of immunisations. Ex: Appropriate staff were not present to provide the injection |
| Lack of Infrastructure or supplies | Use code if the lack of immunisation was because there were no drugs, vaccines, cold chain or other items related to physical infrastructure of the health facility |
| Poor quality services | Immunisations are not common because there is low quality of service. Providers may not be cooperative, slow, unprofessional/rude, unempathetic, abusive, or inconsistent. |
| Lack of accountability | Use code for text referring to issues related to accountability, corruption, governance and transparency in providing services. |
| Lack of Motivation | Use if for text indicating that the health workers are not motivated to perform their duties due to any reason like lack of monetary incentives, recognition, low job satisfaction and other issues. |
| Baseline conditions and descriptive characteristics | Discussions of the baseline conditions and descriptive characteristics of the region or population in which the intervention took place. This does not necessarily come from a baseline survey, but can be through other sources. Do not add sub-codes |
| Health systems context | Discussion of the health system functioning and context Example: The clinic served a population of 1,000 people Do not add sub-codes |
| Immunisation rates | Discussion of baseline immunisation rates. Example: Immunisation rates were low at baseline. Do not add sub-codes |
| Political context | Discussion of the political context Example: Political unrest made the intervention difficult to implement. Do not add sub-codes |
| Socioeconomic status and demographics | Discussions of SES and demographics at baseline. Example: The community was mostly farmers. Population size Do not add sub-codes |
| Facilitators of immunisation | Select any state reasons that people were immunised. Try to use previously added codes to reduce possible redundancies, but add codes if needed. |
| Demand Side Facilitators |  |
| Favourable population SES characteristics | For instance those from a higher wealth quantile, urban areas or mothers with higher educational qualifications are more likely to get their children immunised. Do not add sub-codes. |
| Knowledge of immunisation schedule | Use this code if caregivers are aware of all immunisations in the schedule and know which vaccination is due when. Do not add sub-codes. |
| Lack of fear | Text indicating that there was no fear or vaccine hesitancy. Do not add sub-codes |
| Lack of financial constraints | Caregivers can afford the cost of immunisation, cost of transportation to visit healthcare facilities, taking children for immunisation does not come at a high opportunity cost for the caregiver. Do not add sub-codes. |
| Lack of logistics or distance related challenges | For instance, caregivers were able to access health services because they lived within a 5km radius of a healthcare facility or they received outreach services regularly. Do not add sub-codes. |
| Self-efficacy | Caregivers feel empowered to get their children vaccinated. For example, they know when and where to get vaccinated. Do not add sub-codes. |
| Social norms | The social environment owing to prevailing social pressures and norms is encouraging towards child immunisation. For instance, the mother in law in the household or the traditional/religious leaders in the community support immunisation of children. Do not add sub-codes. |
| Understanding of importance | Caregivers are aware about the importance of immunisation and are willing to get their children vaccinated. For example, caregivers are able to correctly identify vaccine preventable illness and therefore, more likely to get their children immunised. Do not add sub-codes. |
| Supply-side facilitators |  |
| Availability of infrastructure or supplies |  |
| Good quality of services |  |
| High accountability |  |
| High motivation |  |
| Human resources |  |
| Availability of services |  |
| Impacts | What impacts were ultimately achieved? Use this code for general impacts evaluated through the IE that do not fall into one of the other groups. Do not add sub-codes |
| Descriptive impacts | Include authors’ descriptions of impacts that were not directly measured through the IE. Ex: There was generally an increase over time in most sites |
| Equity considerations | If impacts on marginalized groups are considered, select the type of marginalized group considered. Do not add sub-codes |
| Ethnic minority | Impacts on ethnic minorities Do not add sub-codes |
| Other | Impacts on other marginalized groups, like scheduled castes in India. Do not add sub-codes |
| Religious minority | Impacts on religious minorities Do not add sub-codes |
| Women | Impacts on women Do not add sub-codes |
| Long term impacts | Impact measured over a timeframe of more than 5 years from the end of the intervention Do not add additional sub-codes |
| Unintended impacts | Only use this sub-code if the impact is stated as unintended. Do not add sub-codes |
| Reasons for project success or failure | Select text in which authors discuss the reasons for the success or failure of the project. This could be related to intervention design, implementation, or the research approach. Example: The intervention was not successful due to a lack of political will. Do not add sub-codes |
| Failure |  |
| Implementation or scale-up challenges |  |
| Competing priorities of health workers |  |
| Other delays, disruptions or implementation variability |  |
| Payment delays |  |
| Intervention |  |
| Design - Engagement |  |
| Design - other |  |
| Duration, frequency and exposure or reach |  |
| Not accounting for existing constraints or uncontrollable contextual trends |  |
| Caregiver competing priorities |  |
| Civil unrest or political instability or natural calamity |  |
| Fear of AEFI |  |
| Health system issues |  |
| Health service access or availability |  |
| Poor quality of service or infrastructure | Also includes issues related to health worker demotivation, recruitment or retention. |
| High baseline coverage | The interventions are not geared to/designed to achieve last mile coverage or breakthrough stagnating rates. |
| Lack of awareness or understanding |  |
| Migration |  |
| Resource constraints or scarcity | Infrastructure, electricity, cellular network, etc. |
| Social norms |  |
| Wider socio-economic or health-related progress |  |
| Study design |  |
| Contamination |  |
| Other methodological shortcomings |  |
| Mixed results |  |
| Success |  |
| Existing or changing favourable factors or contextual trends |  |
| Health system enablers |  |
| Good quality services and infrastructure | Includes enablers like good outreach, service monitoring, etc. |
| Health service access and availability |  |
| Self efficacy, social norms and awareness |  |
| Socio-economic factors |  |
| Implementation improvements |  |
| Intervention features |  |
| Community engagement |  |
| Behaviour change communication |  |
| Community dialogues |  |
| Community involvement in planning and implementation |  |
| Incentives |  |
| Needs assessments, pilots or stakeholder consultations |  |
| Other types of engagement |  |
| Customisation to local context |  |
| Health system integration and organisational structure |  |
| Health worker training |  |
| Intervention duration, dose and exposure |  |
| Leadership and supportive supervision |  |
| Other design features |  |
| Positive participant or beneficiary views of intervention |  |
| Theory of change | Information related to the ToC. Do not code here, but use sub-codes. Do not add sub-codes. |
| Assumptions | when discussing a causal chain mechanism, we often make assumptions about what will happen. These assumptions are often implicit. We may print materials with the assumption that people will be able to read them. We may train people with the assumption they speak our language. When these assumptions are stated, use the sub-codes to indicate if these assumptions were validated or not. Do not add sub-codes |
| Incorrect assumption | This indicates that there was an assumption in the causal chain mechanism and the assumption was not correct. For example, a study assumed that increasing supply would increase immunisations. Here, we are assuming that people want immunisations and that low supply is the problem. If instead, we find that people did not want the immunisations to begin with, the assumption was incorrect and would be indicated here. Do not add sub-codes |
| Valid assumption | this indicates that there was an assumption in the causal chain mechanism and the assumption was valid. For example, a study assumed that increasing supply would increase immunisations. Here, we are assuming that people want immunisations and that low supply is the problem. If this proves to be true, the text should be selected here. Do not add sub-codes |
| Causal chain mechanisms | Descriptions of how change is expected to occur. These causal chains are often represented as flow charts, or at least they can be. Text that reflects a causal chain mechanism will discuss expected actions and reactions. “We did this and we expected that.” Do not add sub-codes |
| Conclusions | Authors conclusions. Any summary of the take-home message of the article. Tends to be "this worked," "this did not work," "in conclusion." Do not add sub-codes |
| Cost | Discussions of costs, including the costs of the intervention and any form of cost-analysis. Only select key "take-home message." Do not add sub-codes |
| Data source | For anything related to causal mechanisms, uptake, and uptake and fidelity challenges, indicate what data source provided this information. Do not add additional sub-codes |
| Author notes or experiences |  |
| FGDs |  |
| Implementer interpretations or experiences |  |
| Individual interviews |  |
| Literature Review | Use this code if the text alludes to existing evidence on the subject |
| Observations | If the data comes from direct observations. This must be enumerators looking at and noting the state of something. If it is simply "general impressions," use the notes/interpretations or experience codes. Do not add sub-codes |
| Intervention description | This set of codes describes the intervention |
| Beneficiary selection | Select relevant information regarding how beneficiaries were selected for the intervention. Note: This is how beneficiaries of the intervention were selected, NOT a research sampling design or how research participants were selected. Do not add sub-codes |
| Community involvement | Description of how the community was involved in beneficiary selection. Example: A community council decided who was eligible. Do not add sub-codes |
| Equity considerations | Descriptions of efforts to ensure beneficiary selection was fair and equitable. Example: There was a quota to ensure adequate representation for ethnic minorities Do not add sub-groups |
| Other selection | Other descriptions of the selection process and eligibility criteria Do not add sub-codes |
| Researcher involvement | Description of the researcher involvement in selection, including selection criteria designed for academic analysis Example: If only those who were involved in and RCT received the intervention, then the sampling frame for the RCT would also be how beneficiaries for the intervention were selected. Do not add sub-codes |
| Community engagement | This set of codes describes community engagement in the interventions |
| Community mobilization | Select the relevant reference to community mobilization, regardless of how this phrase is used Do not add sub-codes |
| Absent | Statements that there was no effort at community mobilisation Example: A challenge was that community members were not aware of the intervention activities. Do not add sub-codes |
| Definitions of community mobilization | The author's definition for community mobilisation Do not add sub-codes |
| Present - with trust building | Descriptions of community engagement that was done in such a way as to establish trust and buy-in. Example: A local organisation was recruited to inform community members of the intervention and explain its importance. Note: expect significant double coding with "Developing community buy-in, Present" Do not add sub-codes |
| Present - without trust building | Descriptions of community mobilization that was done without establishing trust and buy-in. Example: Simply informing the community of an intervention Do not add sub-codes |
| Inclusion criteria | Select the relevant reason this intervention / activity was included in the SR. Inclusion criteria into the research project should fall under "sampling frame." Do not use main code, only use sub-codes Do not add sub-codes |
| Community engagement as the intervention | Select the way in which community engagement was used as the intervention itself Do not add sub-codes |
| Developing community buy-in | Descriptions of activities whose primary purpose was the establishment of community buy-in / trust Do not add sub codes |
| Absent | Explicit statements that efforts to establish community buy-in / trust were not made should be assigned this code Example: Authors state that the intervention proceeded without engaging the community Do not add sub-codes |
| Present | If there were activities whose primary purpose was to establish community buy in or trust, select this code Example: Meetings with local leaders to get their support Note: expect significant double coding with "community mobilization, present - with trust building" Do not add sub-codes |
| New cadres | Description of the development of new cadres of community based structures or systems for health outreach Examples: Developing community health workers, health volunteers, or the establishment of committees Do not add sub-codes |
| Community engagement in designing the intervention | Select the type of community engagement that was sought before on the design of the intervention Do not add sub-codes |
| Absent | The community was not given the opportunity to provide feedback or make decisions on the design of the intervention Example: Explicit statements that the community was not consulted on the design of the intervention Do not add sub-codes |
| Community decision making | The community was given the opportunity to make decisions regarding the design of an intervention. This could vary from taking iterative feedback from the community to consensus building on the design of the intervention to the design being community led |
| Absent | The community did not have decision making power. Example: The intervention design was established a priori and not subject to change Do not add sub-codes |
| Community led | The community had the ultimate decision-making power and / or the intervention design was community led. Example: The community identified which barriers would be targeted and developed an action plan Do not add sub-codes |
| Partial | The community had some decision-making power Example: Decisions were made through collaboration between the community, implementers, and / or researchers Do not add sub-codes |
| Community feedback | The community was given the opportunity to provide feedback on the design of the intervention |
| Formative evaluation or stakeholder consultation |  |
| Needs assessment |  |
| Pilot |  |
| There was a previous pilot | There was a pilot in which it is explicitly stated that community feedback was taken Do not add sub-codes |
| This was a pilot | The present study is a pilot in which community feedback is taken |
| Community engagement in implementation | Communities had some opportunity to affect the implementation of the intervention. For example, community is required to spend resources on building health infrastructure and decides whether the intervention will be implemented in their community or those where community members come together to form governance structures such as health committees . OR The community has some responsibility for making decisions regarding the delivery of an intervention. Do not add sub-codes |
| Governance and decisions |  |
| Absent | Explicit statements that the community did not have decision making power. Example: Statements that the community was excluded from implementation. This may be stated as a problem during the discussion. Do not add sub-codes |
| Present | The community has some form of decision making in the implementation of the intervention. This could include a community led governance structure. Example: There was a community group monitoring the implementation of the intervention. Do not add-sub codes |
| Provision of resources | The community provides resources during the implementation Examples: Community provides building materials Do not add sub-codes |
| Component 1 | Each unique component should be coded separately. A unique component is defined as the set of activates that are dependent on one another. Activities within separate components are not dependent on one another. Select the depth of engagement for the initial description of the component but code all subsequent activities to the main code. Example: A sticker based reminder and a digital reminder could each be implemented independently and would be separate components Do not add sub-codes |
| Does not include engagement | Descriptions of interventions that involved no community engagement Example: The production of cold chain transport of vaccines Do not add sub-codes |
| Includes some engagement | Description of activities that involved some community engagement Example: Health clinics holding vaccination days and posting fliers Do not add sub-codes |
| Primary focus is engagement | Description of activities whose primary focus was community engagement Example: The establishment of community health councils Do not add sub-codes |
| Component 10 |  |
| Does not include engagement |  |
| Includes some engagement |  |
| Primary focus is engagement |  |
| Component 2 |  |
| Does not include engagement |  |
| Includes some engagement |  |
| Primary focus is engagement |  |
| Component 3 |  |
| Does not include engagement |  |
| Includes some engagement |  |
| Primary focus is engagement |  |
| Component 4 |  |
| Does not include engagement |  |
| Includes some engagement |  |
| Primary focus is engagement |  |
| Component 5 |  |
| Does not include engagement |  |
| Includes some engagement |  |
| Primary focus is engagement |  |
| Component 6 |  |
| Does not include engagement |  |
| Includes some engagement |  |
| Primary focus is engagement |  |
| Component 7 |  |
| Does not include engagement |  |
| Includes some engagement |  |
| Primary focus is engagement |  |
| Component 8 |  |
| Does not include engagement |  |
| Includes some engagement |  |
| Primary focus is engagement |  |
| Component 9 |  |
| Does not include engagement |  |
| Includes some engagement |  |
| Primary focus is engagement |  |
| Future directions for implementation | Discussion of next steps, how the program could be re-designed, or suggestions for the future Do not add sub-codes |
| Intervention exposure or reach | Discussions of the proportion of the eligible population that the intervention tried to reach. For example, the number of people who received the video (regardless of whether it was actually watched). Do not add sub-codes |
| Objective | Statement of the goal, objective, or target of the intervention Do not add sub-codes |
| Participant views of the intervention | Text related to how participants perceived the intervention |
| Negative views | Participants did not view the intervention positively |
| Positive views | Participants viewed the intervention positively |
| Personnel implementing the program | Description of who implemented the program Do not add sub-codes |
| Target group | Description of the target group for this program Note: This could be different from beneficiary selection if (for example) fathers were provided with information to get them to have their wives immunise their children. Do not add additional sub-codes |
| Uptake | Program uptake is defined as initial engagement with the intervention. This could be attending the first meeting, or expressing interest. Use sub-codes when appropriate and only primary code when the text does not fall into the other two. Do not add additional sub-codes |
| Assessment of uptake | Description of how uptake was assessed Example: Attendance sheets were used to collect information on attendance at the first meeting. Do not add sub-codes Do not add sub-codes |
| Description of uptake | Description of uptake Example: Uptake was high, with 80% of eligible women participating in the intervention Do not add sub-codes |
| Research design | This set of codes describes the research design and provides information on internal and external validity. Do not add sub-codes |
| Author discussion of external validity | Any explicit discussion by authors Do not add sub-codes |
| Conflict of interest | Discussion of conflicts of interests. This could include discussions related to the independence of evaluators and data collectors from the implementers and donors. Select the relevant code indicating presence / absence of a conflict Do not add sub-codes Do not add sub-codes |
| No | Select any statement directly indicating that there was no conflict of interest Example: The data collection team was hired as external contractors Do not add sub-codes |
| Unclear | Select any text that makes a potential conflict of interest unclear Do not add sub-codes |
| Yes | Select any text that indicates a clear conflict of interest Example: The implementers were the data collectors Do not add sub-codes |
| Data collection | Discussion of data collection. Use main code for general discussions and only use sub-codes as appropriate Do not add sub-codes |
| Response accuracy | Any measure or discussion of response accuracy Example: We expect that some of our findings may be related to social desirability bias Do not add sub-codes |
| Retrospective | Text indicating that data collection was retrospective. Include discussion of challenges this may have caused Do not add additional sub-codes |
| Sampling frame | Descriptions of the sampling frame. Only select text related to how people were ultimately enrolled / selected. Text related to geographic distribution of villages, the method of randomisation (e.g. computer generated vs. paper based), and other details about the preparation for selection is not needed. Example: Women of child bearing age were selected when they presented at the clinic. Do not add sub-codes |
| Data limitations | Discussions of the limitations to the data that pose challenges (eg. we could not analyse X because of Y) Do not add additional sub-codes |
| Ethical approval | Statement of ethical approval Do not add sub-codes |
| Future directions for research | Descriptions of future directions for research Do not add sub-codes |
| Other limitations | Text related to limitations in the research design that are not data related. Example: Failure in randomisation Example: Failure in randomisation Do not add sub-codes |
| Uptake and fidelity challenges | This set of codes describes challenges with uptake and fidelity. Most include sub-codes titled "present" and "absent." In each case, present reflects a statement that this challenge occurred and absent reflects a statement that this challenge was not encountered. Add sub-codes if needed |
| Adherence | Discussion of people (not) completing the intervention activities (eg. stopped attending training sessions). Could involve discussions of variability in engagement with the intervention or discussions of continued engagement with the intervention. Do not add sub-codes |
| Absent | There were not challenges related to adherence. ie adherence was high |
| Present | There were problems with adherence. ie adherence was low |
| Administrative | Challenges related to record keeping, monitoring, and other administrative activities Do not add sub-codes |
| Absent | There were not administrative challenges. Example: All paperwork was conducted quickly and on time |
| Present | There were administrative challenges Ex: Paperwork was finished late and this delayed implementation |
| Attrition in the research | People did not complete the research study (eg. did not respond to all rounds of data collection Do not add sub-codes |
| Absent | There was not attrition in the research study. ie attrition was low |
| Present | There was significant attrition in the research study. ie attrition was high |
| Budget | Challenges due to budget limitations Do not add sub-codes |
| Budget limitations in the intervention | The intervention could not be implemented in a desired way due to budget limitations Do not add sub-codes |
| Absent | There were not budgetary limitations to the intervention. Ex: Due to the generous funding of our donor, we were able to..... |
| Present | There were budgetary limitations to the intervention Ex: We had planned X, but due to budget limitations has to Y |
| Budget limitations in the research study | The research study could not be implemented as desired due to budget limitations Do not add sub-codes |
| Absent | There were not budgetary limitations to the research study Ex: Due to the generous funding of our donor, we were able to ...... |
| Present | There were budgetary constraints in the research study Ex: We were not able to collect X data due to limited budget |
| Contamination | Select the appropriate type of contamination described Do not add sub-codes |
| Contamination between intervention and control | The control group was incidentally exposed to the intervention. Example: Nearby villages were aware of education campaigns conducted. |
| Absent | Explicit statements that this contamination did not occur Do not add sub-codes |
| Present | Contamination between intervention and control occurred |
| Contamination by other programs | Other ongoing programs may have affected results Do not add additional sub-codes |
| Absent | There was not contamination by other programs Ex: No other similar programs were functioning in the area |
| Present | There was contamination by other programs Ex: The Red Cross was conducting a similar program in control villages at the time of our intervention |
| Hawthorn effects | Any description of the change in behaviour (real or reported) that is the result of people knowing they are being observed, without any real intent to adopt or maintain these behaviours. Example: Respondents in the UCT group may have been affected by the evaluation process: community awareness about the aims of the project could have affected actual or reported behaviours. Do not add sub-codes |
| Absent | Hawthorn effects were not observed or expected Ex: By using an enumeration team that was unaffiliated with the intervention, we expect to have reduced the chances that people adjusted their behaviour simply due to the enumerator's presence |
| Present | Hawthorn effects were observed or expected Ex: Due to significant marketing around the desirability of certain behaviours, people may have changed how they acted in front of enumerators without actually adopting certain behaviours when they were not observed. |
| Low implementation fidelity | Deviations in implementation of the intervention from what was planned. Note: this must be from what was planned, not simply what would be considered desirable Do not add sub-codes |
| Absent | Fidelity challenges were not present. The intervention was implemented as intended. Ex: The intervention was implemented as intended |
| Present | The intervention was not implemented as intended. Ex: Due to confusion among facilitators, some changed the order in which material was presented. |
| Mobilization | Challenges related to mobilizing people to participate Do not add sub-codes |
| Mobilizing in the intervention | People were not willing to engage with the intervention Do not add sub-codes |
| Absent | Low participation was not a challenge observed. ie participation was high. |
| Present | The challenge of low participation was encountered. ie participation was low |
| Mobilizing in the research | Challenges getting those who participated in the intervention to join the research project. (eg. refuse to be interviewed) Do not add sub-codes |
| Absent | Low participation was not a challenge observed. ie participation was high. |
| Present | The challenge of low participation was encountered. ie participation was low |
| Other Challenges |  |

## Appendix F1: Quantitative risk of bias assessment tool

Experimental studies

| Code | Question | Coding format | Criteria |
| --- | --- | --- | --- |
| General | ID | EPPI ID |  |
| Genera | Study first author | Open answer |  |
| General | Time taken to complete assessment | Minutes |  |
| General | Design type: What type of study design is used? | 1= Randomised controlled trial (RCT) (random assignment to households/individuals) or quasi-RCT  2 = Cluster-RCT (quasi-RCT)  3 = Pseudo-RCT | - |
| General | Methods used for analysis: Which methods are used to control for selection bias and confounding? | 1 = Statistical matching (PSM, CEM, covariate matching)  2 = Difference in differences (DID) estimation methods  3 = IV-regression (2-stage least squares or bivariate probit)  4 = Heckman selection model  5 = Fixed effects or random effects regression  6 = Covariate adjusted estimation  7 = Propensity weighted regression  8 = Comparison of means  9 = Other (please state) | - |
| General | Design and analysis method description | Open answer | Briefly describe the study design and analysis method undertaken by the authors. |
| General | Study population | Open answer | Provide any details in the paper that describe how the study population was selected, answering the question: what is the sampling strategy to recruit participants from that population into the evaluation? |
| General | Type of comparison group | 1=No intervention (service delivery as usual)  2=Other intervention  3=Pipeline (wait-list) control (still service delivery as usual) | Indicate type of comparison group |
| General | Type of comparison group (if other) | Open answer |  |
| General | Ethical clearance | Open answer | Provide any details of ethical research clearances granted. Report unclear if this information is not available. |
| General | Study registration | Open answer | Provide any details of study registration, including registry IDs, etc. |
| 1: Bias arising from randomisation process | 1.1 Was the allocation sequence random? | (1) Yes;  (2) Probably yes;  (3) Probably no;  (4) No;  (5) No information; | 1.1 Was the allocation sequence random?  Answer ‘Yes’ if a random component was used in the sequence generation process. Examples include computer-generated random numbers; reference to a random number table; coin tossing; shuffling cards or envelopes; throwing dice; or drawing lots. Minimization is generally implemented with a random element (at least when the scores are equal), so an allocation sequence that is generated using minimization should generally be considered to be random.  Answer ‘No’ if no random element was used in generating the allocation sequence or the sequence is predictable. Examples include alternation; methods based on dates (of birth or admission); patient record numbers; allocation decisions made by clinicians or participants; allocation based on the availability of the intervention; or any other systematic or haphazard method.  Answer ‘No information’ if the only information about randomization methods is a statement that the study is randomized.  Note: In some situations a judgement may be made to answer ‘Probably no’ or ‘Probably yes’. For example, if the study was large, conducted by an independent trials unit or carried out for regulatory purposes, it may be reasonable to assume that the sequence was random. Alternatively, if other (contemporary) trials by the same investigator team have clearly used non-random sequences, it might be reasonable to assume that the current study was done using similar methods. |
| 1: Bias arising from randomisation process | Question 1.1 answer justification | Open answer | Justification for coding decision  (Include a brief summary of justification for rating, mentioning your response to all sub questions, cite relevant pages). |
| 1: Bias arising from randomisation process | 1.2 Did baseline differences between intervention groups suggest a problem with the randomisation process? | (1) Yes;  (2) Probably yes;  (3) Probably no;  (4) No;  (5) No information; | 1.2 Did baseline differences between intervention groups suggest a problem with the randomization process?  Note that differences that are compatible with chance do not lead to a risk of bias.  Answer ‘No’ if no imbalances are apparent or if any observed imbalances are compatible with chance.  Answer ‘Yes’ if there are imbalances that indicate problems with the randomization process, including:  (1) substantial differences between intervention group sizes, compared with the intended allocation ratio;  or  (2) a substantial excess in statistically significant differences in baseline characteristics between intervention groups, beyond that expected by chance (i.e., on more than 10% of the characteristics reported); or  (3) imbalance in one or more key prognostic factors, or baseline measures of outcome variables, that is very unlikely to be due to chance and for which the between-group difference is big enough to result in bias in the intervention effect estimate.  Also answer ‘Yes’ if there are other reasons to suspect that the randomization process was problematic, such as:  (4) excessive similarity in baseline characteristics that is not compatible with chance; or  (5) surprising absence of one or more key baseline characteristics that would be expected to be reported.  Answer ‘No information’ when there is no useful baseline information available (e.g. abstracts, or studies that reported only baseline characteristics of participants in the final analysis).  Note: Trialists may undertake analyses that attempt to deal with flawed randomization by controlling for imbalances in prognostic factors at baseline. To remove the risk of bias caused by problems in the randomization process, it would be necessary to know, and measure, all the prognostic factors that were imbalanced at baseline. It is unlikely that all important prognostic factors are known and measured, so such analyses will at best reduce the risk of bias. |
| 1: Bias arising from randomisation process | Question 1.2 answer justification | Open answer | Justification for coding decision  (Include a brief summary of justification for rating, mentioning your response to all sub questions, cite relevant pages). |
| 1: Bias arising from randomisation process | Assignment mechanism: Was the allocation or identification mechanism random or as good as random? | (0) Low risk of bias  (1) Medium risk of bias/Some concerns  (2) High risk of bias | Utliize your answers for questions 1.1 and 1.2 to determine the overall score. |
| 1: Assignment mechanism - Justification | Assignment justification | Open answer | Justification for coding decision  (Include a brief summary of justification for rating, mentioning your response to all sub questions, cite relevant pages). |
| 2: Unit of analysis - Assessment | Unit of analysis: Is unit of analysis in cluster allocation addressed in standard error calculation? | 1=Yes  2=No  3=Not reported/unclear  4=Not applicable | Score "Yes" if Unit of Analysis = Unit of Randomization OR if UoA ≠ UoR and standard errors are clustered at the UoR level OR data is collapsed to the UoR level    Score "Not reported/unclear" if not enough information is provided on the way the standard errors were calculated or what the unit of analysis is.    Score "Not applicable" if it is not a cluster RCT.    Score "No" otherwise.Score "Yes" if Unit of Analysis = Unit of Randomization OR if UoA ≠ UoR and standard errors are clustered at the UoR level OR data is collapsed to the UoR level    Score "Not reported/unclear" if not enough information is provided on the way the standard errors were calculated or what the unit of analysis is.    Score "Not applicable" if it is not a cluster RCT.    Score "No" otherwise. |
| 2: Unit of analysis - Justification | Question 2 answer justification (provide page numbers) | Open answer | - |
| 3. Bias due to missing outcome data (panel data) | 3.1. Was there attrition, if yes, then what was the rate in treatment and control/comparison groups? | (1) Yes;  (2) Probably yes;  (3) Probably no;  (4) No;  (5) No information; | 3.1. Was there attrition, if yes, then what was the rate in treatment and control/comparison groups?  In case of attrition, answer 'yes' or 'probably yes', otherwise answer 'no' or 'probably no'.  If there is no discussion on attrition then score 'no information'.  Specify the rate of attrition by the study arms.  Specify the attrition rate assumed for power calculations.  Has the study discussed the ability of the follow-up study to detect the hypothesised outcome effect with the sample size attained? |
| 3. Bias due to missing outcome data – Justification (panel data) | Question 3.1 answer justification | Open answer | Justification for coding decision  (Include a brief summary of justification for rating, mentioning your response to all sub questions, cite relevant pages). |
| 3. Bias due to missing outcome data (panel data) | 3.2. Does the study establish that attrition is randomly distributed (e.g. by examining correlation with determinants of outcomes, in both treatment and comparison groups or by presenting data showing balance on key characteristics across treatment and control)? | (1) Yes;  (2) Probably yes;  (3) Probably no;  (4) No;  (5) No information; | 3.2. Does the study establish that attrition is randomly distributed (e.g. by examining correlation with determinants of outcomes, in both treatment and comparison groups or by presenting data showing balance on key characteristics across treatment and control)?  Score 'yes' or 'probably yes' if balance on key characteristics between attritors and non-attritors for each intervention group (including control) using baseline data has been presented and finds that there is no systematic difference on at least observable dimensions.  Score 'no' or 'probably no' if the study finds that there is a systematic difference on observables between attritors and non-attritors in any of the intervention groups.  If the baseline balance table between atritors and non-attritors is not presented or discussed then score 'no information'. |
| 3. Bias due to missing outcome data – Justification (panel data) | Question 3.2 answer justification | Open answer | Justification for coding decision  (Include a brief summary of justification for rating, mentioning your response to all sub questions, cite relevant pages). |
| 3. Bias due to missing outcome data – (panel data) | 3.3. If there is non-random attrition, have authors used convincing statistical techniques to identify and adjust for the attrition bias? | (1) Yes;  (2) Probably yes;  (3) Probably no;  (4) No;  (5) No information;  (6) Not applicable | 3.3. If there is non-random attrition, have authors used convicing statistical techniques to identify and adjust for the attrition bias?  If the study has used convincing parametric or non-parametric techniques (like ignorable maximum likelihood, Manski-Lee bounds) to identify and adjust for attrition bias, then score 'yes' or 'probably yes'. If the techniques used are not convincing then score 'no' or 'probably no'. If the study does not discuss using any statiscal techniques for identifying and adjusting for attrition bias then score 'no information'.  If there is no non-random attrition, then this question does not apply and the score should be 'not applicable' |
| 3. Bias due to missing outcome data – (panel data) | Question 3.3 answer justification | Open answer | Justification for coding decision  (Include a brief summary of justification for rating, mentioning your response to all sub questions, cite relevant pages). |
| 3. Bias due to missing outcome data – Justification (panel data) | Bias due to missing outcome data justification | Open answer | Justification for coding decision  (Include a brief summary of justification for rating, mentioning your response to all sub questions, cite relevant pages). |
| 3. Bias due to missing outcome data - Assessment |  | (0) Low risk of bias  (1) Medium risk of bias/Some concerns  (2) High risk of bias | Utliize your answers for questions 3.1 to 3.3 to determine the overall score. |
| 3. Bias due to missing outcome data (repeated cross section) | 3.1. Was there difference in sample size across baseline and follow-up surveys among treatment and control groups? If yes, what was the rate of difference among treatment and control/comparison groups? Was it lower or higher than baseline? | (1) Yes;  (2) Probably yes;  (3) Probably no;  (4) No;  (5) No information; | 3.1. Was there difference in sample size across baseline and follow-up surveys among treatment and control groups? If yes, what was the rate of difference among treatment and control/comparison groups? Was it lower or higher than baseline?  In case of difference, answer 'yes' or 'probably yes', otherwise answer 'no' or 'probably no'.  If there is no information to assess the differences, then score 'no information'.  Specify the rate of difference by the study arms.  Specify the non-compliance and/or attrition rate assumed for power calculations.  Has the study discussed the ability of the follow-up study to detect the hypothesised outcome effect with the sample size attained? |
| 3. Bias due to missing outcome data – Justification (repeated cross section) | Question 3.1 answer justification | Open answer | Justification for coding decision  (Include a brief summary of justification for rating, mentioning your response to all sub questions, cite relevant pages). |
| 3. Bias due to missing outcome data (repeated cross section) | 3.2. Does the study establish that the sampling frame for baseline and follow-up surveys is same? Are the clusters (eg health center) and sub-clusters (eg. villages) the same across baseline and follow-up surveys? | (1) Yes;  (2) Probably yes;  (3) Probably no;  (4) No;  (5) No information; | 3.2. Does the study establish that the sampling frame for baseline and follow-up surveys is same? Are the clusters (eg health center) and sub-clusters (eg. villages) the same across baseline and follow-up surveys?  Score 'yes' or 'probably yes' if sampling frame (clusters and sub-clusters) are the same for baseline and follow-up surveys  Score 'no' or 'probably no' if the clusters or sub-clusters were different across baseline and follow-up surveys.  If there is no information on sampling frame across baseline or follow-up surveys, then score 'no information'. |
| 3. Bias due to missing outcome data – Justification (repeated cross section) | Question 3.2 answer justification | Open answer | Justification for coding decision  (Include a brief summary of justification for rating, mentioning your response to all sub questions, cite relevant pages). |
| 3. Bias due to missing outcome data –(repeated cross section) | 3.3. Was the sample selection of respondents carried out in the same way as in the baseline? Was the intended number of respondents in the clusters/sub-clusters same across baseline and follow-up surveys? | (1) Yes;  (2) Probably yes;  (3) Probably no;  (4) No;  (5) No information; | 3.3. Was the sample selection of respondents carried out in the same way as in the baseline? Was the intended number of respondents in the sub-clusters same across baseline and follow-up surveys?  Score 'yes' or 'probably yes' if the study used same listing process for selection of respondents and their intended number was same across baseline and follow-up surveys,  Score 'no' or 'probably no' if the study used different listing process for selection of respondents or their intended number was different across baseline and follow-up surveys.  If there is no information on the selection process of respondents then score 'no information'. |
| 3. Bias due to missing outcome data - Justification (repeated cross section) | Question 3.3 answer justification | Open answer | Justification for coding decision  (Include a brief summary of justification for rating, mentioning your response to all sub questions, cite relevant pages). |
| 3. Bias due to missing outcome data – Assessment (repeated cross section) | Bias due to missing outcome data | (0) Low risk of bias  (1) Medium risk of bias/Some concerns  (2) High risk of bias | Utliize your answers for questions 3.1 to 3.3 to determine the overall score using the criteria below |
| Bias due to missing outcome data – Justification (repeated cross section) | Bias due to missing outcome data justification | Open answer | Justification for coding decision  (Include a brief summary of justification for rating, mentioning your response to all sub questions, cite relevant pages). |
| 3. Bias due to missing outcome data - Assessment | (0) Low risk of bias  (1) Medium risk of bias/Some concerns  (2) High risk of bias |  | Utliize your answers for questions 3.1 to 3.3 to determine the overall score. |
| 3.Bias due to missing outcome data - Justification | Bias due to missing outcome data justification | Open answer | Justification for coding decision  (Include a brief summary of justification for rating, mentioning your response to all sub questions, cite relevant pages). |
| 4: Bias due to deviations from intended interventions - Assessment | 4.1. Was the study adequately protected against spill-overs/ contamination? | (1) Yes;  (2) Probably yes;  (3) Probably no;  (4) No;  (5) No information; | 4.1. Was the study adequately protected against spill-overs/ contamination?  Score “Yes” if the intervention is unlikely to spill-over to comparisons (e.g. participants and non-participants are geographically and/or socially separated from one another and general equilibrium effects are not likely) and that the treatment and comparisons are isolated from other interventions which might explain changes in outcomes.  Score “No” if allocation was at the individual or classroom level and there are likely spill-overs within households and communities which are not controlled for (such as using trasferable vouchers), or other interventions likely to affect outcomes operating at the same time in either group.  Score “no information” if spill-overs and/or contamination are not discussed |
| 4: Bias due to deviations from intended interventions - Justification | Question 4.1 answer justification | Open answer | Justification for coding decision  (Include a brief summary of justification for rating, mentioning your response to all sub questions, cite relevant pages). |
| 4: Bias due to deviations from intended interventions - Assessment | 4.2. Was the process of being monitored free from bias? | (1) Yes;  (2) Probably yes;  (3) Probably no;  (4) No;  (5) No information; | 4.2. Was the process of being monitored free from bias?  Score "Yes" if the authors state explicitly that the process of monitoring the intervention is blinded, or argue convincingly why it is not likely that intervention delivery could affect the performance of participants in treatment and comparison groups in different ways (such as resulting in Hawthorne or John Henry effects). or there is nothing in the surveys that might have given the control participants an idea of what the other group might receive (e.g. provide cash transfer to the intervention area participants or reveal informmation that they did not have before or they did but there is no risk that this has changed their behaviours;  Score "No" if the the authors do not use an appropriate method to prevent Hawthorne and John Henry Effects (e.g. blinding of monitoring or other methods to ensure consistent monitoring across groups) and there is a risk that the intervention delivery or survey process could have changed the behaviours of treatment and comparison groups in different ways.  Score "no information" if the authors do not discuss potential bias due to monitoring of intervention.  Hawthorne effects may result where participants know that they are being observed and John Henry Effects may result from participant knowledge of being compared. |
| 4: Bias due to deviations from intended interventions - Justification | Question 4.2 answer justification | Open answer | Justification for coding decision  (Include a brief summary of justification for rating, mentioning your response to all sub questions, cite relevant pages). |
| 4: Bias due to deviations from intended interventions - Assessment | 4.3. Was an appropriate analysis used to estimate the effect of assignment to intervention? | (1) Yes;  (2) Probably yes;  (3) Probably no;  (4) No;  (5) No information; | 44.3. Was an appropriate analysis used to estimate the effect of assignment to intervention?  Both intention-to-treat (ITT) analyses and modified intention to treat (mITT) analyses excluding participants with missing outcome data should be considered appropriate. (Missing outcome data are addressed in a separate domain). Both ‘as treated’ analyses (in which trial participants are grouped according to the intervention that they received, rather than according to their assigned intervention) and naïve ‘per-protocol’ analyses (excluding trial participants who did not receive their assigned intervention) should be considered inappropriate. Analyses excluding eligible trial participants post-randomization should also be considered inappropriate, but post-randomization exclusions of ineligible participants (when eligibility was not confirmed until after randomization, and could not have been influenced by intervention group assignment) can be considered appropriate.  Score "Yes" if ITT or modified ITT analysis has been used, AND common analysis methods are used AND any covariates imbalanced at baseline are included as covariates in the analysis.  Score "No" otherwise. You may also score no if they use an uncommon analysis methods, such as 3SLS, or if they analyse dichotomous outcome using continuous methods (e.g., using linear regression instead of using the more appropriate logit or probit models) or if the is baseline imbalance but those covariates are not included in the model.  If the analytical specification is not provided score "no information" |
| 4: Bias due to deviations from intended interventions - Justification | Question 4.3 answer justification | Open answer | Justification for coding decision  (Include a brief summary of justification for rating, mentioning your response to all sub questions, cite relevant pages). |
| 4: Bias due to deviations from intended interventions - Assessment | Deviations from intended interventions: Spill-overs, cross-overs, contamination and performance bias: was the study adequately protected against spill-overs, cross-overs, contamination, and performance bias? | (0) Low risk of bias  (1) Medium risk of bias/Some concerns  (2) High risk of bias | Utliize your answers for questions 4.1 to 4.3 to determine the overall score. |
| 4: Bias due to deviations from intended interventions - Justification | Deviations justification | Open answer | Justification for coding decision  (Include a brief summary of justification for rating, mentioning your response to all sub questions, cite relevant pages).    For example, intervention groups are geographically separated, authors use intention to treat estimation or instrumental variables to account for non-adherence, and survey questions are not likely to expose individuals in the control group to information about desirable behaviours (‘survey effects’). |
| 5. Outcome measurement bias | 5.1 Could the measurement of the outcome be different between the study arms? | (1) Yes;  (2) Probably yes;  (3) Probably no;  (4) No;  (5) No information; | 5.1 Could the measurement of the outcome be different between the study arms?  Comparable methods of outcome measurement (data collection) involve the same measurement methods and thresholds, used at comparable time points. Differences between intervention groups may arise because of ‘diagnostic detection bias’ in the context of passive collection of outcome data, or if an intervention involves additional visits to a healthcare provider, leading to additional opportunities for outcome events to be identified.  Score 'yes' or 'probably yes' if the outcomes have been measured at different times periods for treatment and control arms or have used different methods of measurement.  Score 'no' or 'probably no' otherwise.  If no information is provided on the the time period and methods of data collection in treatment and control arms, then score 'no information' |
| 5. Outcome measurement bias - Justification | Question 5.1 answer justification | Open answer | Justification for coding decision  (Include a brief summary of justification for rating, mentioning your response to all sub questions, cite relevant pages). |
| 5. Outcome measurement bias | 5.2. Were the outcome assessors (enumerators) blinded to the intervention assignment? | (1) Yes;  (2) Probably yes;  (3) Probably no;  (4) No;  (5) No information; | 5.2. Were the outcome assessors (enumerators) blinded to the intervention assignment?  Score ‘yes’ or 'probably yes' if outcome assessors were blinded to intervention status and 'no' or 'probably no' otherwise.  If no information is provided on blinding of the outcome assessors, then score 'no information'.  Note that for participant-reported outcomes, the outcome assessor is the study participant. |
| 5. Outcome measurement bias - Justification | Question 5.2 answer justification | Open answer | Justification for coding decision  (Include a brief summary of justification for rating, mentioning your response to all sub questions, cite relevant pages). |
| 5. Outcome measurement bias | 5.3. If not blinded, could assessment of the outcome have been influenced by knowledge or administration of intervention received? | (1) Yes;  (2) Probably yes;  (3) Probably no;  (4) No;  (5) No information; | 5.3. If not blinded, could assessment of the outcome have been influenced by knowledge or administration of intervention received?  Knowledge or administration of the assigned intervention could influence participant-reported outcomes (such as level of pain, immunization outcomes using recall), observer-reported outcomes involving some judgement, and intervention provider decision outcomes. They are unlikely to influence observer-reported outcomes that do not involve judgement, for example all-cause mortality.  For participant-reported outcomes, the assessment of outcome is potentially influenced by knowledge or administration of intervention received, leading to a judgement of at least ‘Some concerns’. Review authors will need to judge whether it is likely that participants’ reporting of the outcome was influenced by knowledge or administration of intervention received, in which case risk of bias is considered to be high.  Score 'yes' or 'probably yes' if assessment of outcome could have been influenced by knowledge of intervention received (e.g., if ANY of the immunization coverage outcomes are assessed through caregiver recall) and 'no' or 'probably no' otherwise (e.g., if immunization coverage outcome is assessed by immunization card).  If no information is available to make the assessment then score 'no information'. |
| 5. Outcome measurement bias - Justification | Question 5.3 answer justification | Open answer | Justification for coding decision  (Include a brief summary of justification for rating, mentioning your response to all sub questions, cite relevant pages). |
| 5. Outcome measurement bias - Assessment | Outcome measurement bias: Was the study free from biases in outcome measurement? | (0) Low risk of bias  (1) Medium risk of bias/Some concerns  (2) High risk of bias | Utliize your answers for questions 5.1 to 5.3 to determine the overall score. |
| 5. Outcome measurement bias - Justification | Outcome measurement justification | Open answer | Justification for coding decision  (Include a brief summary of justification for rating, mentioning your response to all sub questions, cite relevant pages). |
| 6. Reporting bias | 6.1. Is a pre-analysis plan or protocol available which provides sufficient detail? | (1) Yes;  (2) Probably yes;  (3) Probably no;  (4) No;  (5) No information; | 6.1. Is a pre-analysis plan or protocol available which provides sufficient detail?  Score 'yes' if they reference a preanalysis plan, and 'no' otherwise. |
| 6. Reporting bias - Justification | Question 6.1 answer justification | Open answer | Justification for coding decision  (Include a brief summary of justification for rating, mentioning your response to all sub questions, cite relevant pages). |
| 6. Reporting bias | 6.2. Were all primary and secondary outcomes reported as per the pre-analysis plan/protocol? | (1) Yes;  (2) Probably yes;  (3) Probably no;  (4) No;  (5) No information; | 6.2. Were all primary and secondary outcomes reported as per the pre-analysis plan/protocol?  Score “Yes” or "probably yes" if there is no evidence that outcomes were selectively reported (e.g. results for all relevant outcomes in the methods section are reported in the results section)  Score “No” or "probably no" if some important outcomes are subsequently omitted from the results or the significance and magnitude of important outcomes was not assessed or if multiple measurements of an outcome were made but only one or a subset is reported on the basis of the results (e.g. statistical significance)  Score “No information” if pre-analysis not available or the outcome intentions are not reported in sufficient detail to enable an assessment. |
| 6. Reporting bias - Justification | Question 6.2 answer justification | Open answer | Justification for coding decision  (Include a brief summary of justification for rating, mentioning your response to all sub questions, cite relevant pages). |
| 6. Reporting bias | 6.3. Do reported results for the outcomes correspond to all intended analyses? | (1) Yes;  (2) Probably yes;  (3) Probably no;  (4) No;  (5) No information; | 6.3. Do reported results for the outcomes correspond to all intended analyses?  A particular outcome domain may be analysed in multiple ways. Examples include: unadjusted and adjusted models; final value vs change from baseline vs analysis of covariance; transformations of variables; different definitions of composite outcomes (e.g. ‘major adverse event’); conversion of continuously scaled outcome to categorical data with different cut-points; different sets of covariates for adjustment; and different strategies for dealing with missing data. Application of multiple methods generates multiple effect estimates for a specific outcome domain. If multiple estimates are generated but only one or a subset is reported on the basis of the results (e.g. statistical significance), there is a high risk of bias in the fully reported result.  Answer ‘No’ or ‘Probably No’ if:  There is clear evidence (usually through examination of a trial protocol or statistical analysis plan) that a domain was analysed in multiple ways, but data for only one or a subset of analyses is fully reported (without justification), and the fully reported result is likely to have been selected on the basis of the results. Selection on the basis of the results arises from a desire for findings to be newsworthy, sufficiently noteworthy to merit publication, or to confirm a prior hypothesis. For example, trialists who have a preconception or vested interest in showing that an experimental intervention is beneficial may be inclined to selectively report analyses that are favourable to the experimental intervention. Score probably no if authors do not report both adjusted and unadjusted models.  Answer ‘Yes’ or ‘Probably Yes’ if:  There is clear evidence (usually through examination of a trial protocol or statistical analysis plan) that all reported results for the outcome domain correspond to all intended analyses.  or  Not all intended analyses have been reported but authors have convincingly justified the reasons for not doing so.  or  There is only one possible way in which the outcome domain can be analysed (hence there is no opportunity to select from multiple analyses).  or  Analyses are inconsistent across different reports on the same trial, but the trialists have provided the reason for the inconsistency and it is not related to the nature of the results.  Answer ‘No information’ if:  Analysis intentions are not available, or the analysis intentions are not reported in sufficient detail to enable an assessment, and there is more than one way in which the outcome domain could have been analysed. |
| 6. Reporting bias - Justification | Question 6.3 answer justification | Open answer | Justification for coding decision  (Include a brief summary of justification for rating, mentioning your response to all sub questions, cite relevant pages). |
| 6. Reporting bias - Assessment | Analysis reporting: Was the study free from selective analysis reporting? | (0) Low risk of bias  (1) Medium risk of bias/Some concerns  (2) High risk of bias | Utliize your answers for questions 6.1 to 6.3 to determine the overall score. |
| 6. Reporting bias - Justification | Analysis reporting justification | Open answer | Justification for coding decision  (Include a brief summary of justification for rating, mentioning your response to all sub questions, cite relevant pages). |
| 7. Other bias - Assessment | Other risks of bias Is the study free from other sources of bias? | 1= Yes,  4 = No | - |
| 7. Other bias - Justification | Other bias justification | Open answer | Justification for coding decision  (Include a brief summary of justification for rating, mentioning your response to all sub questions, cite relevant pages). For example, information is collected using a different survey instrument in different intervention groups; measurement of the intervention received in unclear. |
| 8. Blinding - observers - Assessment | Blinding of participants? | 1 = Yes  2 = No  8 = unclear  9 = N/A | If there is no information, code NO. If there is information but it is ambiguous, code UNCLEAR. |
| 8. Blinding - analysts - Assessment | Blinding of data analysts? | 1 = Yes  2 = No  8 = unclear  9 = N/A | If there is no information, code NO. If there is information but it is ambiguous, code UNCLEAR. |
| 8. Blinding - method(s) | Method(s) used to blind | Open answer (including describe method of placebo control) 9 = N/A | Describe method(s) used to blind |
| 9. External validity - Random Sampling | Was random sampling used? | 1 = Yes  2 = No | Was a random sampling method used (meaning everyone in the population had an equal chance of being selected for the study - note that this is different than random allocation to treatment versus control group)?  Score yes if: (a) there is a sampling frame. The sampling frame is the actual list of individuals that the sample will be drawn from. Ideally, it should include the entire target population (and nobody who is not part of that population). AND  (b) probibility sampling is used (e.g., simple random, stratified random, cluster random, etc.)  Score no if: individuals are selected based on non-random criteria, and not every individual has a chance of being included (e.g., a convenience sample, a purposive sample, a snowball sample, a voluntary response sample, etc.). |
| 9. External validity - Random Sampling Justification | Justification for answer to random sampling question | Open answer | Justification for coding decision  (Include a brief summary of justification for rating, mentioning your response to all sub questions, cite relevant pages).Describe the sampling technique used. |
| 9. External validity - Assessment | External validity | Open answer | a) What do authors say about external validity? Note any additional information related to generalizability. |

## Appendix F2: Qualitative risk of bias appraisal tool

**Critical appraisal of qualitative studies tool**

This tool provides specific questions you should answer with regards to the study being appraised. Every question has three possible responses, which correspond to ‘strong’, ‘weak’, or ‘none’ – these are phrased slightly differently depending on the question. This process is subjective and relies on a basic level of familiarity with qualitative research methods. However, to further structure the assessment, each question is accompanied by a description of criteria you should consider before making your choice.

While attempting to answer a question, you may find that a study contains many of the relevant elements, but not all. Give this paper the benefit of the doubt – if the element being assessed is strong except for one minor element, choose the ‘strong’ option.

There is room for comments after each question. You are encouraged to utilize this space, though only the multiple answer response is mandatory. Use the space to document your thoughts if you feel uneasy about the choice made, or if you have any ideas which would help as we refine and revise the assessment tool. There is also room at the end for any additional notes on the paper as a whole.

The questions are arranged according to the order in which a standard academic article is arranged: Introduction, Methodology, Results, Discussion. This will allow you to answer the questions as you go over each section. That said, some documents will not follow this structure, and some academic articles are more fluid about where certain descriptions are located within the text. After you read through the article, go over all the questions once more to see whether any elements you thought were missing actually showed up in an unexpected place.

* Required

Assessor: *

Paper Title: *

Authors: *

Year: *

**Introduction**

*1. Is the research aim clearly stated? **

Look for this in either the abstract or the introduction. In the best studies, this will be an explicit formulation of the research aims/questions. There may be several. Other studies do not formally state the research aims as such, but they are clearly evident from the text. Note: you might find it useful to write down these research aims, as many of the other questions relate to them.

• Yes, a strong statement

• Yes, but an unclear or weak statement

• No

Question 1 – Notes:

*2. Is there a description of the context in which the study takes place? **

The study should make some reference to the geographical, temporal, or societal greater context within which the study topic is located. A study within a well-established area of research could likely be situated quite specifically. An exploratory study might cover a topic where there is little descriptive context to draw upon.

• Yes, a strong description

• Yes, but a weak description

• No

Question 2 – Notes:

*3. Is there a clear link to relevant literature? **

Literature should be cited not just in general, but in specific relation to the topic of the paper. In assessing whether the literature is relevant and sufficient, consider: Are most of the cited papers about the same area or topic as the study? Are both qualitative and quantitative studies cited? Are there at least some papers published within the last 10 years?

• Yes, a clear strong link

• Yes, but it could be improved

• No

Question 3 – Notes:

*4. Is there a clear link to theory? **

This can be achieved in many ways, and while it will often be in the introduction, it may show up elsewhere, so keep your eyes peeled. This requires a reference to a specific theory. There are two main ways this could happen: A reference could be made to an established theoretical framework (from the social sciences, from public health, etc.) which has been used to investigate similar questions, or which the authors think might apply in this case. The second possibility is that the authors describe a theoretical framework they have created or adapted themselves which they used in structuring their study, or that they refer to in considering their results.

• Yes, a strong one

• Yes, but a weak one

• No

Question 4 – Notes:

**Methodology - Sampling**

*5. Is there a description of the sampling procedure? **

Are there details about how sampling was actually conducted? A helpful way to consider this is whether you would be able to conduct this sampling yourself if you were to replicate the study. Every sampling approach has its own logic, but even for the more straightforward qualitative sampling, there should be some mention of this. Note: This question does not evaluate whether this sampling is actually appropriate (see question 6). It only asks whether we have a clear idea of the process of sampling, at all.

• Yes, a strong description

• Yes, but a partial description

• No

Question 5 – Notes:

*6. Is the sampling strategy appropriate for the aims of the research? **

This question asks whether the sampling strategy was the right for one for choosing the participants/locations/etc. that could yield relevant information. If there is a strong claim of generalizability, was the sampling appropriate for achieving this? If the study attempts to present the views of a group, did sampling actually capture the people the study needed to hear from? Are there any groups which appear to be missing but clearly should have been spoken to in order to meet the research aims? Has saturation been achieved, and can we tell how this was established? Note that this is not a question of whether the method of collection (interview, focus group, etc.) would have yielded strong data – just whether the people recruited to the study were the right people.

• Yes, very appropriate

• Yes, but not completely appropriate

• No

Question 6 – Notes:

*7. Are sample characteristics sufficiently reported? **

The basic characteristics are often gender, location, ethnicity, and other demographic variables, but consider whether there are other sample characteristics specific to the question which should be reported. This might be clear by this point, but you might also want to look ahead and see whether the paper discusses any relevant characteristics that you would want to know about the sample in general (or you could return to this at the end).

• Yes, there's a sufficient description

• There's some description, but not enough

• No

Question 7 – Notes:

**Methodology - Data Collection**

*8. Is it clear how data were collected? **

Each method requires its own information. If interviews were used, is there detail on locations, presence of others, length of interview, questions asked, and other such details? For participant observation, is there a description of the time spent in the field and the activities partaken in? For focus groups, is there detail on the number of participants, questions asked and setting?

• Yes, data collection is clearly described

• Yes, but more details are needed

• No

Question 8 – Notes:

*9. Are the methods of data recording reported? **

This could be as simple as ‘the audio of interviews was taped’, ‘focus group summaries were written during the discussion’, or ‘interviews were transcribed, translated, and the translation was then verified’. Any of these would merit at least a ‘weak’ response, and more detail would be considered ‘strong’.

• Yes, and reported well

• Yes, but only briefly mentioned

• No

Question 9 – Notes:

*10. Did the collection of the data address the research aims? **

Given the data needed to answer the question, is the method chosen actually well suited to obtaining such data? If we have knowledge of the questions in an interview guide or a focus group guide, are all the relevant subject areas covered? This isn’t about whether there might be some problems in the collection (a bad interviewer could screw up even the best interview guide) but whether the method of data collection, if employed properly, would actually capture relevant data.

• Yes, completely

• Yes, but not entirely

• No

Question 10 – Notes:

**Methodology - Analysis**

*11. Are the methods of analysis explicitly stated? **

This should include a basic statement as to the approach used (thematic analysis, grounded theory, etc.) but also a breakdown of the steps taken (different rounds of coding, intermittent checks for quality, revisions, etc.)

• Yes, and stated well

• Yes, but more detail is needed

• No

Question 11 – Notes:

*12. Were there any inbuilt checks to assure the quality of the analysis? **

Some but not all of the following could be present. Was there more than one researcher involved in the analysis? Was there independent peer review during the analysis? Were inter-coder or intra-coder reliability checks conducted? Did the team meet regularly to discuss the analytic process? Did the researcher keep a journal of their thoughts and decisions?

• Yes, there were clear, strong checks throughout

• Yes, but they were weak checks

• No

Question 12 – Notes:

*13. Was there reflection on bias and positionality? **

Have the authors considered their potential biases in relation to the study? This should be a specific statement, and there should be mention of steps that were taken to address this, even if as basic as taking the time to write reflective notes or engaging in a group discussion of the issues.

• Yes, a clear reflection with detail provided

• Yes, but there's only a brief mention of this

• No

Question 13 – Notes:

**Methodology - Researchers**

*14. Are there any details about the people who conducted the sampling, data collection, and analysis?*

Not much is required, but look out for mentions of the training these people had, their positions/titles, their experience, etc.

• Yes, plenty

• Yes, a little

• No

Question 14 – Notes:

**Results**

*15. Is there enough data to support the claims? **

Variable, but consider whether major claims/themes are supported by more than a single quote or other type of original data. Look at whether the data presented is contextualised, and whether it demonstrates the claim that the authors suggest it does.

• Yes, there's plenty of clear, contextualised, relevant data

• There's some data, but it's weak

• No - there's little or no data presented that is supports the claims.

Question 15 – Notes:

*16. Are diverse viewpoints considered? **

At a basic level, we need to know who speakers are if they are quoted. This could be as minimal as (’22-year-old’, or ‘Mother, Group A’). Same goes for summaries with no direct quotes – it should be clear whose viewpoint the data comes from. The majority of the data presented will demonstrate the main themes/arguments, but no study is without some dissenting voices or unusual opinions. Are these ever mentioned? If there were many participants, but it appears that most quotes come from one or two individuals, there should be a reasonable explanation for why this is the case – and what it might mean for the study.

• Yes, thoroughly

• Yes, only occasionally

• No

Question 16 – Notes:

*17. Is there evidence which addresses every research aim? **

Look back to the research aims. Is there data relating to each one of them? If not, is there a clear reason for this? If a study ended up not finding data relevant to every aim, but acknowledges that this is the case and tries to account for the reason, this should still be marked as a yes. A 'null' result is still a result - sometimes collection and analysis can be excellent, yet no clear answer is found. If this appears to be the case, the study can still be considered 'strong'.

• Yes, every aim has some related evidence

• Yes, but some aims have distinctly less attending to them

• No

Question 17 – Notes:

**Discussion**

*18. Has the question been answered? **

A simple test is to go back to the research aims, formulate them as questions (if they’re not already established as such) and see whether the authors have provided you with answers that are reasonable given the data presented. It should be clear how the data presented is appropriate and convincing evidence for the conclusion. In a strong paper, the authors will make a clear and logical statement of how the results provide an answer to the question. A weaker paper might not cover all the questions, or might suggest questionable links between the data in the results and the conclusions reached.

• Yes, with a clear, logical, thorough answer

• Yes, but not well

• No

Question 18 – Notes:

*19. Are relevant literature, theory, or practice discussed in relation to the results? **

A good paper doesn’t need to discuss all of these, but it should relate to at least one. Did it match up or challenge a theory? Does it have implications for practice? Are the results now situated within the broader literature?

• Yes, and discussed well

• Yes, but only briefly or generally discussed

• No

Question 19 – Notes:

*20. Has there been any triangulation? **

This takes many forms, but the basic question is – do the authors present any other sources of data which support or contradict their claims? These could be quantitative/qualitative/mixed studies, some form of evaluation of the conclusions by the participants themselves, comparison to other data recorded, etc. This could be in the results section or the discussion, so have another look.

• Yes, strong triangulation

• Yes, weak triangulation

• No

Question 20 – Notes:

*21. Are weaknesses considered? **

Mentioning them is a good step, but a strong paper will also argue how these weaknesses were attended to.

• Yes, major weaknesses considered and attended to

• Yes, but weaknesses are mentioned without much discussion

• No

Question 21 – Notes:

*Ethics*

22. Have ethical issues been taken into consideration? *

At the very basic level, there should be a description of informed consent. Also look for approval from an IRB (or other ethics committee as applicable), and whether issues of benefit/harm and confidentiality/secrecy were discussed with participants.

• Yes

• No

Question 22 – Notes:

General comments and thoughts:

Remember to look back at the questions before submitting.

Having read the paper as a whole, and having considered each question separately, you might now also have a clearer idea as to the answers to some questions. Have another look, particularly for questions where you noted some thoughts or concerns. You may want return to question 7 in particular, as you may now have a clearer idea of what sample characteristics should have been reported.

## Appendix F3: Critical appraisal tool for cost evidence

| **S.No.** | **Questions** | **Response** |
| --- | --- | --- |
| 1a. | Is the form of economic evaluation clearly stated? Indicate (CBA/CEA/CUA/cost-minimization, or innovative approaches: Social Return on Investment (SROI), Multi-Criteria Appraisal (MCA). Response options [CBA, CEA, CUA, SROI, MCA, or N/A if only "total cost", also ok to insert descriptive results that do not conform, e.g. “cost transfer ratio”] | Yes, No |
| 1b. | Is the perspective of the costing stated? | Yes, No |
| 1d. | Cost data sources? | Yes, No |
| 1e.1 | Are unit costs reported (in Table) | Yes, No |
| 1e.2 | Are cost ingredients listed? | Yes, No |
| 1e.3 | Total cost description | Yes, No |
| 1f. | Total cost reported? | not reported, Value if reported |
| 1h. | Was cost per child immunised reported? | Yes, No, If yes, Cost |
| 1j. | What exchange rate was used for currency adjustments? [e.g. 1.2 USD to 1 Euro] | value if given, Not reported |
| 1j. | Exch rate year (estimated) | Year |
| 1k. | Is time horizon for costs clearly stated? [Operationalise - was a base year of the costing reported? | Yes, No, If yes: year |
| 7a. | Sensitivity analysis? [yes/ no] | n/a, Description if yes |

## Appendix G: Table 1: Characteristics of the included studies

| **Author** | **Country** | **Summary of intervention** | **Engagement type** | **Study design** | **Duration (in months)** | **Outcomes** |
| --- | --- | --- | --- | --- | --- | --- |
| Admassie 2009 | Ethiopia | Formation of a cadre of community-based health extension workers and using community resources for construction of health posts. | Multiple (EII + EAI) | PSM | 48 | FIC, BCG, DPT3, OPV1, OPV2, OPV3, measles, morbidity |
| Adamu 2019 | Nigeria | Quality improvement programme where health workers use iterative processes to develop localised and contextually relevant plans to resolve health service delivery and demand bottlenecks. | Multiple (EID + EII) | ITS | 1 | Dropouts |
| Alhassan 2019 | Ghana | Using a bottom-up approach, the intervention first recruited and trained community groups to identify service delivery gaps in healthcare facilities. | Multiple (EII + EAI) | RCT | 10 | FIC |
| Andersson 2009 | Pakistan | Community dialogues to address barriers to vaccination. The guidelines for the dialogue were created after consultation with the intended beneficiaries. | Multiple (EID + EAI) | RCT | 8 | Knowledge about immunisation, attitude about immunisation, community norms, readiness to vaccinate, household norms & decision-making measles, DPT3 |
| Arifeen 2009 | Bangladesh | Formation of a cadre of village health volunteers and enlisting support of local religious leaders to convey messages about child health. | EAI | RCT | 71 | Measles, mortality |
| Assegaai 2018 | South Africa | Lay community-based workers were formalised as community health workers and served as a part of the outreach teams. | EAI | DID | 36 | FIC, measles, morbidity |
| Banerjee 2010 | India | Provision of immunisation services and incentives to caregivers. A trusted community-based organisation was a key stakeholder in design and delivery of the intervention. | Treatment 1: EAI  Treatment 2:  Multiple (EID + EAI) | RCT | 18 | FIC, BCG, partial immunisation |
| Banerjee 2020 | India | This evaluation tested two different interventions:  1. Incentives to caregivers. The community's feedback was solicited on the kind of incentive. 2. Community influencers were identified to spread information about immunisation. | EID  EAI | RCT | 14 | Knowledge about immunisation, attitude about immunisation, FIC, DPT1, DPT2, DPT3, measles |
| Banwat 2015 | Nigeria | Female members of the community whose children are fully immunised were nominated in each community to serve as peer educators. | EAI | CBA | - | FIC, knowledge about immunisation, attitude about immunisation, readiness to vaccinate |
| Biemba 2016 | Zambia | A national policy to create a cadre of well-trained and motivated community-based health workers. | EAI | DID | 23 | FIC, morbidity |
| Björkman 2009 | Uganda | Communities were involved in monitoring the quality of health services and the performance of health service providers. | EII | RCT | 0.16 | FIC, BCG, OPV0, OPV3, DPT1, DPT3, measles, Partial routine immunisation, mortality |
| Bolam 1998 | Nepal | Training for community health workers and midwives which was developed in collaboration with health workers and experts. | EID | RCT | 3 | FIC |
| Borkum 2014  Carmichael 2019 (linked study) | India | Performance-based incentives to frontline workers. The nature of incentives was decided upon in consultation with the frontline workers. | EID | RCT | 12 | Health card availability, CHW capacity, FIC, BCG, DPT1, DPT2, DPT3, OPV1, OPV2, OPV3, measles, partial immunisation, timeliness |
| Calderón-Ortiz & Mejía-Mejía 1996 | Mexico | Creation of a community-based cadre of volunteers to register and track children in the community for immunisation. | EAI | CBA | 4 | FIC, BCG, DPT3, OPV3, measles |
| Carnell 2014 | Ethiopia | Formation of a cadre of community health workers to mobilize the community and encourage uptake of health services. | EAI | DID | 60 | DPT3, measles |
| Costa-Font 2017 | India | Establishment of the village health and sanitation committees to monitor health service provision at the community level. | EAI | IV | - | BCG, DPT1, OPV0 |
| Demilew 2020 | Ethiopia | A poster/stamp system that reminded health workers of the child’s immunisation status and simultaneously encouraged caregivers to immunise their children. The intervention was designed in consultation with health workers. | Multiple (EID + EAI) | RCT | 17 | FIC, BCG, DPT1, DPT2, DPT3, partial immunisation |
| Dipeolu 2017 | Nigeria | Text message reminders to mothers regarding immunisation schedule. The messages were field tested with mothers to get the content right. | EID | DID | 9 | Knowledge about immunisation, attitude about immunisation, timeliness |
| Domek 2019 | Guatemala | SMS text messages to caregivers. A prior feasibility and acceptability study was conducted for the intervention. | EID | RCT | 2 | Timeliness |
| Engineer 2016 | Afghanistan | Pay-for-performance bonuses paid quarterly to health workers. The bonus amount was revised after receiving health worker feedback. | EID | RCT | 24 | Experience & satisfaction with health services, Formal HW motivation, capacity & performance, DPT3 |
| Findley 2013 | Nigeria | Formation of a cadre of community volunteers to facilitate group discussions on health and track/register women/children for health services. | EAI | DID | 24 | FIC |
| Gibson 2017 | Kenya | SMS reminders and monetary incentives to caregivers. A feasibility study was conducted in 2013 for this intervention. | EID | RCT | 12 | Community norms, FIC, BCG, DPT1, DPT2, DPT3, OPV0, OPV1, OPV2, measles, timeliness |
| Goel 2012 | India | A multi-component campaign which involved women groups in awareness generation to improve health service uptake. | EAI | DID | 48 | FIC |
| Gurley 2020 | India | Community members were trained to design and produce culturally appropriate, ‘hyper-local’ videos to promote health seeking behaviours. | Multiple (EID + EAI) | RCT | 11 | Knowledge about immunisation, attitude about immunisation, FIC, DPT3, partial immunisation, timeliness, dropouts |
| Herrera-Almanza 2018 | Madagascar | Community-based primary health care services intervention that included the deployment of volunteer community health workers in remote areas. | Multiple (EII + EAI) | DID | 26 | Health card availability, OPV3, DPT3, measles, mortality, partial immunisation |
| Igarashi 2010 | Zambia | The GMP+ sessions were conducted by medical personnel from Public Health Centers. During these sessions, community volunteers provided some operational and managerial support to ensure the effective implementation of the sessions. | EII | CBA | 43 | FIC, timeliness, attitude about immunisation, community norms |
| Janssens 2011 | India | Dissemination of health promoting messages to women in the community who are encouraged to further spread the awareness. | EAI | IV | 56.4 | DPT3, measles |
| Johri 2020 | India | Interventions, designed through formative research, to increase caregiver knowledge and adherence to childhood immunisation. | EID | RCT | 3 | Knowledge about immunisation, Awareness of place, time, schedule for vacc., attitude about immunisation |
| Lee 2015 | Zambia | Creation of a new cadre of frontline workers from the community, called community health assistants, to provide primary health care services. | EAU | RCT | 3 | Formal HW motivation, capacity & performance, BCG, OPV3, measles, timeliness, CHW capacity, morbidity |
| Mayumana 2017 | Tanzania | Payment-for-performance scheme for health facilities. Health workers and health facility governing committees decided the allocation of funds. | EII | DID | 30 | Stockouts |
| Memon 2015 | Pakistan | Formation of community health committees to promote perinatal and new-born care. Formative research informed the intervention design. | Multiple (EID + EAI) | DID | 16 | FIC |
| Modi 2019 | India | The mHealth intervention package consisting of mobile phone-based job aids for community health workers. The intervention was piloted in 2015. | EID | RCT | 12 | DPT3, morbidity |
| Mohanan 2020 | India | Social accountability interventions to promote community-based collective action to improve delivery of health and nutrition services to children. | EAI | RCT | 12 | Experience & satisfaction with health services, attitudes about health providers, formal health worker supply, FIC, BCG, DPT3, OPV1, OPV3, IPV, measles, morbidity, mortality |
| More 2012 | India | Urban slum-dweller women's groups used community dialogues to address barriers to improving perinatal health. | EAI | RCT | 36 | Mortality |
| More 2017 | India | The intervention comprised multiple activities like home visits to caregivers, groups meetings, community events and other supportive services. | EAI | RCT | 24 | Health card availability, attitudes about health providers, FIC, BCG, measles, partial immunisation |
| Morris 2004 | Honduras | Monetary vouchers to women in the communities and setting up of community-based committees to oversee health service quality and access. | EAI | RCT | 24 | DPT1, measles |
| Murthy 2019 | India | Voice call reminders to pregnant women and caregivers. The message content was tested for appropriateness through community focused groups. | EID | RCT | 21 | Knowledge about immunisation, FIC |
| Nagar 2018 | India | A digital pendant-based health record of the child and a voice call reminder system. A formative study was conducted in 2016 and communities were consulted on the design of the pendant. | EID | RCT | 3 | Timeliness |
| Nagar 2020 | India | A digital pendant-based health record of the child. Health providers used a mobile application to scan the pendant to update the child’s medical history. Prior formative research informed the intervention design. | EID | RCT | 20 | FIC, DPT1, DPT2, DPT3 |
| Nzioki 2017 | Kenya | Formation of a cadre of community health workers. | EAI | CBA | 0 | FIC |
| Oche 2011 | Nigeria | Group meetings with caregivers and dialogues with community leaders to improve uptake of routine immunization services. | EAI | DID | 9 | Knowledge about immunisation, DPT1, DPT3, dropouts |
| Okeke 2017 | Nigeria | A national scheme to create, train and deploy a cadre of midwives to serve underserved rural and remote populations in Nigeria. | Multiple (EII + EAI) | DID | 40 | BCG, DPT3, OPV3, measles, mortality |
| Okoli 2014 | Nigeria | A conditional cash transfer program to encourage uptake of health services. Community groups were consulted while deciding the cash amount. | EID | ITS | 7 to 18 | OPV0 |
| Olayo 2014 | Kenya | Formation of a cadre of community health workers who then facilitated dialogue at the community level and supported other community-based workers. | Multiple (EII + EAI) | DID | 24 | Health card availability, DPT1, DPT3, measles |
| Olken 2014 | Indonesia | Block grants for maternal and child health that incorporated relative performance incentives were implemented in villages through creation of village-level health committees. | Multiple (EII + EAI) | RCT | 18 to 30 | FIC, morbidity, mortality |
| Oyo-Ita 2020 | Nigeria | A multi-component intervention involving traditional and religious leaders for engaging communities in planning and delivery of immunisation services. | Multiple (EII + EAI) | RCT | 18 | FIC, partial immunisation, Timeliness |
| Pramanik 2020 | India | Trained facilitators from local NGOs interacted with the communities to enable communities to leverage their own strengths for addressing their concerns related to child health. | EAI | RCT | 13 | Knowledge about immunisation, attitude about immunisation, attitudes about health providers, health card availability, FIC, DPT1, DPT2, DPT3, timeliness, dropouts |
| Rahman 2008 | Pakistan | Mental health support program with counselling sessions for pregnant and post-partum women. Prior intervention pilots informed the intervention design. | EID | RCT | 11 | FIC, morbidity |
| Rahman 2016 | Bangladesh | New cadre of community health workers delivered essential maternal, neonatal, and child healthcare and nutrition services. | EAI | DID | 48 | FIC, morbidity |
| Rao 2014 | India | Creation of a cadre of community health workers to improve basic health outcomes through community engagement. | EAI | DID | 60 | BCG, DPT3, OPV3, measles, FIC, partial immunisation, supply of CHWs |
| Robertson 2013 | Zimbabwe | Use of cash transfers for behaviour change. Local NGO and community leaders were involved in beneficiary targeting and compliance monitoring. The intervention was also tested for feasibility during a prior study. | Multiple (EID + EII) | RCT | 12 | FIC, community norms, OPV0 |
| Roy 2008 | Bangladesh | Rural maintenance programme recruited and trained women for road maintenance, health awareness, numeracy, human rights, gender equity, health and nutrition, and business management. | EAI | DID | 11 | FIC, BCG, DPT1, DPT2, DPT3, OPV0, OPV1, OPV2, OPV3, measles, partial immunisation |
| Saggurti 2018 | India | Formation of health-focused self-help groups with women of reproductive age coming from the most marginalized communities. | EAI | DID | 2 | Timeliness |
| Sankar 2013 | India | Formation of committees with representatives of the community, local government and service providers to ensure better convergence and coordination of service delivery. | Multiple (EII +EAI) | DID | 30 | FIC, BCG, DPT1, DPT2, DPT3, OPV0, OPV1, OPV2, OPV3, measles, partial immunisation, timeliness |
| Seth 2018 | India | The study evaluated two different interventions: role of compliance-linked incentives versus text messaging to improve childhood immunisations. Incentive amount was determined after input was received from the local investigators as well as the community workers. | EID | RCT | 9.7 | Partial immunisation, timeliness, attitude about immunisation, attitudes about health providers |
| Shukla 2018 | Afghanistan | Community representatives along with health officials identify the health needs of the communities and communicate those to the service providers. | EII | DID | 6 | Supply of CHWs, DPT3 |
| Siddiqi 2020 | Pakistan | Visual reminders to caregivers in the form of wearable bracelets for the child. The bracelets were designed in consultation with the caregivers. | EID | RCT | 12 | DPT3, measles |
| Tandon 1988 | India | Enlisting community-based volunteers to motivate and encourage family members to utlise maternal and child health services. | EAI | CBA | 120 | FIC, BCG, DPT2, DPT3, OPV2 |
| USAID 2008 | Ethiopia | Creation of a cadre of community health promoters to carry out behaviour change communication activities in the communities. | EAI | CBA | 48 | FIC, BCG, DPT1, DPT3, OPV3, morbidity, health card availability, measles, dropouts |
| Webster 2019 | Uganda | Community-based outreach and follow-up with caregivers to improve immunisation uptake and reduce defaulters. | EII | RCT | 12 | Health card availability, BCG, DPT1, DPT2, DPT3, OPV0, OPV1, OPV2, OPV3, IPV, measles, partial immunisation, timeliness, attitude about immunisation, dropouts, morbidity, mortality |
| Younes 2014 | Bangladesh | The intervention involved 162 women’s groups who used participatory approaches to discuss maternal and neonatal health issues. | EAI | DID | 20 | FIC, morbidity |

Note: The engagement classification is as follows:

EAI: Engagement as intervention

EID: Engagement in Design

EII: Engagement in implementation autonomy

### References to included studies

Adamu, A. A., Uthman, O. A., Gadanya, M. A., & Wiysonge, C. S. (2019). Implementation and evaluation of a collaborative quality improvement program to improve immunization rate and reduce missed opportunities for vaccination in primary health‐care facilities: A time series study in Kano, Nigeria. *Expert Review of Vaccines, 18*(9), 969–991. <https://doi.org/10.1080/14760584.2019.1647782>

Admassie, A., Abebaw, D., & Woldemichael, A. D. (2009). Impact evaluation of the Ethiopian health services extension programme. *Journal of Development Effectiveness, 1*(4), 430–449. <https://doi.org/10.1080/19439340903375724>

Alhassan, R. K., Nketiah‐Amponsah, E., Ayanore, M. A., Afaya, A., Salia, S. M., Milipaak, J., Ansah, E. K., & Owusu‐Agyei, S. (2019). Impact of a bottom‐up community engagement intervention on maternal and child health services utilization in Ghana: A cluster randomised trial. *BMC Public Health, 19*(1), 791. <https://doi.org/10.1186/s12889-019-7180-8>

Andersson, N., Cockcroft, A., Ansari, N. M., Omer, K., Baloch, M., Ho Foster, A., Shea, B., Wells, G. A., & Soberanis, J. L. (2009). Evidence‐based discussion increases childhood vaccination uptake: A randomised cluster controlled trial of knowledge translation in Pakistan. *BMC International Health and Human Rights, 9*(S1), S8. <https://doi.org/10.1186/1472-698X-9-S1-S8>

Arifeen, S. E., Emdadul Hoque, D. M., Akter, T., Rahman, M., Enamul Hoque, M., Begum, K., Chowdhury, E. K., Khan, R., Blum, L. S., Ahmed, S., Hossain, M. A., Siddik, A., Begum, N., Rahman, Q. S., Haque, T. M., Billah, S. M., Islam, M., Rumi, R. A., Law, E., … Black, R. E. (2009). Effect of the integrated management of childhood illness strategy on childhood mortality and nutrition in a rural area in Bangladesh: A cluster randomised trial. *The Lancet, 374*, 393–403. <https://doi.org/10.1016/S0140-6736(09)60828-X>

Assegaai, T., Reagon, G., & Schneider, H. (2018). Evaluating the effect of ward‐based outreach teams on primary healthcare performance in North West Province, South Africa: A plausibility design using routine data. *South African Medical Journal, 108*(4), 329–335. <https://doi.org/10.7196/SAMJ.2018.v108i4.12755>

Banerjee, A. V., Duflo, E., Glennerster, R., & Kothari, D. (2010). Improving immunisation coverage in rural India: Clustered randomised controlled evaluation of immunisation campaigns with and without incentives. *BMJ, 340*(1), c2220. <https://doi.org/10.1136/bmj.c2220>

Banerjee, A., Chandrasekhar, A., Duflo, E., Dalpath, S., Floretta, J., Jackson, M., Kannan, H., Schrimpf, A., & Shrestha, M. (2020). *Evaluating the impact of interventions to improve full immunisation rates in Haryana, India (Impact Evaluation Report 126).* International Initiative for Impact Evaluation (3ie). https://doi.org/10.23846/TW10IE126

Banwat, M. E., Lar, L.A. Abok, I.A., & Yiltok, E.S. (2015). Effect of peer education on knowledge, attitude and completeness of childhood routine immunization in a rural community of Plateau State. Research *Journal of Health Sciences, 3*(4), 264–274. <https://www.ajol.info/index.php/rejhs/article/view/143332>

Biemba, G., Yeboah‐Antwi, K., Vosburg, K. B., Prust, M. L., Keller, B., Worku, Y., Zulu, H., White, E., & Hamer, D. H. (2016). Effect of deploying community health assistants on appropriate treatment for diarrhoea, malaria and pneumonia: Quasi‐experimental study in two districts of Zambia. *Tropical Medicine & International Health, 21*(8), 985–994. <https://doi.org/10.1111/tmi.12730>

Björkman, M., & Svensson, J. (2009). Power to the people: Evidence from a randomized field experiment on community‐based monitoring in Uganda. *Quarterly Journal of Economics*, *124*(2), 735–769. <https://doi.org/10.1162/qjec.2009.124.2.735>

Bolam, A., Manandhar, D. S., Shrestha, P., Ellis, M., & Costello, A. M. dL. (1998). The effects of postnatal health education for mothers on infant care and family planning practices in Nepal: A randomised controlled trial. *BMJ, 316*(7134), 805–811. <https://doi.org/10.1136/bmj.316.7134.805>

Borkum, E., Rangarajan, A., Rotz, D., Sridharan, S., Sethi, S., & Manoranjini, M. (2014). *Evaluation of the Team‐Based Goals and Performance Based Incentives (TBGI) INNOVATion in Bihar (Mathematica Policy Research Reports).* Mathematica Policy Research. <https://ideas.repec.org/p/mpr/mprres/d8e1097122ff47a6bf42580c82677834.html>

Calderón‐Ortiz, R., & Jesus, M. (1996). Estrategia de contratacion permanente dentro del programa de vacunacion universa. *Salud Pública de México, 38*(4), 243–248. <https://www.redalyc.org/articulo.oa?id=10638404>

Carnell, M. A., Dougherty, L., Pomeroy, A. M., Karim, A. M., Mekonnen, Y. M., & Mulligan, B. E. (2014). Effectiveness of scaling up the ‘three pillars’ approach to accelerating MDG 4 progress in Ethiopia. *Journal of Health, Population and Nutrition, 32*(4), 549–563. <https://www.ncbi.nlm.nih.gov/pmc/articles/PMC4438684/>

Costa‐Font, J., & Parmar, D. (2017). *Political Agency and public healthare: Evidence from India* *(CESifo Working Papers 6640).* UNU‐WIDER. <https://doi.org/10.35188/UNU-WIDER/2016/179-6>

Demilew, A., Girma, M., McElwee, E., Datta, S., Barofsky, J., and Disasa, T. (2021). *Impacts of supportive feedback and nonmonetary incentives on child immunisation in Ethiopia* *(3ie Impact Evaluation Report 134).* International Initiative for Impact Evaluation (3ie). <https://doi.org/10.23846/TW10IE134>

Dipeolu, I. O. (2017). *Effect of mobile‐phone reminder test messages on mothers' knowledge and completion of routine immunisation in rural areas of Oyo State, Nigeria* [PhD Thesis, University of Ibadan]. <https://library.adhl.africa/handle/123456789/11865>

Domek, G. J., Contreras‐Roldan, I. L., Bull, S., O'Leary, S. T., Ventura, G. A. B., Bronsert, M., Kempe, A., & Asturias, E. J. (2019). Text message reminders to improve infant immunization in Guatemala: A randomized clinical trial. *Vaccine, 37*(42), 6192–6200. <https://doi.org/10.1016/j.vaccine.2019.08.046>

Engineer, C.Y., Dale, E., Agarwal, A., Agarwal, A., Alonge, O., Edward, A., Gupta, S., Schuh, H. B., Burnham, G., & Peters, D. H. (2016). Effectiveness of a pay‐for‐performance intervention to improve maternal and child health services in Afghanistan: A cluster‐randomized trial. *International Journal of Epidemiology, 45*(2), 451–459. <https://doi.org/10.1093/ije/dyv362>

Findley, S. E., Uwemedimo, O. T., Doctor, H. V., Green, C., Adamu, F., & Afenyadu, G. Y. (2013). Early results of an integrated maternal, newborn, and child health program, Northern Nigeria, 2009 to 2011. *BMC Public Health, 13*(1), 1034. <https://doi.org/10.1186/1471-2458-13-1034>

Gibson, D. G., Ochieng, B., Kagucia, E. W., Were, J., Hayford, K., Moulton, L. H., Levine, O. S., Odhiambo, F., O'Brien, K. L., & Feikin, D. R. (2017). Mobile phone‐delivered reminders and incentives to improve childhood immunisation coverage and timeliness in Kenya (M‐SIMU): A cluster randomised controlled trial. *The Lancet Global Health, 5*(4), e428–e438. <https://doi.org/10.1016/S2214-109X(17)30072-4>

Goel, S., Dogra, V., Gupta, S. K., Lakshmi, P. V. M., Varkey, S., Pradhan, N., Krishna, G., & Kumar, R. (2012). Effectiveness of muskaan ek abhiyan (the smile campaign) for strengthening routine immunization in Bihar, India. *Indian Pediatrics, 49*(2), 103–108. <https://doi.org/10.1007/s13312-012-0023-7>

Gurley, N., Shearer, J., Srivastava, Y., Mahapatra, S., & Desmond, M. (2020). *Impacts of community‐led video education to increase vaccination coverage in Uttar Pradesh, India (Impact Evaluation Report 125).* International Initiative for Impact Evaluation (3ie). <https://doi.org/10.23846/TW10IE125>

Herrera‐Almanza, C., & Rosales‐Rueda, M. F. (2018). Reducing the cost of remoteness: community‐based health interventions and fertility choices. *Journal of Health Economics, 73*(September), 102365. <https://doi.org/10.1016/j.jhealeco.2020.102365>

Igarashi, K., Satoshi, S., Yasuyuki, F., Naohito, T., Mbwili, M. C., Bushimbwa, T., & Hiroshi, S. (2010). The impact of an immunization programme administered through the growth monitoring programme plus as an alternative way of implementing integrated management of childhood illnesses in urban‐slum areas of Lusaka, Zambia. *Transactions of the Royal Society of Tropical Medicine and Hygiene, 104*(9), 577–582. <https://doi.org/10.1016/j.trstmh.2010.05.008>

Janssens, W. (2011). Externalities in program evaluation: The impact of a women's empowerment program on immunization. *Journal of the European Economic Association, 9*(6), 1082–1113. <https://doi.org/10.1111/j.1542-4774.2011.01041.x>

Johri, M., Chandra, D., Kone, K. G., Sylvestre, M.‐P., Mathur, A. K., Harper, S., & Nandi, A. (2020). Social and behavior change communication interventions delivered face‐to‐face and by a mobile phone to strengthen vaccination uptake and improve child health in rural India: Randomized pilot study. *JMIR MHealth and UHealth, 8*(9), 20356. <https://doi.org/10.2196/20356>

Lee, S. S. (2015). *Three field experiments on incentives for health workers* [PhD Thesis, Harvard University]. <https://dash.harvard.edu/handle/1/17467500>

Mayumana, I., Borghi, J., Anselmi, L., Mamdani, M., & Lange, S. (2017). Effects of payment for performance on accountability mechanisms: Evidence from Pwani, Tanzania. *Social Science & Medicine, 179*(April), 61–73. <https://doi.org/10.1016/j.socscimed.2017.02.022>

Memon, Z. A., Khan, G. N., Soofi, S. B., Baig, I. Y., & Bhutta, Z. A. (2015). Impact of a community‐based perinatal and newborn preventive care package on perinatal and neonatal mortality in a remote mountainous district in Northern Pakistan. *BMC Pregnancy and Childbirth, 15*(1), 106. <https://doi.org/10.1186/s12884-015-0538-8>

Modi, D., Dholakia, N., Gopalan, R., Venkatraman, S., Dave, K., Shah, S., Desai, G., Qazi, S. A., Sinha, A., Pandey, R. M., Anand, A., Desai, S., & Shah, P. (2019). MHealth intervention ‘ImTeCHO' to improve delivery of maternal, neonatal, and child care services—A cluster‐randomized trial in tribal areas of Gujarat, India. *PLOS Medicine, 16*(10), 1002939. <https://doi.org/10.1371/journal.pmed.1002939>

Mohanan, M., Rajan, V. S., Swanson, K., & Thirumurthy, H. (2020). *Information and facilitation interventions for accountability in health and nutrition: Evidence from a randomized trial in India (ERID Working Paper 295)*. Duke University. <https://www.ssrn.com/abstract=3544786>

More, N.S., Bapat, U., Das, S., Alcock, G., Patil, S., Porel, M., Vaidya, L., Fernandez, A., Joshi, W., & Osrin, D. (2012). Community mobilization in Mumbai slums to improve perinatal care and outcomes: A cluster randomized controlled trial. *PLoS Medicine, 9*(7), 1001257. <https://doi.org/10.1371/journal.pmed.1001257>

More, N.S., Das, S., Bapat, U., Alcock, G., Manjrekar, S., Kamble, V., Sawant, R., Shende, S., Daruwalla, N., Pantvaidya, S., & Osrin, D. (2017). Community resource centres to improve the health of women and children in informal settlements in Mumbai: A cluster‐randomised, controlled trial. *The Lancet Global Health, 5*(3), e335–e349. <https://doi.org/10.1016/S2214-109X(16)30363-1>

Morris, S. S., Flores, R., Olinto, P., & Medina, J. M. (2004). Monetary incentives in primary health care and effects on use and coverage of preventive health care interventions in rural Honduras: Cluster randomised trial. *The Lancet, 364*(9450), 2030–2037. <https://doi.org/10.1016/S0140-6736(04)17515-6>

Murthy, N., Chandrasekharan, S., Prakash, M. P., Kaonga, N. N., Peter, J., Ganju, A., & Mechael, P. N. (2019). The impact of an MHealth voice message service (MMitra) on infant care knowledge, and practices among low‐income women in India: Findings from a pseudo‐randomized controlled trial. *Maternal and Child Health Journal, 23*(12), 1658–1669. <https://doi.org/10.1007/s10995-019-02805-5>

Nagar, R., Ambiya, M. S., Singh, P., Abdullah, H., Banshiwal, V., Stone, L., Manjanatha, D., Venkat, P., Purawat, D., Supatkar, V., Singh, A., Dalal, S., & Shahnawaz, M. (2020). *Impacts of a novel mHealth platform to track maternal and child health in Udaipur, India (3ie Impact Evaluation Report 129)*. International Initiative for Impact Evaluation (3ie). <https://doi.org/10.23846/TW10IE129>.

Nagar, R., Venkat, P., Stone, L. D., Engel, K. A., Sadda, P., & Shahnawaz, M. (2018). A cluster randomized trial to determine the effectiveness of a novel, digital pendant and voice reminder platform on increasing infant immunization adherence in rural Udaipur, India. *Vaccine, 36*(44), 6567–6477. <https://doi.org/10.1016/j.vaccine.2017.11.023>

Nzioki, J. M., Ouma, J., Ombaka, J. H., & Onyango, R. O. (2017). Community health worker interventions are key to optimal infant immunization coverage, evidence from a pretest‐posttest experiment in Mwingi, Kenya. *Pan African Medical Journal, 28*, 21. <https://doi.org/10.11604/pamj.2017.28.21.11255>

Oche, M. O., Umar, A. S., Ibrahim, M. T. O., & Sabitu, K. (2011). An assessment of the impact of health education on maternal knowledge and practice of childhood immunization in Kware, Sokoto State. *Journal of Public Health and Epidemiology, 3*(10), 440–447. <https://academicjournals.org/article/article1379497483_Oche%20et%20al.pdf>

Okeke, E., Glick, P., Abubakar, I. S., Chari, A. V., Pitchforth, E., Exley, J., Bashir, U., Setodji, C., Gu, K., & Onwujekwe, O. (2017). *Better obstetrics in rural Nigeria: Evaluating the midwives service scheme (3ie Impact Evaluation Report 56)*. International Initiative for Impact Evaluation (3ie). <https://www.3ieimpact.org/evidence-hub/publications/impact-evaluations/better-obstetrics-rural-nigeria-evaluating-midwives>

Okoli, U., Morris, L., Oshin, A., Pate, M. A., Aigbe, C., & Muhammad, A. (2014). Conditional cash transfer schemes in Nigeria: Potential gains for maternal and child health service uptake in a national pilot programme. *BMC Pregnancy and Childbirth, 14*(1), 408. <https://doi.org/10.1186/s12884-014-0408-9>

Olayo, R., Wafula, C., Aseyo, E., Loum, C., & Kaseje, D. (2014). A quasi‐experimental assessment of the effectiveness of the community health strategy on health outcomes in Kenya. *BMC Health Services Research, 14*(S1), S3. <https://doi.org/10.1186/1472-6963-14-S1-S3>

Olken, B. A., Onishi, J., & Wong, S. (2014). Should aid reward performance? Evidence from a field experiment on health and education in Indonesia. *American Economic Journal: Applied Economics, 6*(4), 1–34. <https://doi.org/10.1257/app.6.4.1>

Oyo‐Ita, A., Bosch‐Capblanch, X., Ross, A., Hanlon, P., Oku, A., Esu, E., Ameh, S., Oduwole, B., Arikpo, D., & Meremikwu, M. (2020). *Impacts of engaging communities through traditional and religious leaders on vaccination coverage in Cross River State, Nigeria (Impact Evaluation Report 127)*. International Initiative for Impact Evaluation (3ie). <https://doi.org/10.23846/TW10IE127>

Pramanik, S., Ghosh, A., Goswami, A., Das, T., Nanda, R., Forth, P., & Albert, S. (2020). *Impacts of the stimulate, appreciate, learn, and transfer community engagement approach to increase immunisation coverage in Assam, India (3ie Impact Evaluation Report 13).* International Initiative for Impact Evaluation (3ie). <https://doi.org/10.23846/TW10IE130>

Rahman, A., Malik, A., Sikander, S., Roberts, C., & Creed, F. (2008). Cognitive behaviour therapy‐based intervention by community health workers for mothers with depression and their infants in rural Pakistan: A cluster‐randomised controlled trial. *The Lancet, 372*(9642), 902–909. <https://doi.org/10.1016/S0140-6736(08)61400-2>

Rahman, M., Yunus, F. M., Shah, R., Jhohura, F. T., Mistry, S. K., Quayyum, T., Aktar, B., & Afsana, K. (2016). A controlled before‐and‐after perspective on the improving maternal, neonatal, and child survival program in rural Bangladesh: An impact analysis. *PLoS One, 11*(9), 0161647. <https://doi.org/10.1371/journal.pone.0161647>

Rao, T. (2014). The impact of a community health worker program on childhood immunization: Evidence from India's ‘ASHA’ workers. <http://www.ssrn.com/abstract=2444391>

Robertson, L., Mushati, P., Eaton, J. W., Dumba, L., Mavise, G., Makoni, J., Schumacher, C., Crea, T., Monasch, R., Sherr, L., Garnett, G. P., Nyamukapa, C., & Gregson, S. (2013). Effects of unconditional and conditional cash transfers on child health and development in Zimbabwe: A cluster‐randomised trial. *The Lancet, 381*(9874), 1283–1292. <https://doi.org/10.1016/S0140-6736(12)62168-0>

Roy, S. K., Bilkes, F., Islam, K., Ara, G., Tanner, P., Wosk, I., Rahman, A. S., Chakraborty, B., Jolly, S. P., & Khatun, W. (2008). Impact of pilot project of Rural Maintenance Programme (RMP) on destitute women: CARE, Bangladesh. *Food and Nutrition Bulletin, 29*(1), 67–75. <https://doi.org/10.1177/156482650802900108>

Saggurti, N., Atmavilas, Y., Porwal, A., Schooley, J., Das, R., Kande, N., Irani, L., & Hay, K. (2018). Effect of health intervention integration within women's self‐help groups on collectivization and healthy practices around reproductive, maternal, neonatal and child health in rural India. *PLoS One, 13*(8), 0202562. <https://doi.org/10.1371/journal.pone.0202562>

Sankar, D. (2013). *Improving early childhood development through community mobilization and integrated planning for children. Results from the evaluation of Bachpan Program, Ratlam District, Madhya Pradesh, India (Discussion Paper Series 59)*. The World Bank. <http://crossasia-repository.ub.uni-heidelberg.de/3467/>

Seth, R., Akinboyo, I., Chhabra, A., Qaiyum, Y., Shet, A., Gupte, N., Jain, A. K., & Jain, S. K. (2018). Mobile phone incentives for childhood immunizations in rural India. *Pediatrics, 141*(4), e20173455. <https://doi.org/10.1542/peds.2017-3455>

Shukla, M. (2018). Impact of a health governance intervention on provincial health system performance in Afghanistan: A quasi‐experimental study. *Health Systems & Reform, 4*(3), 249–266. <https://doi.org/10.1080/23288604.2018.1477536>

Siddiqi, D. A., Ali, R. F., Munir, M., Shah, M. T., Khan, A. J., & Chandir, S. (2020). Effect of vaccine reminder and tracker bracelets on routine childhood immunization coverage and timeliness in urban Pakistan (2017–18): A randomized controlled trial. *BMC Public Health, 20*(1), 1086. <https://doi.org/10.1186/s12889-020-09088-4>

Tandon, B. N., & Sahai, A. (1988). Immunization in India: Contribution of integrated child development services scheme to expanded programme of immunization. *Journal of Tropical Pediatrics, 34*(6), 309–312. <https://doi.org/10.1093/tropej/34.6.309>

USAID. (2008). *Essential services for health in Ethiopia: Final report November 2003–September 2008*. The United States Agency for International Development. <https://publications.jsi.com/JSIInternet/Inc/Common/_download_pub.cfm?id=10111&lid=3>

Webster, J., Landegger, J., Bruce, J., Malunda, D., Chantler, T., Kumakech, E., Schmucker, L., Kiapi, L., Kozuki, N., & Olorunsaiye, C. (2019). *Impacts of IRC's Fifth Child Community Engagement Strategy to Increase Immunisation in Northern Uganda (3ie Grantee Final Report)*. International Initiative for Impact Evaluation (3ie). <https://www.3ieimpact.org/sites/default/files/2019-02/gfr-TW10.1018-IRC-Imunization-Program-Uganda.pdf>

Younes, L., Houweling, T. A. J., Azad, K., Kuddus, A., Shaha, S., Haq, B., Nahar, T., Hossen, M., Beard, J., Copas, A., Prost, A., Costello, A., & Fottrell, E. (2014). The effect of participatory women's groups on infant feeding and child health knowledge, behaviour and outcomes in rural Bangladesh: A controlled before‐and‐after study. *Journal of Epidemiology and Community Health, 69*(4), 374–381 <https://doi.org/10.1136/jech-2014-204271>

### Included qualitative studies

Adamu, A. A., Uthman, O. A., Gadanya, M. A., & Wiysonge, C. S. (2020). Using the consolidated framework for implementation research (CFIR) to assess the implementation context of a quality improvement program to reduce missed opportunities for vaccination in Kano, Nigeria: A mixed methods study. *Human Vaccines and Immunotherapeutics, 16*(2), 465–475. <https://doi.org/10.1080/21645515.2019.1654798>

Anwari, Z., Shukla, M., Maseed, B. A., Wardak, G. F., Sardar, S., Matin, J., Rashed, G. S., Hamedi, S. A., Sahak, H., Aziz, A. H., Boyd‐Boffa, M., & Trasi, R. (2015). Implementing people‐centred health systems governance in 3 provinces and 11 districts of Afghanistan: A case study. *Conflict and Health, 9*:2. <https://doi.org/10.1186/1752-1505-9-2>

Baba‐Ari, F., Eboreime, E. A., & Hossain, M. (2018). Conditional cash transfers for maternal health interventions: Factors influencing uptake in North‐Central Nigeria. *International Journal of Health Policy and Management, 7*(10), 934–942. <https://doi.org/10.15171/ijhpm.2018.56>

Balarajan, Y., & Reich, M. R. (2016). Political economy of child nutrition policy: A qualitative study of India's integrated child development services (ICDS) scheme. *Food Policy, 62*, 88–98. <https://doi.org/10.1016/j.foodpol.2016.05.001>

Billah, S. M., Hoque, D. E., Rahman, M., Christou, A., Mugo, N. S., Begum, K., Tahsina, T., Rahman, Q. S., Chowdhury, E. K., Haque, T. M., Khan, R., Siddik, A., Bryce, J., Black, R. E., & El Arifeen, S. (2018). Feasibility of engaging “Village Doctors” in the Community‐based Integrated Management of Childhood Illness (C‐IMCI): Experience from rural Bangladesh. *Journal of Global Health, 8*(2), 020413. <https://doi.org/10.7189/jogh.08.020413>

Chimhutu, V. (2011). *Pay for performance in maternal health in Tanzania: Perceptions, expectations and experiences in Mvomero district* [PhD Thesis, University of Bergen]. <https://bora.uib.no/bora-xmlui/bitstream/handle/1956/5311/84857036.pdf?sequence=1&isAllowed=y>

Chimhutu, V., Songstad, N. G., Tjomsland, M., Mrisho, M., & Moland, K. M. (2016). The inescapable question of fairness in pay‐for‐performance bonus distribution: A qualitative study of health workers' experiences in Tanzania. *Globalization and Health, 12*(1), 77. <https://doi.org/10.1186/s12992-016-0213-5>

Chimhutu, V., Tjomsland, M., & Mrisho, M. (2019). Experiences of care in the context of payment for performance (P4P) in Tanzania. *Globalization and Health, 15*(1), 59. <https://doi.org/10.1186/s12992-019-0503-9>

Chimhutu, V., Tjomsland, M., Songstad, N. G., Mrisho, M., & Moland, K. M. (2015). Introducing payment for performance in the health sector of Tanzania‐ the policy process. *Globalization and Health, 11*(1), 38. <https://doi.org/10.1186/s12992-015-0125-9>

Doctor, H. V., Findley, S. E., Ager, A., Cometto, G., Afenyadu, G. Y., Adamu, F., & Green, C. (2012). Using community‐based research to shape the design and delivery of maternal health services in Northern Nigeria. *Reproductive Health Matters, 20*(39), 104–112. <https://doi.org/10.1016/s0968-8080(12)39615-8>

Febriany, V., Toyamah, N., Sodo, J., Budiyati, S. (2011). *Qualitative impact study for PNPM generasi and PKH on the provision and the utilization of maternal and child health services and basic education services in the provinces of West Java and East Nusa Tenggara*. Jakarta: SMERU Research Institute. <http://www.smeru.or.id/sites/default/files/publication/pnpmgenerasi_eng.pdf>

Grayman, J. H., Ruhanawati, S., & Anggraini, N. (2014). *Opportunities and approaches for better nutrition outcomes through PNPM generasi: A qualitative study*. Washington, D.C.: World Bank Group. <http://documents.worldbank.org/curated/en/537241468266368818/Opportunities-and-approaches-for-better-nutrition-outcomes-through-PNPM-generasi-a-qualitative-study>

Henning, M. J., Zulu, J. M., Michelo, C., Simmons Zuilkowski, S., & Hubner, C. (2020). Adolescent mothers' experiences with community health assistants in rural Zambia. *International Quarterly of Community Health Education, 40*(4), 353–361. <https://doi.org/10.1177/0272684x19896737>

Khuzwayo, L. S., & Moshabela, M. (2017). The perceived role of ward‐based primary healthcare outreach teams in rural KwaZulu‐Natal, South Africa. *African Journal of Primary Health Care & Family Medicine, 9*(1), e1–e5. <https://doi.org/10.4102/phcfm.v9i1.1388>

Lignou, S., Das, S., Mistry, J., Alcock, G., More, N. S., Osrin, D., & Edwards, S. J. L. (2016). Reconstructing communities in cluster trials? *Trials, 17*(1), 166. <https://doi.org/10.1186/s13063-016-1284-6>

Marcus, T. S., Hugo, J., & Jinabhai, C. C. (2017). Which primary care model? A qualitative analysis of ward‐based outreach teams in South Africa. *African Journal Of Primary Health Care & Family Medicine, 9*(1), e1–e8. <https://doi.org/10.4102/phcfm.v9i1.1252>

Ministry of Public Health and Sanitation. (2012). *National Communication strategy for community health services*. Ministry of Public Health and Sanitation. <https://www.slideshare.net/chskenya/chs-kenya-national-communicationstrategyforcommunityhealthservices20122017>

Moosa, S., Derese, A., & Peersman, W. (2017). Insights of health district managers on the implementation of primary health care outreach teams in Johannesburg, South Africa: A descriptive study with focus group discussions. *Human Resources for Health, 15*(1), 7. <https://doi.org/10.1186/s12960-017-0183-6>

Okeke, E. N., Pitchforth, E., Exley, J., Glick, P., Abubakar, I. S., Chari, A. V., Bashir, U., Gu, K., & Onwujekwe, O. (2017). Going to scale: Design and implementation challenges of a program to increase access to skilled birth attendants in Nigeria. *BMC Health Services Research, 17*(1), 356. <https://doi.org/10.1186/s12913-017-2284-2>

Oladepo, O., Dipeolu, I. O., & Oladunni, O. (2019). Nigerian rural mothers' knowledge of routine childhood immunizations and attitudes about use of reminder text messages for promoting timely completion. *Journal of Public Health Policy, 40*(4), 459–477. <https://doi.org/10.1057/s41271-019-00180-7>

Oladepo, O., Dipeolu, I. O., & Oladunni, O. (2020). Outcome of reminder text messages intervention on completion of routine immunization in rural areas, Nigeria. *Health Promotion International, 36*, 765–773. <https://doi.org/10.1093/heapro/daaa092>

Olafsdottir, A. E., Mayumana, I., Mashasi, I., Njau, I., Mamdani, M., Patouillard, E., Binyaruka, P., Abdulla, S., & Borghi, J. (2014). Pay for performance: An analysis of the context of implementation in a pilot project in Tanzania. *BMC Health Services Research, 14*(1), 392. <https://doi.org/10.1186/1472-6963-14-392>

Pérez, M. C., Chandra, D., Koné, G., Singh, R., Ridde, V., Sylvestre, M.‐P., Seth, A., & Johri, M. (2020). Implementation fidelity and acceptability of an intervention to improve vaccination uptake and child health in rural India: A mixed methods evaluation of a pilot cluster randomized controlled trial. *Implementation Science Communications, 1*(1), 88. <https://doi.org/10.1186/s43058-020-00077-7>

Phiri, S. C., Prust, M. L., Chibawe, C. P., Misapa, R., van den Broek, J. W., & Wilmink, N. (2017). An exploration of facilitators and challenges in the scale‐up of a national, public sector community health worker cadre in Zambia: A qualitative study. *Human Resources for Health, 15*(1), 40. <https://doi.org/10.1186/s12960-017-0214-3>

Rahayu, S. K., Toyamah, N., Hutagalung, S. A., Rosfadhila, M., & Syukri, M. (2008). *Qualitative baseline study for PNPM Generasi and PKH: The availability and use of the maternal and child health services and basic education services in the provinces of West Java and East Nusa Tenggara (Development Economics Working Papers 22536)*. East Asian Bureau of Economic Research. <https://www.academia.edu/73623787/Qualitative_Baseline_Study_for_PNPM_Generasi_and_PKH_The_Availability_and_Use_of_the>

Rahman, A. (2007). Challenges and opportunities in developing a psychological intervention for perinatal depression in rural Pakistan—A multi‐method study. *Archives of Women's Mental Health, 10*(5), 211–219. <https://doi.org/10.1007/s00737-007-0193-9>

Rahman, A., Leppard, M., Rashid, S., Jahan, N., & Nasreen, H. E. (2016). Community perceptions of behaviour change communication interventions of the maternal neonatal and child health programme in rural Bangladesh: An exploratory study*. BMC Health Services Research, 16*(1), 389. <https://doi.org/10.1186/s12913-016-1632-y>

Robertson, L., Mushati, P., Skovdal, M., Eaton, J. W., Makoni, J. C., Crea, T., Mavise, G., Dumba, L., Schumacher, C., Sherr, L., Nyamukapa, C., & Gregson, S. (2014). Involving communities in the targeting of cash transfer programs for vulnerable children: Opportunities and challenges. *World Development, 54*, 325–337. <https://doi.org/10.1016/j.worlddev.2013.09.002>

Sacks, A., Grayman, J.H., Afriko, H., Anggraini, N., Endarso, G.K.A., Prahara, H., Prabowo, A., Rozana, L., Subandoro, A., Wrobel, R., & Friel, K.R. (2018). *Indonesia - Long-term generasi qualitative study (English)*. Washington, D.C.: World Bank Group. <http://documents.worldbank.org/curated/en/296651529900604983/Indonesia-Long-term-Generasi-qualitative-study>

Shah, P., Madhiwala, N., Shah, S., Desai, G., Dave, K., Dholakia, N., Patel, S., Desai, S., & Modi, D. (2019). High uptake of an innovative mobile phone application among community health workers in rural India: An implementation study. *The National Medical Journal of India, 32*(5), 262–269. <https://doi.org/10.4103/0970-258x.295956>

Shelley, K. D., Belete, Y. W., Phiri, S. C., Musonda, M., Kawesha, E. C., Muleya, E. M., Chibawe, C. P., van den Broek, J. W., & Vosburg, K. B. (2016). Implementation of the Community Health Assistant (CHA) cadre in Zambia: A process evaluation to guide future scale‐up decisions. *Journal of Community Health, 41*(2), 398–408. <https://doi.org/10.1007/s10900-015-0110-5>

Skovdal, M., Mushati, P., Robertson, L., Munyati, S., Sherr, L., Nyamukapa, C., & Gregson, S. (2013). Social acceptability and perceived impact of a community‐led cash transfer programme in Zimbabwe. *BMC Public Health, 13*(1), 342. <https://doi.org/10.1186/1471-2458-13-342>

Skovdal, M., Robertson, L., Mushati, P., Dumba, L., Sherr, L., Nyamukapa, C., & Gregson, S. (2013). Acceptability of conditions in a community‐led cash transfer programme for orphaned and vulnerable children in Zimbabwe. *Health Policy and Planning, 29*(7), 809–817. <https://doi.org/10.1093/heapol/czt060>

Zulu, J. M., Kinsman, J., Michelo, C., & Hurtig, A. K. (2013). Developing the national community health assistant strategy in Zambia: A policy analysis. *Health Research Policy and Systems, 11*, 24. <https://doi.org/10.1186/1478-4505-11-24>

Zulu, J. M., Kinsman, J., Michelo, C., & Hurtig, A.‐K. (2014). Hope and despair: Community health assistants' experiences of working in a rural district in Zambia. *Human Resources for Health, 12*(1), 30. <https://doi.org/10.1186/1478-4491-12-30>

### Included other studies

Adamu, A. A. (2019). *Using quality improvement approach to address missed opportunities for vaccination in Kano Metropolis, Nigeria* [PhD, Faculty of Medicine and Health Sciences, Stellenbosch University]. <https://scholar.sun.ac.za/handle/10019.1/107226>

Adamu, A. A., Uthman, O. A., Gadanya, M. A., Adetokunboh, O. O., & Wiysonge, C. S. (2019). A multilevel analysis of the determinants of missed opportunities for vaccination among children attending primary healthcare facilities in Kano, Nigeria: Findings from the pre‐implementation phase of a collaborative quality improvement programme. *PLoS One, 14*(7), e0218572. https://doi.org/10.1371/journal.pone.0218572

Alhassan, R. K. (2017). *Healthcare quality in Ghana: Improving healthcare quality and health worker motivation to promote sustainable health insurance* [PhD, Faculty of Medicine (AMC‐UvA), University of Amsterdam]. <https://dare.uva.nl/search?identifier=b294c75f-d7e3-4c5a-ae03-da5011bb01e0>

Banwat, M. E., Bupwatd, P. W., Lar, L. A., Apagu, A. A., & Zoakah, A. I. (2014). Effect of peer education on timeliness and completeness of routine immunization: An assessment in rural communities in North‐Central Nigeria. *International Journal of Community Research, 3*(3), 60–67. <https://www.ajol.info/index.php/ijcr/article/view/107649>

Bekele, A., Kefale, M., & Tadesse, M. (2016). Preliminary assessment of the implementation of the health services extension program: The case of Southern Ethiopia. *The Ethiopian Journal of Health Development, 22*(3), 302-05. <https://www.ejhd.org/index.php/ejhd/article/view/514>

Binyaruka, P., & Anselmi, L. (2020). Understanding efficiency and the effect of pay‐for‐performance across health facilities in Tanzania. *BMJ Global Health, 5*(5), e002326. <https://doi.org/10.1136/bmjgh-2020-002326>

Binyaruka, P., & Borghi, J. (2017). Improving quality of care through payment for performance: examining effects on the availability and stock‐out of essential medical commodities in Tanzania. *Tropical Medicine & International Health, 22*(1), 92–102. <https://doi.org/10.1111/tmi.12809>

Binyaruka, P., Patouillard, E., Powell‐Jackson, T., Greco, G., Maestad, O., & Borghi, J. (2015). Effect of paying for performance on utilisation, quality, and user costs of health services in Tanzania: A controlled before and after study. *PLoS One, 10*(8), e0135013. <https://doi.org/10.1371/journal.pone.0135013>

Binyaruka, P., Robberstad, B., Torsvik, G., & Borghi, J. (2018a). Does payment for performance increase performance inequalities across health providers? A case study of Tanzania. *Health Policy Plan, 33*(9), 1026–1036. <https://doi.org/10.1093/heapol/czy084>

Binyaruka, P., Robberstad, B., Torsvik, G., & Borghi, J. (2018b). Who benefits from increased service utilisation? examining the distributional effects of payment for performance in Tanzania. *International Journal for Equity in Health, 17*(1), 14. <https://doi.org/10.1186/s12939-018-0728-x>

Borghi, J., Little, R., Binyaruka, P., Patouillard, E., & Kuwawenaruwa, A. (2015). In Tanzania, the many costs of pay‐for‐performance leave open to debate whether the strategy is cost‐effective. *Health Affairs (Millwood), 34*(3), 406–414. <https://doi.org/10.1377/hlthaff.2014.0608>

Borkum, E., Sivasankaran, A., Sridharan, S., Rotz, D., Sethi, S., Manoranjini, M., Ramakrishnan, L., & Rangarajan, A. (2015). *Evaluation of the Information and Communication Technology (ICT) Continuum of Care Services (CCS) Intervention in Bihar.* Mathematica Policy Research. <https://www.mathematica.org/publications/evaluation-of-the-information-and-communication-technology-ict-continuum-of-care-services-ccs>

Carmichael, S. L., Mehta, K., Raheel, H., Srikantiah, S., Chaudhuri, I., Trehan, S., Mohanty, S., Borkum, E., Mahapatra, T., Weng, Y., Kaimal, R., Sivasankaran, A., Sridharan, S., Rotz, D., Tarigopula, U. K., Bhattacharya, D., Atmavilas, Y., Munar, W., Rangarajan, A., & Darmstadt, G. L. (2019). Effects of team‐based goals and non‐monetary incentives on front‐line health worker performance and maternal health behaviours: A cluster randomised controlled trial in Bihar, India. *BMJ Global Health, 4*(4), e001146. <https://doi.org/10.1136/bmjgh-2018-001146>

Cockcroft, A., Andersson, N., Omer, K., Ansari, N. M., Khan, A., Chaudhry, U. U., & Ansari, U. (2009). One size does not fit all: Local determinants of measles vaccination in four districts of Pakistan. *BMC International Health and Human Rights, 9*(Suppl. 1), S4. <https://doi.org/10.1186/1472-698x-9-s1-s4>

Costa‐i‐Font, J., & Parmar, D. (2017). *Political agency and public health care: Evidence from India* (CESifo Working Paper Series). United Nationa University—UNU‐WIDER. **This reference to be deleted, it is already cited in included studies**

Crea, T. M., Reynolds, A. D., Sinha, A., Eaton, J. W., Robertson, L. A., Mushati, P., Dumba, L., Mavise, G., Makoni, J. C., Schumacher, C. M., Nyamukapa, C. A., & Gregson, S. (2015). Effects of cash transfers on children's health and social protection in Sub‐Saharan Africa: Differences in outcomes based on orphan status and household assets. *BMC Public Health, 15*(1), 511. <https://doi.org/10.1186/s12889-015-1857-4>

Doctor, H. V., & Dahiru, T. (2010). Utilization of non‐skilled birth attendants in northern Nigeria: A rough terrain to the health‐related MDGs. *African Journal of Reproductive Health, 14*(2), 37–45.

Doctor, H. V., Findley, S. E., Bairagi, R., & Dahiru, T. (2011). Northern Nigeria maternal, newborn and child health programme: Selected analyses from population‐based baseline survey. *The Open Demography Journal, 4*, 11–21. <https://doi.org/10.2174/1874918601104010011>

Doctor, H. V., Findley, S. E., Cometto, G., & Afenyadu, G. Y. (2013). Awareness of critical danger signs of pregnancy and delivery, preparations for delivery, and utilization of skilled birth attendants in Nigeria. *Journal of Health Care for the Poor and Underserved, 24*(1), 152–170. <https://doi.org/10.1353/hpu.2013.0032>

Domek, G. J., Contreras‐Roldan, I. L., Asturias, E. J., Bronsert, M., Bolaños Ventura, G. A., O'Leary, S. T., Kempe, A., & Bull, S. (2018). Characteristics of mobile phone access and usage in rural and urban Guatemala: Assessing feasibility of text message reminders to increase childhood immunizations. *Mhealth, 4*, 9. <https://doi.org/10.21037/mhealth.2018.03.05>

Domek, G. J., Contreras‐Roldan, I. L., O'Leary, S. T., Bull, S., Furniss, A., Kempe, A., & Asturias, E. J. (2016). SMS text message reminders to improve infant vaccination coverage in Guatemala: A pilot randomized controlled trial. *Vaccine, 34*(21), 2437–2443. <https://doi.org/10.1016/j.vaccine.2016.03.065>

Domek, G. J., O'Leary, S. T., Bull, S., Bronsert, M., Contreras‐Roldan, I. L., Bolaños Ventura, G. A., Kempe, A., & Asturias, E. J. (2018). Measuring vaccine hesitancy: field testing the WHO SAGE Working Group on Vaccine Hesitancy survey tool in Guatemala. *Vaccine, 36*(35), 5273–5281. <https://doi.org/10.1016/j.vaccine.2018.07.046>

Findley, S. E., Uwemedimo, O. T., Doctor, H. V., Green, C., Adamu, F., & Afenyadu, G. Y. (2013). Comparison of high‐ versus low‐intensity community health worker intervention to promote newborn and child health in Northern Nigeria*. International Journal of Womens Health, 5*, 717–728. <https://doi.org/10.2147/ijwh.s49785>

Gibson, D. G., Ochieng, B., Kagucia, E. W., Obor, D., Odhiambo, F., O'Brien, K. L., & Feikin, D. R. (2015). Individual level determinants for not receiving immunization, receiving immunization with delay, and being severely underimmunized among rural western Kenyan children. *Vaccine, 33*(48), 6778–6785. <https://doi.org/10.1016/j.vaccine.2015.10.021>

Grant, C., Nawal, D., Guntur, S. M., Kumar, M., Chaudhuri, I., Galavotti, C., Mahapatra, T., Ranjan, K., Kumar, G., Mohanty, S., Alam, M. A., Das, A., & Jiwani, S. (2018). ‘We pledge to improve the health of our entire community’: Improving health worker motivation and performance in Bihar, India through teamwork, recognition, and non‐financial incentives. *PLoS One, 13*(8), e0203265. <https://doi.org/10.1371/journal.pone.0203265>

Mirkuzie, W., Morankar, S. N., Feyissa, G. T., Labonte, R., & Sanders, D. (2015). Coverage of child health services in rural districts of Ethiopia with the health services extension program. *Journal of Public Health and Epidemiology, 7*(7), 223–231. <https://doi.org/10.5897/JPHE2015.0733>

Mitchell, S., Andersson, N., Ansari, N. M., Omer, K., Soberanis, J. L., & Cockcroft, A. (2009). Equity and vaccine uptake: A cross‐sectional study of measles vaccination in Lasbela District, Pakistan. *BMC International Health and Human Rights, 9*(Suppl 1), S7. <https://doi.org/10.1186/1472-698X-9-S1-S7>

Modi, D., Gopalan, R., Shah, S., Venkatraman, S., Desai, G., Desai, S., & Shah, P. (2015). Development and formative evaluation of an innovative mhealth intervention for improving coverage of community‐based maternal, newborn and child health services in rural areas of India. *Global Health Action, 8*, 26769. <https://doi.org/10.3402/gha.v8.26769>

Modi, D., Patel, J., Desai, S., & Shah, P. (2016). Accessing completeness of pregnancy, delivery, and death registration by Accredited Social Health Activists [ASHA] in an innovative mHealth project in the tribal areas of Gujarat: A cross‐sectional study. *Journal of Postgraduate Medicine, 62*(3), 170–172. <https://doi.org/10.4103/0022-3859.183168>

Modi, D., Saha, S., Vaghela, P., Dave, K., Anand, A., Desai, S., & Shah, P. (2020). Costing and Cost‐Effectiveness of a Mobile Health Intervention (ImTeCHO) in improving infant mortality in tribal areas of Gujarat, India: Cluster randomized controlled trial. *JMIR Mhealth Uhealth, 8*(10), e17066. <https://doi.org/10.2196/17066>

Moore, C. (2008). *Assessing Honduras' CCT Programme PRAF, Programa de Asignacion familiar: Expected and unexpected realities*. Brazil: International Poverty Centre. United Nations Development Programme. <https://ipcig.org/sites/default/files/pub/en/IPCCountryStudy15.pdf>

Nagar, R. B. (2016). *Tying community engagement with appropriate technology at the last mile: A cluster randomized trial to determine the effectiveness of a novel, digital pendant and voice reminder platform on increasing infant immunization adherence among mothers in rural Udaipur, India*. [Public Health Thesis 1203, School of Public Health, Yale University] <https://elischolar.library.yale.edu/ysphtdl/1203>

Negusse, H., McAuliffe, E., & MacLachlan, M. (2007). Initial community perspectives on the Health Service Extension Programme in Welkait, Ethiopia. *Human Resources for Health, 5*, 21. <https://doi.org/10.1186/1478-4491-5-21>

Oduenyi, C., Ordu, V., & Okoli, U. (2019). Assessing the operational effectiveness of a maternal and child health (MCH) conditional cash transfer pilot programme in Nigeria. *BMC Pregnancy and Childbirth, 19*(1), 298. <https://doi.org/10.1186/s12884-019-2418-0>

Okeke, E., Glick, P., Chari, A., Abubakar, I. S., Pitchforth, E., Exley, J., Bashir, U., Gu, K., & Onwujekwe, O. (2016). The effect of increasing the supply of skilled health providers on pregnancy and birth outcomes: Evidence from the midwives service scheme in Nigeria. *BMC Health Services Research, 16*(1), 425. <https://doi.org/10.1186/s12913-016-1688-8>

Onwujekwe, O., Ensor, T., Ogbozor, P., Okeke, C., Ezenwaka, U., Hicks, J. P., Etiaba, E., Uzochukwu, B., Ebenso, B., & Mirzoev, T. (2020). Was the maternal health cash transfer programme in Nigeria sustainable and cost‐effective? *Frontiers in Public Health, 8*, 582072. <https://doi.org/10.3389/fpubh.2020.582072>

Padayachee, T., Chetty, N., Matse, M., Mampe, T., & Schneider, H. (2013). *Progress in the establishment of ward based outreach teams: Experiences in the North West Province*. University of Western Cape. <http://hdl.handle.net/10566/1472>

Quayyum, Z., Khan, M. N., Quayyum, T., Nasreen, H. E., Chowdhury, M., & Ensor, T. (2013). “Can community level interventions have an impact on equity and utilization of maternal health care”—Evidence from rural Bangladesh. *International Journal for Equity in Health, 12*, 22. https://doi.org/10.1186/1475-9276-12-22

Rahman, M., Jhohura, F. T., Mistry, S. K., Chowdhury, T. R., Ishaque, T., Shah, R., & Afsana, K. (2015). Assessing community based Improved Maternal Neonatal Child Survival (IMNCS) program in rural Bangladesh. *PLoS One, 10*(9), e0136898. <https://doi.org/10.1371/journal.pone.0136898>

Rangarajan, A., Borkum, E., Sridharan, S., Rotz, D., Manoranjini, M., Morgan, S., Dandona, L., Dandona, R., Chaman, P., & Anil Kumar, G. (2013). *Baseline findings from the ananya evaluation*. Mathematica Policy Research. <https://www.mathematica.org/publications/baseline-findings-from-the-ananya-evaluation>

Robertson, L., Mushati, P., Eaton, J. W., Sherr, L., Makoni, J. C., Skovdal, M., Crea, T., Mavise, G., Dumba, L., Schumacher, C., Munyati, S., Nyamukapa, C., & Gregson, S. (2012). Household‐based cash transfer targeting strategies in Zimbabwe: Are we reaching the most vulnerable children? *Social Science & Medicine (1982)*, *75*(12), 2503–2508. <https://doi.org/10.1016/j.socscimed.2012.09.031>

Robinson, V. C. (2006). *Summary report The rural maintenance program: A cash for work program (Report 5)*. CARE Bangladesh. <https://www.carebangladesh.org/publication/Publication_5538986.pdf>

UNICEF. (2010). *Evaluation report of the community health strategy implementation in Kenya*. Division of Community Health Services. <http://guidelines.health.go.ke:8000/media/Evaluation_Report_of_the_Community_Health_Strategy_Implementation_in_Kenya.pdf>

USAID. (2008b). *Essential services for health in Ethiopia: Final report November 2003–September 2008*. **This is already cited in included list, to be deleted from here.**

Venkat, P. (2016). *Intermediate assessment of the Khushi Baby Crct: Implementation of a novel mhealth solution for vaccination record keeping in rural Udaipur, Rajasthan, India* [Public Health Thesis, School of Public Health, Yale University]. <https://elischolar.library.yale.edu/cgi/viewcontent.cgi?article=1299&context=ysphtdl>

Wakadha, H., Chandir, S., Were, E. V., Rubin, A., Obor, D., Levine, O. S., Gibson, D. G., Odhiambo, F., Laserson, K. F., & Feikin, D. R. (2013). The feasibility of using mobile‐phone based SMS reminders and conditional cash transfers to improve timely immunization in rural Kenya. *Vaccine, 31*(6), 987–993. <https://doi.org/10.1016/j.vaccine.2012.11.093>

### References to ongoing studies

de Hoop, T. (2015). *Impact Evaluation of BRAC's Nutrition and Early Childhood Development Programs*. Registry for International Development for Impact Evaluations (RIDIE), RIDIE‐STUDY‐ID‐563a7d17974f3. Retrieved April 20, 2021, from <https://ridie.3ieimpact.org/index.php?r=search/detailView&id=373>

ISRCTN registry. (2018). *Cash for Improved Nutrition in Somalia*. ISRCTN registry, ISRCTN24757827. BMC. Retrieved April 20, 2021, from <https://www.isrctn.com/ISRCTN24757827>

ISRCTN registry. (2019). *Improving immunization coverage in urban slums in Indonesia*. ISRCTN registry, ISRCTN14961777. BMC. Retrieved April 20, 2021, from <https://www.isrctn.com/ISRCTN14961777>

ISRCTN registry. (2019). *The INSPIRING Project—Building capacity to reduce child deaths in Jigawa State, Nigeria*. ISRCTN registry, ISRCTN39213655. BMC. Retrieved April 20, 2021, from <https://www.isrctn.com/ISRCTN39213655>

The World Bank. (2020). *Nigeria—Immunization plus and malaria progress by accelerating coverage and transforming services project (English)* (Report No: PAD3025). World Bank Group. <http://documents.worldbank.org/curated/en/102621580321213128/Nigeria-Immunization-Plus-and-Malaria-Progress-by-Accelerating-Coverage-and-Transforming-Services-Project>

### References to excluded studies

Aaby, P., Roth, A., Ravn, H., Napirna, B. M., Rodrigues, A., Lisse, I. M., Stensballe, L., Diness, B. R., Lausch, K. R., Lund, N., Biering‐Sørensen, S., Whittle, H., & Benn, C. S. (2011). Randomized trial of BCG vaccination at birth to low‐birth‐weight children: Beneficial nonspecific effects in the neonatal period? *The Journal of Infectious Diseases, 204*(2), 245–252. <https://doi.org/10.1093/infdis/jir240>

Aggarwal, S. (2018). *The long road to health: Healthcare utilization impacts of a road pavement policy in rural India* (Working Paper). Indian School of Business. <http://eprints.exchange.isb.edu/884/>

Ahmed, T., Arur, A., Walque, D. de, & Shapira, G. (2019). *Incentivizing quantity and quality of care: Evidence from an impact evaluation of performance‐based financing in the health sector in Tajikistan* (Policy Research Working Papers 8951). The World Bank. <https://doi.org/10.1596/1813-9450-8951>

Alatas, V., Chandrasekhar, A., Mobius, M., Olken, B., & Paladines, C. (2019). *When celebrities speak: A nationwide Twitter experiment promoting vaccination in Indonesia* (NBER Working Paper Series 25589). National Bureau of Economic Research. <https://doi.org/10.3386/w25589>

Amaral, J., Gouws, E., Bryce, J., Leite, Á. J. M., Alves da Cunha, A. L., & Victora, C. G. (2004). Effect of integrated management of childhood illness (imci) on health worker performance in northeast‐Brazil. *Cadernos de Saúde Pública, 20*(Suppl. 2), S209–S219. <https://doi.org/10.1590/s0102-311x2004000800016>

Andrade, M. V., Chein, F., de Souza, L. R., & Puig‐Junoy, J. (2012). Income transfer policies and the impacts on the immunization of children: the bolsa família program. *Cadernos de Saúde Pública, 28*(7), 1347–1358. <https://doi.org/10.1590/s0102-311x2012000700013>

Andreoni, J., Callen, M., Khan, M. Y., Jaffar, K., & Sprenger, C. (2016). *Using preference estimates to customize incentives: An application to polio vaccination drives in Pakistan* (NBER Working Paper Series 22019). National Bureau of Economic Research. <https://doi.org/10.3386/w22019>

Anjum, Q., Omair, A., Bazmi Inam, S. N., Ahmed, Y., Usman, Y., & Shaikh, S. (2004). Improving vaccination status of children under five through health education. *JPMA. The Journal of the Pakistan Medical Association, 54*(12), 610–613. <https://jpma.org.pk/article-details/515?article_id=515>

Aquino, R., de Oliveira, N. F., & Barreto, M. L. (2009). Impact of the family health program on infant mortality in Brazilian municipalities. *American Journal of Public Health*, *99*(1), 87–93. <https://doi.org/10.2105/AJPH.2007.127480>

Arraiz, I., & Rozo, S. (2011). *Same bureaucracy, different outcomes in human capital? How indigenous and rural non‐indigenous areas in Panama responded to the CCT* (OVE Working Papers 03/11). Inter‐American Development Bank. <http://www.ssrn.com/abstract=1847124>

Atnafu, A., Otto, K., & Herbst, C. H. (2017). The role of MHealth intervention on maternal and child health service delivery: Findings from a randomized controlled field trial in rural Ethiopia. *MHealth*, *3*(September), 39. <https://doi.org/10.21037/mhealth.2017.08.04>

Attanasio, O., Gómez, L. C., Heredia, P., & Vera‐Hernández, M. (2005). The short‐term impact of a conditional cash subsidy on child health and nutrition in Colombia. 15. (Report Summary: Familias 03) Centre for the Evaluation of Development Policies, The Institute for Fiscal Studies. <https://ifs.org.uk/edepo/rs_fam03.pdf>

Ayieko, P., Ntoburi, S., Wagai, J., Opondo, C., Opiyo, N., Migiro, S., Wamae, A., Mogoa, W., Were, F., Wasunna, A., Fegan, G., Irimu, G., & English, M. (2011). A multifaceted intervention to implement guidelines and improve admission paediatric care in Kenyan district hospitals: A cluster randomised trial. *PLoS Medicine, 8*(4), 1001018. <https://doi.org/10.1371/journal.pmed.1001018>

Balasubramaniam, S., Kumar, S., Sethi, R., Charurat, E., Lalchandani, K., Schuster, A., & Sood, B. (2018). Quasi‐experimental study of systematic screening for family planning services among postpartum women attending village health and nutrition days in Jharkhand, India. *International Journal of Integrated Care, 18*(1), 7. <https://doi.org/10.5334/ijic.3078>

Banerjee, R., & Sachdeva, A. (2015). *Pathways to preventive health, evidence from India's Rural Road Program* (USC‐INET Research Paper 15–19). USC Dornsife Institute for New Economic Thinking. <http://www.ssrn.com/abstract=2636999>

Bangure, D., Chirundu, D., Gombe, N., Marufu, T., Mandozana, G., Tshimanga, M., & Takundwa, L. (2015). Effectiveness of short message services reminder on childhood immunization programme in kadoma, Zimbabwe—A randomized controlled trial, 2013. *BMC Public Health, 15*(1), 137. <https://doi.org/10.1186/s12889-015-1470-6>

Barham, T. (2005). *The impact of the Mexican conditional cash transfer program on immunization rates*. University of Colorado. <https://ibs.colorado.edu/barham/wp/CCTimmunWBfin.pdf>

Barham, T., Brenzel, L. E., & Maluccio, J. A. (2007). “*Beyond 80%: Are there new ways of increasing vaccination coverage? Evaluation of CCT programs in Mexico and Nicaragua*” (HNP Discussion Paper 41537). World Bank. <http://www.ssrn.com/abstract=993760>

Barham, T., & Maluccio, J. A. (2009). Eradicating diseases: the effect of conditional cash transfers on vaccination coverage in rural Nicaragua. *Journal of Health Economics, 28*(3), 611–621. https://doi.org/10.1016/j.jhealeco.2008.12.010

Basinga, P., Gertler, P. J., Binagwaho, A., Soucat, A. L. B., Sturdy, J. R., & Vermeersch, C. M. J. (2009). *Impact of performance based financing in rwanda: Health facility level analysis* (Working Paper 32). Global Development Network. <http://www.gdn.int/impact-performance-based-financing-rwanda-health-facility-level-analysis>

Basinga, P., Gertler, P. J., Binagwaho, A., Soucat, A. L. B., Sturdy, J., & Vermeersch, C. M. J. (2011). Effect on maternal and child health services in Rwanda of payment to primary health‐care providers for performance: An impact evaluation. *The Lancet, 377*(9775), 1421–1428. <https://doi.org/10.1016/S0140-6736(11)60177-3>

Beck, S., Pulkki‐Brännström, A.‐M., & Sebastián, M. S. (2015). Basic income—Healthy outcome? Effects on health of an Indian basic income pilot project: A cluster randomised trial. *Journal of Development Effectiveness, 7*(1), 111–126. <https://doi.org/10.1080/19439342.2014.974200>

Berhane, Y., & Pickering, J. (1993). Are reminder stickers effective in reducing immunization dropout rates in Addis Ababa, Ethiopia? *Journal of Tropical Medicine and Hygiene, 96*(3), 139–145. <https://europepmc.org/article/med/8505766>

Bernal, P., & Martinez, S. (2020). In‐kind incentives and health worker performance: Experimental evidence from El Salvador, *Journal of Health Economics 70*(March), 102267. <https://doi.org/10.1016/j.jhealeco.2019.102267>

Binyaruka, P., Patouillard, E., Powell‐Jackson, T., Greco, G., Maestad, O., & Borghi, J. (2015). Effect of paying for performance on utilisation, quality, and user costs of health services in Tanzania: A controlled before and after study. *PLoS One*, *10*(8), 0135013. <https://doi.org/10.1371/journal.pone.0135013>

Bonfrer, I., Breebaart, L., & Van de Poel, E. (2016). The effects of Ghana's National Health Insurance Scheme on maternal and infant health care utilization. *PLoS One*, *11*(11), 0165623. <https://doi.org/10.1371/journal.pone.0165623>

Bonfrer, I., Van de Poel, E., & Van Doorslaer, E. (2014). The effects of performance incentives on the utilization and quality of maternal and child care in Burundi. *Social Science & Medicine*, *123*(December), 96–104. <https://doi.org/10.1016/j.socscimed.2014.11.004>

Bossio, J. C., Sanchis, I., Armando, G. A., Arias, S. J., & Jure, H. (2019). Resultado de una estrategia de recordatorios previos y posteriores a la fecha de vacunación para mejorar la oportunidad de la vacunación a los seis meses. *Cadernos de Saúde Pública*, *35*(12), 00214518. <https://doi.org/10.1590/0102-311x00214518>.

Bradley, J., & Igras, S. (2005). Improving the quality of child health services: Participatory action by providers. *International Journal for Quality in Health Care*, *17*(5), 391–399. <https://doi.org/10.1093/intqhc/mzi057>

Brenner, S., Chase, R. P., McMahon, S. A., Lohmann, J., Makwero, C. J., Muula, A. S., & De Allegri, M. (2020). Effect heterogeneity in responding to performance‐based incentives: A quasi‐experimental comparison of impacts on health service indicators between hospitals and health centers in Malawi. *Health Systems & Reform*, *6*(1), 1745580. <https://doi.org/10.1080/23288604.2020.1745580>

Briere, E. C., Ryman, T. K., Cartwright, E., Russo, E. T., Wannemuehler, K. A., Nygren, B. L., Kola, S., Sadumah, I., Ochieng, C., Watkins, M. L., & Quick, R. (2012). Impact of integration of hygiene kit distribution with routine immunizations on infant vaccine coverage and water treatment and handwashing practices of Kenyan mothers. *Journal of Infectious Diseases, 205*(suppl 1), S56–S64. <https://doi.org/10.1093/infdis/jir779>

Brown, V. B., Oluwatosin, O. A., Akinyemi, J. O., & Adeyemo, A. A. (2016). Effects of community health nurse‐led intervention on childhood routine immunization completion in primary health care centers in Ibadan, Nigeria. *Journal of Community Health, 41*(2), 265–273. <https://doi.org/10.1007/s10900-015-0092-3>

Brown, V. B., Oluwatosin, O. A., & Ogundeji, M. O. (2017). Impact of training intervention on immunization providers' knowledge and practice of routine immunization in Ibadan, South‐Western Nigeria: A primary health care experience. *Pan African Medical Journal, 26,* 216. <https://doi.org/10.11604/pamj.2017.26.216.11545>

Brughal, R. F., & Kevany, J. P. (1996). Maximizing immunization coverage through home visits: A controlled trial in an urban area of Ghana. *Bulletin of the World Health Organization, 74*(5), 517–524. <https://www.ncbi.nlm.nih.gov/pmc/articles/PMC2486871/>

Busso, M., Cristia, J., & Humpage, S. (2015). Did you get your shots? Experimental evidence on the role of reminders. *Journal of Health Economics, 44*(December), 226–237. <https://doi.org/10.1016/j.jhealeco.2015.08.005>

Cahyadi, N., Hanna, R., Olken, B. A., Adi Prima, R., Satriawan, E., Syamsulhakim, E., & Hanna, R. (2018). *Cumulative impacts of conditional cash transfer programs: Experimental evidence from Indonesia* (NBER Working Paper Series 24670). National Bureau of Economic Research. <https://www.nber.org/papers/w24670>

Carrillo, B., Iglesias, W. J., & Trujillo, J. C. (2015). Attainments and limitations of an early childhood programme in Colombia. *Health Policy and Planning*, *30*(7), 906–916. <https://doi.org/10.1093/heapol/czu091>

Carvalho, N., Thacker, N., Gupta, S. S., & Salomon, J. A. (2014). More evidence on the impact of India's Conditional Cash Transfer Program, Janani Suraksha Yojana: Quasi‐experimental evaluation of the effects on childhood immunization and other reproductive and child health outcomes. *PLoS One, 9*(10), e109311. <https://doi.org/10.1371/journal.pone.0109311>

Chakrabarti, A., Grépin, K. A., & Helleringer, S. (2019). The impact of supplementary immunization activities on routine vaccination coverage: An instrumental variable analysis in five low‐income countries. *PLoS One*, *14*(2), e0212049. <https://doi.org/10.1371/journal.pone.0212049>

Chandir, S., Khan, A. J., Hussain, H., Usman, H. R., Khowaja, S., Halsey, N. A., & Omer, S. B. (2010). Effect of food coupon incentives on timely completion of DTP immunization series in children from a low‐income area in Karachi, Pakistan: A longitudinal intervention study. *Vaccine, 28*(19). 3473–3478. <https://doi.org/10.1016/j.vaccine.2010.02.061>

Chansa, C., Das, A., Qamruddin, J., Friedman, J., Mkandawire, A., & Vledder, M. (2015). *Linking results to performance: Evidence from a results based financing pre‐pilot project in Katete District, Zambia* (Discussion Paper 98265). The World Bank. <https://openknowledge.worldbank.org/handle/10986/22390?show=full>

Chelagat, T., Kokwaro, G., Onyango, J., & Rice, J. (2020). Effect of project‐based experiential learning on the health service delivery indicators: A quasi‐experiment study. *BMC Health Services Research, 20*(1), 144. <https://doi.org/10.1186/s12913-020-4949-5>

Chen, L., Du, X., Zhang, L., van Velthoven, M. H., Wu, Q., Yang, R., Cao, Y., Wang, W., Xie, L., Rao, X., Zhang, Y., & Koepsell, J. C. (2016). Effectiveness of a smartphone app on improving immunization of children in rural Sichuan Province, China: A cluster randomized controlled trial. *BMC Public Health, 16*(1), 909. <https://doi.org/10.1186/s12889-016-3549-0>

Chen, Y. J., Chindarkar, N., & Xiao, Y. (2019). Effect of reliable electricity on health facilities, health information, and child and maternal health services utilization: Evidence from rural Gujarat, India. *Journal of Health, Population and Nutrition, 38*(1), 7. <https://doi.org/10.1186/s41043-019-0164-6>

Cristia, J., Evans, W. N., & Kim, B. (2015). Improving the health coverage of the rural poor: Does contracting‐out mobile medical teams work? *The Journal of Development Studies*, *51*(3), 247-261. <https://doi.org/10.1080/00220388.2014.976617>

Cristia, J. P., Evans, W. N., & Kim, B. (2011). *Does contracting‐out primary care services work? The case of rural Guatemala* (IDB Working Paper Series IDB‐WP‐273). <https://www.econstor.eu/handle/10419/88975>

Cristia, J., Prado, A. G., & Peluffo, C. (2015). The impact of contracting in and contracting out basic health services: The Guatemalan experience. World Development, 70(June), 215–227. <https://doi.org/10.1016/j.worlddev.2015.02.003>

Cruzado de la Vega, V. (2017). Pagos por desempeño para mejorar estado nutricional infantil: impacto de los convenios de apoyo presupuestario en tres regiones peruanas con alta prevalencia de desnutrición cronica infantil, 2010–2014. *Revista Peruana de Medicina Experimental y Salud Pública, 34*(3), 365–376. <http://dx.doi.org/10.17843/rpmesp.2017.343.2987>

Daoud, A., & Reinsberg, B. (2019). Structural adjustment, state capacity and child health: Evidence from IMF programmes. *International Journal of Epidemiology*, *48*(2), 445–454. <https://doi.org/10.1093/ije/dyy251>

Dicko, A., Toure, S. O., Traore, M., Sagara, I., Toure, O. B., Sissoko, M. S., Diallo, A. T., Rogier, C., Salomon, R., de Sousa, A., & Doumbo, O. K. (2011). Increase in EPI vaccines coverage after implementation of intermittent preventive treatment of malaria in infant with sulfadoxine ‐pyrimethamine in the district of Kolokani, Mali: Results from a cluster randomized control trial. *BMC Public Health, 11*(1), 573. <https://doi.org/10.1186/1471-2458-11-573>

Dissieka, R., Soohoo, M., Janmohamed, A., & Doledec, D. (2019). Providing mothers with mobile phone message reminders increases childhood immunisation and vitamin A supplementation coverage in Côte d'Ivoire: A randomised controlled trial. *Journal of Public Health in Africa, 10*(1), 56–60. <https://doi.org/10.4081/jphia.2019.1032>

Djibuti, M., Gotsadze, G., Zoidze, A., Mataradze, G., Esmail, L. C., & Kohler, J. C. (2009). The role of supportive supervision on immunization program outcome—A randomized field trial from Georgia. *BMC International Health and Human Rights, 9*(S1), S11. <https://doi.org/10.1186/1472-698X-9-S1-S11>

Domek, G. J., Contreras‐Roldan, I. L., Bull, S., O'Leary, S. T., Ventura, G. A. B., Bronsert, M., Kempe, A., & Asturias, E. J. (2019). Text message reminders to improve infant immunization in Guatemala: A randomized clinical trial. *Vaccine*, *37*(42), 6192–6200. This is cited in included list of studies. TO be deleted from here.

Drain, P. K., Ralaivao, J. S., Rakotonandrasana, A., & Carnell, M. A. (2003). Introducing auto‐disable syringes to the national immunization programme in Madagascar. Bulletin of the World Health Organization, 8, 553-60. <https://www.ncbi.nlm.nih.gov/pmc/articles/PMC2572525/>

D'Souza, P. V., & Umarani, J. (2014). Teaching package improves mothers knowledge on vaccine preventable diseases and vaccination: A quasi experimental study. *International Journal of Research in Medical Sciences*, *2*(3), 976–982.<https://www.msjonline.org/index.php/ijrms/article/view/2336>

Dykstra, S., Glassman, A., Kenny, C., & Sandefur, J. (2019). Regression discontinuity analysis of Gavi's impact on vaccination rates. *Journal of Development Economics*, *140*(September), 12–25. <https://doi.org/10.1016/j.jdeveco.2019.04.005>

Edoka, I., Ensor, T., McPake, B., Amara, R., Tseng, F.‐M., & Edem‐Hotah, J. (2016). Free health care for under‐fives, expectant and recent mothers? Evaluating the impact of Sierra Leone's free health care initiative. *Health Economics Review*, *6*(1), 19. <https://doi.org/10.1186/s13561-016-0096-4>

Eichler, R., Auxila, P., Antoine, U., & Desmangles, B. (2007). *Performance‐based incentives for health: Six years of results from supply‐side programs in Haiti* (CGD Working Paper 121). Center for Global Development. <http://www.ssrn.com/abstract=1003249>

Ekhaguere, O. A., Oluwafemi, R. O., Badejoko, B., Oyeneyin, L. O., Butali, A., Lowenthal, E. D., & Steenhoff, A. P. (2019). Automated phone call and text reminders for childhood immunisations (PRIMM): A randomised controlled trial in Nigeria. *BMJ Global Health, 4*(2), 001232. <http://dx.doi.org/10.1136/bmjgh-2018-001232>

Eze, G. U., & Adeleye, A. O. (2015). Enhancing routine immunization performance using innovative technology in an urban area of Nigeria. *West African Journal of Medicine*, *34*(1), 3–10. <https://files.givewell.org/files/DWDA%202009/Interventions/SMS_reminders_for_vaccination/Eze_and_Adeleye_2015.pdf>

Gajate‐Garrido, G., & Ahiadeke, C. (2012). The effect of parents' insurance enrollment on health care utilization: Evidence from Ghana (SSRN Scholarly Paper ID 2158824). Social Science Research Network. <https://doi.org/10.2139/ssrn.2158824>

Garly, M. L., Martins, C. L., Bale, C., da Costa, F., Dias, F., Whittle, H., & Aaby, P. (1999). Early two‐dose measles vaccination schedule in Guinea‐Bissau: Good protection and coverage in infancy. *International Journal of Epidemiology, 28*(2), 347–352. <https://doi.org/10.1093/ije/28.2.347>

Gauri, V. (2009). *Do international treaties promote development? The convention on the rights of the child and basic immunization* (Policy research working paper 4964). The World Bank. <https://doi.org/10.1596/1813-9450-4964>

Gauri, V., & Khaleghian, P. (2002). *Immunization in developing countries: Its political and organizational determinants* (Policy Research Working Paper 2769). The World Bank. <https://www.infona.pl/resource/bwmeta1.element.elsevier-4a3b393c-c1da-3dff-9b7c-91c05767fb9d>

Gilbert, S. S., Bulula, N., Yohana, E., Thompson, J., Beylerian, E., Werner, L., & Shearer, J. C. (2020). The impact of an integrated Electronic Immunization Registry and Logistics Management Information System (EIR‐ELMIS) on vaccine availability in three regions in Tanzania: A pre‐post and time‐series analysis. *Vaccine, 38*(3), 562–569.<https://doi.org/10.1016/j.vaccine.2019.10.059>

Goodson, J. L., Kulkarni, M. A., Vanden Eng, J. L., Wannemuehler, K. A., Cotte, A. H., Desrochers, R. E., Randriamanalina, B., & Luman, E. T. (2012). Improved equity in measles vaccination from integrating insecticide‐treated bednets in a vaccination campaign, Madagascar: Equity in measles vaccination in Madagascar. *Tropical Medicine & International Health, 17*(4), 430–437. <https://doi.org/10.1111/j.1365-3156.2011.02953.x>

Guindon, G. E. (2014). The impact of health insurance on health services utilization and health outcomes in Vietnam. *Health Economics, Policy and Law, 9*(4), 359–382. <https://doi.org/10.1017/S174413311400005X>

Gultiano, S. A., & King, E. M. (2006). A better start in life: Evaluation results from an early childhood development program. *Philippine Journal of Development, 33*(1/2), 101–128. <https://ideas.repec.org/p/phd/pjdevt/pjd_2006_vol__xxxiii_nos__1and2-d.html>

Habib, M. A., Soofi, S., Cousens, S., Anwar, S., ul Haque, N., Ahmed, I., Ali, N., Tahir, R., & Bhutta, Z. A. (2017). Community engagement and integrated health and polio immunisation campaigns in conflict‐affected areas of Pakistan: A cluster randomised controlled trial. *The Lancet Global Health, 5*(6), e593–e603. <https://doi.org/10.1016/S2214-109X(17)30184-5>

Hagiwara, A., Ueyama, M., Ramlawi, A., & Sawada, Y. (2013). Is the maternal and child health (MCH) handbook effective in improving health‐related behavior? Evidence from Palestine. *Journal of Public Health Policy, 34*(1), 31–45. <https://doi.org/10.1057/jphp.2012.56>

Haji, A. H. (2017). *Evaluation of SMS and sticker reminders in reducing dropout rates in routine child immunization in selected districts in Kenya* [Master Thesis, Juja: Jomo Kenyatta University of Agriculture and Technology]. <http://ir.jkuat.ac.ke/handle/123456789/3069>

Hajizadeh, M., Heymann, J., Strumpf, E., Harper, S., & Nandi, A. (2015). Paid maternity leave and childhood vaccination uptake: Longitudinal evidence from 20 low‐and‐middle‐income countries. *Social Science & Medicine, 140*(September), 104–117. <https://doi.org/10.1016/j.socscimed.2015.07.008>

Hategeka, C., Ruton, H., & Law, M. R. (2019). Effect of a community health worker MHealth monitoring system on uptake of maternal and newborn health services in Rwanda. *Global Health Research and Policy, 4*(1), 8. <https://doi.org/10.1186/s41256-019-0098-y>

Heinrich, C. J., & Knowles, M. T. (2020). A fine predicament: Conditioning, compliance and consequences in a labeled cash transfer program. *World Development 129*(May), 104876. <https://doi.org/10.1016/j.worlddev.2020.104876>

Helleringer, S., Asuming, P. O., & Abdelwahab, J. (2016). The effect of mass vaccination campaigns against polio on the utilization of routine immunization services: A regression discontinuity design. *Vaccine, 34*(33), 3817–3822. <https://doi.org/10.1016/j.vaccine.2016.05.037>

Hu, Y., Chen, Y., Wang, Y., Song, Q., & Li, Q. (2017). Prenatal vaccination education intervention improves both the mothers' knowledge and children's vaccination coverage: Evidence from randomized controlled trial from eastern China. *Human Vaccines & Immunotherapeutics, 13*(6), 1477–1484. <https://doi.org/10.1080/21645515.2017.1285476>

Huillery, E., & Seban, J. (2019). Financial incentives, efforts, and performances in the health sector: experimental evidence from the Democratic Republic of Congo. *Economic Development and Cultural Change, 69*(3), 1115–1164. <https://doi.org/10.1086/703235>

Hutchinson, P., Lance, P., Guilkey, D. K., Shahjahan, M., & Haque, S. (2006). Measuring the cost‐effectiveness of a National Health Communication Program in rural Bangladesh. *Journal of Health Communication, 11*(Suppl. 2), 91–121. <https://doi.org/10.1080/10810730600974647>

Ikilezi, G., Augusto, O. J., Dieleman, J. L., Sherr, K., & Lim, S. S. (2020). Effect of donor funding for immunization from GAVI and other development assistance channels on vaccine coverage: Evidence from 120 low and middle income recipient countries. *Vaccine, 38*(3), 588–596. <https://doi.org/10.1016/j.vaccine.2019.10.057>

Ikilezi, G., Augusto, O. J., Sbarra, A., Sherr, K., Dieleman, J. L., & Lim, S. S. (2020). Determinants of geographical inequalities for DTP3 vaccine coverage in Sub‐Saharan Africa. *Vaccine, 38*(18), 3447–3454. <https://doi.org/10.1016/j.vaccine.2020.03.005>

Jaupart, P., Dipple, L., & Dercon, S. (2019). Has Gavi lived up to its promise? Quasi‐experimental evidence on country immunisation rates and child mortality. *BMJ Global Health, 4*(6), 001789. <https://doi.org/10.1136/bmjgh-2019-001789>

Kagucia, E. W. (2018). *MHealth interventions to improve measles vaccination coverage and timeliness: An assessment of the immediate and long‐term impact on vaccine‐seeking in rural Kenya* [PhD Thesis, Johns Hopkins University]. <https://jscholarship.library.jhu.edu/handle/1774.2/61130>

Kamatsuchi, M., Gheorghe, A., & Balabanova, D. (2019). The global scale and implications of delivering multiple interventions through integrated child health events. BMJ Global Health, 4(4), 001333. <https://doi.org/10.1136/bmjgh-2018-001333>

Kandpal, E., Alderman, H., Friedman, J., Filmer, D., Onishi, J., & Avalos, J. (2016). A conditional cash transfer program in the Philippines reduces severe stunting. *The Journal of Nutrition, 146*(9), 1793–1800. <https://doi.org/10.3945/jn.116.233684>

Karing, A. (2018). *Social signaling and childhood immunization: A field experiment in Sierra Leone* (Berkeley Working Paper). University of California. <https://economics.yale.edu/sites/default/files/jmp_socialsignaling.pdf>

Kawakatsu, Y., Sugishita, T., Oruenjo, K., Wakhule, S., Kibosia, K., Were, E., & Honda, S. (2015). Effectiveness of and factors related to possession of a mother and child health handbook: An analysis using propensity score matching. *Health Education Research, 30*(October), 935–946. <https://doi.org/10.1093/her/cyv048>

Kazi, A. M., Ali, M., Zubair, K., Kalimuddin, H., Kazi, A. N., Iqbal, S. P., Collet, J.‐P., & Ali, S. A. (2018). Effect of mobile phone text message reminders on routine immunization uptake in Pakistan: Randomized controlled trial. *JMIR Public Health and Surveillance, 4*(1), 20. <https://doi.org/10.2196/publichealth.7026>

Kern, A. P., de Toledo Vieira, M., & da Silva Freguglia, R. (2018). *Impactos Do Programa Bolsa Família Na Imunização Das Crianças* (working paper). <https://www.anpec.org.br/encontro/2018/submissao/files_I/i12-cb6dfc0f070c8ca9e77abc8563c3801f.pdf>

Khaleghian, P. (2003). *Decentralization and public services: The case of immunization* (Policy Research Working Paper 2989). The World Bank. <https://openknowledge.worldbank.org/handle/10986/19159?show=full>

Kusuma, D., Thabrany, H., Hidayat, B., McConnell, M., Berman, P., & Cohen, J. (2017). New evidence on the impact of large‐scale conditional cash transfers on child vaccination rates: The case of a clustered‐randomized trial in Indonesia. *World Development, 98*(October), 497–505. <https://doi.org/10.1016/j.worlddev.2017.05.007>

Lamanna, C., & Byrne, L. (2019). A pilot study of a novel, incentivised MHealth technology to monitor the vaccine supply chain in rural Zambia. *Pan African Medical Journal, 33*, 50. <https://doi.org/10.11604/pamj.2019.33.50.16318>

Levy, D., & Ohls, J. (2010). Evaluation of Jamaica's PATH conditional cash transfer programme. *Journal of Development Effectiveness, 2*(4), 421–441. <https://doi.org/10.11604/pamj.2019.33.50.16318>

Lin, M. A. (2016). *Stimulating demand: An assessment of the conditional cash transfer project in Afghanistan* [PhD Thesis, Johns Hopkins University]. <https://jscholarship.library.jhu.edu/handle/1774.2/39740>

Liu, Y., Yuan, Z., Liu, Y., Jayasinghe, U. W., & Harris, M. F. (2014). Changing community health service delivery in economically less‐developed rural areas in China: Impact on service use and satisfaction*. BMJ Open, 4*(2), 004148. <https://doi.org/10.1136/bmjopen-2013-004148>

Loevinsohn, B. P., & Gareaballah, E. (1992). Missed opportunities for immunization during visits for curative care: A randomized cross‐over trial in Sudan. *Bulletin of the World Health Organization, 70*(3), 335–339.

Loevinsohn, B. P., & Loevinsohn, M. E. (1986). Improvement in coverage of primary health care in a developing country through use of food incentives. *The Lancet, 327*(8493), 1314–1316. <https://doi.org/10.1016/s0140-6736(86)91231-6>

Looij, F. vande, Mureyi, D., Sisimayi, C., Koot, J., Manangazira, P., & Musuka, N. (2015). Early evidence from results‐based financing in rural Zimbabwe. *African Health Monitor, 6*, 32–36.

Maluccio, J. A., & Flores, R. (2005). *Impact evaluation of a conditional cash transfer program the Nicaraguan Red de Protección Social* (Research Report 141). International Food Policy Research Institute. <https://doi.org/10.2499/0896291464RR141>

Mampe, T., Schneider, H., & Reagon, G. (2016). *Effectiveness of ward based outreach teams in the North West Province: An evaluation*. University of the Western Cape. <https://doi.org/10.13140/RG.2.2.36191.66725>

Masuda, K., & Yamauchi, C. (2020). How does female education reduce adolescent pregnancy and improve child health? Evidence from Uganda's universal primary education for fully treated cohorts. *The Journal of Development Studies, 56*(1), 63–86. <https://doi.org/10.1080/00220388.2018.1546844>

Mathanga, D. P., Luman, E. T., Campbell, C. H., Silwimba, C., & Malenga, G. (2009). Integration of insecticide‐treated net distribution into routine immunization services in Malawi: A pilot study. *Tropical Medicine & International Health, 14*(7), 792–801. <https://doi.org/10.1111/j.1365-3156.2009.02295.x>

Mazumder, S., Taneja, S., Bahl, R., Mohan, P., Strand, T. A., Sommerfelt, H., Kirkwood, B. R., Goyal, N., Hombergh, H. V. F., Martines, J., & Bhandari, N. (2014). Effect of implementation of integrated management of neonatal and childhood illness programme on treatment seeking practices for morbidities in infants: Cluster randomised trial. *BMJ, 349*(August), g4988. <https://doi.org/10.1136/bmj.g4988>

McMahon, S. A., Brenner, S., Lohmann, J., Makwero, C., Torbica, A., Mathanga, D. P., Muula, A. S., & De Allegri, M. (2016). Evaluating complex health financing interventions: Using mixed methods to inform further implementation of a novel PBI intervention in rural Malawi. *BMC Health Services Research, 16*(1), 414. <https://doi.org/10.1186/s12913-016-1612-2>

Mehta, K. M., Rerolle, F., Rammohan, S. V., Albohm, D. C., Muwowo, G., Moseson, H., Sept, L., Lee, H. L., & Bendavid, E. (2016). Systematic motorcycle management and health care delivery: A field trial*. American Journal of Public Health, 106*(1), 87–94. <https://doi.org/10.2105/AJPH.2015.302891>

Mensah, J., Oppong, J. R., & Schmidt, C. M. (2009). *Ghana's National Health Insurance Scheme in the context of the health MDGs: An empirical evaluation using propensity score matching* (Ruhr Economic Papers 157). <https://papers.ssrn.com/sol3/papers.cfm?abstract_id=1532169>

Mohan, P., Kishore, B., Singh, S., Bahl, R., Puri, A., & Kumar, R. (2012). Assessment of implementation of integrated management of neonatal and childhood illness in India. *Journal of Health, Population and Nutrition, 29*(6), 629–638. <https://doi.org/10.3329/jhpn.v29i6.9900>

Musa, O. I., Parakoyi, D. B., & Akanbi, A. A. (2006). Evaluation of health education intervention on safe immunization injection among health workers in Ilorin, Nigeria. *Annals of African Medicine, 5*(3), 122–128.

Nanyunja, M., Lewis, R. F., Makumbi, I., Seruyange, R., Kabwongera, E., Mugyenyi, P., & Talisuna, A. (2003). Impact of mass measles campaigns among children less than 5 years old in Uganda. *The Journal of Infectious Diseases, 187*(s1), S63–S68. <https://doi.org/10.1086/368026>

Nasir, N. M., Amran, Y., & Nakamura, Y. (2017). Changing knowledge and practices of mothers on newborn care through mother class: An intervention study in Indonesia. *Journal of Tropical Pediatrics, 63*(6), 440–446. <https://doi.org/10.1093/tropej/fmx010>

Onishi, J. (2014). *Philippines conditional cash transfer program impact evaluation 2012* (World Bank Report 75533‐PH). The World Bank. <https://assessments.hpc.tools/sites/default/files/assessments/Philippines%20Conditional%20Cash%20Transfer%20Program%2C%20Impact%20Evaluation%202012.pdf>

Owais, A., Hanif, B., Siddiqui, A. R., Agha, A., & Zaidi, A. K. M. (2011). Does improving maternal knowledge of vaccines impact infant immunization rates? A community‐based randomized‐controlled trial in Karachi, Pakistan. *BMC Public Health, 11*(1), 239. <https://doi.org/10.1186/1471-2458-11-239>

Özer, M., Fidrmuc, J., & Eryurt, M. A. (2018). Maternal education and childhood immunization in Turkey. *Health Economics, 27*(8), 1218–1229. <https://doi.org/10.1002/hec.3770>

Padilla, A. deJ., & Trujillo, J. C. (2015). An impact assessment of the child growth, development and care program in the Caribbean Region of Colombia. *Cadernos de Saúde Pública, 31*(10), 2099–2109. <https://doi.org/10.1590/0102-311X00153514>

Pandey, P., Sehgal, A. R., Riboud, M., Levine, D., & Goyal, M. (2007). Informing resource‐poor populations and the delivery of entitled health and social services in rural India: A cluster randomized controlled trial. *JAMA, 298*(16), 1867–1875. <https://doi.org/10.1001/jama.298.16.1867>

Pathak, Y., & Macours, K. (2017). Women's political reservation, early childhood development, and learning in India. *Economic Development and Cultural Change, 65*(4), 741–766.

Powell‐Jackson, T., Fabbri, C., Dutt, V., Tougher, S., & Singh, K. (2018). Effect and cost‐effectiveness of educating mothers about childhood DPT vaccination on immunisation uptake, knowledge, and perceptions in Uttar Pradesh, India: A randomised controlled trial. *PLoS Medicine, 15*(3), 1002519. <https://doi.org/10.1371/journal.pmed.1002519>

Pramanik, S., Ghosh, A., Nanda, R. B., de Rouw, M., Forth, P., & Albert, S. (2018). Impact evaluation of a community engagement intervention in improving childhood immunization coverage: A cluster randomized controlled trial in Assam, India. *BMC Public Health, 18*(1), 534. <https://doi.org/10.1186/s12889-018-5458-x>

Prinja, S., Nimesh, R., Gupta, A., Bahuguna, P., Gupta, M., & Thakur, J. S. (2017). Impact of M‐Health application used by community health volunteers on improving utilisation of maternal, new‐born and child health care services in a rural area of Uttar Pradesh, India. *Tropical Medicine & International Health, 22*(7), 895–907. <https://doi.org/10.1111/tmi.12895>

Prosser, W., Jaillard, P., Assy, E., Brown, S. T., Matsinhe, G., Dekoun, M., & Lee, B. Y. (2017). System redesign of the immunization supply chain: Experiences from Benin and Mozambique. *Vaccine, 35*(17), 2162–2166. <https://doi.org/10.1016/j.vaccine.2016.09.073>

Rahman, M. M., & Pallikadavath, S. (2019, April). Maternal and child health care services' utilization data from the fourth round of district level household survey in India. *Data Brief, 23*(March), 103738. <https://doi.org/10.1016/j.dib.2019.103738>

Rajkotia, Y., Zang, O., Nguimkeu, P., Gergen, J., Djurovic, I., Vaz, P., Mbofana, F., & Jobarteh, K. (2017). The effect of a performance‐based financing program on HIV and maternal/child health services in Mozambique—An impact evaluation. *Health Policy and Planning, 32*(10), 1386–1396. <https://doi.org/10.1093/heapol/czx106>

Rasella, D., Aquino, R., Santos, C. A. T., Paes‐Sousa, R., & Barreto, M. L. (2013). Effect of a conditional cash transfer programme on childhood mortality: A nationwide analysis of Brazilian municipalities. *The Lancet, 382*(9886), 57–64. <https://doi.org/10.1016/S0140-6736(13)60715-1>

Reinbold, G. W. (2019). Effects of the convention on the rights of the child on child mortality and vaccination rates: A synthetic control analysis. *BMC International Health and Human Rights, 19*(1), 24. <https://doi.org/10.1186/s12914-019-0211-9>

Robinson, J. S., Burkhalter, B. R., Rasmussen, B., & Sugiono, R. (2001). Low‐cost on‐the‐job peer training of nurses improved immunization coverage in Indonesia. *Bulletin of the World Health Organization, 79*(2), 150–158. <https://apps.who.int/iris/handle/10665/268259>

Roth, A. E., Stabell Benn, C., Ravn, H., Rodrigues, A., Lisse, I. M., Yazdanbakhsh, M., Whittle, H., & Aaby, P. (2010). Effect of revaccination with BCG in early childhood on mortality: randomised trial in Guinea‐Bissau. *BMJ, 340*(1), c671. <https://doi.org/10.1136/bmj.c671>

Rusa, L., Ngirabega, J. deD., Janssen, W., Van Bastelaere, S., Porignon, D., & Vandenbulcke, W. (2009). Performance‐based financing for better quality of services in Rwandan Health Centres: 3‐year experience. *Tropical Medicine & International Health, 14*(7), 830–837. <https://doi.org/10.1111/j.1365-3156.2009.02292.x>

Ryman, T. K., Briere, E. C., Cartwright, E., Schlanger, K., Wannemuehler, K. A., Russo, E. T., Kola, S., Sadumah, I., Nygren, B. L., Ochieng, C., Quick, R., & Watkins, M. L. (2012). Integration of routine vaccination and hygiene interventions: A comparison of 2 strategies in Kenya. The *Journal of Infectious Diseases, 205*(Suppl_1), S65–S76. <https://doi.org/10.1093/infdis/jir777>

Ryman, T. K., Trakroo, A., Wallace, A., Kumar Gupta, S., Wilkins, K., Mehta, P., & Dietz, V. (2011). Implementation and evaluation of the Reaching Every District (RED) strategy in Assam, India, 2005–2008. *Vaccine, 29*(14), 2555–2560. <https://doi.org/10.1016/j.vaccine.2011.01.061>

Salami, L., Ouendo, E.‐M. D., & Fayomi, B. (2016). Effects of results based financing models on data quality improvement in Benin on 2014. *Universal Journal of Public Health, 4*(6), 324–331. <https://doi.org/10.13189/ujph.2016.040605>

Salami, L., Ouendo, E.‐M., & Fayomi, B. (2018). Effects of results based financing models on the performance of exposed health zones in Benin. *International Journal Of Community Medicine And Public Health, 5*(10), 4188–4199. <http://dx.doi.org/10.18203/2394-6040.ijcmph20183953>

Sangwan, S., & Manocha, A. (2009). Maternal knowledge and child health. *Journal of Human Ecology, 25*(1), 51–54. <https://doi.org/10.1080/09709274.2009.11906135>

Sato, R., & Belel, A. (2020). The effect of performance‐based financing on child vaccinations in Northern Nigeria. *Vaccine, 38*(9), 2209–2215. <https://doi.org/10.1016/j.vaccine.2020.01.033>

Schlumberger, M., Bamoko, A., Yaméogo, T. M., Rouvet, F., Ouedraogo, R., Traoré, B., Tinto, M., Bakyono, J. F., Sombie, I., Bazié, B. B., Ganama, S., Savadogo, Y., & Yelkoumi, G. A. (2015). Impact positif sur le Programme élargi de vaccinations de l'envoi de SMS de rappel à partir d'un registre informatisé, Bobo‐Dioulasso (Burkina Faso). *Bulletin de la Société de pathologie exotique, 108*(5), 349–354. <https://doi.org/10.1007/s13149-015-0455-4>

Sengupta, N., & Sinha, A. (2018). Is India's safe motherhood scheme leading to better child health care practices? *Global Social Welfare, 5*(1), 49–58. <https://doi.org/10.1007/s40609-017-0103-7>

Shei, A., Costa, F., Reis, M. G., & Ko, A. I. (2014). The impact of Brazil's Bolsa Família conditional cash transfer program on children's health care utilization and health outcomes. *BMC International Health and Human Rights, 14*(1), 10. <https://doi.org/10.1186/1472-698X-14-10>

Sherry, T. B., Bauhoff, S., & Mohanan, M. (2017). Multitasking and heterogeneous treatment effects in pay‐for‐performance in health care: Evidence from Rwanda. *American Journal of Health Economics, 3*(2), 192–226.

Shuaib, W., Suarez, J. M., Romero, J. D., Pamello, C. D., Alweis, R., Khan, A. A., Shah, S. R., Shahid, H., PierreCharles, S. B., & Sanchez, L. R. (2016). Transforming patient care by introducing an electronic medical records initiative in a developing country. *Health Informatics Journal, 22*(4), 975–983. <https://doi.org/10.1177/1460458215589204>

Sinha, N., & Yoong, J. K. (2009). *Long‐term financial incentives and investment in daughters: Evidence from conditional cash transfers in North India* (RAND Labor and Population Working Paper Series WR‐667). RAND Corporation. <https://doi.org/10.1596/1813-9450-4860>

Soeters, R., Peerenboom, P. B., Mushagalusa, P., & Kimanuka, C. (2011). Performance‐based financing experiment improved health care in the democratic Republic of Congo. *Health Affairs, 30*(8), 1518–1527. <https://doi.org/10.1377/hlthaff.2009.0019>

Steinhardt, L. C., Aman, I., Pakzad, I., Kumar, B., Singh, L. P., & Peters, D. H. (2011). Removing user fees for basic health services: A pilot study and national roll‐out in Afghanistan. *Health Policy and Planning, 26*(Suppl 2), ii92–103. <https://doi.org/10.1093/heapol/czr069>

Subedi, S., Sharma, B. P., & Adhikari, C. (2018). Cost and consequences of integrated public health campaign in Baglung District, Nepal. *Economic Journal of Development Issues, 23 & 24*(January), 113–122. <https://doi.org/10.3126/ejdi.v23i1-2.19068>

Talukder, M., Rob, U., Musa, S. A., Bajracharya, A., Keya, K., Noor, F., Jahan, E., Hossain, M., Saha, J., & Bellows, B. (2014). *Evaluation of the impact of the voucher program for improving maternal health behavior and status in Bangladesh*. Dhaka: Population Council. <https://doi.org/10.31899/rh10.1000>

Tanner, J., Hayashi, R., & Li, Y. (2015). I*mproving coverage and utilization of maternal and child health services in Lao PDR: Impact evaluation of the community nutrition project* (IEG Working Paper 99929). The World Bank Group. <https://documents.worldbank.org/en/publication/documents-reports/documentdetail/441841468187767237/improving-coverage-and-utilization-of-maternal-and-child-health-services-in-lao-pdr-impact-evaluation-of-the-community-nutrition-project>

Thomas, R. A. (2011). *Essays on ex ante evaluations of cash transfer programs* [PhD Thesis, York: University of York]. <http://etheses.whiterose.ac.uk/2004/>

Thomson, D. R., Amoroso, C., Atwood, S., Bonds, M. H., Rwabukwisi, F. C., Drobac, P., Finnegan, K. E., Farmer, D. B., Farmer, P. E., Habinshuti, A., Hirschhorn, L. R., Manzi, A., Niyigena, P., Rich, M. L., Stulac, S., Murray, M. B., & Binagwaho, A. (2018). Impact of a health system strengthening intervention on maternal and child health outputs and outcomes in rural Rwanda 2005–2010. *BMJ Global Health, 3*(2), 000674. <https://doi.org/10.1136/bmjgh-2017-000674>

Thysen, S. M., Byberg, S., Pedersen, M., Rodrigues, A., Ravn, H., Martins, C., Benn, C. S., Aaby, P., & Fisker, A. B. (2014). BCG coverage and barriers to BCG vaccination in Guinea‐Bissau: An observational study. *BMC Public Health, 14*(1), 1037. <https://doi.org/10.1186/1471-2458-14-1037>

Uddin, M. J., Shamsuzzaman, M., Horng, L., Labrique, A., Vasudevan, L., Zeller, K., Chowdhury, M., Larson, C. P., Bishai, D., & Alam, N. (2016). Use of mobile phones for improving vaccination coverage among children living in rural hard‐to‐reach areas and urban streets of Bangladesh. *Vaccine, 34*(2), 276–283. <https://doi.org/10.1016/j.vaccine.2015.11.024>

Unger, J.‐P. (1991). Can intensive campaigns dynamize front line health services? the evaluation of an immunization campaign in Thiès Health District, Senegal. *Social Science & Medicine, 32*(3), 249–259. <https://doi.org/10.1016/0277-9536(91)90101-h>

Usman, H. R., Akhtar, S., Habib, F., & Jehan, I. (2009). Redesigned immunization card and center‐based education to reduce childhood immunization dropouts in urban Pakistan: A randomized controlled trial. *Vaccine, 27*(3), 467–472. <https://doi.org/10.1016/j.vaccine.2008.10.048>

Usman, H. R., Rahbar, M. H., Kristensen, S., Vermund, S. H., Kirby, R. S., Habib, F., & Chamot, E. (2011). Randomized controlled trial to improve childhood immunization adherence in rural Pakistan: Redesigned immunization card and maternal education. *Tropical Medicine & International Health, 16*(3), 334–342. <https://doi.org/10.1111/j.1365-3156.2010.02698.x>

Vaidyanathan, R. (2019). Immunization coverage among under‐five children living along a school student through child‐to‐child and child‐to‐parent information, education and communication strategy. *Indian Journal of Public Health, 63*(4), 334–340. <https://doi.org/10.4103/ijph.IJPH_424_18>

Van de Poel, E., Flores, G., Ir, P., & O'Donnell, O. (2016). Impact of performance‐based financing in a low‐resource setting: A decade of experience in Cambodia. *Health Economics, 25*(6), 688–705. <https://doi.org/10.1002/hec.3219>

Varghese, B., Roy, R., Saha, S., & Roalkvam, S. (2014). Fostering maternal and newborn care in India the Yashoda way: Does this improve maternal and newborn care practices during institutional delivery? *PLoS One, 9*(1), 84145. <https://doi.org/10.1371/journal.pone.0084145>

Verma, M., Chhatwal, J., & Varughese, P. V. (1995). Antenatal period: An educational opportunity. *Indian Pediatrics, 32*, 171–177.

Wadhwa, S. (2019). *Conditional cash transfers and parental investment in daughters: Evidence from India*. Paper presented at the Population Association of America 2019 Annual Meeting, Austin, TX. <http://paa2019.populationassociation.org/abstracts/193315>

Wagstaff, A., & Yu, S. (2007). Do health sector reforms have their intended impacts? The world bank's health VIII project in Gansu Province, China. *Journal of Health Economics, 26*, 505–535. <https://doi.org/10.1016/j.jhealeco.2006.10.006>

Wallace, A. S., Peetosutan, K., Untung, A., Ricardo, M., Yosephine, P., Wannemuehler, K., Brown, D. W., McFarland, D. A., Orenstein, W. A., Rosenberg, E. S., Omer, S. B., & Daniels, D. (2019). Home‐based records and vaccination appointment stickers as parental reminders to reduce vaccination dropout in Indonesia: A cluster‐randomized controlled trial. *Vaccine, 37*(45), 6814–6823. <https://doi.org/10.1016/j.vaccine.2019.09.040>

Walque, D. de, Robyn, P. J., Saidou, H., Sorgho, G., & Steenland, M. (2017). *Looking into the performance‐based financing black box: Evidence from an impact evaluation in the health sector in Cameroon* (Policy Research Working Paper 8162). The World Bank. <https://doi.org/10.1596/1813-9450-8162>

Wang, P. C., Mwango, A., Moberley, S., Brockman, B. J., Connor, A. L., Kalesha‐Masumbu, P., Mutembo, S., Bweupe, M., Chanda‐Kapata, P., Biemba, G., Hamer, D. H., Chibuye, B., & McCarthy, E. (2015). A cluster randomised trial on the impact of integrating early infant HIV diagnosis with the expanded programme on immunization on immunization and HIV testing rates in rural health facilities in Southern Zambia. *PLoS One, 10*(10):e0141455. <https://doi.org/10.1371/journal.pone.0141455>

Weldemariam, M. (2010). *The impact of fiscal decentralization on education and health outcomes in Ethiopia: A regional panel data analysis* [Master Thesis, Addis Ababa University]. <http://etd.aau.edu.et/handle/123456789/14217?show=full>

Wong, B. K. C., Fadel, S. A., Awasthi, S., Khera, A., Kumar, R., Menon, G., & Jha, P. (2019). The impact of measles immunization campaigns in India using a nationally representative sample of 27,000 child deaths. *Elife, 8*(March), e43290. <https://doi.org/10.7554/eLife.43290>

Yoong, J. (2007). *Does decentralization hurt childhood immunization?* Stanford University. <http://citeseerx.ist.psu.edu/viewdoc/summary?doi=10.1.1.370.4021>

Yoosuf, A.‐S. (1993). Group learning by mothers about primary health care. *World Health Forum, 14*(1), 20–22.

Zang, O., Djienouassi, S., Sorgho, G., & Taptue, J. C. (2015). Impact of performance‐based financing on health‐care quality and utilization in urban areas of Cameroon. *African Health Monitor, 7*(21), 10–14.

Zeng, W., Shepard, D. S., de Dieu Rusatira, J., Blaakman, A. P., & Nsitou, B. M. (2018). Evaluation of results‐based financing in the republic of the Congo: A comparison group pre–post study. *Health Policy and Planning, 33*(3), 392–400. <https://doi.org/10.1093/heapol/czx195>

Zeng, W., Sun, D., Mphwanthe, H., Huan, T., Nam, J. E., Saint‐Firmin, P., Manthalu, G., Sharma, S., & Dutta, A. (2019). The impact and cost‐effectiveness of user fee exemption by contracting out essential health package services in Malawi. *BMJ Global Health, 4*(2):e001286. <http://dx.doi.org/10.1136/bmjgh-2018-001286>

Zizien, Z. R., Korachais, C., Compaoré, P., Ridde, V., & De Brouwere, V. (2019). Contribution of the results‐based financing strategy to improving maternal and child health indicators in Burkina Faso. *The International Journal of Health Planning and Management, 34*(1), 111–129. <http://dx.doi.org/10.1002/hpm.2589>

Zombré, D., De Allegri, M., & Ridde, V. (2020). No effects of pilot performance‐based intervention implementation and withdrawal on the coverage of maternal and child health services in the koulikoro region, Mali: An interrupted time series analysis. *Health Policy and Planning, 35*(4), 379–387. <http://dx.doi.org/10.1093/heapol/czaa001>

## Appendix H: Table 2: Distribution of the quality of the 47 papers evaluated for risk of bias

|  | Q1: Research aim | Q2: Context | Q3: Literature | Q4: Theory based |
| --- | --- | --- | --- | --- |
| None | 1 | 1 | 6 | 27 |
| Weak | 6 | 3 | 13 | 3 |
| Strong | 39 | 42 | 27 | 16 |
|  | Q5: Sampling process | Q6: Sampling appropriateness | Q7: Sample characteristics described | Q8: Data collection |
| None | 5 | 6 | 16 | 5 |
| Weak | 18 | 22 | 16 | 23 |
| Strong | 23 | 18 | 14 | 18 |
|  | Q9: Data recording | Q10: Collection fits aims | Q11: Analytic method | Q12: Analysis checks |
| None | 8 | 3 | 16 | 29 |
| Weak | 15 | 13 | 9 | 10 |
| Strong | 23 | 30 | 21 | 7 |
|  | Q13: Reflexivity | Q14: Staff | Q15: Data presented | Q16: Diverse viewpoints |
| None | 38 | 17 | 8 | 9 |
| Weak | 4 | 15 | 12 | 11 |
| Strong | 4 | 14 | 26 | 26 |
|  | Q17: Evidence for each aim | Q18: Answer quality | Q19: Discussion | Q20: Triangulation |
| None | 2 | 3 | 4 | 9 |
| Weak | 10 | 19 | 11 | 23 |
| Strong | 34 | 24 | 31 | 14 |
|  | Q21: Weaknesses | Q22: Ethics^[[1]](#footnote-1)^ |  |  |
| None | 17 | 11 |  |  |
| Weak | 14 | -- |  |  |
| Strong | 15 | 35 |  |  |

## Appendix I: Table 3: Summary of all moderator analyses

|  | Exp IntMo | Eval InMo | Design | Year | Region | Card | Post int v CFB | Gov Implement | Cadres | Vaccine hesitency | Baseline coverage |
| --- | --- | --- | --- | --- | --- | --- | --- | --- | --- | --- | --- |
|  |  |  |  |  |  |  |  |  |  |  |  |
|  | β (SE) | β (SE) | β (SE) | β (SE) | β (SE) | β (SE) | β (SE) | β (SE) | β (SE) | β (SE) | β (SE) |
| All effects RVE (k = 56) | -0.001 (0.003) | 0.002 (0.006) | -0.10 (0.10) | -0.019* (0.007) | NP | 0.06 (0.05) | -0.08 (0.05) | -0.08 (0.04) | -0.02 (0.05) | -0.04 (0.05) | -0.31 (0.15) |
| Full Immunisation RVE (k = 28) | 0.0001 (0.002) | NP | -0.03 (0.09) | NP | NS (all comparisons) | 0.04 (0.11) | -0.16 (0.12) | -0.06 (0.10) | 0.02 (0.09) | 0.10 (0.19) | -0.13 (0.15) |
| Full Immunisation Ind RE (k = 28) | -0.0002 (0.004) | -0.01 (0.02) | -0.05 (0.11) | -0.01 (0.01) | NS (all comparisons) | 0.04 (0.12) | -0.13 (0.10) | 0.06 (0.12) | -0.06 (0.12) | 0.17 (0.11) | -0.33 (0.25) |
| Full Immunisation Ind EAI (k = 12) | -0.003* (0.002) | 0.01 (0.01) | -0.06 (0.06) | 0.01 (0.01) | 0.02  (0.07) | -0.01 (0.06) | -0.04 (0.08) | 0.08 (0.06) | -0.09 (0.06) | 0.08 (0.07) | -0.06 (0.11) |
| Full Immunisation Ind EID (k = 5) | N/A^c^ | N/A^c^ | N/A^c^ | N/A^c^ | N/A^c^ | N/A^c^ | N/A^c^ | N/A^c^ | N/A^c^ | N/A^c^ | N/A^c^ |
| Full Immunisation Ind EII (k = 2) | NP | NP | NP | NP | NP | NP | NP | NP | NP | NP | NP |
| Full Immunisation Ind MET (k = 9) | 0.005 (0.01) | -0.06 (0.06) | -0.12 (0.38) | -0.06 (0.03) | NS (all comparisons) | N/A^b^ | -0.49 (0.27) | 0.04 (0.35) | N/A^b^ | 0.33 (0.31) | -0.66 (0.68) |
| Partial Immunisation RVE (k = 9) | NP | NP | NP | NP | NP | NP | 0.10 (0.18) | NP | NP | 0.27 (0.12) | N/A^e^ |
| Partial Immunisation Ind RE (k = 9) | -0.003 (0.006) | -0.01 (0.01) | -0.10 (0.19) | -0.03 (0.03) | -0.03  (0.19) | 0.06 (0.21) | 0.09 (0.18) | -0.30 (0.20) | -0.30 (0.19) | 0.18 (0.19) | N/A^e^ |
| Partial Immunisation Ind EAI (k = 2) | NP | NP | NP | NP | NP | NP | NP | NP | NP | NP | N/A^e^ |
| Partial Immunisation Ind EID (k = 2) | NP | NP | NP | NP | NP | NP | NP | NP | NP | NP | N/A^e^ |
| Partial Immunisation Ind EII (k = 1) | NP | NP | NP | NP | NP | NP | NP | NP | NP | NP | N/A^e^ |
| Partial Immunisation Ind MET (k = 4) | 0.01 (0.02) | -0.01  (0.01) | -0.10 (0.35) | -0.02 0.06) | 0.05  (0.36) | N/A^b^ | 0.35 (0.33) | N/A^a^ | N/A^b^ | 0.08 (0.35) | N/A^e^ |
| Measles RVE (k = 20) | 0.0001 (0.001) | NP | -0.03 (0.05) | -0.001 (0.004) | NP | 0.01  (0.04) | -0.04  (0.03) | 0.03  (0.03) | 0.04 (0.04) | NP | N/A^e^ |
| Measles Ind RE (k = 20) | 0.000 (0.001) | -0.003 (0.003) | -0.05 (0.06) | -0.003 (0.007) | NS (all comparisons) | 0.02 (0.07) | -0.04 (0.06) | 0.06 (0.06) | 0.05 (0.06) | 0.13 (0.09) | N/A^d^ |
| Measles: Ind EAI (k = 10) | -0.001 (0.001) | -0.004 (0.006) | 0.07 (0.05) | 0.004 (0.01) | NS (all comparisons) | 0.02 (0.08) | 0.05 (0.05) | -0.003 (0.05) | 0.03 (0.05) | 0.10 (0.09) | N/A^d^ |
| Measles: Ind EID (k = 2) | NP | NP | NP | NP | NP | NP | NP | NP | NP | NP | N/A^d^ |
| Measles: Ind EII (k = 2) | NP | NP | NP | NP | NP | NP | NP | NP | NP | NP | N/A^d^ |
| Measles: Ind MET (k = 6) | 0.004 (0.01) | -0.004 (0.01) | -0.15 (0.31) | -0.04 (0.03) | 0.18  (0.25) | 0.07 (0.33) | -0.33 (0.19) | 0.31 (0.20) | 0.18 (0.25) | N/A^b^ | N/A^d^ |
| BCG RVE (k = 12) | NP | NP | -0.10 (0.10) | -0.03 (0.02) | -0.08  (0.12) | NP | -0.12 (0.10) | -0.10 (0.14) | NP | 0.37*** (0.03) | N/A^d^ |
| BCG Ind RE (k = 12) | 0.001 (0.004) | -0.01 (0.01) | -0.05 (0.11) | -0.03 ** (0.01) | 0.004  (0.11) | -0.02 (0.04) | -0.12 (0.12) | -0.04 (0.13) | 0.06 (0.13) | N/A^b^ | N/A^d^ |
| BCG: Ind EAI (k = 4) | N/A^c^ | N/A^c^ | N/A^c^ | N/A^c^ | N/A^c^ | N/A^c^ | N/A^c^ | N/A^c^ | N/A^c^ | N/A^c^ | N/A^d^ |
| BCG: Ind EID (k = 2) | NP | NP | NP | NP | NP | NP | NP | NP | NP | NP | N/A^d^ |
| BCG: Ind EII (k = 2) | NP | NP | NP | NP | NP | NP | NP | NP | NP | NP | N/A^d^ |
| BCG: Ind MET (k = 4) | -0.002 (0.02) | N/A^b^ | N/A^b^ | -0.08  (0.04) | 0.03  (0.41) | N/A^a^ | -0.55*** (0.07) | 0.03 (0.41) | 0.03 (0.41) | N/A^b^ | N/A^d^ |
| DPT1: RVE (k = 8) | NP | NP | -0.10  (0.10) | 0.001 (0.01) | NP | NP | NP | NP | NP | 0.07 (0.03) | -0.13 (0.20) |
| DPT1: Ind RE (k = 8) | -0.005  (0.01) | N/A^a^ | -0.15* (0.07) | -0.004 (0.01) | NS (all comparisons) | -0.08 (0.10) | 0.02 (0.11) | 0.17* (0.08) | N/A^b^ | N/A^b^ | N/A^e^ |
| DPT1: Ind EAI (k = 3) | NP | NP | NP | NP | NP | NP | NP | NP | NP | NP | NP |
| DPT1: Ind EID (k = 2) | NP | NP | NP | NP | NP | NP | NP | NP | NP | NP | NP |
| DPT1: Ind EII (k = 1) | NP | NP | NP | NP | NP | NP | NP | NP | NP | NP | NP |
| DPT1: Ind MET (k = 2) | NP | NP | NP | NP | NP | NP | NP | NP | NP | NP | NP |
| DPT2 RVE (k = 5) | NP | NP | NP | NP | NP | NP | NP | NP | NP | NP | NP |
| DPT2 Ind RE (k = 5) | N/A^c^ | N/A^c^ | N/A^c^ | N/A^c^ | N/A^c^ | N/A^c^ | N/A^c^ | N/A^c^ | N/A^c^ | N/A^c^ | N/A^c^ |
| DPT2 Ind EAI (k = 0) | NP | NP | NP | NP | NP | NP | NP | NP | NP | NP | NP |
| DPT2 Ind EID (k = 2) | NP | NP | NP | NP | NP | NP | NP | NP | NP | NP | NP |
| DPT2 Ind EII (k = 0) | NP | NP | NP | NP | NP | NP | NP | NP | NP | NP | NP |
| DPT2 Ind MET (k = 3) | NP | NP | NP | NP | NP | NP | NP | NP | NP | NP | NP |
| DPT3 RVE (k = 22) | 0.0003 (0.001) | NP | -0.03 (0.05) | -0.02 (0.01) | -0.01  (0.05) | NP | -0.01 (0.05) | -0.04 (0.05) | NP | NP | -0.50  (0.21) |
| DPT3 Ind RE (k = 22) | -0.001 (0.001) | -0.002 (0.004) | 0.05 (0.05) | -0.014* (0.006) | -0.02  (0.05) | 0.14 (0.09) | -0.01 (0.05) | -0.01 (0.05) | -0.03 (0.06) | 0.04 (0.06) | -0.40 (0.22) |
| DPT3 Ind EAI (k = 6) | -0.001 (0.002) | 0.01 (0.01) | -0.05 (0.07) | 0.01 (0.01) | N/A^b^ | N/A^a^ | -0.08 (0.07) | 0.05 (0.07) | -0.04 (0.07) | -0.03 (0.08) | -0.40 (0.22) |
| DPT3 Ind EID (k = 6) | N/A^c^ | N/A^c^ | N/A^c^ | N/A^c^ | N/A^c^ | N/A^c^ | N/A^c^ | N/A^c^ | N/A^c^ | N/A^c^ | N/A^c^ |
| DPT3 Ind EII (k = 3) | NP | NP | NP | NP | NP | NP | NP | NP | NP | NP | NP |
| DPT3 Ind MET (k =7) | 0.003 (0.006) | -0.01** (0.004) | 0.07 (0.16) | -0.03* (0.01) | -0.10  (0.15) | N/A^a^ | -0.25 (0.15) | 0.05 (0.17) | -0.04 (0.17) | -0.02 (0.17) | -0.56** (0.18) |
| OPV0 RVE (k = 5) | NP | NP | NP | NP | NP | NP | NP | NP | NP | NP | NP |
| OPV0 Ind RE (k = 5) | 0.02*** (0.004) | N/A^a^ | 0.16 (0.19) | -0.07** (0.02) | -0.20  (0.18) | 0.16 (0.19) | N/A^b^ | -0.21 (0.17) | N/A^a^ | N/A^a^ | N/A^d^ |
| OPV0 Ind EAI (k = 1) | NP | NP | NP | NP | NP | NP | NP | NP | NP | NP | N/A^d^ |
| OPV0 Ind EID (k = 2) | NP | NP | NP | NP | NP | NP | NP | NP | NP | NP | N/A^d^ |
| OPV0 Ind EII (k = 1) | NP | NP | NP | NP | NP | NP | NP | NP | NP | NP | N/A^d^ |
| OPV0 Ind MET (k = 1) | NP | NP | NP | NP | NP | NP | NP | NP | NP | NP | N/A^d^ |
| OPV1 RVE (k = 5) | NP | NP | NP | NP | NP | NP | NP | NP | NP | NP | N/A^d^ |
| OPV1 Ind RE (k = 5) | N/A^c^ | N/A^c^ | N/A^c^ | N/A^c^ | N/A^c^ | N/A^c^ | N/A^c^ | N/A^c^ | N/A^c^ | N/A^c^ | N/A^d^ |
| OPV01 Ind EAI (k = 0) | NP | NP | NP | NP | NP | NP | NP | NP | NP | NP | N/A^d^ |
| OPV1 Ind EID (k = 2) | NP | NP | NP | NP | NP | NP | NP | NP | NP | NP | N/A^d^ |
| OPV1 Ind EII (k = 1) | NP | NP | NP | NP | NP | NP | NP | NP | NP | NP | N/A^d^ |
| OPV1 Ind MET (k = 2) | NP | NP | NP | NP | NP | NP | NP | NP | NP | NP | N/A^d^ |
| OPV2 RVE (k = 5) | NP | NP | NP | NP | NP | NP | NP | NP | NP | NP | N/A^d^ |
| OPV2 Ind RE (k = 5) | 0.02***  (0.003) | N/A^a^ | 0.22  (0.18) | -0.05**  (0.02) | -0.26***  (0.07) | 0.27  (0.07) | N/A^b^ | 0.03  (0.25) | N/A^b^ | N/A^a^ | N/A^d^ |
| OPV02 Ind EAI (k = 0) | NP | NP | NP | NP | NP | NP | NP | NP | NP | NP | N/A^d^ |
| OPV2 Ind EID (k = 2) | NP | NP | NP | NP | NP | NP | NP | NP | NP | NP | N/A^d^ |
| OPV2 Ind EII (k = 1) | NP | NP | NP | NP | NP | NP | NP | NP | NP | NP | N/A^d^ |
| OPV2 Ind MET (k = 2) | NP | NP | NP | NP | NP | NP | NP | NP | NP | NP | N/A^d^ |
| OPV3 RVE (k = 9) | NP | NP | -0.03  (0.05) | NP | -0.24  (0.31) | NP | 0.26  (0.30) | NP | -0.18  (0.33) | NP | N/A^d^ |
| OPV3 Ind RE (k = 9) | -0.001  (0.01) | -0.01  (0.01) | 0.17  (0.23) | -0.03  (0.03) | -0.12  (0.23) | 0.04  (0.32) | 0.14  (0.25) | -0.34  (0.27) | -0.03  (0.27) | N/A^a^ | N/A^d^ |
| OPV3 Ind EAI (k = 3) | NP | NP | NP | NP | NP | NP | NP | NP | NP | NP | N/A^d^ |
| OPV3 Ind EID (k = 1) | NP | NP | NP | NP | NP | NP | NP | NP | NP | NP | N/A^d^ |
| OPV3 Ind EII (k = 2) | NP | NP | NP | NP | NP | NP | NP | NP | NP | NP | N/A^d^ |
| OPV3 Ind MET (k = 3) | NP | NP | NP | NP | NP | NP | NP | NP | NP | NP | N/A^d^ |
| Timeliness RVE (k = 11) | NP | NP | NP | NP | NP | NP | NP | NP | NP | NP | N/A^d^ |
| DPT3 Timeliness RE (k = 7) | N/A^c^ | N/A^c^ | N/A^c^ | N/A^c^ | N/A^c^ | N/A^c^ | N/A^c^ | N/A^c^ | N/A^c^ | N/A^c^ | N/A^c^ |
| DPT3 Timeliness EAI (k = 0) | -- | -- | -- | -- | -- | -- | -- | -- | -- | -- | -- |
| DPT3 Timeliness EID (k = 4) | N/A^c^ | N/A^c^ | N/A^c^ | N/A^c^ | N/A^c^ | N/A^c^ | N/A^c^ | N/A^c^ | N/A^c^ | N/A^c^ | N/A^c^ |
| DPT3 Timeliness EII (k =2) | NP | NP | NP | NP | NP | NP | NP | NP | NP | NP | NP |
| DPT3 Timeliness MET (k = 1) | NP | NP | NP | NP | NP | NP | NP | NP | NP | NP | NP |
| Measles Timeliness RE (k = 2) | NP | NP | NP | NP | NP | NP | NP | NP | NP | NP | NP |
| Measles Timeliness EAI (k = 0) | -- | -- | -- | -- | -- | -- | -- | -- | -- | -- | -- |
| Measles Timeliness EID (k = 1) | NP | NP | NP | NP | NP | NP | NP | NP | NP | NP | NP |
| Measles Timeliness EII (k =0) | -- | -- | -- | -- | -- | -- | -- | -- | -- | -- | -- |
| Measles Timeliness MET (k = 1) | NP | NP | NP | NP | NP | NP | NP | NP | NP | NP | NP |
| FI Timeliness RE (k =5) | N/A^c^ | N/A^c^ | N/A^c^ | N/A^c^ | N/A^c^ | N/A^c^ | N/A^c^ | N/A^c^ | N/A^c^ | N/A^c^ | N/A^c^ |
| FI Timeliness EAI (k = 1) | NP | NP | NP | NP | NP | NP | NP | NP | NP | NP | NP |
| FI Timeliness EID (k = 2) | NP | NP | NP | NP | NP | NP | NP | NP | NP | NP | NP |
| FI Timeliness EII (k = 2) | NP | NP | NP | NP | NP | NP | NP | NP | NP | NP | NP |
| FI Timeliness MET (k = 0) | -- | -- | -- | -- | -- | -- | -- | -- | -- | -- | -- |
| Dropout RVE  (k = 5) | NP | NP | NP | NP | NP | NP | NP | NP | NP | NP | NP |
| Dropout RE (k = 5) | 0.04  (0.04) | 0.03  (0.03) | N/A^a^ | 0.33**  (0.12) | -0.050  (0.183) | N/A^b^ | N/A^a^ | -0.23  (0.13) | N/A^a^ | 0.05  (0.18) | N/A^d^ |
| Dropout EAI (k =2 ) | NP | NP | NP | NP | NP | NP | NP | NP | NP | NP | NP |
| Dropout EID (k = 0) | -- | -- | -- | -- | -- | -- | -- | -- | -- | -- | -- |
| Dropout EII (k =1 ) | NP | NP | NP | NP | NP | NP | NP | NP | NP | NP | NP |
| Dropout MET (k = 2) | NP | NP | NP | NP | NP | NP | NP | NP | NP | NP | NP |
| Morbidity RVE  (k = 10) | NP | NP | NP | 0.01  (0.01) | NP | N/A^e^ | 0.02  (0.08) | NP | NP | N/A^a^ | N/A^e^ |
| Morbidity RE (k = 10) | 0.003 (0.005) | -0.01 (0.01) | -0.04 0.11 | 0.01 (0.01) | NS (all comparisons) | -0.08 (0.10) | 0.13 (0.15) | 0.08 (0.11) | -0.05 (0.13) | N/A^a^ | N/A^e^ |
| Morbidity EAI (k = 5) | 0.02** (0.01) | -0.01 (0.02) | N/A^b^ | 0.03 (0.05) | 0.04  (0.22) | N/A^d^ | 0.12  (0.21) | 0.26 (0.22) | 0.12 (0.27) | N/A^a^ | N/A^e^ |
| Morbidity EID (k = 2) | NP | NP | NP | NP | NP | NP | NP | NP | NP | NP | NP |
| Morbidity EII (k = 1) | NP | NP | NP | NP | NP | NP | NP | NP | NP | NP | NP |
| Morbidity MET (k = 2) | NP | NP | NP | NP | NP | NP | NP | NP | NP | NP | NP |
| MORTALITY: RVE (k = 6) | NP | NP | NP | NP | NP | N/A^e^ | NP | NP | NP | N/A^a^ | N/A^d^ |
| MORTALITY RE (6) | -0.002  (0.001) | 0.002 (0.002) | 0.03  (0.05) | 0.01***  (0.003) | NS (all comparisons) | N/A^d^ | -0.05  (0.05) | 0.02  (0.05) | 0.02  (0.05) | N/A^a^ | N/A^d^ |
| MORTALITY EAI (3) | NP | NP | NP | NP | NP | NP | NP | NP | NP | NP | NP |
| MORTALITY EID (0) | NP | NP | NP | NP | NP | NP | NP | NP | NP | NP | NP |
| MORTALITY EII (0) | NP | NP | NP | NP | NP | NP | NP | NP | NP | NP | NP |
| MORTALITY MET (3) | NP | NP | NP | NP | NP | NP | NP | NP | NP | NP | NP |
| Knowledge RVE (k = 7) | NP | NP | -0.10 (0.10) | NP | NP | NP | -0.06 (0.13) | NP | NP | NP | N/A^d^ |
| Knowledge RE (k = 9) | -0.02  (0.01) | -0.01  (0.02) | 0.44***  (0.11) | -0.01  (0.02) | 0.44***  (0.11) | N/A^d^ | -0.05  (0.18) | 0.05  (0.23) | N/A^a^ | -0.01  (0.23) | N/A^d^ |
| Knowledge EAI (k = 4) | N/A^e^ | N/A^e^ | 0.52*  (0.21) | -0.05  (0.05) | 0.52*  (0.21) | N/A^e^ | N/A^b^ | N/A^b^ | N/A^a^ | N/A^a^ | N/A^d^ |
| Knowledge EID (k = 3) | NP | NP | NP | NP | NP | NP | NP | NP | NP | NP | NP |
| Knowledge EII (k = 0) | -- | -- | -- | -- | -- | -- | -- | -- | -- | -- | -- |
| Knowledge MET (k = 2) | NP | NP | NP | NP | NP | NP | NP | NP | NP | NP | NP |
| Attitudes RVE  (No dependent effects) | N/A | N/A | N/A | N/A | N/A | N/A | N/A | N/A | N/A |  |  |
| Attitudes RE (k = 6) | 0.01  (0.02) | -0.004  (0.02) | 0.17  (0.34) | -0.01  (0.04) | 0.17  (0.34) | N/A^e^ | 0.38  (0.29) | -0.09  (0.43) | N/A^a^ | N/A^b^ | N/A^d^ |
| Attitudes EAI (k = 2) | NP | NP | NP | NP | NP | NP | NP | NP | NP | NP | NP |
| Attitudes EID (k = 2) | NP | NP | NP | NP | NP | NP | NP | NP | NP | NP | NP |
| Attitudes EII (k = 0) | -- | -- | -- | -- | -- | -- | -- | -- | -- | -- | -- |
| Attitudes MET (k = 2) | NP | NP | NP | NP | NP | NP | NP | NP | NP | NP | NP |
| Card Avail/ Retention RVE  (No dependent effects) | N/A | N/A | N/A | N/A | N/A | N/A | N/A | N/A | N/A | N/A | N/A |
| Card Avail/ Retention RE (k = 4) | N/A^c^ | N/A^c^ | N/A^c^ | N/A^c^ | N/A^c^ | N/A^c^ | N/A^c^ | N/A^c^ | N/A^c^ | N/A^c^ | N/A^d^ |
| Card Avail/ Retention EAI (k = 1) | NP | NP | NP | NP | NP | NP | NP | NP | NP | NP | NP |
| Card Avail/ Retention EID (k = 0) | -- | -- | -- | -- | -- | -- | -- | -- | -- | -- | -- |
| Card Avail/ Retention EII (k = 1) | NP | NP | NP | NP | NP | NP | NP | NP | NP | NP | NP |
| Card Avail/ Retention MET (k = 2) | NP | NP | NP | NP | NP | NP | NP | NP | NP | NP | NP |
| SATISFACTION W/ HEALTH WORKERS  (K = 2) | NP | NP | NP | NP | NP | NP | NP | NP | NP | NP | NP |
| SATISFACTION W/ HEALTH WORKERS  (k = 1) | NP | NP | NP | NP | NP | NP | NP | NP | NP | NP | NP |
| SATISFACTION W/ HEALTH WORKERS  EAI (k = 1) | NP | NP | NP | NP | NP | NP | NP | NP | NP | NP | NP |
| SATISFACTION W/ HEALTH WORKERS  EID (k = 1) | NP | NP | NP | NP | NP | NP | NP | NP | NP | NP | NP |
| SATISFACTION W/ HEALTH WORKERS  EII (k = 0) | -- | -- | -- | -- | -- | -- | -- | -- | -- | -- | -- |
| SATISFACTION W/ HEALTH WORKERS  MET (k = 0) | -- | -- | -- | -- | -- | -- | -- | -- | -- | -- | -- |
| formal HEALTH WORKERS motivation, capacity, and performance (K = 2) | NP | NP | NP | NP | NP | NP | NP | NP | NP | NP | NP |
| formal HEALTH WORKERS motivation, capacity, and performance (k = 1) | NP | NP | NP | NP | NP | NP | NP | NP | NP | NP | NP |
| formal HEALTH WORKERS motivation, capacity, and performance EAI (k = 1) | NP | NP | NP | NP | NP | NP | NP | NP | NP | NP | NP |
| formal HEALTH WORKERS motivation, capacity, and performance EID (k = 1) | NP | NP | NP | NP | NP | NP | NP | NP | NP | NP | NP |
| formal HEALTH WORKERS motivation, capacity, and performance EII (k = 0) | -- | -- | -- | -- | -- | -- | -- | -- | -- | -- | -- |
| formal HEALTH WORKERS motivation, capacity, and performance MET (k = 0) | -- | -- | -- | -- | -- | -- | -- | -- | -- | -- | -- |

Note. NP = not powered

^a^ Not applicable because all studies were in the same moderator group

^b^ Not applicable because only one study was in a different moderator group

^c^ Not applicable because there was no heterogeneity

^d^ Not applicable because the moderator does not apply to the outcome category

^e^ Not applicable because there was missing data

## Appendix J: Qualitative synthesis – hierarchy charts and representative quotes

Barriers to immunisation by type of community engagement

Engagement as intervention


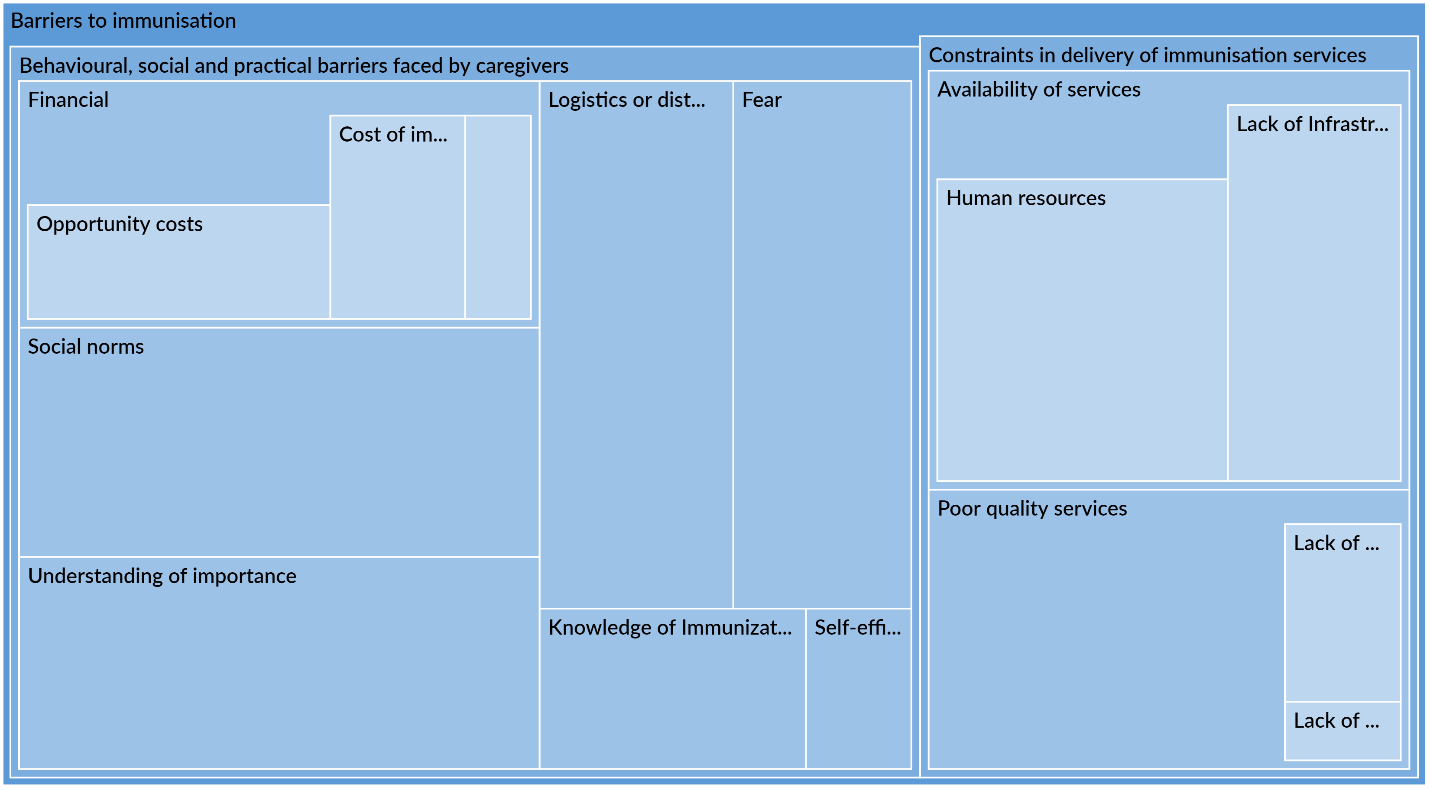


Engagement in design


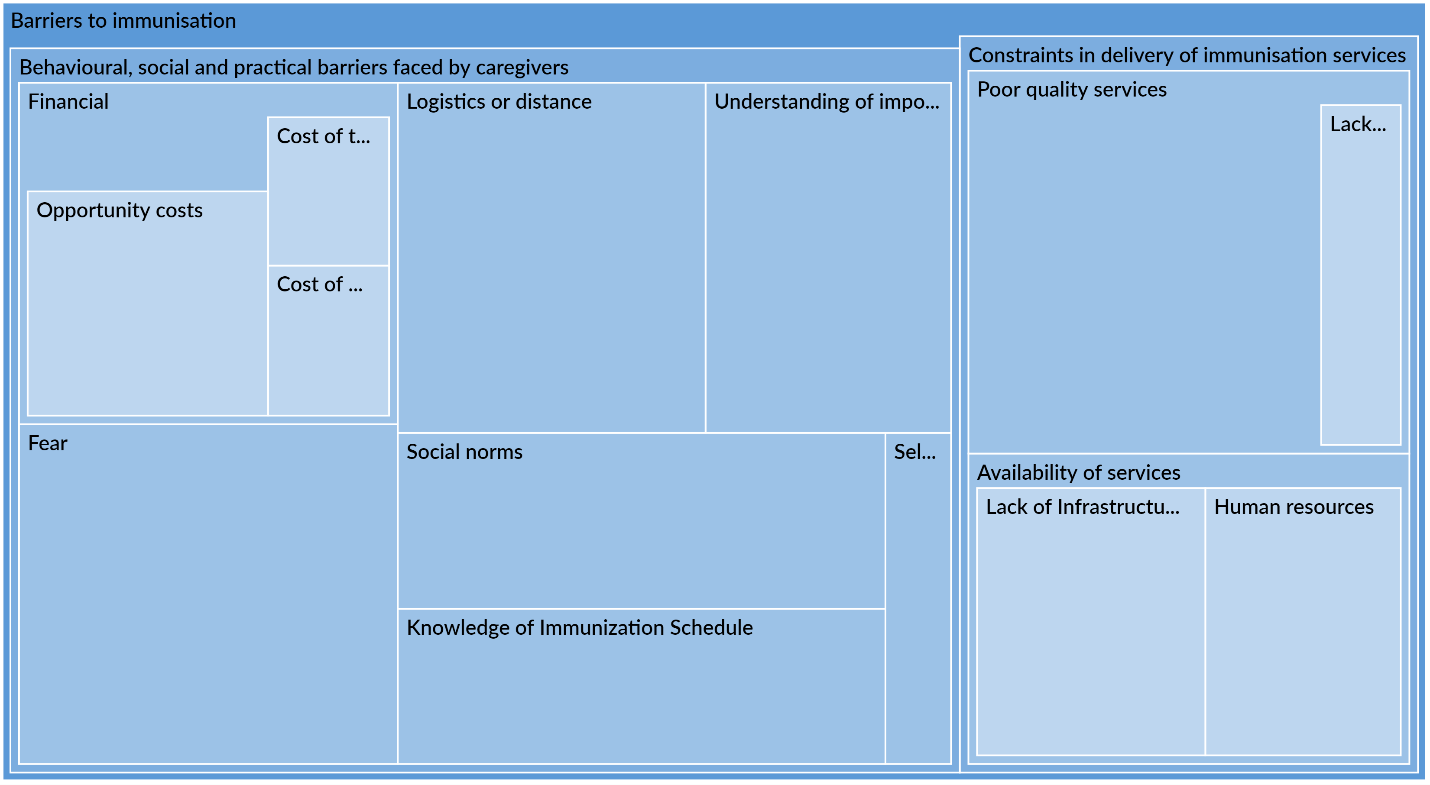


Engagement in implementation


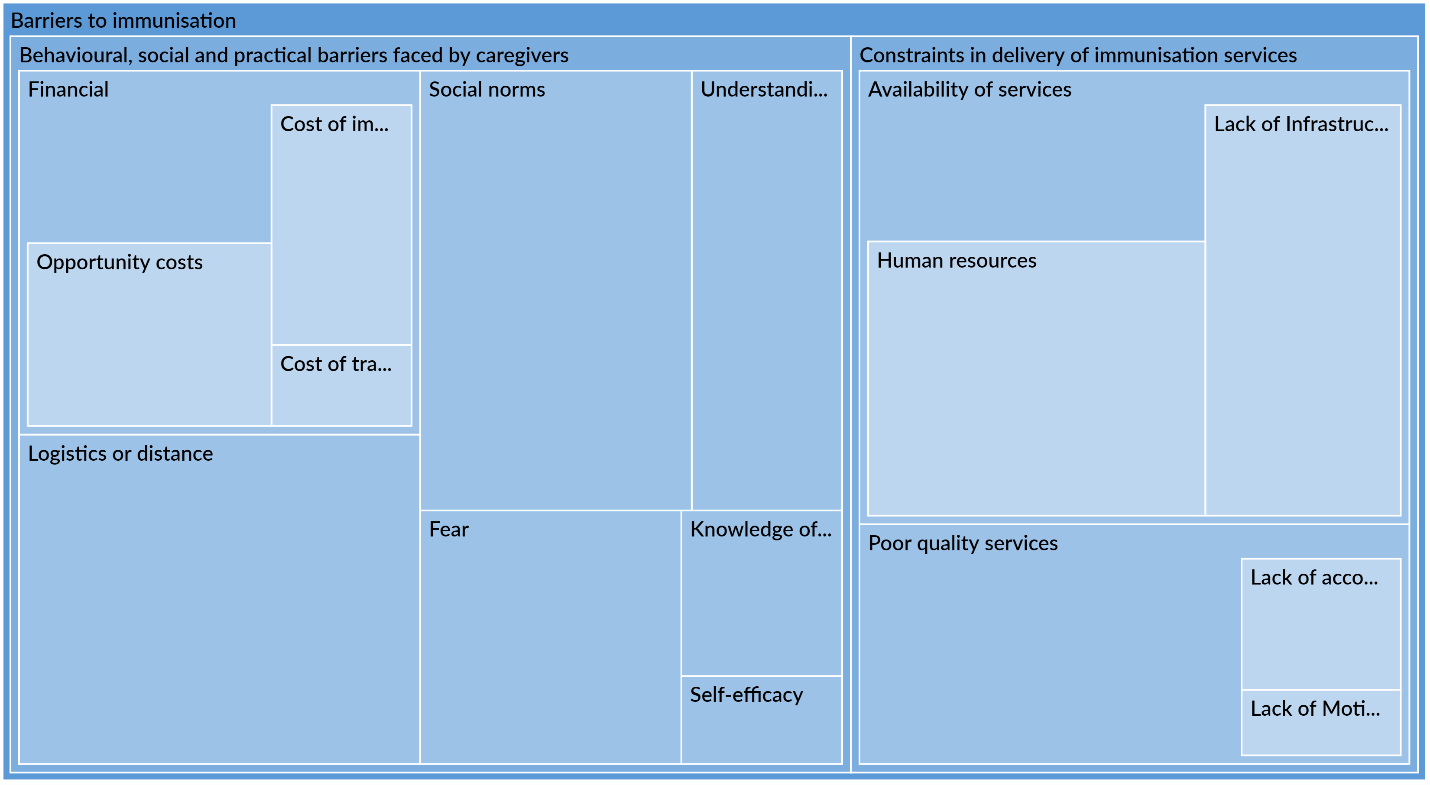


Table 4: Characteristic quotes of themes identified related to barriers to immunisation for interventions were community engagement.

| **Primary paper** | **Paper from which citation comes** | **Theme demonstrated** | **Quote** | **Page number** |
| --- | --- | --- | --- | --- |
| *Financial* | |  |  |  |
| Okeke et al 2017 | Okeke et al 2017 | Cost of vaccination | costs associated with use of services and lack of adequate transportation, hindered uptake. | 54 |
| Andersson 2009 | Mitchell 2009 | Other costs | Yet the groups noted that immediate financial and time costs associated with having a child vaccinated weigh very heavily in comparison with the potential costs associated with measles in the future. | 7 |
| *Social norms* | |  |  |  |
| Banerjee et al 2020 | Appendix K | Religion | Religion emerged as an implicit theme. Only two respondents mentioned explicitly that religious belief might inhibit immunisation: that God would decide the fate of their child. | 7 |
| Andersson 2009 | Cockroft 2009 | Beliefs of others | “My mother-in-law says ‘What kind of children have you produced that they ought to be vaccinated? We were never vaccinated, so why are you behaving so delicately?’” (female group, Khanewal) | 8 |
| *Understanding of importance* | | |  |  |
| Olken 2014 | Grayman 2013 | Costs and importance | Typically the answer is that the parents are too busy working on their farms, or they feel that posyandu is no longer necessary after they are older than two years of age. | 66 |
| Paramanik 2018 | Paramanik 2020 | Fear and importance | As reported by ASHAs the mothers-in-law were not able to accept the concept of vaccination. They apparently argued on the ground that they had children too and that they never took them for any vaccination, that post vaccination the child gets fever which causes sleep disturbance for the child and in turn negatively affects the whole family. | 84 |
| *Logistics or distance* | |  |  |  |
| Alhassan 2019 | Alhassan 2019 | Distance | In Ghana, access to maternal and child health services is impeded by longer travel times to health facilities. | 2 |
| Oyo-Ita 2020 | Oyo-Ita 2020 | Distance | children whose mothers found it difficult to reach the health facility |  |
| *Fear* | |  |  |  |
| Banerjee et al 2020 | Appendix K | Trust | A couple of respondents suggested this could be interacting with a lack of trust of front line health workers. | 20 |
| Oyo-Ita 2020 | Oyo-Ita 2020 | Side effects | She said she doesn’t want anybody to give her child injection so that the child will not become sick | 31 |
| Gurley 2020 | Gurley 2020 | Overcoming fear of side effects | We observed changes in how FGD respondents discussed side effects over the course of the study, from a perception that they were severe and not worth the risk of vaccination, to a sense they could be handled and are not a reason to forgo vaccination. I also took my child for vaccination. She cried for two days and I said let her cry; at least she will be safe in future from diseases. Mothers FGD, September 2017, intervention village | 35 |
| *Human resources* | |  |  |  |
| Gurley 2020 | Gurley 2020 | Staff | In addition to suboptimal ANM clinical quality, nine ANM posts were vacant during the study period, resulting in suboptimal availability of services in some communities, which was beyond our influence | 38 |
| Olken 2014 | Rahayu 2008 | Absenteeism | the community is often disappointed with the services of puskesmas officers who do not attend posyandu services as this means that immunisations must be postponed until the posyandu session scheduled for the next month. | 33 |
| *Infrastructure and supplies* | |  |  |  |
| Okeke et al 2017 | Okeke et al 2017 | Building infrastructure | That is supposed to be our office. If you get there now, half of it is just sand; even the doors to this clinic are not closing, they are eating up by termite. the condition of two of the facilities was perceived to be detrimental to their ability to deliver care. | 43 |
| Findley et al 2013 | Doctor et al 2011 | Vaccine stockout and long wait times | 67% of parents were unable to receive all immunisations reported lack of vaccine as a problem, and 13% had difficulties with the long wait. | 17 |
| *Multiple barriers* | |  |  |  |
| Okeke et al 2017 | Okeke et al 2017 | Cost of vaccination and logistics | costs associated with use of services and lack of adequate transportation, hindered uptake. |  |
| Arifeen 2019 | Billah 2018 | Many simultaneous barriers | Furthermore, the lack of trust in formal health care providers, high out of pocket costs of health care in the public sector, and challenges with accessibility has led to the rapid proliferation of informal health providers to fill the gap between supply and demand across both rural and urban areas | 2 |
| Tandon 1988 | Islam 2013 | Many simultaneous barriers | low capacity to supervise monitor and implement micro plans at district level, lack of effective vaccine distribution to immunisation sites, ageing and poorly maintained Cold Chain, lack of adequately trained human resources, low managerial and support capacity at the state and district immunisation units and weak management of fund flows. | 10 |

Table 5: Characteristic quotes of themes identified related to barriers to immunisation for interventions that used community engagement in their design.

| **Primary paper** | **Paper from which citation comes** | **Theme demonstrated** | **Quote** | **Page number** |
| --- | --- | --- | --- | --- |
| *Financial* | |  |  |  |
| Anderson 2009 | Andersson 2009 | Other costs | Discussions in our focus groups confirmed the importance of poverty as a barrier to vaccination in many cases, as parents described being unable to afford the costs of the supposedly “free” immunisations: travel costs, opportunity costs, and demands for unofficial payments. | 10 |
| *Fear* | |  |  |  |
| Dipeolu 2017 | Oladepo 2019 | Fear of side effects | Prominent factors perceived as affecting timely and full completion of routine immunisations for children aged below 12 months include lack of awareness of immunisation (61.6%) and of subsequent doses (58.4%), and fear of side effects (59.7%). Others include rumours (for example, an association with future infertility) | 464 |
| Adamu 2019 | Adamu 2019 Dissertation | Past experience | Experiences with the side effects of vaccines can influence the behaviour of caregivers and reduce their motivation to immunize eligible children. | 134 |
| *Logistics* | |  |  |  |
| Andersson 2009 | Mitchell 2009 | Physical access to facilities | In both urban and rural areas, access to a government facility providing vaccinations, a key equity factor, was a determining factor for uptake. | 8 |
| Nagar 2018 | Nagar 2016 | Convenience | not knowing where to go, not having time or mutually convenient time, facing long wait times at the camp, having fear of side effects, and acting under misguided advice | 5 |
| *Human resources* | |  |  |  |
| Borkum 2014 | Rangarajan 2013 | Staff | Staff vacancies are common, especially for lady health visitors and pharmacists. Few of the PHCs surveyed are fully staffed. |  |
| Infrastructure or supplies | |  |  |  |
| Domek 2019 | Domek et al 2018 Vaccine hesitancy | Stock outs | Of note, Guatemala experienced significant political instability during our study period, which led to considerable vaccine shortages experienced by all of our clinics. | 3 |
| *Poor quality* | |  |  |  |
| Dipeolu 2017 | Dipeolu 2017 | Attitudes of health workers | Attitudes and behaviours of healthcare workers such as treating mothers in an unfriendly, disrespectful, or even abusive manner are frequently cited as discouraging children’s vaccination. Healthcare workers reportedly screamed at mothers who forgot the child’s card, missed a scheduled vaccination | 49 |
| Gurely 2020 | Gurley 2020 | Quality affected attendance | For example, the existence of ANM vaccinators did not ensure their quality, and we observed that suboptimal clinical ANM quality discouraged retention across the vaccine schedule and perhaps negatively impacted beneficiaries’ trust in the health system. | 37 |

Table 6: Characteristic quotes of themes identified related to barriers to immunisation for interventions that used community engagement in their implementation

| **Primary paper** | **Paper from which citation comes** | **Theme demonstrated** | **Quote** | **Page number** |
| --- | --- | --- | --- | --- |
| *Financial* | |  |  |  |
| Okeke et al 2017 | Okeke et al 2017 | Cost of the vaccine itself | costs associated with use of services and lack of adequate transportation, hindered uptake. | 54 |
| Adamu 2019 | Adamu 2019 Dissertation | Other costs associated with vaccination | However, the cost of treating vaccine reactions like fever that might occur following immunisation are borne for the caregivers, which can result in out-of-pocket expenditure. | 133 |
| *Social norms* | |  |  |  |
| Adamu 2019 | Adamu 2019 Dissertation | Spousal consent | In addition, we found that the high level of social control that men have over women in this area could also cause MOV among children. Caregivers reported that without their husband’s consent, they still cannot vaccinate their children, even if they’re in a health facility for other preventive or curative services. | 133 |
| Olken 2014 | Rahayu 2008 | Religious beliefs | The community trusts and believes in the choice that their parents made to use the dukun beranak, to the point where it becomes the norm. | 34 |
| *Logistics or distance* | |  |  |  |
| Webster 2019 | Webster et al 2019 | Convenience | Key reasons cited for non-immunisation were related to lack of convenience; the aforementioned distance between homes and health facilities, moving to the fields during farming seasons, | 42 |
| Alhassan 2019 | Alhassan 2019 | Transport | In Ghana, access to maternal and child health services is impeded by longer travel times to health facilities. | 2 |
| *Human resources* | |  |  |  |
| Olken 2014 | Febriany 2011 | Post abandonment | Poor facilities adversely affected the availability and utilization of MCH and basic education services. The midwife did not want to live in the village because here there is no electricity and no water (FGD Female-NTT). | 9 |
| Herrara-Almanza 2018 | Herrara-Almanza & Rossales-Rueda 2018 | Lack of personnel | Poor transportation infrastructure and a shortage of medical personnel limit the basic health access to the population living in remote areas | 7 |
| *Infrastructure or supplies* | |  |  |  |
| Webster 2019 | Webster et al 2019 | Vaccine stockouts | Furthermore, vaccine stock-outs at health facilities discouraged caregivers from attending vaccination services. | 42 |
| *Multiple barriers* | |  |  |  |
| Webster 2019 | Webster et al 2019 | Demand and supply side | Health system factors affecting immunisation service delivery and uptake in northern Uganda include interruptions in the vaccine cold chain due to poor management of equipment and supplies, limited supervision of health teams by the district health teams, low staffing levels, long distances to the health facilities especially in the hard-to-reach areas, and limited resources to support outreach services. There is also a lack of good quality data to support decision-making. | 1 |
| Olken 2014 | Rahayu 2008 | Poor quality and post abandonment | Dissatisfaction is often related to the midwife’s character, ineffective medicine, minimal experience of the midwife, difficulties in reaching the midwife, and a midwife’s absence from the post. | 68 |
| Herrara-Almanza 2018 | Herrara-Almanza & Rossales-Rueda 2018 | Transport and personnel | Poor transportation infrastructure and a shortage of medical personnel limit the basic health access to the population living in remote areas | 7 |

Facilitators of immunisation by type of community engagement


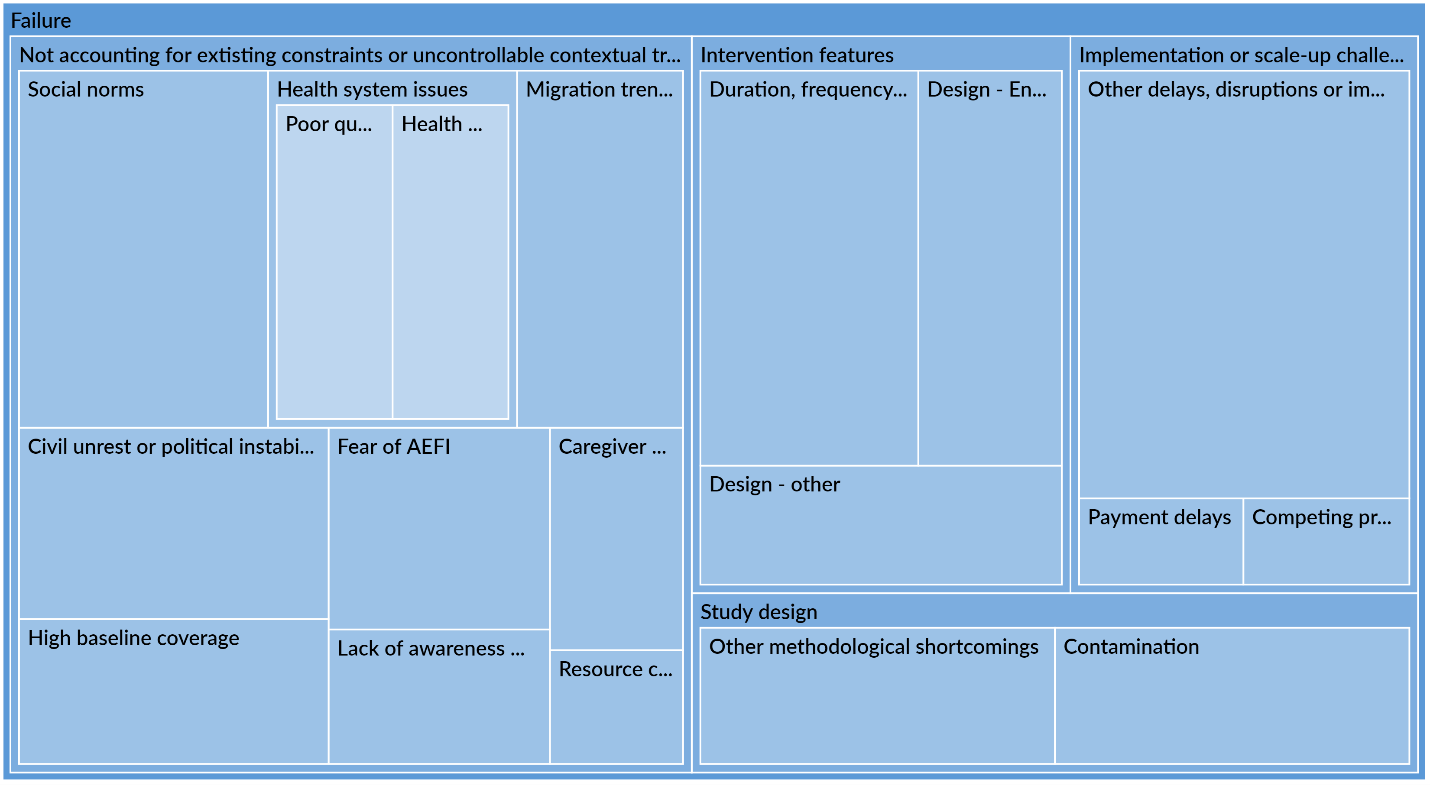
Engagement as intervention

Engagement in design


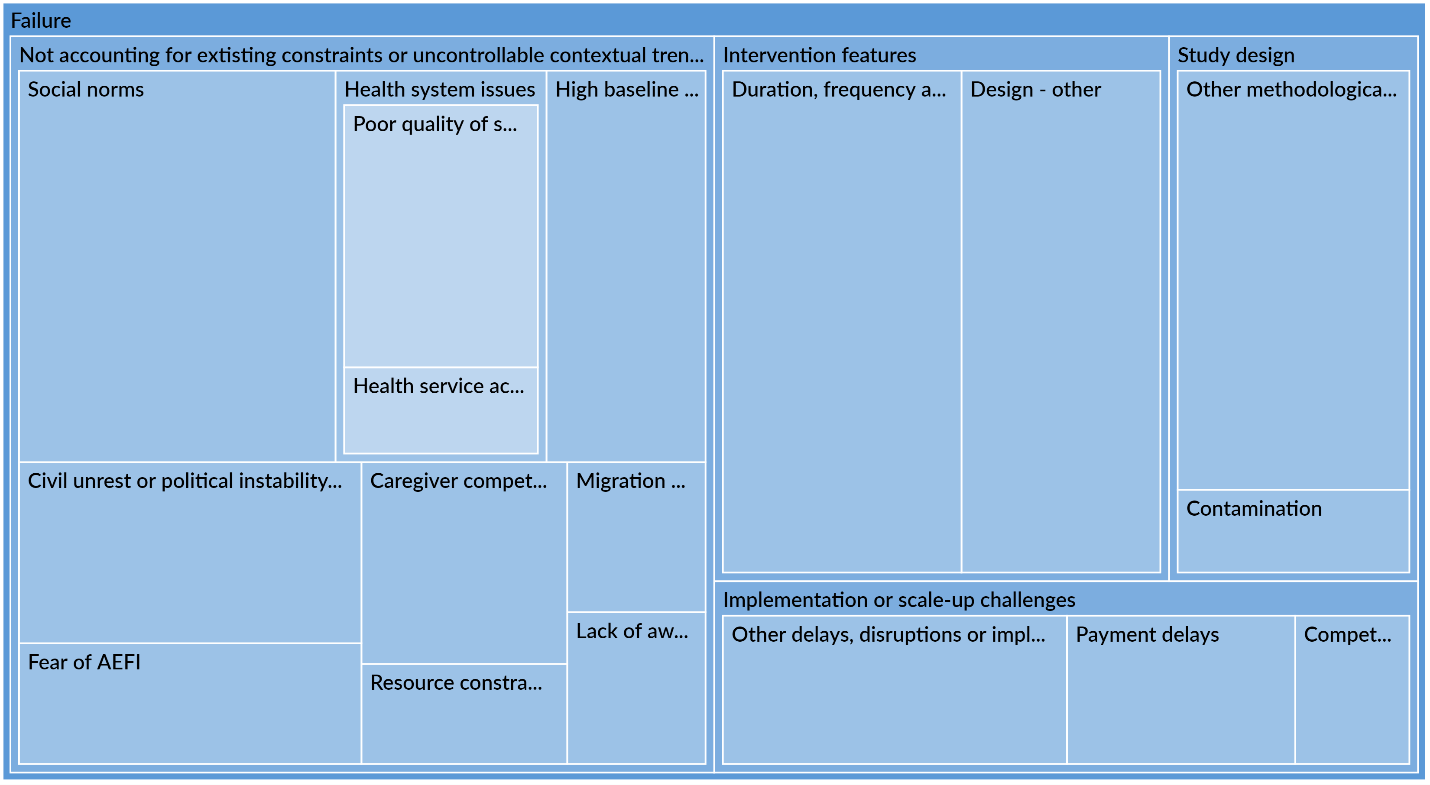


Engagement in implementation


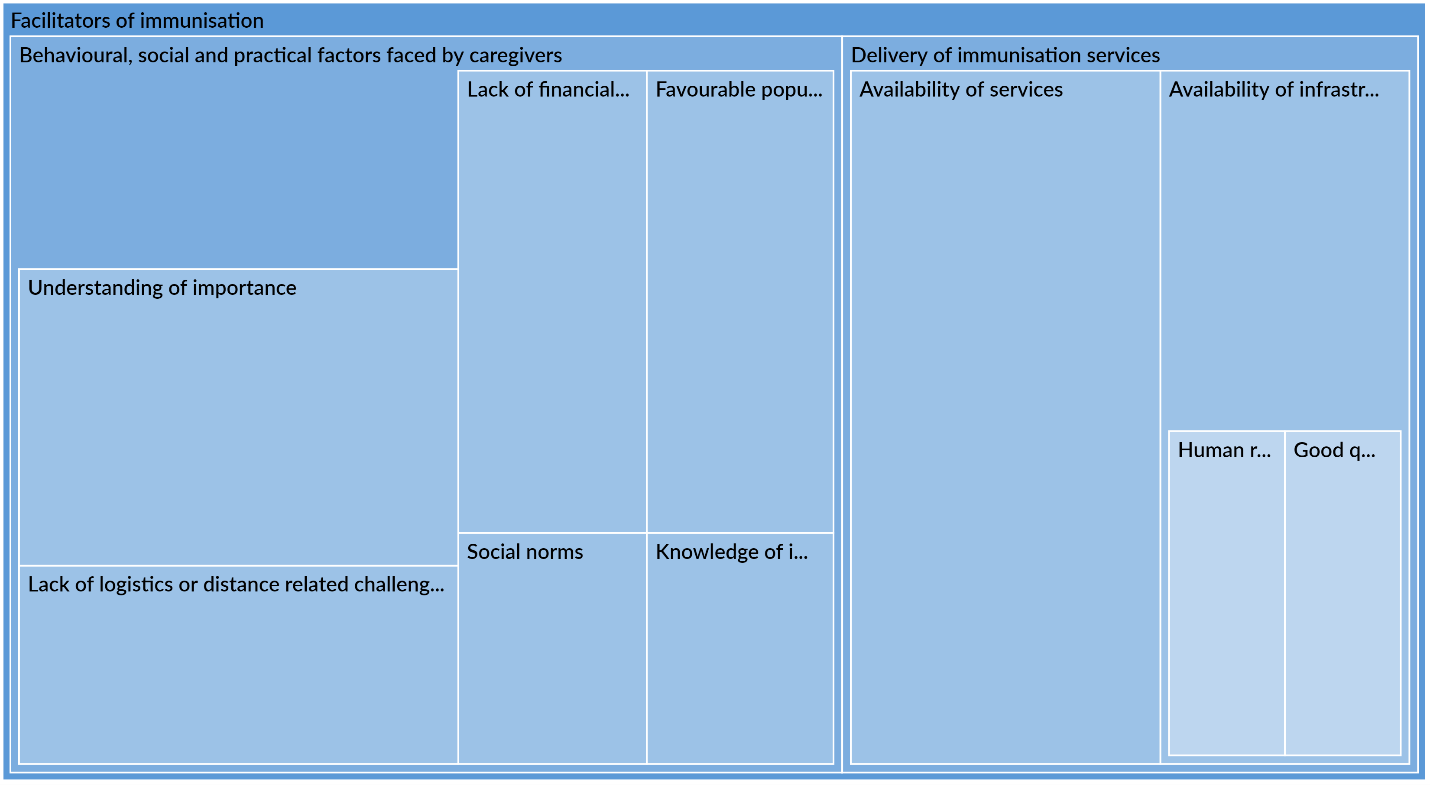


Table 7: Characteristic quotes of themes identified related to facilitators of immunisation for community engagement interventions

| **Primary paper** | **Paper from which citation comes** | **Theme demonstrated** | **Quote** | **Page number** |
| --- | --- | --- | --- | --- |
| *Understanding of importance* | | | |  |
| Adamu et al. 2019 | Adamu 2019 Dissertation | Awareness of benefits | In addition, caregiver beliefs about the capabilities of immunization was overwhelmingly strong: “… this vaccination is very important because it prevents infection from measles, cough, hepatitis, fever, pneumonia and yellow fever. | 129 |
| Oyo-Ita et al. 2020 | Oyo-Ita et al. 2020 | Positive perception of vaccination | It was found from the baseline qualitative study that respondents were generally knowledgeable about and had a positive attitude towards vaccination. They believed vaccines prevented their children from acquiring deadly infections and attributed low numbers of deaths of children to vaccination | 31 |
| *Socio-economic characteristics* | | | |  |
| Andersson et al. 2009 | Cockroft 2009 | Importance of maternal education | Mother’s education was related to measles vaccination in all four districts, in urban and rural sites. | 10 |
| *Social norms* | | | |  |
| Pramanik 2020 | Pramanik 2020 | Household decision-making | Key informants further mentioned that in rare cases, it was seen that presence of a knowledgeable daughter in law in the family compelled families to vaccinate their children | 85 |
| *Availability of infrastructure, supplies or services* | | | |  |
| Olken et al. 2014 | Rahayu 2008 | Availability of medical supplies | The midwife has comprehensive equipment. She has infusion equipment, blood pressure monitor, injections, medicines, scales for infants. The midwife also provides a room in the polindes for mothers who have just given birth. (Women’s FGD, Kuanek, East Miomaffo, TTU, NTT) | 25 |
| Andersson et al. 2009 | Cockroft 2009 | Outreach by healthcare workers | in rural sites a vaccination team visiting the community also increased the likelihood that the child had received measles vaccine; this effect was much stronger in rural Khanewal. | 5 |
| Adamu et al. 2019 | Adamu 2019 Dissertation | Availability of vaccination services | In this study, some caregivers indicated that recommended birth doses of vaccines are provided in the labor room as soon as the child is born. This practice saves time and reduces the chance of missed opportunities in this service delivery point. | 134 |

Reasons for interventions success by type of community engagement

Engagement as intervention


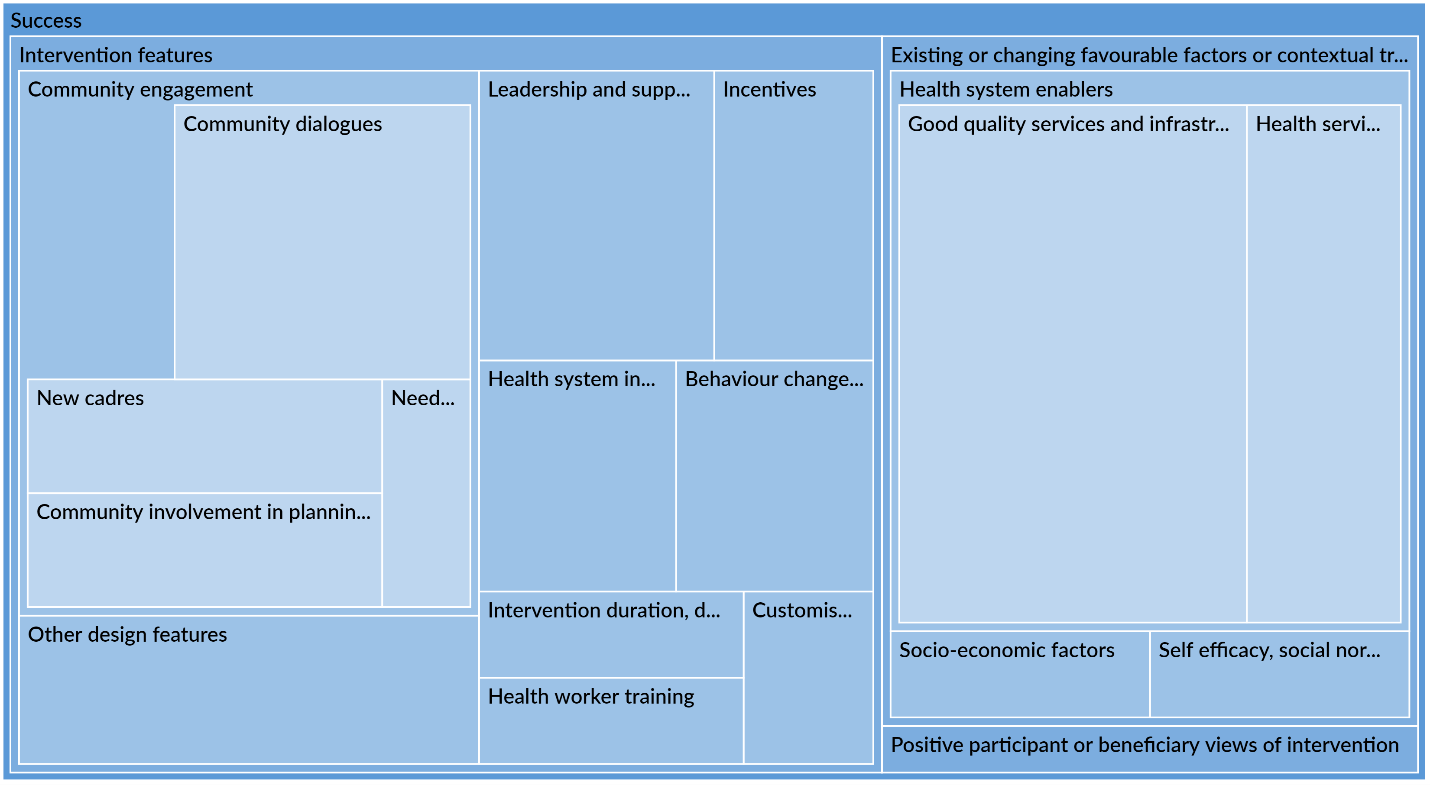


Engagement in design


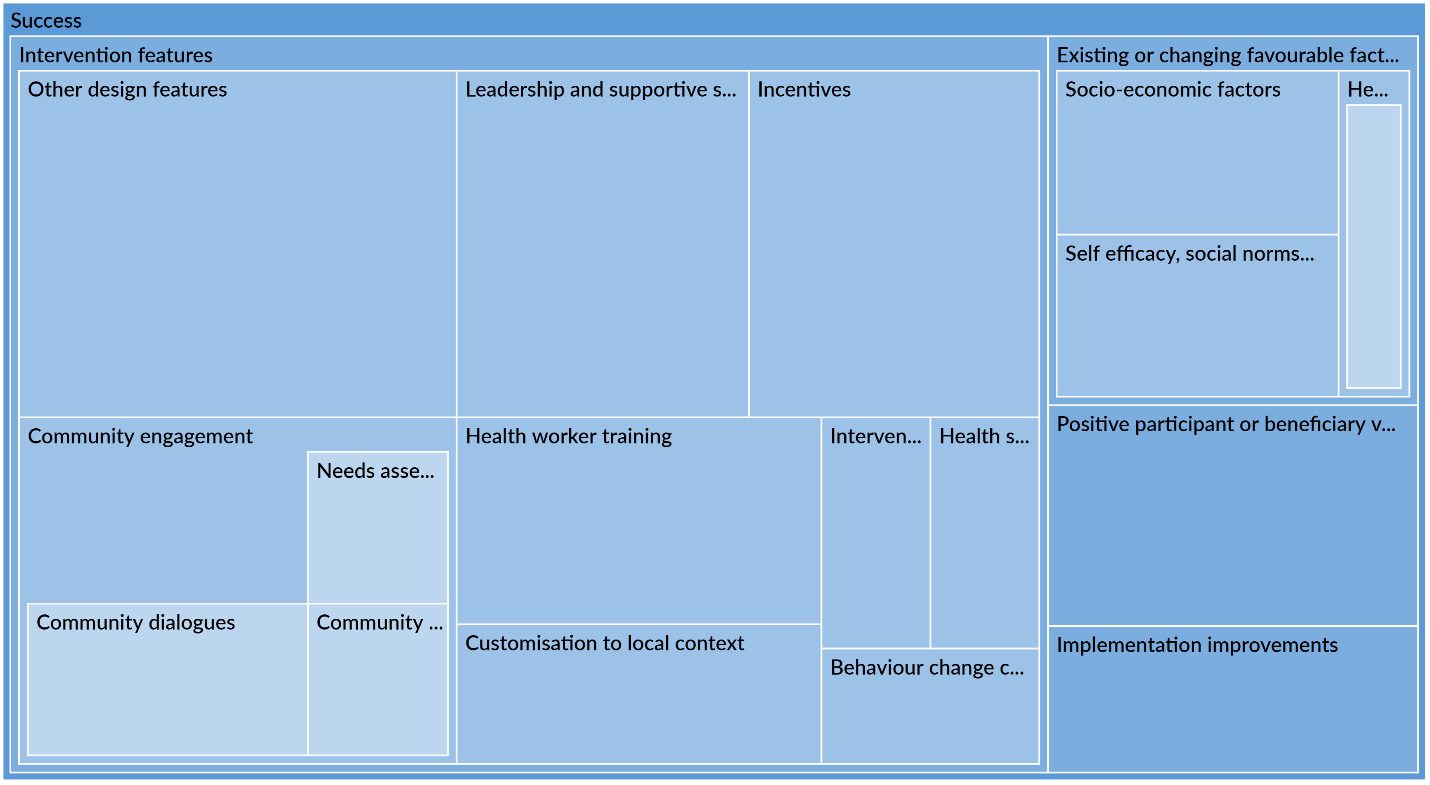


Engagement in implementation


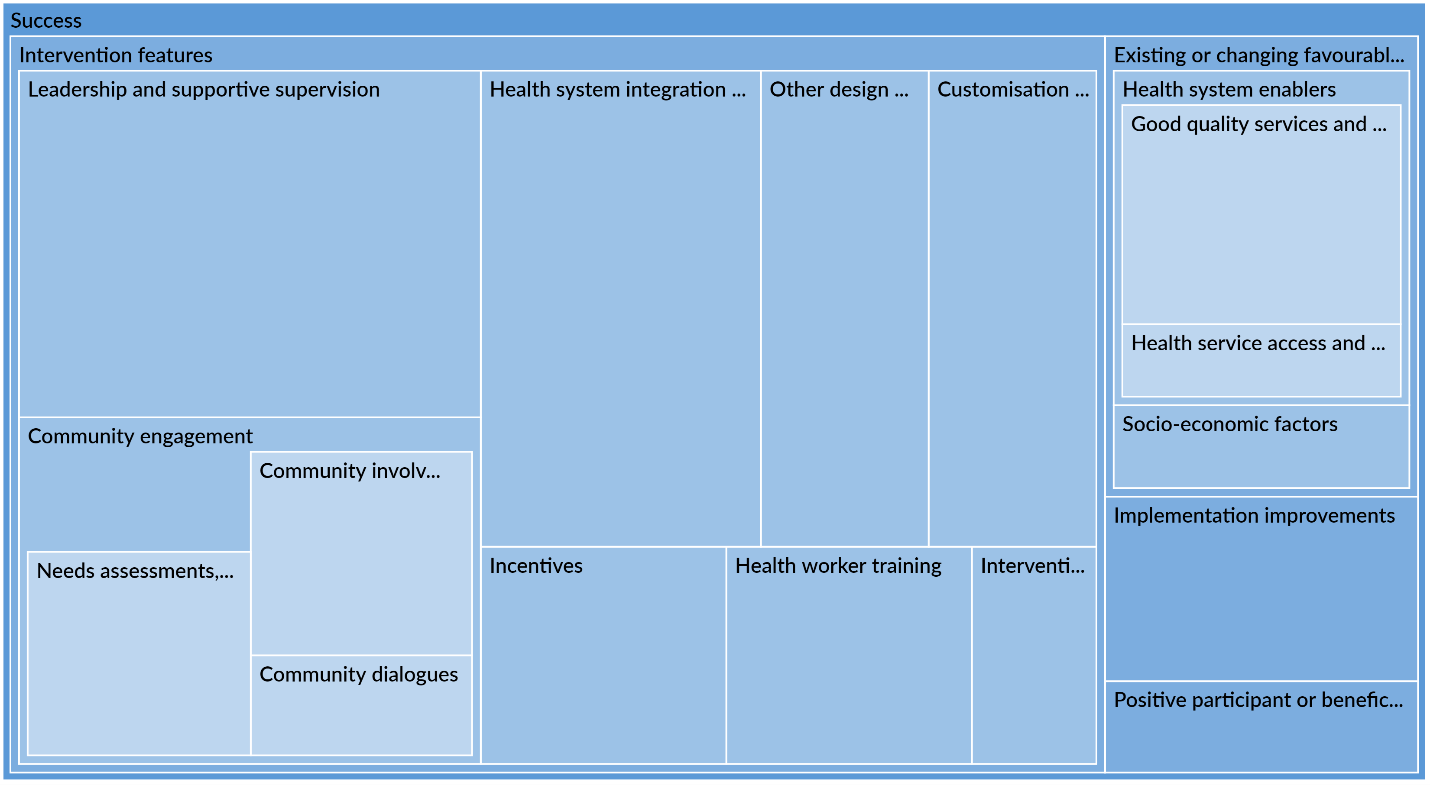


Table 8: Characteristic quotes of themes identified related to reasons for success for interventions were community engagement.

| **Primary paper** | **Paper from which citation comes** | **Sub-theme demonstrated** | **Quote** | **Page number** |
| --- | --- | --- | --- | --- |
| Intervention features | | | | |
| Assegaai 2018 | Padayachee 2013 | *Community dialogue* | Interviewees spoke of the tangible benefits of the community dialogues and proactive household approaches as expanding access, improving relationships with communities, and increasing knowledge and uptake of services. | 76 |
| Oyo-Ita 2020 | - | Community involvement in planning and implementation | Inclusion of TRLs in the planning, implementation and evaluation of an intervention is useful in ensuring support from the community. | 42 |
| Biemba 2016 | Phiri 2017 | Leadership and supportive supervision | CHAs reported that supervision by experienced health workers was valuable to them because it reinforced skills they learned in training and provided general encouragement. This study finds that the quality and frequency of supervision is heavily influenced by the proximity of supervisors’ work station to that of the CHAs. | 5 |
| Banerjee 2020 | - | Incentives | Most primary caregivers expressed positive opinions about recharges. Some admitted to finding them motivating or influencing their decision to immunise their child (whether or not they actually received them). This was linked to generally liking free things (‘What’s not to like?’), their self-assessed poverty level, or their trust in the government ‘investing in them’. | 53 |
| Existing or changing favourable factors or contextual trends | | | | |
| Rao et al. 2014 | Rao et al. 2016 | Good quality of services and infrastructure | Moreover, the ASHA worker is envisioned not to work in isolation but as an integral part of the public health system, working together with other health personnel to both ease their work-pressures and assist them in performing their duties more efﬁciently. For instance, the ASHA worker works with the ANM and the anganwadi worker to organize monthly village health days. Therefore, the extent to which the increase in information provision via other health workers is attributable to the ASHA worker is not quantiﬁable in this context. | 25 |
| Findley et al. 2013 | - | Good quality of services and infrastructure | simultaneous improvement in the quality of care provided by the CHW and nurse-midwives at the health post gave women the confidence that they could go to the health post to seek advice and care. | 10 |
| Banwat 2015 | Banwat 2014- | Health service access and availability | A child was also found to be most likely to be fully and timely immunized if he/she lived within 30 minutes walking distance from the health facility. | 66 |

Table 9: Characteristic quotes of themes identified related to reasons for success for interventions that used community engagement in their design.

| **Primary paper** | **Paper from which citation comes** | **Sub-theme demonstrated** | **Quote** | **Page number** |
| --- | --- | --- | --- | --- |
| Intervention features | | | | |
| Andersson 2009 | - | Community dialogue | The structured discussion rounds sometimes led to action plans in the intervention communities beyond stimulating discussion about vaccinations within households. Particularly in those villages with poor access to vaccination services, plans included sharing transport to vaccination points and providing care for some children while parents took others to be vaccinated. These community initiatives may have helped to maintain vaccination levels in the face of generally falling levels. | 8 |
| Adamu 2019 | Adamu 2019 CFIR | Leadership and supportive supervision | Participants expressed satisfaction with the supervisory plan that was put in place for the quality improvement program as it enabled quick feedback. Supervisory visits were conducted by different stakeholders that are higher-ranking officials within the health systems. These include local government and zonal primary health-care management board officials. “We receive supervision from local government, they use to come and supervised us to check how we conduct our duties.”– PHC 3 | 468 |
| Assegaai 2018 | Khuzwayo 2017 | Referral to caregivers | Referrals to clinics by the teams were regarded as an important aspect of bringing services closer to the communities. Respondents said that they would not have gone to the clinic had they not been referred by the WBOT, for example for deworming of children, or continuation of care for complicated cases. | 3 |
| Existing or changing favourable factors or contextual trends | | | | |
| Banerjee 2020 | - | Self efficacy, social norms and awareness | By and large the main, explicitly stated driver for primary caregivers to get their child vaccinated is their positive perception or attitude toward immunization: that immunization will benefit their child. This acts as a push even for primary caregivers who have limited knowledge, who don’t understand exactly how or why immunization is beneficial. Some of these primary caregivers hold strong personal conviction of the importance of immunization, despite their lack of knowledge, while others are undecided or unsure about the importance of immunization, but are still overall positive. In both cases, it seems that the positive perception or attitude is influenced by others: what others say and do. This is often mediated by trust: either trust in government and authority (e.g. ANM), household or family members, other primary caregivers in the community, or people in positions of power in the community (e.g. religious leaders in Mewat). Interestingly, improved knowledge and awareness was stated by a number of respondents as a key factor to convince primary caregivers (themselves, or others) to attend immunization camps. This demonstrates, as was mentioned in response to ANM performance, that primary caregivers value information about vaccines | Appendix K – qualitative findings |
| Gibson et al. 2017 | - | Socio-economic factors | The success of SMS reminders to elicit a behaviour is multifactorial; the content of the message, the type of behaviour being reminded, indirect and direct costs incurred, literacy level, and other contextual factors all being potential explanatory factors. | e436 |
| Implementation improvements | | | | |
| Modi 2019 | - | Operational improvements | Finally, we identified critical operational requirements that improved adherence to the intervention; this included supportive supervision, timely resolution of technology problems, and change management, including monetary and/ or nonmonetary incentives depending on the context. | 20 |

Table 10: Characteristic quotes of themes identified related to reasons for success for interventions that used community engagement in implementation.

| **Primary paper** | **Paper from which citation comes** | **Sub-theme demonstrated** | **Quote** | **Page number** |
| --- | --- | --- | --- | --- |
| Intervention features | | | | |
| Adamu 2019 | - | Community involvement in planning and implementation | Stakeholders were systematically involved in the planning and execution of the QI program. The change ideas were selected by frontline health workers and they tailored them to their local context. Also, change ideas are multi-faceted and multimodal, and this is suitable for addressing complex problems in complex health systems. | 9 |
| Sankar 2013 | - | Needs assessments, pilots or stakeholder consultations | The project interventions were developed in keeping with the especial needs of the population, and rooted in the conditions and circumstances of the area. It drew heavily from the understanding and opinions of local people. The results reflect the fruits of a highly contextualised program. | 40 |
| Shukla 2018 | Anwari 2015 | Leadership and supportive supervision | District Health Offices and DHCCs were less well established compared to the Provincial Public Health Directorates and PPHCCs; the ministry did not have adequate resources to equip them well. Despite these challenges, the leadership and involvement of the ministry in the intervention mattered. The provincial and district health governance leaders were inspired to improve their governance because the ministry leaders were interested in the pilot intervention. | 14 |
| Existing or changing favourable factors or contextual trends | | | | |
| Admassie 2009 | - | Good quality services and infrastructure | The impact of the programme on the proportion of children who got vaccines against  major childhood illnesses also varies according to supply side variables (see Table 6). The programme has larger effects in villages with one HEW and with better quality health posts. | 441 |
| Herrera-Almanza and Rosales-Rueda 2018 | - | Health service access and availability | However, the fact that we find results on vaccination uptake is an indication that the health workers were somehow present in remote areas. | 29 |
| Admassie 2009 | - | Socio-economic factors | This implies that encouraging and supporting primary schooling for girls and women will enhance the programme’s impact on proportion of children vaccinated against major childhood illnesses. | 441 |
| Implementation improvements | | | | |
| Mayumana 2017 | Binyaruka 2017 | Managing vaccine stockouts | The veriﬁcation system under P4P also meant that district supervision was intensiﬁed, providing more opportunities for district managers to identify and address stock-outs of a wider range of drugs. | 97 |

**Reasons for intervention failure by type of engagement**

Engagement as intervention

**
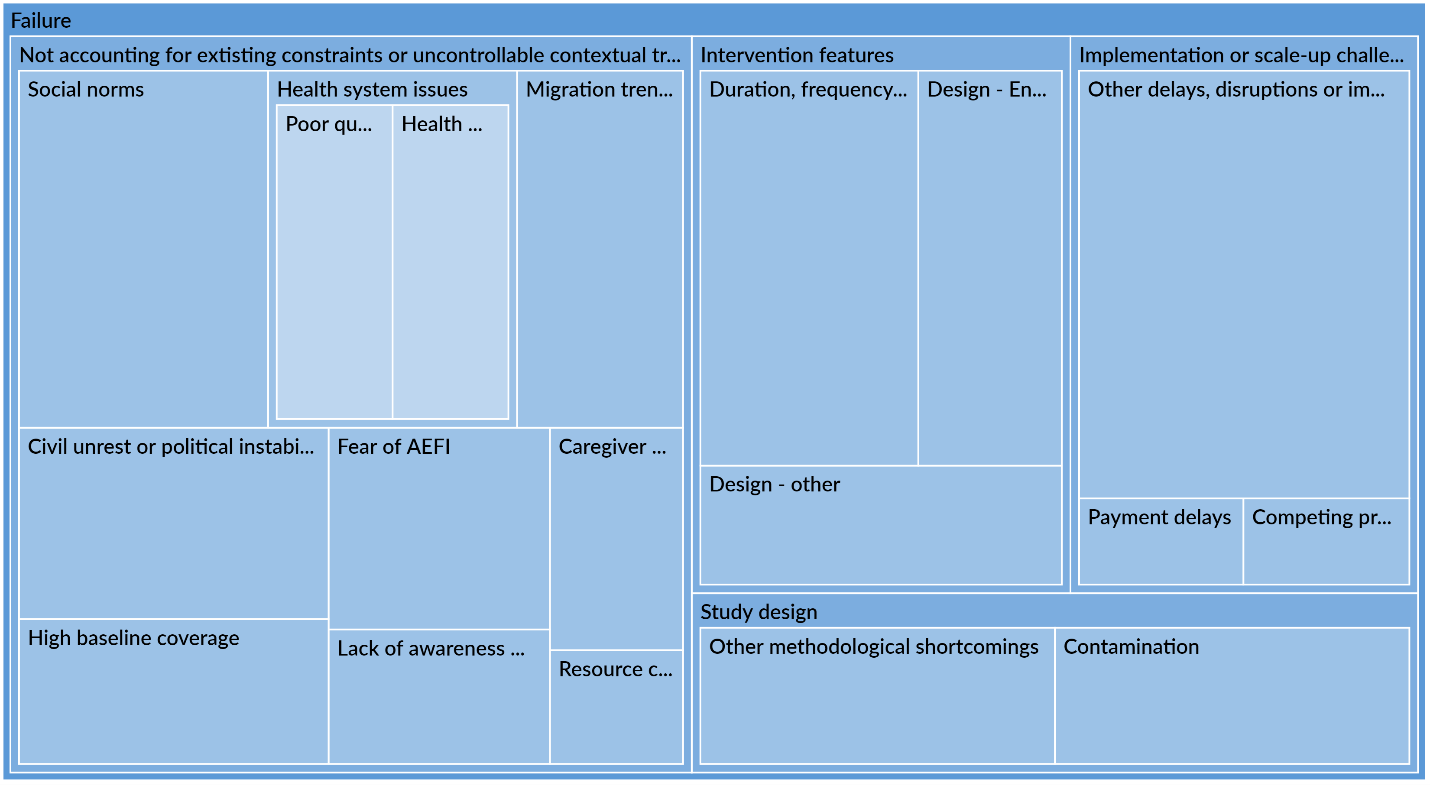
**

Engagement in design


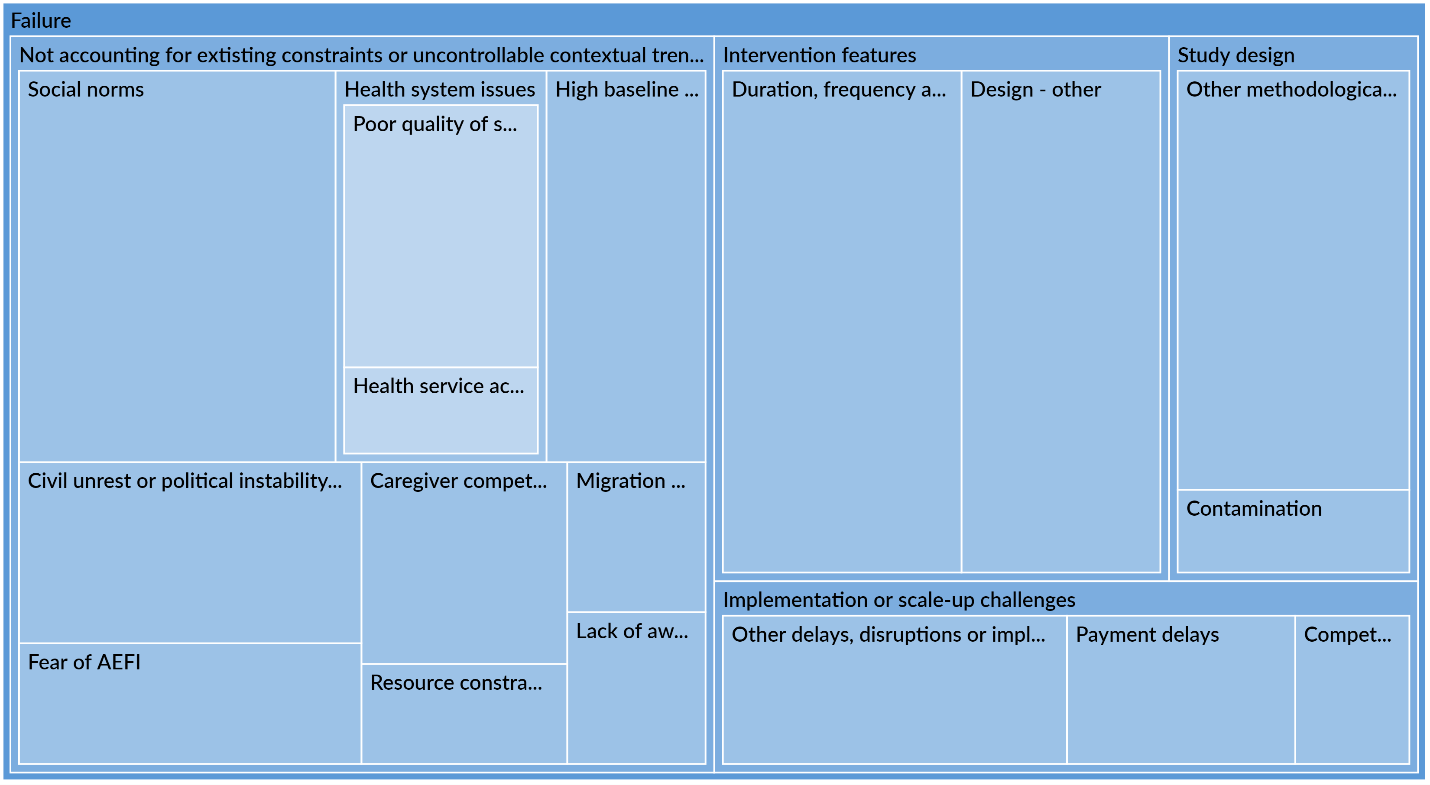


Engagement in implementation


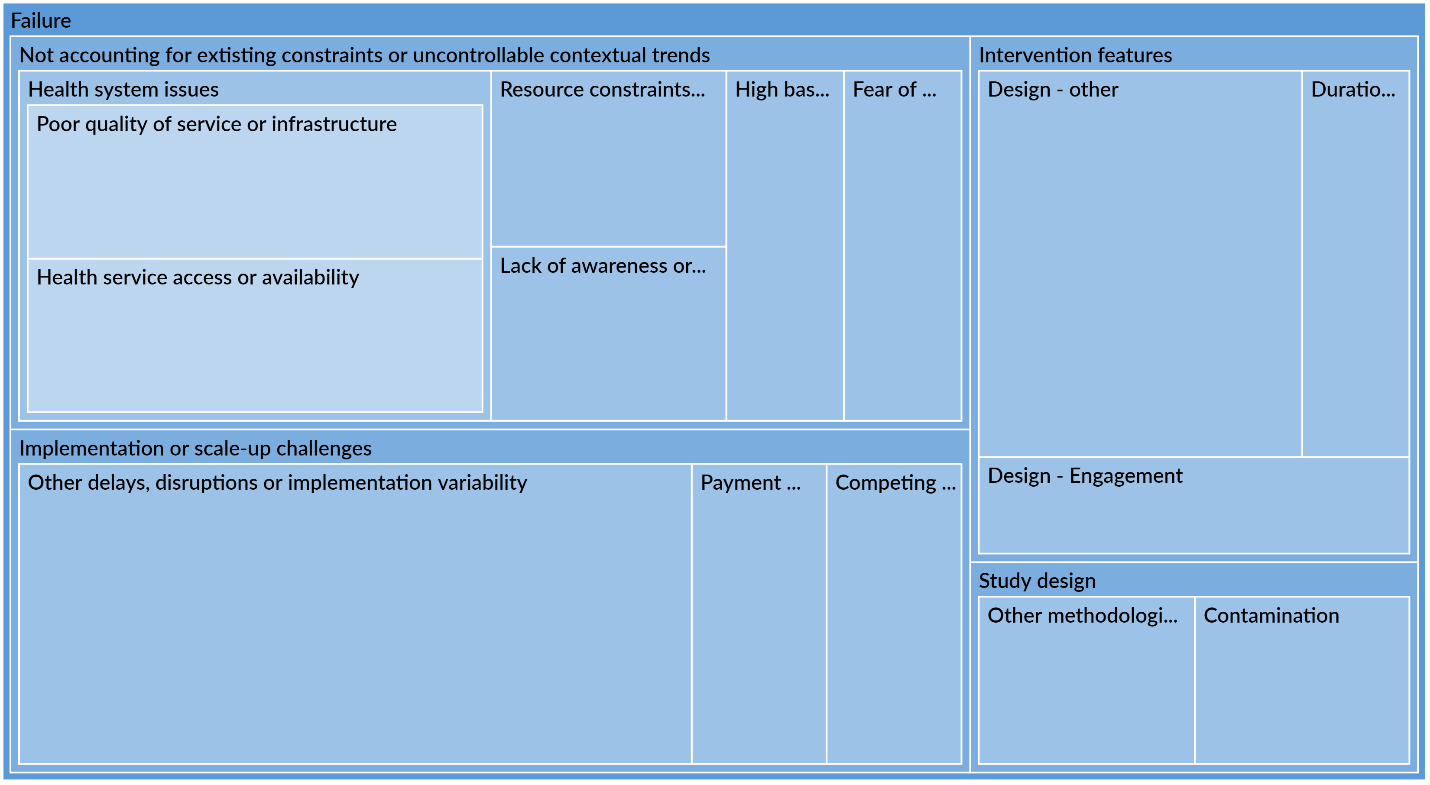


Table 11: Characteristic quotes of themes identified related to reasons for failure for interventions were community engagement.

| **Primary paper** | **Paper from which citation comes** | **Sub-theme demonstrated** | **Quote** | **Page number** |
| --- | --- | --- | --- | --- |
| *Implementation or scale-up challenges* | | | | |
| Carnell 2014 | - | Attrition of implementation personnel | The ESHE and RHB teams both faced personnel challenges in Amhara, which may have affected management and performance. | 559 |
| Gurley 2020 | - | Infrastructure constraints | Community constraints around screening spaces and economic activities also introduced challenges. | 73 |
| *Not accounting for existing constraints or uncontrollable trends* | | | | |
| More 2012 | - | Lack of access to health care | The third issue was the complexity of urban health care. Antenatal care was the norm and the nadir for institutional delivery in trial clusters was 75%. Around 57% of antenatal care and 30% of deliveries were in the private sector (this in a slumdwelling population). Open access to private providers, and to institutions at all levels of the public sector hierarchy, is a challenge to systematic health care delivery. Our findings confirmed the tendency to bypass public maternity homes, which should handle uncomplicated deliveries, in favour of tertiary institutions. | e1001257 |
| Pramanik 2020 | - | Natural calamity | Assam has a wonderful collection of festivals that are of the utmost importance. And more tragically when the Brahmaputra River floods the priority is survival. The project simply has to adapt to these ebbs and flows, but there is a pressure to deliver to a schedule that inevitably leads to a box ticking approach that is inconsistent with the development of ownership by the community. | 68 |
| Oyo-Ita 2020 | - | Fear of AEFI | These responses are indications that fear of side effects can hinder vaccine uptake. This may have contributed to non-impact on the proportion of fully vaccinated children in this study, as the TRLs in the post-intervention qualitative study still mentioned this as a common reason for poor uptake of vaccines. | 32 |
| *Intervention features* | | | | |
| Olken 2014 | Grayman 2013 | Engagement failure due to elite capture | In general, researchers discovered that Generasi provides opportunities for elites to retain and fortify their social standing. As brokers delivering CDD resources into their communities, local elites leverage Generasi to accumulate additional status for themselves. Without exception, every Generasi actor that the researchers met held other leadership roles in their community and maintained close ties with the local structures of village and sub-district governance. | 48 |
| Robertson 2013 | - | Inadequate exposure to the intervention | Our study was limited by the short intervention period. Whether the effects of the programmes would change with time is unclear. The follow-up survey was done 2 months after interventions had finished, so fear of penalties should not have biased responses from CCT households, although the effects of the programmes could have attenuated by the time of the survey. | 8 |
| *Study design* | | | | |
| More 2017 | - | Contamination due to other programmes | although we found no evidence of contamination of control clusters by the intervention, government schemes and the activities of municipal and non-governmental providers might have improved health in control clusters. | e347 |

Table 12: Characteristic quotes of themes identified related to reasons for failure for interventions that used community engagement in their design.

| **Primary paper** | **Paper from which citation comes** | **Sub-theme demonstrated** | **Quote** | **Page number** |
| --- | --- | --- | --- | --- |
| *Implementation or scale-up challenges* | | | | |
| Gurley 2020 | - | Staff turnover | On the implementer side, staff turnover at NYST and research activities introduced delays in video production. | 73 |
| Engineer 2016 | - | Payment delays | Problems with implementation likely dampened any potential effect. The scheme was rolled out in phases, but there were some delays, particularly with the initial payments. | 456 |
| *Not accounting for existing constraints or uncontrollable trends* | | | | |
| Gibson 2017 | - | High baseline immunisation coverage | SMS reminders were probably not effective at improving full immunisation and vaccine-specific coverages in this study because of high baseline coverage levels and because SMS reminders might not have addressed the demand side deficiencies in this study area. | e436 |
| Banerjee 2020 | - | Caregiver competing priorities | Across districts, there are ‘hard-to-convince’ populations who are not affected by incentives. Some ANMs listed specific populations groups they felt continued to be unaffected by the programme, and the incentives specifically, such as migrant workers, daily wage workers and the Muslim community. In Panipat, the top reason given by ANMs for resistance to immunisation by the few still unconvinced (migrants, Muslims) was the fever brought on as an after-effect by a certain vaccine (penta)30 and direct costs involved for daily wage workers. | 92 |
| Gurley 2020 | - | Social norms | It is worth noting that in contrast to the high intent recorded through the household survey, the process evaluation indicated there were individuals who actively chose to not vaccinate their children. This may be a function of social desirability bias in the household survey, though we posit that it may also reflect family constraints or logistical barriers that prevent a mother from taking a vaccine decision regardless of own personal intention to vaccinate. As noted elsewhere, mothers are not the sole or primary decision-makers and other family members reported lower intent to vaccinate. | 75 |
| *Intervention features* | | | | |
| Siddiqi 2020 | - | Inadequate exposure to the intervention | Our findings also provide evidence of the strong reliance on the immunization card being the established immunization recall method and we may also postulate that the short duration of the study did not provide enough time to ‘institutionalize’ the use of the bracelets. | 12 |
| Okoli 2014 | Baba-Ari 2018 | Communication failure | Experiences of the beneficiaries with the programme point to problems with programme communication as they had a poor understanding of the aims of the CCT programme aside from receiving its immediate cash benefits and as such might revert to their original behaviours after the programme ends. | 940 |
| *Study design* | | | | |
| Domek 2019 | - | Selection bias in study enrollment | This may have been partly because our study population had higher baseline immunization coverage than we were adequately powered to assess. These higher than expected completion rates were likely due in part to a selection bias in enrolling children as they presented for their ﬁrst immunization visit, which would have unintentionally excluded children who either presented signiﬁcantly delayed or not at all for vaccines. | 6197 |

Table 13: Characteristic quotes of themes identified related to reasons for failure for interventions that used community engagement in implementation.

| **Primary paper** | **Paper from which citation comes** | **Sub-theme demonstrated** | **Quote** | **Page number** |
| --- | --- | --- | --- | --- |
| *Implementation or scale-up challenges* | | | | |
| Oyo-Ita 2020 | - | Low implementation fidelity | The non-impact on up-to-date vaccination could have been accounted for by the weak link in the intervention caused by not sharing data directly with community members as planned. | 38 |
| Olken 2014 | World Bank 2018 | Staff turnover | High village-level staff turnover may partially explain why Generasi staff in the villages are so unfamiliar with the bonus system. The entire Generasi team in Desa Lelaok (Petis subdistrict, Pamekasan district) and the PK in Desa Rampe (Nelle subdistrict, Lembata) had never heard of the bonus system, but all of them had started within the past year. | 38 |
| Webster 2019 | - | Competing priorities of health workers | Although the proportion of respondents reporting a home visit by a VHT decreased from baseline to endline in both control and intervention clusters the proportion of visits where immunization was the reason for the visit increased. The decrease in VHT home visits may be due in part to observations that in the latter months of the intervention, VHT services were sought by other organisations involved in the refugee response in some of the project areas, who paid VHTs higher allowances. | 47 |
| *Not accounting for existing constraints or uncontrollable trends* | | | | |
| Okeke 2017 | - | Resource constraints or scarcity | Lack of electricity and water were also frequently cited as problems. | 43 |
| Okeke 2017 | - | Poor quality of services or infrastructure | The data suggest that part of the reason why the program did not have larger impacts is that other dimensions of quality did not improve. For example, clinic infrastructure in many cases remained poor, as did availability of drugs and supplies. | 49 |
| *Intervention features* | | | | |
| Webster 2019 | - | Intervention design failure | The main mechanisms of change identified were improved accessibility to immunization services through increased numbers of vaccination outreaches, increased VHT motivation through monthly allowances together with social motivation in outdoing each other in defaulter tracing, use of community resources to achieve outcomes that is the support of local community leadership; and increased interaction between health workers and HCWs and VHTs at monthly VHT/HCW meetings. These identified mechanisms of change were predominantly due to supplementary activities that were implemented in both intervention and control clusters. | 49 |
| *Study design* | | | | |
| Webster 2019 | - | Contamination between treatment and control | There was some contamination of control clusters, largely due to transfer of health workers from intervention to control sites. At two control sites it was evident that the health workers sought to set up a similar defaulter tracing approach. | 49 |

Uptake and fidelity challenges

Engagement as intervention


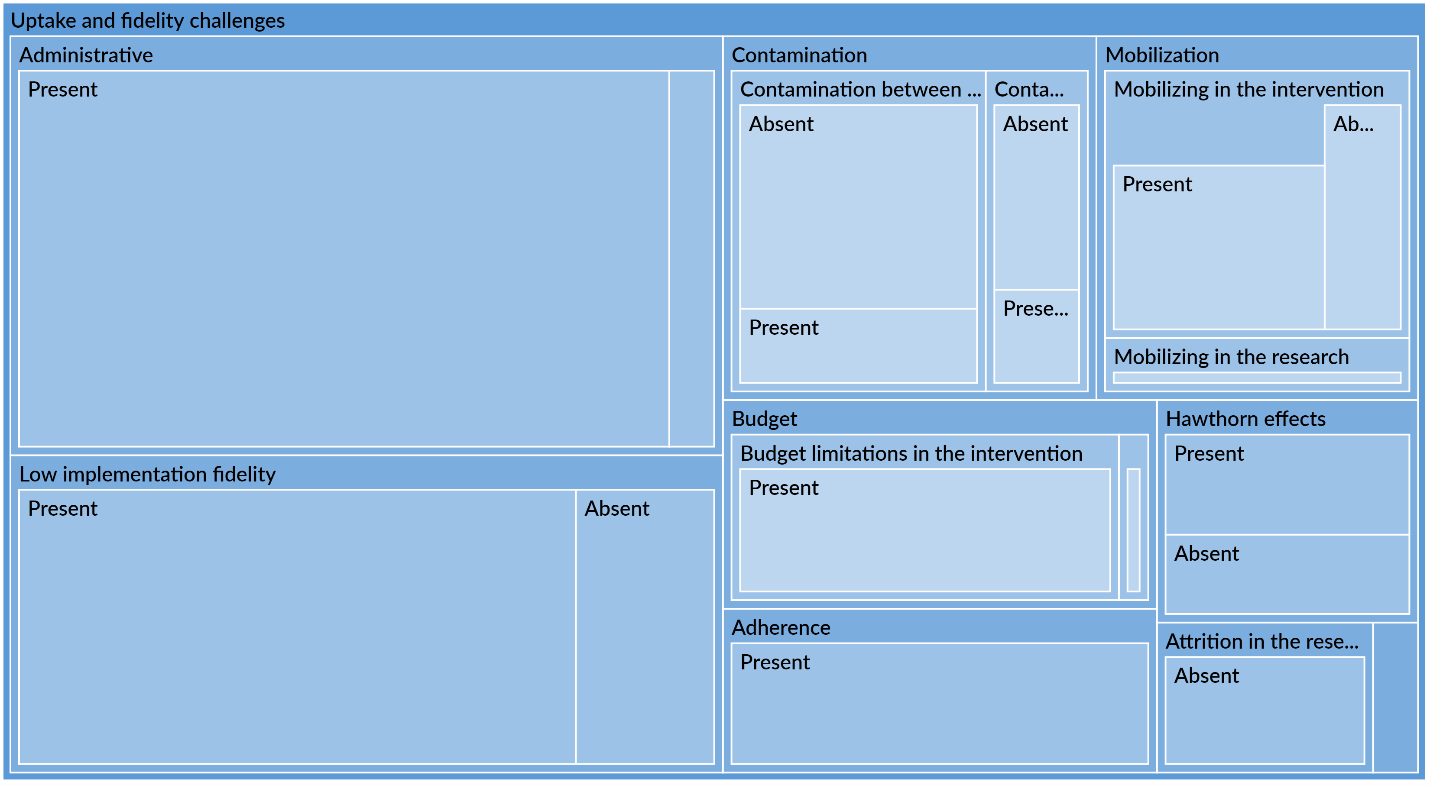


Engagement in design


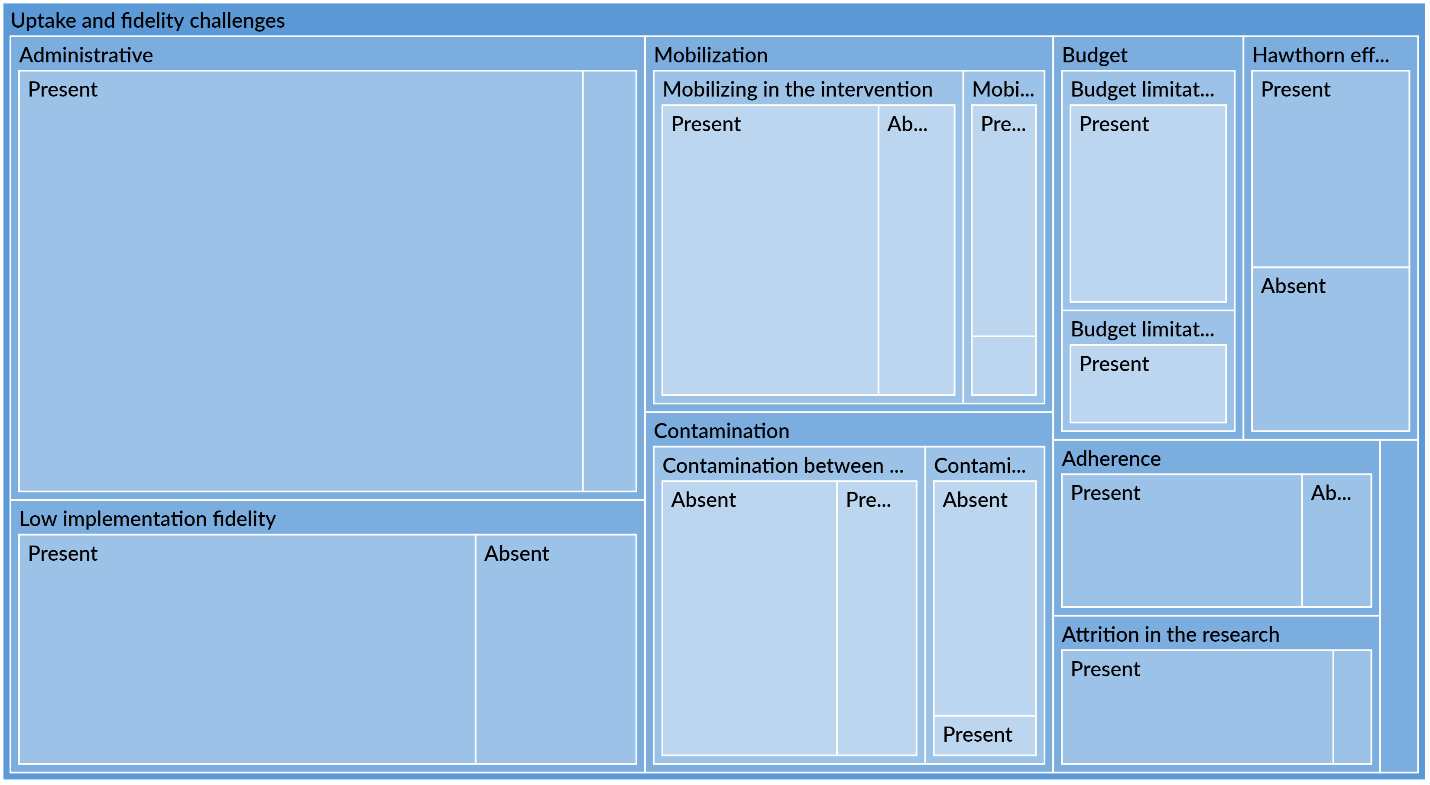


Engagement in implementation


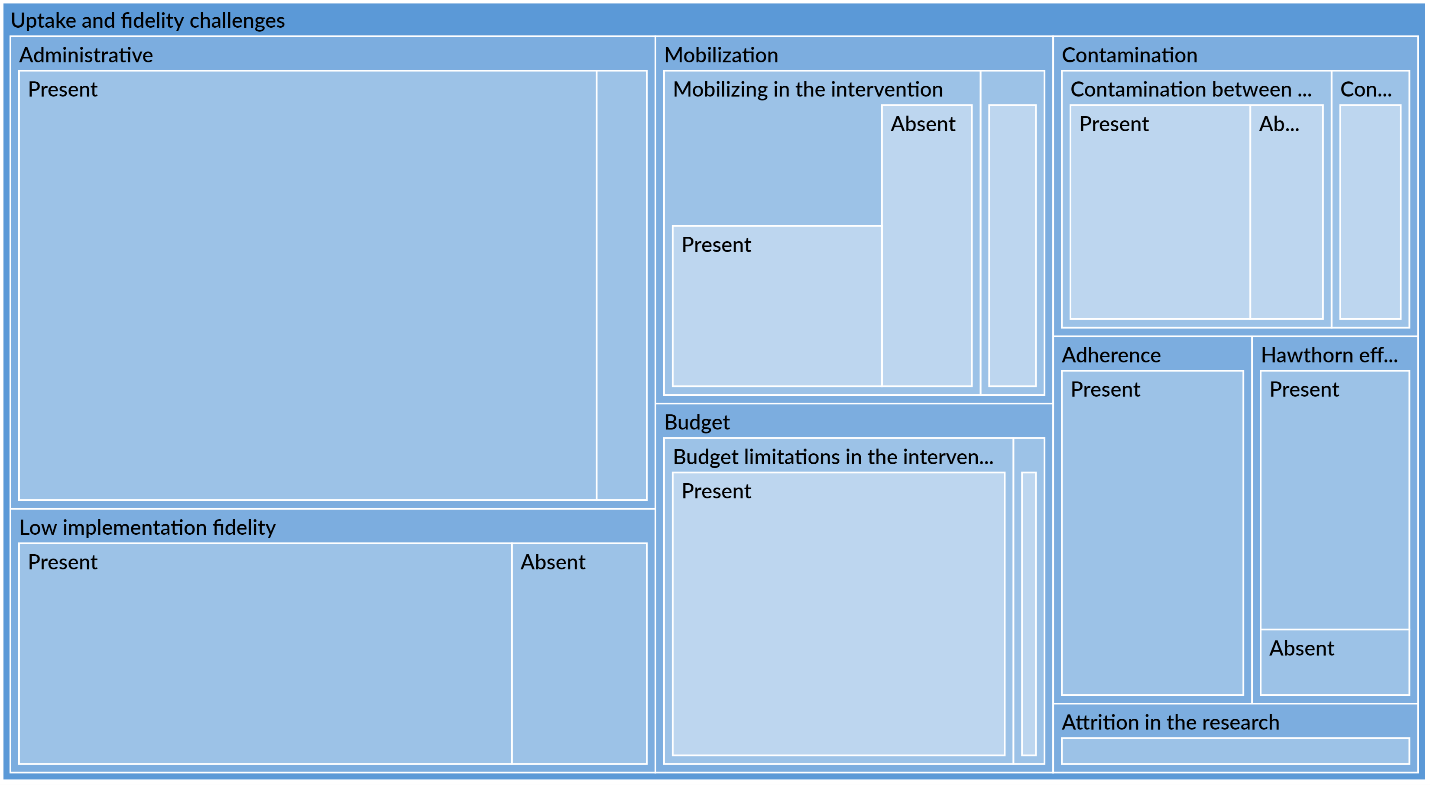


Table 14: Characteristic quotes of themes identified related to uptake and fidelity challenges for interventions were community engagement.

| **Primary paper** | **Paper from which citation comes** | **Sub-theme demonstrated** | **Quote** | **Page number** |
| --- | --- | --- | --- | --- |
| *Administrative challenges** | |  |  |  |
| Assengai et al 2018 | Khuzwayo et al 2017 | Coordination challenges | Respondents report a lack of communication about visit scheduling. | 3 |
|  |  |  | This is compounded by dual reporting lines in many parts of the country, where CHWs remain linked to and receive stipends through NGO intermediaries, while being accountable to PHC facility managers. |  |
| Demilew et al 2019 | Demilew et al 2020 | Technical and political challenges | The Mobile Intervention was left from being implemented because of Internet service inaccessibility that resulted from the state of emergency declared by the Ethiopian government. | 23 |
| Banerjee et al 2020 | Banerjee et al 2020 | Political challenges | This study has faced various degrees of support from our primary government stakeholder since its inception, and this is the main reason why the timeline has been extended multiple times over the past seven years (when discussions with the Haryana government first began). We faced issues in continuity and buy-in especially when higher-ranking officers were transferred. | 69 |
|  |  | Absence of administrative barriers | Given that J-PAL South Asia was also responsible for implementation, we recruited a dedicated team with relevant experience to do this. As a result, we were acutely aware of what was happening in the field, and able to identify and resolve issues quickly. | 71 |
| *Implementation fidelity* | |  |  |  |
| Biemba et al 2016 | Phiri et al 2017 | Inadequate supervision | Supervision and mentorship is crucial to optimizing the skills of CHAs, but this study outlines that supervision is not always implemented as intended and that in the absence of regular supervision | 7 |
| Morris et al 2004 | Morris et al 2004 | Expectations did not match realities on the ground | The service-level package was not implemented in accordance with protocol because no legal means could be identiﬁed of transferring resources | 2034 |
| Demilew et al 2019 | Demilew et al 2020 | Administrative challenges caused implementation challenges | This intervention was designed on the expectation that the text message portion could be rolled out in tandem with the ECIIN, an immunisation tracking program that planned to have HEWs report immunisation dates, mother’s data, child’s data, using text messages. Due to data issues and conflict in the region ECIIN was discontinued. | 34 |
| Gurley et al 2020 | Gurley et al 2020 | High fidelity | The types of screenings were conducted as planned, with 446 mothers group screenings, 445 VHND screenings, 223 men’s screenings, and 444 HTR screenings (Table 6). Within a single village, this translated to an average of 12 sessions held in mothers groups, 12 held in VHNDs, 12 held in HTR areas, and 6 held in men’s groups. | 23 |
| *Contamination* | |  |  |  |
| Moore et al 2017 | Moore et al 2017 | Absence of contamination | Our intervention and control groups were generally similar, with high coverage and ﬁdelity to planned activities and negligible contamination. | e347 |
| Paraminik et al 2018 | Paraminik et al 2020 | Distance reduced contamination | To mitigate potential contamination between intervention and control villages, we attempted to ensure that the intervention and control villages are sufficiently far apart from one another. | 25 |

*Challenges related to mobilisation are not presented because this information largely came from a single paper making the identification of characteristic issues *across* papers impossible.

Table 15: Characteristic quotes of themes identified related to uptake and fidelity challenges for interventions that used community engagement in their design

| **Primary paper** | **Paper from which citation comes** | **Sub-theme demonstrated** | **Quote** | **Page number** |
| --- | --- | --- | --- | --- |
| *Administrative challenges** | |  |  |  |
| Nagar et al 2020 | Nagar et al 2020 | Technological problems | The most prevalent issues reported by ANMs using the KB App included having to reenter data after an app crash (60.9%), having issues with scanning the pendant (65.2%), and issues with the application crashing (56.5%), and time required to enter data (33.3%). | 138 |
| Modi et al 2019 | Modi et al 2015 | Technological problems | Limited phone memory occasionally created technology-related issues, with the increasing requirement to store data on the mobile phone. | 10 |
|  |  | Absence of technological problems | The failure rate, defined as the proportion of forms that failed to get synced with the server, was <1%. | 266 |
| *Implementation fidelity* | |  |  |  |
| Gruley et al 2020 | Gruley et al 2020 | Reason changes were implemented | Last, due to resource constraints, the project was not able to implement the intervention component for sharing videos from phone to phone using Bluetooth technology. | 22 |
| Engineer et al 2019 | Engineer et al 2016 | Implications of low fidelity for the research | Problems with implementation likely dampened any potential effect. The scheme was rolled out in phases, but there were some delays, particularly with the initial payments. | 456 |
| Banerjee et al 2010 | Banerjee et al 2010 | High implementation fidelity | Review of records showed that of 1336 planned camps, 95% (1269) took place. | 2 |
| *Contamination* | |  |  |  |
| Borkum et al 2014 | Borkum et al 2014 | Absence of contamination with other interventions | Because FLWs and households in the treatment and control groups received non-TBGI Ananya program interventions that were being implemented simultaneously across Begusarai, the RCT was designed to measure the value-added of TBGI beyond these other Ananya interventions. It was not designed to measure the impact of TBGI introduced in isolation. | 6 |
| Nagar et al 2018 | Nagar et al 2016 | Steps to avoid contamination | a cluster randomised approach further allowed non-contamination of social signaling interventions within the contacts of a given village | 34 |
| Johri et al 2018 | Pereze et al 2020 | Little contamination observed | According to the analysis of the records, only people who belonged to the intervention group attended the community meetings. The people who communicated with or received calls from the IVR platform were from the intervention group, except for one (1/166) control group member. | 15 |

*Challenges related to mobilisation are not presented because this information largely came from a single paper making the identification of characteristic issues *across* papers impossible.

Table 16: Characteristic quotes of themes identified related to uptake and fidelity challenges for interventions that used community engagement in implementing the intervention.

| **Primary paper** | **Paper from which citation comes** | **Sub-theme demonstrated** | **Quote** | **Page number** |
| --- | --- | --- | --- | --- |
| Administrative challenges* | |  |  |  |
| Olken 2014 | Febriany et al 2011 | Delays in funding | The most frequent complaint by FDs regarding barriers to program implementation was the delays in aid disbursement. | 14 |
| Mayumana et al 2017 | Chimbutu et al 2011 | Stakeholder communication problem | The NTPI‟s programme, however, did not start in 2007 as planned… The major reason for this is on the implementation challenges between the two partners. The MOHSW preferred an accelerated approach while NORAD preferred an implementation framework to be designed by the Ifakara Health Institute (IHI). | 16 |
| Oyo-Ita 2020 | Oyo-Ita 2020 | Staffing and bureaucracy challenges | Low staffing of facilities constrained health staff from deploying the defaulters’ register. Some complained that they had many registers other than the defaulters’ register to fill. | 18 |
| *Implementation fidelity* | |  |  |  |
| Oyo-Ita 2020 | Oyo-Ita 2020 | Inconsistent implementation | The community engagement did not use town hall meetings as planned. Only one town hall meeting was held. | 18 |
|  |  |  | The TRL training intervention was carried out as planned. | 17 |
| Okeke et al 2017 | Okeke et al 2017 | Expectations did not match realities on the ground | Contrary to the role of the states outlined in the MOU, policymakers in Enugu state reported playing no role in the implementation of the scheme. One suggested that the division of responsibility drawn up in the MOU did not align with the setup of health care in Nigeria given that the state government has responsibility for secondary care only. | 50 |
| Mayumana et al 2017 | Olfasdottir et al 2014 | Intervention did not function as expected | Firstly, the work load on staff is considerably higher than they are contracted for, which is not reflected in the remuneration; payments are delayed and overtime and eligible allowances are not always paid causing demotivation among staff. | 7 |
|  |  | Intervention was implemented according to clear standards | The direct and transparent payment of funds, as well as more frequent contact with their managers was reported to enhance trust and improve the relationship between health workers and their managers. | 64 |
| *Budgetary constraints* | |  |  |  |
| Okeke et al 2017 | Okeke et al 2017 | Pay was severely delayed | Some local government don’t have the money to pay and they have not paid for a period of time. Most of them [midwives] have even finished their service and gone out without receiving any penny from the local government. | 41 |
| Mayumana et al 2017 | Mayumana et al 2017 | Activities were suspended | According to the qualitative data one of the obstacles for providing more frequent supervision visits was a lack of financial resources:  We do supervision every quarter. Our plan is to do supervision monthly but due to limited budget we haven’t yet done this. | 6 |

*Challenges related to mobilization are not presented because this information came from a single paper making the identification of characteristic issues *across* papers impossible.

## Appendix K: Sensitivity analysis for qualitative evidence

We carried out a sensitivity analysis in which we considered only the 17 qualitative studies associated with 12 impact evaluations that had a risk of bias assessment score of 20 or higher. Broadly, the themes that emerged from this analysis were consistent with those of the full analysis. We did not have enough qualitative evidence to conduct a sensitivity analysis by engagement type. An important caveat of this analysis is that nearly half of the papers rated as high “quality” were associated with three impact evaluations. The panel figures below provide comparison of the emerging themes in the full analysis versus the sensitivity analysis by comparing hierarchy charts related to barriers, facilitators, reasons for project success or failure, and uptake and fidelity challenges.

Barriers to immunisation


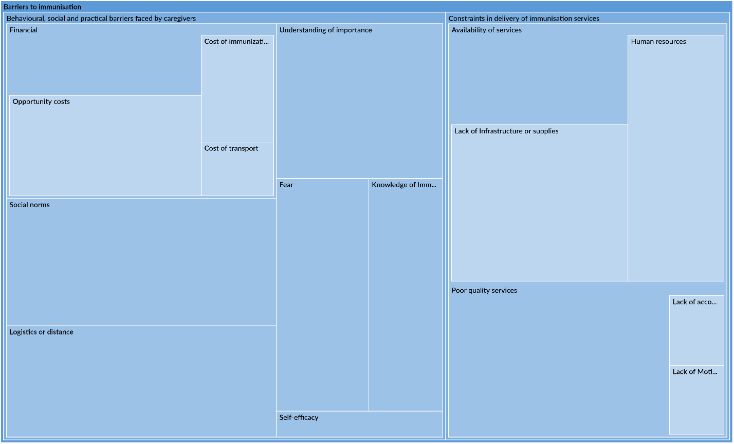

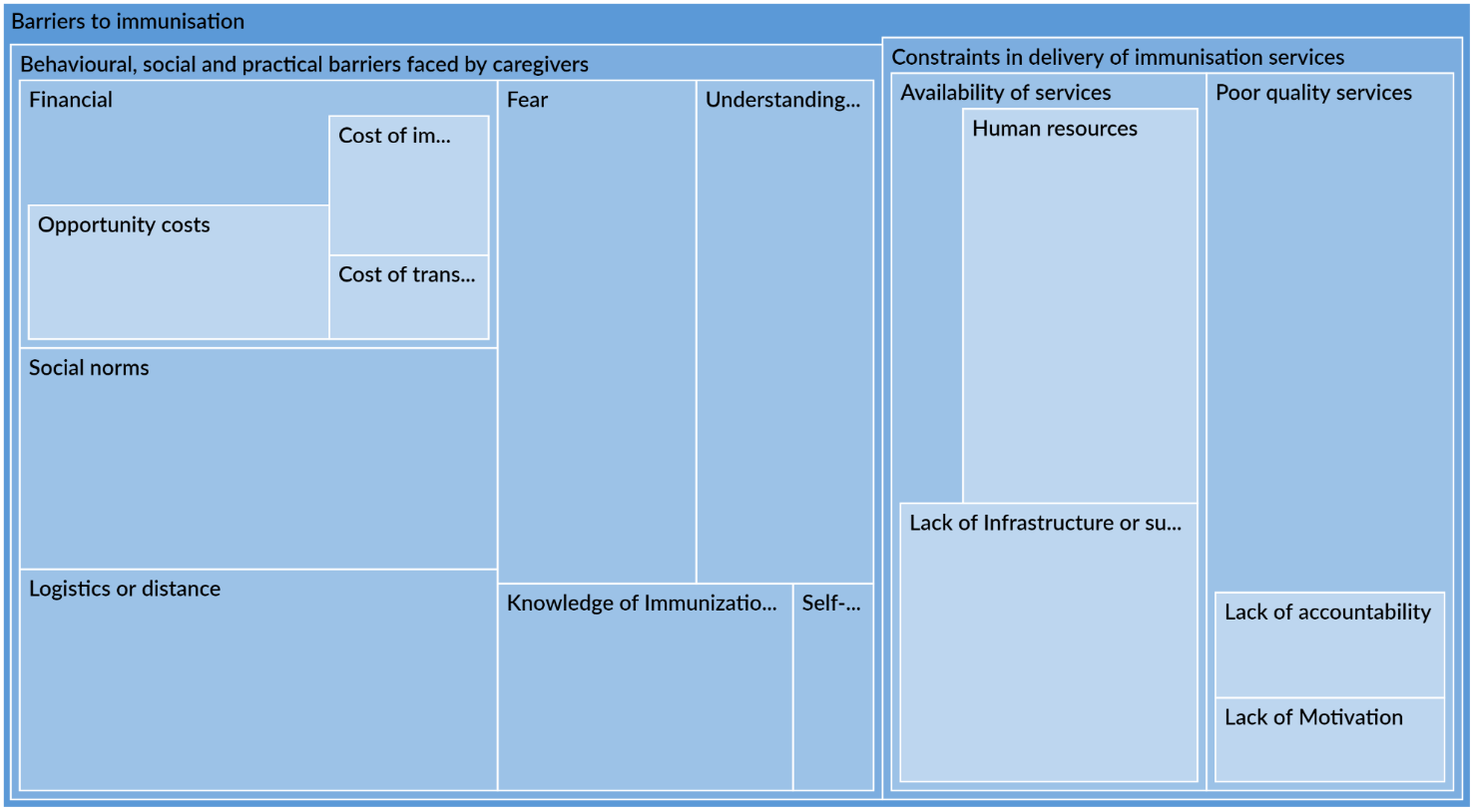
The most common barriers were broadly similar across the full analysis and the sensitivity analysis. *Behavioural, social and practical barriers faced by caregivers* were the most consistently reported barriers followed by *constraints in delivery of immunisation services* (figure 1)*.* The most common barriers faced by caregivers were unavailability of immunisation services, financial constraints and social norms.

**Figure 1 (a): Full analysis (b): Sensitivity analysis**

Facilitators of immunisation

The most common facilitators of immunisation were broadly similar across the full analysis and the sensitivity analysis. *Behavioural, social and practical factors faced by caregivers* were the most consistently reported facilitators of immunisation in a given context followed by good *delivery of immunisation services* (figure 2)*.* The most consistently reported facilitators were availability of immunisation services, enabling social norms and caregivers’ understanding of importance of immunisation.


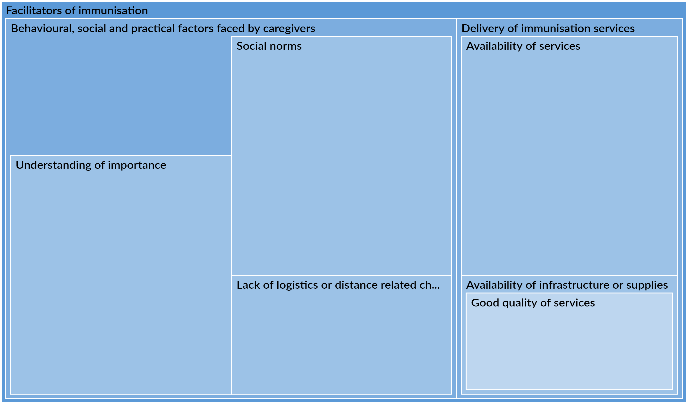

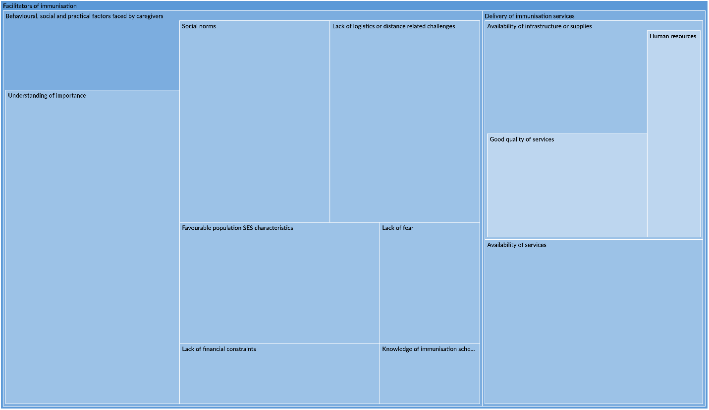


**Figure 2 (a): Full analysis (b): Sensitivity analysis**

Reasons for intervention success

The most consistently reported reasons for success were broadly similar across the full analysis and the sensitivity analysis. Success was consistently attributed to *intervention features* including leadership and supportive supervision, community engagement, health worker training, incentives and customisation to local context. Though not as common, success was also attributed to *existing or changing favourable characteristics* within a given context such as positive participant views, enabling social norms and availability of and access to good quality health services. Given that a very small subset of papers were included in the sensitivity analysis, a few themes that emerged in the full analysis were missing in the sensitivity analysis. Figure 3 provides the hierarchy charts comparing the two analyses.


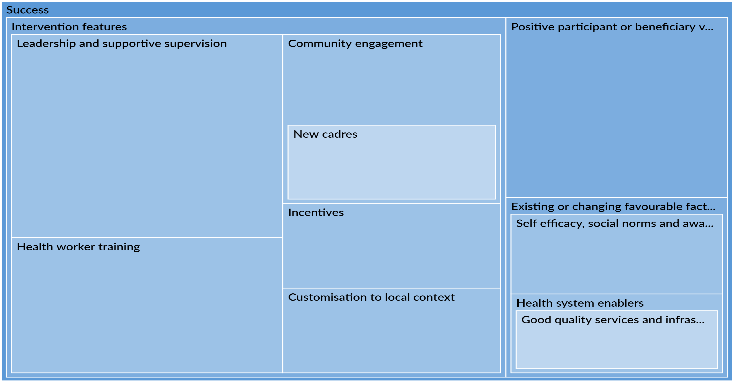

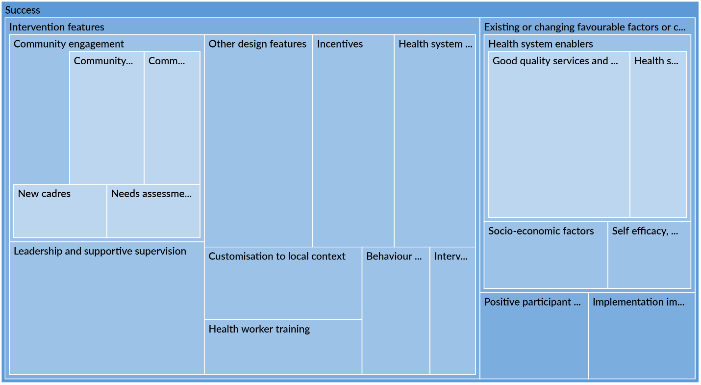


**Figure 3 (a): Full analysis (b): Sensitivity analysis**

Reasons for intervention failure

The most consistently reported reasons for failure were broadly similar across the full analysis and the sensitivity analysis. The most consistent reasons for failure were attributed to *accounting for contextual constraints* and inadequate *intervention features*. Social norms, health system related issues, political or civil unrest and high baseline coverage were some of the most common contextual reasons for failure. Among the intervention features, inadequate duration, frequency or exposure to the intervention were the most notable reasons for failure. Though not as prominently reported as compared to the full analysis, *implementation challenges* were also noted to have caused intervention failure in the sensitivity analysis. Among these, disruption due to inadequate implementation instructions and competing priorities of health workers were noted as some of the prevalent challenges.


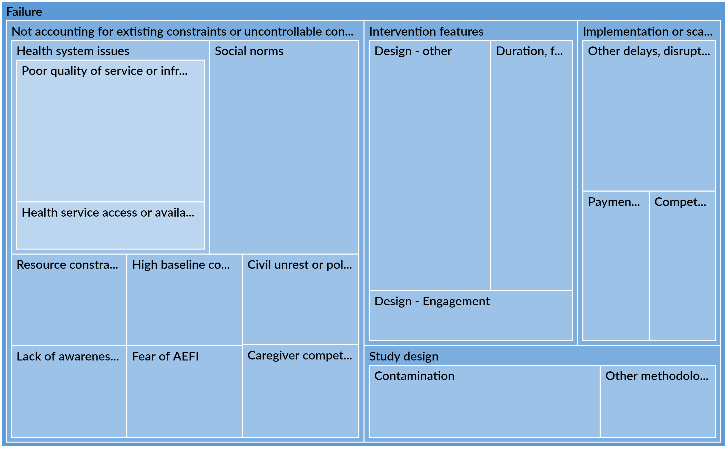

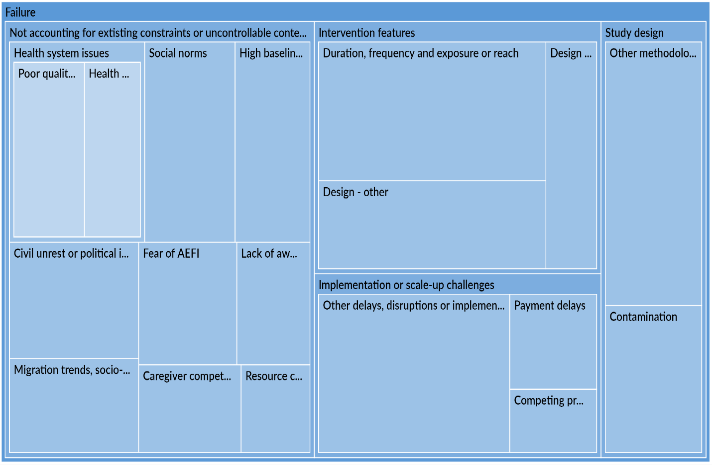


**Figure 4 (a): Full analysis (b): Sensitivity analysis**

Uptake and fidelity challenges

The most consistently reported uptake and fidelity issues were broadly similar across the full analysis and the sensitivity analysis. The most common challenges consistently reported challenges were related to administration, mobilization, fidelity, contamination and budget constraints. Administrative challenges were cited consistently. Similar to the full analysis, the primary most consistent issue was that realities on the ground forced changes to the intervention.


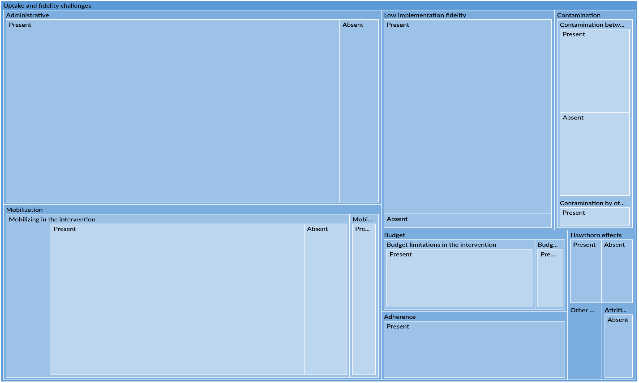

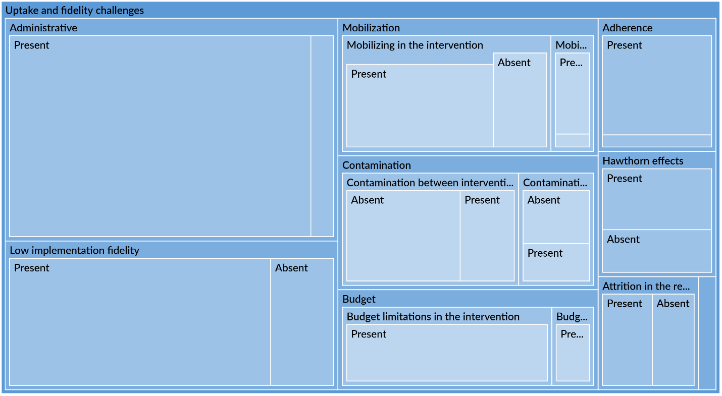


**Figure 5 (a): Full analysis (b): Sensitivity analysis**

1. Only scored as yes/no [↑](#footnote-ref-1)
