# Supplementary material for: Use of community engagement interventions to improve child immunisation in low‐ and middle‐income countries: A systematic review and meta‐analysis
Source: Campbell Syst Rev. 2022 Jul 27;18(3):e1253. doi: 10.1002/cl2.1253 (PMC9359116; doi:10.1002/cl2.1253)
Supplement: Supplementary file 2 — Supporting information. [file CL2-18-e1253-s002.docx]

1. List of abbreviations

**ANM**: Auxiliary nurse midwife

**AEFI:** Adverse Events Following Immunisation

**ASHA**: Accredited Social Health Activist

**ATE**: Average Treatment Effect

**ATET**: Average Treatment Effect on the Treated

**BCG**: Bacillus Calmette-Guerin

**CASP:** Critical Appraisal Skills Programme

**CBA**: Controlled before-after

**CEA**: Cost-effective analysis

**CHC:** Community Health Committee

**CHW**: Community health worker

**CHA:** Community health assistant

**CLCP:** Community Life Competence Process

**CPI-U**: Consumer price index for all urban consumers

**CSO**: Civil society organisations

**DALY**: Disability-Adjusted Life Years

**DID**: Difference-in-Difference

**DPT**: Diphtheria, Pertussis Tetanus

**EGM**: Evidence Gap Maps

**ESHE**: Essential Services for Health in Ethiopia

**FIC**: Full immunisation coverage

**FGD**: Focus Group Discussion

**FLWs**: Frontline workers

**HAD**: Health Development Army

**HCW**: Health Care Worker

**HEW:** Health Extension Worker

**HICs**: High-income countries

**HTR**: Hard-to-reach

**HW**: Health worker

**IAP2**: Institute of Public Participation

**IE**: Impact evaluation

**IMCI:** Integrated Management of Childhood Illness

**ITS**: Interrupted Time Series

**ITT**: Intention to Treat

**IV**: Instrumental Variable

**KB**: Khushi Baby

**LATE**: Local Average Treatment Effect

**L&MIC**: Low- and middle-income countries

**MCH**: Maternal and Child Health

**MI**: Mission Indradhanush

**MoH:** Ministry of Health

**MOV:** Missed Opportunities for Vaccination

**MNCH:** Maternal, Newborn and Child Health

**NFC**: Near Field Communication

**NGO**: Non-Governmental Organisation

**NRHM:** National Rural Health Mission

**QALY**: Quality-adjusted life year

**OPV**: Oral Polio Vaccine

**PHC**: Primary Health Care

**PRISMA**: Preferred Reporting Items for Systematic Reviews and Meta-Analyses

**PROGRESS-PLUS**: Place of residence, Race/ethnicity, Occupation, Gender, Religion, Education, Social capital, Socioeconomic status, Plus age, disability and sexual orientation

**PSM**: Propensity Score Matching

**QED**: Quasi Experimental Design

**RCT**: Randomised Controlled Trial

**RE**: Random Effects

RFP: Request For Proposal

**RIDIE**: Registry for International Development Impact Evaluations

**ROB:** Risk of Bias

**RVE**: Robust Variance Estimation

**SALT:** Stimulate, Appreciate, Learn, Transfer

**SD**: Standard Deviation

**SE**: Standard Error

**SES**: Socio-Economic Status

**SMD**: Standardised Mean Difference

**SMS**: Short Message Service

**SR**: Systematic review

**TBGI**: Team-Based Goals and Performance Based Incentives

**TIDieR**: Template for Intervention Description and Replication

**ToC**: Theory of Change

**TRL**: Traditional and religious leader

**UNICEF**: United Nations Children's Fund

**USAID:** United States Agency for International Development

**VHND**: Village Health and Nutrition Days

**VHT**: Village health team

**VRG:** Village Resource Groups

**WBOT**: Ward-based outreach team

**WHO**: World Health Organization
